# Overexpression of *VvASMT1* from grapevine enhanced salt and osmotic stress tolerance in *Nicotiana benthamiana*

**DOI:** 10.1371/journal.pone.0269028

**Published:** 2022-06-16

**Authors:** Yanyan Yu, Yong Ni, Tian Qiao, Xiaomin Ji, Jinghao Xu, Bo Li, Qinghua Sun

**Affiliations:** 1 College of Life Science, State Key Laboratory of Crop Biology, Shandong Agricultural University, Shandong, Taian, People’s Republic of China; 2 Department of Biological Engineering, Shandong Medicine Technician College, Taian, Shandong, People’s Republic of China; 3 Shandong Academy of Grape, Shandong Academy of Agricultural Sciences, Taian, Shandong, People’s Republic of China; National Taiwan University, TAIWAN

## Abstract

Salt and drought stresses are major environmental conditions that severely limit grape growth and productivity, while exogenous melatonin can alleviate the drought and salt damage to grapevines. N-acetylserotonin methyltransferase (ASMT) is the key enzyme in melatonin synthesis, which plays a critical role in regulating stress responses. However, the roles of *ASMTs* from grapevine under drought and salt stresses responses remain largely unclear. In this study, the *VvASMT1* gene was isolated from grapevine, and its physiological functions in salt and mimic drought stress tolerance were investigated. Expression pattern analysis revealed that *VvASMT1* was significantly induced by different salt and osmotic stresses. Ectopic expression of *VvASMT1* in *Nicotiana benthamiana* significantly enhanced melatonin production in transgenic plants. Compared with wild-type plants, the transgenic lines exhibited a higher germination ratio, longer root length, lower degree of leaf wilting and relative water content (RWC) under salt and osmotic stresses. In addition, under salt and osmotic stresses, overexpression of *VvASMT1* improved proline and malondialdehyde (MDA) contents, increased the activity of antioxidant enzymes and decreased the accumulation of reactive oxygen species (ROS). Taken together, our results demonstrate the explicit role of *VvASMT1* in salt and osmotic stress responses, which provides a theoretical foundation for the genetic engineering of grapevine.

## Introduction

Grapevines (*Vitis vinifera*) are one of the most economically important perennial fruit crops, both for wine and fresh consumption. It can be cultivated in a wide range of environments, but different growing conditions, such as salinity and drought stresses, can significantly affect the yield and quality of grapes, limiting grape growth and development dramatically [1]. To prevent the potential damage of such stresses, plants have evolved a series of defenses including certain metabolic, physiological, biochemical and molecular mechanisms such as changes in photosynthesis, osmotic stress, production excessive reactive oxygen species (ROS) and cell death [2]. It is well known that low concentrations of ROS can act as signaling molecules, which play a critical role in plant development, flowering and repair of cellular damage [3]. However, high concentrations of ROS in cells lead to oxidative damage in plants and even cause programmed cell death and plant death [4].

Melatonin (N-acetyl-5-methoxytryptamine), a new plant growth regulator, participates in multiple physiological actions during plant development and abiotic stress responses [5]. In recent years, melatonin has been identified as a broad-spectrum antioxidant that plays a vital role in ROS and reactive nitrogen species (RNS) scavenging [6]. Therefore, melatonin has been widely used to protect plants against various environmental stressors, including salinity [7], drought [8], extreme temperature [9], etc. For example, exogenous melatonin application significantly improved the activities of antioxidant enzymes (peroxidase [POD], catalase [CAT], superoxide dismutase [SOD] and ascorbate peroxidase [APX]) and decreased the concentrations of ROS in various plant species under abiotic stresses, such as grapevine [10], apple [11], soybean [12] and wheat [13]. Recently, an increasing number of researchers have revealed that overexpression of melatonin biosynthesis genes can also enhance plant tolerance to various environmental stresses [14–16].

Melatonin biosynthesis is catalyzed from tryptophan by four enzymatic steps involving at least six enzymes, including tryptophan decarboxylase (TDC), tryptophan hydroxylase (TPH), tryptamine 5-hydroxylase (T5H), serotonin *N*-acetyltransferase (SNAT), *N*-acetylserotonin methyltransferase (ASMT) and caffeic acid *O*-methyltransferase (COMT) [17]. First, tryptophan is decarboxylated to tryptamine by TDC; second, tryptamine is catalyzed to synthesize serotonin by T5H. In some other plant species, the first two steps of melatonin biosynthesis are inverted. That is, tryptophan is first converted into 5-hydroxytryptophan by TPH and then to serotonin by TDC [18]. Afterwards, serotonin is catalyzed into *N*-acetylserotonin by SNAT in the chloroplast and 5-methoxytryptamine by ASMT/COMT in the cytoplasm. Then, melatonin is produced by SNAT in the chloroplast or ASMT/COMT in the cytoplasm [17]. In total, at least six enzymes are involved in phytomelatonin biosynthesis, which are related to four different routes.

ASMT is believed to be the last step enzyme in the synthesis of melatonin, playing a critical role in plant melatonin synthesis. The first *ASMT* was successfully cloned from rice [19]; subsequently, *ASMT* homologs were identified from other plant species, such as apple and *Arabidopsis* [14, 20]. The expression of *OsASMT1* was induced by abscisic acid (ABA), copper and high salinity [19]. Zuo *et al*. (2014) reported that overexpression of *MzASMT1* improved the endogenous melatonin content and enhanced drought tolerance in transgenic *Arabidopsis* [14]. Zhuang (2020) found that ectopic expression of *MzASMT1* in tobacco enhanced the salt tolerance of transgenic plants [16]. In addition, the possible functions of apple *MzASMT9* were also investigated. The results revealed that the *MzASMT9* expression level was induced by salt stress, and overexpression of *MzASMT9* in *Arabidopsis* also increased the endogenous melatonin levels and improved the salt tolerance of transgenic plants [21].

Given the important role of the *ASMT* gene in melatonin synthesis and abiotic stress responses, surprisingly little is known about *ASMTs* in grapevine. In the present study, *VvASMT1* was isolated from grape, and its functions in drought and salt responses were investigated through ectopic expression of *VvASMT1* in *Nicotiana benthamiana*. To our knowledge, this is the first study to examine the function of *VvASMT1* in response to drought and salt stresses. The results improve our understanding of *VvASMT1* functions in grapevine under abiotic stress and lay a theoretical foundation for the use of genetic engineering in plant breeding.

## Materials and methods

### Plant materials and treatments

The tissue-cultured seedlings of grapevine rootstock ‘A35’ were cultured in Murashige and Skoog (MS) solid medium with 0.2 mM indole-3-butytric acid (IBA) under a 16 h light/8 h dark cycle at 24°C for one month. Then, uniform seedlings were selected for various stress treatments. For salt and drought stress treatments, the grapevine seedlings were transferred to liquid medium containing 200 mM NaCl or 200 mM mannitol, respectively. Mannitol was used to simulate drought.

For tissue-specific expression analyses, different grape organs, including young leaves, mature leaves, petioles, stems and roots, were harvested from one-year-old ‘A35’ plants grown in the plant growth room of Shandong Agricultural University, China.

*Nicotiana benthamiana* seeds were sown in soil, germinated and grown under a 16 h light/8 h dark photoperiod at 25°C. Seedlings at the three- to four-leaf stage were transplanted into pots with soil and grown in the plant growth room of Shandong Agricultural University, China. Then, uniform seedlings were selected for further experiments.

### RNA extraction, cDNA synthesis and quantitative qRT- PCR

Total RNA was extracted from the leaves of grapevine rootstock ‘A35’ and *N*. *benthamiana* using the improved CTAB method and TRIzol reagent (Invitrogen, Carlsbad, CA, USA), respectively, as previously described [3, 22, 23]. Then, RNA was used for cDNA synthesis with the PrimeScript^™^ RT reagent kit with gDNA Eraser (Vazyme, Nanjing, China). Quantitative reverse transcription PCR (qRT-PCR) was performed according to the instructions of the SYBR^®^ PrimeScript^™^ RT-PCR Kit (TaKaRa, Dalian, China) in the CFX96TM Real-Time PCR Detection System (Bio-Rad, Hercules, CA, USA). The actin1 (AY680701) and actin7 (XM_034827164) were used as internal references in the *VvASMT1* expression pattern in grapevine under NaCl and mannitol stresses, and actin (XM_033660572.1) and Tubulin (N181029A17) genes were used as internal references to determine ROS scavenging-related target genes and *VvASMT1* expression in *Nicotiana benthamiana* qRT-PCR analyses. All experiments were repeated at least three times, and all the primers used in this study are listed in S1 Table in S1 File. The data were automatically analyzed using the CFX Manager software program (version 1.1).

### *VvASMT1* cloning, vector construction and genetic transformation

To obtain *ASMT1* in grapevine, the protein sequence of apple MzASMT1 (KJ123721) was downloaded from NCBI and used as a query to search the grape genome library (https://www.genoscope.cns.fr/externe/GenomeBrowser/Vitis/). Then, sequences with higher homology were obtained, and specific primers were designed according to the nucleotide sequences of this gene (GSVIVT01020642001) (S1 Table in S1 File). The open reading frame (ORF) of *VvASMT1* was isolated by PCR amplification with the specific primers VvASMT1-F and VvASMT1-R (S1 Table in S1 File), which were synthesized by Biosune Biotechnological Company, Shanghai, China. The PCR products were purified and combined with pMD19-T vectors (TaKaRa, Dalian, China) and transformed into *E*. *coli* cells (DH5α) for sequencing (Biosune Biotechnological Company, Shanghai, China).

The full-length cDNA of *VvASMT1* was inserted into the binary vector pBI121 controlled by the Cauliflower mosaic virus (CaMV) 35S promoter. Then, the recombinant plasmid was introduced into *Agrobacterium tumefaciens* LBA4404 and transformed into *N*. *benthamiana* plants using the leaf disc method, as described by *Jia et al*. [3]. The transgenic seedlings were selected on MS agar medium containing 100 mg/L kanamycin and further confirmed by PCR and Western blot. Subsequently, three lines overexpressing *VvASMT1* (OE1, OE2, OE3) were selected by qRT-PCR for further analysis.

### Bioinformatic analysis

Amino acid and nucleotide sequences of other plant ASMT genes were retrieved from GenBank (http://www.ncbi.gov/Genbank) and aligned using the DNAMAN program (version 5.2.2). The phylogenetic tree was constructed by the neighbor-joining method using MEGA 11.

### Subcellular localization of *VvASMT1*

The ORF sequence of *VvASMT1* was fused to the N-terminus of the green fluorescent protein (GFP) gene driven by the CaMV 35S promoter. The recombinant plasmid was transformed into *Agrobacterium tumefaciens* GV3101 instead of LBA4404 because GV3101 showed higher transformation efficiency for transient transfection assay in *Nicotiana benthamiana* based on the experience of our laboratory. Then, *Agrobacterium* cells cultured overnight were resuspended in osmotic solution (10 mM MES, 10 mM MgCl_2_, and 150 mM acetosyringone) and injected into leaves from 5-week-old *N*. *benthamiana* seedlings after being placed in the dark for 3 h. The fluorescent signal of VvASMT1-GFP was detected with a laser confocal microscope (LSM 510 META; Carl Zeiss) after 2–3 days of transformation. Leaves expressing the 35S-GFP construct were used as a control.

### Salt and osmotic tolerance analysis of transgenic *Nicotiana benthamiana* lines

Seed germination and root length experiments were performed as previously described [24]. Wild-type (WT) and transgenic *Nicotiana benthamiana* (OE) seeds were disinfected and sown on 1/2 MS medium containing different concentrations (0, 100, 150 and 200 mM) of NaCl or mannitol. Seed germination was observed and counted every day. For root length analysis, WT and transgenic seeds germinated on MS medium for 5 days were transferred to MS medium containing different concentrations of NaCl and mannitol. Root length was measured after 6 days of vertical culture. Each experiment was carried out with at least three independent biological replicates.

In addition, 4-week-old WT and transgenic *Nicotiana benthamiana* seedlings were irrigated with water, 200 mM NaCl and 200 mM mannitol solution, respectively. Plant growth status was observed during the following days. After 7 days of stress treatments, the leaves were harvested for physiological index and gene expression analyses. After 10 days of stress treatments, the plants were photographed.

For physiological index measurement, relative electrolyte leakage (REL) was measured according to the protocol previously described by Jia *et al*. [3]. The free proline content was determined using the spectrophotometry PRO Kit (Solarbio Life Sciences, China). MDA was measured using the thiobarbituric acid reactive substances assay [25]. The accumulation of hydrogen peroxide (H_2_O_2_) and superoxide anions (O_2_^.-^) was detected qualitatively with DAB and NBT staining as previously described [26], and their contents were measured using O_2_^.-^ and H_2_O_2_ kits (Nanjing Jiancheng Bioengineering Institute, China). Total protein concentrations were quantified with the BCA Protein Assay Kit (Nanjing Jiancheng Bioengineering Institute, China). The activities of POD, CAT, SOD and APX were examined according to the protocols of the corresponding kits (Nanjing Jiancheng Bioengineering Institute, China). The expression levels of ROS-related genes were checked by qRT-PCR as previously described [27]. Melatonin extraction and content measurement were performed according to a previous study [23].

### Statistical analysis

Statistical significance was analyzed using Duncan’s multiple range tests with analysis of variance (ANOVA), and calculations were performed with SPSS Statistics. Significance was set at P < 0.05.

## Results

### Isolation and sequence analysis of *VvASMT1*

**Fig 1:**
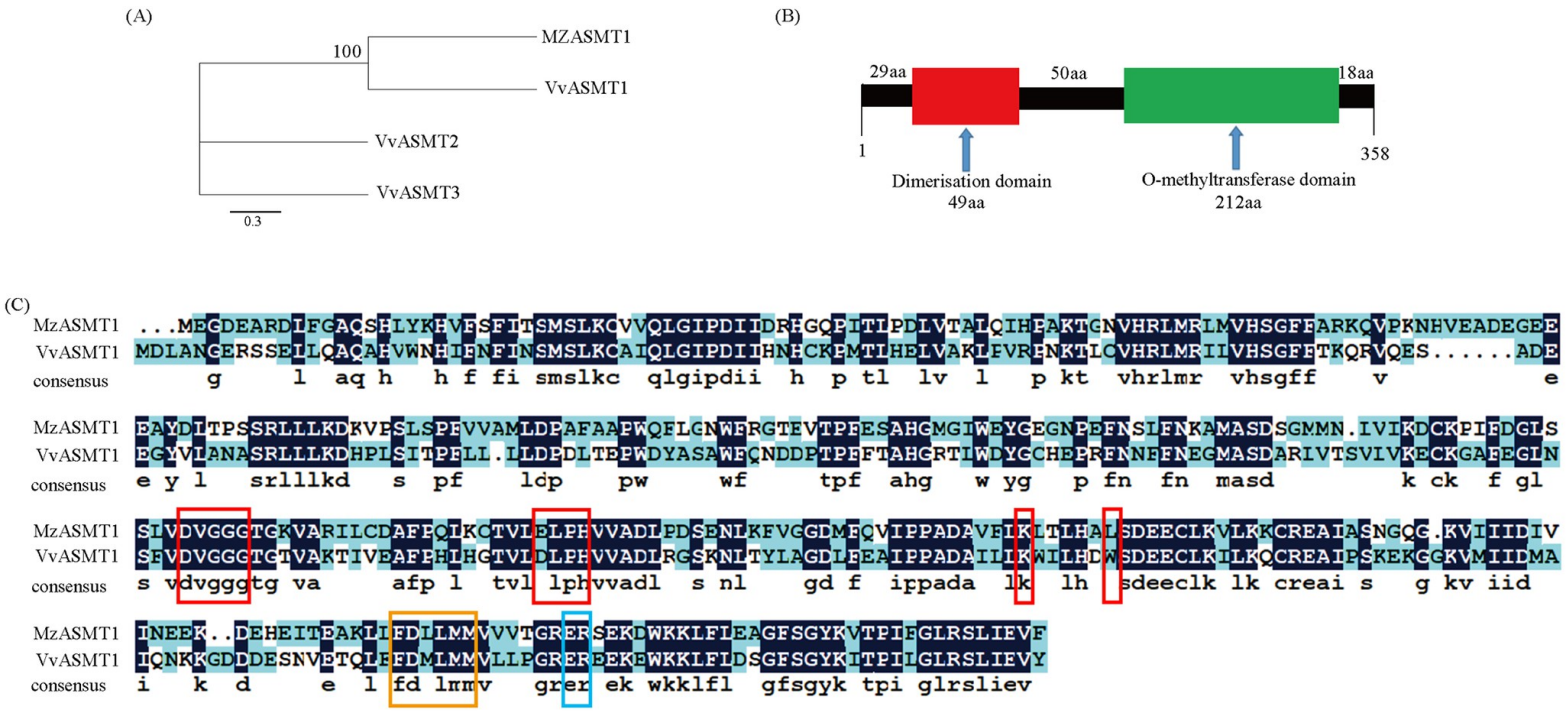
Bioinformatic analysis of the VvASMT1 protein. (A) Phylogenetic tree was constructed using the protein sequences of three grape ASMTs and apple ASMT1. (B) Schematic diagram of the domain organization of the VvASMT1. (C) Multiple sequence alignment of predicted VvASMT1 protein sequence with MzASMT1. The binding region of adenosine-L-methionine is marked by red frame, the binding region of substrate is marked by blue frame, and the catalytic region is marked by orange frame. VvASMT1 (GSVIVT01020642001), MzASMT1 (KJ123721). Vv, *Vitis vinifera*; Mz, *Malus zumi*.

To identify the melatonin biosynthesis-related gene *ASMT* in grapevine, the MzASMT1 protein sequence from apple was used as a query to perform a local BLASTp search of the grape genome. Three protein sequences with high homology to MzASMT1 were identified. Phylogenetic tree analysis revealed that VvASMT1 shared the highest homology with MzASMT1 (55.61%) in apple (Fig 1A), which contains a dimerization domain (30–78 aa) and an O-methyltransferase domain (129–340 aa) (Fig 1B and 1C). Therefore, this gene was named *VvASMT1* (GSVIVT01020642001).

**Fig 2:**
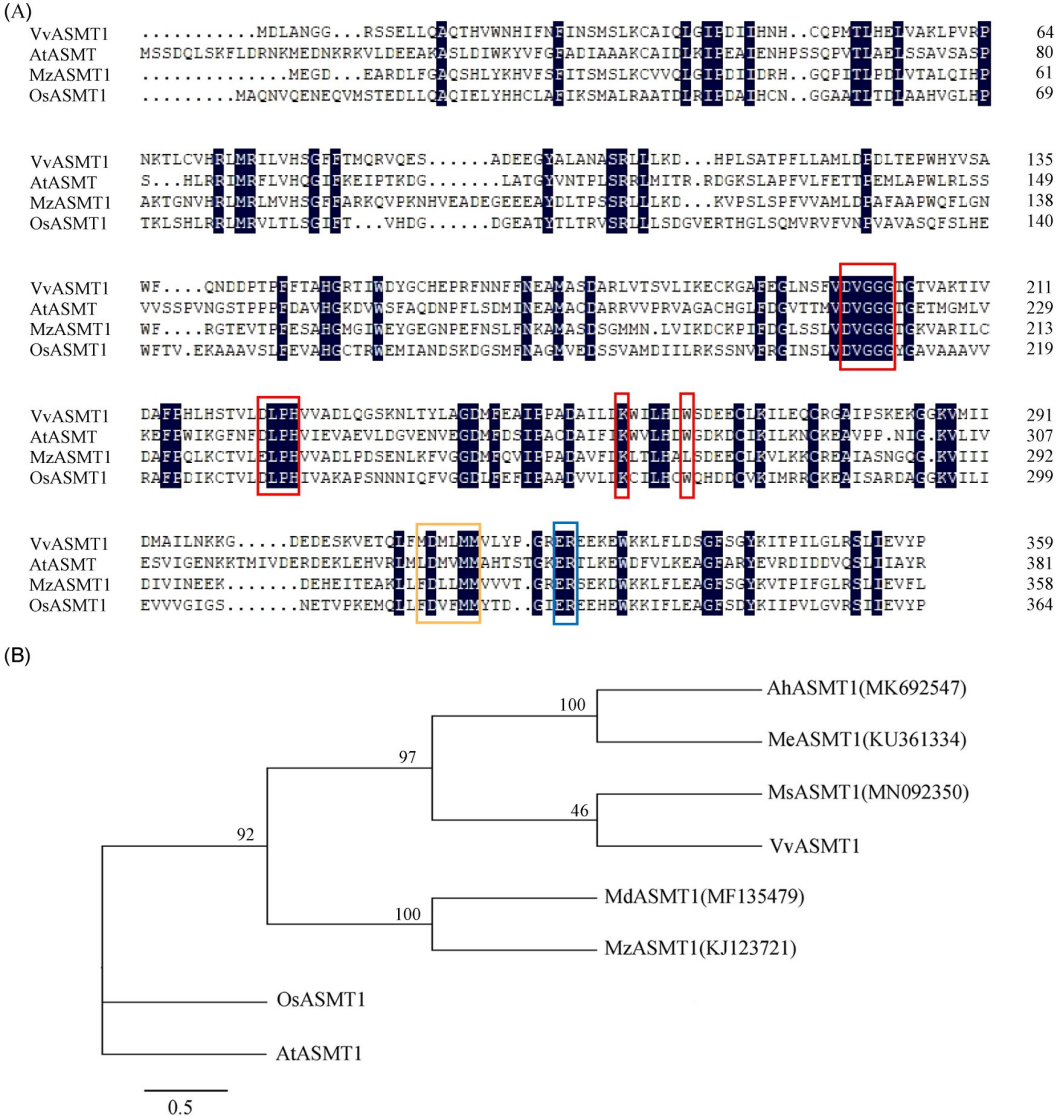
Sequence alignment and phylogenetic tree analysis of ASMT in different plants. (A) Amino acid sequence of VvASMT1 (XP_002278316) in grapevine was compared with AtASMT1 in *Arabidopsis*, OsASMT1 in rice and MzASMT1 in apple. The same amino acids are covered in black, the binding region of adenosine -L- methionine is marked by red frame, the binding region of substrate is marked by blue frame, and the catalytic region is marked by orange frame. (B) Phylogenetic relationship between VvASMT1 and ASMT1s in other plants. Each gene is followed by its ID, and the abbreviation of the species name is before the genetic name. Ah, *Arachis hypogaea*; At, *Arabidopsis thaliana*; Os, *oryza sativa*; Me, *Manihot esculenta*; Ms, *Medicago sativa*; Mz, *Malus zumi*; Vv, *Vitis vinifera*.

The ORF sequence of *VvASMT1* was obtained by PCR with specific primers (S1 Table, S1 Fig in S1 File) that encoded a protein of 358 amino acids with a predicted molecular mass of 40.49 kDa and a theoretical isoelectric point (pI) of 5.98. Multisequence alignment with other plant ASMT1s revealed that VvASMT1 contained conserved O-methyltransferase domains with S-adenosyl methionine binding sites and catalytic sites (Fig 2A). Phylogenetic tree analysis showed that the homology of ASMTs was not very high among different plant species, and VvASMT1 shared higher homology with MsASMT1 in *Medicago sativa* than with other proteins (Fig 2B). Furthermore, *VvASMT1* was overexpressed in *E*. *coli* with several histidine tags after IPTG induction, and the recombinant *VvASMT1* protein was separated by SDS-PAGE. As shown in S2 Fig in S1 File, the molecular mass of the *VvASMT1* protein was approximately 47 kD with a His-tag, which was consistent with the predicted results.

### Subcellular localization

To investigate the localization of VvASMT1, the VvASMT1-GFP fusion construct and empty GFP vector were transformed into epidermal cells of *Nicotiana benthamiana* leaves by *Agrobacterium* GV3101-mediated transient transformation. Green fluorescence signals of both the VvASMT1-GFP fusion protein and 35S-GFP were observed in the cytoplasm and nucleus by laser confocal microscopy, revealing that the VvASMT1 protein is localized in the cytoplasm and nucleus (S3 Fig in S1 File).

### Temporal and spatial expression of *VvASMT1*

The temporal and spatial expression of *VvASMT1* was determined by qRT-PCR in the grapevine seedlings. As shown in S4 Fig in S1 File, *VvASMT1* is constitutively expressed in the selected grapevine tissues, but the expression levels are different. *VvASMT1* was most highly expressed in young leaves, followed by mature leaves, stems, petioles and roots.

### *VvASMT1* expression patterns during salt and osmotic stresses

**Fig 3:**
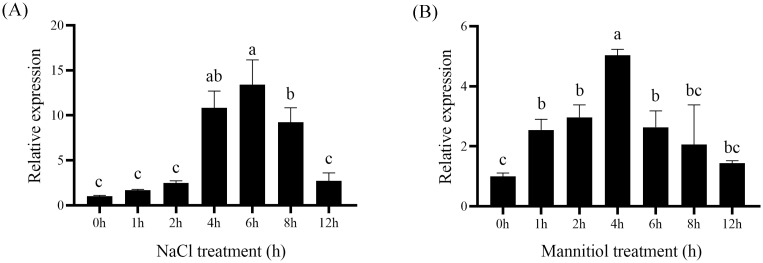
Expression pattern of *VvASMT1* in grapevine under salt and osmotic stresses. (A) NaCl, (B)Mannitol. The data were presented as the mean ± SE of three independent experiments. Different letters above the columns indicate significant differences (P < 0.05) according to Duncan’s multiple range test.

To investigate the putative role of *VvASMT1* in the response to abiotic stress, its transcriptional levels under different stresses were examined by qRT-PCR. The results showed that the expression levels of *VvASMT1* were significantly induced by salt and osmotic treatments. For NaCl treatment, the expression of *VvASMT1* was significantly upregulated and peaked after 6 h (Fig 3A). Under the mannitol treatments, *VvASMT1* expression was significantly increased, with the highest levels observed at 4 h (Fig 3B). The above results indicated that *VvASMT1* might be involved in the response to salt and osmotic stresses.

### Overexpression of *VvASMT1* in *Nicotiana benthamiana* enhanced the melatonin content and plant tolerance to salt and osmotic stresses

**Fig 4:**
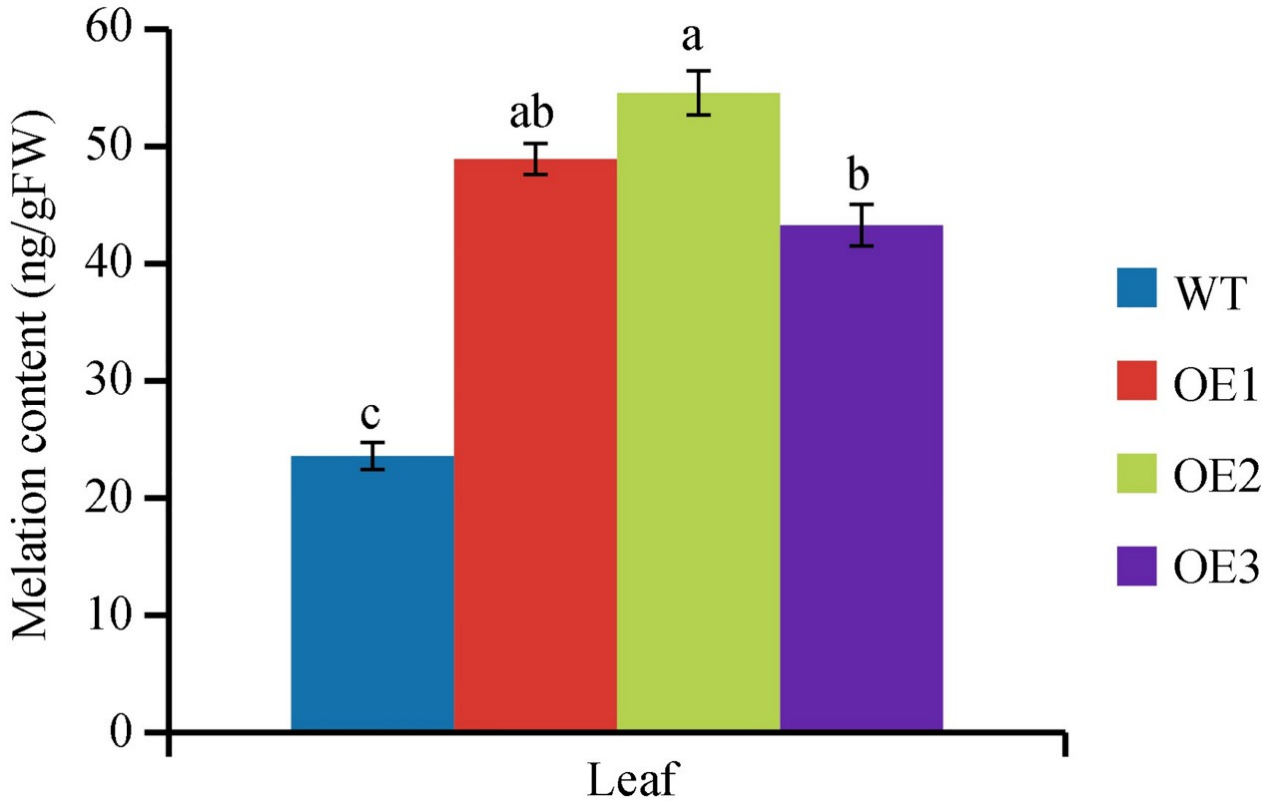
Melatonin content in WT and transgenic lines.

To further explore the role of *VvASMT1* in plant defense against abiotic stress, *VvASMT1* driven by the 35S promoter was genetically transformed into *N*. *benthamiana*, and transgenic lines were selected by kanamycin and confirmed by PCR and western blot (S5 Fig in S1 File). Then, three representative transgenic lines (OE1, OE2 and OE3) with similar expression levels of *VvASMT1* were selected for further experiments. Given the important role of *VvASMT1* in melatonin synthesis, we measured the melatonin content in WT and transgenic plants. The results showed that the melatonin content was significantly higher (43–55 ng/gFW) in OE plants than in WT plants (23.59 ng/gFW) (Fig 4), indicating that *VvASMT1* overexpression prompted melatonin synthesis in transgenic plants.

**Fig 5:**
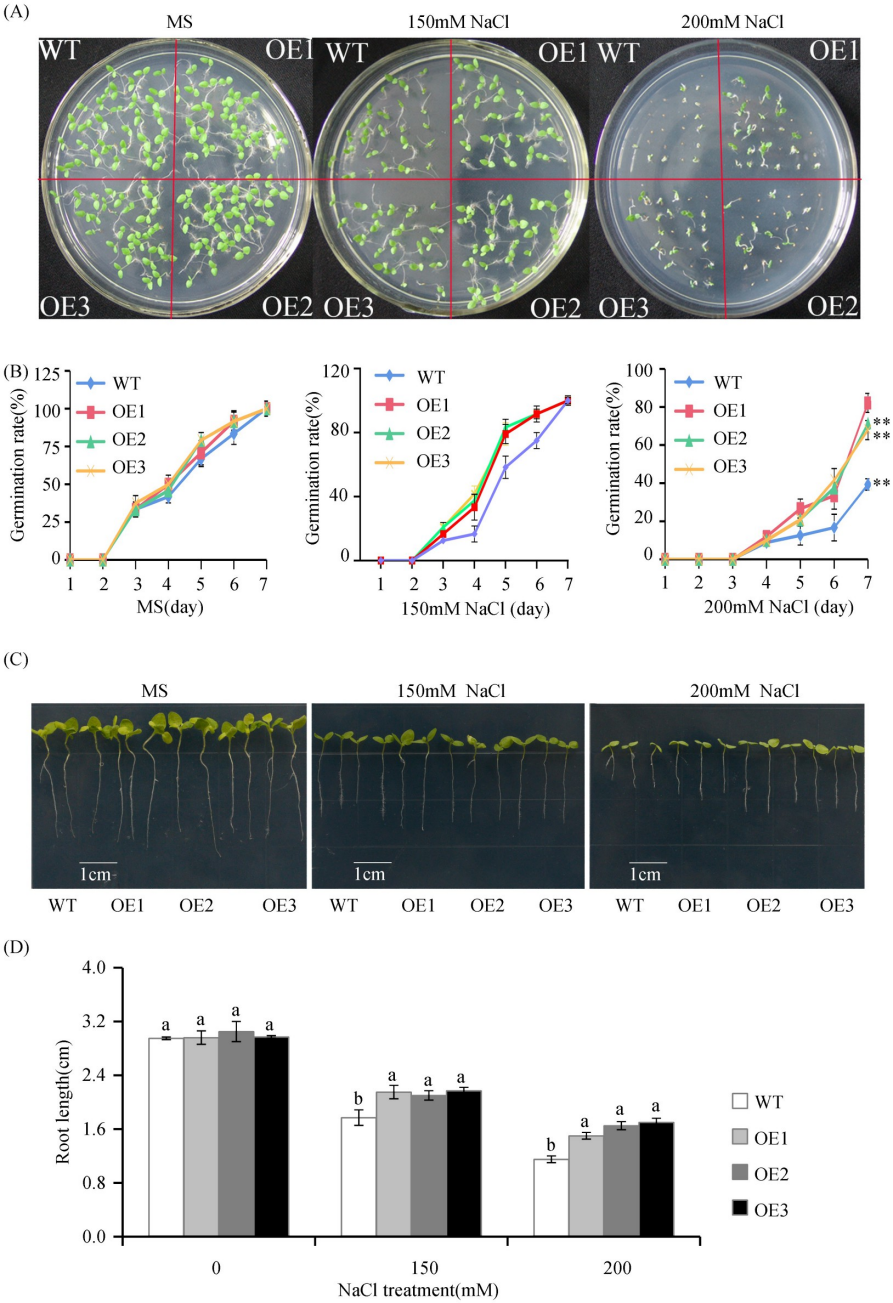
Seed germination and growth of WT and *VvASMT1* overexpressing plants under salt stress. (A) The seed germination phenotype, (B) The germination rate, (C) The root phenotype, (D) The root length. The data were presented as the mean ± SE of three independent experiments. Different letters above the columns indicate significant differences (P < 0.05) according to Duncan’s multiple range test.

**Fig 6:**
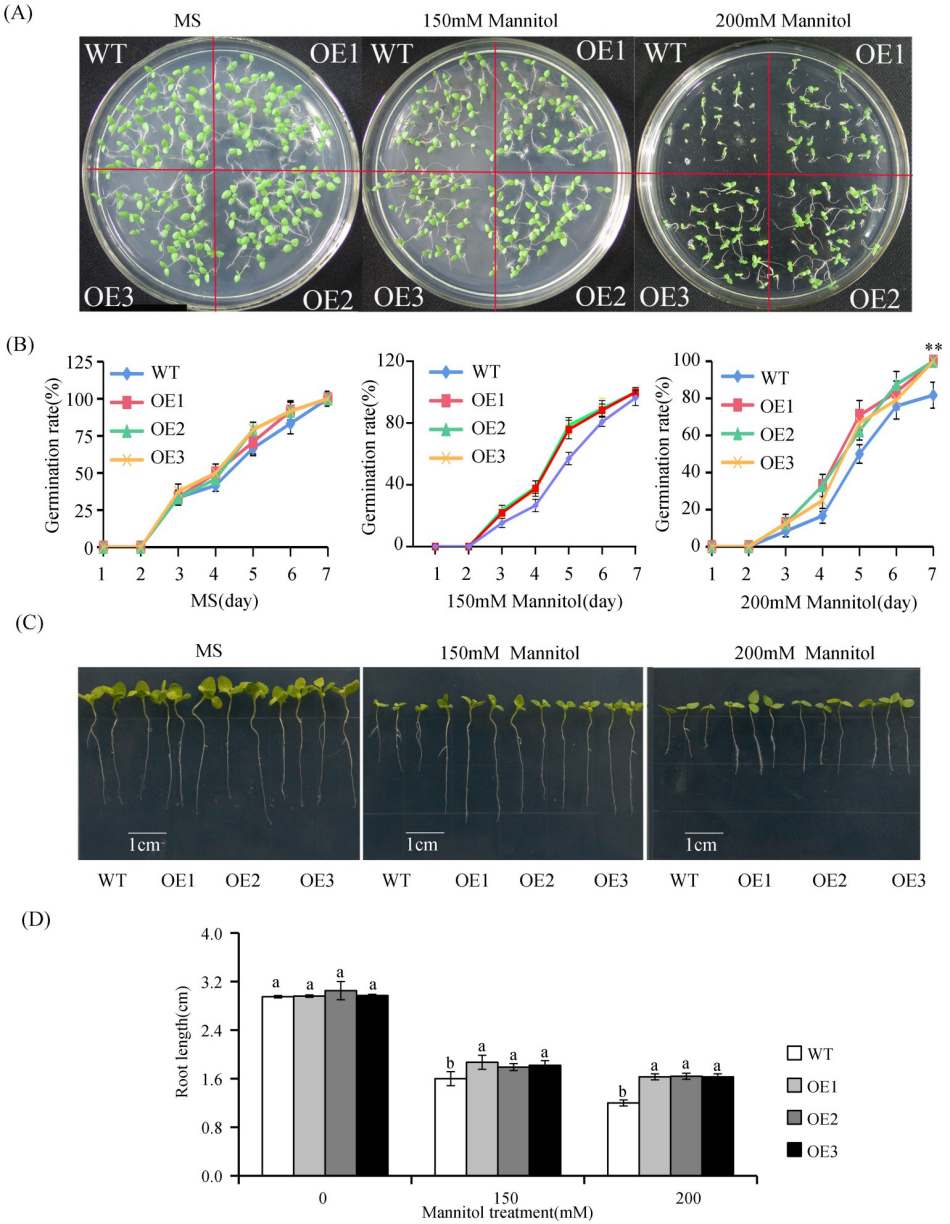
Seed germination and growth of WT and *VvASMT1* overexpressing plants under mannitol treatment. (A) The seed germination phenotype, (B) The germination rate, (C) The root phenotype, (D) The root length. The data were presented as the mean ± SE of three independent experiments. Different letters above the columns indicate significant differences (P < 0.05) according to Duncan’s multiple range test.

It was previously reported that exogenous melatonin could enhance the resistance of plants to abiotic stress [28–30]. To investigate whether *VvASMT1* overexpression can improve salt and osmotic tolerance in *Nicotiana benthamiana*, the growth phenotype was observed during the germination stage in this study. Under normal conditions, no obvious difference was found in the germination rate between the WT and transgenic plants. However, when treated with 150 mM or 200 mM NaCl in 1/2 MS agar medium, the seed germination rate of OE lines was significantly higher than that of WT plants (Fig 5A and 5B). On the stress medium with 150 mM NaCl, the germination rate of the OE seeds reached about 80% compared with 58.3% for WT seeds after treatment for 5 days. Moreover, with the increase of NaCl concentration in medium, the difference in germination rate between transgenic and WT seeds was even more significant. As shown in Fig 5B, on the medium containing 200 mM NaCl, the germination rates of the OE seeds reached more than 60%, while that of WT was only 40%. Concurrently, root length of WT and transgenic seedlings was measured after 6 days of vertical culture on 1/2MS medium containing 0, 150 and 200 mM NaCl, respectively. The root length of OE lines was significantly longer than that of WT plants under salt stress (Fig 5C and 5D). Similarly, after mannitol treatments, the seeds of the OE lines displayed significantly higher germination rates and longer root lengths than those of the WT lines (Fig 6). These results suggested that *VvASMT1* was involved in the plant defense against salt and osmotic stresses.

**Fig 7:**
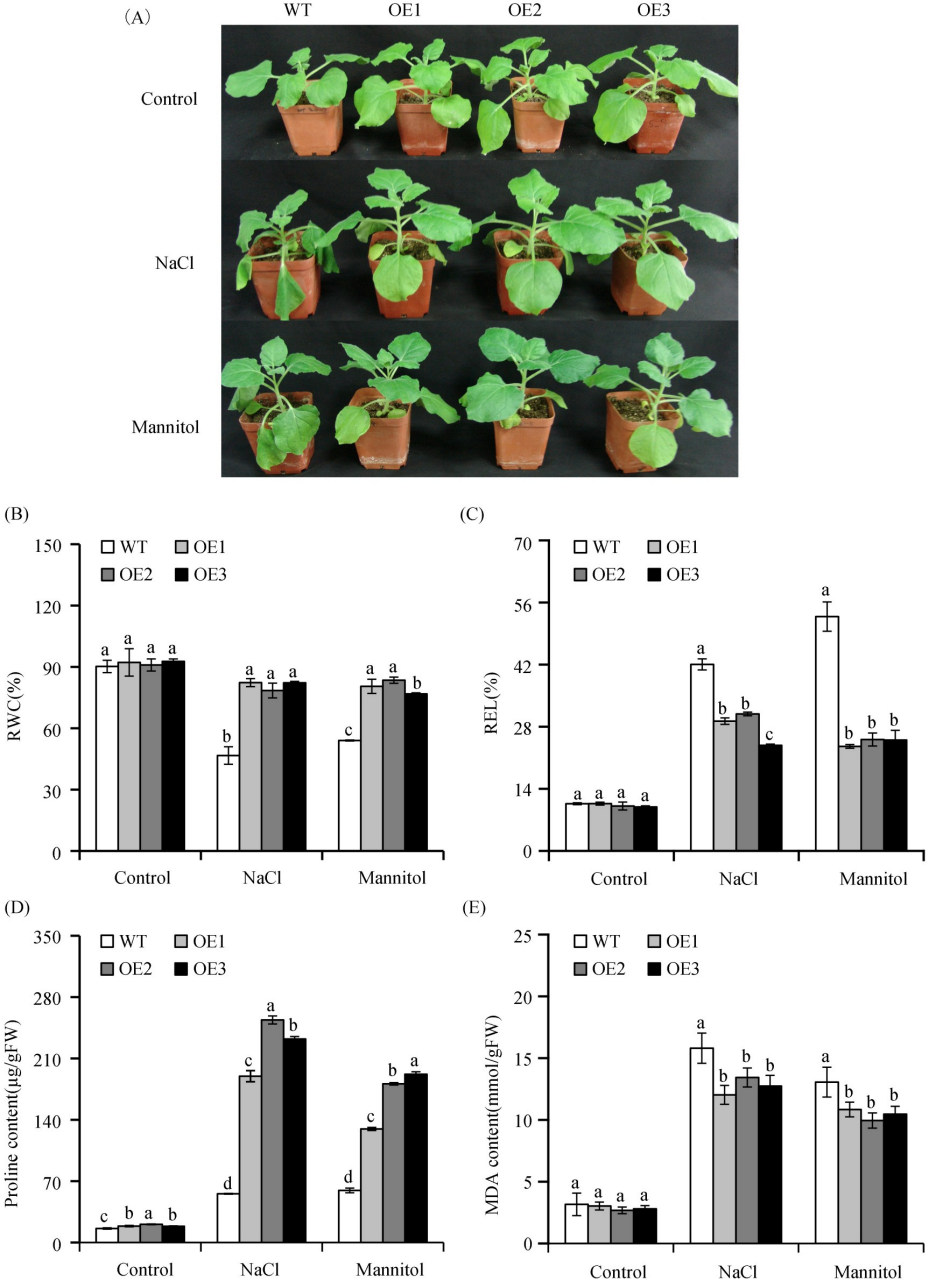
Phenotypes and physiological indexes of *Nicotiana benthamiana* under salt and osmotic stresses. (A) The phenotypes of WT and transgenic plants were treated with NaCl and mannitol for 10 days, (B-E) were respectively the RWC, REL, PRO and MDA content in WT and transgenic plants under NaCl and mannitol for 10 days, respectively.

To confirm that *VvASMT1* overexpression enhanced the salt and osmotic tolerance of plants during the vegetative growth stage, 4-week-old WT and OE plants were exposed to 200 mM NaCl or 200 mM mannitol treatments for 10 days, respectively. Compared with the transgenic plants, the leaves of the WT appeared wilted and dropped under high salt and osmotic conditions (Fig 7A). Then, the levels of RWC, REL, proline and MDA were measured in both WT and transgenic plants. Under normal conditions, these examined physiological indexes were similar for the WT and transgenic plants. Under salt and osmotic stress, the RWC of both the WT and OE plants decreased, but the RWC of OE plants (about 80%) was obviously higher than that (lower than 60%) of WT plants (Fig 7B). In contrast, the REL of the OE and WT plants was all increased under the same salinity and osmotic conditions, but the REL of the OE plants was considerably lower than that of the WT plants (Fig 7C). Under salt stress, the REL of the WT plants increased to 42.04%, while that of the OE plants was only about 28%. Similarly, under mannitol treatment, the REL of the WT plants was 52.82%, whereas that of the OE plants was below 26%. Proline is one of the primary osmotic regulation substances in plant cells under salt and osmotic stress. Under normal condition, the proline contents of both the OE and WT plants were at a low level, and only slight difference was observed. However, when exposed to salt and osmotic stress, significant differences of the proline content were observed between the OE and WT plants. In addition, under salt and osmotic treatment, the MDA content of OE plants was obviously lower than that of the OE plants (Fig 7D and 7E). Taken together, overexpression of *VvASMT1* not only enhanced melatonin production but also improved the salt and osmotic tolerance of transgenic plants.

### Overexpression of *VvASMT1* activated the antioxidant system of plants under salt and osmotic stresses

**Fig 8:**
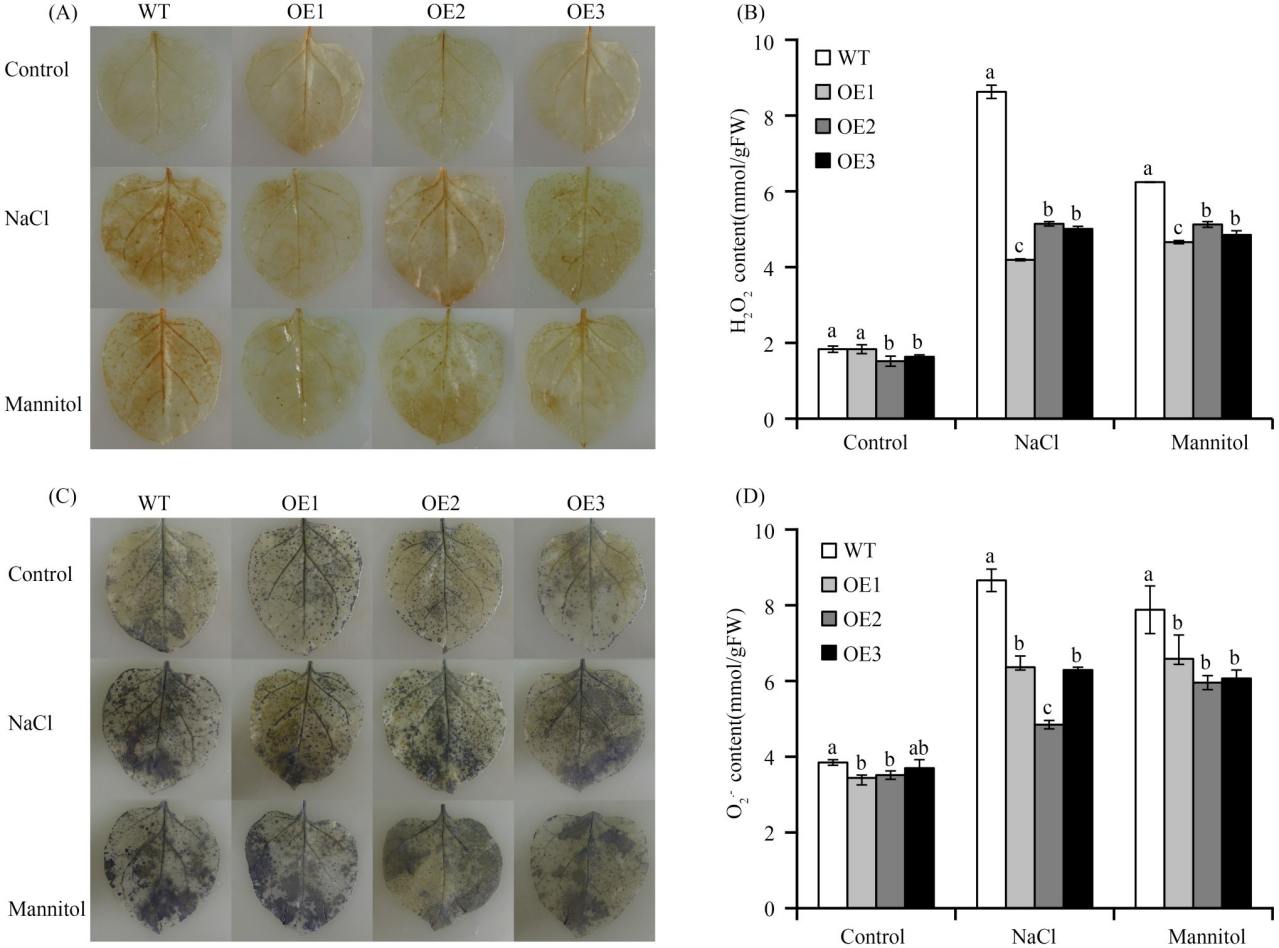
ROS levels of transgenic plants under high salt and osmotic stress conditions. (A) DAB staining for detection of H_2_O_2_ in WT and transgenic plant leaves after salt and mannitol treatment, (B) The content of H_2_O_2_ in WT and transgenic lines after salt and mannitol treatment, (C) NBT staining for detection of O^2·-^ in WT and transgenic plant leaves after salt and mannitol treatment, (D) The content of O^2·-^ in WT and transgenic lines after salt and mannitol treatment.

**Fig 9:**
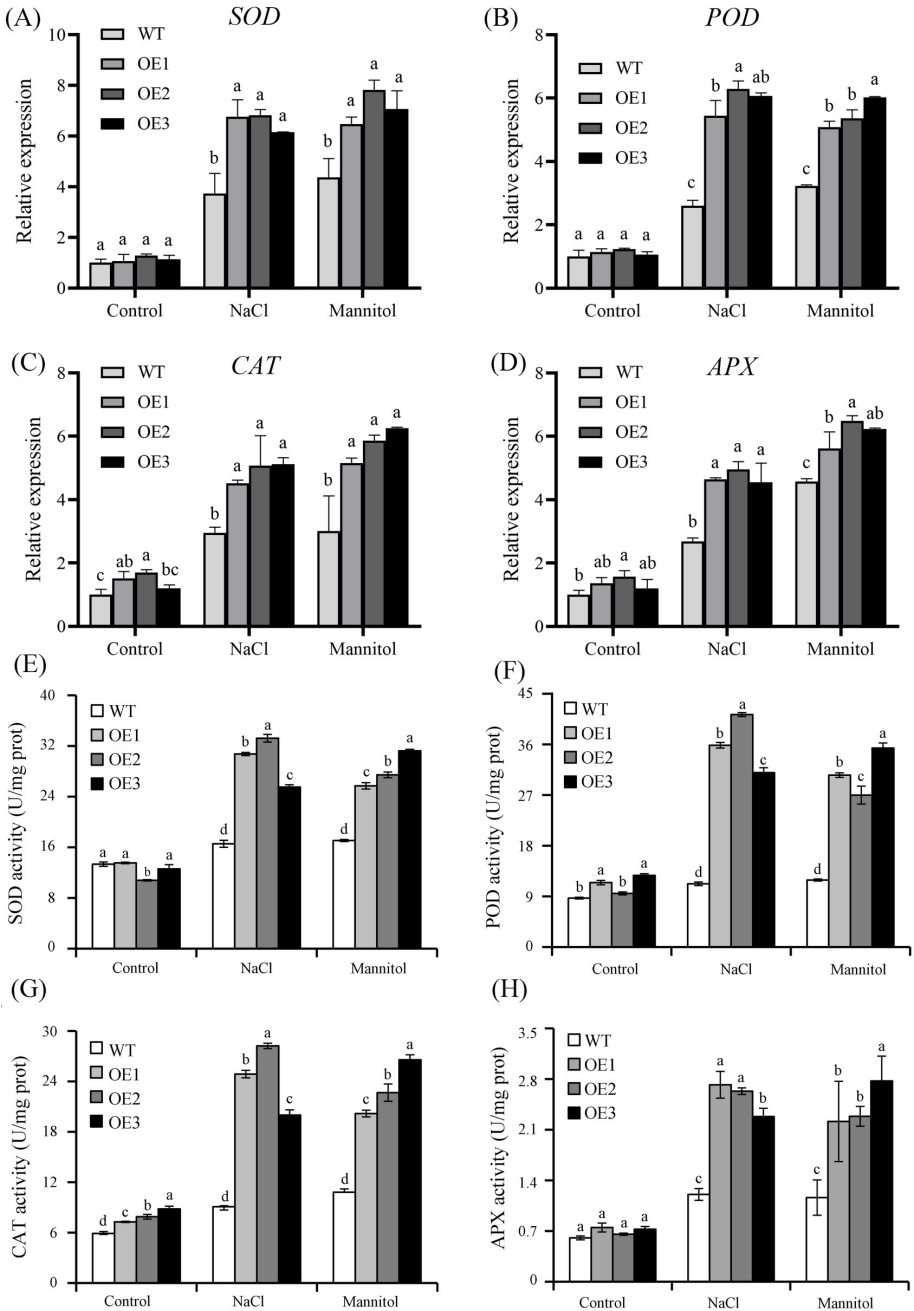
The expression of ROS scavenging-related genes and the enzyme activities in WT and transgenic plants under salt and osmotic stresses. (A-D) The expression of ROS scavenging-related genes (SOD, POD, CAT, APX) in WT and transgenic lines, (E, F) The activities of SOD, POD, CAT and APX enzyme in WT and transgenic lines. The data are presented as the mean ± SE of three independent experiments. Different letters above the columns indicate significant differences (P < 0.05) according to Duncan’s multiple range test.

To investigate the mechanisms that potentially regulate *VvASMT1*-induced salt and drought resistance, we measured the accumulation of H_2_O_2_ and O_2_^.-^. Under normal conditions, the accumulation of O_2_^.-^ and H_2_O_2_ varied slightly in WT and OE plants. However, after the NaCl or mannitol treatment, the leaves of OE lines accumulated lower levels of H_2_O_2_ and O_2_^.-^ than WT leaves (Fig 8A and 8C), and the contents of H_2_O_2_ and O_2_^.-^ in OE lines were significantly lower than those in WT plants (Fig 8B and 8D). Furthermore, the expression levels of genes encoding ROS-scavenging enzymes, including SOD, POD, CAT, and APX, were determined. As shown in Fig 9A–9D, the expression levels of *SOD*, *POD*, *CAT* and *APX* were significantly higher in the transgenic lines than in the WT lines under salt and osmotic stresses. To confirm the results, the activities of ROS-scavenging enzymes, including SOD, CAT, POD and APX, were evaluated. Under normal conditions, the activities of these examined ROS-scavenging enzymes varied slightly in the WT and transgenic lines. However, under salt or osmotic stresses, the SOD, POD, CAT and APX activities were much higher in the OE lines than in the WT lines (Fig 9E–9H), which were consistent with the change trend in the expression of their corresponding genes. All the above results suggested that the overexpression of *VvASMT1* activated the antioxidant system, indicating that *VvASMT1* might positively regulate salt and osmotic stresses by mediating the antioxidant system.

## Discussion

Salt and drought stresses are serious abiotic stresses that severely limit plant growth and productivity. Extensive studies have reported that exogenous melatonin plays crucial roles in enhancing salt and drought tolerance in various plant species [29, 31–33]. Grapevine is an important economic fruit crop, which is deeply loved by people because of its delicious taste and high nutritional value. Previous studies have proved that the application of exogenous melatonin could effectively improve the tolerance to abiotic stresses in grapevine, such as drought [34], salt [35], and senescence [36]. However, few studies have focused on the effects of endogenous melatonin on salt and osmotic tolerance in grapevine.

*ASMT* is considered the rate-limiting enzyme of melatonin synthesis [37–39]. To date, some *ASMTs* have been isolated from several plants [14, 19, 20], but the homology of their protein sequences is not very high. For instance, the protein sequence of apple *MzASMT1* has only 39.7% homology with the amino acid sequence of the rice OsASMT1 protein [14]. The amino acid sequence of *Arabidopsis* AtASMT1 showed only 31% identity to rice OsASMT1. This finding indicated that it is also possible to find ASMTs when the protein sequence similarity is below 40% in other plant species [20]. Recently, several studies have revealed that overexpression of ASMT genes in plants could promote endogenous melatonin production [14, 20, 21], which suggest that ASMT could participate in the synthesis of melatonin in plants. However, whether orthologous ASMT genes performing the same function exist in grapevine remains unclear. In this present study, a grape ASMT gene (*VvASMT1*) were identified and cloned from the grape rootstock A35, which shared higher homology with MsASMT1 (57.78%) (Fig 2B), whereas only 38.65% identity to the protein sequence of OsASMT1. Moreover, *VvASMT1* contained conserved O-methyltransferase domains with S-adenosyl methionine binding sites and catalytic sites of ASMTs (Fig 2A) as previous study reported [19], suggesting that it was an ASMT protein. Further study revealed that overexpression *VvASMT1* in *Nicotiana benthamiana* significantly enhanced the melatonin content in OE lines, which indicated that VvASMT1 was involved in melatonin synthesis.

Previous studies revealed that *ASMTs* are involved in plant responses to abiotic stresses. For instance, overexpression of *MzASMT1* enhanced drought tolerance in transgenic *Arabidopsis* and improved salt tolerance in tobacco by altering ROS balance [14, 16]. Similarly, overexpression of *MzASMT9* in *Arabidopsis* positively regulated the salt tolerance of transgenic plants [21]. However, the functions of *VvASMT1* from grapevine under salt and drought stresses responses remain unknown. Our results showed that the expression of *VvASMT1* was induced by salt and mimic drought stresses, implying that *VvASMT1* may be a salt and drought stress responsive gene. To further confirm this hypothesis, *VvASMT1* was transferred into *Nicotiana benthamiana*. It was found that the transgenic plants showed better phenotypic morphology than WT lines, such as higher germination rates, longer root lengths (Figs 5 and 6), lower degrees of leaf wilting under salt and osmotic stresses, accompanied by higher RWC and proline contents, and lower REL and MDA contents under salt and mannitol treatment (Fig 7). All the above results suggested that increasing melatonin content in *VvASMT1* overexpressing lines can effectively enhancing the growth and tolerance to salt and mimic drought stress in *Nicotiana benthamiana*.

Environmental stresses disrupt the normal homeostasis of plants, promoting ROS generation. Low concentrations of ROS are signaling molecules that can lead to the repair of cellular damage resulting from stresses [40–42]. However, excessive ROS lead to oxidative damage and repress the normal growth of plants [43]. To minimize the damage of ROS, plant cells simultaneously activate a series of response mechanisms, including ROS-scavenging enzymes (POD, SOD, CAT and APX) and non-enzymatic antioxidants (ascorbate acid [AsA], glutathione [GSH], melatonin), which play an important role in regulating ROS homeostasis. It was previously shown that exogenous melatonin application may help plants scavenge excess ROS and reduce oxidative damage caused by abiotic stress [8, 44–46]. Recently, several researches revealed that overexpression of melatonin synthesis genes, such as ASMT, SNAT and COMT, can increase the content of endogenous melatonin and improve salt and drought resistance by regulating ROS scavenging [14, 16]. For instance, overexpression of *SlCOMT1* in tomato significantly improved the content of endogenous melatonin and alleviated ROS accumulation under salt stress [47]. In tobacco, overexpression of *MzASMT1* positively regulates salt tolerance in transgenic plants by altering ROS balance and stress-responsive gene regulation [16]. In this present study, overexpression of *VvASMT1* also promoted the production of endogenous melatonin, and reduced the accumulation of ROS and MDA than WT under salt and osmotic stresses. In plants, antioxidant enzymes (SOD, POD, CAT and APX) play the important role in ROS scavenging. Our results revealed that the activities of SOD, CAT, POD and APX in OE lines were significantly higher than those in the WT lines during salt and osmotic stress. Consistent with these results, the expression levels of these ROS scavenging genes were also obviously higher in the transgenic lines. Taken together, these results indicated that melatonin accumulation in transgenic lines could improve the ROS scavenging ability under salt and osmotic stresses. However, the mechanism regarding the scavenging of reactive oxygen species by melatonin is complex. A previous study reported that melatonin, as a broad-spectrum antioxidant, not only directly scavenges ROS but also increase the levels of antioxidants and the activities of related enzymes to scavenge ROS [48]. Under abiotic stress, the initial H_2_O_2_ burst is always followed by the expression of the melatonin synthase genes (*TDC*, *SNAT*, *ASMT* and *COMT*) burst, which lead to the accumulation of endogenous melatonin. Subsequently, the transcript level and the activity levels of the antioxidant enzymes were up-regulated and increased [5]. In other words, ROS can enhance the accumulation of melatonin, which in turn can directly and indirectly scavenge the ROS.

In conclusion, the function of *VvASMT1* was investigated in *Nicotiana benthamiana* under salt and osmotic stresses. The transcription level of *VvASMT1* was significantly induced by salt and osmotic stresses. Overexpression of *VvASMT1* in *Nicotiana benthamiana* enhanced the accumulation of melatonin and improved the salt and osmotic stress tolerance of transgenic plants. In addition, the transgenic lines displayed lower accumulation of ROS and MDA, and higher proline contents and antioxidant enzyme activities. All these results indicated that *VvASMT1* might play a critical role in melatonin production and improve plant resistance to salt and osmotic stresses by mediating the antioxidant system.

## Supporting information

S1 File (DOCX)

S1 Data (ZIP)
