# Supplementary material for: Overexpression of VvASMT1 from grapevine enhanced salt and osmotic stress tolerance in Nicotiana benthamiana
Source: PLoS One. 2022 Jun 16;17(6):e0269028. doi: 10.1371/journal.pone.0269028 (PMC9202941; doi:10.1371/journal.pone.0269028)

**Supplementary Material**

S1 Table. Primers used in this investigation.

| Primers Name | Sequences（5′-3′） | Function |
| --- | --- | --- |
| VvASMT1-F | ATGGATTTGGCAAATGGTGAGAG | Full length cDNA primer |
| VvASMT1-R | TCAAGGATAAACCTCAATAAGAGAC | Full length cDNA primer |
| VvASMT1-ZH-F | GAGCTCATGGATTTGGCAAATGGTGAGAG (Sac I) | Construct vector primer |
| VvASMT1-ZH-R | GGATCCAGGATAAACCTCAATAAGAGAC (BamH I) | Construct vector primer, |
| VvASMT1-YH-F | GGATCCATGGATTTGGCAAATGGTGA (BamH I) | Construct vector primer |
| VvASMT1-YH-R | GTCGACAGGATAAACCTCAATAAGAG (Sal I) | Construct vector primer |
| VvASMT1-GFP-F | GGATCCATGGATTTGGCAAATGGTGAGAG (BamH I) | Construct vector primer |
| VvASMT1-GFP-R | GGTACCAGGATAAACCTCAATAAGAGAC (Kpn I) | Construct vector primer |
| 35S-F | GGGTGCATCATCATCTTCTGTTG | 35S promoter primer |
| 35S-R | GAAGACGTGGTTTTAACG | 35S promoter primer |
| VvASMT1-Q-F | AAGAAAGTGCGGACGAAGAG | qRT-PCR primers |
| VvASMT1-Q-R | TCCGTTAAATCTGGGTCGAG | qRT-PCR primers |
| Nbβ-actin F | TGGACTCTGGTGATGGTGTC | qRT-PCR primers |
| Nbβ-actin R | CCTCCAATCCAAACACTGTA | qRT-PCR primers |
| NtTubulin-F | AGATGTTCCGTCGTGTCAGTG | qRT-PCR primers |
| NtTubulin-R | TGCTTCCTCTTCATCCTCATATCC | qRT-PCR primers |
| Vvβ-actin F | TCAGGAAGGACCTCTATGGC | qRT-PCR primers |
| Vvβ-actin R | CTGTGGACAATGGATGGACC | qRT-PCR primers |
| Vvactin 1-F | GACAATGGATGGACCAGATTCA | qRT-PCR primers |
| Vvactin 1-R | CTTGCATCCCTCAGCACCTT | qRT-PCR primers |
| NbCAT-F | CACAGCCACGCTACTCAAGAC | qRT-PCR primers |
| NbCAT-R | CCACCCACCGACGAATAAAG | qRT-PCR primers |
| NbAPX-F | GGAGTGGTTGCTGTTGAAGTC | qRT-PCR primers |
| NbAPX-R | GGAGAGCCTTGTCGATGG | qRT-PCR primers |
| NbSOD-F | CAACTCCACGGCTTCCAGAC | qRT-PCR primers |
| NbSOD-R | TGGGTCCTGATTAGCAGTGGT | qRT-PCR primers |
| NbPOD-F | CTCCATTTCCATGACTGCTTTG | qRT-PCR primers |
| NbPOD-R | GTTGGGTGGTGAGGTCTTT | qRT-PCR primers |

S1 Fig. Isolation and structural diagram of *VvASMT1*. (A) Isolation of full-length cDNA of *VvASMT1* by PCR from grapevine. M, marker DL2000; 1 and 2, PCR amplification production of *VvASMT1*, (B) Structural Gene structural diagram of *VvASMT1*. The dark gray boxes indicate the exons, and the line indicate the intron. The light gray boxes indicate 3´UTR and 5´UTR.


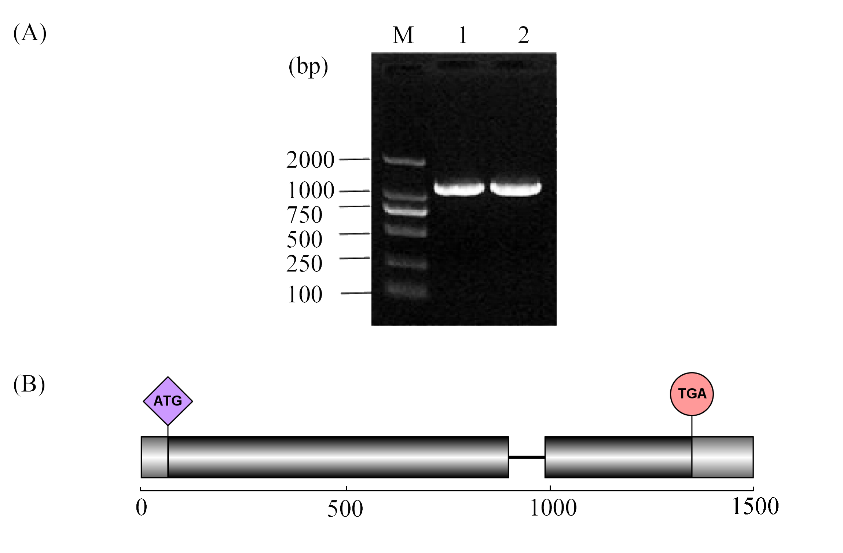


S2 Fig. Induction of theVvASMT1 recombinant protein in *E. coli.* The recombinant protein was separated by SDS-PAGE and marked by the red box, M：protein molecular weight marker; Lane 1, uninduced expression of pET30a (+); Lane 2, uninduced expression of recombinant VvASMT1 protein; Lane3-8, expression of recombinant VvASMT1 protein induced by IPTG.


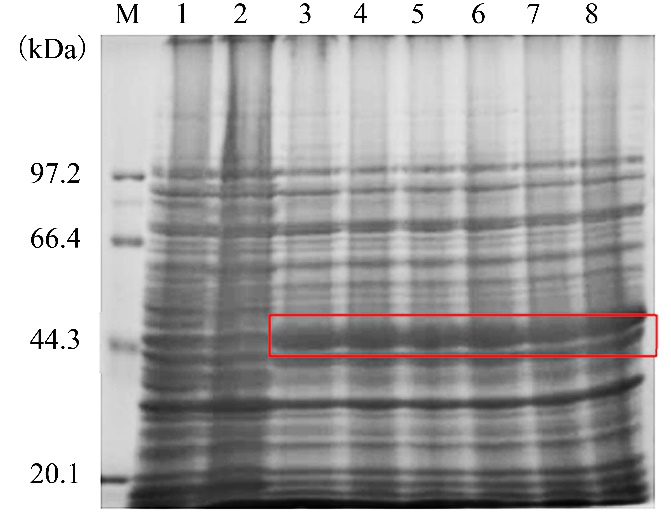


S3 Fig. Subcellular localization of VvASMT1 in *Nicotiana benthamiana*. (A) Schematic diagram of 35S-VvASMT1-GFP fusion construct and 35S-GFP construct, (B) Subcellular localization of VvASMT1-GFP fusion protein. 35S::VvASMT1-GFP construct was transformed into *Nicotiana benthamiana* leaves and was examined in the epidermal cells at 48 h after the transformation by confocal fluorescence microscopy.


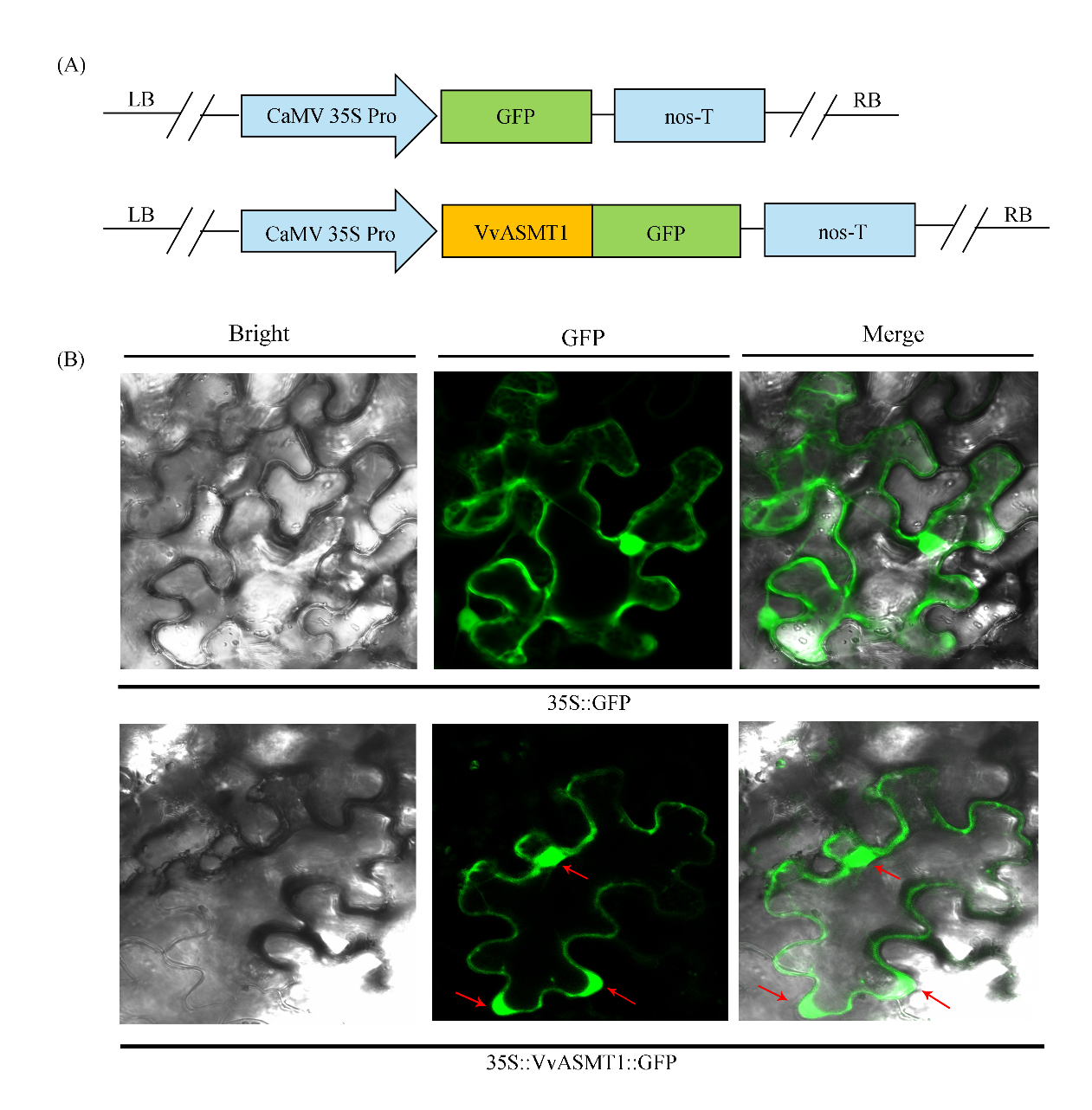


S4 Fig. Expression pattern of *VvASMT1* in different organs of grape. The experiments were repeated three times and vertical bars indicate the standard error of the mean. The letters above the columns represents significant differences (P < 0.05) based on Duncan’s multiple test.


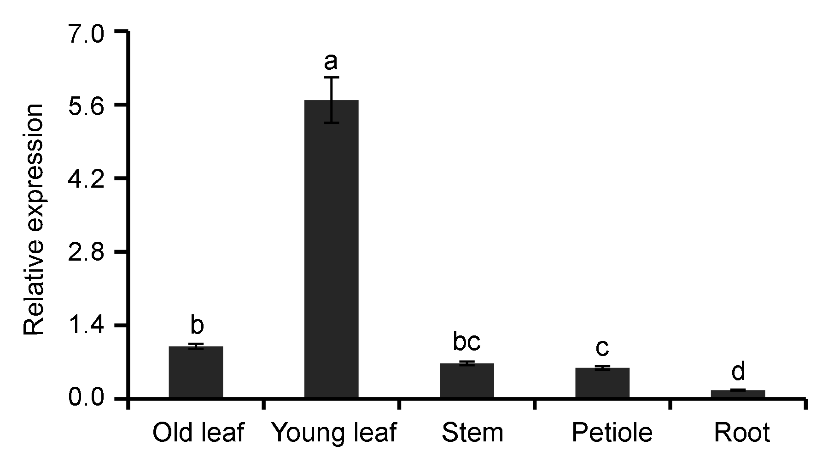


S5 Fig. Acquisition and identification of *VvASMT1* transgenic *Nicotiana benthamiana*. (A-D) Transgenic plants were obtained by disc conversion, (E) Identification of *Nicotiana benthamiana* 35S promoter by PCR, (F) The qRT-PCR of *VvASMT1* in T2 generation plants, (G) Western blot analysis of *VvASMT1* protein expression in T2 generation plants.


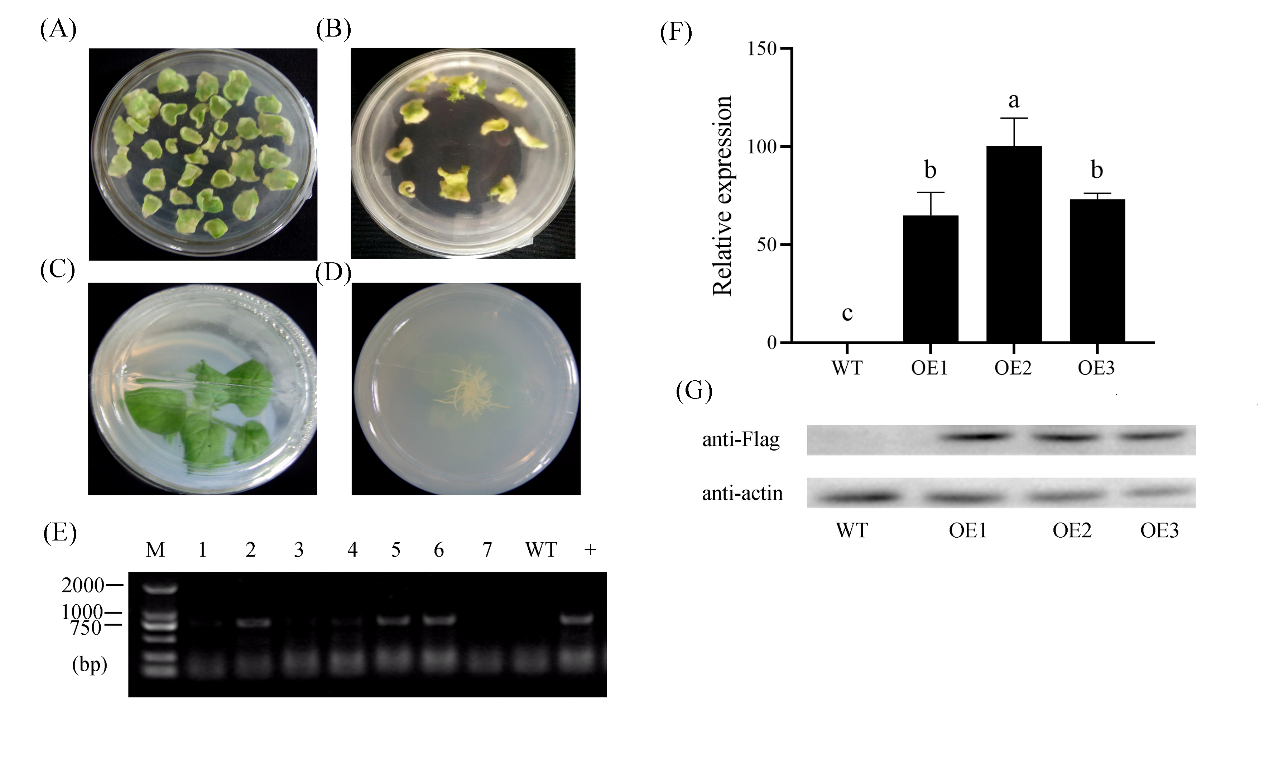

Supplement: S1 File — (DOCX) [file pone.0269028.s001.docx]
