# Supplementary material for: Overexpression of VvASMT1 from grapevine enhanced salt and osmotic stress tolerance in Nicotiana benthamiana
Source: PLoS One. 2022 Jun 16;17(6):e0269028. doi: 10.1371/journal.pone.0269028 (PMC9202941; doi:10.1371/journal.pone.0269028)
Supplement: S1 Data — (ZIP) [file pone.0269028.s002.zip › ╘¡╩╝╩2╛▌/╞⌠╢»╫╙╖╓╬÷.docx]

| *Cis*-acting elements | Numbers | Function of the *Cis*-acting element |
| --- | --- | --- |
| Abiotic stress responsive element |  |  |
| [MBS](http://bioinformatics.psb.ugent.be/webtools/plantcare/cgi-bin/show_site_info.htpl?QWhere=ID_of_Site like 'CAACTG'&StartAt=0&NbRecs=10) | 1 | MYB binding site involved in drought-inducibility |
| [LTR](http://bioinformatics.psb.ugent.be/webtools/plantcare/cgi-bin/show_site_info.htpl?QWhere=ID_of_Site like 'CCGAAA'&StartAt=0&NbRecs=10) | 1 | cis-acting element involved in low-temperature responsiveness |
| [WUN-motif](http://bioinformatics.psb.ugent.be/webtools/plantcare/cgi-bin/show_site_info.htpl?QWhere=ID_of_Site like 'AAATTTCTT'&StartAt=0&NbRecs=10) | 3 | Wound-responsive element |
| [GC-motif](http://bioinformatics.psb.ugent.be/webtools/plantcare/cgi-bin/show_site_info.htpl?QWhere=ID_of_Site like 'CCCCCG'&StartAt=0&NbRecs=10) | 1 | enhancer-like element involved in anoxic specific inducibility |
| HSE | 2 | cis-acting regulatory element involved in heat stress responsiveness |
| TC-rich repeats | 1 | cis-acting regulatory element involved in defense and stress responsiveness |
| Signaling molecules  responsive element |  |  |
| [TGA-element](http://bioinformatics.psb.ugent.be/webtools/plantcare/cgi-bin/show_site_info.htpl?QWhere=ID_of_Site like 'AACGAC'&StartAt=0&NbRecs=10) | 1 | auxin-responsive element |
| AuxRR-core | 2 | cis-acting regulatory element involved in auxin responsiveness |
| ABRE | 3 | cis-acting element involved in the abscisic acid responsiveness |
| [TGACG-motif](http://bioinformatics.psb.ugent.be/webtools/plantcare/cgi-bin/show_site_info.htpl?QWhere=ID_of_Site like 'TGACG'&StartAt=0&NbRecs=10) | 2 | cis-acting regulatory element involved in the MeJA-responsiveness |
| [TCA-element](http://bioinformatics.psb.ugent.be/webtools/plantcare/cgi-bin/show_site_info.htpl?QWhere=ID_of_Site like 'CCATCTTTTT'&StartAt=0&NbRecs=10) | 1 | cis-acting element involved in salicylic acid responsiveness |
| [TATC-box](http://bioinformatics.psb.ugent.be/webtools/plantcare/cgi-bin/show_site_info.htpl?QWhere=ID_of_Site like 'TATCCCA'&StartAt=0&NbRecs=10) | 1 | cis-acting element involved in gibberellin-responsiveness |
| Tissue-specific and development-related elements |  |  |
| [CAT-box](http://bioinformatics.psb.ugent.be/webtools/plantcare/cgi-bin/show_site_info.htpl?QWhere=ID_of_Site like 'GCCACT'&StartAt=0&NbRecs=10) | 1 | cis-acting regulatory element related to meristem expression |
| [GCN4_motif](http://bioinformatics.psb.ugent.be/webtools/plantcare/cgi-bin/show_site_info.htpl?QWhere=ID_of_Site like 'TGAGTCA'&StartAt=0&NbRecs=10) | 1 | cis-regulatory element involved in endosperm expression |

3000bp
