# Supplementary material for: Overexpression of VvASMT1 from grapevine enhanced salt and osmotic stress tolerance in Nicotiana benthamiana
Source: PLoS One. 2022 Jun 16;17(6):e0269028. doi: 10.1371/journal.pone.0269028 (PMC9202941; doi:10.1371/journal.pone.0269028)
Supplement: S1 Data — (ZIP) [file pone.0269028.s002.zip › ╘¡╩╝╩2╛▌/PlantCARE_32120__plantCARE.tar.gz!/PlantCARE_32120_plantcare.html]

Content-Type: text/html; charset=ISO-8859-1


PlantCARE


Webmaster Firefox specific output  
To save the result:
click on the frame with the right mouse button and save the source code as a text file with extension .html  
REFERENCE:PlantCARE: a database of plant cis-acting regulatory elements and a portal to tools for in silico analysis of promoter sequences.  
Lescot, M., Déhais, P., Moreau, Y., De Moor, B., Rouzé ,P.,and Rombauts, S.  
Nucleic Acids Res., Database issue(2002), 30(1):325-327.   


---

>PlantCARE\_32120   
+ AATCATTAAA GTCTAATTCT TCAAAATTCC GAATTCTAAA TTTAGCTATT TAAAATTCCA ATTGTCCTAT   
  
  
+ CTCCGAACTT AATTTTCAGT TTCCCATGCT CCAATTCCCG TATTTACCCC GCTACTTATT ATTATCATTA   
  
  
+ TCATTATCAC CTTATTCATT ATTTCTATAA ATTCCGCCTT CGAATGATTT CTAAGATCTC CAATTTTTAT   
  
  
+ ACATCAACTT TCATAATTTC TACGTCTCAA ATAACATACG ATTCTGATTC CTTTCAAAAT TTAACTCCCA   
  
  
+ TAATTCCAAT TTTCGTAACT TAATTTTTCT TAAAAATCCC GAACTTTCAA AATCCCAATT TTCTTTCAAA   
  
  
+ TTTAATTTCT AAATGTTCTC ATTCTTACAT CTAATTCCTA AGAATCCAAT TTTCAAATAA TCTCTAAGAT   
  
  
+ TCCGATTCTC AAATGAATTT CAAAAATCCA GTTTCCCTTC GAACAATTTT AATCCCTGTT TAGTGATGCA   
  
  
+ TGTAATATGT TTTGTGCTCT ATATGGTGTA CTAACCCTCT CTATTGATAG CGCATGGTGG TTCGTGGCCC   
  
  
+ AGGTATGTAC CCATTTTCTC TCTAATCGTT GTCTATGCAT GTCTCGATTC TTATGTGTGC ACGATCATTC   
  
  
+ AGGATATTCA TTGATTTACT CATCAATTGT CACGGCAGCT TCATTTTATT AGTAGAGACC CGACTTTAGG   
  
  
+ GACTTAGAGG GGTGCTACGG TCCTTACCGT ACCTTCCTGA TAAGTAACCT GACCCCCGAA CCCGATCCGG   
  
  
+ TTTTTCACAG ACCATCTTTT CCAAAATAAG GAGTCACACT TAGGGTTTTT CTTTCTTATT TTGTTTACCC   
  
  
+ TTTAAAAATA AAACAAAAAT AAGTGGCGAC TCCAAGTCAT TTTTCTTAAT CAATAAAGAT CATTTTTTCG   
  
  
+ AAAACGAAAA TCGAGCTCGC CATCGAGTGG GAAACGCATT GAGCCGAAAT GCGGGGTCCA TAGGTATGTT   
  
  
+ TTTTATTTAT GAGATAAAAA GTAAATAATA TTTTTTTCGG GTTTAAAAAA TATATTATTG ATTACTTGTA   
  
  
+ ATTATATGAG CCTCGTTTGT ACTTATTATT AAATACATAT TCAAGGTAAA ATTATCCTTT AAAAAATTCT   
  
  
+ TAGGCCGAGG GAACCTTCCT ATTAATATAA GTCATCCAGC CCACCTTATG GGGGTAATTC TCCCCATATA   
  
  
+ CCATGGGTCC AAATCGGCGG CCCGGTCAGT TCAACGACAG TACGATGGCA CTCTGAACCC TGTGCTGCAT   
  
  
+ CTGCTCACAA AAAGTACACC ACAGTTGTCT TTCACTCTCT CTGCCCATTC ACTATGCAAA CACGTTTATA   
  
  
+ AAATTGGTTA TTTTATAAAC GTTTCTCTTT CAAATGGTTA TTTTATATTA GTAACAAAAA TATTATAATA   
  
  
+ ATAAAAAAAT CAATATTACG TAATTGCTAA AAATGAAATT ACATCATGTT GTAAGATATT TCATTTGCCT   
  
  
+ ATTAATAATT TTTTATTATT TATAAAATTG GGTTGAATTT CAATATTTTT TAAAATAAGT CTTATTTATT   
  
  
+ TAAGTGAAAT TTTTTTTATA GATAAAGTCA CAAAAATCAT TTTTCTAAAT AATTTATAAA AAGATACTTT   
  
  
+ TCCAATAAAA ATTAAATTAA AATGCTTTTA TAAATAAAAT TGTAACAATT CATTCCATAG CAAAGGTAAA   
  
  
+ TAAAGCCATT TTCTTATCAA TTAAATCAAG CTTCTCTTTT TTATGAAATT GTTTTTAATA AATTCTTTTA   
  
  
+ GTAACAATAA GATTTTGTTC ATATTTTAAT ACATCTCCTT GGCAATTGGG CAAAGATGGC TGGTAGTGAC   
  
  
+ ATGAATCCAA GCATGAATTA GTCCGGGCGT TGTTACTTGC TACAAGGGTT ATGAGCATGG AAGGAAAAAT   
  
  
+ AATTAAAAAA TAAAAAAAAT TAAATTACTT CAATCACAAA GTTGAAAATA ATAAAATTGT GTTTGGAAGT   
  
  
+ ATTTGTACTT AAAGTTTTTT AGTAAAAGTG TTTCCCTAAA AGGTGTTTTT TTTAGAGAAA GCAGCTATAA   
  
  
+ AATGTTTTTT CAAAAATATT TTTTTATTTA AAAACACTTT CTTAAATCAC TTGTTATAAG TCACCTTAGA   
  
  
+ ATGACTGACG GTGTTGCACA TATCAAAAAG CATTTATGCC CTAGTGCCAT TTTATTATTT TATATCTTAA   
  
  
+ TTTTAGATAT TTTTATTTTT ATTTAATGAT TTATATTTGA ACGTTAATTT TCAATATATC ATATTGTATC   
  
  
+ TTTTATAGCC ACGTTTATTC AAATATATTT TTTATATTTA TAAATATTTT TTCTAAAATA TGATTCGAGA   
  
  
+ ATGCTGACAT GTGAATCGCA TGGGGGTAGG GCAAGCCATC TTGGACCCAT TAAACTTCAA ATTCCATGAT   
  
  
+ TGACTAGGAC TTGCCCAAAA ATTTCATTAC CCCAGATGCC CACATGTGAA TGCAGTCTCT GACAGAGTAG   
  
  
+ GTGGGAATAA ACCAAAATGC ATTTATTTAT TTTTCAAGAT TTTAATTATT ATTCACCTAA AATCCTTCAT   
  
  
+ AGGGATTAAA ATATTAGACC ATTGGATTTG CTCTCCTCAA TAGGGTTGAA CTTTGATTAG TACGTTTATA   
  
  
+ TTATTTTTTT CCTTTTTCGT CAAAATGCAT TCTGTCACAA AATTAATGTA GTCAAGGAAT TGACAAGTGA   
  
  
+ GAAGAGAGTC ACTTTAAATG ATTCAGCTGT CCACATTAAA GATAAGTCAC ATTTGTGGTC CATACTCCCC   
  
  
+ AAGACTTTAA TTTGAATTTA GCTCAGACTT TTCGTATTTT ATTTGTGTTT TTCTATTTAA GCTATCAATA   
  
  
+ CATTTATAAA TAAGTGATTT TAATATAAAA GATCAAGTAA AAAACAAATA AAGTACATAA AATATTTTTA   
  
  
+ TTCAATTACA TATTGTATCA AATTTTCAAG AGCAAAATTT ATAGCATTTT TCAATGTTTT GGAAATCAAA   
  
  
+ AAAGTAATAT ATTATCAGTT CACTAGTTAA ATTGGTGATT GAACCATAGA TGATGTCATA ATATATATTT   
  
  
+ TATATTTATT AAAATTAAAA ATATTTATAA GAAATTTTAA ATATATGAGA AATTAAAAGT CAATAATATT   
  
  
+ TTTTTCTTCA TCTTTAATAT TATCAAATGA ATATATATAT ATATATAAAT GTATTGATAT TTTAAAATTT   
  
  
+ TGTTAAATAA AATATATATA ATATGAAGAT ATTTATTGAA AGTTATTTAA TTTACCTATA TATATATTAA   
  
  
+ AAGTTATTTA TGGTTGTAAA AAATATTACA TTATTTTATT TATATTATTA ATATTACATT ATTTTATTTA   
  
  
+ TATTATTTAT AATTTCATTA TTATAAAAAG TTTGAAAATA TTATTGCTAT ATAATTTGTT GTTGTGTGAA   
  
  
+ GATCGGCAAA AAAACAGGAG TAGCAGTGTG GTGTCGTCCT TGCCCCTTGA ACTGGAGGTT GGTTTCCCAT   
  
  
+ TCCTCCCTCC CACGAATCCC ACATGTAAAG CAAATAAAAG AGCTTTACAT ACACATGATT AATGTATTTT   
  
  
+ TATTGTCAAT AAATTTTATA ATAAAACTTA TAAATCTTAT AAATTTAATA GGAAAAAAAA AAGTCTTCTA   
  
  
+ GAATTGATTT TATAATACAC CATACCAAGA TCACATGAAT TGCATGTGTC GGTGGACAAG GTTGCCAAAA   
  
  
+ TTCCTTATTC CTATACAGAA TCTTTTCATT GTTATATTAT CATAGTCGAA ACCCAGTAGT ATATAAAGAG   
  
  
+ GTTACGAGTG GAGGATGGAT ATGAAATAAA TAAGAGGCCT TAGAGATCAG GGTGTGAGAC ATGGATTTGG   
  
  
+ CAAATGGTGA GAGATCTAGT GAGC  

- TTAGTAATTT CAGATTAAGA AGTTTTAAGG CTTAAGATTT AAATCGATAA ATTTTAAGGT TAACAGGATA   
  
  
- GAGGCTTGAA TTAAAAGTCA AAGGGTACGA GGTTAAGGGC ATAAATGGGG CGATGAATAA TAATAGTAAT   
  
  
- AGTAATAGTG GAATAAGTAA TAAAGATATT TAAGGCGGAA GCTTACTAAA GATTCTAGAG GTTAAAAATA   
  
  
- TGTAGTTGAA AGTATTAAAG ATGCAGAGTT TATTGTATGC TAAGACTAAG GAAAGTTTTA AATTGAGGGT   
  
  
- ATTAAGGTTA AAAGCATTGA ATTAAAAAGA ATTTTTAGGG CTTGAAAGTT TTAGGGTTAA AAGAAAGTTT   
  
  
- AAATTAAAGA TTTACAAGAG TAAGAATGTA GATTAAGGAT TCTTAGGTTA AAAGTTTATT AGAGATTCTA   
  
  
- AGGCTAAGAG TTTACTTAAA GTTTTTAGGT CAAAGGGAAG CTTGTTAAAA TTAGGGACAA ATCACTACGT   
  
  
- ACATTATACA AAACACGAGA TATACCACAT GATTGGGAGA GATAACTATC GCGTACCACC AAGCACCGGG   
  
  
- TCCATACATG GGTAAAAGAG AGATTAGCAA CAGATACGTA CAGAGCTAAG AATACACACG TGCTAGTAAG   
  
  
- TCCTATAAGT AACTAAATGA GTAGTTAACA GTGCCGTCGA AGTAAAATAA TCATCTCTGG GCTGAAATCC   
  
  
- CTGAATCTCC CCACGATGCC AGGAATGGCA TGGAAGGACT ATTCATTGGA CTGGGGGCTT GGGCTAGGCC   
  
  
- AAAAAGTGTC TGGTAGAAAA GGTTTTATTC CTCAGTGTGA ATCCCAAAAA GAAAGAATAA AACAAATGGG   
  
  
- AAATTTTTAT TTTGTTTTTA TTCACCGCTG AGGTTCAGTA AAAAGAATTA GTTATTTCTA GTAAAAAAGC   
  
  
- TTTTGCTTTT AGCTCGAGCG GTAGCTCACC CTTTGCGTAA CTCGGCTTTA CGCCCCAGGT ATCCATACAA   
  
  
- AAAATAAATA CTCTATTTTT CATTTATTAT AAAAAAAGCC CAAATTTTTT ATATAATAAC TAATGAACAT   
  
  
- TAATATACTC GGAGCAAACA TGAATAATAA TTTATGTATA AGTTCCATTT TAATAGGAAA TTTTTTAAGA   
  
  
- ATCCGGCTCC CTTGGAAGGA TAATTATATT CAGTAGGTCG GGTGGAATAC CCCCATTAAG AGGGGTATAT   
  
  
- GGTACCCAGG TTTAGCCGCC GGGCCAGTCA AGTTGCTGTC ATGCTACCGT GAGACTTGGG ACACGACGTA   
  
  
- GACGAGTGTT TTTCATGTGG TGTCAACAGA AAGTGAGAGA GACGGGTAAG TGATACGTTT GTGCAAATAT   
  
  
- TTTAACCAAT AAAATATTTG CAAAGAGAAA GTTTACCAAT AAAATATAAT CATTGTTTTT ATAATATTAT   
  
  
- TATTTTTTTA GTTATAATGC ATTAACGATT TTTACTTTAA TGTAGTACAA CATTCTATAA AGTAAACGGA   
  
  
- TAATTATTAA AAAATAATAA ATATTTTAAC CCAACTTAAA GTTATAAAAA ATTTTATTCA GAATAAATAA   
  
  
- ATTCACTTTA AAAAAAATAT CTATTTCAGT GTTTTTAGTA AAAAGATTTA TTAAATATTT TTCTATGAAA   
  
  
- AGGTTATTTT TAATTTAATT TTACGAAAAT ATTTATTTTA ACATTGTTAA GTAAGGTATC GTTTCCATTT   
  
  
- ATTTCGGTAA AAGAATAGTT AATTTAGTTC GAAGAGAAAA AATACTTTAA CAAAAATTAT TTAAGAAAAT   
  
  
- CATTGTTATT CTAAAACAAG TATAAAATTA TGTAGAGGAA CCGTTAACCC GTTTCTACCG ACCATCACTG   
  
  
- TACTTAGGTT CGTACTTAAT CAGGCCCGCA ACAATGAACG ATGTTCCCAA TACTCGTACC TTCCTTTTTA   
  
  
- TTAATTTTTT ATTTTTTTTA ATTTAATGAA GTTAGTGTTT CAACTTTTAT TATTTTAACA CAAACCTTCA   
  
  
- TAAACATGAA TTTCAAAAAA TCATTTTCAC AAAGGGATTT TCCACAAAAA AAATCTCTTT CGTCGATATT   
  
  
- TTACAAAAAA GTTTTTATAA AAAAATAAAT TTTTGTGAAA GAATTTAGTG AACAATATTC AGTGGAATCT   
  
  
- TACTGACTGC CACAACGTGT ATAGTTTTTC GTAAATACGG GATCACGGTA AAATAATAAA ATATAGAATT   
  
  
- AAAATCTATA AAAATAAAAA TAAATTACTA AATATAAACT TGCAATTAAA AGTTATATAG TATAACATAG   
  
  
- AAAATATCGG TGCAAATAAG TTTATATAAA AAATATAAAT ATTTATAAAA AAGATTTTAT ACTAAGCTCT   
  
  
- TACGACTGTA CACTTAGCGT ACCCCCATCC CGTTCGGTAG AACCTGGGTA ATTTGAAGTT TAAGGTACTA   
  
  
- ACTGATCCTG AACGGGTTTT TAAAGTAATG GGGTCTACGG GTGTACACTT ACGTCAGAGA CTGTCTCATC   
  
  
- CACCCTTATT TGGTTTTACG TAAATAAATA AAAAGTTCTA AAATTAATAA TAAGTGGATT TTAGGAAGTA   
  
  
- TCCCTAATTT TATAATCTGG TAACCTAAAC GAGAGGAGTT ATCCCAACTT GAAACTAATC ATGCAAATAT   
  
  
- AATAAAAAAA GGAAAAAGCA GTTTTACGTA AGACAGTGTT TTAATTACAT CAGTTCCTTA ACTGTTCACT   
  
  
- CTTCTCTCAG TGAAATTTAC TAAGTCGACA GGTGTAATTT CTATTCAGTG TAAACACCAG GTATGAGGGG   
  
  
- TTCTGAAATT AAACTTAAAT CGAGTCTGAA AAGCATAAAA TAAACACAAA AAGATAAATT CGATAGTTAT   
  
  
- GTAAATATTT ATTCACTAAA ATTATATTTT CTAGTTCATT TTTTGTTTAT TTCATGTATT TTATAAAAAT   
  
  
- AAGTTAATGT ATAACATAGT TTAAAAGTTC TCGTTTTAAA TATCGTAAAA AGTTACAAAA CCTTTAGTTT   
  
  
- TTTCATTATA TAATAGTCAA GTGATCAATT TAACCACTAA CTTGGTATCT ACTACAGTAT TATATATAAA   
  
  
- ATATAAATAA TTTTAATTTT TATAAATATT CTTTAAAATT TATATACTCT TTAATTTTCA GTTATTATAA   
  
  
- AAAAAGAAGT AGAAATTATA ATAGTTTACT TATATATATA TATATATTTA CATAACTATA AAATTTTAAA   
  
  
- ACAATTTATT TTATATATAT TATACTTCTA TAAATAACTT TCAATAAATT AAATGGATAT ATATATAATT   
  
  
- TTCAATAAAT ACCAACATTT TTTATAATGT AATAAAATAA ATATAATAAT TATAATGTAA TAAAATAAAT   
  
  
- ATAATAAATA TTAAAGTAAT AATATTTTTC AAACTTTTAT AATAACGATA TATTAAACAA CAACACACTT   
  
  
- CTAGCCGTTT TTTTGTCCTC ATCGTCACAC CACAGCAGGA ACGGGGAACT TGACCTCCAA CCAAAGGGTA   
  
  
- AGGAGGGAGG GTGCTTAGGG TGTACATTTC GTTTATTTTC TCGAAATGTA TGTGTACTAA TTACATAAAA   
  
  
- ATAACAGTTA TTTAAAATAT TATTTTGAAT ATTTAGAATA TTTAAATTAT CCTTTTTTTT TTCAGAAGAT   
  
  
- CTTAACTAAA ATATTATGTG GTATGGTTCT AGTGTACTTA ACGTACACAG CCACCTGTTC CAACGGTTTT   
  
  
- AAGGAATAAG GATATGTCTT AGAAAAGTAA CAATATAATA GTATCAGCTT TGGGTCATCA TATATTTCTC   
  
  
- CAATGCTCAC CTCCTACCTA TACTTTATTT ATTCTCCGGA ATCTCTAGTC CCACACTCTG TACCTAAACC   
  
  
- GTTTACCACT CTCTAGATCA CTCG

  
  
Motifs Found  

+   

| Site Name | Organism | Position | Strand | Matrix score. | sequence | function |
| --- | --- | --- | --- | --- | --- | --- |
|  | organism | 1783 | + | 4 | motif\_sequence | short\_function |
|  | organism | 69 | + | 4 | motif\_sequence | short\_function |
|  | organism | 2687 | + | 4 | motif\_sequence | short\_function |
|  | organism | 682 | - | 4 | motif\_sequence | short\_function |
|  | organism | 363 | + | 4 | motif\_sequence | short\_function |
|  | organism | 2551 | + | 4 | motif\_sequence | short\_function |
|  | organism | 3175 | - | 4 | motif\_sequence | short\_function |
|  | organism | 1259 | + | 4 | motif\_sequence | short\_function |
|  | organism | 196 | + | 4 | motif\_sequence | short\_function |
|  | organism | 2853 | - | 4 | motif\_sequence | short\_function |
|  | organism | 1250 | + | 4 | motif\_sequence | short\_function |
|  | organism | 3358 | - | 4 | motif\_sequence | short\_function |
|  | organism | 565 | + | 4 | motif\_sequence | short\_function |
|  | organism | 2116 | - | 4 | motif\_sequence | short\_function |
|  | organism | 2661 | - | 4 | motif\_sequence | short\_function |
|  | organism | 1299 | + | 4 | motif\_sequence | short\_function |

>PlantCARE\_32120   
+ AATCATTAAA GTCTAATTCT TCAAAATTCC GAATTCTAAA TTTAGCTATT TAAAATTCCA ATTGTCCTAT   
  
  
+ CTCCGAACTT AATTTTCAGT TTCCCATGCT CCAATTCCCG TATTTACCCC GCTACTTATT ATTATCATTA   
  
  
+ TCATTATCAC CTTATTCATT ATTTCTATAA ATTCCGCCTT CGAATGATTT CTAAGATCTC CAATTTTTAT   
  
  
+ ACATCAACTT TCATAATTTC TACGTCTCAA ATAACATACG ATTCTGATTC CTTTCAAAAT TTAACTCCCA   
  
  
+ TAATTCCAAT TTTCGTAACT TAATTTTTCT TAAAAATCCC GAACTTTCAA AATCCCAATT TTCTTTCAAA   
  
  
+ TTTAATTTCT AAATGTTCTC ATTCTTACAT CTAATTCCTA AGAATCCAAT TTTCAAATAA TCTCTAAGAT   
  
  
+ TCCGATTCTC AAATGAATTT CAAAAATCCA GTTTCCCTTC GAACAATTTT AATCCCTGTT TAGTGATGCA   
  
  
+ TGTAATATGT TTTGTGCTCT ATATGGTGTA CTAACCCTCT CTATTGATAG CGCATGGTGG TTCGTGGCCC   
  
  
+ AGGTATGTAC CCATTTTCTC TCTAATCGTT GTCTATGCAT GTCTCGATTC TTATGTGTGC ACGATCATTC   
  
  
+ AGGATATTCA TTGATTTACT CATCAATTGT CACGGCAGCT TCATTTTATT AGTAGAGACC CGACTTTAGG   
  
  
+ GACTTAGAGG GGTGCTACGG TCCTTACCGT ACCTTCCTGA TAAGTAACCT GACCCCCGAA CCCGATCCGG   
  
  
+ TTTTTCACAG ACCATCTTTT CCAAAATAAG GAGTCACACT TAGGGTTTTT CTTTCTTATT TTGTTTACCC   
  
  
+ TTTAAAAATA AAACAAAAAT AAGTGGCGAC TCCAAGTCAT TTTTCTTAAT CAATAAAGAT CATTTTTTCG   
  
  
+ AAAACGAAAA TCGAGCTCGC CATCGAGTGG GAAACGCATT GAGCCGAAAT GCGGGGTCCA TAGGTATGTT   
  
  
+ TTTTATTTAT GAGATAAAAA GTAAATAATA TTTTTTTCGG GTTTAAAAAA TATATTATTG ATTACTTGTA   
  
  
+ ATTATATGAG CCTCGTTTGT ACTTATTATT AAATACATAT TCAAGGTAAA ATTATCCTTT AAAAAATTCT   
  
  
+ TAGGCCGAGG GAACCTTCCT ATTAATATAA GTCATCCAGC CCACCTTATG GGGGTAATTC TCCCCATATA   
  
  
+ CCATGGGTCC AAATCGGCGG CCCGGTCAGT TCAACGACAG TACGATGGCA CTCTGAACCC TGTGCTGCAT   
  
  
+ CTGCTCACAA AAAGTACACC ACAGTTGTCT TTCACTCTCT CTGCCCATTC ACTATGCAAA CACGTTTATA   
  
  
+ AAATTGGTTA TTTTATAAAC GTTTCTCTTT CAAATGGTTA TTTTATATTA GTAACAAAAA TATTATAATA   
  
  
+ ATAAAAAAAT CAATATTACG TAATTGCTAA AAATGAAATT ACATCATGTT GTAAGATATT TCATTTGCCT   
  
  
+ ATTAATAATT TTTTATTATT TATAAAATTG GGTTGAATTT CAATATTTTT TAAAATAAGT CTTATTTATT   
  
  
+ TAAGTGAAAT TTTTTTTATA GATAAAGTCA CAAAAATCAT TTTTCTAAAT AATTTATAAA AAGATACTTT   
  
  
+ TCCAATAAAA ATTAAATTAA AATGCTTTTA TAAATAAAAT TGTAACAATT CATTCCATAG CAAAGGTAAA   
  
  
+ TAAAGCCATT TTCTTATCAA TTAAATCAAG CTTCTCTTTT TTATGAAATT GTTTTTAATA AATTCTTTTA   
  
  
+ GTAACAATAA GATTTTGTTC ATATTTTAAT ACATCTCCTT GGCAATTGGG CAAAGATGGC TGGTAGTGAC   
  
  
+ ATGAATCCAA GCATGAATTA GTCCGGGCGT TGTTACTTGC TACAAGGGTT ATGAGCATGG AAGGAAAAAT   
  
  
+ AATTAAAAAA TAAAAAAAAT TAAATTACTT CAATCACAAA GTTGAAAATA ATAAAATTGT GTTTGGAAGT   
  
  
+ ATTTGTACTT AAAGTTTTTT AGTAAAAGTG TTTCCCTAAA AGGTGTTTTT TTTAGAGAAA GCAGCTATAA   
  
  
+ AATGTTTTTT CAAAAATATT TTTTTATTTA AAAACACTTT CTTAAATCAC TTGTTATAAG TCACCTTAGA   
  
  
+ ATGACTGACG GTGTTGCACA TATCAAAAAG CATTTATGCC CTAGTGCCAT TTTATTATTT TATATCTTAA   
  
  
+ TTTTAGATAT TTTTATTTTT ATTTAATGAT TTATATTTGA ACGTTAATTT TCAATATATC ATATTGTATC   
  
  
+ TTTTATAGCC ACGTTTATTC AAATATATTT TTTATATTTA TAAATATTTT TTCTAAAATA TGATTCGAGA   
  
  
+ ATGCTGACAT GTGAATCGCA TGGGGGTAGG GCAAGCCATC TTGGACCCAT TAAACTTCAA ATTCCATGAT   
  
  
+ TGACTAGGAC TTGCCCAAAA ATTTCATTAC CCCAGATGCC CACATGTGAA TGCAGTCTCT GACAGAGTAG   
  
  
+ GTGGGAATAA ACCAAAATGC ATTTATTTAT TTTTCAAGAT TTTAATTATT ATTCACCTAA AATCCTTCAT   
  
  
+ AGGGATTAAA ATATTAGACC ATTGGATTTG CTCTCCTCAA TAGGGTTGAA CTTTGATTAG TACGTTTATA   
  
  
+ TTATTTTTTT CCTTTTTCGT CAAAATGCAT TCTGTCACAA AATTAATGTA GTCAAGGAAT TGACAAGTGA   
  
  
+ GAAGAGAGTC ACTTTAAATG ATTCAGCTGT CCACATTAAA GATAAGTCAC ATTTGTGGTC CATACTCCCC   
  
  
+ AAGACTTTAA TTTGAATTTA GCTCAGACTT TTCGTATTTT ATTTGTGTTT TTCTATTTAA GCTATCAATA   
  
  
+ CATTTATAAA TAAGTGATTT TAATATAAAA GATCAAGTAA AAAACAAATA AAGTACATAA AATATTTTTA   
  
  
+ TTCAATTACA TATTGTATCA AATTTTCAAG AGCAAAATTT ATAGCATTTT TCAATGTTTT GGAAATCAAA   
  
  
+ AAAGTAATAT ATTATCAGTT CACTAGTTAA ATTGGTGATT GAACCATAGA TGATGTCATA ATATATATTT   
  
  
+ TATATTTATT AAAATTAAAA ATATTTATAA GAAATTTTAA ATATATGAGA AATTAAAAGT CAATAATATT   
  
  
+ TTTTTCTTCA TCTTTAATAT TATCAAATGA ATATATATAT ATATATAAAT GTATTGATAT TTTAAAATTT   
  
  
+ TGTTAAATAA AATATATATA ATATGAAGAT ATTTATTGAA AGTTATTTAA TTTACCTATA TATATATTAA   
  
  
+ AAGTTATTTA TGGTTGTAAA AAATATTACA TTATTTTATT TATATTATTA ATATTACATT ATTTTATTTA   
  
  
+ TATTATTTAT AATTTCATTA TTATAAAAAG TTTGAAAATA TTATTGCTAT ATAATTTGTT GTTGTGTGAA   
  
  
+ GATCGGCAAA AAAACAGGAG TAGCAGTGTG GTGTCGTCCT TGCCCCTTGA ACTGGAGGTT GGTTTCCCAT   
  
  
+ TCCTCCCTCC CACGAATCCC ACATGTAAAG CAAATAAAAG AGCTTTACAT ACACATGATT AATGTATTTT   
  
  
+ TATTGTCAAT AAATTTTATA ATAAAACTTA TAAATCTTAT AAATTTAATA GGAAAAAAAA AAGTCTTCTA   
  
  
+ GAATTGATTT TATAATACAC CATACCAAGA TCACATGAAT TGCATGTGTC GGTGGACAAG GTTGCCAAAA   
  
  
+ TTCCTTATTC CTATACAGAA TCTTTTCATT GTTATATTAT CATAGTCGAA ACCCAGTAGT ATATAAAGAG   
  
  
+ GTTACGAGTG GAGGATGGAT ATGAAATAAA TAAGAGGCCT TAGAGATCAG GGTGTGAGAC ATGGATTTGG   
  
  
+ CAAATGGTGA GAGATCTAGT GAGC  

- TTAGTAATTT CAGATTAAGA AGTTTTAAGG CTTAAGATTT AAATCGATAA ATTTTAAGGT TAACAGGATA   
  
  
- GAGGCTTGAA TTAAAAGTCA AAGGGTACGA GGTTAAGGGC ATAAATGGGG CGATGAATAA TAATAGTAAT   
  
  
- AGTAATAGTG GAATAAGTAA TAAAGATATT TAAGGCGGAA GCTTACTAAA GATTCTAGAG GTTAAAAATA   
  
  
- TGTAGTTGAA AGTATTAAAG ATGCAGAGTT TATTGTATGC TAAGACTAAG GAAAGTTTTA AATTGAGGGT   
  
  
- ATTAAGGTTA AAAGCATTGA ATTAAAAAGA ATTTTTAGGG CTTGAAAGTT TTAGGGTTAA AAGAAAGTTT   
  
  
- AAATTAAAGA TTTACAAGAG TAAGAATGTA GATTAAGGAT TCTTAGGTTA AAAGTTTATT AGAGATTCTA   
  
  
- AGGCTAAGAG TTTACTTAAA GTTTTTAGGT CAAAGGGAAG CTTGTTAAAA TTAGGGACAA ATCACTACGT   
  
  
- ACATTATACA AAACACGAGA TATACCACAT GATTGGGAGA GATAACTATC GCGTACCACC AAGCACCGGG   
  
  
- TCCATACATG GGTAAAAGAG AGATTAGCAA CAGATACGTA CAGAGCTAAG AATACACACG TGCTAGTAAG   
  
  
- TCCTATAAGT AACTAAATGA GTAGTTAACA GTGCCGTCGA AGTAAAATAA TCATCTCTGG GCTGAAATCC   
  
  
- CTGAATCTCC CCACGATGCC AGGAATGGCA TGGAAGGACT ATTCATTGGA CTGGGGGCTT GGGCTAGGCC   
  
  
- AAAAAGTGTC TGGTAGAAAA GGTTTTATTC CTCAGTGTGA ATCCCAAAAA GAAAGAATAA AACAAATGGG   
  
  
- AAATTTTTAT TTTGTTTTTA TTCACCGCTG AGGTTCAGTA AAAAGAATTA GTTATTTCTA GTAAAAAAGC   
  
  
- TTTTGCTTTT AGCTCGAGCG GTAGCTCACC CTTTGCGTAA CTCGGCTTTA CGCCCCAGGT ATCCATACAA   
  
  
- AAAATAAATA CTCTATTTTT CATTTATTAT AAAAAAAGCC CAAATTTTTT ATATAATAAC TAATGAACAT   
  
  
- TAATATACTC GGAGCAAACA TGAATAATAA TTTATGTATA AGTTCCATTT TAATAGGAAA TTTTTTAAGA   
  
  
- ATCCGGCTCC CTTGGAAGGA TAATTATATT CAGTAGGTCG GGTGGAATAC CCCCATTAAG AGGGGTATAT   
  
  
- GGTACCCAGG TTTAGCCGCC GGGCCAGTCA AGTTGCTGTC ATGCTACCGT GAGACTTGGG ACACGACGTA   
  
  
- GACGAGTGTT TTTCATGTGG TGTCAACAGA AAGTGAGAGA GACGGGTAAG TGATACGTTT GTGCAAATAT   
  
  
- TTTAACCAAT AAAATATTTG CAAAGAGAAA GTTTACCAAT AAAATATAAT CATTGTTTTT ATAATATTAT   
  
  
- TATTTTTTTA GTTATAATGC ATTAACGATT TTTACTTTAA TGTAGTACAA CATTCTATAA AGTAAACGGA   
  
  
- TAATTATTAA AAAATAATAA ATATTTTAAC CCAACTTAAA GTTATAAAAA ATTTTATTCA GAATAAATAA   
  
  
- ATTCACTTTA AAAAAAATAT CTATTTCAGT GTTTTTAGTA AAAAGATTTA TTAAATATTT TTCTATGAAA   
  
  
- AGGTTATTTT TAATTTAATT TTACGAAAAT ATTTATTTTA ACATTGTTAA GTAAGGTATC GTTTCCATTT   
  
  
- ATTTCGGTAA AAGAATAGTT AATTTAGTTC GAAGAGAAAA AATACTTTAA CAAAAATTAT TTAAGAAAAT   
  
  
- CATTGTTATT CTAAAACAAG TATAAAATTA TGTAGAGGAA CCGTTAACCC GTTTCTACCG ACCATCACTG   
  
  
- TACTTAGGTT CGTACTTAAT CAGGCCCGCA ACAATGAACG ATGTTCCCAA TACTCGTACC TTCCTTTTTA   
  
  
- TTAATTTTTT ATTTTTTTTA ATTTAATGAA GTTAGTGTTT CAACTTTTAT TATTTTAACA CAAACCTTCA   
  
  
- TAAACATGAA TTTCAAAAAA TCATTTTCAC AAAGGGATTT TCCACAAAAA AAATCTCTTT CGTCGATATT   
  
  
- TTACAAAAAA GTTTTTATAA AAAAATAAAT TTTTGTGAAA GAATTTAGTG AACAATATTC AGTGGAATCT   
  
  
- TACTGACTGC CACAACGTGT ATAGTTTTTC GTAAATACGG GATCACGGTA AAATAATAAA ATATAGAATT   
  
  
- AAAATCTATA AAAATAAAAA TAAATTACTA AATATAAACT TGCAATTAAA AGTTATATAG TATAACATAG   
  
  
- AAAATATCGG TGCAAATAAG TTTATATAAA AAATATAAAT ATTTATAAAA AAGATTTTAT ACTAAGCTCT   
  
  
- TACGACTGTA CACTTAGCGT ACCCCCATCC CGTTCGGTAG AACCTGGGTA ATTTGAAGTT TAAGGTACTA   
  
  
- ACTGATCCTG AACGGGTTTT TAAAGTAATG GGGTCTACGG GTGTACACTT ACGTCAGAGA CTGTCTCATC   
  
  
- CACCCTTATT TGGTTTTACG TAAATAAATA AAAAGTTCTA AAATTAATAA TAAGTGGATT TTAGGAAGTA   
  
  
- TCCCTAATTT TATAATCTGG TAACCTAAAC GAGAGGAGTT ATCCCAACTT GAAACTAATC ATGCAAATAT   
  
  
- AATAAAAAAA GGAAAAAGCA GTTTTACGTA AGACAGTGTT TTAATTACAT CAGTTCCTTA ACTGTTCACT   
  
  
- CTTCTCTCAG TGAAATTTAC TAAGTCGACA GGTGTAATTT CTATTCAGTG TAAACACCAG GTATGAGGGG   
  
  
- TTCTGAAATT AAACTTAAAT CGAGTCTGAA AAGCATAAAA TAAACACAAA AAGATAAATT CGATAGTTAT   
  
  
- GTAAATATTT ATTCACTAAA ATTATATTTT CTAGTTCATT TTTTGTTTAT TTCATGTATT TTATAAAAAT   
  
  
- AAGTTAATGT ATAACATAGT TTAAAAGTTC TCGTTTTAAA TATCGTAAAA AGTTACAAAA CCTTTAGTTT   
  
  
- TTTCATTATA TAATAGTCAA GTGATCAATT TAACCACTAA CTTGGTATCT ACTACAGTAT TATATATAAA   
  
  
- ATATAAATAA TTTTAATTTT TATAAATATT CTTTAAAATT TATATACTCT TTAATTTTCA GTTATTATAA   
  
  
- AAAAAGAAGT AGAAATTATA ATAGTTTACT TATATATATA TATATATTTA CATAACTATA AAATTTTAAA   
  
  
- ACAATTTATT TTATATATAT TATACTTCTA TAAATAACTT TCAATAAATT AAATGGATAT ATATATAATT   
  
  
- TTCAATAAAT ACCAACATTT TTTATAATGT AATAAAATAA ATATAATAAT TATAATGTAA TAAAATAAAT   
  
  
- ATAATAAATA TTAAAGTAAT AATATTTTTC AAACTTTTAT AATAACGATA TATTAAACAA CAACACACTT   
  
  
- CTAGCCGTTT TTTTGTCCTC ATCGTCACAC CACAGCAGGA ACGGGGAACT TGACCTCCAA CCAAAGGGTA   
  
  
- AGGAGGGAGG GTGCTTAGGG TGTACATTTC GTTTATTTTC TCGAAATGTA TGTGTACTAA TTACATAAAA   
  
  
- ATAACAGTTA TTTAAAATAT TATTTTGAAT ATTTAGAATA TTTAAATTAT CCTTTTTTTT TTCAGAAGAT   
  
  
- CTTAACTAAA ATATTATGTG GTATGGTTCT AGTGTACTTA ACGTACACAG CCACCTGTTC CAACGGTTTT   
  
  
- AAGGAATAAG GATATGTCTT AGAAAAGTAA CAATATAATA GTATCAGCTT TGGGTCATCA TATATTTCTC   
  
  
- CAATGCTCAC CTCCTACCTA TACTTTATTT ATTCTCCGGA ATCTCTAGTC CCACACTCTG TACCTAAACC   
  
  
- GTTTACCACT CTCTAGATCA CTCG

+     AAGAA-motif

| Site Name | Organism | Position | Strand | Matrix score. | sequence | function |
| --- | --- | --- | --- | --- | --- | --- |
| AAGAA-motif | Avena sativa | 1675 | + | 9 | gGTAAAGAAA |  |
| AAGAA-motif | Avena sativa | 2402 | - | 9 | gGTAAAGAAA |  |
| AAGAA-motif | Avena sativa | 830 | - | 9 | gGTAAAGAAA |  |
| AAGAA-motif | Avena sativa | 819 | - | 7 | GAAAGAA |  |
| AAGAA-motif | Avena sativa | 341 | - | 7 | GAAAGAA |  |

>PlantCARE\_32120   
+ AATCATTAAA GTCTAATTCT TCAAAATTCC GAATTCTAAA TTTAGCTATT TAAAATTCCA ATTGTCCTAT   
  
  
+ CTCCGAACTT AATTTTCAGT TTCCCATGCT CCAATTCCCG TATTTACCCC GCTACTTATT ATTATCATTA   
  
  
+ TCATTATCAC CTTATTCATT ATTTCTATAA ATTCCGCCTT CGAATGATTT CTAAGATCTC CAATTTTTAT   
  
  
+ ACATCAACTT TCATAATTTC TACGTCTCAA ATAACATACG ATTCTGATTC CTTTCAAAAT TTAACTCCCA   
  
  
+ TAATTCCAAT TTTCGTAACT TAATTTTTCT TAAAAATCCC GAACTTTCAA AATCCCAATT TTCTTTCAAA   
  
  
+ TTTAATTTCT AAATGTTCTC ATTCTTACAT CTAATTCCTA AGAATCCAAT TTTCAAATAA TCTCTAAGAT   
  
  
+ TCCGATTCTC AAATGAATTT CAAAAATCCA GTTTCCCTTC GAACAATTTT AATCCCTGTT TAGTGATGCA   
  
  
+ TGTAATATGT TTTGTGCTCT ATATGGTGTA CTAACCCTCT CTATTGATAG CGCATGGTGG TTCGTGGCCC   
  
  
+ AGGTATGTAC CCATTTTCTC TCTAATCGTT GTCTATGCAT GTCTCGATTC TTATGTGTGC ACGATCATTC   
  
  
+ AGGATATTCA TTGATTTACT CATCAATTGT CACGGCAGCT TCATTTTATT AGTAGAGACC CGACTTTAGG   
  
  
+ GACTTAGAGG GGTGCTACGG TCCTTACCGT ACCTTCCTGA TAAGTAACCT GACCCCCGAA CCCGATCCGG   
  
  
+ TTTTTCACAG ACCATCTTTT CCAAAATAAG GAGTCACACT TAGGGTTTTT CTTTCTTATT TTGTTTACCC   
  
  
+ TTTAAAAATA AAACAAAAAT AAGTGGCGAC TCCAAGTCAT TTTTCTTAAT CAATAAAGAT CATTTTTTCG   
  
  
+ AAAACGAAAA TCGAGCTCGC CATCGAGTGG GAAACGCATT GAGCCGAAAT GCGGGGTCCA TAGGTATGTT   
  
  
+ TTTTATTTAT GAGATAAAAA GTAAATAATA TTTTTTTCGG GTTTAAAAAA TATATTATTG ATTACTTGTA   
  
  
+ ATTATATGAG CCTCGTTTGT ACTTATTATT AAATACATAT TCAAGGTAAA ATTATCCTTT AAAAAATTCT   
  
  
+ TAGGCCGAGG GAACCTTCCT ATTAATATAA GTCATCCAGC CCACCTTATG GGGGTAATTC TCCCCATATA   
  
  
+ CCATGGGTCC AAATCGGCGG CCCGGTCAGT TCAACGACAG TACGATGGCA CTCTGAACCC TGTGCTGCAT   
  
  
+ CTGCTCACAA AAAGTACACC ACAGTTGTCT TTCACTCTCT CTGCCCATTC ACTATGCAAA CACGTTTATA   
  
  
+ AAATTGGTTA TTTTATAAAC GTTTCTCTTT CAAATGGTTA TTTTATATTA GTAACAAAAA TATTATAATA   
  
  
+ ATAAAAAAAT CAATATTACG TAATTGCTAA AAATGAAATT ACATCATGTT GTAAGATATT TCATTTGCCT   
  
  
+ ATTAATAATT TTTTATTATT TATAAAATTG GGTTGAATTT CAATATTTTT TAAAATAAGT CTTATTTATT   
  
  
+ TAAGTGAAAT TTTTTTTATA GATAAAGTCA CAAAAATCAT TTTTCTAAAT AATTTATAAA AAGATACTTT   
  
  
+ TCCAATAAAA ATTAAATTAA AATGCTTTTA TAAATAAAAT TGTAACAATT CATTCCATAG CAAAGGTAAA   
  
  
+ TAAAGCCATT TTCTTATCAA TTAAATCAAG CTTCTCTTTT TTATGAAATT GTTTTTAATA AATTCTTTTA   
  
  
+ GTAACAATAA GATTTTGTTC ATATTTTAAT ACATCTCCTT GGCAATTGGG CAAAGATGGC TGGTAGTGAC   
  
  
+ ATGAATCCAA GCATGAATTA GTCCGGGCGT TGTTACTTGC TACAAGGGTT ATGAGCATGG AAGGAAAAAT   
  
  
+ AATTAAAAAA TAAAAAAAAT TAAATTACTT CAATCACAAA GTTGAAAATA ATAAAATTGT GTTTGGAAGT   
  
  
+ ATTTGTACTT AAAGTTTTTT AGTAAAAGTG TTTCCCTAAA AGGTGTTTTT TTTAGAGAAA GCAGCTATAA   
  
  
+ AATGTTTTTT CAAAAATATT TTTTTATTTA AAAACACTTT CTTAAATCAC TTGTTATAAG TCACCTTAGA   
  
  
+ ATGACTGACG GTGTTGCACA TATCAAAAAG CATTTATGCC CTAGTGCCAT TTTATTATTT TATATCTTAA   
  
  
+ TTTTAGATAT TTTTATTTTT ATTTAATGAT TTATATTTGA ACGTTAATTT TCAATATATC ATATTGTATC   
  
  
+ TTTTATAGCC ACGTTTATTC AAATATATTT TTTATATTTA TAAATATTTT TTCTAAAATA TGATTCGAGA   
  
  
+ ATGCTGACAT GTGAATCGCA TGGGGGTAGG GCAAGCCATC TTGGACCCAT TAAACTTCAA ATTCCATGAT   
  
  
+ TGACTAGGAC TTGCCCAAAA ATTTCATTAC CCCAGATGCC CACATGTGAA TGCAGTCTCT GACAGAGTAG   
  
  
+ GTGGGAATAA ACCAAAATGC ATTTATTTAT TTTTCAAGAT TTTAATTATT ATTCACCTAA AATCCTTCAT   
  
  
+ AGGGATTAAA ATATTAGACC ATTGGATTTG CTCTCCTCAA TAGGGTTGAA CTTTGATTAG TACGTTTATA   
  
  
+ TTATTTTTTT CCTTTTTCGT CAAAATGCAT TCTGTCACAA AATTAATGTA GTCAAGGAAT TGACAAGTGA   
  
  
+ GAAGAGAGTC ACTTTAAATG ATTCAGCTGT CCACATTAAA GATAAGTCAC ATTTGTGGTC CATACTCCCC   
  
  
+ AAGACTTTAA TTTGAATTTA GCTCAGACTT TTCGTATTTT ATTTGTGTTT TTCTATTTAA GCTATCAATA   
  
  
+ CATTTATAAA TAAGTGATTT TAATATAAAA GATCAAGTAA AAAACAAATA AAGTACATAA AATATTTTTA   
  
  
+ TTCAATTACA TATTGTATCA AATTTTCAAG AGCAAAATTT ATAGCATTTT TCAATGTTTT GGAAATCAAA   
  
  
+ AAAGTAATAT ATTATCAGTT CACTAGTTAA ATTGGTGATT GAACCATAGA TGATGTCATA ATATATATTT   
  
  
+ TATATTTATT AAAATTAAAA ATATTTATAA GAAATTTTAA ATATATGAGA AATTAAAAGT CAATAATATT   
  
  
+ TTTTTCTTCA TCTTTAATAT TATCAAATGA ATATATATAT ATATATAAAT GTATTGATAT TTTAAAATTT   
  
  
+ TGTTAAATAA AATATATATA ATATGAAGAT ATTTATTGAA AGTTATTTAA TTTACCTATA TATATATTAA   
  
  
+ AAGTTATTTA TGGTTGTAAA AAATATTACA TTATTTTATT TATATTATTA ATATTACATT ATTTTATTTA   
  
  
+ TATTATTTAT AATTTCATTA TTATAAAAAG TTTGAAAATA TTATTGCTAT ATAATTTGTT GTTGTGTGAA   
  
  
+ GATCGGCAAA AAAACAGGAG TAGCAGTGTG GTGTCGTCCT TGCCCCTTGA ACTGGAGGTT GGTTTCCCAT   
  
  
+ TCCTCCCTCC CACGAATCCC ACATGTAAAG CAAATAAAAG AGCTTTACAT ACACATGATT AATGTATTTT   
  
  
+ TATTGTCAAT AAATTTTATA ATAAAACTTA TAAATCTTAT AAATTTAATA GGAAAAAAAA AAGTCTTCTA   
  
  
+ GAATTGATTT TATAATACAC CATACCAAGA TCACATGAAT TGCATGTGTC GGTGGACAAG GTTGCCAAAA   
  
  
+ TTCCTTATTC CTATACAGAA TCTTTTCATT GTTATATTAT CATAGTCGAA ACCCAGTAGT ATATAAAGAG   
  
  
+ GTTACGAGTG GAGGATGGAT ATGAAATAAA TAAGAGGCCT TAGAGATCAG GGTGTGAGAC ATGGATTTGG   
  
  
+ CAAATGGTGA GAGATCTAGT GAGC  

- TTAGTAATTT CAGATTAAGA AGTTTTAAGG CTTAAGATTT AAATCGATAA ATTTTAAGGT TAACAGGATA   
  
  
- GAGGCTTGAA TTAAAAGTCA AAGGGTACGA GGTTAAGGGC ATAAATGGGG CGATGAATAA TAATAGTAAT   
  
  
- AGTAATAGTG GAATAAGTAA TAAAGATATT TAAGGCGGAA GCTTACTAAA GATTCTAGAG GTTAAAAATA   
  
  
- TGTAGTTGAA AGTATTAAAG ATGCAGAGTT TATTGTATGC TAAGACTAAG GAAAGTTTTA AATTGAGGGT   
  
  
- ATTAAGGTTA AAAGCATTGA ATTAAAAAGA ATTTTTAGGG CTTGAAAGTT TTAGGGTTAA AAGAAAGTTT   
  
  
- AAATTAAAGA TTTACAAGAG TAAGAATGTA GATTAAGGAT TCTTAGGTTA AAAGTTTATT AGAGATTCTA   
  
  
- AGGCTAAGAG TTTACTTAAA GTTTTTAGGT CAAAGGGAAG CTTGTTAAAA TTAGGGACAA ATCACTACGT   
  
  
- ACATTATACA AAACACGAGA TATACCACAT GATTGGGAGA GATAACTATC GCGTACCACC AAGCACCGGG   
  
  
- TCCATACATG GGTAAAAGAG AGATTAGCAA CAGATACGTA CAGAGCTAAG AATACACACG TGCTAGTAAG   
  
  
- TCCTATAAGT AACTAAATGA GTAGTTAACA GTGCCGTCGA AGTAAAATAA TCATCTCTGG GCTGAAATCC   
  
  
- CTGAATCTCC CCACGATGCC AGGAATGGCA TGGAAGGACT ATTCATTGGA CTGGGGGCTT GGGCTAGGCC   
  
  
- AAAAAGTGTC TGGTAGAAAA GGTTTTATTC CTCAGTGTGA ATCCCAAAAA GAAAGAATAA AACAAATGGG   
  
  
- AAATTTTTAT TTTGTTTTTA TTCACCGCTG AGGTTCAGTA AAAAGAATTA GTTATTTCTA GTAAAAAAGC   
  
  
- TTTTGCTTTT AGCTCGAGCG GTAGCTCACC CTTTGCGTAA CTCGGCTTTA CGCCCCAGGT ATCCATACAA   
  
  
- AAAATAAATA CTCTATTTTT CATTTATTAT AAAAAAAGCC CAAATTTTTT ATATAATAAC TAATGAACAT   
  
  
- TAATATACTC GGAGCAAACA TGAATAATAA TTTATGTATA AGTTCCATTT TAATAGGAAA TTTTTTAAGA   
  
  
- ATCCGGCTCC CTTGGAAGGA TAATTATATT CAGTAGGTCG GGTGGAATAC CCCCATTAAG AGGGGTATAT   
  
  
- GGTACCCAGG TTTAGCCGCC GGGCCAGTCA AGTTGCTGTC ATGCTACCGT GAGACTTGGG ACACGACGTA   
  
  
- GACGAGTGTT TTTCATGTGG TGTCAACAGA AAGTGAGAGA GACGGGTAAG TGATACGTTT GTGCAAATAT   
  
  
- TTTAACCAAT AAAATATTTG CAAAGAGAAA GTTTACCAAT AAAATATAAT CATTGTTTTT ATAATATTAT   
  
  
- TATTTTTTTA GTTATAATGC ATTAACGATT TTTACTTTAA TGTAGTACAA CATTCTATAA AGTAAACGGA   
  
  
- TAATTATTAA AAAATAATAA ATATTTTAAC CCAACTTAAA GTTATAAAAA ATTTTATTCA GAATAAATAA   
  
  
- ATTCACTTTA AAAAAAATAT CTATTTCAGT GTTTTTAGTA AAAAGATTTA TTAAATATTT TTCTATGAAA   
  
  
- AGGTTATTTT TAATTTAATT TTACGAAAAT ATTTATTTTA ACATTGTTAA GTAAGGTATC GTTTCCATTT   
  
  
- ATTTCGGTAA AAGAATAGTT AATTTAGTTC GAAGAGAAAA AATACTTTAA CAAAAATTAT TTAAGAAAAT   
  
  
- CATTGTTATT CTAAAACAAG TATAAAATTA TGTAGAGGAA CCGTTAACCC GTTTCTACCG ACCATCACTG   
  
  
- TACTTAGGTT CGTACTTAAT CAGGCCCGCA ACAATGAACG ATGTTCCCAA TACTCGTACC TTCCTTTTTA   
  
  
- TTAATTTTTT ATTTTTTTTA ATTTAATGAA GTTAGTGTTT CAACTTTTAT TATTTTAACA CAAACCTTCA   
  
  
- TAAACATGAA TTTCAAAAAA TCATTTTCAC AAAGGGATTT TCCACAAAAA AAATCTCTTT CGTCGATATT   
  
  
- TTACAAAAAA GTTTTTATAA AAAAATAAAT TTTTGTGAAA GAATTTAGTG AACAATATTC AGTGGAATCT   
  
  
- TACTGACTGC CACAACGTGT ATAGTTTTTC GTAAATACGG GATCACGGTA AAATAATAAA ATATAGAATT   
  
  
- AAAATCTATA AAAATAAAAA TAAATTACTA AATATAAACT TGCAATTAAA AGTTATATAG TATAACATAG   
  
  
- AAAATATCGG TGCAAATAAG TTTATATAAA AAATATAAAT ATTTATAAAA AAGATTTTAT ACTAAGCTCT   
  
  
- TACGACTGTA CACTTAGCGT ACCCCCATCC CGTTCGGTAG AACCTGGGTA ATTTGAAGTT TAAGGTACTA   
  
  
- ACTGATCCTG AACGGGTTTT TAAAGTAATG GGGTCTACGG GTGTACACTT ACGTCAGAGA CTGTCTCATC   
  
  
- CACCCTTATT TGGTTTTACG TAAATAAATA AAAAGTTCTA AAATTAATAA TAAGTGGATT TTAGGAAGTA   
  
  
- TCCCTAATTT TATAATCTGG TAACCTAAAC GAGAGGAGTT ATCCCAACTT GAAACTAATC ATGCAAATAT   
  
  
- AATAAAAAAA GGAAAAAGCA GTTTTACGTA AGACAGTGTT TTAATTACAT CAGTTCCTTA ACTGTTCACT   
  
  
- CTTCTCTCAG TGAAATTTAC TAAGTCGACA GGTGTAATTT CTATTCAGTG TAAACACCAG GTATGAGGGG   
  
  
- TTCTGAAATT AAACTTAAAT CGAGTCTGAA AAGCATAAAA TAAACACAAA AAGATAAATT CGATAGTTAT   
  
  
- GTAAATATTT ATTCACTAAA ATTATATTTT CTAGTTCATT TTTTGTTTAT TTCATGTATT TTATAAAAAT   
  
  
- AAGTTAATGT ATAACATAGT TTAAAAGTTC TCGTTTTAAA TATCGTAAAA AGTTACAAAA CCTTTAGTTT   
  
  
- TTTCATTATA TAATAGTCAA GTGATCAATT TAACCACTAA CTTGGTATCT ACTACAGTAT TATATATAAA   
  
  
- ATATAAATAA TTTTAATTTT TATAAATATT CTTTAAAATT TATATACTCT TTAATTTTCA GTTATTATAA   
  
  
- AAAAAGAAGT AGAAATTATA ATAGTTTACT TATATATATA TATATATTTA CATAACTATA AAATTTTAAA   
  
  
- ACAATTTATT TTATATATAT TATACTTCTA TAAATAACTT TCAATAAATT AAATGGATAT ATATATAATT   
  
  
- TTCAATAAAT ACCAACATTT TTTATAATGT AATAAAATAA ATATAATAAT TATAATGTAA TAAAATAAAT   
  
  
- ATAATAAATA TTAAAGTAAT AATATTTTTC AAACTTTTAT AATAACGATA TATTAAACAA CAACACACTT   
  
  
- CTAGCCGTTT TTTTGTCCTC ATCGTCACAC CACAGCAGGA ACGGGGAACT TGACCTCCAA CCAAAGGGTA   
  
  
- AGGAGGGAGG GTGCTTAGGG TGTACATTTC GTTTATTTTC TCGAAATGTA TGTGTACTAA TTACATAAAA   
  
  
- ATAACAGTTA TTTAAAATAT TATTTTGAAT ATTTAGAATA TTTAAATTAT CCTTTTTTTT TTCAGAAGAT   
  
  
- CTTAACTAAA ATATTATGTG GTATGGTTCT AGTGTACTTA ACGTACACAG CCACCTGTTC CAACGGTTTT   
  
  
- AAGGAATAAG GATATGTCTT AGAAAAGTAA CAATATAATA GTATCAGCTT TGGGTCATCA TATATTTCTC   
  
  
- CAATGCTCAC CTCCTACCTA TACTTTATTT ATTCTCCGGA ATCTCTAGTC CCACACTCTG TACCTAAACC   
  
  
- GTTTACCACT CTCTAGATCA CTCG

+     ABRE

| Site Name | Organism | Position | Strand | Matrix score. | sequence | function |
| --- | --- | --- | --- | --- | --- | --- |
| ABRE | Arabidopsis thaliana | 716 | + | 7 | TACGGTC | cis-acting element involved in the abscisic acid responsiveness |
| ABRE | Arabidopsis thaliana | 2250 | - | 5 | ACGTG | cis-acting element involved in the abscisic acid responsiveness |
| ABRE | Arabidopsis thaliana | 1321 | - | 5 | ACGTG | cis-acting element involved in the abscisic acid responsiveness |

>PlantCARE\_32120   
+ AATCATTAAA GTCTAATTCT TCAAAATTCC GAATTCTAAA TTTAGCTATT TAAAATTCCA ATTGTCCTAT   
  
  
+ CTCCGAACTT AATTTTCAGT TTCCCATGCT CCAATTCCCG TATTTACCCC GCTACTTATT ATTATCATTA   
  
  
+ TCATTATCAC CTTATTCATT ATTTCTATAA ATTCCGCCTT CGAATGATTT CTAAGATCTC CAATTTTTAT   
  
  
+ ACATCAACTT TCATAATTTC TACGTCTCAA ATAACATACG ATTCTGATTC CTTTCAAAAT TTAACTCCCA   
  
  
+ TAATTCCAAT TTTCGTAACT TAATTTTTCT TAAAAATCCC GAACTTTCAA AATCCCAATT TTCTTTCAAA   
  
  
+ TTTAATTTCT AAATGTTCTC ATTCTTACAT CTAATTCCTA AGAATCCAAT TTTCAAATAA TCTCTAAGAT   
  
  
+ TCCGATTCTC AAATGAATTT CAAAAATCCA GTTTCCCTTC GAACAATTTT AATCCCTGTT TAGTGATGCA   
  
  
+ TGTAATATGT TTTGTGCTCT ATATGGTGTA CTAACCCTCT CTATTGATAG CGCATGGTGG TTCGTGGCCC   
  
  
+ AGGTATGTAC CCATTTTCTC TCTAATCGTT GTCTATGCAT GTCTCGATTC TTATGTGTGC ACGATCATTC   
  
  
+ AGGATATTCA TTGATTTACT CATCAATTGT CACGGCAGCT TCATTTTATT AGTAGAGACC CGACTTTAGG   
  
  
+ GACTTAGAGG GGTGCTACGG TCCTTACCGT ACCTTCCTGA TAAGTAACCT GACCCCCGAA CCCGATCCGG   
  
  
+ TTTTTCACAG ACCATCTTTT CCAAAATAAG GAGTCACACT TAGGGTTTTT CTTTCTTATT TTGTTTACCC   
  
  
+ TTTAAAAATA AAACAAAAAT AAGTGGCGAC TCCAAGTCAT TTTTCTTAAT CAATAAAGAT CATTTTTTCG   
  
  
+ AAAACGAAAA TCGAGCTCGC CATCGAGTGG GAAACGCATT GAGCCGAAAT GCGGGGTCCA TAGGTATGTT   
  
  
+ TTTTATTTAT GAGATAAAAA GTAAATAATA TTTTTTTCGG GTTTAAAAAA TATATTATTG ATTACTTGTA   
  
  
+ ATTATATGAG CCTCGTTTGT ACTTATTATT AAATACATAT TCAAGGTAAA ATTATCCTTT AAAAAATTCT   
  
  
+ TAGGCCGAGG GAACCTTCCT ATTAATATAA GTCATCCAGC CCACCTTATG GGGGTAATTC TCCCCATATA   
  
  
+ CCATGGGTCC AAATCGGCGG CCCGGTCAGT TCAACGACAG TACGATGGCA CTCTGAACCC TGTGCTGCAT   
  
  
+ CTGCTCACAA AAAGTACACC ACAGTTGTCT TTCACTCTCT CTGCCCATTC ACTATGCAAA CACGTTTATA   
  
  
+ AAATTGGTTA TTTTATAAAC GTTTCTCTTT CAAATGGTTA TTTTATATTA GTAACAAAAA TATTATAATA   
  
  
+ ATAAAAAAAT CAATATTACG TAATTGCTAA AAATGAAATT ACATCATGTT GTAAGATATT TCATTTGCCT   
  
  
+ ATTAATAATT TTTTATTATT TATAAAATTG GGTTGAATTT CAATATTTTT TAAAATAAGT CTTATTTATT   
  
  
+ TAAGTGAAAT TTTTTTTATA GATAAAGTCA CAAAAATCAT TTTTCTAAAT AATTTATAAA AAGATACTTT   
  
  
+ TCCAATAAAA ATTAAATTAA AATGCTTTTA TAAATAAAAT TGTAACAATT CATTCCATAG CAAAGGTAAA   
  
  
+ TAAAGCCATT TTCTTATCAA TTAAATCAAG CTTCTCTTTT TTATGAAATT GTTTTTAATA AATTCTTTTA   
  
  
+ GTAACAATAA GATTTTGTTC ATATTTTAAT ACATCTCCTT GGCAATTGGG CAAAGATGGC TGGTAGTGAC   
  
  
+ ATGAATCCAA GCATGAATTA GTCCGGGCGT TGTTACTTGC TACAAGGGTT ATGAGCATGG AAGGAAAAAT   
  
  
+ AATTAAAAAA TAAAAAAAAT TAAATTACTT CAATCACAAA GTTGAAAATA ATAAAATTGT GTTTGGAAGT   
  
  
+ ATTTGTACTT AAAGTTTTTT AGTAAAAGTG TTTCCCTAAA AGGTGTTTTT TTTAGAGAAA GCAGCTATAA   
  
  
+ AATGTTTTTT CAAAAATATT TTTTTATTTA AAAACACTTT CTTAAATCAC TTGTTATAAG TCACCTTAGA   
  
  
+ ATGACTGACG GTGTTGCACA TATCAAAAAG CATTTATGCC CTAGTGCCAT TTTATTATTT TATATCTTAA   
  
  
+ TTTTAGATAT TTTTATTTTT ATTTAATGAT TTATATTTGA ACGTTAATTT TCAATATATC ATATTGTATC   
  
  
+ TTTTATAGCC ACGTTTATTC AAATATATTT TTTATATTTA TAAATATTTT TTCTAAAATA TGATTCGAGA   
  
  
+ ATGCTGACAT GTGAATCGCA TGGGGGTAGG GCAAGCCATC TTGGACCCAT TAAACTTCAA ATTCCATGAT   
  
  
+ TGACTAGGAC TTGCCCAAAA ATTTCATTAC CCCAGATGCC CACATGTGAA TGCAGTCTCT GACAGAGTAG   
  
  
+ GTGGGAATAA ACCAAAATGC ATTTATTTAT TTTTCAAGAT TTTAATTATT ATTCACCTAA AATCCTTCAT   
  
  
+ AGGGATTAAA ATATTAGACC ATTGGATTTG CTCTCCTCAA TAGGGTTGAA CTTTGATTAG TACGTTTATA   
  
  
+ TTATTTTTTT CCTTTTTCGT CAAAATGCAT TCTGTCACAA AATTAATGTA GTCAAGGAAT TGACAAGTGA   
  
  
+ GAAGAGAGTC ACTTTAAATG ATTCAGCTGT CCACATTAAA GATAAGTCAC ATTTGTGGTC CATACTCCCC   
  
  
+ AAGACTTTAA TTTGAATTTA GCTCAGACTT TTCGTATTTT ATTTGTGTTT TTCTATTTAA GCTATCAATA   
  
  
+ CATTTATAAA TAAGTGATTT TAATATAAAA GATCAAGTAA AAAACAAATA AAGTACATAA AATATTTTTA   
  
  
+ TTCAATTACA TATTGTATCA AATTTTCAAG AGCAAAATTT ATAGCATTTT TCAATGTTTT GGAAATCAAA   
  
  
+ AAAGTAATAT ATTATCAGTT CACTAGTTAA ATTGGTGATT GAACCATAGA TGATGTCATA ATATATATTT   
  
  
+ TATATTTATT AAAATTAAAA ATATTTATAA GAAATTTTAA ATATATGAGA AATTAAAAGT CAATAATATT   
  
  
+ TTTTTCTTCA TCTTTAATAT TATCAAATGA ATATATATAT ATATATAAAT GTATTGATAT TTTAAAATTT   
  
  
+ TGTTAAATAA AATATATATA ATATGAAGAT ATTTATTGAA AGTTATTTAA TTTACCTATA TATATATTAA   
  
  
+ AAGTTATTTA TGGTTGTAAA AAATATTACA TTATTTTATT TATATTATTA ATATTACATT ATTTTATTTA   
  
  
+ TATTATTTAT AATTTCATTA TTATAAAAAG TTTGAAAATA TTATTGCTAT ATAATTTGTT GTTGTGTGAA   
  
  
+ GATCGGCAAA AAAACAGGAG TAGCAGTGTG GTGTCGTCCT TGCCCCTTGA ACTGGAGGTT GGTTTCCCAT   
  
  
+ TCCTCCCTCC CACGAATCCC ACATGTAAAG CAAATAAAAG AGCTTTACAT ACACATGATT AATGTATTTT   
  
  
+ TATTGTCAAT AAATTTTATA ATAAAACTTA TAAATCTTAT AAATTTAATA GGAAAAAAAA AAGTCTTCTA   
  
  
+ GAATTGATTT TATAATACAC CATACCAAGA TCACATGAAT TGCATGTGTC GGTGGACAAG GTTGCCAAAA   
  
  
+ TTCCTTATTC CTATACAGAA TCTTTTCATT GTTATATTAT CATAGTCGAA ACCCAGTAGT ATATAAAGAG   
  
  
+ GTTACGAGTG GAGGATGGAT ATGAAATAAA TAAGAGGCCT TAGAGATCAG GGTGTGAGAC ATGGATTTGG   
  
  
+ CAAATGGTGA GAGATCTAGT GAGC  

- TTAGTAATTT CAGATTAAGA AGTTTTAAGG CTTAAGATTT AAATCGATAA ATTTTAAGGT TAACAGGATA   
  
  
- GAGGCTTGAA TTAAAAGTCA AAGGGTACGA GGTTAAGGGC ATAAATGGGG CGATGAATAA TAATAGTAAT   
  
  
- AGTAATAGTG GAATAAGTAA TAAAGATATT TAAGGCGGAA GCTTACTAAA GATTCTAGAG GTTAAAAATA   
  
  
- TGTAGTTGAA AGTATTAAAG ATGCAGAGTT TATTGTATGC TAAGACTAAG GAAAGTTTTA AATTGAGGGT   
  
  
- ATTAAGGTTA AAAGCATTGA ATTAAAAAGA ATTTTTAGGG CTTGAAAGTT TTAGGGTTAA AAGAAAGTTT   
  
  
- AAATTAAAGA TTTACAAGAG TAAGAATGTA GATTAAGGAT TCTTAGGTTA AAAGTTTATT AGAGATTCTA   
  
  
- AGGCTAAGAG TTTACTTAAA GTTTTTAGGT CAAAGGGAAG CTTGTTAAAA TTAGGGACAA ATCACTACGT   
  
  
- ACATTATACA AAACACGAGA TATACCACAT GATTGGGAGA GATAACTATC GCGTACCACC AAGCACCGGG   
  
  
- TCCATACATG GGTAAAAGAG AGATTAGCAA CAGATACGTA CAGAGCTAAG AATACACACG TGCTAGTAAG   
  
  
- TCCTATAAGT AACTAAATGA GTAGTTAACA GTGCCGTCGA AGTAAAATAA TCATCTCTGG GCTGAAATCC   
  
  
- CTGAATCTCC CCACGATGCC AGGAATGGCA TGGAAGGACT ATTCATTGGA CTGGGGGCTT GGGCTAGGCC   
  
  
- AAAAAGTGTC TGGTAGAAAA GGTTTTATTC CTCAGTGTGA ATCCCAAAAA GAAAGAATAA AACAAATGGG   
  
  
- AAATTTTTAT TTTGTTTTTA TTCACCGCTG AGGTTCAGTA AAAAGAATTA GTTATTTCTA GTAAAAAAGC   
  
  
- TTTTGCTTTT AGCTCGAGCG GTAGCTCACC CTTTGCGTAA CTCGGCTTTA CGCCCCAGGT ATCCATACAA   
  
  
- AAAATAAATA CTCTATTTTT CATTTATTAT AAAAAAAGCC CAAATTTTTT ATATAATAAC TAATGAACAT   
  
  
- TAATATACTC GGAGCAAACA TGAATAATAA TTTATGTATA AGTTCCATTT TAATAGGAAA TTTTTTAAGA   
  
  
- ATCCGGCTCC CTTGGAAGGA TAATTATATT CAGTAGGTCG GGTGGAATAC CCCCATTAAG AGGGGTATAT   
  
  
- GGTACCCAGG TTTAGCCGCC GGGCCAGTCA AGTTGCTGTC ATGCTACCGT GAGACTTGGG ACACGACGTA   
  
  
- GACGAGTGTT TTTCATGTGG TGTCAACAGA AAGTGAGAGA GACGGGTAAG TGATACGTTT GTGCAAATAT   
  
  
- TTTAACCAAT AAAATATTTG CAAAGAGAAA GTTTACCAAT AAAATATAAT CATTGTTTTT ATAATATTAT   
  
  
- TATTTTTTTA GTTATAATGC ATTAACGATT TTTACTTTAA TGTAGTACAA CATTCTATAA AGTAAACGGA   
  
  
- TAATTATTAA AAAATAATAA ATATTTTAAC CCAACTTAAA GTTATAAAAA ATTTTATTCA GAATAAATAA   
  
  
- ATTCACTTTA AAAAAAATAT CTATTTCAGT GTTTTTAGTA AAAAGATTTA TTAAATATTT TTCTATGAAA   
  
  
- AGGTTATTTT TAATTTAATT TTACGAAAAT ATTTATTTTA ACATTGTTAA GTAAGGTATC GTTTCCATTT   
  
  
- ATTTCGGTAA AAGAATAGTT AATTTAGTTC GAAGAGAAAA AATACTTTAA CAAAAATTAT TTAAGAAAAT   
  
  
- CATTGTTATT CTAAAACAAG TATAAAATTA TGTAGAGGAA CCGTTAACCC GTTTCTACCG ACCATCACTG   
  
  
- TACTTAGGTT CGTACTTAAT CAGGCCCGCA ACAATGAACG ATGTTCCCAA TACTCGTACC TTCCTTTTTA   
  
  
- TTAATTTTTT ATTTTTTTTA ATTTAATGAA GTTAGTGTTT CAACTTTTAT TATTTTAACA CAAACCTTCA   
  
  
- TAAACATGAA TTTCAAAAAA TCATTTTCAC AAAGGGATTT TCCACAAAAA AAATCTCTTT CGTCGATATT   
  
  
- TTACAAAAAA GTTTTTATAA AAAAATAAAT TTTTGTGAAA GAATTTAGTG AACAATATTC AGTGGAATCT   
  
  
- TACTGACTGC CACAACGTGT ATAGTTTTTC GTAAATACGG GATCACGGTA AAATAATAAA ATATAGAATT   
  
  
- AAAATCTATA AAAATAAAAA TAAATTACTA AATATAAACT TGCAATTAAA AGTTATATAG TATAACATAG   
  
  
- AAAATATCGG TGCAAATAAG TTTATATAAA AAATATAAAT ATTTATAAAA AAGATTTTAT ACTAAGCTCT   
  
  
- TACGACTGTA CACTTAGCGT ACCCCCATCC CGTTCGGTAG AACCTGGGTA ATTTGAAGTT TAAGGTACTA   
  
  
- ACTGATCCTG AACGGGTTTT TAAAGTAATG GGGTCTACGG GTGTACACTT ACGTCAGAGA CTGTCTCATC   
  
  
- CACCCTTATT TGGTTTTACG TAAATAAATA AAAAGTTCTA AAATTAATAA TAAGTGGATT TTAGGAAGTA   
  
  
- TCCCTAATTT TATAATCTGG TAACCTAAAC GAGAGGAGTT ATCCCAACTT GAAACTAATC ATGCAAATAT   
  
  
- AATAAAAAAA GGAAAAAGCA GTTTTACGTA AGACAGTGTT TTAATTACAT CAGTTCCTTA ACTGTTCACT   
  
  
- CTTCTCTCAG TGAAATTTAC TAAGTCGACA GGTGTAATTT CTATTCAGTG TAAACACCAG GTATGAGGGG   
  
  
- TTCTGAAATT AAACTTAAAT CGAGTCTGAA AAGCATAAAA TAAACACAAA AAGATAAATT CGATAGTTAT   
  
  
- GTAAATATTT ATTCACTAAA ATTATATTTT CTAGTTCATT TTTTGTTTAT TTCATGTATT TTATAAAAAT   
  
  
- AAGTTAATGT ATAACATAGT TTAAAAGTTC TCGTTTTAAA TATCGTAAAA AGTTACAAAA CCTTTAGTTT   
  
  
- TTTCATTATA TAATAGTCAA GTGATCAATT TAACCACTAA CTTGGTATCT ACTACAGTAT TATATATAAA   
  
  
- ATATAAATAA TTTTAATTTT TATAAATATT CTTTAAAATT TATATACTCT TTAATTTTCA GTTATTATAA   
  
  
- AAAAAGAAGT AGAAATTATA ATAGTTTACT TATATATATA TATATATTTA CATAACTATA AAATTTTAAA   
  
  
- ACAATTTATT TTATATATAT TATACTTCTA TAAATAACTT TCAATAAATT AAATGGATAT ATATATAATT   
  
  
- TTCAATAAAT ACCAACATTT TTTATAATGT AATAAAATAA ATATAATAAT TATAATGTAA TAAAATAAAT   
  
  
- ATAATAAATA TTAAAGTAAT AATATTTTTC AAACTTTTAT AATAACGATA TATTAAACAA CAACACACTT   
  
  
- CTAGCCGTTT TTTTGTCCTC ATCGTCACAC CACAGCAGGA ACGGGGAACT TGACCTCCAA CCAAAGGGTA   
  
  
- AGGAGGGAGG GTGCTTAGGG TGTACATTTC GTTTATTTTC TCGAAATGTA TGTGTACTAA TTACATAAAA   
  
  
- ATAACAGTTA TTTAAAATAT TATTTTGAAT ATTTAGAATA TTTAAATTAT CCTTTTTTTT TTCAGAAGAT   
  
  
- CTTAACTAAA ATATTATGTG GTATGGTTCT AGTGTACTTA ACGTACACAG CCACCTGTTC CAACGGTTTT   
  
  
- AAGGAATAAG GATATGTCTT AGAAAAGTAA CAATATAATA GTATCAGCTT TGGGTCATCA TATATTTCTC   
  
  
- CAATGCTCAC CTCCTACCTA TACTTTATTT ATTCTCCGGA ATCTCTAGTC CCACACTCTG TACCTAAACC   
  
  
- GTTTACCACT CTCTAGATCA CTCG

+     ARE

| Site Name | Organism | Position | Strand | Matrix score. | sequence | function |
| --- | --- | --- | --- | --- | --- | --- |
| ARE | Zea mays | 2459 | + | 6 | AAACCA | cis-acting regulatory element essential for the anaerobic induction |
| ARE | Zea mays | 3420 | - | 6 | AAACCA | cis-acting regulatory element essential for the anaerobic induction |

>PlantCARE\_32120   
+ AATCATTAAA GTCTAATTCT TCAAAATTCC GAATTCTAAA TTTAGCTATT TAAAATTCCA ATTGTCCTAT   
  
  
+ CTCCGAACTT AATTTTCAGT TTCCCATGCT CCAATTCCCG TATTTACCCC GCTACTTATT ATTATCATTA   
  
  
+ TCATTATCAC CTTATTCATT ATTTCTATAA ATTCCGCCTT CGAATGATTT CTAAGATCTC CAATTTTTAT   
  
  
+ ACATCAACTT TCATAATTTC TACGTCTCAA ATAACATACG ATTCTGATTC CTTTCAAAAT TTAACTCCCA   
  
  
+ TAATTCCAAT TTTCGTAACT TAATTTTTCT TAAAAATCCC GAACTTTCAA AATCCCAATT TTCTTTCAAA   
  
  
+ TTTAATTTCT AAATGTTCTC ATTCTTACAT CTAATTCCTA AGAATCCAAT TTTCAAATAA TCTCTAAGAT   
  
  
+ TCCGATTCTC AAATGAATTT CAAAAATCCA GTTTCCCTTC GAACAATTTT AATCCCTGTT TAGTGATGCA   
  
  
+ TGTAATATGT TTTGTGCTCT ATATGGTGTA CTAACCCTCT CTATTGATAG CGCATGGTGG TTCGTGGCCC   
  
  
+ AGGTATGTAC CCATTTTCTC TCTAATCGTT GTCTATGCAT GTCTCGATTC TTATGTGTGC ACGATCATTC   
  
  
+ AGGATATTCA TTGATTTACT CATCAATTGT CACGGCAGCT TCATTTTATT AGTAGAGACC CGACTTTAGG   
  
  
+ GACTTAGAGG GGTGCTACGG TCCTTACCGT ACCTTCCTGA TAAGTAACCT GACCCCCGAA CCCGATCCGG   
  
  
+ TTTTTCACAG ACCATCTTTT CCAAAATAAG GAGTCACACT TAGGGTTTTT CTTTCTTATT TTGTTTACCC   
  
  
+ TTTAAAAATA AAACAAAAAT AAGTGGCGAC TCCAAGTCAT TTTTCTTAAT CAATAAAGAT CATTTTTTCG   
  
  
+ AAAACGAAAA TCGAGCTCGC CATCGAGTGG GAAACGCATT GAGCCGAAAT GCGGGGTCCA TAGGTATGTT   
  
  
+ TTTTATTTAT GAGATAAAAA GTAAATAATA TTTTTTTCGG GTTTAAAAAA TATATTATTG ATTACTTGTA   
  
  
+ ATTATATGAG CCTCGTTTGT ACTTATTATT AAATACATAT TCAAGGTAAA ATTATCCTTT AAAAAATTCT   
  
  
+ TAGGCCGAGG GAACCTTCCT ATTAATATAA GTCATCCAGC CCACCTTATG GGGGTAATTC TCCCCATATA   
  
  
+ CCATGGGTCC AAATCGGCGG CCCGGTCAGT TCAACGACAG TACGATGGCA CTCTGAACCC TGTGCTGCAT   
  
  
+ CTGCTCACAA AAAGTACACC ACAGTTGTCT TTCACTCTCT CTGCCCATTC ACTATGCAAA CACGTTTATA   
  
  
+ AAATTGGTTA TTTTATAAAC GTTTCTCTTT CAAATGGTTA TTTTATATTA GTAACAAAAA TATTATAATA   
  
  
+ ATAAAAAAAT CAATATTACG TAATTGCTAA AAATGAAATT ACATCATGTT GTAAGATATT TCATTTGCCT   
  
  
+ ATTAATAATT TTTTATTATT TATAAAATTG GGTTGAATTT CAATATTTTT TAAAATAAGT CTTATTTATT   
  
  
+ TAAGTGAAAT TTTTTTTATA GATAAAGTCA CAAAAATCAT TTTTCTAAAT AATTTATAAA AAGATACTTT   
  
  
+ TCCAATAAAA ATTAAATTAA AATGCTTTTA TAAATAAAAT TGTAACAATT CATTCCATAG CAAAGGTAAA   
  
  
+ TAAAGCCATT TTCTTATCAA TTAAATCAAG CTTCTCTTTT TTATGAAATT GTTTTTAATA AATTCTTTTA   
  
  
+ GTAACAATAA GATTTTGTTC ATATTTTAAT ACATCTCCTT GGCAATTGGG CAAAGATGGC TGGTAGTGAC   
  
  
+ ATGAATCCAA GCATGAATTA GTCCGGGCGT TGTTACTTGC TACAAGGGTT ATGAGCATGG AAGGAAAAAT   
  
  
+ AATTAAAAAA TAAAAAAAAT TAAATTACTT CAATCACAAA GTTGAAAATA ATAAAATTGT GTTTGGAAGT   
  
  
+ ATTTGTACTT AAAGTTTTTT AGTAAAAGTG TTTCCCTAAA AGGTGTTTTT TTTAGAGAAA GCAGCTATAA   
  
  
+ AATGTTTTTT CAAAAATATT TTTTTATTTA AAAACACTTT CTTAAATCAC TTGTTATAAG TCACCTTAGA   
  
  
+ ATGACTGACG GTGTTGCACA TATCAAAAAG CATTTATGCC CTAGTGCCAT TTTATTATTT TATATCTTAA   
  
  
+ TTTTAGATAT TTTTATTTTT ATTTAATGAT TTATATTTGA ACGTTAATTT TCAATATATC ATATTGTATC   
  
  
+ TTTTATAGCC ACGTTTATTC AAATATATTT TTTATATTTA TAAATATTTT TTCTAAAATA TGATTCGAGA   
  
  
+ ATGCTGACAT GTGAATCGCA TGGGGGTAGG GCAAGCCATC TTGGACCCAT TAAACTTCAA ATTCCATGAT   
  
  
+ TGACTAGGAC TTGCCCAAAA ATTTCATTAC CCCAGATGCC CACATGTGAA TGCAGTCTCT GACAGAGTAG   
  
  
+ GTGGGAATAA ACCAAAATGC ATTTATTTAT TTTTCAAGAT TTTAATTATT ATTCACCTAA AATCCTTCAT   
  
  
+ AGGGATTAAA ATATTAGACC ATTGGATTTG CTCTCCTCAA TAGGGTTGAA CTTTGATTAG TACGTTTATA   
  
  
+ TTATTTTTTT CCTTTTTCGT CAAAATGCAT TCTGTCACAA AATTAATGTA GTCAAGGAAT TGACAAGTGA   
  
  
+ GAAGAGAGTC ACTTTAAATG ATTCAGCTGT CCACATTAAA GATAAGTCAC ATTTGTGGTC CATACTCCCC   
  
  
+ AAGACTTTAA TTTGAATTTA GCTCAGACTT TTCGTATTTT ATTTGTGTTT TTCTATTTAA GCTATCAATA   
  
  
+ CATTTATAAA TAAGTGATTT TAATATAAAA GATCAAGTAA AAAACAAATA AAGTACATAA AATATTTTTA   
  
  
+ TTCAATTACA TATTGTATCA AATTTTCAAG AGCAAAATTT ATAGCATTTT TCAATGTTTT GGAAATCAAA   
  
  
+ AAAGTAATAT ATTATCAGTT CACTAGTTAA ATTGGTGATT GAACCATAGA TGATGTCATA ATATATATTT   
  
  
+ TATATTTATT AAAATTAAAA ATATTTATAA GAAATTTTAA ATATATGAGA AATTAAAAGT CAATAATATT   
  
  
+ TTTTTCTTCA TCTTTAATAT TATCAAATGA ATATATATAT ATATATAAAT GTATTGATAT TTTAAAATTT   
  
  
+ TGTTAAATAA AATATATATA ATATGAAGAT ATTTATTGAA AGTTATTTAA TTTACCTATA TATATATTAA   
  
  
+ AAGTTATTTA TGGTTGTAAA AAATATTACA TTATTTTATT TATATTATTA ATATTACATT ATTTTATTTA   
  
  
+ TATTATTTAT AATTTCATTA TTATAAAAAG TTTGAAAATA TTATTGCTAT ATAATTTGTT GTTGTGTGAA   
  
  
+ GATCGGCAAA AAAACAGGAG TAGCAGTGTG GTGTCGTCCT TGCCCCTTGA ACTGGAGGTT GGTTTCCCAT   
  
  
+ TCCTCCCTCC CACGAATCCC ACATGTAAAG CAAATAAAAG AGCTTTACAT ACACATGATT AATGTATTTT   
  
  
+ TATTGTCAAT AAATTTTATA ATAAAACTTA TAAATCTTAT AAATTTAATA GGAAAAAAAA AAGTCTTCTA   
  
  
+ GAATTGATTT TATAATACAC CATACCAAGA TCACATGAAT TGCATGTGTC GGTGGACAAG GTTGCCAAAA   
  
  
+ TTCCTTATTC CTATACAGAA TCTTTTCATT GTTATATTAT CATAGTCGAA ACCCAGTAGT ATATAAAGAG   
  
  
+ GTTACGAGTG GAGGATGGAT ATGAAATAAA TAAGAGGCCT TAGAGATCAG GGTGTGAGAC ATGGATTTGG   
  
  
+ CAAATGGTGA GAGATCTAGT GAGC  

- TTAGTAATTT CAGATTAAGA AGTTTTAAGG CTTAAGATTT AAATCGATAA ATTTTAAGGT TAACAGGATA   
  
  
- GAGGCTTGAA TTAAAAGTCA AAGGGTACGA GGTTAAGGGC ATAAATGGGG CGATGAATAA TAATAGTAAT   
  
  
- AGTAATAGTG GAATAAGTAA TAAAGATATT TAAGGCGGAA GCTTACTAAA GATTCTAGAG GTTAAAAATA   
  
  
- TGTAGTTGAA AGTATTAAAG ATGCAGAGTT TATTGTATGC TAAGACTAAG GAAAGTTTTA AATTGAGGGT   
  
  
- ATTAAGGTTA AAAGCATTGA ATTAAAAAGA ATTTTTAGGG CTTGAAAGTT TTAGGGTTAA AAGAAAGTTT   
  
  
- AAATTAAAGA TTTACAAGAG TAAGAATGTA GATTAAGGAT TCTTAGGTTA AAAGTTTATT AGAGATTCTA   
  
  
- AGGCTAAGAG TTTACTTAAA GTTTTTAGGT CAAAGGGAAG CTTGTTAAAA TTAGGGACAA ATCACTACGT   
  
  
- ACATTATACA AAACACGAGA TATACCACAT GATTGGGAGA GATAACTATC GCGTACCACC AAGCACCGGG   
  
  
- TCCATACATG GGTAAAAGAG AGATTAGCAA CAGATACGTA CAGAGCTAAG AATACACACG TGCTAGTAAG   
  
  
- TCCTATAAGT AACTAAATGA GTAGTTAACA GTGCCGTCGA AGTAAAATAA TCATCTCTGG GCTGAAATCC   
  
  
- CTGAATCTCC CCACGATGCC AGGAATGGCA TGGAAGGACT ATTCATTGGA CTGGGGGCTT GGGCTAGGCC   
  
  
- AAAAAGTGTC TGGTAGAAAA GGTTTTATTC CTCAGTGTGA ATCCCAAAAA GAAAGAATAA AACAAATGGG   
  
  
- AAATTTTTAT TTTGTTTTTA TTCACCGCTG AGGTTCAGTA AAAAGAATTA GTTATTTCTA GTAAAAAAGC   
  
  
- TTTTGCTTTT AGCTCGAGCG GTAGCTCACC CTTTGCGTAA CTCGGCTTTA CGCCCCAGGT ATCCATACAA   
  
  
- AAAATAAATA CTCTATTTTT CATTTATTAT AAAAAAAGCC CAAATTTTTT ATATAATAAC TAATGAACAT   
  
  
- TAATATACTC GGAGCAAACA TGAATAATAA TTTATGTATA AGTTCCATTT TAATAGGAAA TTTTTTAAGA   
  
  
- ATCCGGCTCC CTTGGAAGGA TAATTATATT CAGTAGGTCG GGTGGAATAC CCCCATTAAG AGGGGTATAT   
  
  
- GGTACCCAGG TTTAGCCGCC GGGCCAGTCA AGTTGCTGTC ATGCTACCGT GAGACTTGGG ACACGACGTA   
  
  
- GACGAGTGTT TTTCATGTGG TGTCAACAGA AAGTGAGAGA GACGGGTAAG TGATACGTTT GTGCAAATAT   
  
  
- TTTAACCAAT AAAATATTTG CAAAGAGAAA GTTTACCAAT AAAATATAAT CATTGTTTTT ATAATATTAT   
  
  
- TATTTTTTTA GTTATAATGC ATTAACGATT TTTACTTTAA TGTAGTACAA CATTCTATAA AGTAAACGGA   
  
  
- TAATTATTAA AAAATAATAA ATATTTTAAC CCAACTTAAA GTTATAAAAA ATTTTATTCA GAATAAATAA   
  
  
- ATTCACTTTA AAAAAAATAT CTATTTCAGT GTTTTTAGTA AAAAGATTTA TTAAATATTT TTCTATGAAA   
  
  
- AGGTTATTTT TAATTTAATT TTACGAAAAT ATTTATTTTA ACATTGTTAA GTAAGGTATC GTTTCCATTT   
  
  
- ATTTCGGTAA AAGAATAGTT AATTTAGTTC GAAGAGAAAA AATACTTTAA CAAAAATTAT TTAAGAAAAT   
  
  
- CATTGTTATT CTAAAACAAG TATAAAATTA TGTAGAGGAA CCGTTAACCC GTTTCTACCG ACCATCACTG   
  
  
- TACTTAGGTT CGTACTTAAT CAGGCCCGCA ACAATGAACG ATGTTCCCAA TACTCGTACC TTCCTTTTTA   
  
  
- TTAATTTTTT ATTTTTTTTA ATTTAATGAA GTTAGTGTTT CAACTTTTAT TATTTTAACA CAAACCTTCA   
  
  
- TAAACATGAA TTTCAAAAAA TCATTTTCAC AAAGGGATTT TCCACAAAAA AAATCTCTTT CGTCGATATT   
  
  
- TTACAAAAAA GTTTTTATAA AAAAATAAAT TTTTGTGAAA GAATTTAGTG AACAATATTC AGTGGAATCT   
  
  
- TACTGACTGC CACAACGTGT ATAGTTTTTC GTAAATACGG GATCACGGTA AAATAATAAA ATATAGAATT   
  
  
- AAAATCTATA AAAATAAAAA TAAATTACTA AATATAAACT TGCAATTAAA AGTTATATAG TATAACATAG   
  
  
- AAAATATCGG TGCAAATAAG TTTATATAAA AAATATAAAT ATTTATAAAA AAGATTTTAT ACTAAGCTCT   
  
  
- TACGACTGTA CACTTAGCGT ACCCCCATCC CGTTCGGTAG AACCTGGGTA ATTTGAAGTT TAAGGTACTA   
  
  
- ACTGATCCTG AACGGGTTTT TAAAGTAATG GGGTCTACGG GTGTACACTT ACGTCAGAGA CTGTCTCATC   
  
  
- CACCCTTATT TGGTTTTACG TAAATAAATA AAAAGTTCTA AAATTAATAA TAAGTGGATT TTAGGAAGTA   
  
  
- TCCCTAATTT TATAATCTGG TAACCTAAAC GAGAGGAGTT ATCCCAACTT GAAACTAATC ATGCAAATAT   
  
  
- AATAAAAAAA GGAAAAAGCA GTTTTACGTA AGACAGTGTT TTAATTACAT CAGTTCCTTA ACTGTTCACT   
  
  
- CTTCTCTCAG TGAAATTTAC TAAGTCGACA GGTGTAATTT CTATTCAGTG TAAACACCAG GTATGAGGGG   
  
  
- TTCTGAAATT AAACTTAAAT CGAGTCTGAA AAGCATAAAA TAAACACAAA AAGATAAATT CGATAGTTAT   
  
  
- GTAAATATTT ATTCACTAAA ATTATATTTT CTAGTTCATT TTTTGTTTAT TTCATGTATT TTATAAAAAT   
  
  
- AAGTTAATGT ATAACATAGT TTAAAAGTTC TCGTTTTAAA TATCGTAAAA AGTTACAAAA CCTTTAGTTT   
  
  
- TTTCATTATA TAATAGTCAA GTGATCAATT TAACCACTAA CTTGGTATCT ACTACAGTAT TATATATAAA   
  
  
- ATATAAATAA TTTTAATTTT TATAAATATT CTTTAAAATT TATATACTCT TTAATTTTCA GTTATTATAA   
  
  
- AAAAAGAAGT AGAAATTATA ATAGTTTACT TATATATATA TATATATTTA CATAACTATA AAATTTTAAA   
  
  
- ACAATTTATT TTATATATAT TATACTTCTA TAAATAACTT TCAATAAATT AAATGGATAT ATATATAATT   
  
  
- TTCAATAAAT ACCAACATTT TTTATAATGT AATAAAATAA ATATAATAAT TATAATGTAA TAAAATAAAT   
  
  
- ATAATAAATA TTAAAGTAAT AATATTTTTC AAACTTTTAT AATAACGATA TATTAAACAA CAACACACTT   
  
  
- CTAGCCGTTT TTTTGTCCTC ATCGTCACAC CACAGCAGGA ACGGGGAACT TGACCTCCAA CCAAAGGGTA   
  
  
- AGGAGGGAGG GTGCTTAGGG TGTACATTTC GTTTATTTTC TCGAAATGTA TGTGTACTAA TTACATAAAA   
  
  
- ATAACAGTTA TTTAAAATAT TATTTTGAAT ATTTAGAATA TTTAAATTAT CCTTTTTTTT TTCAGAAGAT   
  
  
- CTTAACTAAA ATATTATGTG GTATGGTTCT AGTGTACTTA ACGTACACAG CCACCTGTTC CAACGGTTTT   
  
  
- AAGGAATAAG GATATGTCTT AGAAAAGTAA CAATATAATA GTATCAGCTT TGGGTCATCA TATATTTCTC   
  
  
- CAATGCTCAC CTCCTACCTA TACTTTATTT ATTCTCCGGA ATCTCTAGTC CCACACTCTG TACCTAAACC   
  
  
- GTTTACCACT CTCTAGATCA CTCG

+     AT1-motif

| Site Name | Organism | Position | Strand | Matrix score. | sequence | function |
| --- | --- | --- | --- | --- | --- | --- |
| AT1-motif | Solanum tuberosum | 1474 | + | 13 | AATTATTTTTTATT | part of a light responsive module |

>PlantCARE\_32120   
+ AATCATTAAA GTCTAATTCT TCAAAATTCC GAATTCTAAA TTTAGCTATT TAAAATTCCA ATTGTCCTAT   
  
  
+ CTCCGAACTT AATTTTCAGT TTCCCATGCT CCAATTCCCG TATTTACCCC GCTACTTATT ATTATCATTA   
  
  
+ TCATTATCAC CTTATTCATT ATTTCTATAA ATTCCGCCTT CGAATGATTT CTAAGATCTC CAATTTTTAT   
  
  
+ ACATCAACTT TCATAATTTC TACGTCTCAA ATAACATACG ATTCTGATTC CTTTCAAAAT TTAACTCCCA   
  
  
+ TAATTCCAAT TTTCGTAACT TAATTTTTCT TAAAAATCCC GAACTTTCAA AATCCCAATT TTCTTTCAAA   
  
  
+ TTTAATTTCT AAATGTTCTC ATTCTTACAT CTAATTCCTA AGAATCCAAT TTTCAAATAA TCTCTAAGAT   
  
  
+ TCCGATTCTC AAATGAATTT CAAAAATCCA GTTTCCCTTC GAACAATTTT AATCCCTGTT TAGTGATGCA   
  
  
+ TGTAATATGT TTTGTGCTCT ATATGGTGTA CTAACCCTCT CTATTGATAG CGCATGGTGG TTCGTGGCCC   
  
  
+ AGGTATGTAC CCATTTTCTC TCTAATCGTT GTCTATGCAT GTCTCGATTC TTATGTGTGC ACGATCATTC   
  
  
+ AGGATATTCA TTGATTTACT CATCAATTGT CACGGCAGCT TCATTTTATT AGTAGAGACC CGACTTTAGG   
  
  
+ GACTTAGAGG GGTGCTACGG TCCTTACCGT ACCTTCCTGA TAAGTAACCT GACCCCCGAA CCCGATCCGG   
  
  
+ TTTTTCACAG ACCATCTTTT CCAAAATAAG GAGTCACACT TAGGGTTTTT CTTTCTTATT TTGTTTACCC   
  
  
+ TTTAAAAATA AAACAAAAAT AAGTGGCGAC TCCAAGTCAT TTTTCTTAAT CAATAAAGAT CATTTTTTCG   
  
  
+ AAAACGAAAA TCGAGCTCGC CATCGAGTGG GAAACGCATT GAGCCGAAAT GCGGGGTCCA TAGGTATGTT   
  
  
+ TTTTATTTAT GAGATAAAAA GTAAATAATA TTTTTTTCGG GTTTAAAAAA TATATTATTG ATTACTTGTA   
  
  
+ ATTATATGAG CCTCGTTTGT ACTTATTATT AAATACATAT TCAAGGTAAA ATTATCCTTT AAAAAATTCT   
  
  
+ TAGGCCGAGG GAACCTTCCT ATTAATATAA GTCATCCAGC CCACCTTATG GGGGTAATTC TCCCCATATA   
  
  
+ CCATGGGTCC AAATCGGCGG CCCGGTCAGT TCAACGACAG TACGATGGCA CTCTGAACCC TGTGCTGCAT   
  
  
+ CTGCTCACAA AAAGTACACC ACAGTTGTCT TTCACTCTCT CTGCCCATTC ACTATGCAAA CACGTTTATA   
  
  
+ AAATTGGTTA TTTTATAAAC GTTTCTCTTT CAAATGGTTA TTTTATATTA GTAACAAAAA TATTATAATA   
  
  
+ ATAAAAAAAT CAATATTACG TAATTGCTAA AAATGAAATT ACATCATGTT GTAAGATATT TCATTTGCCT   
  
  
+ ATTAATAATT TTTTATTATT TATAAAATTG GGTTGAATTT CAATATTTTT TAAAATAAGT CTTATTTATT   
  
  
+ TAAGTGAAAT TTTTTTTATA GATAAAGTCA CAAAAATCAT TTTTCTAAAT AATTTATAAA AAGATACTTT   
  
  
+ TCCAATAAAA ATTAAATTAA AATGCTTTTA TAAATAAAAT TGTAACAATT CATTCCATAG CAAAGGTAAA   
  
  
+ TAAAGCCATT TTCTTATCAA TTAAATCAAG CTTCTCTTTT TTATGAAATT GTTTTTAATA AATTCTTTTA   
  
  
+ GTAACAATAA GATTTTGTTC ATATTTTAAT ACATCTCCTT GGCAATTGGG CAAAGATGGC TGGTAGTGAC   
  
  
+ ATGAATCCAA GCATGAATTA GTCCGGGCGT TGTTACTTGC TACAAGGGTT ATGAGCATGG AAGGAAAAAT   
  
  
+ AATTAAAAAA TAAAAAAAAT TAAATTACTT CAATCACAAA GTTGAAAATA ATAAAATTGT GTTTGGAAGT   
  
  
+ ATTTGTACTT AAAGTTTTTT AGTAAAAGTG TTTCCCTAAA AGGTGTTTTT TTTAGAGAAA GCAGCTATAA   
  
  
+ AATGTTTTTT CAAAAATATT TTTTTATTTA AAAACACTTT CTTAAATCAC TTGTTATAAG TCACCTTAGA   
  
  
+ ATGACTGACG GTGTTGCACA TATCAAAAAG CATTTATGCC CTAGTGCCAT TTTATTATTT TATATCTTAA   
  
  
+ TTTTAGATAT TTTTATTTTT ATTTAATGAT TTATATTTGA ACGTTAATTT TCAATATATC ATATTGTATC   
  
  
+ TTTTATAGCC ACGTTTATTC AAATATATTT TTTATATTTA TAAATATTTT TTCTAAAATA TGATTCGAGA   
  
  
+ ATGCTGACAT GTGAATCGCA TGGGGGTAGG GCAAGCCATC TTGGACCCAT TAAACTTCAA ATTCCATGAT   
  
  
+ TGACTAGGAC TTGCCCAAAA ATTTCATTAC CCCAGATGCC CACATGTGAA TGCAGTCTCT GACAGAGTAG   
  
  
+ GTGGGAATAA ACCAAAATGC ATTTATTTAT TTTTCAAGAT TTTAATTATT ATTCACCTAA AATCCTTCAT   
  
  
+ AGGGATTAAA ATATTAGACC ATTGGATTTG CTCTCCTCAA TAGGGTTGAA CTTTGATTAG TACGTTTATA   
  
  
+ TTATTTTTTT CCTTTTTCGT CAAAATGCAT TCTGTCACAA AATTAATGTA GTCAAGGAAT TGACAAGTGA   
  
  
+ GAAGAGAGTC ACTTTAAATG ATTCAGCTGT CCACATTAAA GATAAGTCAC ATTTGTGGTC CATACTCCCC   
  
  
+ AAGACTTTAA TTTGAATTTA GCTCAGACTT TTCGTATTTT ATTTGTGTTT TTCTATTTAA GCTATCAATA   
  
  
+ CATTTATAAA TAAGTGATTT TAATATAAAA GATCAAGTAA AAAACAAATA AAGTACATAA AATATTTTTA   
  
  
+ TTCAATTACA TATTGTATCA AATTTTCAAG AGCAAAATTT ATAGCATTTT TCAATGTTTT GGAAATCAAA   
  
  
+ AAAGTAATAT ATTATCAGTT CACTAGTTAA ATTGGTGATT GAACCATAGA TGATGTCATA ATATATATTT   
  
  
+ TATATTTATT AAAATTAAAA ATATTTATAA GAAATTTTAA ATATATGAGA AATTAAAAGT CAATAATATT   
  
  
+ TTTTTCTTCA TCTTTAATAT TATCAAATGA ATATATATAT ATATATAAAT GTATTGATAT TTTAAAATTT   
  
  
+ TGTTAAATAA AATATATATA ATATGAAGAT ATTTATTGAA AGTTATTTAA TTTACCTATA TATATATTAA   
  
  
+ AAGTTATTTA TGGTTGTAAA AAATATTACA TTATTTTATT TATATTATTA ATATTACATT ATTTTATTTA   
  
  
+ TATTATTTAT AATTTCATTA TTATAAAAAG TTTGAAAATA TTATTGCTAT ATAATTTGTT GTTGTGTGAA   
  
  
+ GATCGGCAAA AAAACAGGAG TAGCAGTGTG GTGTCGTCCT TGCCCCTTGA ACTGGAGGTT GGTTTCCCAT   
  
  
+ TCCTCCCTCC CACGAATCCC ACATGTAAAG CAAATAAAAG AGCTTTACAT ACACATGATT AATGTATTTT   
  
  
+ TATTGTCAAT AAATTTTATA ATAAAACTTA TAAATCTTAT AAATTTAATA GGAAAAAAAA AAGTCTTCTA   
  
  
+ GAATTGATTT TATAATACAC CATACCAAGA TCACATGAAT TGCATGTGTC GGTGGACAAG GTTGCCAAAA   
  
  
+ TTCCTTATTC CTATACAGAA TCTTTTCATT GTTATATTAT CATAGTCGAA ACCCAGTAGT ATATAAAGAG   
  
  
+ GTTACGAGTG GAGGATGGAT ATGAAATAAA TAAGAGGCCT TAGAGATCAG GGTGTGAGAC ATGGATTTGG   
  
  
+ CAAATGGTGA GAGATCTAGT GAGC  

- TTAGTAATTT CAGATTAAGA AGTTTTAAGG CTTAAGATTT AAATCGATAA ATTTTAAGGT TAACAGGATA   
  
  
- GAGGCTTGAA TTAAAAGTCA AAGGGTACGA GGTTAAGGGC ATAAATGGGG CGATGAATAA TAATAGTAAT   
  
  
- AGTAATAGTG GAATAAGTAA TAAAGATATT TAAGGCGGAA GCTTACTAAA GATTCTAGAG GTTAAAAATA   
  
  
- TGTAGTTGAA AGTATTAAAG ATGCAGAGTT TATTGTATGC TAAGACTAAG GAAAGTTTTA AATTGAGGGT   
  
  
- ATTAAGGTTA AAAGCATTGA ATTAAAAAGA ATTTTTAGGG CTTGAAAGTT TTAGGGTTAA AAGAAAGTTT   
  
  
- AAATTAAAGA TTTACAAGAG TAAGAATGTA GATTAAGGAT TCTTAGGTTA AAAGTTTATT AGAGATTCTA   
  
  
- AGGCTAAGAG TTTACTTAAA GTTTTTAGGT CAAAGGGAAG CTTGTTAAAA TTAGGGACAA ATCACTACGT   
  
  
- ACATTATACA AAACACGAGA TATACCACAT GATTGGGAGA GATAACTATC GCGTACCACC AAGCACCGGG   
  
  
- TCCATACATG GGTAAAAGAG AGATTAGCAA CAGATACGTA CAGAGCTAAG AATACACACG TGCTAGTAAG   
  
  
- TCCTATAAGT AACTAAATGA GTAGTTAACA GTGCCGTCGA AGTAAAATAA TCATCTCTGG GCTGAAATCC   
  
  
- CTGAATCTCC CCACGATGCC AGGAATGGCA TGGAAGGACT ATTCATTGGA CTGGGGGCTT GGGCTAGGCC   
  
  
- AAAAAGTGTC TGGTAGAAAA GGTTTTATTC CTCAGTGTGA ATCCCAAAAA GAAAGAATAA AACAAATGGG   
  
  
- AAATTTTTAT TTTGTTTTTA TTCACCGCTG AGGTTCAGTA AAAAGAATTA GTTATTTCTA GTAAAAAAGC   
  
  
- TTTTGCTTTT AGCTCGAGCG GTAGCTCACC CTTTGCGTAA CTCGGCTTTA CGCCCCAGGT ATCCATACAA   
  
  
- AAAATAAATA CTCTATTTTT CATTTATTAT AAAAAAAGCC CAAATTTTTT ATATAATAAC TAATGAACAT   
  
  
- TAATATACTC GGAGCAAACA TGAATAATAA TTTATGTATA AGTTCCATTT TAATAGGAAA TTTTTTAAGA   
  
  
- ATCCGGCTCC CTTGGAAGGA TAATTATATT CAGTAGGTCG GGTGGAATAC CCCCATTAAG AGGGGTATAT   
  
  
- GGTACCCAGG TTTAGCCGCC GGGCCAGTCA AGTTGCTGTC ATGCTACCGT GAGACTTGGG ACACGACGTA   
  
  
- GACGAGTGTT TTTCATGTGG TGTCAACAGA AAGTGAGAGA GACGGGTAAG TGATACGTTT GTGCAAATAT   
  
  
- TTTAACCAAT AAAATATTTG CAAAGAGAAA GTTTACCAAT AAAATATAAT CATTGTTTTT ATAATATTAT   
  
  
- TATTTTTTTA GTTATAATGC ATTAACGATT TTTACTTTAA TGTAGTACAA CATTCTATAA AGTAAACGGA   
  
  
- TAATTATTAA AAAATAATAA ATATTTTAAC CCAACTTAAA GTTATAAAAA ATTTTATTCA GAATAAATAA   
  
  
- ATTCACTTTA AAAAAAATAT CTATTTCAGT GTTTTTAGTA AAAAGATTTA TTAAATATTT TTCTATGAAA   
  
  
- AGGTTATTTT TAATTTAATT TTACGAAAAT ATTTATTTTA ACATTGTTAA GTAAGGTATC GTTTCCATTT   
  
  
- ATTTCGGTAA AAGAATAGTT AATTTAGTTC GAAGAGAAAA AATACTTTAA CAAAAATTAT TTAAGAAAAT   
  
  
- CATTGTTATT CTAAAACAAG TATAAAATTA TGTAGAGGAA CCGTTAACCC GTTTCTACCG ACCATCACTG   
  
  
- TACTTAGGTT CGTACTTAAT CAGGCCCGCA ACAATGAACG ATGTTCCCAA TACTCGTACC TTCCTTTTTA   
  
  
- TTAATTTTTT ATTTTTTTTA ATTTAATGAA GTTAGTGTTT CAACTTTTAT TATTTTAACA CAAACCTTCA   
  
  
- TAAACATGAA TTTCAAAAAA TCATTTTCAC AAAGGGATTT TCCACAAAAA AAATCTCTTT CGTCGATATT   
  
  
- TTACAAAAAA GTTTTTATAA AAAAATAAAT TTTTGTGAAA GAATTTAGTG AACAATATTC AGTGGAATCT   
  
  
- TACTGACTGC CACAACGTGT ATAGTTTTTC GTAAATACGG GATCACGGTA AAATAATAAA ATATAGAATT   
  
  
- AAAATCTATA AAAATAAAAA TAAATTACTA AATATAAACT TGCAATTAAA AGTTATATAG TATAACATAG   
  
  
- AAAATATCGG TGCAAATAAG TTTATATAAA AAATATAAAT ATTTATAAAA AAGATTTTAT ACTAAGCTCT   
  
  
- TACGACTGTA CACTTAGCGT ACCCCCATCC CGTTCGGTAG AACCTGGGTA ATTTGAAGTT TAAGGTACTA   
  
  
- ACTGATCCTG AACGGGTTTT TAAAGTAATG GGGTCTACGG GTGTACACTT ACGTCAGAGA CTGTCTCATC   
  
  
- CACCCTTATT TGGTTTTACG TAAATAAATA AAAAGTTCTA AAATTAATAA TAAGTGGATT TTAGGAAGTA   
  
  
- TCCCTAATTT TATAATCTGG TAACCTAAAC GAGAGGAGTT ATCCCAACTT GAAACTAATC ATGCAAATAT   
  
  
- AATAAAAAAA GGAAAAAGCA GTTTTACGTA AGACAGTGTT TTAATTACAT CAGTTCCTTA ACTGTTCACT   
  
  
- CTTCTCTCAG TGAAATTTAC TAAGTCGACA GGTGTAATTT CTATTCAGTG TAAACACCAG GTATGAGGGG   
  
  
- TTCTGAAATT AAACTTAAAT CGAGTCTGAA AAGCATAAAA TAAACACAAA AAGATAAATT CGATAGTTAT   
  
  
- GTAAATATTT ATTCACTAAA ATTATATTTT CTAGTTCATT TTTTGTTTAT TTCATGTATT TTATAAAAAT   
  
  
- AAGTTAATGT ATAACATAGT TTAAAAGTTC TCGTTTTAAA TATCGTAAAA AGTTACAAAA CCTTTAGTTT   
  
  
- TTTCATTATA TAATAGTCAA GTGATCAATT TAACCACTAA CTTGGTATCT ACTACAGTAT TATATATAAA   
  
  
- ATATAAATAA TTTTAATTTT TATAAATATT CTTTAAAATT TATATACTCT TTAATTTTCA GTTATTATAA   
  
  
- AAAAAGAAGT AGAAATTATA ATAGTTTACT TATATATATA TATATATTTA CATAACTATA AAATTTTAAA   
  
  
- ACAATTTATT TTATATATAT TATACTTCTA TAAATAACTT TCAATAAATT AAATGGATAT ATATATAATT   
  
  
- TTCAATAAAT ACCAACATTT TTTATAATGT AATAAAATAA ATATAATAAT TATAATGTAA TAAAATAAAT   
  
  
- ATAATAAATA TTAAAGTAAT AATATTTTTC AAACTTTTAT AATAACGATA TATTAAACAA CAACACACTT   
  
  
- CTAGCCGTTT TTTTGTCCTC ATCGTCACAC CACAGCAGGA ACGGGGAACT TGACCTCCAA CCAAAGGGTA   
  
  
- AGGAGGGAGG GTGCTTAGGG TGTACATTTC GTTTATTTTC TCGAAATGTA TGTGTACTAA TTACATAAAA   
  
  
- ATAACAGTTA TTTAAAATAT TATTTTGAAT ATTTAGAATA TTTAAATTAT CCTTTTTTTT TTCAGAAGAT   
  
  
- CTTAACTAAA ATATTATGTG GTATGGTTCT AGTGTACTTA ACGTACACAG CCACCTGTTC CAACGGTTTT   
  
  
- AAGGAATAAG GATATGTCTT AGAAAAGTAA CAATATAATA GTATCAGCTT TGGGTCATCA TATATTTCTC   
  
  
- CAATGCTCAC CTCCTACCTA TACTTTATTT ATTCTCCGGA ATCTCTAGTC CCACACTCTG TACCTAAACC   
  
  
- GTTTACCACT CTCTAGATCA CTCG

+     AT~TATA-box

| Site Name | Organism | Position | Strand | Matrix score. | sequence | function |
| --- | --- | --- | --- | --- | --- | --- |
| AT~TATA-box | Arabidopsis thaliana | 3002 | - | 6 | TATATA |  |
| AT~TATA-box | Arabidopsis thaliana | 3207 | - | 6 | TATATA |  |
| AT~TATA-box | Arabidopsis thaliana | 3118 | - | 6 | TATATA |  |
| AT~TATA-box | Arabidopsis thaliana | 3338 | - | 6 | TATATA |  |
| AT~TATA-box | Arabidopsis thaliana | 3116 | - | 6 | TATATA |  |
| AT~TATA-box | Arabidopsis thaliana | 3209 | - | 6 | TATATA |  |
| AT~TATA-box | Arabidopsis thaliana | 3163 | - | 6 | TATATA |  |
| AT~TATA-box | Arabidopsis thaliana | 3700 | - | 6 | TATATA |  |
| AT~TATA-box | Arabidopsis thaliana | 3165 | - | 6 | TATATA |  |
| AT~TATA-box | Arabidopsis thaliana | 3211 | - | 6 | TATATA |  |
| AT~TATA-box | Arabidopsis thaliana | 3112 | - | 6 | TATATA |  |
| AT~TATA-box | Arabidopsis thaliana | 3120 | - | 6 | TATATA |  |
| AT~TATA-box | Arabidopsis thaliana | 3114 | - | 6 | TATATA |  |
| AT~TATA-box | Arabidopsis thaliana | 3122 | - | 6 | TATATA |  |

>PlantCARE\_32120   
+ AATCATTAAA GTCTAATTCT TCAAAATTCC GAATTCTAAA TTTAGCTATT TAAAATTCCA ATTGTCCTAT   
  
  
+ CTCCGAACTT AATTTTCAGT TTCCCATGCT CCAATTCCCG TATTTACCCC GCTACTTATT ATTATCATTA   
  
  
+ TCATTATCAC CTTATTCATT ATTTCTATAA ATTCCGCCTT CGAATGATTT CTAAGATCTC CAATTTTTAT   
  
  
+ ACATCAACTT TCATAATTTC TACGTCTCAA ATAACATACG ATTCTGATTC CTTTCAAAAT TTAACTCCCA   
  
  
+ TAATTCCAAT TTTCGTAACT TAATTTTTCT TAAAAATCCC GAACTTTCAA AATCCCAATT TTCTTTCAAA   
  
  
+ TTTAATTTCT AAATGTTCTC ATTCTTACAT CTAATTCCTA AGAATCCAAT TTTCAAATAA TCTCTAAGAT   
  
  
+ TCCGATTCTC AAATGAATTT CAAAAATCCA GTTTCCCTTC GAACAATTTT AATCCCTGTT TAGTGATGCA   
  
  
+ TGTAATATGT TTTGTGCTCT ATATGGTGTA CTAACCCTCT CTATTGATAG CGCATGGTGG TTCGTGGCCC   
  
  
+ AGGTATGTAC CCATTTTCTC TCTAATCGTT GTCTATGCAT GTCTCGATTC TTATGTGTGC ACGATCATTC   
  
  
+ AGGATATTCA TTGATTTACT CATCAATTGT CACGGCAGCT TCATTTTATT AGTAGAGACC CGACTTTAGG   
  
  
+ GACTTAGAGG GGTGCTACGG TCCTTACCGT ACCTTCCTGA TAAGTAACCT GACCCCCGAA CCCGATCCGG   
  
  
+ TTTTTCACAG ACCATCTTTT CCAAAATAAG GAGTCACACT TAGGGTTTTT CTTTCTTATT TTGTTTACCC   
  
  
+ TTTAAAAATA AAACAAAAAT AAGTGGCGAC TCCAAGTCAT TTTTCTTAAT CAATAAAGAT CATTTTTTCG   
  
  
+ AAAACGAAAA TCGAGCTCGC CATCGAGTGG GAAACGCATT GAGCCGAAAT GCGGGGTCCA TAGGTATGTT   
  
  
+ TTTTATTTAT GAGATAAAAA GTAAATAATA TTTTTTTCGG GTTTAAAAAA TATATTATTG ATTACTTGTA   
  
  
+ ATTATATGAG CCTCGTTTGT ACTTATTATT AAATACATAT TCAAGGTAAA ATTATCCTTT AAAAAATTCT   
  
  
+ TAGGCCGAGG GAACCTTCCT ATTAATATAA GTCATCCAGC CCACCTTATG GGGGTAATTC TCCCCATATA   
  
  
+ CCATGGGTCC AAATCGGCGG CCCGGTCAGT TCAACGACAG TACGATGGCA CTCTGAACCC TGTGCTGCAT   
  
  
+ CTGCTCACAA AAAGTACACC ACAGTTGTCT TTCACTCTCT CTGCCCATTC ACTATGCAAA CACGTTTATA   
  
  
+ AAATTGGTTA TTTTATAAAC GTTTCTCTTT CAAATGGTTA TTTTATATTA GTAACAAAAA TATTATAATA   
  
  
+ ATAAAAAAAT CAATATTACG TAATTGCTAA AAATGAAATT ACATCATGTT GTAAGATATT TCATTTGCCT   
  
  
+ ATTAATAATT TTTTATTATT TATAAAATTG GGTTGAATTT CAATATTTTT TAAAATAAGT CTTATTTATT   
  
  
+ TAAGTGAAAT TTTTTTTATA GATAAAGTCA CAAAAATCAT TTTTCTAAAT AATTTATAAA AAGATACTTT   
  
  
+ TCCAATAAAA ATTAAATTAA AATGCTTTTA TAAATAAAAT TGTAACAATT CATTCCATAG CAAAGGTAAA   
  
  
+ TAAAGCCATT TTCTTATCAA TTAAATCAAG CTTCTCTTTT TTATGAAATT GTTTTTAATA AATTCTTTTA   
  
  
+ GTAACAATAA GATTTTGTTC ATATTTTAAT ACATCTCCTT GGCAATTGGG CAAAGATGGC TGGTAGTGAC   
  
  
+ ATGAATCCAA GCATGAATTA GTCCGGGCGT TGTTACTTGC TACAAGGGTT ATGAGCATGG AAGGAAAAAT   
  
  
+ AATTAAAAAA TAAAAAAAAT TAAATTACTT CAATCACAAA GTTGAAAATA ATAAAATTGT GTTTGGAAGT   
  
  
+ ATTTGTACTT AAAGTTTTTT AGTAAAAGTG TTTCCCTAAA AGGTGTTTTT TTTAGAGAAA GCAGCTATAA   
  
  
+ AATGTTTTTT CAAAAATATT TTTTTATTTA AAAACACTTT CTTAAATCAC TTGTTATAAG TCACCTTAGA   
  
  
+ ATGACTGACG GTGTTGCACA TATCAAAAAG CATTTATGCC CTAGTGCCAT TTTATTATTT TATATCTTAA   
  
  
+ TTTTAGATAT TTTTATTTTT ATTTAATGAT TTATATTTGA ACGTTAATTT TCAATATATC ATATTGTATC   
  
  
+ TTTTATAGCC ACGTTTATTC AAATATATTT TTTATATTTA TAAATATTTT TTCTAAAATA TGATTCGAGA   
  
  
+ ATGCTGACAT GTGAATCGCA TGGGGGTAGG GCAAGCCATC TTGGACCCAT TAAACTTCAA ATTCCATGAT   
  
  
+ TGACTAGGAC TTGCCCAAAA ATTTCATTAC CCCAGATGCC CACATGTGAA TGCAGTCTCT GACAGAGTAG   
  
  
+ GTGGGAATAA ACCAAAATGC ATTTATTTAT TTTTCAAGAT TTTAATTATT ATTCACCTAA AATCCTTCAT   
  
  
+ AGGGATTAAA ATATTAGACC ATTGGATTTG CTCTCCTCAA TAGGGTTGAA CTTTGATTAG TACGTTTATA   
  
  
+ TTATTTTTTT CCTTTTTCGT CAAAATGCAT TCTGTCACAA AATTAATGTA GTCAAGGAAT TGACAAGTGA   
  
  
+ GAAGAGAGTC ACTTTAAATG ATTCAGCTGT CCACATTAAA GATAAGTCAC ATTTGTGGTC CATACTCCCC   
  
  
+ AAGACTTTAA TTTGAATTTA GCTCAGACTT TTCGTATTTT ATTTGTGTTT TTCTATTTAA GCTATCAATA   
  
  
+ CATTTATAAA TAAGTGATTT TAATATAAAA GATCAAGTAA AAAACAAATA AAGTACATAA AATATTTTTA   
  
  
+ TTCAATTACA TATTGTATCA AATTTTCAAG AGCAAAATTT ATAGCATTTT TCAATGTTTT GGAAATCAAA   
  
  
+ AAAGTAATAT ATTATCAGTT CACTAGTTAA ATTGGTGATT GAACCATAGA TGATGTCATA ATATATATTT   
  
  
+ TATATTTATT AAAATTAAAA ATATTTATAA GAAATTTTAA ATATATGAGA AATTAAAAGT CAATAATATT   
  
  
+ TTTTTCTTCA TCTTTAATAT TATCAAATGA ATATATATAT ATATATAAAT GTATTGATAT TTTAAAATTT   
  
  
+ TGTTAAATAA AATATATATA ATATGAAGAT ATTTATTGAA AGTTATTTAA TTTACCTATA TATATATTAA   
  
  
+ AAGTTATTTA TGGTTGTAAA AAATATTACA TTATTTTATT TATATTATTA ATATTACATT ATTTTATTTA   
  
  
+ TATTATTTAT AATTTCATTA TTATAAAAAG TTTGAAAATA TTATTGCTAT ATAATTTGTT GTTGTGTGAA   
  
  
+ GATCGGCAAA AAAACAGGAG TAGCAGTGTG GTGTCGTCCT TGCCCCTTGA ACTGGAGGTT GGTTTCCCAT   
  
  
+ TCCTCCCTCC CACGAATCCC ACATGTAAAG CAAATAAAAG AGCTTTACAT ACACATGATT AATGTATTTT   
  
  
+ TATTGTCAAT AAATTTTATA ATAAAACTTA TAAATCTTAT AAATTTAATA GGAAAAAAAA AAGTCTTCTA   
  
  
+ GAATTGATTT TATAATACAC CATACCAAGA TCACATGAAT TGCATGTGTC GGTGGACAAG GTTGCCAAAA   
  
  
+ TTCCTTATTC CTATACAGAA TCTTTTCATT GTTATATTAT CATAGTCGAA ACCCAGTAGT ATATAAAGAG   
  
  
+ GTTACGAGTG GAGGATGGAT ATGAAATAAA TAAGAGGCCT TAGAGATCAG GGTGTGAGAC ATGGATTTGG   
  
  
+ CAAATGGTGA GAGATCTAGT GAGC  

- TTAGTAATTT CAGATTAAGA AGTTTTAAGG CTTAAGATTT AAATCGATAA ATTTTAAGGT TAACAGGATA   
  
  
- GAGGCTTGAA TTAAAAGTCA AAGGGTACGA GGTTAAGGGC ATAAATGGGG CGATGAATAA TAATAGTAAT   
  
  
- AGTAATAGTG GAATAAGTAA TAAAGATATT TAAGGCGGAA GCTTACTAAA GATTCTAGAG GTTAAAAATA   
  
  
- TGTAGTTGAA AGTATTAAAG ATGCAGAGTT TATTGTATGC TAAGACTAAG GAAAGTTTTA AATTGAGGGT   
  
  
- ATTAAGGTTA AAAGCATTGA ATTAAAAAGA ATTTTTAGGG CTTGAAAGTT TTAGGGTTAA AAGAAAGTTT   
  
  
- AAATTAAAGA TTTACAAGAG TAAGAATGTA GATTAAGGAT TCTTAGGTTA AAAGTTTATT AGAGATTCTA   
  
  
- AGGCTAAGAG TTTACTTAAA GTTTTTAGGT CAAAGGGAAG CTTGTTAAAA TTAGGGACAA ATCACTACGT   
  
  
- ACATTATACA AAACACGAGA TATACCACAT GATTGGGAGA GATAACTATC GCGTACCACC AAGCACCGGG   
  
  
- TCCATACATG GGTAAAAGAG AGATTAGCAA CAGATACGTA CAGAGCTAAG AATACACACG TGCTAGTAAG   
  
  
- TCCTATAAGT AACTAAATGA GTAGTTAACA GTGCCGTCGA AGTAAAATAA TCATCTCTGG GCTGAAATCC   
  
  
- CTGAATCTCC CCACGATGCC AGGAATGGCA TGGAAGGACT ATTCATTGGA CTGGGGGCTT GGGCTAGGCC   
  
  
- AAAAAGTGTC TGGTAGAAAA GGTTTTATTC CTCAGTGTGA ATCCCAAAAA GAAAGAATAA AACAAATGGG   
  
  
- AAATTTTTAT TTTGTTTTTA TTCACCGCTG AGGTTCAGTA AAAAGAATTA GTTATTTCTA GTAAAAAAGC   
  
  
- TTTTGCTTTT AGCTCGAGCG GTAGCTCACC CTTTGCGTAA CTCGGCTTTA CGCCCCAGGT ATCCATACAA   
  
  
- AAAATAAATA CTCTATTTTT CATTTATTAT AAAAAAAGCC CAAATTTTTT ATATAATAAC TAATGAACAT   
  
  
- TAATATACTC GGAGCAAACA TGAATAATAA TTTATGTATA AGTTCCATTT TAATAGGAAA TTTTTTAAGA   
  
  
- ATCCGGCTCC CTTGGAAGGA TAATTATATT CAGTAGGTCG GGTGGAATAC CCCCATTAAG AGGGGTATAT   
  
  
- GGTACCCAGG TTTAGCCGCC GGGCCAGTCA AGTTGCTGTC ATGCTACCGT GAGACTTGGG ACACGACGTA   
  
  
- GACGAGTGTT TTTCATGTGG TGTCAACAGA AAGTGAGAGA GACGGGTAAG TGATACGTTT GTGCAAATAT   
  
  
- TTTAACCAAT AAAATATTTG CAAAGAGAAA GTTTACCAAT AAAATATAAT CATTGTTTTT ATAATATTAT   
  
  
- TATTTTTTTA GTTATAATGC ATTAACGATT TTTACTTTAA TGTAGTACAA CATTCTATAA AGTAAACGGA   
  
  
- TAATTATTAA AAAATAATAA ATATTTTAAC CCAACTTAAA GTTATAAAAA ATTTTATTCA GAATAAATAA   
  
  
- ATTCACTTTA AAAAAAATAT CTATTTCAGT GTTTTTAGTA AAAAGATTTA TTAAATATTT TTCTATGAAA   
  
  
- AGGTTATTTT TAATTTAATT TTACGAAAAT ATTTATTTTA ACATTGTTAA GTAAGGTATC GTTTCCATTT   
  
  
- ATTTCGGTAA AAGAATAGTT AATTTAGTTC GAAGAGAAAA AATACTTTAA CAAAAATTAT TTAAGAAAAT   
  
  
- CATTGTTATT CTAAAACAAG TATAAAATTA TGTAGAGGAA CCGTTAACCC GTTTCTACCG ACCATCACTG   
  
  
- TACTTAGGTT CGTACTTAAT CAGGCCCGCA ACAATGAACG ATGTTCCCAA TACTCGTACC TTCCTTTTTA   
  
  
- TTAATTTTTT ATTTTTTTTA ATTTAATGAA GTTAGTGTTT CAACTTTTAT TATTTTAACA CAAACCTTCA   
  
  
- TAAACATGAA TTTCAAAAAA TCATTTTCAC AAAGGGATTT TCCACAAAAA AAATCTCTTT CGTCGATATT   
  
  
- TTACAAAAAA GTTTTTATAA AAAAATAAAT TTTTGTGAAA GAATTTAGTG AACAATATTC AGTGGAATCT   
  
  
- TACTGACTGC CACAACGTGT ATAGTTTTTC GTAAATACGG GATCACGGTA AAATAATAAA ATATAGAATT   
  
  
- AAAATCTATA AAAATAAAAA TAAATTACTA AATATAAACT TGCAATTAAA AGTTATATAG TATAACATAG   
  
  
- AAAATATCGG TGCAAATAAG TTTATATAAA AAATATAAAT ATTTATAAAA AAGATTTTAT ACTAAGCTCT   
  
  
- TACGACTGTA CACTTAGCGT ACCCCCATCC CGTTCGGTAG AACCTGGGTA ATTTGAAGTT TAAGGTACTA   
  
  
- ACTGATCCTG AACGGGTTTT TAAAGTAATG GGGTCTACGG GTGTACACTT ACGTCAGAGA CTGTCTCATC   
  
  
- CACCCTTATT TGGTTTTACG TAAATAAATA AAAAGTTCTA AAATTAATAA TAAGTGGATT TTAGGAAGTA   
  
  
- TCCCTAATTT TATAATCTGG TAACCTAAAC GAGAGGAGTT ATCCCAACTT GAAACTAATC ATGCAAATAT   
  
  
- AATAAAAAAA GGAAAAAGCA GTTTTACGTA AGACAGTGTT TTAATTACAT CAGTTCCTTA ACTGTTCACT   
  
  
- CTTCTCTCAG TGAAATTTAC TAAGTCGACA GGTGTAATTT CTATTCAGTG TAAACACCAG GTATGAGGGG   
  
  
- TTCTGAAATT AAACTTAAAT CGAGTCTGAA AAGCATAAAA TAAACACAAA AAGATAAATT CGATAGTTAT   
  
  
- GTAAATATTT ATTCACTAAA ATTATATTTT CTAGTTCATT TTTTGTTTAT TTCATGTATT TTATAAAAAT   
  
  
- AAGTTAATGT ATAACATAGT TTAAAAGTTC TCGTTTTAAA TATCGTAAAA AGTTACAAAA CCTTTAGTTT   
  
  
- TTTCATTATA TAATAGTCAA GTGATCAATT TAACCACTAA CTTGGTATCT ACTACAGTAT TATATATAAA   
  
  
- ATATAAATAA TTTTAATTTT TATAAATATT CTTTAAAATT TATATACTCT TTAATTTTCA GTTATTATAA   
  
  
- AAAAAGAAGT AGAAATTATA ATAGTTTACT TATATATATA TATATATTTA CATAACTATA AAATTTTAAA   
  
  
- ACAATTTATT TTATATATAT TATACTTCTA TAAATAACTT TCAATAAATT AAATGGATAT ATATATAATT   
  
  
- TTCAATAAAT ACCAACATTT TTTATAATGT AATAAAATAA ATATAATAAT TATAATGTAA TAAAATAAAT   
  
  
- ATAATAAATA TTAAAGTAAT AATATTTTTC AAACTTTTAT AATAACGATA TATTAAACAA CAACACACTT   
  
  
- CTAGCCGTTT TTTTGTCCTC ATCGTCACAC CACAGCAGGA ACGGGGAACT TGACCTCCAA CCAAAGGGTA   
  
  
- AGGAGGGAGG GTGCTTAGGG TGTACATTTC GTTTATTTTC TCGAAATGTA TGTGTACTAA TTACATAAAA   
  
  
- ATAACAGTTA TTTAAAATAT TATTTTGAAT ATTTAGAATA TTTAAATTAT CCTTTTTTTT TTCAGAAGAT   
  
  
- CTTAACTAAA ATATTATGTG GTATGGTTCT AGTGTACTTA ACGTACACAG CCACCTGTTC CAACGGTTTT   
  
  
- AAGGAATAAG GATATGTCTT AGAAAAGTAA CAATATAATA GTATCAGCTT TGGGTCATCA TATATTTCTC   
  
  
- CAATGCTCAC CTCCTACCTA TACTTTATTT ATTCTCCGGA ATCTCTAGTC CCACACTCTG TACCTAAACC   
  
  
- GTTTACCACT CTCTAGATCA CTCG

+     AuxRR-core

| Site Name | Organism | Position | Strand | Matrix score. | sequence | function |
| --- | --- | --- | --- | --- | --- | --- |
| AuxRR-core | Nicotiana tabacum | 965 | + | 7 | GGTCCAT | cis-acting regulatory element involved in auxin responsiveness |
| AuxRR-core | Nicotiana tabacum | 2717 | + | 7 | GGTCCAT | cis-acting regulatory element involved in auxin responsiveness |

>PlantCARE\_32120   
+ AATCATTAAA GTCTAATTCT TCAAAATTCC GAATTCTAAA TTTAGCTATT TAAAATTCCA ATTGTCCTAT   
  
  
+ CTCCGAACTT AATTTTCAGT TTCCCATGCT CCAATTCCCG TATTTACCCC GCTACTTATT ATTATCATTA   
  
  
+ TCATTATCAC CTTATTCATT ATTTCTATAA ATTCCGCCTT CGAATGATTT CTAAGATCTC CAATTTTTAT   
  
  
+ ACATCAACTT TCATAATTTC TACGTCTCAA ATAACATACG ATTCTGATTC CTTTCAAAAT TTAACTCCCA   
  
  
+ TAATTCCAAT TTTCGTAACT TAATTTTTCT TAAAAATCCC GAACTTTCAA AATCCCAATT TTCTTTCAAA   
  
  
+ TTTAATTTCT AAATGTTCTC ATTCTTACAT CTAATTCCTA AGAATCCAAT TTTCAAATAA TCTCTAAGAT   
  
  
+ TCCGATTCTC AAATGAATTT CAAAAATCCA GTTTCCCTTC GAACAATTTT AATCCCTGTT TAGTGATGCA   
  
  
+ TGTAATATGT TTTGTGCTCT ATATGGTGTA CTAACCCTCT CTATTGATAG CGCATGGTGG TTCGTGGCCC   
  
  
+ AGGTATGTAC CCATTTTCTC TCTAATCGTT GTCTATGCAT GTCTCGATTC TTATGTGTGC ACGATCATTC   
  
  
+ AGGATATTCA TTGATTTACT CATCAATTGT CACGGCAGCT TCATTTTATT AGTAGAGACC CGACTTTAGG   
  
  
+ GACTTAGAGG GGTGCTACGG TCCTTACCGT ACCTTCCTGA TAAGTAACCT GACCCCCGAA CCCGATCCGG   
  
  
+ TTTTTCACAG ACCATCTTTT CCAAAATAAG GAGTCACACT TAGGGTTTTT CTTTCTTATT TTGTTTACCC   
  
  
+ TTTAAAAATA AAACAAAAAT AAGTGGCGAC TCCAAGTCAT TTTTCTTAAT CAATAAAGAT CATTTTTTCG   
  
  
+ AAAACGAAAA TCGAGCTCGC CATCGAGTGG GAAACGCATT GAGCCGAAAT GCGGGGTCCA TAGGTATGTT   
  
  
+ TTTTATTTAT GAGATAAAAA GTAAATAATA TTTTTTTCGG GTTTAAAAAA TATATTATTG ATTACTTGTA   
  
  
+ ATTATATGAG CCTCGTTTGT ACTTATTATT AAATACATAT TCAAGGTAAA ATTATCCTTT AAAAAATTCT   
  
  
+ TAGGCCGAGG GAACCTTCCT ATTAATATAA GTCATCCAGC CCACCTTATG GGGGTAATTC TCCCCATATA   
  
  
+ CCATGGGTCC AAATCGGCGG CCCGGTCAGT TCAACGACAG TACGATGGCA CTCTGAACCC TGTGCTGCAT   
  
  
+ CTGCTCACAA AAAGTACACC ACAGTTGTCT TTCACTCTCT CTGCCCATTC ACTATGCAAA CACGTTTATA   
  
  
+ AAATTGGTTA TTTTATAAAC GTTTCTCTTT CAAATGGTTA TTTTATATTA GTAACAAAAA TATTATAATA   
  
  
+ ATAAAAAAAT CAATATTACG TAATTGCTAA AAATGAAATT ACATCATGTT GTAAGATATT TCATTTGCCT   
  
  
+ ATTAATAATT TTTTATTATT TATAAAATTG GGTTGAATTT CAATATTTTT TAAAATAAGT CTTATTTATT   
  
  
+ TAAGTGAAAT TTTTTTTATA GATAAAGTCA CAAAAATCAT TTTTCTAAAT AATTTATAAA AAGATACTTT   
  
  
+ TCCAATAAAA ATTAAATTAA AATGCTTTTA TAAATAAAAT TGTAACAATT CATTCCATAG CAAAGGTAAA   
  
  
+ TAAAGCCATT TTCTTATCAA TTAAATCAAG CTTCTCTTTT TTATGAAATT GTTTTTAATA AATTCTTTTA   
  
  
+ GTAACAATAA GATTTTGTTC ATATTTTAAT ACATCTCCTT GGCAATTGGG CAAAGATGGC TGGTAGTGAC   
  
  
+ ATGAATCCAA GCATGAATTA GTCCGGGCGT TGTTACTTGC TACAAGGGTT ATGAGCATGG AAGGAAAAAT   
  
  
+ AATTAAAAAA TAAAAAAAAT TAAATTACTT CAATCACAAA GTTGAAAATA ATAAAATTGT GTTTGGAAGT   
  
  
+ ATTTGTACTT AAAGTTTTTT AGTAAAAGTG TTTCCCTAAA AGGTGTTTTT TTTAGAGAAA GCAGCTATAA   
  
  
+ AATGTTTTTT CAAAAATATT TTTTTATTTA AAAACACTTT CTTAAATCAC TTGTTATAAG TCACCTTAGA   
  
  
+ ATGACTGACG GTGTTGCACA TATCAAAAAG CATTTATGCC CTAGTGCCAT TTTATTATTT TATATCTTAA   
  
  
+ TTTTAGATAT TTTTATTTTT ATTTAATGAT TTATATTTGA ACGTTAATTT TCAATATATC ATATTGTATC   
  
  
+ TTTTATAGCC ACGTTTATTC AAATATATTT TTTATATTTA TAAATATTTT TTCTAAAATA TGATTCGAGA   
  
  
+ ATGCTGACAT GTGAATCGCA TGGGGGTAGG GCAAGCCATC TTGGACCCAT TAAACTTCAA ATTCCATGAT   
  
  
+ TGACTAGGAC TTGCCCAAAA ATTTCATTAC CCCAGATGCC CACATGTGAA TGCAGTCTCT GACAGAGTAG   
  
  
+ GTGGGAATAA ACCAAAATGC ATTTATTTAT TTTTCAAGAT TTTAATTATT ATTCACCTAA AATCCTTCAT   
  
  
+ AGGGATTAAA ATATTAGACC ATTGGATTTG CTCTCCTCAA TAGGGTTGAA CTTTGATTAG TACGTTTATA   
  
  
+ TTATTTTTTT CCTTTTTCGT CAAAATGCAT TCTGTCACAA AATTAATGTA GTCAAGGAAT TGACAAGTGA   
  
  
+ GAAGAGAGTC ACTTTAAATG ATTCAGCTGT CCACATTAAA GATAAGTCAC ATTTGTGGTC CATACTCCCC   
  
  
+ AAGACTTTAA TTTGAATTTA GCTCAGACTT TTCGTATTTT ATTTGTGTTT TTCTATTTAA GCTATCAATA   
  
  
+ CATTTATAAA TAAGTGATTT TAATATAAAA GATCAAGTAA AAAACAAATA AAGTACATAA AATATTTTTA   
  
  
+ TTCAATTACA TATTGTATCA AATTTTCAAG AGCAAAATTT ATAGCATTTT TCAATGTTTT GGAAATCAAA   
  
  
+ AAAGTAATAT ATTATCAGTT CACTAGTTAA ATTGGTGATT GAACCATAGA TGATGTCATA ATATATATTT   
  
  
+ TATATTTATT AAAATTAAAA ATATTTATAA GAAATTTTAA ATATATGAGA AATTAAAAGT CAATAATATT   
  
  
+ TTTTTCTTCA TCTTTAATAT TATCAAATGA ATATATATAT ATATATAAAT GTATTGATAT TTTAAAATTT   
  
  
+ TGTTAAATAA AATATATATA ATATGAAGAT ATTTATTGAA AGTTATTTAA TTTACCTATA TATATATTAA   
  
  
+ AAGTTATTTA TGGTTGTAAA AAATATTACA TTATTTTATT TATATTATTA ATATTACATT ATTTTATTTA   
  
  
+ TATTATTTAT AATTTCATTA TTATAAAAAG TTTGAAAATA TTATTGCTAT ATAATTTGTT GTTGTGTGAA   
  
  
+ GATCGGCAAA AAAACAGGAG TAGCAGTGTG GTGTCGTCCT TGCCCCTTGA ACTGGAGGTT GGTTTCCCAT   
  
  
+ TCCTCCCTCC CACGAATCCC ACATGTAAAG CAAATAAAAG AGCTTTACAT ACACATGATT AATGTATTTT   
  
  
+ TATTGTCAAT AAATTTTATA ATAAAACTTA TAAATCTTAT AAATTTAATA GGAAAAAAAA AAGTCTTCTA   
  
  
+ GAATTGATTT TATAATACAC CATACCAAGA TCACATGAAT TGCATGTGTC GGTGGACAAG GTTGCCAAAA   
  
  
+ TTCCTTATTC CTATACAGAA TCTTTTCATT GTTATATTAT CATAGTCGAA ACCCAGTAGT ATATAAAGAG   
  
  
+ GTTACGAGTG GAGGATGGAT ATGAAATAAA TAAGAGGCCT TAGAGATCAG GGTGTGAGAC ATGGATTTGG   
  
  
+ CAAATGGTGA GAGATCTAGT GAGC  

- TTAGTAATTT CAGATTAAGA AGTTTTAAGG CTTAAGATTT AAATCGATAA ATTTTAAGGT TAACAGGATA   
  
  
- GAGGCTTGAA TTAAAAGTCA AAGGGTACGA GGTTAAGGGC ATAAATGGGG CGATGAATAA TAATAGTAAT   
  
  
- AGTAATAGTG GAATAAGTAA TAAAGATATT TAAGGCGGAA GCTTACTAAA GATTCTAGAG GTTAAAAATA   
  
  
- TGTAGTTGAA AGTATTAAAG ATGCAGAGTT TATTGTATGC TAAGACTAAG GAAAGTTTTA AATTGAGGGT   
  
  
- ATTAAGGTTA AAAGCATTGA ATTAAAAAGA ATTTTTAGGG CTTGAAAGTT TTAGGGTTAA AAGAAAGTTT   
  
  
- AAATTAAAGA TTTACAAGAG TAAGAATGTA GATTAAGGAT TCTTAGGTTA AAAGTTTATT AGAGATTCTA   
  
  
- AGGCTAAGAG TTTACTTAAA GTTTTTAGGT CAAAGGGAAG CTTGTTAAAA TTAGGGACAA ATCACTACGT   
  
  
- ACATTATACA AAACACGAGA TATACCACAT GATTGGGAGA GATAACTATC GCGTACCACC AAGCACCGGG   
  
  
- TCCATACATG GGTAAAAGAG AGATTAGCAA CAGATACGTA CAGAGCTAAG AATACACACG TGCTAGTAAG   
  
  
- TCCTATAAGT AACTAAATGA GTAGTTAACA GTGCCGTCGA AGTAAAATAA TCATCTCTGG GCTGAAATCC   
  
  
- CTGAATCTCC CCACGATGCC AGGAATGGCA TGGAAGGACT ATTCATTGGA CTGGGGGCTT GGGCTAGGCC   
  
  
- AAAAAGTGTC TGGTAGAAAA GGTTTTATTC CTCAGTGTGA ATCCCAAAAA GAAAGAATAA AACAAATGGG   
  
  
- AAATTTTTAT TTTGTTTTTA TTCACCGCTG AGGTTCAGTA AAAAGAATTA GTTATTTCTA GTAAAAAAGC   
  
  
- TTTTGCTTTT AGCTCGAGCG GTAGCTCACC CTTTGCGTAA CTCGGCTTTA CGCCCCAGGT ATCCATACAA   
  
  
- AAAATAAATA CTCTATTTTT CATTTATTAT AAAAAAAGCC CAAATTTTTT ATATAATAAC TAATGAACAT   
  
  
- TAATATACTC GGAGCAAACA TGAATAATAA TTTATGTATA AGTTCCATTT TAATAGGAAA TTTTTTAAGA   
  
  
- ATCCGGCTCC CTTGGAAGGA TAATTATATT CAGTAGGTCG GGTGGAATAC CCCCATTAAG AGGGGTATAT   
  
  
- GGTACCCAGG TTTAGCCGCC GGGCCAGTCA AGTTGCTGTC ATGCTACCGT GAGACTTGGG ACACGACGTA   
  
  
- GACGAGTGTT TTTCATGTGG TGTCAACAGA AAGTGAGAGA GACGGGTAAG TGATACGTTT GTGCAAATAT   
  
  
- TTTAACCAAT AAAATATTTG CAAAGAGAAA GTTTACCAAT AAAATATAAT CATTGTTTTT ATAATATTAT   
  
  
- TATTTTTTTA GTTATAATGC ATTAACGATT TTTACTTTAA TGTAGTACAA CATTCTATAA AGTAAACGGA   
  
  
- TAATTATTAA AAAATAATAA ATATTTTAAC CCAACTTAAA GTTATAAAAA ATTTTATTCA GAATAAATAA   
  
  
- ATTCACTTTA AAAAAAATAT CTATTTCAGT GTTTTTAGTA AAAAGATTTA TTAAATATTT TTCTATGAAA   
  
  
- AGGTTATTTT TAATTTAATT TTACGAAAAT ATTTATTTTA ACATTGTTAA GTAAGGTATC GTTTCCATTT   
  
  
- ATTTCGGTAA AAGAATAGTT AATTTAGTTC GAAGAGAAAA AATACTTTAA CAAAAATTAT TTAAGAAAAT   
  
  
- CATTGTTATT CTAAAACAAG TATAAAATTA TGTAGAGGAA CCGTTAACCC GTTTCTACCG ACCATCACTG   
  
  
- TACTTAGGTT CGTACTTAAT CAGGCCCGCA ACAATGAACG ATGTTCCCAA TACTCGTACC TTCCTTTTTA   
  
  
- TTAATTTTTT ATTTTTTTTA ATTTAATGAA GTTAGTGTTT CAACTTTTAT TATTTTAACA CAAACCTTCA   
  
  
- TAAACATGAA TTTCAAAAAA TCATTTTCAC AAAGGGATTT TCCACAAAAA AAATCTCTTT CGTCGATATT   
  
  
- TTACAAAAAA GTTTTTATAA AAAAATAAAT TTTTGTGAAA GAATTTAGTG AACAATATTC AGTGGAATCT   
  
  
- TACTGACTGC CACAACGTGT ATAGTTTTTC GTAAATACGG GATCACGGTA AAATAATAAA ATATAGAATT   
  
  
- AAAATCTATA AAAATAAAAA TAAATTACTA AATATAAACT TGCAATTAAA AGTTATATAG TATAACATAG   
  
  
- AAAATATCGG TGCAAATAAG TTTATATAAA AAATATAAAT ATTTATAAAA AAGATTTTAT ACTAAGCTCT   
  
  
- TACGACTGTA CACTTAGCGT ACCCCCATCC CGTTCGGTAG AACCTGGGTA ATTTGAAGTT TAAGGTACTA   
  
  
- ACTGATCCTG AACGGGTTTT TAAAGTAATG GGGTCTACGG GTGTACACTT ACGTCAGAGA CTGTCTCATC   
  
  
- CACCCTTATT TGGTTTTACG TAAATAAATA AAAAGTTCTA AAATTAATAA TAAGTGGATT TTAGGAAGTA   
  
  
- TCCCTAATTT TATAATCTGG TAACCTAAAC GAGAGGAGTT ATCCCAACTT GAAACTAATC ATGCAAATAT   
  
  
- AATAAAAAAA GGAAAAAGCA GTTTTACGTA AGACAGTGTT TTAATTACAT CAGTTCCTTA ACTGTTCACT   
  
  
- CTTCTCTCAG TGAAATTTAC TAAGTCGACA GGTGTAATTT CTATTCAGTG TAAACACCAG GTATGAGGGG   
  
  
- TTCTGAAATT AAACTTAAAT CGAGTCTGAA AAGCATAAAA TAAACACAAA AAGATAAATT CGATAGTTAT   
  
  
- GTAAATATTT ATTCACTAAA ATTATATTTT CTAGTTCATT TTTTGTTTAT TTCATGTATT TTATAAAAAT   
  
  
- AAGTTAATGT ATAACATAGT TTAAAAGTTC TCGTTTTAAA TATCGTAAAA AGTTACAAAA CCTTTAGTTT   
  
  
- TTTCATTATA TAATAGTCAA GTGATCAATT TAACCACTAA CTTGGTATCT ACTACAGTAT TATATATAAA   
  
  
- ATATAAATAA TTTTAATTTT TATAAATATT CTTTAAAATT TATATACTCT TTAATTTTCA GTTATTATAA   
  
  
- AAAAAGAAGT AGAAATTATA ATAGTTTACT TATATATATA TATATATTTA CATAACTATA AAATTTTAAA   
  
  
- ACAATTTATT TTATATATAT TATACTTCTA TAAATAACTT TCAATAAATT AAATGGATAT ATATATAATT   
  
  
- TTCAATAAAT ACCAACATTT TTTATAATGT AATAAAATAA ATATAATAAT TATAATGTAA TAAAATAAAT   
  
  
- ATAATAAATA TTAAAGTAAT AATATTTTTC AAACTTTTAT AATAACGATA TATTAAACAA CAACACACTT   
  
  
- CTAGCCGTTT TTTTGTCCTC ATCGTCACAC CACAGCAGGA ACGGGGAACT TGACCTCCAA CCAAAGGGTA   
  
  
- AGGAGGGAGG GTGCTTAGGG TGTACATTTC GTTTATTTTC TCGAAATGTA TGTGTACTAA TTACATAAAA   
  
  
- ATAACAGTTA TTTAAAATAT TATTTTGAAT ATTTAGAATA TTTAAATTAT CCTTTTTTTT TTCAGAAGAT   
  
  
- CTTAACTAAA ATATTATGTG GTATGGTTCT AGTGTACTTA ACGTACACAG CCACCTGTTC CAACGGTTTT   
  
  
- AAGGAATAAG GATATGTCTT AGAAAAGTAA CAATATAATA GTATCAGCTT TGGGTCATCA TATATTTCTC   
  
  
- CAATGCTCAC CTCCTACCTA TACTTTATTT ATTCTCCGGA ATCTCTAGTC CCACACTCTG TACCTAAACC   
  
  
- GTTTACCACT CTCTAGATCA CTCG

+     Box 4

| Site Name | Organism | Position | Strand | Matrix score. | sequence | function |
| --- | --- | --- | --- | --- | --- | --- |
| Box 4 | Petroselinum crispum | 1141 | + | 6 | ATTAAT | part of a conserved DNA module involved in light responsiveness |
| Box 4 | Petroselinum crispum | 3488 | - | 6 | ATTAAT | part of a conserved DNA module involved in light responsiveness |
| Box 4 | Petroselinum crispum | 2632 | - | 6 | ATTAAT | part of a conserved DNA module involved in light responsiveness |
| Box 4 | Petroselinum crispum | 1471 | + | 6 | ATTAAT | part of a conserved DNA module involved in light responsiveness |
| Box 4 | Petroselinum crispum | 3267 | - | 6 | ATTAAT | part of a conserved DNA module involved in light responsiveness |

>PlantCARE\_32120   
+ AATCATTAAA GTCTAATTCT TCAAAATTCC GAATTCTAAA TTTAGCTATT TAAAATTCCA ATTGTCCTAT   
  
  
+ CTCCGAACTT AATTTTCAGT TTCCCATGCT CCAATTCCCG TATTTACCCC GCTACTTATT ATTATCATTA   
  
  
+ TCATTATCAC CTTATTCATT ATTTCTATAA ATTCCGCCTT CGAATGATTT CTAAGATCTC CAATTTTTAT   
  
  
+ ACATCAACTT TCATAATTTC TACGTCTCAA ATAACATACG ATTCTGATTC CTTTCAAAAT TTAACTCCCA   
  
  
+ TAATTCCAAT TTTCGTAACT TAATTTTTCT TAAAAATCCC GAACTTTCAA AATCCCAATT TTCTTTCAAA   
  
  
+ TTTAATTTCT AAATGTTCTC ATTCTTACAT CTAATTCCTA AGAATCCAAT TTTCAAATAA TCTCTAAGAT   
  
  
+ TCCGATTCTC AAATGAATTT CAAAAATCCA GTTTCCCTTC GAACAATTTT AATCCCTGTT TAGTGATGCA   
  
  
+ TGTAATATGT TTTGTGCTCT ATATGGTGTA CTAACCCTCT CTATTGATAG CGCATGGTGG TTCGTGGCCC   
  
  
+ AGGTATGTAC CCATTTTCTC TCTAATCGTT GTCTATGCAT GTCTCGATTC TTATGTGTGC ACGATCATTC   
  
  
+ AGGATATTCA TTGATTTACT CATCAATTGT CACGGCAGCT TCATTTTATT AGTAGAGACC CGACTTTAGG   
  
  
+ GACTTAGAGG GGTGCTACGG TCCTTACCGT ACCTTCCTGA TAAGTAACCT GACCCCCGAA CCCGATCCGG   
  
  
+ TTTTTCACAG ACCATCTTTT CCAAAATAAG GAGTCACACT TAGGGTTTTT CTTTCTTATT TTGTTTACCC   
  
  
+ TTTAAAAATA AAACAAAAAT AAGTGGCGAC TCCAAGTCAT TTTTCTTAAT CAATAAAGAT CATTTTTTCG   
  
  
+ AAAACGAAAA TCGAGCTCGC CATCGAGTGG GAAACGCATT GAGCCGAAAT GCGGGGTCCA TAGGTATGTT   
  
  
+ TTTTATTTAT GAGATAAAAA GTAAATAATA TTTTTTTCGG GTTTAAAAAA TATATTATTG ATTACTTGTA   
  
  
+ ATTATATGAG CCTCGTTTGT ACTTATTATT AAATACATAT TCAAGGTAAA ATTATCCTTT AAAAAATTCT   
  
  
+ TAGGCCGAGG GAACCTTCCT ATTAATATAA GTCATCCAGC CCACCTTATG GGGGTAATTC TCCCCATATA   
  
  
+ CCATGGGTCC AAATCGGCGG CCCGGTCAGT TCAACGACAG TACGATGGCA CTCTGAACCC TGTGCTGCAT   
  
  
+ CTGCTCACAA AAAGTACACC ACAGTTGTCT TTCACTCTCT CTGCCCATTC ACTATGCAAA CACGTTTATA   
  
  
+ AAATTGGTTA TTTTATAAAC GTTTCTCTTT CAAATGGTTA TTTTATATTA GTAACAAAAA TATTATAATA   
  
  
+ ATAAAAAAAT CAATATTACG TAATTGCTAA AAATGAAATT ACATCATGTT GTAAGATATT TCATTTGCCT   
  
  
+ ATTAATAATT TTTTATTATT TATAAAATTG GGTTGAATTT CAATATTTTT TAAAATAAGT CTTATTTATT   
  
  
+ TAAGTGAAAT TTTTTTTATA GATAAAGTCA CAAAAATCAT TTTTCTAAAT AATTTATAAA AAGATACTTT   
  
  
+ TCCAATAAAA ATTAAATTAA AATGCTTTTA TAAATAAAAT TGTAACAATT CATTCCATAG CAAAGGTAAA   
  
  
+ TAAAGCCATT TTCTTATCAA TTAAATCAAG CTTCTCTTTT TTATGAAATT GTTTTTAATA AATTCTTTTA   
  
  
+ GTAACAATAA GATTTTGTTC ATATTTTAAT ACATCTCCTT GGCAATTGGG CAAAGATGGC TGGTAGTGAC   
  
  
+ ATGAATCCAA GCATGAATTA GTCCGGGCGT TGTTACTTGC TACAAGGGTT ATGAGCATGG AAGGAAAAAT   
  
  
+ AATTAAAAAA TAAAAAAAAT TAAATTACTT CAATCACAAA GTTGAAAATA ATAAAATTGT GTTTGGAAGT   
  
  
+ ATTTGTACTT AAAGTTTTTT AGTAAAAGTG TTTCCCTAAA AGGTGTTTTT TTTAGAGAAA GCAGCTATAA   
  
  
+ AATGTTTTTT CAAAAATATT TTTTTATTTA AAAACACTTT CTTAAATCAC TTGTTATAAG TCACCTTAGA   
  
  
+ ATGACTGACG GTGTTGCACA TATCAAAAAG CATTTATGCC CTAGTGCCAT TTTATTATTT TATATCTTAA   
  
  
+ TTTTAGATAT TTTTATTTTT ATTTAATGAT TTATATTTGA ACGTTAATTT TCAATATATC ATATTGTATC   
  
  
+ TTTTATAGCC ACGTTTATTC AAATATATTT TTTATATTTA TAAATATTTT TTCTAAAATA TGATTCGAGA   
  
  
+ ATGCTGACAT GTGAATCGCA TGGGGGTAGG GCAAGCCATC TTGGACCCAT TAAACTTCAA ATTCCATGAT   
  
  
+ TGACTAGGAC TTGCCCAAAA ATTTCATTAC CCCAGATGCC CACATGTGAA TGCAGTCTCT GACAGAGTAG   
  
  
+ GTGGGAATAA ACCAAAATGC ATTTATTTAT TTTTCAAGAT TTTAATTATT ATTCACCTAA AATCCTTCAT   
  
  
+ AGGGATTAAA ATATTAGACC ATTGGATTTG CTCTCCTCAA TAGGGTTGAA CTTTGATTAG TACGTTTATA   
  
  
+ TTATTTTTTT CCTTTTTCGT CAAAATGCAT TCTGTCACAA AATTAATGTA GTCAAGGAAT TGACAAGTGA   
  
  
+ GAAGAGAGTC ACTTTAAATG ATTCAGCTGT CCACATTAAA GATAAGTCAC ATTTGTGGTC CATACTCCCC   
  
  
+ AAGACTTTAA TTTGAATTTA GCTCAGACTT TTCGTATTTT ATTTGTGTTT TTCTATTTAA GCTATCAATA   
  
  
+ CATTTATAAA TAAGTGATTT TAATATAAAA GATCAAGTAA AAAACAAATA AAGTACATAA AATATTTTTA   
  
  
+ TTCAATTACA TATTGTATCA AATTTTCAAG AGCAAAATTT ATAGCATTTT TCAATGTTTT GGAAATCAAA   
  
  
+ AAAGTAATAT ATTATCAGTT CACTAGTTAA ATTGGTGATT GAACCATAGA TGATGTCATA ATATATATTT   
  
  
+ TATATTTATT AAAATTAAAA ATATTTATAA GAAATTTTAA ATATATGAGA AATTAAAAGT CAATAATATT   
  
  
+ TTTTTCTTCA TCTTTAATAT TATCAAATGA ATATATATAT ATATATAAAT GTATTGATAT TTTAAAATTT   
  
  
+ TGTTAAATAA AATATATATA ATATGAAGAT ATTTATTGAA AGTTATTTAA TTTACCTATA TATATATTAA   
  
  
+ AAGTTATTTA TGGTTGTAAA AAATATTACA TTATTTTATT TATATTATTA ATATTACATT ATTTTATTTA   
  
  
+ TATTATTTAT AATTTCATTA TTATAAAAAG TTTGAAAATA TTATTGCTAT ATAATTTGTT GTTGTGTGAA   
  
  
+ GATCGGCAAA AAAACAGGAG TAGCAGTGTG GTGTCGTCCT TGCCCCTTGA ACTGGAGGTT GGTTTCCCAT   
  
  
+ TCCTCCCTCC CACGAATCCC ACATGTAAAG CAAATAAAAG AGCTTTACAT ACACATGATT AATGTATTTT   
  
  
+ TATTGTCAAT AAATTTTATA ATAAAACTTA TAAATCTTAT AAATTTAATA GGAAAAAAAA AAGTCTTCTA   
  
  
+ GAATTGATTT TATAATACAC CATACCAAGA TCACATGAAT TGCATGTGTC GGTGGACAAG GTTGCCAAAA   
  
  
+ TTCCTTATTC CTATACAGAA TCTTTTCATT GTTATATTAT CATAGTCGAA ACCCAGTAGT ATATAAAGAG   
  
  
+ GTTACGAGTG GAGGATGGAT ATGAAATAAA TAAGAGGCCT TAGAGATCAG GGTGTGAGAC ATGGATTTGG   
  
  
+ CAAATGGTGA GAGATCTAGT GAGC  

- TTAGTAATTT CAGATTAAGA AGTTTTAAGG CTTAAGATTT AAATCGATAA ATTTTAAGGT TAACAGGATA   
  
  
- GAGGCTTGAA TTAAAAGTCA AAGGGTACGA GGTTAAGGGC ATAAATGGGG CGATGAATAA TAATAGTAAT   
  
  
- AGTAATAGTG GAATAAGTAA TAAAGATATT TAAGGCGGAA GCTTACTAAA GATTCTAGAG GTTAAAAATA   
  
  
- TGTAGTTGAA AGTATTAAAG ATGCAGAGTT TATTGTATGC TAAGACTAAG GAAAGTTTTA AATTGAGGGT   
  
  
- ATTAAGGTTA AAAGCATTGA ATTAAAAAGA ATTTTTAGGG CTTGAAAGTT TTAGGGTTAA AAGAAAGTTT   
  
  
- AAATTAAAGA TTTACAAGAG TAAGAATGTA GATTAAGGAT TCTTAGGTTA AAAGTTTATT AGAGATTCTA   
  
  
- AGGCTAAGAG TTTACTTAAA GTTTTTAGGT CAAAGGGAAG CTTGTTAAAA TTAGGGACAA ATCACTACGT   
  
  
- ACATTATACA AAACACGAGA TATACCACAT GATTGGGAGA GATAACTATC GCGTACCACC AAGCACCGGG   
  
  
- TCCATACATG GGTAAAAGAG AGATTAGCAA CAGATACGTA CAGAGCTAAG AATACACACG TGCTAGTAAG   
  
  
- TCCTATAAGT AACTAAATGA GTAGTTAACA GTGCCGTCGA AGTAAAATAA TCATCTCTGG GCTGAAATCC   
  
  
- CTGAATCTCC CCACGATGCC AGGAATGGCA TGGAAGGACT ATTCATTGGA CTGGGGGCTT GGGCTAGGCC   
  
  
- AAAAAGTGTC TGGTAGAAAA GGTTTTATTC CTCAGTGTGA ATCCCAAAAA GAAAGAATAA AACAAATGGG   
  
  
- AAATTTTTAT TTTGTTTTTA TTCACCGCTG AGGTTCAGTA AAAAGAATTA GTTATTTCTA GTAAAAAAGC   
  
  
- TTTTGCTTTT AGCTCGAGCG GTAGCTCACC CTTTGCGTAA CTCGGCTTTA CGCCCCAGGT ATCCATACAA   
  
  
- AAAATAAATA CTCTATTTTT CATTTATTAT AAAAAAAGCC CAAATTTTTT ATATAATAAC TAATGAACAT   
  
  
- TAATATACTC GGAGCAAACA TGAATAATAA TTTATGTATA AGTTCCATTT TAATAGGAAA TTTTTTAAGA   
  
  
- ATCCGGCTCC CTTGGAAGGA TAATTATATT CAGTAGGTCG GGTGGAATAC CCCCATTAAG AGGGGTATAT   
  
  
- GGTACCCAGG TTTAGCCGCC GGGCCAGTCA AGTTGCTGTC ATGCTACCGT GAGACTTGGG ACACGACGTA   
  
  
- GACGAGTGTT TTTCATGTGG TGTCAACAGA AAGTGAGAGA GACGGGTAAG TGATACGTTT GTGCAAATAT   
  
  
- TTTAACCAAT AAAATATTTG CAAAGAGAAA GTTTACCAAT AAAATATAAT CATTGTTTTT ATAATATTAT   
  
  
- TATTTTTTTA GTTATAATGC ATTAACGATT TTTACTTTAA TGTAGTACAA CATTCTATAA AGTAAACGGA   
  
  
- TAATTATTAA AAAATAATAA ATATTTTAAC CCAACTTAAA GTTATAAAAA ATTTTATTCA GAATAAATAA   
  
  
- ATTCACTTTA AAAAAAATAT CTATTTCAGT GTTTTTAGTA AAAAGATTTA TTAAATATTT TTCTATGAAA   
  
  
- AGGTTATTTT TAATTTAATT TTACGAAAAT ATTTATTTTA ACATTGTTAA GTAAGGTATC GTTTCCATTT   
  
  
- ATTTCGGTAA AAGAATAGTT AATTTAGTTC GAAGAGAAAA AATACTTTAA CAAAAATTAT TTAAGAAAAT   
  
  
- CATTGTTATT CTAAAACAAG TATAAAATTA TGTAGAGGAA CCGTTAACCC GTTTCTACCG ACCATCACTG   
  
  
- TACTTAGGTT CGTACTTAAT CAGGCCCGCA ACAATGAACG ATGTTCCCAA TACTCGTACC TTCCTTTTTA   
  
  
- TTAATTTTTT ATTTTTTTTA ATTTAATGAA GTTAGTGTTT CAACTTTTAT TATTTTAACA CAAACCTTCA   
  
  
- TAAACATGAA TTTCAAAAAA TCATTTTCAC AAAGGGATTT TCCACAAAAA AAATCTCTTT CGTCGATATT   
  
  
- TTACAAAAAA GTTTTTATAA AAAAATAAAT TTTTGTGAAA GAATTTAGTG AACAATATTC AGTGGAATCT   
  
  
- TACTGACTGC CACAACGTGT ATAGTTTTTC GTAAATACGG GATCACGGTA AAATAATAAA ATATAGAATT   
  
  
- AAAATCTATA AAAATAAAAA TAAATTACTA AATATAAACT TGCAATTAAA AGTTATATAG TATAACATAG   
  
  
- AAAATATCGG TGCAAATAAG TTTATATAAA AAATATAAAT ATTTATAAAA AAGATTTTAT ACTAAGCTCT   
  
  
- TACGACTGTA CACTTAGCGT ACCCCCATCC CGTTCGGTAG AACCTGGGTA ATTTGAAGTT TAAGGTACTA   
  
  
- ACTGATCCTG AACGGGTTTT TAAAGTAATG GGGTCTACGG GTGTACACTT ACGTCAGAGA CTGTCTCATC   
  
  
- CACCCTTATT TGGTTTTACG TAAATAAATA AAAAGTTCTA AAATTAATAA TAAGTGGATT TTAGGAAGTA   
  
  
- TCCCTAATTT TATAATCTGG TAACCTAAAC GAGAGGAGTT ATCCCAACTT GAAACTAATC ATGCAAATAT   
  
  
- AATAAAAAAA GGAAAAAGCA GTTTTACGTA AGACAGTGTT TTAATTACAT CAGTTCCTTA ACTGTTCACT   
  
  
- CTTCTCTCAG TGAAATTTAC TAAGTCGACA GGTGTAATTT CTATTCAGTG TAAACACCAG GTATGAGGGG   
  
  
- TTCTGAAATT AAACTTAAAT CGAGTCTGAA AAGCATAAAA TAAACACAAA AAGATAAATT CGATAGTTAT   
  
  
- GTAAATATTT ATTCACTAAA ATTATATTTT CTAGTTCATT TTTTGTTTAT TTCATGTATT TTATAAAAAT   
  
  
- AAGTTAATGT ATAACATAGT TTAAAAGTTC TCGTTTTAAA TATCGTAAAA AGTTACAAAA CCTTTAGTTT   
  
  
- TTTCATTATA TAATAGTCAA GTGATCAATT TAACCACTAA CTTGGTATCT ACTACAGTAT TATATATAAA   
  
  
- ATATAAATAA TTTTAATTTT TATAAATATT CTTTAAAATT TATATACTCT TTAATTTTCA GTTATTATAA   
  
  
- AAAAAGAAGT AGAAATTATA ATAGTTTACT TATATATATA TATATATTTA CATAACTATA AAATTTTAAA   
  
  
- ACAATTTATT TTATATATAT TATACTTCTA TAAATAACTT TCAATAAATT AAATGGATAT ATATATAATT   
  
  
- TTCAATAAAT ACCAACATTT TTTATAATGT AATAAAATAA ATATAATAAT TATAATGTAA TAAAATAAAT   
  
  
- ATAATAAATA TTAAAGTAAT AATATTTTTC AAACTTTTAT AATAACGATA TATTAAACAA CAACACACTT   
  
  
- CTAGCCGTTT TTTTGTCCTC ATCGTCACAC CACAGCAGGA ACGGGGAACT TGACCTCCAA CCAAAGGGTA   
  
  
- AGGAGGGAGG GTGCTTAGGG TGTACATTTC GTTTATTTTC TCGAAATGTA TGTGTACTAA TTACATAAAA   
  
  
- ATAACAGTTA TTTAAAATAT TATTTTGAAT ATTTAGAATA TTTAAATTAT CCTTTTTTTT TTCAGAAGAT   
  
  
- CTTAACTAAA ATATTATGTG GTATGGTTCT AGTGTACTTA ACGTACACAG CCACCTGTTC CAACGGTTTT   
  
  
- AAGGAATAAG GATATGTCTT AGAAAAGTAA CAATATAATA GTATCAGCTT TGGGTCATCA TATATTTCTC   
  
  
- CAATGCTCAC CTCCTACCTA TACTTTATTT ATTCTCCGGA ATCTCTAGTC CCACACTCTG TACCTAAACC   
  
  
- GTTTACCACT CTCTAGATCA CTCG

+     CAAT-box

| Site Name | Organism | Position | Strand | Matrix score. | sequence | function |
| --- | --- | --- | --- | --- | --- | --- |
| CAAT-box | Arabidopsis thaliana | 396 | + | 5 | CCAAT | common cis-acting element in promoter and enhancer regions |
| CAAT-box | Nicotiana glutinosa | 1511 | + | 4 | CAAT |  |
| CAAT-box | Pisum sativum | 404 | + | 5 | CAAAT | common cis-acting element in promoter and enhancer regions |
| CAAT-box | Nicotiana glutinosa | 3071 | + | 4 | CAAT |  |
| CAAT-box | Arabidopsis thaliana | 335 | + | 5 | CCAAT | common cis-acting element in promoter and enhancer regions |
| CAAT-box | Nicotiana glutinosa | 1793 | + | 4 | CAAT |  |
| CAAT-box | Nicotiana glutinosa | 948 | - | 4 | CAAT |  |
| CAAT-box | Nicotiana glutinosa | 3507 | + | 4 | CAAT |  |
| CAAT-box | Arabidopsis thaliana | 334 | + | 8 | CCCAATTT | common cis-acting element in promoter and enhancer regions |
| CAAT-box | Nicotiana glutinosa | 1656 | + | 4 | CAAT |  |
| CAAT-box | Nicotiana glutinosa | 640 | - | 4 | CAAT |  |
| CAAT-box | Nicotiana glutinosa | 3333 | - | 4 | CAAT |  |
| CAAT-box | Arabidopsis thaliana | 286 | + | 5 | CCAAT | common cis-acting element in promoter and enhancer regions |
| CAAT-box | Pisum sativum | 1961 | - | 5 | CAAAT | common cis-acting element in promoter and enhancer regions |
| CAAT-box | Nicotiana glutinosa | 1423 | - | 4 | CAAT |  |
| CAAT-box | Pisum sativum | 3775 | - | 5 | CAAAT | common cis-acting element in promoter and enhancer regions |
| CAAT-box | Arabidopsis thaliana | 101 | + | 5 | CCAAT | common cis-acting element in promoter and enhancer regions |
| CAAT-box | Nicotiana glutinosa | 1613 | + | 4 | CAAT |  |
| CAAT-box | Nicotiana glutinosa | 656 | - | 4 | CAAT |  |
| CAAT-box | Nicotiana glutinosa | 3133 | - | 4 | CAAT |  |
| CAAT-box | Arabidopsis thaliana | 58 | + | 5 | CCAAT | common cis-acting element in promoter and enhancer regions |
| CAAT-box | Nicotiana glutinosa | 1921 | + | 4 | CAAT |  |
| CAAT-box | Pisum sativum | 1200 | + | 5 | CAAAT | common cis-acting element in promoter and enhancer regions |
| CAAT-box | Nicotiana glutinosa | 3609 | - | 4 | CAAT |  |
| CAAT-box | Arabidopsis thaliana | 200 | + | 5 | CCAAT | common cis-acting element in promoter and enhancer regions |
| CAAT-box | Nicotiana glutinosa | 1728 | - | 4 | CAAT |  |
| CAAT-box | Nicotiana glutinosa | 654 | + | 4 | CAAT |  |
| CAAT-box | Pisum sativum | 3461 | + | 5 | CAAAT | common cis-acting element in promoter and enhancer regions |
| CAAT-box | Nicotiana glutinosa | 336 | + | 4 | CAAT |  |
| CAAT-box | Arabidopsis thaliana | 2971 | - | 5 | CCAAT | common cis-acting element in promoter and enhancer regions |
| CAAT-box | Arabidopsis thaliana | 1495 | - | 8 | CCCAATTT | common cis-acting element in promoter and enhancer regions |
| CAAT-box | Pisum sativum | 3781 | + | 5 | CAAAT | common cis-acting element in promoter and enhancer regions |
| CAAT-box | Nicotiana glutinosa | 102 | + | 4 | CAAT |  |
| CAAT-box | Arabidopsis thaliana | 1612 | + | 5 | CCAAT | common cis-acting element in promoter and enhancer regions |
| CAAT-box | Nicotiana glutinosa | 464 | + | 4 | CAAT |  |
| CAAT-box | Pisum sativum | 3104 | + | 5 | CAAAT | common cis-acting element in promoter and enhancer regions |
| CAAT-box | Nicotiana glutinosa | 287 | + | 4 | CAAT |  |
| CAAT-box | Arabidopsis thaliana | 1795 | - | 5 | CCAAT | common cis-acting element in promoter and enhancer regions |
| CAAT-box | Nicotiana glutinosa | 1037 | - | 4 | CAAT |  |
| CAAT-box | Nicotiana glutinosa | 3573 | - | 4 | CAAT |  |
| CAAT-box | Nicotiana glutinosa | 201 | + | 4 | CAAT |  |
| CAAT-box | Nicotiana glutinosa | 1698 | + | 4 | CAAT |  |
| CAAT-box | Pisum sativum | 430 | + | 5 | CAAAT | common cis-acting element in promoter and enhancer regions |
| CAAT-box | Pisum sativum | 3344 | - | 5 | CAAAT | common cis-acting element in promoter and enhancer regions |
| CAAT-box | Pisum sativum | 347 | + | 5 | CAAAT | common cis-acting element in promoter and enhancer regions |
| CAAT-box | Pisum sativum | 2205 | - | 5 | CAAAT | common cis-acting element in promoter and enhancer regions |
| CAAT-box | Pisum sativum | 1463 | - | 5 | CAAAT | common cis-acting element in promoter and enhancer regions |
| CAAT-box | Nicotiana glutinosa | 3668 | - | 4 | CAAT |  |
| CAAT-box | Nicotiana glutinosa | 61 | - | 4 | CAAT |  |
| CAAT-box | Nicotiana glutinosa | 1649 | - | 4 | CAAT |  |
| CAAT-box | Nicotiana glutinosa | 891 | + | 4 | CAAT |  |
| CAAT-box | Nicotiana glutinosa | 3185 | - | 4 | CAAT |  |
| CAAT-box | Nicotiana glutinosa | 397 | + | 4 | CAAT |  |
| CAAT-box | Nicotiana glutinosa | 1946 | - | 4 | CAAT |  |
| CAAT-box | Arabidopsis thaliana | 1333 | - | 5 | CCAAT | common cis-acting element in promoter and enhancer regions |
| CAAT-box | Nicotiana glutinosa | 3502 | - | 4 | CAAT |  |
| CAAT-box | Pisum sativum | 238 | + | 5 | CAAAT | common cis-acting element in promoter and enhancer regions |
| CAAT-box | Nicotiana glutinosa | 1755 | + | 4 | CAAT |  |
| CAAT-box | Nicotiana glutinosa | 533 | - | 4 | CAAT |  |
| CAAT-box | Nicotiana glutinosa | 2978 | - | 4 | CAAT |  |
| CAAT-box | Nicotiana glutinosa | 59 | + | 4 | CAAT |  |
| CAAT-box | Nicotiana glutinosa | 2222 | + | 4 | CAAT |  |
| CAAT-box | Arabidopsis thaliana | 1497 | - | 5 | CCAAT | common cis-acting element in promoter and enhancer regions |
| CAAT-box | Nicotiana glutinosa | 2233 | - | 4 | CAAT |  |
| CAAT-box | Pisum sativum | 1361 | + | 5 | CAAAT | common cis-acting element in promoter and enhancer regions |
| CAAT-box | Pisum sativum | 2260 | + | 5 | CAAAT | common cis-acting element in promoter and enhancer regions |
| CAAT-box | Nicotiana glutinosa | 1411 | + | 4 | CAAT |  |
| CAAT-box | Pisum sativum | 2368 | + | 5 | CAAAT | common cis-acting element in promoter and enhancer regions |
| CAAT-box | Nicotiana glutinosa | 2379 | - | 4 | CAAT |  |
| CAAT-box | Arabidopsis thaliana | 2541 | - | 5 | CCAAT | common cis-acting element in promoter and enhancer regions |
| CAAT-box | Pisum sativum | 2546 | - | 5 | CAAAT | common cis-acting element in promoter and enhancer regions |
| CAAT-box | Nicotiana glutinosa | 2558 | + | 4 | CAAT |  |
| CAAT-box | Nicotiana glutinosa | 2649 | - | 4 | CAAT |  |
| CAAT-box | Pisum sativum | 2711 | - | 5 | CAAAT | common cis-acting element in promoter and enhancer regions |
| CAAT-box | Pisum sativum | 2740 | - | 5 | CAAAT | common cis-acting element in promoter and enhancer regions |
| CAAT-box | Pisum sativum | 2771 | - | 5 | CAAAT | common cis-acting element in promoter and enhancer regions |
| CAAT-box | Nicotiana glutinosa | 2796 | + | 4 | CAAT |  |
| CAAT-box | Pisum sativum | 2845 | + | 5 | CAAAT | common cis-acting element in promoter and enhancer regions |
| CAAT-box | Nicotiana glutinosa | 2873 | + | 4 | CAAT |  |
| CAAT-box | Nicotiana glutinosa | 2882 | - | 4 | CAAT |  |
| CAAT-box | Pisum sativum | 2889 | + | 5 | CAAAT | common cis-acting element in promoter and enhancer regions |
| CAAT-box | Nicotiana glutinosa | 2922 | + | 4 | CAAT |  |

>PlantCARE\_32120   
+ AATCATTAAA GTCTAATTCT TCAAAATTCC GAATTCTAAA TTTAGCTATT TAAAATTCCA ATTGTCCTAT   
  
  
+ CTCCGAACTT AATTTTCAGT TTCCCATGCT CCAATTCCCG TATTTACCCC GCTACTTATT ATTATCATTA   
  
  
+ TCATTATCAC CTTATTCATT ATTTCTATAA ATTCCGCCTT CGAATGATTT CTAAGATCTC CAATTTTTAT   
  
  
+ ACATCAACTT TCATAATTTC TACGTCTCAA ATAACATACG ATTCTGATTC CTTTCAAAAT TTAACTCCCA   
  
  
+ TAATTCCAAT TTTCGTAACT TAATTTTTCT TAAAAATCCC GAACTTTCAA AATCCCAATT TTCTTTCAAA   
  
  
+ TTTAATTTCT AAATGTTCTC ATTCTTACAT CTAATTCCTA AGAATCCAAT TTTCAAATAA TCTCTAAGAT   
  
  
+ TCCGATTCTC AAATGAATTT CAAAAATCCA GTTTCCCTTC GAACAATTTT AATCCCTGTT TAGTGATGCA   
  
  
+ TGTAATATGT TTTGTGCTCT ATATGGTGTA CTAACCCTCT CTATTGATAG CGCATGGTGG TTCGTGGCCC   
  
  
+ AGGTATGTAC CCATTTTCTC TCTAATCGTT GTCTATGCAT GTCTCGATTC TTATGTGTGC ACGATCATTC   
  
  
+ AGGATATTCA TTGATTTACT CATCAATTGT CACGGCAGCT TCATTTTATT AGTAGAGACC CGACTTTAGG   
  
  
+ GACTTAGAGG GGTGCTACGG TCCTTACCGT ACCTTCCTGA TAAGTAACCT GACCCCCGAA CCCGATCCGG   
  
  
+ TTTTTCACAG ACCATCTTTT CCAAAATAAG GAGTCACACT TAGGGTTTTT CTTTCTTATT TTGTTTACCC   
  
  
+ TTTAAAAATA AAACAAAAAT AAGTGGCGAC TCCAAGTCAT TTTTCTTAAT CAATAAAGAT CATTTTTTCG   
  
  
+ AAAACGAAAA TCGAGCTCGC CATCGAGTGG GAAACGCATT GAGCCGAAAT GCGGGGTCCA TAGGTATGTT   
  
  
+ TTTTATTTAT GAGATAAAAA GTAAATAATA TTTTTTTCGG GTTTAAAAAA TATATTATTG ATTACTTGTA   
  
  
+ ATTATATGAG CCTCGTTTGT ACTTATTATT AAATACATAT TCAAGGTAAA ATTATCCTTT AAAAAATTCT   
  
  
+ TAGGCCGAGG GAACCTTCCT ATTAATATAA GTCATCCAGC CCACCTTATG GGGGTAATTC TCCCCATATA   
  
  
+ CCATGGGTCC AAATCGGCGG CCCGGTCAGT TCAACGACAG TACGATGGCA CTCTGAACCC TGTGCTGCAT   
  
  
+ CTGCTCACAA AAAGTACACC ACAGTTGTCT TTCACTCTCT CTGCCCATTC ACTATGCAAA CACGTTTATA   
  
  
+ AAATTGGTTA TTTTATAAAC GTTTCTCTTT CAAATGGTTA TTTTATATTA GTAACAAAAA TATTATAATA   
  
  
+ ATAAAAAAAT CAATATTACG TAATTGCTAA AAATGAAATT ACATCATGTT GTAAGATATT TCATTTGCCT   
  
  
+ ATTAATAATT TTTTATTATT TATAAAATTG GGTTGAATTT CAATATTTTT TAAAATAAGT CTTATTTATT   
  
  
+ TAAGTGAAAT TTTTTTTATA GATAAAGTCA CAAAAATCAT TTTTCTAAAT AATTTATAAA AAGATACTTT   
  
  
+ TCCAATAAAA ATTAAATTAA AATGCTTTTA TAAATAAAAT TGTAACAATT CATTCCATAG CAAAGGTAAA   
  
  
+ TAAAGCCATT TTCTTATCAA TTAAATCAAG CTTCTCTTTT TTATGAAATT GTTTTTAATA AATTCTTTTA   
  
  
+ GTAACAATAA GATTTTGTTC ATATTTTAAT ACATCTCCTT GGCAATTGGG CAAAGATGGC TGGTAGTGAC   
  
  
+ ATGAATCCAA GCATGAATTA GTCCGGGCGT TGTTACTTGC TACAAGGGTT ATGAGCATGG AAGGAAAAAT   
  
  
+ AATTAAAAAA TAAAAAAAAT TAAATTACTT CAATCACAAA GTTGAAAATA ATAAAATTGT GTTTGGAAGT   
  
  
+ ATTTGTACTT AAAGTTTTTT AGTAAAAGTG TTTCCCTAAA AGGTGTTTTT TTTAGAGAAA GCAGCTATAA   
  
  
+ AATGTTTTTT CAAAAATATT TTTTTATTTA AAAACACTTT CTTAAATCAC TTGTTATAAG TCACCTTAGA   
  
  
+ ATGACTGACG GTGTTGCACA TATCAAAAAG CATTTATGCC CTAGTGCCAT TTTATTATTT TATATCTTAA   
  
  
+ TTTTAGATAT TTTTATTTTT ATTTAATGAT TTATATTTGA ACGTTAATTT TCAATATATC ATATTGTATC   
  
  
+ TTTTATAGCC ACGTTTATTC AAATATATTT TTTATATTTA TAAATATTTT TTCTAAAATA TGATTCGAGA   
  
  
+ ATGCTGACAT GTGAATCGCA TGGGGGTAGG GCAAGCCATC TTGGACCCAT TAAACTTCAA ATTCCATGAT   
  
  
+ TGACTAGGAC TTGCCCAAAA ATTTCATTAC CCCAGATGCC CACATGTGAA TGCAGTCTCT GACAGAGTAG   
  
  
+ GTGGGAATAA ACCAAAATGC ATTTATTTAT TTTTCAAGAT TTTAATTATT ATTCACCTAA AATCCTTCAT   
  
  
+ AGGGATTAAA ATATTAGACC ATTGGATTTG CTCTCCTCAA TAGGGTTGAA CTTTGATTAG TACGTTTATA   
  
  
+ TTATTTTTTT CCTTTTTCGT CAAAATGCAT TCTGTCACAA AATTAATGTA GTCAAGGAAT TGACAAGTGA   
  
  
+ GAAGAGAGTC ACTTTAAATG ATTCAGCTGT CCACATTAAA GATAAGTCAC ATTTGTGGTC CATACTCCCC   
  
  
+ AAGACTTTAA TTTGAATTTA GCTCAGACTT TTCGTATTTT ATTTGTGTTT TTCTATTTAA GCTATCAATA   
  
  
+ CATTTATAAA TAAGTGATTT TAATATAAAA GATCAAGTAA AAAACAAATA AAGTACATAA AATATTTTTA   
  
  
+ TTCAATTACA TATTGTATCA AATTTTCAAG AGCAAAATTT ATAGCATTTT TCAATGTTTT GGAAATCAAA   
  
  
+ AAAGTAATAT ATTATCAGTT CACTAGTTAA ATTGGTGATT GAACCATAGA TGATGTCATA ATATATATTT   
  
  
+ TATATTTATT AAAATTAAAA ATATTTATAA GAAATTTTAA ATATATGAGA AATTAAAAGT CAATAATATT   
  
  
+ TTTTTCTTCA TCTTTAATAT TATCAAATGA ATATATATAT ATATATAAAT GTATTGATAT TTTAAAATTT   
  
  
+ TGTTAAATAA AATATATATA ATATGAAGAT ATTTATTGAA AGTTATTTAA TTTACCTATA TATATATTAA   
  
  
+ AAGTTATTTA TGGTTGTAAA AAATATTACA TTATTTTATT TATATTATTA ATATTACATT ATTTTATTTA   
  
  
+ TATTATTTAT AATTTCATTA TTATAAAAAG TTTGAAAATA TTATTGCTAT ATAATTTGTT GTTGTGTGAA   
  
  
+ GATCGGCAAA AAAACAGGAG TAGCAGTGTG GTGTCGTCCT TGCCCCTTGA ACTGGAGGTT GGTTTCCCAT   
  
  
+ TCCTCCCTCC CACGAATCCC ACATGTAAAG CAAATAAAAG AGCTTTACAT ACACATGATT AATGTATTTT   
  
  
+ TATTGTCAAT AAATTTTATA ATAAAACTTA TAAATCTTAT AAATTTAATA GGAAAAAAAA AAGTCTTCTA   
  
  
+ GAATTGATTT TATAATACAC CATACCAAGA TCACATGAAT TGCATGTGTC GGTGGACAAG GTTGCCAAAA   
  
  
+ TTCCTTATTC CTATACAGAA TCTTTTCATT GTTATATTAT CATAGTCGAA ACCCAGTAGT ATATAAAGAG   
  
  
+ GTTACGAGTG GAGGATGGAT ATGAAATAAA TAAGAGGCCT TAGAGATCAG GGTGTGAGAC ATGGATTTGG   
  
  
+ CAAATGGTGA GAGATCTAGT GAGC  

- TTAGTAATTT CAGATTAAGA AGTTTTAAGG CTTAAGATTT AAATCGATAA ATTTTAAGGT TAACAGGATA   
  
  
- GAGGCTTGAA TTAAAAGTCA AAGGGTACGA GGTTAAGGGC ATAAATGGGG CGATGAATAA TAATAGTAAT   
  
  
- AGTAATAGTG GAATAAGTAA TAAAGATATT TAAGGCGGAA GCTTACTAAA GATTCTAGAG GTTAAAAATA   
  
  
- TGTAGTTGAA AGTATTAAAG ATGCAGAGTT TATTGTATGC TAAGACTAAG GAAAGTTTTA AATTGAGGGT   
  
  
- ATTAAGGTTA AAAGCATTGA ATTAAAAAGA ATTTTTAGGG CTTGAAAGTT TTAGGGTTAA AAGAAAGTTT   
  
  
- AAATTAAAGA TTTACAAGAG TAAGAATGTA GATTAAGGAT TCTTAGGTTA AAAGTTTATT AGAGATTCTA   
  
  
- AGGCTAAGAG TTTACTTAAA GTTTTTAGGT CAAAGGGAAG CTTGTTAAAA TTAGGGACAA ATCACTACGT   
  
  
- ACATTATACA AAACACGAGA TATACCACAT GATTGGGAGA GATAACTATC GCGTACCACC AAGCACCGGG   
  
  
- TCCATACATG GGTAAAAGAG AGATTAGCAA CAGATACGTA CAGAGCTAAG AATACACACG TGCTAGTAAG   
  
  
- TCCTATAAGT AACTAAATGA GTAGTTAACA GTGCCGTCGA AGTAAAATAA TCATCTCTGG GCTGAAATCC   
  
  
- CTGAATCTCC CCACGATGCC AGGAATGGCA TGGAAGGACT ATTCATTGGA CTGGGGGCTT GGGCTAGGCC   
  
  
- AAAAAGTGTC TGGTAGAAAA GGTTTTATTC CTCAGTGTGA ATCCCAAAAA GAAAGAATAA AACAAATGGG   
  
  
- AAATTTTTAT TTTGTTTTTA TTCACCGCTG AGGTTCAGTA AAAAGAATTA GTTATTTCTA GTAAAAAAGC   
  
  
- TTTTGCTTTT AGCTCGAGCG GTAGCTCACC CTTTGCGTAA CTCGGCTTTA CGCCCCAGGT ATCCATACAA   
  
  
- AAAATAAATA CTCTATTTTT CATTTATTAT AAAAAAAGCC CAAATTTTTT ATATAATAAC TAATGAACAT   
  
  
- TAATATACTC GGAGCAAACA TGAATAATAA TTTATGTATA AGTTCCATTT TAATAGGAAA TTTTTTAAGA   
  
  
- ATCCGGCTCC CTTGGAAGGA TAATTATATT CAGTAGGTCG GGTGGAATAC CCCCATTAAG AGGGGTATAT   
  
  
- GGTACCCAGG TTTAGCCGCC GGGCCAGTCA AGTTGCTGTC ATGCTACCGT GAGACTTGGG ACACGACGTA   
  
  
- GACGAGTGTT TTTCATGTGG TGTCAACAGA AAGTGAGAGA GACGGGTAAG TGATACGTTT GTGCAAATAT   
  
  
- TTTAACCAAT AAAATATTTG CAAAGAGAAA GTTTACCAAT AAAATATAAT CATTGTTTTT ATAATATTAT   
  
  
- TATTTTTTTA GTTATAATGC ATTAACGATT TTTACTTTAA TGTAGTACAA CATTCTATAA AGTAAACGGA   
  
  
- TAATTATTAA AAAATAATAA ATATTTTAAC CCAACTTAAA GTTATAAAAA ATTTTATTCA GAATAAATAA   
  
  
- ATTCACTTTA AAAAAAATAT CTATTTCAGT GTTTTTAGTA AAAAGATTTA TTAAATATTT TTCTATGAAA   
  
  
- AGGTTATTTT TAATTTAATT TTACGAAAAT ATTTATTTTA ACATTGTTAA GTAAGGTATC GTTTCCATTT   
  
  
- ATTTCGGTAA AAGAATAGTT AATTTAGTTC GAAGAGAAAA AATACTTTAA CAAAAATTAT TTAAGAAAAT   
  
  
- CATTGTTATT CTAAAACAAG TATAAAATTA TGTAGAGGAA CCGTTAACCC GTTTCTACCG ACCATCACTG   
  
  
- TACTTAGGTT CGTACTTAAT CAGGCCCGCA ACAATGAACG ATGTTCCCAA TACTCGTACC TTCCTTTTTA   
  
  
- TTAATTTTTT ATTTTTTTTA ATTTAATGAA GTTAGTGTTT CAACTTTTAT TATTTTAACA CAAACCTTCA   
  
  
- TAAACATGAA TTTCAAAAAA TCATTTTCAC AAAGGGATTT TCCACAAAAA AAATCTCTTT CGTCGATATT   
  
  
- TTACAAAAAA GTTTTTATAA AAAAATAAAT TTTTGTGAAA GAATTTAGTG AACAATATTC AGTGGAATCT   
  
  
- TACTGACTGC CACAACGTGT ATAGTTTTTC GTAAATACGG GATCACGGTA AAATAATAAA ATATAGAATT   
  
  
- AAAATCTATA AAAATAAAAA TAAATTACTA AATATAAACT TGCAATTAAA AGTTATATAG TATAACATAG   
  
  
- AAAATATCGG TGCAAATAAG TTTATATAAA AAATATAAAT ATTTATAAAA AAGATTTTAT ACTAAGCTCT   
  
  
- TACGACTGTA CACTTAGCGT ACCCCCATCC CGTTCGGTAG AACCTGGGTA ATTTGAAGTT TAAGGTACTA   
  
  
- ACTGATCCTG AACGGGTTTT TAAAGTAATG GGGTCTACGG GTGTACACTT ACGTCAGAGA CTGTCTCATC   
  
  
- CACCCTTATT TGGTTTTACG TAAATAAATA AAAAGTTCTA AAATTAATAA TAAGTGGATT TTAGGAAGTA   
  
  
- TCCCTAATTT TATAATCTGG TAACCTAAAC GAGAGGAGTT ATCCCAACTT GAAACTAATC ATGCAAATAT   
  
  
- AATAAAAAAA GGAAAAAGCA GTTTTACGTA AGACAGTGTT TTAATTACAT CAGTTCCTTA ACTGTTCACT   
  
  
- CTTCTCTCAG TGAAATTTAC TAAGTCGACA GGTGTAATTT CTATTCAGTG TAAACACCAG GTATGAGGGG   
  
  
- TTCTGAAATT AAACTTAAAT CGAGTCTGAA AAGCATAAAA TAAACACAAA AAGATAAATT CGATAGTTAT   
  
  
- GTAAATATTT ATTCACTAAA ATTATATTTT CTAGTTCATT TTTTGTTTAT TTCATGTATT TTATAAAAAT   
  
  
- AAGTTAATGT ATAACATAGT TTAAAAGTTC TCGTTTTAAA TATCGTAAAA AGTTACAAAA CCTTTAGTTT   
  
  
- TTTCATTATA TAATAGTCAA GTGATCAATT TAACCACTAA CTTGGTATCT ACTACAGTAT TATATATAAA   
  
  
- ATATAAATAA TTTTAATTTT TATAAATATT CTTTAAAATT TATATACTCT TTAATTTTCA GTTATTATAA   
  
  
- AAAAAGAAGT AGAAATTATA ATAGTTTACT TATATATATA TATATATTTA CATAACTATA AAATTTTAAA   
  
  
- ACAATTTATT TTATATATAT TATACTTCTA TAAATAACTT TCAATAAATT AAATGGATAT ATATATAATT   
  
  
- TTCAATAAAT ACCAACATTT TTTATAATGT AATAAAATAA ATATAATAAT TATAATGTAA TAAAATAAAT   
  
  
- ATAATAAATA TTAAAGTAAT AATATTTTTC AAACTTTTAT AATAACGATA TATTAAACAA CAACACACTT   
  
  
- CTAGCCGTTT TTTTGTCCTC ATCGTCACAC CACAGCAGGA ACGGGGAACT TGACCTCCAA CCAAAGGGTA   
  
  
- AGGAGGGAGG GTGCTTAGGG TGTACATTTC GTTTATTTTC TCGAAATGTA TGTGTACTAA TTACATAAAA   
  
  
- ATAACAGTTA TTTAAAATAT TATTTTGAAT ATTTAGAATA TTTAAATTAT CCTTTTTTTT TTCAGAAGAT   
  
  
- CTTAACTAAA ATATTATGTG GTATGGTTCT AGTGTACTTA ACGTACACAG CCACCTGTTC CAACGGTTTT   
  
  
- AAGGAATAAG GATATGTCTT AGAAAAGTAA CAATATAATA GTATCAGCTT TGGGTCATCA TATATTTCTC   
  
  
- CAATGCTCAC CTCCTACCTA TACTTTATTT ATTCTCCGGA ATCTCTAGTC CCACACTCTG TACCTAAACC   
  
  
- GTTTACCACT CTCTAGATCA CTCG

+     CAT-box

| Site Name | Organism | Position | Strand | Matrix score. | sequence | function |
| --- | --- | --- | --- | --- | --- | --- |
| CAT-box | Arabidopsis thaliana | 862 | - | 6 | GCCACT | cis-acting regulatory element related to meristem expression |

>PlantCARE\_32120   
+ AATCATTAAA GTCTAATTCT TCAAAATTCC GAATTCTAAA TTTAGCTATT TAAAATTCCA ATTGTCCTAT   
  
  
+ CTCCGAACTT AATTTTCAGT TTCCCATGCT CCAATTCCCG TATTTACCCC GCTACTTATT ATTATCATTA   
  
  
+ TCATTATCAC CTTATTCATT ATTTCTATAA ATTCCGCCTT CGAATGATTT CTAAGATCTC CAATTTTTAT   
  
  
+ ACATCAACTT TCATAATTTC TACGTCTCAA ATAACATACG ATTCTGATTC CTTTCAAAAT TTAACTCCCA   
  
  
+ TAATTCCAAT TTTCGTAACT TAATTTTTCT TAAAAATCCC GAACTTTCAA AATCCCAATT TTCTTTCAAA   
  
  
+ TTTAATTTCT AAATGTTCTC ATTCTTACAT CTAATTCCTA AGAATCCAAT TTTCAAATAA TCTCTAAGAT   
  
  
+ TCCGATTCTC AAATGAATTT CAAAAATCCA GTTTCCCTTC GAACAATTTT AATCCCTGTT TAGTGATGCA   
  
  
+ TGTAATATGT TTTGTGCTCT ATATGGTGTA CTAACCCTCT CTATTGATAG CGCATGGTGG TTCGTGGCCC   
  
  
+ AGGTATGTAC CCATTTTCTC TCTAATCGTT GTCTATGCAT GTCTCGATTC TTATGTGTGC ACGATCATTC   
  
  
+ AGGATATTCA TTGATTTACT CATCAATTGT CACGGCAGCT TCATTTTATT AGTAGAGACC CGACTTTAGG   
  
  
+ GACTTAGAGG GGTGCTACGG TCCTTACCGT ACCTTCCTGA TAAGTAACCT GACCCCCGAA CCCGATCCGG   
  
  
+ TTTTTCACAG ACCATCTTTT CCAAAATAAG GAGTCACACT TAGGGTTTTT CTTTCTTATT TTGTTTACCC   
  
  
+ TTTAAAAATA AAACAAAAAT AAGTGGCGAC TCCAAGTCAT TTTTCTTAAT CAATAAAGAT CATTTTTTCG   
  
  
+ AAAACGAAAA TCGAGCTCGC CATCGAGTGG GAAACGCATT GAGCCGAAAT GCGGGGTCCA TAGGTATGTT   
  
  
+ TTTTATTTAT GAGATAAAAA GTAAATAATA TTTTTTTCGG GTTTAAAAAA TATATTATTG ATTACTTGTA   
  
  
+ ATTATATGAG CCTCGTTTGT ACTTATTATT AAATACATAT TCAAGGTAAA ATTATCCTTT AAAAAATTCT   
  
  
+ TAGGCCGAGG GAACCTTCCT ATTAATATAA GTCATCCAGC CCACCTTATG GGGGTAATTC TCCCCATATA   
  
  
+ CCATGGGTCC AAATCGGCGG CCCGGTCAGT TCAACGACAG TACGATGGCA CTCTGAACCC TGTGCTGCAT   
  
  
+ CTGCTCACAA AAAGTACACC ACAGTTGTCT TTCACTCTCT CTGCCCATTC ACTATGCAAA CACGTTTATA   
  
  
+ AAATTGGTTA TTTTATAAAC GTTTCTCTTT CAAATGGTTA TTTTATATTA GTAACAAAAA TATTATAATA   
  
  
+ ATAAAAAAAT CAATATTACG TAATTGCTAA AAATGAAATT ACATCATGTT GTAAGATATT TCATTTGCCT   
  
  
+ ATTAATAATT TTTTATTATT TATAAAATTG GGTTGAATTT CAATATTTTT TAAAATAAGT CTTATTTATT   
  
  
+ TAAGTGAAAT TTTTTTTATA GATAAAGTCA CAAAAATCAT TTTTCTAAAT AATTTATAAA AAGATACTTT   
  
  
+ TCCAATAAAA ATTAAATTAA AATGCTTTTA TAAATAAAAT TGTAACAATT CATTCCATAG CAAAGGTAAA   
  
  
+ TAAAGCCATT TTCTTATCAA TTAAATCAAG CTTCTCTTTT TTATGAAATT GTTTTTAATA AATTCTTTTA   
  
  
+ GTAACAATAA GATTTTGTTC ATATTTTAAT ACATCTCCTT GGCAATTGGG CAAAGATGGC TGGTAGTGAC   
  
  
+ ATGAATCCAA GCATGAATTA GTCCGGGCGT TGTTACTTGC TACAAGGGTT ATGAGCATGG AAGGAAAAAT   
  
  
+ AATTAAAAAA TAAAAAAAAT TAAATTACTT CAATCACAAA GTTGAAAATA ATAAAATTGT GTTTGGAAGT   
  
  
+ ATTTGTACTT AAAGTTTTTT AGTAAAAGTG TTTCCCTAAA AGGTGTTTTT TTTAGAGAAA GCAGCTATAA   
  
  
+ AATGTTTTTT CAAAAATATT TTTTTATTTA AAAACACTTT CTTAAATCAC TTGTTATAAG TCACCTTAGA   
  
  
+ ATGACTGACG GTGTTGCACA TATCAAAAAG CATTTATGCC CTAGTGCCAT TTTATTATTT TATATCTTAA   
  
  
+ TTTTAGATAT TTTTATTTTT ATTTAATGAT TTATATTTGA ACGTTAATTT TCAATATATC ATATTGTATC   
  
  
+ TTTTATAGCC ACGTTTATTC AAATATATTT TTTATATTTA TAAATATTTT TTCTAAAATA TGATTCGAGA   
  
  
+ ATGCTGACAT GTGAATCGCA TGGGGGTAGG GCAAGCCATC TTGGACCCAT TAAACTTCAA ATTCCATGAT   
  
  
+ TGACTAGGAC TTGCCCAAAA ATTTCATTAC CCCAGATGCC CACATGTGAA TGCAGTCTCT GACAGAGTAG   
  
  
+ GTGGGAATAA ACCAAAATGC ATTTATTTAT TTTTCAAGAT TTTAATTATT ATTCACCTAA AATCCTTCAT   
  
  
+ AGGGATTAAA ATATTAGACC ATTGGATTTG CTCTCCTCAA TAGGGTTGAA CTTTGATTAG TACGTTTATA   
  
  
+ TTATTTTTTT CCTTTTTCGT CAAAATGCAT TCTGTCACAA AATTAATGTA GTCAAGGAAT TGACAAGTGA   
  
  
+ GAAGAGAGTC ACTTTAAATG ATTCAGCTGT CCACATTAAA GATAAGTCAC ATTTGTGGTC CATACTCCCC   
  
  
+ AAGACTTTAA TTTGAATTTA GCTCAGACTT TTCGTATTTT ATTTGTGTTT TTCTATTTAA GCTATCAATA   
  
  
+ CATTTATAAA TAAGTGATTT TAATATAAAA GATCAAGTAA AAAACAAATA AAGTACATAA AATATTTTTA   
  
  
+ TTCAATTACA TATTGTATCA AATTTTCAAG AGCAAAATTT ATAGCATTTT TCAATGTTTT GGAAATCAAA   
  
  
+ AAAGTAATAT ATTATCAGTT CACTAGTTAA ATTGGTGATT GAACCATAGA TGATGTCATA ATATATATTT   
  
  
+ TATATTTATT AAAATTAAAA ATATTTATAA GAAATTTTAA ATATATGAGA AATTAAAAGT CAATAATATT   
  
  
+ TTTTTCTTCA TCTTTAATAT TATCAAATGA ATATATATAT ATATATAAAT GTATTGATAT TTTAAAATTT   
  
  
+ TGTTAAATAA AATATATATA ATATGAAGAT ATTTATTGAA AGTTATTTAA TTTACCTATA TATATATTAA   
  
  
+ AAGTTATTTA TGGTTGTAAA AAATATTACA TTATTTTATT TATATTATTA ATATTACATT ATTTTATTTA   
  
  
+ TATTATTTAT AATTTCATTA TTATAAAAAG TTTGAAAATA TTATTGCTAT ATAATTTGTT GTTGTGTGAA   
  
  
+ GATCGGCAAA AAAACAGGAG TAGCAGTGTG GTGTCGTCCT TGCCCCTTGA ACTGGAGGTT GGTTTCCCAT   
  
  
+ TCCTCCCTCC CACGAATCCC ACATGTAAAG CAAATAAAAG AGCTTTACAT ACACATGATT AATGTATTTT   
  
  
+ TATTGTCAAT AAATTTTATA ATAAAACTTA TAAATCTTAT AAATTTAATA GGAAAAAAAA AAGTCTTCTA   
  
  
+ GAATTGATTT TATAATACAC CATACCAAGA TCACATGAAT TGCATGTGTC GGTGGACAAG GTTGCCAAAA   
  
  
+ TTCCTTATTC CTATACAGAA TCTTTTCATT GTTATATTAT CATAGTCGAA ACCCAGTAGT ATATAAAGAG   
  
  
+ GTTACGAGTG GAGGATGGAT ATGAAATAAA TAAGAGGCCT TAGAGATCAG GGTGTGAGAC ATGGATTTGG   
  
  
+ CAAATGGTGA GAGATCTAGT GAGC  

- TTAGTAATTT CAGATTAAGA AGTTTTAAGG CTTAAGATTT AAATCGATAA ATTTTAAGGT TAACAGGATA   
  
  
- GAGGCTTGAA TTAAAAGTCA AAGGGTACGA GGTTAAGGGC ATAAATGGGG CGATGAATAA TAATAGTAAT   
  
  
- AGTAATAGTG GAATAAGTAA TAAAGATATT TAAGGCGGAA GCTTACTAAA GATTCTAGAG GTTAAAAATA   
  
  
- TGTAGTTGAA AGTATTAAAG ATGCAGAGTT TATTGTATGC TAAGACTAAG GAAAGTTTTA AATTGAGGGT   
  
  
- ATTAAGGTTA AAAGCATTGA ATTAAAAAGA ATTTTTAGGG CTTGAAAGTT TTAGGGTTAA AAGAAAGTTT   
  
  
- AAATTAAAGA TTTACAAGAG TAAGAATGTA GATTAAGGAT TCTTAGGTTA AAAGTTTATT AGAGATTCTA   
  
  
- AGGCTAAGAG TTTACTTAAA GTTTTTAGGT CAAAGGGAAG CTTGTTAAAA TTAGGGACAA ATCACTACGT   
  
  
- ACATTATACA AAACACGAGA TATACCACAT GATTGGGAGA GATAACTATC GCGTACCACC AAGCACCGGG   
  
  
- TCCATACATG GGTAAAAGAG AGATTAGCAA CAGATACGTA CAGAGCTAAG AATACACACG TGCTAGTAAG   
  
  
- TCCTATAAGT AACTAAATGA GTAGTTAACA GTGCCGTCGA AGTAAAATAA TCATCTCTGG GCTGAAATCC   
  
  
- CTGAATCTCC CCACGATGCC AGGAATGGCA TGGAAGGACT ATTCATTGGA CTGGGGGCTT GGGCTAGGCC   
  
  
- AAAAAGTGTC TGGTAGAAAA GGTTTTATTC CTCAGTGTGA ATCCCAAAAA GAAAGAATAA AACAAATGGG   
  
  
- AAATTTTTAT TTTGTTTTTA TTCACCGCTG AGGTTCAGTA AAAAGAATTA GTTATTTCTA GTAAAAAAGC   
  
  
- TTTTGCTTTT AGCTCGAGCG GTAGCTCACC CTTTGCGTAA CTCGGCTTTA CGCCCCAGGT ATCCATACAA   
  
  
- AAAATAAATA CTCTATTTTT CATTTATTAT AAAAAAAGCC CAAATTTTTT ATATAATAAC TAATGAACAT   
  
  
- TAATATACTC GGAGCAAACA TGAATAATAA TTTATGTATA AGTTCCATTT TAATAGGAAA TTTTTTAAGA   
  
  
- ATCCGGCTCC CTTGGAAGGA TAATTATATT CAGTAGGTCG GGTGGAATAC CCCCATTAAG AGGGGTATAT   
  
  
- GGTACCCAGG TTTAGCCGCC GGGCCAGTCA AGTTGCTGTC ATGCTACCGT GAGACTTGGG ACACGACGTA   
  
  
- GACGAGTGTT TTTCATGTGG TGTCAACAGA AAGTGAGAGA GACGGGTAAG TGATACGTTT GTGCAAATAT   
  
  
- TTTAACCAAT AAAATATTTG CAAAGAGAAA GTTTACCAAT AAAATATAAT CATTGTTTTT ATAATATTAT   
  
  
- TATTTTTTTA GTTATAATGC ATTAACGATT TTTACTTTAA TGTAGTACAA CATTCTATAA AGTAAACGGA   
  
  
- TAATTATTAA AAAATAATAA ATATTTTAAC CCAACTTAAA GTTATAAAAA ATTTTATTCA GAATAAATAA   
  
  
- ATTCACTTTA AAAAAAATAT CTATTTCAGT GTTTTTAGTA AAAAGATTTA TTAAATATTT TTCTATGAAA   
  
  
- AGGTTATTTT TAATTTAATT TTACGAAAAT ATTTATTTTA ACATTGTTAA GTAAGGTATC GTTTCCATTT   
  
  
- ATTTCGGTAA AAGAATAGTT AATTTAGTTC GAAGAGAAAA AATACTTTAA CAAAAATTAT TTAAGAAAAT   
  
  
- CATTGTTATT CTAAAACAAG TATAAAATTA TGTAGAGGAA CCGTTAACCC GTTTCTACCG ACCATCACTG   
  
  
- TACTTAGGTT CGTACTTAAT CAGGCCCGCA ACAATGAACG ATGTTCCCAA TACTCGTACC TTCCTTTTTA   
  
  
- TTAATTTTTT ATTTTTTTTA ATTTAATGAA GTTAGTGTTT CAACTTTTAT TATTTTAACA CAAACCTTCA   
  
  
- TAAACATGAA TTTCAAAAAA TCATTTTCAC AAAGGGATTT TCCACAAAAA AAATCTCTTT CGTCGATATT   
  
  
- TTACAAAAAA GTTTTTATAA AAAAATAAAT TTTTGTGAAA GAATTTAGTG AACAATATTC AGTGGAATCT   
  
  
- TACTGACTGC CACAACGTGT ATAGTTTTTC GTAAATACGG GATCACGGTA AAATAATAAA ATATAGAATT   
  
  
- AAAATCTATA AAAATAAAAA TAAATTACTA AATATAAACT TGCAATTAAA AGTTATATAG TATAACATAG   
  
  
- AAAATATCGG TGCAAATAAG TTTATATAAA AAATATAAAT ATTTATAAAA AAGATTTTAT ACTAAGCTCT   
  
  
- TACGACTGTA CACTTAGCGT ACCCCCATCC CGTTCGGTAG AACCTGGGTA ATTTGAAGTT TAAGGTACTA   
  
  
- ACTGATCCTG AACGGGTTTT TAAAGTAATG GGGTCTACGG GTGTACACTT ACGTCAGAGA CTGTCTCATC   
  
  
- CACCCTTATT TGGTTTTACG TAAATAAATA AAAAGTTCTA AAATTAATAA TAAGTGGATT TTAGGAAGTA   
  
  
- TCCCTAATTT TATAATCTGG TAACCTAAAC GAGAGGAGTT ATCCCAACTT GAAACTAATC ATGCAAATAT   
  
  
- AATAAAAAAA GGAAAAAGCA GTTTTACGTA AGACAGTGTT TTAATTACAT CAGTTCCTTA ACTGTTCACT   
  
  
- CTTCTCTCAG TGAAATTTAC TAAGTCGACA GGTGTAATTT CTATTCAGTG TAAACACCAG GTATGAGGGG   
  
  
- TTCTGAAATT AAACTTAAAT CGAGTCTGAA AAGCATAAAA TAAACACAAA AAGATAAATT CGATAGTTAT   
  
  
- GTAAATATTT ATTCACTAAA ATTATATTTT CTAGTTCATT TTTTGTTTAT TTCATGTATT TTATAAAAAT   
  
  
- AAGTTAATGT ATAACATAGT TTAAAAGTTC TCGTTTTAAA TATCGTAAAA AGTTACAAAA CCTTTAGTTT   
  
  
- TTTCATTATA TAATAGTCAA GTGATCAATT TAACCACTAA CTTGGTATCT ACTACAGTAT TATATATAAA   
  
  
- ATATAAATAA TTTTAATTTT TATAAATATT CTTTAAAATT TATATACTCT TTAATTTTCA GTTATTATAA   
  
  
- AAAAAGAAGT AGAAATTATA ATAGTTTACT TATATATATA TATATATTTA CATAACTATA AAATTTTAAA   
  
  
- ACAATTTATT TTATATATAT TATACTTCTA TAAATAACTT TCAATAAATT AAATGGATAT ATATATAATT   
  
  
- TTCAATAAAT ACCAACATTT TTTATAATGT AATAAAATAA ATATAATAAT TATAATGTAA TAAAATAAAT   
  
  
- ATAATAAATA TTAAAGTAAT AATATTTTTC AAACTTTTAT AATAACGATA TATTAAACAA CAACACACTT   
  
  
- CTAGCCGTTT TTTTGTCCTC ATCGTCACAC CACAGCAGGA ACGGGGAACT TGACCTCCAA CCAAAGGGTA   
  
  
- AGGAGGGAGG GTGCTTAGGG TGTACATTTC GTTTATTTTC TCGAAATGTA TGTGTACTAA TTACATAAAA   
  
  
- ATAACAGTTA TTTAAAATAT TATTTTGAAT ATTTAGAATA TTTAAATTAT CCTTTTTTTT TTCAGAAGAT   
  
  
- CTTAACTAAA ATATTATGTG GTATGGTTCT AGTGTACTTA ACGTACACAG CCACCTGTTC CAACGGTTTT   
  
  
- AAGGAATAAG GATATGTCTT AGAAAAGTAA CAATATAATA GTATCAGCTT TGGGTCATCA TATATTTCTC   
  
  
- CAATGCTCAC CTCCTACCTA TACTTTATTT ATTCTCCGGA ATCTCTAGTC CCACACTCTG TACCTAAACC   
  
  
- GTTTACCACT CTCTAGATCA CTCG

+     CGTCA-motif

| Site Name | Organism | Position | Strand | Matrix score. | sequence | function |
| --- | --- | --- | --- | --- | --- | --- |
| CGTCA-motif | Hordeum vulgare | 2106 | - | 5 | CGTCA | cis-acting regulatory element involved in the MeJA-responsiveness |
| CGTCA-motif | Hordeum vulgare | 2608 | + | 5 | CGTCA | cis-acting regulatory element involved in the MeJA-responsiveness |

>PlantCARE\_32120   
+ AATCATTAAA GTCTAATTCT TCAAAATTCC GAATTCTAAA TTTAGCTATT TAAAATTCCA ATTGTCCTAT   
  
  
+ CTCCGAACTT AATTTTCAGT TTCCCATGCT CCAATTCCCG TATTTACCCC GCTACTTATT ATTATCATTA   
  
  
+ TCATTATCAC CTTATTCATT ATTTCTATAA ATTCCGCCTT CGAATGATTT CTAAGATCTC CAATTTTTAT   
  
  
+ ACATCAACTT TCATAATTTC TACGTCTCAA ATAACATACG ATTCTGATTC CTTTCAAAAT TTAACTCCCA   
  
  
+ TAATTCCAAT TTTCGTAACT TAATTTTTCT TAAAAATCCC GAACTTTCAA AATCCCAATT TTCTTTCAAA   
  
  
+ TTTAATTTCT AAATGTTCTC ATTCTTACAT CTAATTCCTA AGAATCCAAT TTTCAAATAA TCTCTAAGAT   
  
  
+ TCCGATTCTC AAATGAATTT CAAAAATCCA GTTTCCCTTC GAACAATTTT AATCCCTGTT TAGTGATGCA   
  
  
+ TGTAATATGT TTTGTGCTCT ATATGGTGTA CTAACCCTCT CTATTGATAG CGCATGGTGG TTCGTGGCCC   
  
  
+ AGGTATGTAC CCATTTTCTC TCTAATCGTT GTCTATGCAT GTCTCGATTC TTATGTGTGC ACGATCATTC   
  
  
+ AGGATATTCA TTGATTTACT CATCAATTGT CACGGCAGCT TCATTTTATT AGTAGAGACC CGACTTTAGG   
  
  
+ GACTTAGAGG GGTGCTACGG TCCTTACCGT ACCTTCCTGA TAAGTAACCT GACCCCCGAA CCCGATCCGG   
  
  
+ TTTTTCACAG ACCATCTTTT CCAAAATAAG GAGTCACACT TAGGGTTTTT CTTTCTTATT TTGTTTACCC   
  
  
+ TTTAAAAATA AAACAAAAAT AAGTGGCGAC TCCAAGTCAT TTTTCTTAAT CAATAAAGAT CATTTTTTCG   
  
  
+ AAAACGAAAA TCGAGCTCGC CATCGAGTGG GAAACGCATT GAGCCGAAAT GCGGGGTCCA TAGGTATGTT   
  
  
+ TTTTATTTAT GAGATAAAAA GTAAATAATA TTTTTTTCGG GTTTAAAAAA TATATTATTG ATTACTTGTA   
  
  
+ ATTATATGAG CCTCGTTTGT ACTTATTATT AAATACATAT TCAAGGTAAA ATTATCCTTT AAAAAATTCT   
  
  
+ TAGGCCGAGG GAACCTTCCT ATTAATATAA GTCATCCAGC CCACCTTATG GGGGTAATTC TCCCCATATA   
  
  
+ CCATGGGTCC AAATCGGCGG CCCGGTCAGT TCAACGACAG TACGATGGCA CTCTGAACCC TGTGCTGCAT   
  
  
+ CTGCTCACAA AAAGTACACC ACAGTTGTCT TTCACTCTCT CTGCCCATTC ACTATGCAAA CACGTTTATA   
  
  
+ AAATTGGTTA TTTTATAAAC GTTTCTCTTT CAAATGGTTA TTTTATATTA GTAACAAAAA TATTATAATA   
  
  
+ ATAAAAAAAT CAATATTACG TAATTGCTAA AAATGAAATT ACATCATGTT GTAAGATATT TCATTTGCCT   
  
  
+ ATTAATAATT TTTTATTATT TATAAAATTG GGTTGAATTT CAATATTTTT TAAAATAAGT CTTATTTATT   
  
  
+ TAAGTGAAAT TTTTTTTATA GATAAAGTCA CAAAAATCAT TTTTCTAAAT AATTTATAAA AAGATACTTT   
  
  
+ TCCAATAAAA ATTAAATTAA AATGCTTTTA TAAATAAAAT TGTAACAATT CATTCCATAG CAAAGGTAAA   
  
  
+ TAAAGCCATT TTCTTATCAA TTAAATCAAG CTTCTCTTTT TTATGAAATT GTTTTTAATA AATTCTTTTA   
  
  
+ GTAACAATAA GATTTTGTTC ATATTTTAAT ACATCTCCTT GGCAATTGGG CAAAGATGGC TGGTAGTGAC   
  
  
+ ATGAATCCAA GCATGAATTA GTCCGGGCGT TGTTACTTGC TACAAGGGTT ATGAGCATGG AAGGAAAAAT   
  
  
+ AATTAAAAAA TAAAAAAAAT TAAATTACTT CAATCACAAA GTTGAAAATA ATAAAATTGT GTTTGGAAGT   
  
  
+ ATTTGTACTT AAAGTTTTTT AGTAAAAGTG TTTCCCTAAA AGGTGTTTTT TTTAGAGAAA GCAGCTATAA   
  
  
+ AATGTTTTTT CAAAAATATT TTTTTATTTA AAAACACTTT CTTAAATCAC TTGTTATAAG TCACCTTAGA   
  
  
+ ATGACTGACG GTGTTGCACA TATCAAAAAG CATTTATGCC CTAGTGCCAT TTTATTATTT TATATCTTAA   
  
  
+ TTTTAGATAT TTTTATTTTT ATTTAATGAT TTATATTTGA ACGTTAATTT TCAATATATC ATATTGTATC   
  
  
+ TTTTATAGCC ACGTTTATTC AAATATATTT TTTATATTTA TAAATATTTT TTCTAAAATA TGATTCGAGA   
  
  
+ ATGCTGACAT GTGAATCGCA TGGGGGTAGG GCAAGCCATC TTGGACCCAT TAAACTTCAA ATTCCATGAT   
  
  
+ TGACTAGGAC TTGCCCAAAA ATTTCATTAC CCCAGATGCC CACATGTGAA TGCAGTCTCT GACAGAGTAG   
  
  
+ GTGGGAATAA ACCAAAATGC ATTTATTTAT TTTTCAAGAT TTTAATTATT ATTCACCTAA AATCCTTCAT   
  
  
+ AGGGATTAAA ATATTAGACC ATTGGATTTG CTCTCCTCAA TAGGGTTGAA CTTTGATTAG TACGTTTATA   
  
  
+ TTATTTTTTT CCTTTTTCGT CAAAATGCAT TCTGTCACAA AATTAATGTA GTCAAGGAAT TGACAAGTGA   
  
  
+ GAAGAGAGTC ACTTTAAATG ATTCAGCTGT CCACATTAAA GATAAGTCAC ATTTGTGGTC CATACTCCCC   
  
  
+ AAGACTTTAA TTTGAATTTA GCTCAGACTT TTCGTATTTT ATTTGTGTTT TTCTATTTAA GCTATCAATA   
  
  
+ CATTTATAAA TAAGTGATTT TAATATAAAA GATCAAGTAA AAAACAAATA AAGTACATAA AATATTTTTA   
  
  
+ TTCAATTACA TATTGTATCA AATTTTCAAG AGCAAAATTT ATAGCATTTT TCAATGTTTT GGAAATCAAA   
  
  
+ AAAGTAATAT ATTATCAGTT CACTAGTTAA ATTGGTGATT GAACCATAGA TGATGTCATA ATATATATTT   
  
  
+ TATATTTATT AAAATTAAAA ATATTTATAA GAAATTTTAA ATATATGAGA AATTAAAAGT CAATAATATT   
  
  
+ TTTTTCTTCA TCTTTAATAT TATCAAATGA ATATATATAT ATATATAAAT GTATTGATAT TTTAAAATTT   
  
  
+ TGTTAAATAA AATATATATA ATATGAAGAT ATTTATTGAA AGTTATTTAA TTTACCTATA TATATATTAA   
  
  
+ AAGTTATTTA TGGTTGTAAA AAATATTACA TTATTTTATT TATATTATTA ATATTACATT ATTTTATTTA   
  
  
+ TATTATTTAT AATTTCATTA TTATAAAAAG TTTGAAAATA TTATTGCTAT ATAATTTGTT GTTGTGTGAA   
  
  
+ GATCGGCAAA AAAACAGGAG TAGCAGTGTG GTGTCGTCCT TGCCCCTTGA ACTGGAGGTT GGTTTCCCAT   
  
  
+ TCCTCCCTCC CACGAATCCC ACATGTAAAG CAAATAAAAG AGCTTTACAT ACACATGATT AATGTATTTT   
  
  
+ TATTGTCAAT AAATTTTATA ATAAAACTTA TAAATCTTAT AAATTTAATA GGAAAAAAAA AAGTCTTCTA   
  
  
+ GAATTGATTT TATAATACAC CATACCAAGA TCACATGAAT TGCATGTGTC GGTGGACAAG GTTGCCAAAA   
  
  
+ TTCCTTATTC CTATACAGAA TCTTTTCATT GTTATATTAT CATAGTCGAA ACCCAGTAGT ATATAAAGAG   
  
  
+ GTTACGAGTG GAGGATGGAT ATGAAATAAA TAAGAGGCCT TAGAGATCAG GGTGTGAGAC ATGGATTTGG   
  
  
+ CAAATGGTGA GAGATCTAGT GAGC  

- TTAGTAATTT CAGATTAAGA AGTTTTAAGG CTTAAGATTT AAATCGATAA ATTTTAAGGT TAACAGGATA   
  
  
- GAGGCTTGAA TTAAAAGTCA AAGGGTACGA GGTTAAGGGC ATAAATGGGG CGATGAATAA TAATAGTAAT   
  
  
- AGTAATAGTG GAATAAGTAA TAAAGATATT TAAGGCGGAA GCTTACTAAA GATTCTAGAG GTTAAAAATA   
  
  
- TGTAGTTGAA AGTATTAAAG ATGCAGAGTT TATTGTATGC TAAGACTAAG GAAAGTTTTA AATTGAGGGT   
  
  
- ATTAAGGTTA AAAGCATTGA ATTAAAAAGA ATTTTTAGGG CTTGAAAGTT TTAGGGTTAA AAGAAAGTTT   
  
  
- AAATTAAAGA TTTACAAGAG TAAGAATGTA GATTAAGGAT TCTTAGGTTA AAAGTTTATT AGAGATTCTA   
  
  
- AGGCTAAGAG TTTACTTAAA GTTTTTAGGT CAAAGGGAAG CTTGTTAAAA TTAGGGACAA ATCACTACGT   
  
  
- ACATTATACA AAACACGAGA TATACCACAT GATTGGGAGA GATAACTATC GCGTACCACC AAGCACCGGG   
  
  
- TCCATACATG GGTAAAAGAG AGATTAGCAA CAGATACGTA CAGAGCTAAG AATACACACG TGCTAGTAAG   
  
  
- TCCTATAAGT AACTAAATGA GTAGTTAACA GTGCCGTCGA AGTAAAATAA TCATCTCTGG GCTGAAATCC   
  
  
- CTGAATCTCC CCACGATGCC AGGAATGGCA TGGAAGGACT ATTCATTGGA CTGGGGGCTT GGGCTAGGCC   
  
  
- AAAAAGTGTC TGGTAGAAAA GGTTTTATTC CTCAGTGTGA ATCCCAAAAA GAAAGAATAA AACAAATGGG   
  
  
- AAATTTTTAT TTTGTTTTTA TTCACCGCTG AGGTTCAGTA AAAAGAATTA GTTATTTCTA GTAAAAAAGC   
  
  
- TTTTGCTTTT AGCTCGAGCG GTAGCTCACC CTTTGCGTAA CTCGGCTTTA CGCCCCAGGT ATCCATACAA   
  
  
- AAAATAAATA CTCTATTTTT CATTTATTAT AAAAAAAGCC CAAATTTTTT ATATAATAAC TAATGAACAT   
  
  
- TAATATACTC GGAGCAAACA TGAATAATAA TTTATGTATA AGTTCCATTT TAATAGGAAA TTTTTTAAGA   
  
  
- ATCCGGCTCC CTTGGAAGGA TAATTATATT CAGTAGGTCG GGTGGAATAC CCCCATTAAG AGGGGTATAT   
  
  
- GGTACCCAGG TTTAGCCGCC GGGCCAGTCA AGTTGCTGTC ATGCTACCGT GAGACTTGGG ACACGACGTA   
  
  
- GACGAGTGTT TTTCATGTGG TGTCAACAGA AAGTGAGAGA GACGGGTAAG TGATACGTTT GTGCAAATAT   
  
  
- TTTAACCAAT AAAATATTTG CAAAGAGAAA GTTTACCAAT AAAATATAAT CATTGTTTTT ATAATATTAT   
  
  
- TATTTTTTTA GTTATAATGC ATTAACGATT TTTACTTTAA TGTAGTACAA CATTCTATAA AGTAAACGGA   
  
  
- TAATTATTAA AAAATAATAA ATATTTTAAC CCAACTTAAA GTTATAAAAA ATTTTATTCA GAATAAATAA   
  
  
- ATTCACTTTA AAAAAAATAT CTATTTCAGT GTTTTTAGTA AAAAGATTTA TTAAATATTT TTCTATGAAA   
  
  
- AGGTTATTTT TAATTTAATT TTACGAAAAT ATTTATTTTA ACATTGTTAA GTAAGGTATC GTTTCCATTT   
  
  
- ATTTCGGTAA AAGAATAGTT AATTTAGTTC GAAGAGAAAA AATACTTTAA CAAAAATTAT TTAAGAAAAT   
  
  
- CATTGTTATT CTAAAACAAG TATAAAATTA TGTAGAGGAA CCGTTAACCC GTTTCTACCG ACCATCACTG   
  
  
- TACTTAGGTT CGTACTTAAT CAGGCCCGCA ACAATGAACG ATGTTCCCAA TACTCGTACC TTCCTTTTTA   
  
  
- TTAATTTTTT ATTTTTTTTA ATTTAATGAA GTTAGTGTTT CAACTTTTAT TATTTTAACA CAAACCTTCA   
  
  
- TAAACATGAA TTTCAAAAAA TCATTTTCAC AAAGGGATTT TCCACAAAAA AAATCTCTTT CGTCGATATT   
  
  
- TTACAAAAAA GTTTTTATAA AAAAATAAAT TTTTGTGAAA GAATTTAGTG AACAATATTC AGTGGAATCT   
  
  
- TACTGACTGC CACAACGTGT ATAGTTTTTC GTAAATACGG GATCACGGTA AAATAATAAA ATATAGAATT   
  
  
- AAAATCTATA AAAATAAAAA TAAATTACTA AATATAAACT TGCAATTAAA AGTTATATAG TATAACATAG   
  
  
- AAAATATCGG TGCAAATAAG TTTATATAAA AAATATAAAT ATTTATAAAA AAGATTTTAT ACTAAGCTCT   
  
  
- TACGACTGTA CACTTAGCGT ACCCCCATCC CGTTCGGTAG AACCTGGGTA ATTTGAAGTT TAAGGTACTA   
  
  
- ACTGATCCTG AACGGGTTTT TAAAGTAATG GGGTCTACGG GTGTACACTT ACGTCAGAGA CTGTCTCATC   
  
  
- CACCCTTATT TGGTTTTACG TAAATAAATA AAAAGTTCTA AAATTAATAA TAAGTGGATT TTAGGAAGTA   
  
  
- TCCCTAATTT TATAATCTGG TAACCTAAAC GAGAGGAGTT ATCCCAACTT GAAACTAATC ATGCAAATAT   
  
  
- AATAAAAAAA GGAAAAAGCA GTTTTACGTA AGACAGTGTT TTAATTACAT CAGTTCCTTA ACTGTTCACT   
  
  
- CTTCTCTCAG TGAAATTTAC TAAGTCGACA GGTGTAATTT CTATTCAGTG TAAACACCAG GTATGAGGGG   
  
  
- TTCTGAAATT AAACTTAAAT CGAGTCTGAA AAGCATAAAA TAAACACAAA AAGATAAATT CGATAGTTAT   
  
  
- GTAAATATTT ATTCACTAAA ATTATATTTT CTAGTTCATT TTTTGTTTAT TTCATGTATT TTATAAAAAT   
  
  
- AAGTTAATGT ATAACATAGT TTAAAAGTTC TCGTTTTAAA TATCGTAAAA AGTTACAAAA CCTTTAGTTT   
  
  
- TTTCATTATA TAATAGTCAA GTGATCAATT TAACCACTAA CTTGGTATCT ACTACAGTAT TATATATAAA   
  
  
- ATATAAATAA TTTTAATTTT TATAAATATT CTTTAAAATT TATATACTCT TTAATTTTCA GTTATTATAA   
  
  
- AAAAAGAAGT AGAAATTATA ATAGTTTACT TATATATATA TATATATTTA CATAACTATA AAATTTTAAA   
  
  
- ACAATTTATT TTATATATAT TATACTTCTA TAAATAACTT TCAATAAATT AAATGGATAT ATATATAATT   
  
  
- TTCAATAAAT ACCAACATTT TTTATAATGT AATAAAATAA ATATAATAAT TATAATGTAA TAAAATAAAT   
  
  
- ATAATAAATA TTAAAGTAAT AATATTTTTC AAACTTTTAT AATAACGATA TATTAAACAA CAACACACTT   
  
  
- CTAGCCGTTT TTTTGTCCTC ATCGTCACAC CACAGCAGGA ACGGGGAACT TGACCTCCAA CCAAAGGGTA   
  
  
- AGGAGGGAGG GTGCTTAGGG TGTACATTTC GTTTATTTTC TCGAAATGTA TGTGTACTAA TTACATAAAA   
  
  
- ATAACAGTTA TTTAAAATAT TATTTTGAAT ATTTAGAATA TTTAAATTAT CCTTTTTTTT TTCAGAAGAT   
  
  
- CTTAACTAAA ATATTATGTG GTATGGTTCT AGTGTACTTA ACGTACACAG CCACCTGTTC CAACGGTTTT   
  
  
- AAGGAATAAG GATATGTCTT AGAAAAGTAA CAATATAATA GTATCAGCTT TGGGTCATCA TATATTTCTC   
  
  
- CAATGCTCAC CTCCTACCTA TACTTTATTT ATTCTCCGGA ATCTCTAGTC CCACACTCTG TACCTAAACC   
  
  
- GTTTACCACT CTCTAGATCA CTCG

+     ERE

| Site Name | Organism | Position | Strand | Matrix score. | sequence | function |
| --- | --- | --- | --- | --- | --- | --- |
| ERE | Nicotiana glutinos | 49 | - | 8 | ATTTTAAA |  |
| ERE | Nicotiana glutinos | 3139 | + | 8 | ATTTTAAA |  |
| ERE | Nicotiana glutinos | 1722 | - | 8 | ATTTCATA |  |
| ERE | Nicotiana glutinos | 3730 | - | 8 | ATTTCATA |  |
| ERE | Nicotiana glutinos | 1519 | - | 8 | ATTTTAAA |  |
| ERE | Nicotiana glutinos | 3141 | - | 8 | ATTTTAAA |  |
| ERE | Nicotiana glutinos | 3044 | + | 8 | ATTTTAAA |  |

>PlantCARE\_32120   
+ AATCATTAAA GTCTAATTCT TCAAAATTCC GAATTCTAAA TTTAGCTATT TAAAATTCCA ATTGTCCTAT   
  
  
+ CTCCGAACTT AATTTTCAGT TTCCCATGCT CCAATTCCCG TATTTACCCC GCTACTTATT ATTATCATTA   
  
  
+ TCATTATCAC CTTATTCATT ATTTCTATAA ATTCCGCCTT CGAATGATTT CTAAGATCTC CAATTTTTAT   
  
  
+ ACATCAACTT TCATAATTTC TACGTCTCAA ATAACATACG ATTCTGATTC CTTTCAAAAT TTAACTCCCA   
  
  
+ TAATTCCAAT TTTCGTAACT TAATTTTTCT TAAAAATCCC GAACTTTCAA AATCCCAATT TTCTTTCAAA   
  
  
+ TTTAATTTCT AAATGTTCTC ATTCTTACAT CTAATTCCTA AGAATCCAAT TTTCAAATAA TCTCTAAGAT   
  
  
+ TCCGATTCTC AAATGAATTT CAAAAATCCA GTTTCCCTTC GAACAATTTT AATCCCTGTT TAGTGATGCA   
  
  
+ TGTAATATGT TTTGTGCTCT ATATGGTGTA CTAACCCTCT CTATTGATAG CGCATGGTGG TTCGTGGCCC   
  
  
+ AGGTATGTAC CCATTTTCTC TCTAATCGTT GTCTATGCAT GTCTCGATTC TTATGTGTGC ACGATCATTC   
  
  
+ AGGATATTCA TTGATTTACT CATCAATTGT CACGGCAGCT TCATTTTATT AGTAGAGACC CGACTTTAGG   
  
  
+ GACTTAGAGG GGTGCTACGG TCCTTACCGT ACCTTCCTGA TAAGTAACCT GACCCCCGAA CCCGATCCGG   
  
  
+ TTTTTCACAG ACCATCTTTT CCAAAATAAG GAGTCACACT TAGGGTTTTT CTTTCTTATT TTGTTTACCC   
  
  
+ TTTAAAAATA AAACAAAAAT AAGTGGCGAC TCCAAGTCAT TTTTCTTAAT CAATAAAGAT CATTTTTTCG   
  
  
+ AAAACGAAAA TCGAGCTCGC CATCGAGTGG GAAACGCATT GAGCCGAAAT GCGGGGTCCA TAGGTATGTT   
  
  
+ TTTTATTTAT GAGATAAAAA GTAAATAATA TTTTTTTCGG GTTTAAAAAA TATATTATTG ATTACTTGTA   
  
  
+ ATTATATGAG CCTCGTTTGT ACTTATTATT AAATACATAT TCAAGGTAAA ATTATCCTTT AAAAAATTCT   
  
  
+ TAGGCCGAGG GAACCTTCCT ATTAATATAA GTCATCCAGC CCACCTTATG GGGGTAATTC TCCCCATATA   
  
  
+ CCATGGGTCC AAATCGGCGG CCCGGTCAGT TCAACGACAG TACGATGGCA CTCTGAACCC TGTGCTGCAT   
  
  
+ CTGCTCACAA AAAGTACACC ACAGTTGTCT TTCACTCTCT CTGCCCATTC ACTATGCAAA CACGTTTATA   
  
  
+ AAATTGGTTA TTTTATAAAC GTTTCTCTTT CAAATGGTTA TTTTATATTA GTAACAAAAA TATTATAATA   
  
  
+ ATAAAAAAAT CAATATTACG TAATTGCTAA AAATGAAATT ACATCATGTT GTAAGATATT TCATTTGCCT   
  
  
+ ATTAATAATT TTTTATTATT TATAAAATTG GGTTGAATTT CAATATTTTT TAAAATAAGT CTTATTTATT   
  
  
+ TAAGTGAAAT TTTTTTTATA GATAAAGTCA CAAAAATCAT TTTTCTAAAT AATTTATAAA AAGATACTTT   
  
  
+ TCCAATAAAA ATTAAATTAA AATGCTTTTA TAAATAAAAT TGTAACAATT CATTCCATAG CAAAGGTAAA   
  
  
+ TAAAGCCATT TTCTTATCAA TTAAATCAAG CTTCTCTTTT TTATGAAATT GTTTTTAATA AATTCTTTTA   
  
  
+ GTAACAATAA GATTTTGTTC ATATTTTAAT ACATCTCCTT GGCAATTGGG CAAAGATGGC TGGTAGTGAC   
  
  
+ ATGAATCCAA GCATGAATTA GTCCGGGCGT TGTTACTTGC TACAAGGGTT ATGAGCATGG AAGGAAAAAT   
  
  
+ AATTAAAAAA TAAAAAAAAT TAAATTACTT CAATCACAAA GTTGAAAATA ATAAAATTGT GTTTGGAAGT   
  
  
+ ATTTGTACTT AAAGTTTTTT AGTAAAAGTG TTTCCCTAAA AGGTGTTTTT TTTAGAGAAA GCAGCTATAA   
  
  
+ AATGTTTTTT CAAAAATATT TTTTTATTTA AAAACACTTT CTTAAATCAC TTGTTATAAG TCACCTTAGA   
  
  
+ ATGACTGACG GTGTTGCACA TATCAAAAAG CATTTATGCC CTAGTGCCAT TTTATTATTT TATATCTTAA   
  
  
+ TTTTAGATAT TTTTATTTTT ATTTAATGAT TTATATTTGA ACGTTAATTT TCAATATATC ATATTGTATC   
  
  
+ TTTTATAGCC ACGTTTATTC AAATATATTT TTTATATTTA TAAATATTTT TTCTAAAATA TGATTCGAGA   
  
  
+ ATGCTGACAT GTGAATCGCA TGGGGGTAGG GCAAGCCATC TTGGACCCAT TAAACTTCAA ATTCCATGAT   
  
  
+ TGACTAGGAC TTGCCCAAAA ATTTCATTAC CCCAGATGCC CACATGTGAA TGCAGTCTCT GACAGAGTAG   
  
  
+ GTGGGAATAA ACCAAAATGC ATTTATTTAT TTTTCAAGAT TTTAATTATT ATTCACCTAA AATCCTTCAT   
  
  
+ AGGGATTAAA ATATTAGACC ATTGGATTTG CTCTCCTCAA TAGGGTTGAA CTTTGATTAG TACGTTTATA   
  
  
+ TTATTTTTTT CCTTTTTCGT CAAAATGCAT TCTGTCACAA AATTAATGTA GTCAAGGAAT TGACAAGTGA   
  
  
+ GAAGAGAGTC ACTTTAAATG ATTCAGCTGT CCACATTAAA GATAAGTCAC ATTTGTGGTC CATACTCCCC   
  
  
+ AAGACTTTAA TTTGAATTTA GCTCAGACTT TTCGTATTTT ATTTGTGTTT TTCTATTTAA GCTATCAATA   
  
  
+ CATTTATAAA TAAGTGATTT TAATATAAAA GATCAAGTAA AAAACAAATA AAGTACATAA AATATTTTTA   
  
  
+ TTCAATTACA TATTGTATCA AATTTTCAAG AGCAAAATTT ATAGCATTTT TCAATGTTTT GGAAATCAAA   
  
  
+ AAAGTAATAT ATTATCAGTT CACTAGTTAA ATTGGTGATT GAACCATAGA TGATGTCATA ATATATATTT   
  
  
+ TATATTTATT AAAATTAAAA ATATTTATAA GAAATTTTAA ATATATGAGA AATTAAAAGT CAATAATATT   
  
  
+ TTTTTCTTCA TCTTTAATAT TATCAAATGA ATATATATAT ATATATAAAT GTATTGATAT TTTAAAATTT   
  
  
+ TGTTAAATAA AATATATATA ATATGAAGAT ATTTATTGAA AGTTATTTAA TTTACCTATA TATATATTAA   
  
  
+ AAGTTATTTA TGGTTGTAAA AAATATTACA TTATTTTATT TATATTATTA ATATTACATT ATTTTATTTA   
  
  
+ TATTATTTAT AATTTCATTA TTATAAAAAG TTTGAAAATA TTATTGCTAT ATAATTTGTT GTTGTGTGAA   
  
  
+ GATCGGCAAA AAAACAGGAG TAGCAGTGTG GTGTCGTCCT TGCCCCTTGA ACTGGAGGTT GGTTTCCCAT   
  
  
+ TCCTCCCTCC CACGAATCCC ACATGTAAAG CAAATAAAAG AGCTTTACAT ACACATGATT AATGTATTTT   
  
  
+ TATTGTCAAT AAATTTTATA ATAAAACTTA TAAATCTTAT AAATTTAATA GGAAAAAAAA AAGTCTTCTA   
  
  
+ GAATTGATTT TATAATACAC CATACCAAGA TCACATGAAT TGCATGTGTC GGTGGACAAG GTTGCCAAAA   
  
  
+ TTCCTTATTC CTATACAGAA TCTTTTCATT GTTATATTAT CATAGTCGAA ACCCAGTAGT ATATAAAGAG   
  
  
+ GTTACGAGTG GAGGATGGAT ATGAAATAAA TAAGAGGCCT TAGAGATCAG GGTGTGAGAC ATGGATTTGG   
  
  
+ CAAATGGTGA GAGATCTAGT GAGC  

- TTAGTAATTT CAGATTAAGA AGTTTTAAGG CTTAAGATTT AAATCGATAA ATTTTAAGGT TAACAGGATA   
  
  
- GAGGCTTGAA TTAAAAGTCA AAGGGTACGA GGTTAAGGGC ATAAATGGGG CGATGAATAA TAATAGTAAT   
  
  
- AGTAATAGTG GAATAAGTAA TAAAGATATT TAAGGCGGAA GCTTACTAAA GATTCTAGAG GTTAAAAATA   
  
  
- TGTAGTTGAA AGTATTAAAG ATGCAGAGTT TATTGTATGC TAAGACTAAG GAAAGTTTTA AATTGAGGGT   
  
  
- ATTAAGGTTA AAAGCATTGA ATTAAAAAGA ATTTTTAGGG CTTGAAAGTT TTAGGGTTAA AAGAAAGTTT   
  
  
- AAATTAAAGA TTTACAAGAG TAAGAATGTA GATTAAGGAT TCTTAGGTTA AAAGTTTATT AGAGATTCTA   
  
  
- AGGCTAAGAG TTTACTTAAA GTTTTTAGGT CAAAGGGAAG CTTGTTAAAA TTAGGGACAA ATCACTACGT   
  
  
- ACATTATACA AAACACGAGA TATACCACAT GATTGGGAGA GATAACTATC GCGTACCACC AAGCACCGGG   
  
  
- TCCATACATG GGTAAAAGAG AGATTAGCAA CAGATACGTA CAGAGCTAAG AATACACACG TGCTAGTAAG   
  
  
- TCCTATAAGT AACTAAATGA GTAGTTAACA GTGCCGTCGA AGTAAAATAA TCATCTCTGG GCTGAAATCC   
  
  
- CTGAATCTCC CCACGATGCC AGGAATGGCA TGGAAGGACT ATTCATTGGA CTGGGGGCTT GGGCTAGGCC   
  
  
- AAAAAGTGTC TGGTAGAAAA GGTTTTATTC CTCAGTGTGA ATCCCAAAAA GAAAGAATAA AACAAATGGG   
  
  
- AAATTTTTAT TTTGTTTTTA TTCACCGCTG AGGTTCAGTA AAAAGAATTA GTTATTTCTA GTAAAAAAGC   
  
  
- TTTTGCTTTT AGCTCGAGCG GTAGCTCACC CTTTGCGTAA CTCGGCTTTA CGCCCCAGGT ATCCATACAA   
  
  
- AAAATAAATA CTCTATTTTT CATTTATTAT AAAAAAAGCC CAAATTTTTT ATATAATAAC TAATGAACAT   
  
  
- TAATATACTC GGAGCAAACA TGAATAATAA TTTATGTATA AGTTCCATTT TAATAGGAAA TTTTTTAAGA   
  
  
- ATCCGGCTCC CTTGGAAGGA TAATTATATT CAGTAGGTCG GGTGGAATAC CCCCATTAAG AGGGGTATAT   
  
  
- GGTACCCAGG TTTAGCCGCC GGGCCAGTCA AGTTGCTGTC ATGCTACCGT GAGACTTGGG ACACGACGTA   
  
  
- GACGAGTGTT TTTCATGTGG TGTCAACAGA AAGTGAGAGA GACGGGTAAG TGATACGTTT GTGCAAATAT   
  
  
- TTTAACCAAT AAAATATTTG CAAAGAGAAA GTTTACCAAT AAAATATAAT CATTGTTTTT ATAATATTAT   
  
  
- TATTTTTTTA GTTATAATGC ATTAACGATT TTTACTTTAA TGTAGTACAA CATTCTATAA AGTAAACGGA   
  
  
- TAATTATTAA AAAATAATAA ATATTTTAAC CCAACTTAAA GTTATAAAAA ATTTTATTCA GAATAAATAA   
  
  
- ATTCACTTTA AAAAAAATAT CTATTTCAGT GTTTTTAGTA AAAAGATTTA TTAAATATTT TTCTATGAAA   
  
  
- AGGTTATTTT TAATTTAATT TTACGAAAAT ATTTATTTTA ACATTGTTAA GTAAGGTATC GTTTCCATTT   
  
  
- ATTTCGGTAA AAGAATAGTT AATTTAGTTC GAAGAGAAAA AATACTTTAA CAAAAATTAT TTAAGAAAAT   
  
  
- CATTGTTATT CTAAAACAAG TATAAAATTA TGTAGAGGAA CCGTTAACCC GTTTCTACCG ACCATCACTG   
  
  
- TACTTAGGTT CGTACTTAAT CAGGCCCGCA ACAATGAACG ATGTTCCCAA TACTCGTACC TTCCTTTTTA   
  
  
- TTAATTTTTT ATTTTTTTTA ATTTAATGAA GTTAGTGTTT CAACTTTTAT TATTTTAACA CAAACCTTCA   
  
  
- TAAACATGAA TTTCAAAAAA TCATTTTCAC AAAGGGATTT TCCACAAAAA AAATCTCTTT CGTCGATATT   
  
  
- TTACAAAAAA GTTTTTATAA AAAAATAAAT TTTTGTGAAA GAATTTAGTG AACAATATTC AGTGGAATCT   
  
  
- TACTGACTGC CACAACGTGT ATAGTTTTTC GTAAATACGG GATCACGGTA AAATAATAAA ATATAGAATT   
  
  
- AAAATCTATA AAAATAAAAA TAAATTACTA AATATAAACT TGCAATTAAA AGTTATATAG TATAACATAG   
  
  
- AAAATATCGG TGCAAATAAG TTTATATAAA AAATATAAAT ATTTATAAAA AAGATTTTAT ACTAAGCTCT   
  
  
- TACGACTGTA CACTTAGCGT ACCCCCATCC CGTTCGGTAG AACCTGGGTA ATTTGAAGTT TAAGGTACTA   
  
  
- ACTGATCCTG AACGGGTTTT TAAAGTAATG GGGTCTACGG GTGTACACTT ACGTCAGAGA CTGTCTCATC   
  
  
- CACCCTTATT TGGTTTTACG TAAATAAATA AAAAGTTCTA AAATTAATAA TAAGTGGATT TTAGGAAGTA   
  
  
- TCCCTAATTT TATAATCTGG TAACCTAAAC GAGAGGAGTT ATCCCAACTT GAAACTAATC ATGCAAATAT   
  
  
- AATAAAAAAA GGAAAAAGCA GTTTTACGTA AGACAGTGTT TTAATTACAT CAGTTCCTTA ACTGTTCACT   
  
  
- CTTCTCTCAG TGAAATTTAC TAAGTCGACA GGTGTAATTT CTATTCAGTG TAAACACCAG GTATGAGGGG   
  
  
- TTCTGAAATT AAACTTAAAT CGAGTCTGAA AAGCATAAAA TAAACACAAA AAGATAAATT CGATAGTTAT   
  
  
- GTAAATATTT ATTCACTAAA ATTATATTTT CTAGTTCATT TTTTGTTTAT TTCATGTATT TTATAAAAAT   
  
  
- AAGTTAATGT ATAACATAGT TTAAAAGTTC TCGTTTTAAA TATCGTAAAA AGTTACAAAA CCTTTAGTTT   
  
  
- TTTCATTATA TAATAGTCAA GTGATCAATT TAACCACTAA CTTGGTATCT ACTACAGTAT TATATATAAA   
  
  
- ATATAAATAA TTTTAATTTT TATAAATATT CTTTAAAATT TATATACTCT TTAATTTTCA GTTATTATAA   
  
  
- AAAAAGAAGT AGAAATTATA ATAGTTTACT TATATATATA TATATATTTA CATAACTATA AAATTTTAAA   
  
  
- ACAATTTATT TTATATATAT TATACTTCTA TAAATAACTT TCAATAAATT AAATGGATAT ATATATAATT   
  
  
- TTCAATAAAT ACCAACATTT TTTATAATGT AATAAAATAA ATATAATAAT TATAATGTAA TAAAATAAAT   
  
  
- ATAATAAATA TTAAAGTAAT AATATTTTTC AAACTTTTAT AATAACGATA TATTAAACAA CAACACACTT   
  
  
- CTAGCCGTTT TTTTGTCCTC ATCGTCACAC CACAGCAGGA ACGGGGAACT TGACCTCCAA CCAAAGGGTA   
  
  
- AGGAGGGAGG GTGCTTAGGG TGTACATTTC GTTTATTTTC TCGAAATGTA TGTGTACTAA TTACATAAAA   
  
  
- ATAACAGTTA TTTAAAATAT TATTTTGAAT ATTTAGAATA TTTAAATTAT CCTTTTTTTT TTCAGAAGAT   
  
  
- CTTAACTAAA ATATTATGTG GTATGGTTCT AGTGTACTTA ACGTACACAG CCACCTGTTC CAACGGTTTT   
  
  
- AAGGAATAAG GATATGTCTT AGAAAAGTAA CAATATAATA GTATCAGCTT TGGGTCATCA TATATTTCTC   
  
  
- CAATGCTCAC CTCCTACCTA TACTTTATTT ATTCTCCGGA ATCTCTAGTC CCACACTCTG TACCTAAACC   
  
  
- GTTTACCACT CTCTAGATCA CTCG

+     G-Box

| Site Name | Organism | Position | Strand | Matrix score. | sequence | function |
| --- | --- | --- | --- | --- | --- | --- |
| G-Box | Pisum sativum | 1321 | + | 6 | CACGTT | cis-acting regulatory element involved in light responsiveness |
| G-Box | Pisum sativum | 2250 | + | 6 | CACGTT | cis-acting regulatory element involved in light responsiveness |

>PlantCARE\_32120   
+ AATCATTAAA GTCTAATTCT TCAAAATTCC GAATTCTAAA TTTAGCTATT TAAAATTCCA ATTGTCCTAT   
  
  
+ CTCCGAACTT AATTTTCAGT TTCCCATGCT CCAATTCCCG TATTTACCCC GCTACTTATT ATTATCATTA   
  
  
+ TCATTATCAC CTTATTCATT ATTTCTATAA ATTCCGCCTT CGAATGATTT CTAAGATCTC CAATTTTTAT   
  
  
+ ACATCAACTT TCATAATTTC TACGTCTCAA ATAACATACG ATTCTGATTC CTTTCAAAAT TTAACTCCCA   
  
  
+ TAATTCCAAT TTTCGTAACT TAATTTTTCT TAAAAATCCC GAACTTTCAA AATCCCAATT TTCTTTCAAA   
  
  
+ TTTAATTTCT AAATGTTCTC ATTCTTACAT CTAATTCCTA AGAATCCAAT TTTCAAATAA TCTCTAAGAT   
  
  
+ TCCGATTCTC AAATGAATTT CAAAAATCCA GTTTCCCTTC GAACAATTTT AATCCCTGTT TAGTGATGCA   
  
  
+ TGTAATATGT TTTGTGCTCT ATATGGTGTA CTAACCCTCT CTATTGATAG CGCATGGTGG TTCGTGGCCC   
  
  
+ AGGTATGTAC CCATTTTCTC TCTAATCGTT GTCTATGCAT GTCTCGATTC TTATGTGTGC ACGATCATTC   
  
  
+ AGGATATTCA TTGATTTACT CATCAATTGT CACGGCAGCT TCATTTTATT AGTAGAGACC CGACTTTAGG   
  
  
+ GACTTAGAGG GGTGCTACGG TCCTTACCGT ACCTTCCTGA TAAGTAACCT GACCCCCGAA CCCGATCCGG   
  
  
+ TTTTTCACAG ACCATCTTTT CCAAAATAAG GAGTCACACT TAGGGTTTTT CTTTCTTATT TTGTTTACCC   
  
  
+ TTTAAAAATA AAACAAAAAT AAGTGGCGAC TCCAAGTCAT TTTTCTTAAT CAATAAAGAT CATTTTTTCG   
  
  
+ AAAACGAAAA TCGAGCTCGC CATCGAGTGG GAAACGCATT GAGCCGAAAT GCGGGGTCCA TAGGTATGTT   
  
  
+ TTTTATTTAT GAGATAAAAA GTAAATAATA TTTTTTTCGG GTTTAAAAAA TATATTATTG ATTACTTGTA   
  
  
+ ATTATATGAG CCTCGTTTGT ACTTATTATT AAATACATAT TCAAGGTAAA ATTATCCTTT AAAAAATTCT   
  
  
+ TAGGCCGAGG GAACCTTCCT ATTAATATAA GTCATCCAGC CCACCTTATG GGGGTAATTC TCCCCATATA   
  
  
+ CCATGGGTCC AAATCGGCGG CCCGGTCAGT TCAACGACAG TACGATGGCA CTCTGAACCC TGTGCTGCAT   
  
  
+ CTGCTCACAA AAAGTACACC ACAGTTGTCT TTCACTCTCT CTGCCCATTC ACTATGCAAA CACGTTTATA   
  
  
+ AAATTGGTTA TTTTATAAAC GTTTCTCTTT CAAATGGTTA TTTTATATTA GTAACAAAAA TATTATAATA   
  
  
+ ATAAAAAAAT CAATATTACG TAATTGCTAA AAATGAAATT ACATCATGTT GTAAGATATT TCATTTGCCT   
  
  
+ ATTAATAATT TTTTATTATT TATAAAATTG GGTTGAATTT CAATATTTTT TAAAATAAGT CTTATTTATT   
  
  
+ TAAGTGAAAT TTTTTTTATA GATAAAGTCA CAAAAATCAT TTTTCTAAAT AATTTATAAA AAGATACTTT   
  
  
+ TCCAATAAAA ATTAAATTAA AATGCTTTTA TAAATAAAAT TGTAACAATT CATTCCATAG CAAAGGTAAA   
  
  
+ TAAAGCCATT TTCTTATCAA TTAAATCAAG CTTCTCTTTT TTATGAAATT GTTTTTAATA AATTCTTTTA   
  
  
+ GTAACAATAA GATTTTGTTC ATATTTTAAT ACATCTCCTT GGCAATTGGG CAAAGATGGC TGGTAGTGAC   
  
  
+ ATGAATCCAA GCATGAATTA GTCCGGGCGT TGTTACTTGC TACAAGGGTT ATGAGCATGG AAGGAAAAAT   
  
  
+ AATTAAAAAA TAAAAAAAAT TAAATTACTT CAATCACAAA GTTGAAAATA ATAAAATTGT GTTTGGAAGT   
  
  
+ ATTTGTACTT AAAGTTTTTT AGTAAAAGTG TTTCCCTAAA AGGTGTTTTT TTTAGAGAAA GCAGCTATAA   
  
  
+ AATGTTTTTT CAAAAATATT TTTTTATTTA AAAACACTTT CTTAAATCAC TTGTTATAAG TCACCTTAGA   
  
  
+ ATGACTGACG GTGTTGCACA TATCAAAAAG CATTTATGCC CTAGTGCCAT TTTATTATTT TATATCTTAA   
  
  
+ TTTTAGATAT TTTTATTTTT ATTTAATGAT TTATATTTGA ACGTTAATTT TCAATATATC ATATTGTATC   
  
  
+ TTTTATAGCC ACGTTTATTC AAATATATTT TTTATATTTA TAAATATTTT TTCTAAAATA TGATTCGAGA   
  
  
+ ATGCTGACAT GTGAATCGCA TGGGGGTAGG GCAAGCCATC TTGGACCCAT TAAACTTCAA ATTCCATGAT   
  
  
+ TGACTAGGAC TTGCCCAAAA ATTTCATTAC CCCAGATGCC CACATGTGAA TGCAGTCTCT GACAGAGTAG   
  
  
+ GTGGGAATAA ACCAAAATGC ATTTATTTAT TTTTCAAGAT TTTAATTATT ATTCACCTAA AATCCTTCAT   
  
  
+ AGGGATTAAA ATATTAGACC ATTGGATTTG CTCTCCTCAA TAGGGTTGAA CTTTGATTAG TACGTTTATA   
  
  
+ TTATTTTTTT CCTTTTTCGT CAAAATGCAT TCTGTCACAA AATTAATGTA GTCAAGGAAT TGACAAGTGA   
  
  
+ GAAGAGAGTC ACTTTAAATG ATTCAGCTGT CCACATTAAA GATAAGTCAC ATTTGTGGTC CATACTCCCC   
  
  
+ AAGACTTTAA TTTGAATTTA GCTCAGACTT TTCGTATTTT ATTTGTGTTT TTCTATTTAA GCTATCAATA   
  
  
+ CATTTATAAA TAAGTGATTT TAATATAAAA GATCAAGTAA AAAACAAATA AAGTACATAA AATATTTTTA   
  
  
+ TTCAATTACA TATTGTATCA AATTTTCAAG AGCAAAATTT ATAGCATTTT TCAATGTTTT GGAAATCAAA   
  
  
+ AAAGTAATAT ATTATCAGTT CACTAGTTAA ATTGGTGATT GAACCATAGA TGATGTCATA ATATATATTT   
  
  
+ TATATTTATT AAAATTAAAA ATATTTATAA GAAATTTTAA ATATATGAGA AATTAAAAGT CAATAATATT   
  
  
+ TTTTTCTTCA TCTTTAATAT TATCAAATGA ATATATATAT ATATATAAAT GTATTGATAT TTTAAAATTT   
  
  
+ TGTTAAATAA AATATATATA ATATGAAGAT ATTTATTGAA AGTTATTTAA TTTACCTATA TATATATTAA   
  
  
+ AAGTTATTTA TGGTTGTAAA AAATATTACA TTATTTTATT TATATTATTA ATATTACATT ATTTTATTTA   
  
  
+ TATTATTTAT AATTTCATTA TTATAAAAAG TTTGAAAATA TTATTGCTAT ATAATTTGTT GTTGTGTGAA   
  
  
+ GATCGGCAAA AAAACAGGAG TAGCAGTGTG GTGTCGTCCT TGCCCCTTGA ACTGGAGGTT GGTTTCCCAT   
  
  
+ TCCTCCCTCC CACGAATCCC ACATGTAAAG CAAATAAAAG AGCTTTACAT ACACATGATT AATGTATTTT   
  
  
+ TATTGTCAAT AAATTTTATA ATAAAACTTA TAAATCTTAT AAATTTAATA GGAAAAAAAA AAGTCTTCTA   
  
  
+ GAATTGATTT TATAATACAC CATACCAAGA TCACATGAAT TGCATGTGTC GGTGGACAAG GTTGCCAAAA   
  
  
+ TTCCTTATTC CTATACAGAA TCTTTTCATT GTTATATTAT CATAGTCGAA ACCCAGTAGT ATATAAAGAG   
  
  
+ GTTACGAGTG GAGGATGGAT ATGAAATAAA TAAGAGGCCT TAGAGATCAG GGTGTGAGAC ATGGATTTGG   
  
  
+ CAAATGGTGA GAGATCTAGT GAGC  

- TTAGTAATTT CAGATTAAGA AGTTTTAAGG CTTAAGATTT AAATCGATAA ATTTTAAGGT TAACAGGATA   
  
  
- GAGGCTTGAA TTAAAAGTCA AAGGGTACGA GGTTAAGGGC ATAAATGGGG CGATGAATAA TAATAGTAAT   
  
  
- AGTAATAGTG GAATAAGTAA TAAAGATATT TAAGGCGGAA GCTTACTAAA GATTCTAGAG GTTAAAAATA   
  
  
- TGTAGTTGAA AGTATTAAAG ATGCAGAGTT TATTGTATGC TAAGACTAAG GAAAGTTTTA AATTGAGGGT   
  
  
- ATTAAGGTTA AAAGCATTGA ATTAAAAAGA ATTTTTAGGG CTTGAAAGTT TTAGGGTTAA AAGAAAGTTT   
  
  
- AAATTAAAGA TTTACAAGAG TAAGAATGTA GATTAAGGAT TCTTAGGTTA AAAGTTTATT AGAGATTCTA   
  
  
- AGGCTAAGAG TTTACTTAAA GTTTTTAGGT CAAAGGGAAG CTTGTTAAAA TTAGGGACAA ATCACTACGT   
  
  
- ACATTATACA AAACACGAGA TATACCACAT GATTGGGAGA GATAACTATC GCGTACCACC AAGCACCGGG   
  
  
- TCCATACATG GGTAAAAGAG AGATTAGCAA CAGATACGTA CAGAGCTAAG AATACACACG TGCTAGTAAG   
  
  
- TCCTATAAGT AACTAAATGA GTAGTTAACA GTGCCGTCGA AGTAAAATAA TCATCTCTGG GCTGAAATCC   
  
  
- CTGAATCTCC CCACGATGCC AGGAATGGCA TGGAAGGACT ATTCATTGGA CTGGGGGCTT GGGCTAGGCC   
  
  
- AAAAAGTGTC TGGTAGAAAA GGTTTTATTC CTCAGTGTGA ATCCCAAAAA GAAAGAATAA AACAAATGGG   
  
  
- AAATTTTTAT TTTGTTTTTA TTCACCGCTG AGGTTCAGTA AAAAGAATTA GTTATTTCTA GTAAAAAAGC   
  
  
- TTTTGCTTTT AGCTCGAGCG GTAGCTCACC CTTTGCGTAA CTCGGCTTTA CGCCCCAGGT ATCCATACAA   
  
  
- AAAATAAATA CTCTATTTTT CATTTATTAT AAAAAAAGCC CAAATTTTTT ATATAATAAC TAATGAACAT   
  
  
- TAATATACTC GGAGCAAACA TGAATAATAA TTTATGTATA AGTTCCATTT TAATAGGAAA TTTTTTAAGA   
  
  
- ATCCGGCTCC CTTGGAAGGA TAATTATATT CAGTAGGTCG GGTGGAATAC CCCCATTAAG AGGGGTATAT   
  
  
- GGTACCCAGG TTTAGCCGCC GGGCCAGTCA AGTTGCTGTC ATGCTACCGT GAGACTTGGG ACACGACGTA   
  
  
- GACGAGTGTT TTTCATGTGG TGTCAACAGA AAGTGAGAGA GACGGGTAAG TGATACGTTT GTGCAAATAT   
  
  
- TTTAACCAAT AAAATATTTG CAAAGAGAAA GTTTACCAAT AAAATATAAT CATTGTTTTT ATAATATTAT   
  
  
- TATTTTTTTA GTTATAATGC ATTAACGATT TTTACTTTAA TGTAGTACAA CATTCTATAA AGTAAACGGA   
  
  
- TAATTATTAA AAAATAATAA ATATTTTAAC CCAACTTAAA GTTATAAAAA ATTTTATTCA GAATAAATAA   
  
  
- ATTCACTTTA AAAAAAATAT CTATTTCAGT GTTTTTAGTA AAAAGATTTA TTAAATATTT TTCTATGAAA   
  
  
- AGGTTATTTT TAATTTAATT TTACGAAAAT ATTTATTTTA ACATTGTTAA GTAAGGTATC GTTTCCATTT   
  
  
- ATTTCGGTAA AAGAATAGTT AATTTAGTTC GAAGAGAAAA AATACTTTAA CAAAAATTAT TTAAGAAAAT   
  
  
- CATTGTTATT CTAAAACAAG TATAAAATTA TGTAGAGGAA CCGTTAACCC GTTTCTACCG ACCATCACTG   
  
  
- TACTTAGGTT CGTACTTAAT CAGGCCCGCA ACAATGAACG ATGTTCCCAA TACTCGTACC TTCCTTTTTA   
  
  
- TTAATTTTTT ATTTTTTTTA ATTTAATGAA GTTAGTGTTT CAACTTTTAT TATTTTAACA CAAACCTTCA   
  
  
- TAAACATGAA TTTCAAAAAA TCATTTTCAC AAAGGGATTT TCCACAAAAA AAATCTCTTT CGTCGATATT   
  
  
- TTACAAAAAA GTTTTTATAA AAAAATAAAT TTTTGTGAAA GAATTTAGTG AACAATATTC AGTGGAATCT   
  
  
- TACTGACTGC CACAACGTGT ATAGTTTTTC GTAAATACGG GATCACGGTA AAATAATAAA ATATAGAATT   
  
  
- AAAATCTATA AAAATAAAAA TAAATTACTA AATATAAACT TGCAATTAAA AGTTATATAG TATAACATAG   
  
  
- AAAATATCGG TGCAAATAAG TTTATATAAA AAATATAAAT ATTTATAAAA AAGATTTTAT ACTAAGCTCT   
  
  
- TACGACTGTA CACTTAGCGT ACCCCCATCC CGTTCGGTAG AACCTGGGTA ATTTGAAGTT TAAGGTACTA   
  
  
- ACTGATCCTG AACGGGTTTT TAAAGTAATG GGGTCTACGG GTGTACACTT ACGTCAGAGA CTGTCTCATC   
  
  
- CACCCTTATT TGGTTTTACG TAAATAAATA AAAAGTTCTA AAATTAATAA TAAGTGGATT TTAGGAAGTA   
  
  
- TCCCTAATTT TATAATCTGG TAACCTAAAC GAGAGGAGTT ATCCCAACTT GAAACTAATC ATGCAAATAT   
  
  
- AATAAAAAAA GGAAAAAGCA GTTTTACGTA AGACAGTGTT TTAATTACAT CAGTTCCTTA ACTGTTCACT   
  
  
- CTTCTCTCAG TGAAATTTAC TAAGTCGACA GGTGTAATTT CTATTCAGTG TAAACACCAG GTATGAGGGG   
  
  
- TTCTGAAATT AAACTTAAAT CGAGTCTGAA AAGCATAAAA TAAACACAAA AAGATAAATT CGATAGTTAT   
  
  
- GTAAATATTT ATTCACTAAA ATTATATTTT CTAGTTCATT TTTTGTTTAT TTCATGTATT TTATAAAAAT   
  
  
- AAGTTAATGT ATAACATAGT TTAAAAGTTC TCGTTTTAAA TATCGTAAAA AGTTACAAAA CCTTTAGTTT   
  
  
- TTTCATTATA TAATAGTCAA GTGATCAATT TAACCACTAA CTTGGTATCT ACTACAGTAT TATATATAAA   
  
  
- ATATAAATAA TTTTAATTTT TATAAATATT CTTTAAAATT TATATACTCT TTAATTTTCA GTTATTATAA   
  
  
- AAAAAGAAGT AGAAATTATA ATAGTTTACT TATATATATA TATATATTTA CATAACTATA AAATTTTAAA   
  
  
- ACAATTTATT TTATATATAT TATACTTCTA TAAATAACTT TCAATAAATT AAATGGATAT ATATATAATT   
  
  
- TTCAATAAAT ACCAACATTT TTTATAATGT AATAAAATAA ATATAATAAT TATAATGTAA TAAAATAAAT   
  
  
- ATAATAAATA TTAAAGTAAT AATATTTTTC AAACTTTTAT AATAACGATA TATTAAACAA CAACACACTT   
  
  
- CTAGCCGTTT TTTTGTCCTC ATCGTCACAC CACAGCAGGA ACGGGGAACT TGACCTCCAA CCAAAGGGTA   
  
  
- AGGAGGGAGG GTGCTTAGGG TGTACATTTC GTTTATTTTC TCGAAATGTA TGTGTACTAA TTACATAAAA   
  
  
- ATAACAGTTA TTTAAAATAT TATTTTGAAT ATTTAGAATA TTTAAATTAT CCTTTTTTTT TTCAGAAGAT   
  
  
- CTTAACTAAA ATATTATGTG GTATGGTTCT AGTGTACTTA ACGTACACAG CCACCTGTTC CAACGGTTTT   
  
  
- AAGGAATAAG GATATGTCTT AGAAAAGTAA CAATATAATA GTATCAGCTT TGGGTCATCA TATATTTCTC   
  
  
- CAATGCTCAC CTCCTACCTA TACTTTATTT ATTCTCCGGA ATCTCTAGTC CCACACTCTG TACCTAAACC   
  
  
- GTTTACCACT CTCTAGATCA CTCG

+     G-box

| Site Name | Organism | Position | Strand | Matrix score. | sequence | function |
| --- | --- | --- | --- | --- | --- | --- |
| G-box | Brassica oleracea | 1321 | - | 8 | TAAACGTG | cis-acting regulatory element involved in light responsiveness |
| G-box | Brassica oleracea | 2250 | - | 8 | TAAACGTG | cis-acting regulatory element involved in light responsiveness |

>PlantCARE\_32120   
+ AATCATTAAA GTCTAATTCT TCAAAATTCC GAATTCTAAA TTTAGCTATT TAAAATTCCA ATTGTCCTAT   
  
  
+ CTCCGAACTT AATTTTCAGT TTCCCATGCT CCAATTCCCG TATTTACCCC GCTACTTATT ATTATCATTA   
  
  
+ TCATTATCAC CTTATTCATT ATTTCTATAA ATTCCGCCTT CGAATGATTT CTAAGATCTC CAATTTTTAT   
  
  
+ ACATCAACTT TCATAATTTC TACGTCTCAA ATAACATACG ATTCTGATTC CTTTCAAAAT TTAACTCCCA   
  
  
+ TAATTCCAAT TTTCGTAACT TAATTTTTCT TAAAAATCCC GAACTTTCAA AATCCCAATT TTCTTTCAAA   
  
  
+ TTTAATTTCT AAATGTTCTC ATTCTTACAT CTAATTCCTA AGAATCCAAT TTTCAAATAA TCTCTAAGAT   
  
  
+ TCCGATTCTC AAATGAATTT CAAAAATCCA GTTTCCCTTC GAACAATTTT AATCCCTGTT TAGTGATGCA   
  
  
+ TGTAATATGT TTTGTGCTCT ATATGGTGTA CTAACCCTCT CTATTGATAG CGCATGGTGG TTCGTGGCCC   
  
  
+ AGGTATGTAC CCATTTTCTC TCTAATCGTT GTCTATGCAT GTCTCGATTC TTATGTGTGC ACGATCATTC   
  
  
+ AGGATATTCA TTGATTTACT CATCAATTGT CACGGCAGCT TCATTTTATT AGTAGAGACC CGACTTTAGG   
  
  
+ GACTTAGAGG GGTGCTACGG TCCTTACCGT ACCTTCCTGA TAAGTAACCT GACCCCCGAA CCCGATCCGG   
  
  
+ TTTTTCACAG ACCATCTTTT CCAAAATAAG GAGTCACACT TAGGGTTTTT CTTTCTTATT TTGTTTACCC   
  
  
+ TTTAAAAATA AAACAAAAAT AAGTGGCGAC TCCAAGTCAT TTTTCTTAAT CAATAAAGAT CATTTTTTCG   
  
  
+ AAAACGAAAA TCGAGCTCGC CATCGAGTGG GAAACGCATT GAGCCGAAAT GCGGGGTCCA TAGGTATGTT   
  
  
+ TTTTATTTAT GAGATAAAAA GTAAATAATA TTTTTTTCGG GTTTAAAAAA TATATTATTG ATTACTTGTA   
  
  
+ ATTATATGAG CCTCGTTTGT ACTTATTATT AAATACATAT TCAAGGTAAA ATTATCCTTT AAAAAATTCT   
  
  
+ TAGGCCGAGG GAACCTTCCT ATTAATATAA GTCATCCAGC CCACCTTATG GGGGTAATTC TCCCCATATA   
  
  
+ CCATGGGTCC AAATCGGCGG CCCGGTCAGT TCAACGACAG TACGATGGCA CTCTGAACCC TGTGCTGCAT   
  
  
+ CTGCTCACAA AAAGTACACC ACAGTTGTCT TTCACTCTCT CTGCCCATTC ACTATGCAAA CACGTTTATA   
  
  
+ AAATTGGTTA TTTTATAAAC GTTTCTCTTT CAAATGGTTA TTTTATATTA GTAACAAAAA TATTATAATA   
  
  
+ ATAAAAAAAT CAATATTACG TAATTGCTAA AAATGAAATT ACATCATGTT GTAAGATATT TCATTTGCCT   
  
  
+ ATTAATAATT TTTTATTATT TATAAAATTG GGTTGAATTT CAATATTTTT TAAAATAAGT CTTATTTATT   
  
  
+ TAAGTGAAAT TTTTTTTATA GATAAAGTCA CAAAAATCAT TTTTCTAAAT AATTTATAAA AAGATACTTT   
  
  
+ TCCAATAAAA ATTAAATTAA AATGCTTTTA TAAATAAAAT TGTAACAATT CATTCCATAG CAAAGGTAAA   
  
  
+ TAAAGCCATT TTCTTATCAA TTAAATCAAG CTTCTCTTTT TTATGAAATT GTTTTTAATA AATTCTTTTA   
  
  
+ GTAACAATAA GATTTTGTTC ATATTTTAAT ACATCTCCTT GGCAATTGGG CAAAGATGGC TGGTAGTGAC   
  
  
+ ATGAATCCAA GCATGAATTA GTCCGGGCGT TGTTACTTGC TACAAGGGTT ATGAGCATGG AAGGAAAAAT   
  
  
+ AATTAAAAAA TAAAAAAAAT TAAATTACTT CAATCACAAA GTTGAAAATA ATAAAATTGT GTTTGGAAGT   
  
  
+ ATTTGTACTT AAAGTTTTTT AGTAAAAGTG TTTCCCTAAA AGGTGTTTTT TTTAGAGAAA GCAGCTATAA   
  
  
+ AATGTTTTTT CAAAAATATT TTTTTATTTA AAAACACTTT CTTAAATCAC TTGTTATAAG TCACCTTAGA   
  
  
+ ATGACTGACG GTGTTGCACA TATCAAAAAG CATTTATGCC CTAGTGCCAT TTTATTATTT TATATCTTAA   
  
  
+ TTTTAGATAT TTTTATTTTT ATTTAATGAT TTATATTTGA ACGTTAATTT TCAATATATC ATATTGTATC   
  
  
+ TTTTATAGCC ACGTTTATTC AAATATATTT TTTATATTTA TAAATATTTT TTCTAAAATA TGATTCGAGA   
  
  
+ ATGCTGACAT GTGAATCGCA TGGGGGTAGG GCAAGCCATC TTGGACCCAT TAAACTTCAA ATTCCATGAT   
  
  
+ TGACTAGGAC TTGCCCAAAA ATTTCATTAC CCCAGATGCC CACATGTGAA TGCAGTCTCT GACAGAGTAG   
  
  
+ GTGGGAATAA ACCAAAATGC ATTTATTTAT TTTTCAAGAT TTTAATTATT ATTCACCTAA AATCCTTCAT   
  
  
+ AGGGATTAAA ATATTAGACC ATTGGATTTG CTCTCCTCAA TAGGGTTGAA CTTTGATTAG TACGTTTATA   
  
  
+ TTATTTTTTT CCTTTTTCGT CAAAATGCAT TCTGTCACAA AATTAATGTA GTCAAGGAAT TGACAAGTGA   
  
  
+ GAAGAGAGTC ACTTTAAATG ATTCAGCTGT CCACATTAAA GATAAGTCAC ATTTGTGGTC CATACTCCCC   
  
  
+ AAGACTTTAA TTTGAATTTA GCTCAGACTT TTCGTATTTT ATTTGTGTTT TTCTATTTAA GCTATCAATA   
  
  
+ CATTTATAAA TAAGTGATTT TAATATAAAA GATCAAGTAA AAAACAAATA AAGTACATAA AATATTTTTA   
  
  
+ TTCAATTACA TATTGTATCA AATTTTCAAG AGCAAAATTT ATAGCATTTT TCAATGTTTT GGAAATCAAA   
  
  
+ AAAGTAATAT ATTATCAGTT CACTAGTTAA ATTGGTGATT GAACCATAGA TGATGTCATA ATATATATTT   
  
  
+ TATATTTATT AAAATTAAAA ATATTTATAA GAAATTTTAA ATATATGAGA AATTAAAAGT CAATAATATT   
  
  
+ TTTTTCTTCA TCTTTAATAT TATCAAATGA ATATATATAT ATATATAAAT GTATTGATAT TTTAAAATTT   
  
  
+ TGTTAAATAA AATATATATA ATATGAAGAT ATTTATTGAA AGTTATTTAA TTTACCTATA TATATATTAA   
  
  
+ AAGTTATTTA TGGTTGTAAA AAATATTACA TTATTTTATT TATATTATTA ATATTACATT ATTTTATTTA   
  
  
+ TATTATTTAT AATTTCATTA TTATAAAAAG TTTGAAAATA TTATTGCTAT ATAATTTGTT GTTGTGTGAA   
  
  
+ GATCGGCAAA AAAACAGGAG TAGCAGTGTG GTGTCGTCCT TGCCCCTTGA ACTGGAGGTT GGTTTCCCAT   
  
  
+ TCCTCCCTCC CACGAATCCC ACATGTAAAG CAAATAAAAG AGCTTTACAT ACACATGATT AATGTATTTT   
  
  
+ TATTGTCAAT AAATTTTATA ATAAAACTTA TAAATCTTAT AAATTTAATA GGAAAAAAAA AAGTCTTCTA   
  
  
+ GAATTGATTT TATAATACAC CATACCAAGA TCACATGAAT TGCATGTGTC GGTGGACAAG GTTGCCAAAA   
  
  
+ TTCCTTATTC CTATACAGAA TCTTTTCATT GTTATATTAT CATAGTCGAA ACCCAGTAGT ATATAAAGAG   
  
  
+ GTTACGAGTG GAGGATGGAT ATGAAATAAA TAAGAGGCCT TAGAGATCAG GGTGTGAGAC ATGGATTTGG   
  
  
+ CAAATGGTGA GAGATCTAGT GAGC  

- TTAGTAATTT CAGATTAAGA AGTTTTAAGG CTTAAGATTT AAATCGATAA ATTTTAAGGT TAACAGGATA   
  
  
- GAGGCTTGAA TTAAAAGTCA AAGGGTACGA GGTTAAGGGC ATAAATGGGG CGATGAATAA TAATAGTAAT   
  
  
- AGTAATAGTG GAATAAGTAA TAAAGATATT TAAGGCGGAA GCTTACTAAA GATTCTAGAG GTTAAAAATA   
  
  
- TGTAGTTGAA AGTATTAAAG ATGCAGAGTT TATTGTATGC TAAGACTAAG GAAAGTTTTA AATTGAGGGT   
  
  
- ATTAAGGTTA AAAGCATTGA ATTAAAAAGA ATTTTTAGGG CTTGAAAGTT TTAGGGTTAA AAGAAAGTTT   
  
  
- AAATTAAAGA TTTACAAGAG TAAGAATGTA GATTAAGGAT TCTTAGGTTA AAAGTTTATT AGAGATTCTA   
  
  
- AGGCTAAGAG TTTACTTAAA GTTTTTAGGT CAAAGGGAAG CTTGTTAAAA TTAGGGACAA ATCACTACGT   
  
  
- ACATTATACA AAACACGAGA TATACCACAT GATTGGGAGA GATAACTATC GCGTACCACC AAGCACCGGG   
  
  
- TCCATACATG GGTAAAAGAG AGATTAGCAA CAGATACGTA CAGAGCTAAG AATACACACG TGCTAGTAAG   
  
  
- TCCTATAAGT AACTAAATGA GTAGTTAACA GTGCCGTCGA AGTAAAATAA TCATCTCTGG GCTGAAATCC   
  
  
- CTGAATCTCC CCACGATGCC AGGAATGGCA TGGAAGGACT ATTCATTGGA CTGGGGGCTT GGGCTAGGCC   
  
  
- AAAAAGTGTC TGGTAGAAAA GGTTTTATTC CTCAGTGTGA ATCCCAAAAA GAAAGAATAA AACAAATGGG   
  
  
- AAATTTTTAT TTTGTTTTTA TTCACCGCTG AGGTTCAGTA AAAAGAATTA GTTATTTCTA GTAAAAAAGC   
  
  
- TTTTGCTTTT AGCTCGAGCG GTAGCTCACC CTTTGCGTAA CTCGGCTTTA CGCCCCAGGT ATCCATACAA   
  
  
- AAAATAAATA CTCTATTTTT CATTTATTAT AAAAAAAGCC CAAATTTTTT ATATAATAAC TAATGAACAT   
  
  
- TAATATACTC GGAGCAAACA TGAATAATAA TTTATGTATA AGTTCCATTT TAATAGGAAA TTTTTTAAGA   
  
  
- ATCCGGCTCC CTTGGAAGGA TAATTATATT CAGTAGGTCG GGTGGAATAC CCCCATTAAG AGGGGTATAT   
  
  
- GGTACCCAGG TTTAGCCGCC GGGCCAGTCA AGTTGCTGTC ATGCTACCGT GAGACTTGGG ACACGACGTA   
  
  
- GACGAGTGTT TTTCATGTGG TGTCAACAGA AAGTGAGAGA GACGGGTAAG TGATACGTTT GTGCAAATAT   
  
  
- TTTAACCAAT AAAATATTTG CAAAGAGAAA GTTTACCAAT AAAATATAAT CATTGTTTTT ATAATATTAT   
  
  
- TATTTTTTTA GTTATAATGC ATTAACGATT TTTACTTTAA TGTAGTACAA CATTCTATAA AGTAAACGGA   
  
  
- TAATTATTAA AAAATAATAA ATATTTTAAC CCAACTTAAA GTTATAAAAA ATTTTATTCA GAATAAATAA   
  
  
- ATTCACTTTA AAAAAAATAT CTATTTCAGT GTTTTTAGTA AAAAGATTTA TTAAATATTT TTCTATGAAA   
  
  
- AGGTTATTTT TAATTTAATT TTACGAAAAT ATTTATTTTA ACATTGTTAA GTAAGGTATC GTTTCCATTT   
  
  
- ATTTCGGTAA AAGAATAGTT AATTTAGTTC GAAGAGAAAA AATACTTTAA CAAAAATTAT TTAAGAAAAT   
  
  
- CATTGTTATT CTAAAACAAG TATAAAATTA TGTAGAGGAA CCGTTAACCC GTTTCTACCG ACCATCACTG   
  
  
- TACTTAGGTT CGTACTTAAT CAGGCCCGCA ACAATGAACG ATGTTCCCAA TACTCGTACC TTCCTTTTTA   
  
  
- TTAATTTTTT ATTTTTTTTA ATTTAATGAA GTTAGTGTTT CAACTTTTAT TATTTTAACA CAAACCTTCA   
  
  
- TAAACATGAA TTTCAAAAAA TCATTTTCAC AAAGGGATTT TCCACAAAAA AAATCTCTTT CGTCGATATT   
  
  
- TTACAAAAAA GTTTTTATAA AAAAATAAAT TTTTGTGAAA GAATTTAGTG AACAATATTC AGTGGAATCT   
  
  
- TACTGACTGC CACAACGTGT ATAGTTTTTC GTAAATACGG GATCACGGTA AAATAATAAA ATATAGAATT   
  
  
- AAAATCTATA AAAATAAAAA TAAATTACTA AATATAAACT TGCAATTAAA AGTTATATAG TATAACATAG   
  
  
- AAAATATCGG TGCAAATAAG TTTATATAAA AAATATAAAT ATTTATAAAA AAGATTTTAT ACTAAGCTCT   
  
  
- TACGACTGTA CACTTAGCGT ACCCCCATCC CGTTCGGTAG AACCTGGGTA ATTTGAAGTT TAAGGTACTA   
  
  
- ACTGATCCTG AACGGGTTTT TAAAGTAATG GGGTCTACGG GTGTACACTT ACGTCAGAGA CTGTCTCATC   
  
  
- CACCCTTATT TGGTTTTACG TAAATAAATA AAAAGTTCTA AAATTAATAA TAAGTGGATT TTAGGAAGTA   
  
  
- TCCCTAATTT TATAATCTGG TAACCTAAAC GAGAGGAGTT ATCCCAACTT GAAACTAATC ATGCAAATAT   
  
  
- AATAAAAAAA GGAAAAAGCA GTTTTACGTA AGACAGTGTT TTAATTACAT CAGTTCCTTA ACTGTTCACT   
  
  
- CTTCTCTCAG TGAAATTTAC TAAGTCGACA GGTGTAATTT CTATTCAGTG TAAACACCAG GTATGAGGGG   
  
  
- TTCTGAAATT AAACTTAAAT CGAGTCTGAA AAGCATAAAA TAAACACAAA AAGATAAATT CGATAGTTAT   
  
  
- GTAAATATTT ATTCACTAAA ATTATATTTT CTAGTTCATT TTTTGTTTAT TTCATGTATT TTATAAAAAT   
  
  
- AAGTTAATGT ATAACATAGT TTAAAAGTTC TCGTTTTAAA TATCGTAAAA AGTTACAAAA CCTTTAGTTT   
  
  
- TTTCATTATA TAATAGTCAA GTGATCAATT TAACCACTAA CTTGGTATCT ACTACAGTAT TATATATAAA   
  
  
- ATATAAATAA TTTTAATTTT TATAAATATT CTTTAAAATT TATATACTCT TTAATTTTCA GTTATTATAA   
  
  
- AAAAAGAAGT AGAAATTATA ATAGTTTACT TATATATATA TATATATTTA CATAACTATA AAATTTTAAA   
  
  
- ACAATTTATT TTATATATAT TATACTTCTA TAAATAACTT TCAATAAATT AAATGGATAT ATATATAATT   
  
  
- TTCAATAAAT ACCAACATTT TTTATAATGT AATAAAATAA ATATAATAAT TATAATGTAA TAAAATAAAT   
  
  
- ATAATAAATA TTAAAGTAAT AATATTTTTC AAACTTTTAT AATAACGATA TATTAAACAA CAACACACTT   
  
  
- CTAGCCGTTT TTTTGTCCTC ATCGTCACAC CACAGCAGGA ACGGGGAACT TGACCTCCAA CCAAAGGGTA   
  
  
- AGGAGGGAGG GTGCTTAGGG TGTACATTTC GTTTATTTTC TCGAAATGTA TGTGTACTAA TTACATAAAA   
  
  
- ATAACAGTTA TTTAAAATAT TATTTTGAAT ATTTAGAATA TTTAAATTAT CCTTTTTTTT TTCAGAAGAT   
  
  
- CTTAACTAAA ATATTATGTG GTATGGTTCT AGTGTACTTA ACGTACACAG CCACCTGTTC CAACGGTTTT   
  
  
- AAGGAATAAG GATATGTCTT AGAAAAGTAA CAATATAATA GTATCAGCTT TGGGTCATCA TATATTTCTC   
  
  
- CAATGCTCAC CTCCTACCTA TACTTTATTT ATTCTCCGGA ATCTCTAGTC CCACACTCTG TACCTAAACC   
  
  
- GTTTACCACT CTCTAGATCA CTCG

+     GA-motif

| Site Name | Organism | Position | Strand | Matrix score. | sequence | function |
| --- | --- | --- | --- | --- | --- | --- |
| GA-motif | Arabidopsis thaliana | 1558 | + | 8 | ATAGATAA | part of a light responsive element |

>PlantCARE\_32120   
+ AATCATTAAA GTCTAATTCT TCAAAATTCC GAATTCTAAA TTTAGCTATT TAAAATTCCA ATTGTCCTAT   
  
  
+ CTCCGAACTT AATTTTCAGT TTCCCATGCT CCAATTCCCG TATTTACCCC GCTACTTATT ATTATCATTA   
  
  
+ TCATTATCAC CTTATTCATT ATTTCTATAA ATTCCGCCTT CGAATGATTT CTAAGATCTC CAATTTTTAT   
  
  
+ ACATCAACTT TCATAATTTC TACGTCTCAA ATAACATACG ATTCTGATTC CTTTCAAAAT TTAACTCCCA   
  
  
+ TAATTCCAAT TTTCGTAACT TAATTTTTCT TAAAAATCCC GAACTTTCAA AATCCCAATT TTCTTTCAAA   
  
  
+ TTTAATTTCT AAATGTTCTC ATTCTTACAT CTAATTCCTA AGAATCCAAT TTTCAAATAA TCTCTAAGAT   
  
  
+ TCCGATTCTC AAATGAATTT CAAAAATCCA GTTTCCCTTC GAACAATTTT AATCCCTGTT TAGTGATGCA   
  
  
+ TGTAATATGT TTTGTGCTCT ATATGGTGTA CTAACCCTCT CTATTGATAG CGCATGGTGG TTCGTGGCCC   
  
  
+ AGGTATGTAC CCATTTTCTC TCTAATCGTT GTCTATGCAT GTCTCGATTC TTATGTGTGC ACGATCATTC   
  
  
+ AGGATATTCA TTGATTTACT CATCAATTGT CACGGCAGCT TCATTTTATT AGTAGAGACC CGACTTTAGG   
  
  
+ GACTTAGAGG GGTGCTACGG TCCTTACCGT ACCTTCCTGA TAAGTAACCT GACCCCCGAA CCCGATCCGG   
  
  
+ TTTTTCACAG ACCATCTTTT CCAAAATAAG GAGTCACACT TAGGGTTTTT CTTTCTTATT TTGTTTACCC   
  
  
+ TTTAAAAATA AAACAAAAAT AAGTGGCGAC TCCAAGTCAT TTTTCTTAAT CAATAAAGAT CATTTTTTCG   
  
  
+ AAAACGAAAA TCGAGCTCGC CATCGAGTGG GAAACGCATT GAGCCGAAAT GCGGGGTCCA TAGGTATGTT   
  
  
+ TTTTATTTAT GAGATAAAAA GTAAATAATA TTTTTTTCGG GTTTAAAAAA TATATTATTG ATTACTTGTA   
  
  
+ ATTATATGAG CCTCGTTTGT ACTTATTATT AAATACATAT TCAAGGTAAA ATTATCCTTT AAAAAATTCT   
  
  
+ TAGGCCGAGG GAACCTTCCT ATTAATATAA GTCATCCAGC CCACCTTATG GGGGTAATTC TCCCCATATA   
  
  
+ CCATGGGTCC AAATCGGCGG CCCGGTCAGT TCAACGACAG TACGATGGCA CTCTGAACCC TGTGCTGCAT   
  
  
+ CTGCTCACAA AAAGTACACC ACAGTTGTCT TTCACTCTCT CTGCCCATTC ACTATGCAAA CACGTTTATA   
  
  
+ AAATTGGTTA TTTTATAAAC GTTTCTCTTT CAAATGGTTA TTTTATATTA GTAACAAAAA TATTATAATA   
  
  
+ ATAAAAAAAT CAATATTACG TAATTGCTAA AAATGAAATT ACATCATGTT GTAAGATATT TCATTTGCCT   
  
  
+ ATTAATAATT TTTTATTATT TATAAAATTG GGTTGAATTT CAATATTTTT TAAAATAAGT CTTATTTATT   
  
  
+ TAAGTGAAAT TTTTTTTATA GATAAAGTCA CAAAAATCAT TTTTCTAAAT AATTTATAAA AAGATACTTT   
  
  
+ TCCAATAAAA ATTAAATTAA AATGCTTTTA TAAATAAAAT TGTAACAATT CATTCCATAG CAAAGGTAAA   
  
  
+ TAAAGCCATT TTCTTATCAA TTAAATCAAG CTTCTCTTTT TTATGAAATT GTTTTTAATA AATTCTTTTA   
  
  
+ GTAACAATAA GATTTTGTTC ATATTTTAAT ACATCTCCTT GGCAATTGGG CAAAGATGGC TGGTAGTGAC   
  
  
+ ATGAATCCAA GCATGAATTA GTCCGGGCGT TGTTACTTGC TACAAGGGTT ATGAGCATGG AAGGAAAAAT   
  
  
+ AATTAAAAAA TAAAAAAAAT TAAATTACTT CAATCACAAA GTTGAAAATA ATAAAATTGT GTTTGGAAGT   
  
  
+ ATTTGTACTT AAAGTTTTTT AGTAAAAGTG TTTCCCTAAA AGGTGTTTTT TTTAGAGAAA GCAGCTATAA   
  
  
+ AATGTTTTTT CAAAAATATT TTTTTATTTA AAAACACTTT CTTAAATCAC TTGTTATAAG TCACCTTAGA   
  
  
+ ATGACTGACG GTGTTGCACA TATCAAAAAG CATTTATGCC CTAGTGCCAT TTTATTATTT TATATCTTAA   
  
  
+ TTTTAGATAT TTTTATTTTT ATTTAATGAT TTATATTTGA ACGTTAATTT TCAATATATC ATATTGTATC   
  
  
+ TTTTATAGCC ACGTTTATTC AAATATATTT TTTATATTTA TAAATATTTT TTCTAAAATA TGATTCGAGA   
  
  
+ ATGCTGACAT GTGAATCGCA TGGGGGTAGG GCAAGCCATC TTGGACCCAT TAAACTTCAA ATTCCATGAT   
  
  
+ TGACTAGGAC TTGCCCAAAA ATTTCATTAC CCCAGATGCC CACATGTGAA TGCAGTCTCT GACAGAGTAG   
  
  
+ GTGGGAATAA ACCAAAATGC ATTTATTTAT TTTTCAAGAT TTTAATTATT ATTCACCTAA AATCCTTCAT   
  
  
+ AGGGATTAAA ATATTAGACC ATTGGATTTG CTCTCCTCAA TAGGGTTGAA CTTTGATTAG TACGTTTATA   
  
  
+ TTATTTTTTT CCTTTTTCGT CAAAATGCAT TCTGTCACAA AATTAATGTA GTCAAGGAAT TGACAAGTGA   
  
  
+ GAAGAGAGTC ACTTTAAATG ATTCAGCTGT CCACATTAAA GATAAGTCAC ATTTGTGGTC CATACTCCCC   
  
  
+ AAGACTTTAA TTTGAATTTA GCTCAGACTT TTCGTATTTT ATTTGTGTTT TTCTATTTAA GCTATCAATA   
  
  
+ CATTTATAAA TAAGTGATTT TAATATAAAA GATCAAGTAA AAAACAAATA AAGTACATAA AATATTTTTA   
  
  
+ TTCAATTACA TATTGTATCA AATTTTCAAG AGCAAAATTT ATAGCATTTT TCAATGTTTT GGAAATCAAA   
  
  
+ AAAGTAATAT ATTATCAGTT CACTAGTTAA ATTGGTGATT GAACCATAGA TGATGTCATA ATATATATTT   
  
  
+ TATATTTATT AAAATTAAAA ATATTTATAA GAAATTTTAA ATATATGAGA AATTAAAAGT CAATAATATT   
  
  
+ TTTTTCTTCA TCTTTAATAT TATCAAATGA ATATATATAT ATATATAAAT GTATTGATAT TTTAAAATTT   
  
  
+ TGTTAAATAA AATATATATA ATATGAAGAT ATTTATTGAA AGTTATTTAA TTTACCTATA TATATATTAA   
  
  
+ AAGTTATTTA TGGTTGTAAA AAATATTACA TTATTTTATT TATATTATTA ATATTACATT ATTTTATTTA   
  
  
+ TATTATTTAT AATTTCATTA TTATAAAAAG TTTGAAAATA TTATTGCTAT ATAATTTGTT GTTGTGTGAA   
  
  
+ GATCGGCAAA AAAACAGGAG TAGCAGTGTG GTGTCGTCCT TGCCCCTTGA ACTGGAGGTT GGTTTCCCAT   
  
  
+ TCCTCCCTCC CACGAATCCC ACATGTAAAG CAAATAAAAG AGCTTTACAT ACACATGATT AATGTATTTT   
  
  
+ TATTGTCAAT AAATTTTATA ATAAAACTTA TAAATCTTAT AAATTTAATA GGAAAAAAAA AAGTCTTCTA   
  
  
+ GAATTGATTT TATAATACAC CATACCAAGA TCACATGAAT TGCATGTGTC GGTGGACAAG GTTGCCAAAA   
  
  
+ TTCCTTATTC CTATACAGAA TCTTTTCATT GTTATATTAT CATAGTCGAA ACCCAGTAGT ATATAAAGAG   
  
  
+ GTTACGAGTG GAGGATGGAT ATGAAATAAA TAAGAGGCCT TAGAGATCAG GGTGTGAGAC ATGGATTTGG   
  
  
+ CAAATGGTGA GAGATCTAGT GAGC  

- TTAGTAATTT CAGATTAAGA AGTTTTAAGG CTTAAGATTT AAATCGATAA ATTTTAAGGT TAACAGGATA   
  
  
- GAGGCTTGAA TTAAAAGTCA AAGGGTACGA GGTTAAGGGC ATAAATGGGG CGATGAATAA TAATAGTAAT   
  
  
- AGTAATAGTG GAATAAGTAA TAAAGATATT TAAGGCGGAA GCTTACTAAA GATTCTAGAG GTTAAAAATA   
  
  
- TGTAGTTGAA AGTATTAAAG ATGCAGAGTT TATTGTATGC TAAGACTAAG GAAAGTTTTA AATTGAGGGT   
  
  
- ATTAAGGTTA AAAGCATTGA ATTAAAAAGA ATTTTTAGGG CTTGAAAGTT TTAGGGTTAA AAGAAAGTTT   
  
  
- AAATTAAAGA TTTACAAGAG TAAGAATGTA GATTAAGGAT TCTTAGGTTA AAAGTTTATT AGAGATTCTA   
  
  
- AGGCTAAGAG TTTACTTAAA GTTTTTAGGT CAAAGGGAAG CTTGTTAAAA TTAGGGACAA ATCACTACGT   
  
  
- ACATTATACA AAACACGAGA TATACCACAT GATTGGGAGA GATAACTATC GCGTACCACC AAGCACCGGG   
  
  
- TCCATACATG GGTAAAAGAG AGATTAGCAA CAGATACGTA CAGAGCTAAG AATACACACG TGCTAGTAAG   
  
  
- TCCTATAAGT AACTAAATGA GTAGTTAACA GTGCCGTCGA AGTAAAATAA TCATCTCTGG GCTGAAATCC   
  
  
- CTGAATCTCC CCACGATGCC AGGAATGGCA TGGAAGGACT ATTCATTGGA CTGGGGGCTT GGGCTAGGCC   
  
  
- AAAAAGTGTC TGGTAGAAAA GGTTTTATTC CTCAGTGTGA ATCCCAAAAA GAAAGAATAA AACAAATGGG   
  
  
- AAATTTTTAT TTTGTTTTTA TTCACCGCTG AGGTTCAGTA AAAAGAATTA GTTATTTCTA GTAAAAAAGC   
  
  
- TTTTGCTTTT AGCTCGAGCG GTAGCTCACC CTTTGCGTAA CTCGGCTTTA CGCCCCAGGT ATCCATACAA   
  
  
- AAAATAAATA CTCTATTTTT CATTTATTAT AAAAAAAGCC CAAATTTTTT ATATAATAAC TAATGAACAT   
  
  
- TAATATACTC GGAGCAAACA TGAATAATAA TTTATGTATA AGTTCCATTT TAATAGGAAA TTTTTTAAGA   
  
  
- ATCCGGCTCC CTTGGAAGGA TAATTATATT CAGTAGGTCG GGTGGAATAC CCCCATTAAG AGGGGTATAT   
  
  
- GGTACCCAGG TTTAGCCGCC GGGCCAGTCA AGTTGCTGTC ATGCTACCGT GAGACTTGGG ACACGACGTA   
  
  
- GACGAGTGTT TTTCATGTGG TGTCAACAGA AAGTGAGAGA GACGGGTAAG TGATACGTTT GTGCAAATAT   
  
  
- TTTAACCAAT AAAATATTTG CAAAGAGAAA GTTTACCAAT AAAATATAAT CATTGTTTTT ATAATATTAT   
  
  
- TATTTTTTTA GTTATAATGC ATTAACGATT TTTACTTTAA TGTAGTACAA CATTCTATAA AGTAAACGGA   
  
  
- TAATTATTAA AAAATAATAA ATATTTTAAC CCAACTTAAA GTTATAAAAA ATTTTATTCA GAATAAATAA   
  
  
- ATTCACTTTA AAAAAAATAT CTATTTCAGT GTTTTTAGTA AAAAGATTTA TTAAATATTT TTCTATGAAA   
  
  
- AGGTTATTTT TAATTTAATT TTACGAAAAT ATTTATTTTA ACATTGTTAA GTAAGGTATC GTTTCCATTT   
  
  
- ATTTCGGTAA AAGAATAGTT AATTTAGTTC GAAGAGAAAA AATACTTTAA CAAAAATTAT TTAAGAAAAT   
  
  
- CATTGTTATT CTAAAACAAG TATAAAATTA TGTAGAGGAA CCGTTAACCC GTTTCTACCG ACCATCACTG   
  
  
- TACTTAGGTT CGTACTTAAT CAGGCCCGCA ACAATGAACG ATGTTCCCAA TACTCGTACC TTCCTTTTTA   
  
  
- TTAATTTTTT ATTTTTTTTA ATTTAATGAA GTTAGTGTTT CAACTTTTAT TATTTTAACA CAAACCTTCA   
  
  
- TAAACATGAA TTTCAAAAAA TCATTTTCAC AAAGGGATTT TCCACAAAAA AAATCTCTTT CGTCGATATT   
  
  
- TTACAAAAAA GTTTTTATAA AAAAATAAAT TTTTGTGAAA GAATTTAGTG AACAATATTC AGTGGAATCT   
  
  
- TACTGACTGC CACAACGTGT ATAGTTTTTC GTAAATACGG GATCACGGTA AAATAATAAA ATATAGAATT   
  
  
- AAAATCTATA AAAATAAAAA TAAATTACTA AATATAAACT TGCAATTAAA AGTTATATAG TATAACATAG   
  
  
- AAAATATCGG TGCAAATAAG TTTATATAAA AAATATAAAT ATTTATAAAA AAGATTTTAT ACTAAGCTCT   
  
  
- TACGACTGTA CACTTAGCGT ACCCCCATCC CGTTCGGTAG AACCTGGGTA ATTTGAAGTT TAAGGTACTA   
  
  
- ACTGATCCTG AACGGGTTTT TAAAGTAATG GGGTCTACGG GTGTACACTT ACGTCAGAGA CTGTCTCATC   
  
  
- CACCCTTATT TGGTTTTACG TAAATAAATA AAAAGTTCTA AAATTAATAA TAAGTGGATT TTAGGAAGTA   
  
  
- TCCCTAATTT TATAATCTGG TAACCTAAAC GAGAGGAGTT ATCCCAACTT GAAACTAATC ATGCAAATAT   
  
  
- AATAAAAAAA GGAAAAAGCA GTTTTACGTA AGACAGTGTT TTAATTACAT CAGTTCCTTA ACTGTTCACT   
  
  
- CTTCTCTCAG TGAAATTTAC TAAGTCGACA GGTGTAATTT CTATTCAGTG TAAACACCAG GTATGAGGGG   
  
  
- TTCTGAAATT AAACTTAAAT CGAGTCTGAA AAGCATAAAA TAAACACAAA AAGATAAATT CGATAGTTAT   
  
  
- GTAAATATTT ATTCACTAAA ATTATATTTT CTAGTTCATT TTTTGTTTAT TTCATGTATT TTATAAAAAT   
  
  
- AAGTTAATGT ATAACATAGT TTAAAAGTTC TCGTTTTAAA TATCGTAAAA AGTTACAAAA CCTTTAGTTT   
  
  
- TTTCATTATA TAATAGTCAA GTGATCAATT TAACCACTAA CTTGGTATCT ACTACAGTAT TATATATAAA   
  
  
- ATATAAATAA TTTTAATTTT TATAAATATT CTTTAAAATT TATATACTCT TTAATTTTCA GTTATTATAA   
  
  
- AAAAAGAAGT AGAAATTATA ATAGTTTACT TATATATATA TATATATTTA CATAACTATA AAATTTTAAA   
  
  
- ACAATTTATT TTATATATAT TATACTTCTA TAAATAACTT TCAATAAATT AAATGGATAT ATATATAATT   
  
  
- TTCAATAAAT ACCAACATTT TTTATAATGT AATAAAATAA ATATAATAAT TATAATGTAA TAAAATAAAT   
  
  
- ATAATAAATA TTAAAGTAAT AATATTTTTC AAACTTTTAT AATAACGATA TATTAAACAA CAACACACTT   
  
  
- CTAGCCGTTT TTTTGTCCTC ATCGTCACAC CACAGCAGGA ACGGGGAACT TGACCTCCAA CCAAAGGGTA   
  
  
- AGGAGGGAGG GTGCTTAGGG TGTACATTTC GTTTATTTTC TCGAAATGTA TGTGTACTAA TTACATAAAA   
  
  
- ATAACAGTTA TTTAAAATAT TATTTTGAAT ATTTAGAATA TTTAAATTAT CCTTTTTTTT TTCAGAAGAT   
  
  
- CTTAACTAAA ATATTATGTG GTATGGTTCT AGTGTACTTA ACGTACACAG CCACCTGTTC CAACGGTTTT   
  
  
- AAGGAATAAG GATATGTCTT AGAAAAGTAA CAATATAATA GTATCAGCTT TGGGTCATCA TATATTTCTC   
  
  
- CAATGCTCAC CTCCTACCTA TACTTTATTT ATTCTCCGGA ATCTCTAGTC CCACACTCTG TACCTAAACC   
  
  
- GTTTACCACT CTCTAGATCA CTCG

+     GATA-motif

| Site Name | Organism | Position | Strand | Matrix score. | sequence | function |
| --- | --- | --- | --- | --- | --- | --- |
| GATA-motif | Arabidopsis thaliana | 65 | - | 7 | GATAGGA | part of a light responsive element |

>PlantCARE\_32120   
+ AATCATTAAA GTCTAATTCT TCAAAATTCC GAATTCTAAA TTTAGCTATT TAAAATTCCA ATTGTCCTAT   
  
  
+ CTCCGAACTT AATTTTCAGT TTCCCATGCT CCAATTCCCG TATTTACCCC GCTACTTATT ATTATCATTA   
  
  
+ TCATTATCAC CTTATTCATT ATTTCTATAA ATTCCGCCTT CGAATGATTT CTAAGATCTC CAATTTTTAT   
  
  
+ ACATCAACTT TCATAATTTC TACGTCTCAA ATAACATACG ATTCTGATTC CTTTCAAAAT TTAACTCCCA   
  
  
+ TAATTCCAAT TTTCGTAACT TAATTTTTCT TAAAAATCCC GAACTTTCAA AATCCCAATT TTCTTTCAAA   
  
  
+ TTTAATTTCT AAATGTTCTC ATTCTTACAT CTAATTCCTA AGAATCCAAT TTTCAAATAA TCTCTAAGAT   
  
  
+ TCCGATTCTC AAATGAATTT CAAAAATCCA GTTTCCCTTC GAACAATTTT AATCCCTGTT TAGTGATGCA   
  
  
+ TGTAATATGT TTTGTGCTCT ATATGGTGTA CTAACCCTCT CTATTGATAG CGCATGGTGG TTCGTGGCCC   
  
  
+ AGGTATGTAC CCATTTTCTC TCTAATCGTT GTCTATGCAT GTCTCGATTC TTATGTGTGC ACGATCATTC   
  
  
+ AGGATATTCA TTGATTTACT CATCAATTGT CACGGCAGCT TCATTTTATT AGTAGAGACC CGACTTTAGG   
  
  
+ GACTTAGAGG GGTGCTACGG TCCTTACCGT ACCTTCCTGA TAAGTAACCT GACCCCCGAA CCCGATCCGG   
  
  
+ TTTTTCACAG ACCATCTTTT CCAAAATAAG GAGTCACACT TAGGGTTTTT CTTTCTTATT TTGTTTACCC   
  
  
+ TTTAAAAATA AAACAAAAAT AAGTGGCGAC TCCAAGTCAT TTTTCTTAAT CAATAAAGAT CATTTTTTCG   
  
  
+ AAAACGAAAA TCGAGCTCGC CATCGAGTGG GAAACGCATT GAGCCGAAAT GCGGGGTCCA TAGGTATGTT   
  
  
+ TTTTATTTAT GAGATAAAAA GTAAATAATA TTTTTTTCGG GTTTAAAAAA TATATTATTG ATTACTTGTA   
  
  
+ ATTATATGAG CCTCGTTTGT ACTTATTATT AAATACATAT TCAAGGTAAA ATTATCCTTT AAAAAATTCT   
  
  
+ TAGGCCGAGG GAACCTTCCT ATTAATATAA GTCATCCAGC CCACCTTATG GGGGTAATTC TCCCCATATA   
  
  
+ CCATGGGTCC AAATCGGCGG CCCGGTCAGT TCAACGACAG TACGATGGCA CTCTGAACCC TGTGCTGCAT   
  
  
+ CTGCTCACAA AAAGTACACC ACAGTTGTCT TTCACTCTCT CTGCCCATTC ACTATGCAAA CACGTTTATA   
  
  
+ AAATTGGTTA TTTTATAAAC GTTTCTCTTT CAAATGGTTA TTTTATATTA GTAACAAAAA TATTATAATA   
  
  
+ ATAAAAAAAT CAATATTACG TAATTGCTAA AAATGAAATT ACATCATGTT GTAAGATATT TCATTTGCCT   
  
  
+ ATTAATAATT TTTTATTATT TATAAAATTG GGTTGAATTT CAATATTTTT TAAAATAAGT CTTATTTATT   
  
  
+ TAAGTGAAAT TTTTTTTATA GATAAAGTCA CAAAAATCAT TTTTCTAAAT AATTTATAAA AAGATACTTT   
  
  
+ TCCAATAAAA ATTAAATTAA AATGCTTTTA TAAATAAAAT TGTAACAATT CATTCCATAG CAAAGGTAAA   
  
  
+ TAAAGCCATT TTCTTATCAA TTAAATCAAG CTTCTCTTTT TTATGAAATT GTTTTTAATA AATTCTTTTA   
  
  
+ GTAACAATAA GATTTTGTTC ATATTTTAAT ACATCTCCTT GGCAATTGGG CAAAGATGGC TGGTAGTGAC   
  
  
+ ATGAATCCAA GCATGAATTA GTCCGGGCGT TGTTACTTGC TACAAGGGTT ATGAGCATGG AAGGAAAAAT   
  
  
+ AATTAAAAAA TAAAAAAAAT TAAATTACTT CAATCACAAA GTTGAAAATA ATAAAATTGT GTTTGGAAGT   
  
  
+ ATTTGTACTT AAAGTTTTTT AGTAAAAGTG TTTCCCTAAA AGGTGTTTTT TTTAGAGAAA GCAGCTATAA   
  
  
+ AATGTTTTTT CAAAAATATT TTTTTATTTA AAAACACTTT CTTAAATCAC TTGTTATAAG TCACCTTAGA   
  
  
+ ATGACTGACG GTGTTGCACA TATCAAAAAG CATTTATGCC CTAGTGCCAT TTTATTATTT TATATCTTAA   
  
  
+ TTTTAGATAT TTTTATTTTT ATTTAATGAT TTATATTTGA ACGTTAATTT TCAATATATC ATATTGTATC   
  
  
+ TTTTATAGCC ACGTTTATTC AAATATATTT TTTATATTTA TAAATATTTT TTCTAAAATA TGATTCGAGA   
  
  
+ ATGCTGACAT GTGAATCGCA TGGGGGTAGG GCAAGCCATC TTGGACCCAT TAAACTTCAA ATTCCATGAT   
  
  
+ TGACTAGGAC TTGCCCAAAA ATTTCATTAC CCCAGATGCC CACATGTGAA TGCAGTCTCT GACAGAGTAG   
  
  
+ GTGGGAATAA ACCAAAATGC ATTTATTTAT TTTTCAAGAT TTTAATTATT ATTCACCTAA AATCCTTCAT   
  
  
+ AGGGATTAAA ATATTAGACC ATTGGATTTG CTCTCCTCAA TAGGGTTGAA CTTTGATTAG TACGTTTATA   
  
  
+ TTATTTTTTT CCTTTTTCGT CAAAATGCAT TCTGTCACAA AATTAATGTA GTCAAGGAAT TGACAAGTGA   
  
  
+ GAAGAGAGTC ACTTTAAATG ATTCAGCTGT CCACATTAAA GATAAGTCAC ATTTGTGGTC CATACTCCCC   
  
  
+ AAGACTTTAA TTTGAATTTA GCTCAGACTT TTCGTATTTT ATTTGTGTTT TTCTATTTAA GCTATCAATA   
  
  
+ CATTTATAAA TAAGTGATTT TAATATAAAA GATCAAGTAA AAAACAAATA AAGTACATAA AATATTTTTA   
  
  
+ TTCAATTACA TATTGTATCA AATTTTCAAG AGCAAAATTT ATAGCATTTT TCAATGTTTT GGAAATCAAA   
  
  
+ AAAGTAATAT ATTATCAGTT CACTAGTTAA ATTGGTGATT GAACCATAGA TGATGTCATA ATATATATTT   
  
  
+ TATATTTATT AAAATTAAAA ATATTTATAA GAAATTTTAA ATATATGAGA AATTAAAAGT CAATAATATT   
  
  
+ TTTTTCTTCA TCTTTAATAT TATCAAATGA ATATATATAT ATATATAAAT GTATTGATAT TTTAAAATTT   
  
  
+ TGTTAAATAA AATATATATA ATATGAAGAT ATTTATTGAA AGTTATTTAA TTTACCTATA TATATATTAA   
  
  
+ AAGTTATTTA TGGTTGTAAA AAATATTACA TTATTTTATT TATATTATTA ATATTACATT ATTTTATTTA   
  
  
+ TATTATTTAT AATTTCATTA TTATAAAAAG TTTGAAAATA TTATTGCTAT ATAATTTGTT GTTGTGTGAA   
  
  
+ GATCGGCAAA AAAACAGGAG TAGCAGTGTG GTGTCGTCCT TGCCCCTTGA ACTGGAGGTT GGTTTCCCAT   
  
  
+ TCCTCCCTCC CACGAATCCC ACATGTAAAG CAAATAAAAG AGCTTTACAT ACACATGATT AATGTATTTT   
  
  
+ TATTGTCAAT AAATTTTATA ATAAAACTTA TAAATCTTAT AAATTTAATA GGAAAAAAAA AAGTCTTCTA   
  
  
+ GAATTGATTT TATAATACAC CATACCAAGA TCACATGAAT TGCATGTGTC GGTGGACAAG GTTGCCAAAA   
  
  
+ TTCCTTATTC CTATACAGAA TCTTTTCATT GTTATATTAT CATAGTCGAA ACCCAGTAGT ATATAAAGAG   
  
  
+ GTTACGAGTG GAGGATGGAT ATGAAATAAA TAAGAGGCCT TAGAGATCAG GGTGTGAGAC ATGGATTTGG   
  
  
+ CAAATGGTGA GAGATCTAGT GAGC  

- TTAGTAATTT CAGATTAAGA AGTTTTAAGG CTTAAGATTT AAATCGATAA ATTTTAAGGT TAACAGGATA   
  
  
- GAGGCTTGAA TTAAAAGTCA AAGGGTACGA GGTTAAGGGC ATAAATGGGG CGATGAATAA TAATAGTAAT   
  
  
- AGTAATAGTG GAATAAGTAA TAAAGATATT TAAGGCGGAA GCTTACTAAA GATTCTAGAG GTTAAAAATA   
  
  
- TGTAGTTGAA AGTATTAAAG ATGCAGAGTT TATTGTATGC TAAGACTAAG GAAAGTTTTA AATTGAGGGT   
  
  
- ATTAAGGTTA AAAGCATTGA ATTAAAAAGA ATTTTTAGGG CTTGAAAGTT TTAGGGTTAA AAGAAAGTTT   
  
  
- AAATTAAAGA TTTACAAGAG TAAGAATGTA GATTAAGGAT TCTTAGGTTA AAAGTTTATT AGAGATTCTA   
  
  
- AGGCTAAGAG TTTACTTAAA GTTTTTAGGT CAAAGGGAAG CTTGTTAAAA TTAGGGACAA ATCACTACGT   
  
  
- ACATTATACA AAACACGAGA TATACCACAT GATTGGGAGA GATAACTATC GCGTACCACC AAGCACCGGG   
  
  
- TCCATACATG GGTAAAAGAG AGATTAGCAA CAGATACGTA CAGAGCTAAG AATACACACG TGCTAGTAAG   
  
  
- TCCTATAAGT AACTAAATGA GTAGTTAACA GTGCCGTCGA AGTAAAATAA TCATCTCTGG GCTGAAATCC   
  
  
- CTGAATCTCC CCACGATGCC AGGAATGGCA TGGAAGGACT ATTCATTGGA CTGGGGGCTT GGGCTAGGCC   
  
  
- AAAAAGTGTC TGGTAGAAAA GGTTTTATTC CTCAGTGTGA ATCCCAAAAA GAAAGAATAA AACAAATGGG   
  
  
- AAATTTTTAT TTTGTTTTTA TTCACCGCTG AGGTTCAGTA AAAAGAATTA GTTATTTCTA GTAAAAAAGC   
  
  
- TTTTGCTTTT AGCTCGAGCG GTAGCTCACC CTTTGCGTAA CTCGGCTTTA CGCCCCAGGT ATCCATACAA   
  
  
- AAAATAAATA CTCTATTTTT CATTTATTAT AAAAAAAGCC CAAATTTTTT ATATAATAAC TAATGAACAT   
  
  
- TAATATACTC GGAGCAAACA TGAATAATAA TTTATGTATA AGTTCCATTT TAATAGGAAA TTTTTTAAGA   
  
  
- ATCCGGCTCC CTTGGAAGGA TAATTATATT CAGTAGGTCG GGTGGAATAC CCCCATTAAG AGGGGTATAT   
  
  
- GGTACCCAGG TTTAGCCGCC GGGCCAGTCA AGTTGCTGTC ATGCTACCGT GAGACTTGGG ACACGACGTA   
  
  
- GACGAGTGTT TTTCATGTGG TGTCAACAGA AAGTGAGAGA GACGGGTAAG TGATACGTTT GTGCAAATAT   
  
  
- TTTAACCAAT AAAATATTTG CAAAGAGAAA GTTTACCAAT AAAATATAAT CATTGTTTTT ATAATATTAT   
  
  
- TATTTTTTTA GTTATAATGC ATTAACGATT TTTACTTTAA TGTAGTACAA CATTCTATAA AGTAAACGGA   
  
  
- TAATTATTAA AAAATAATAA ATATTTTAAC CCAACTTAAA GTTATAAAAA ATTTTATTCA GAATAAATAA   
  
  
- ATTCACTTTA AAAAAAATAT CTATTTCAGT GTTTTTAGTA AAAAGATTTA TTAAATATTT TTCTATGAAA   
  
  
- AGGTTATTTT TAATTTAATT TTACGAAAAT ATTTATTTTA ACATTGTTAA GTAAGGTATC GTTTCCATTT   
  
  
- ATTTCGGTAA AAGAATAGTT AATTTAGTTC GAAGAGAAAA AATACTTTAA CAAAAATTAT TTAAGAAAAT   
  
  
- CATTGTTATT CTAAAACAAG TATAAAATTA TGTAGAGGAA CCGTTAACCC GTTTCTACCG ACCATCACTG   
  
  
- TACTTAGGTT CGTACTTAAT CAGGCCCGCA ACAATGAACG ATGTTCCCAA TACTCGTACC TTCCTTTTTA   
  
  
- TTAATTTTTT ATTTTTTTTA ATTTAATGAA GTTAGTGTTT CAACTTTTAT TATTTTAACA CAAACCTTCA   
  
  
- TAAACATGAA TTTCAAAAAA TCATTTTCAC AAAGGGATTT TCCACAAAAA AAATCTCTTT CGTCGATATT   
  
  
- TTACAAAAAA GTTTTTATAA AAAAATAAAT TTTTGTGAAA GAATTTAGTG AACAATATTC AGTGGAATCT   
  
  
- TACTGACTGC CACAACGTGT ATAGTTTTTC GTAAATACGG GATCACGGTA AAATAATAAA ATATAGAATT   
  
  
- AAAATCTATA AAAATAAAAA TAAATTACTA AATATAAACT TGCAATTAAA AGTTATATAG TATAACATAG   
  
  
- AAAATATCGG TGCAAATAAG TTTATATAAA AAATATAAAT ATTTATAAAA AAGATTTTAT ACTAAGCTCT   
  
  
- TACGACTGTA CACTTAGCGT ACCCCCATCC CGTTCGGTAG AACCTGGGTA ATTTGAAGTT TAAGGTACTA   
  
  
- ACTGATCCTG AACGGGTTTT TAAAGTAATG GGGTCTACGG GTGTACACTT ACGTCAGAGA CTGTCTCATC   
  
  
- CACCCTTATT TGGTTTTACG TAAATAAATA AAAAGTTCTA AAATTAATAA TAAGTGGATT TTAGGAAGTA   
  
  
- TCCCTAATTT TATAATCTGG TAACCTAAAC GAGAGGAGTT ATCCCAACTT GAAACTAATC ATGCAAATAT   
  
  
- AATAAAAAAA GGAAAAAGCA GTTTTACGTA AGACAGTGTT TTAATTACAT CAGTTCCTTA ACTGTTCACT   
  
  
- CTTCTCTCAG TGAAATTTAC TAAGTCGACA GGTGTAATTT CTATTCAGTG TAAACACCAG GTATGAGGGG   
  
  
- TTCTGAAATT AAACTTAAAT CGAGTCTGAA AAGCATAAAA TAAACACAAA AAGATAAATT CGATAGTTAT   
  
  
- GTAAATATTT ATTCACTAAA ATTATATTTT CTAGTTCATT TTTTGTTTAT TTCATGTATT TTATAAAAAT   
  
  
- AAGTTAATGT ATAACATAGT TTAAAAGTTC TCGTTTTAAA TATCGTAAAA AGTTACAAAA CCTTTAGTTT   
  
  
- TTTCATTATA TAATAGTCAA GTGATCAATT TAACCACTAA CTTGGTATCT ACTACAGTAT TATATATAAA   
  
  
- ATATAAATAA TTTTAATTTT TATAAATATT CTTTAAAATT TATATACTCT TTAATTTTCA GTTATTATAA   
  
  
- AAAAAGAAGT AGAAATTATA ATAGTTTACT TATATATATA TATATATTTA CATAACTATA AAATTTTAAA   
  
  
- ACAATTTATT TTATATATAT TATACTTCTA TAAATAACTT TCAATAAATT AAATGGATAT ATATATAATT   
  
  
- TTCAATAAAT ACCAACATTT TTTATAATGT AATAAAATAA ATATAATAAT TATAATGTAA TAAAATAAAT   
  
  
- ATAATAAATA TTAAAGTAAT AATATTTTTC AAACTTTTAT AATAACGATA TATTAAACAA CAACACACTT   
  
  
- CTAGCCGTTT TTTTGTCCTC ATCGTCACAC CACAGCAGGA ACGGGGAACT TGACCTCCAA CCAAAGGGTA   
  
  
- AGGAGGGAGG GTGCTTAGGG TGTACATTTC GTTTATTTTC TCGAAATGTA TGTGTACTAA TTACATAAAA   
  
  
- ATAACAGTTA TTTAAAATAT TATTTTGAAT ATTTAGAATA TTTAAATTAT CCTTTTTTTT TTCAGAAGAT   
  
  
- CTTAACTAAA ATATTATGTG GTATGGTTCT AGTGTACTTA ACGTACACAG CCACCTGTTC CAACGGTTTT   
  
  
- AAGGAATAAG GATATGTCTT AGAAAAGTAA CAATATAATA GTATCAGCTT TGGGTCATCA TATATTTCTC   
  
  
- CAATGCTCAC CTCCTACCTA TACTTTATTT ATTCTCCGGA ATCTCTAGTC CCACACTCTG TACCTAAACC   
  
  
- GTTTACCACT CTCTAGATCA CTCG

+     GC-motif

| Site Name | Organism | Position | Strand | Matrix score. | sequence | function |
| --- | --- | --- | --- | --- | --- | --- |
| GC-motif | Zea mays | 753 | + | 6 | CCCCCG | enhancer-like element involved in anoxic specific inducibility |

>PlantCARE\_32120   
+ AATCATTAAA GTCTAATTCT TCAAAATTCC GAATTCTAAA TTTAGCTATT TAAAATTCCA ATTGTCCTAT   
  
  
+ CTCCGAACTT AATTTTCAGT TTCCCATGCT CCAATTCCCG TATTTACCCC GCTACTTATT ATTATCATTA   
  
  
+ TCATTATCAC CTTATTCATT ATTTCTATAA ATTCCGCCTT CGAATGATTT CTAAGATCTC CAATTTTTAT   
  
  
+ ACATCAACTT TCATAATTTC TACGTCTCAA ATAACATACG ATTCTGATTC CTTTCAAAAT TTAACTCCCA   
  
  
+ TAATTCCAAT TTTCGTAACT TAATTTTTCT TAAAAATCCC GAACTTTCAA AATCCCAATT TTCTTTCAAA   
  
  
+ TTTAATTTCT AAATGTTCTC ATTCTTACAT CTAATTCCTA AGAATCCAAT TTTCAAATAA TCTCTAAGAT   
  
  
+ TCCGATTCTC AAATGAATTT CAAAAATCCA GTTTCCCTTC GAACAATTTT AATCCCTGTT TAGTGATGCA   
  
  
+ TGTAATATGT TTTGTGCTCT ATATGGTGTA CTAACCCTCT CTATTGATAG CGCATGGTGG TTCGTGGCCC   
  
  
+ AGGTATGTAC CCATTTTCTC TCTAATCGTT GTCTATGCAT GTCTCGATTC TTATGTGTGC ACGATCATTC   
  
  
+ AGGATATTCA TTGATTTACT CATCAATTGT CACGGCAGCT TCATTTTATT AGTAGAGACC CGACTTTAGG   
  
  
+ GACTTAGAGG GGTGCTACGG TCCTTACCGT ACCTTCCTGA TAAGTAACCT GACCCCCGAA CCCGATCCGG   
  
  
+ TTTTTCACAG ACCATCTTTT CCAAAATAAG GAGTCACACT TAGGGTTTTT CTTTCTTATT TTGTTTACCC   
  
  
+ TTTAAAAATA AAACAAAAAT AAGTGGCGAC TCCAAGTCAT TTTTCTTAAT CAATAAAGAT CATTTTTTCG   
  
  
+ AAAACGAAAA TCGAGCTCGC CATCGAGTGG GAAACGCATT GAGCCGAAAT GCGGGGTCCA TAGGTATGTT   
  
  
+ TTTTATTTAT GAGATAAAAA GTAAATAATA TTTTTTTCGG GTTTAAAAAA TATATTATTG ATTACTTGTA   
  
  
+ ATTATATGAG CCTCGTTTGT ACTTATTATT AAATACATAT TCAAGGTAAA ATTATCCTTT AAAAAATTCT   
  
  
+ TAGGCCGAGG GAACCTTCCT ATTAATATAA GTCATCCAGC CCACCTTATG GGGGTAATTC TCCCCATATA   
  
  
+ CCATGGGTCC AAATCGGCGG CCCGGTCAGT TCAACGACAG TACGATGGCA CTCTGAACCC TGTGCTGCAT   
  
  
+ CTGCTCACAA AAAGTACACC ACAGTTGTCT TTCACTCTCT CTGCCCATTC ACTATGCAAA CACGTTTATA   
  
  
+ AAATTGGTTA TTTTATAAAC GTTTCTCTTT CAAATGGTTA TTTTATATTA GTAACAAAAA TATTATAATA   
  
  
+ ATAAAAAAAT CAATATTACG TAATTGCTAA AAATGAAATT ACATCATGTT GTAAGATATT TCATTTGCCT   
  
  
+ ATTAATAATT TTTTATTATT TATAAAATTG GGTTGAATTT CAATATTTTT TAAAATAAGT CTTATTTATT   
  
  
+ TAAGTGAAAT TTTTTTTATA GATAAAGTCA CAAAAATCAT TTTTCTAAAT AATTTATAAA AAGATACTTT   
  
  
+ TCCAATAAAA ATTAAATTAA AATGCTTTTA TAAATAAAAT TGTAACAATT CATTCCATAG CAAAGGTAAA   
  
  
+ TAAAGCCATT TTCTTATCAA TTAAATCAAG CTTCTCTTTT TTATGAAATT GTTTTTAATA AATTCTTTTA   
  
  
+ GTAACAATAA GATTTTGTTC ATATTTTAAT ACATCTCCTT GGCAATTGGG CAAAGATGGC TGGTAGTGAC   
  
  
+ ATGAATCCAA GCATGAATTA GTCCGGGCGT TGTTACTTGC TACAAGGGTT ATGAGCATGG AAGGAAAAAT   
  
  
+ AATTAAAAAA TAAAAAAAAT TAAATTACTT CAATCACAAA GTTGAAAATA ATAAAATTGT GTTTGGAAGT   
  
  
+ ATTTGTACTT AAAGTTTTTT AGTAAAAGTG TTTCCCTAAA AGGTGTTTTT TTTAGAGAAA GCAGCTATAA   
  
  
+ AATGTTTTTT CAAAAATATT TTTTTATTTA AAAACACTTT CTTAAATCAC TTGTTATAAG TCACCTTAGA   
  
  
+ ATGACTGACG GTGTTGCACA TATCAAAAAG CATTTATGCC CTAGTGCCAT TTTATTATTT TATATCTTAA   
  
  
+ TTTTAGATAT TTTTATTTTT ATTTAATGAT TTATATTTGA ACGTTAATTT TCAATATATC ATATTGTATC   
  
  
+ TTTTATAGCC ACGTTTATTC AAATATATTT TTTATATTTA TAAATATTTT TTCTAAAATA TGATTCGAGA   
  
  
+ ATGCTGACAT GTGAATCGCA TGGGGGTAGG GCAAGCCATC TTGGACCCAT TAAACTTCAA ATTCCATGAT   
  
  
+ TGACTAGGAC TTGCCCAAAA ATTTCATTAC CCCAGATGCC CACATGTGAA TGCAGTCTCT GACAGAGTAG   
  
  
+ GTGGGAATAA ACCAAAATGC ATTTATTTAT TTTTCAAGAT TTTAATTATT ATTCACCTAA AATCCTTCAT   
  
  
+ AGGGATTAAA ATATTAGACC ATTGGATTTG CTCTCCTCAA TAGGGTTGAA CTTTGATTAG TACGTTTATA   
  
  
+ TTATTTTTTT CCTTTTTCGT CAAAATGCAT TCTGTCACAA AATTAATGTA GTCAAGGAAT TGACAAGTGA   
  
  
+ GAAGAGAGTC ACTTTAAATG ATTCAGCTGT CCACATTAAA GATAAGTCAC ATTTGTGGTC CATACTCCCC   
  
  
+ AAGACTTTAA TTTGAATTTA GCTCAGACTT TTCGTATTTT ATTTGTGTTT TTCTATTTAA GCTATCAATA   
  
  
+ CATTTATAAA TAAGTGATTT TAATATAAAA GATCAAGTAA AAAACAAATA AAGTACATAA AATATTTTTA   
  
  
+ TTCAATTACA TATTGTATCA AATTTTCAAG AGCAAAATTT ATAGCATTTT TCAATGTTTT GGAAATCAAA   
  
  
+ AAAGTAATAT ATTATCAGTT CACTAGTTAA ATTGGTGATT GAACCATAGA TGATGTCATA ATATATATTT   
  
  
+ TATATTTATT AAAATTAAAA ATATTTATAA GAAATTTTAA ATATATGAGA AATTAAAAGT CAATAATATT   
  
  
+ TTTTTCTTCA TCTTTAATAT TATCAAATGA ATATATATAT ATATATAAAT GTATTGATAT TTTAAAATTT   
  
  
+ TGTTAAATAA AATATATATA ATATGAAGAT ATTTATTGAA AGTTATTTAA TTTACCTATA TATATATTAA   
  
  
+ AAGTTATTTA TGGTTGTAAA AAATATTACA TTATTTTATT TATATTATTA ATATTACATT ATTTTATTTA   
  
  
+ TATTATTTAT AATTTCATTA TTATAAAAAG TTTGAAAATA TTATTGCTAT ATAATTTGTT GTTGTGTGAA   
  
  
+ GATCGGCAAA AAAACAGGAG TAGCAGTGTG GTGTCGTCCT TGCCCCTTGA ACTGGAGGTT GGTTTCCCAT   
  
  
+ TCCTCCCTCC CACGAATCCC ACATGTAAAG CAAATAAAAG AGCTTTACAT ACACATGATT AATGTATTTT   
  
  
+ TATTGTCAAT AAATTTTATA ATAAAACTTA TAAATCTTAT AAATTTAATA GGAAAAAAAA AAGTCTTCTA   
  
  
+ GAATTGATTT TATAATACAC CATACCAAGA TCACATGAAT TGCATGTGTC GGTGGACAAG GTTGCCAAAA   
  
  
+ TTCCTTATTC CTATACAGAA TCTTTTCATT GTTATATTAT CATAGTCGAA ACCCAGTAGT ATATAAAGAG   
  
  
+ GTTACGAGTG GAGGATGGAT ATGAAATAAA TAAGAGGCCT TAGAGATCAG GGTGTGAGAC ATGGATTTGG   
  
  
+ CAAATGGTGA GAGATCTAGT GAGC  

- TTAGTAATTT CAGATTAAGA AGTTTTAAGG CTTAAGATTT AAATCGATAA ATTTTAAGGT TAACAGGATA   
  
  
- GAGGCTTGAA TTAAAAGTCA AAGGGTACGA GGTTAAGGGC ATAAATGGGG CGATGAATAA TAATAGTAAT   
  
  
- AGTAATAGTG GAATAAGTAA TAAAGATATT TAAGGCGGAA GCTTACTAAA GATTCTAGAG GTTAAAAATA   
  
  
- TGTAGTTGAA AGTATTAAAG ATGCAGAGTT TATTGTATGC TAAGACTAAG GAAAGTTTTA AATTGAGGGT   
  
  
- ATTAAGGTTA AAAGCATTGA ATTAAAAAGA ATTTTTAGGG CTTGAAAGTT TTAGGGTTAA AAGAAAGTTT   
  
  
- AAATTAAAGA TTTACAAGAG TAAGAATGTA GATTAAGGAT TCTTAGGTTA AAAGTTTATT AGAGATTCTA   
  
  
- AGGCTAAGAG TTTACTTAAA GTTTTTAGGT CAAAGGGAAG CTTGTTAAAA TTAGGGACAA ATCACTACGT   
  
  
- ACATTATACA AAACACGAGA TATACCACAT GATTGGGAGA GATAACTATC GCGTACCACC AAGCACCGGG   
  
  
- TCCATACATG GGTAAAAGAG AGATTAGCAA CAGATACGTA CAGAGCTAAG AATACACACG TGCTAGTAAG   
  
  
- TCCTATAAGT AACTAAATGA GTAGTTAACA GTGCCGTCGA AGTAAAATAA TCATCTCTGG GCTGAAATCC   
  
  
- CTGAATCTCC CCACGATGCC AGGAATGGCA TGGAAGGACT ATTCATTGGA CTGGGGGCTT GGGCTAGGCC   
  
  
- AAAAAGTGTC TGGTAGAAAA GGTTTTATTC CTCAGTGTGA ATCCCAAAAA GAAAGAATAA AACAAATGGG   
  
  
- AAATTTTTAT TTTGTTTTTA TTCACCGCTG AGGTTCAGTA AAAAGAATTA GTTATTTCTA GTAAAAAAGC   
  
  
- TTTTGCTTTT AGCTCGAGCG GTAGCTCACC CTTTGCGTAA CTCGGCTTTA CGCCCCAGGT ATCCATACAA   
  
  
- AAAATAAATA CTCTATTTTT CATTTATTAT AAAAAAAGCC CAAATTTTTT ATATAATAAC TAATGAACAT   
  
  
- TAATATACTC GGAGCAAACA TGAATAATAA TTTATGTATA AGTTCCATTT TAATAGGAAA TTTTTTAAGA   
  
  
- ATCCGGCTCC CTTGGAAGGA TAATTATATT CAGTAGGTCG GGTGGAATAC CCCCATTAAG AGGGGTATAT   
  
  
- GGTACCCAGG TTTAGCCGCC GGGCCAGTCA AGTTGCTGTC ATGCTACCGT GAGACTTGGG ACACGACGTA   
  
  
- GACGAGTGTT TTTCATGTGG TGTCAACAGA AAGTGAGAGA GACGGGTAAG TGATACGTTT GTGCAAATAT   
  
  
- TTTAACCAAT AAAATATTTG CAAAGAGAAA GTTTACCAAT AAAATATAAT CATTGTTTTT ATAATATTAT   
  
  
- TATTTTTTTA GTTATAATGC ATTAACGATT TTTACTTTAA TGTAGTACAA CATTCTATAA AGTAAACGGA   
  
  
- TAATTATTAA AAAATAATAA ATATTTTAAC CCAACTTAAA GTTATAAAAA ATTTTATTCA GAATAAATAA   
  
  
- ATTCACTTTA AAAAAAATAT CTATTTCAGT GTTTTTAGTA AAAAGATTTA TTAAATATTT TTCTATGAAA   
  
  
- AGGTTATTTT TAATTTAATT TTACGAAAAT ATTTATTTTA ACATTGTTAA GTAAGGTATC GTTTCCATTT   
  
  
- ATTTCGGTAA AAGAATAGTT AATTTAGTTC GAAGAGAAAA AATACTTTAA CAAAAATTAT TTAAGAAAAT   
  
  
- CATTGTTATT CTAAAACAAG TATAAAATTA TGTAGAGGAA CCGTTAACCC GTTTCTACCG ACCATCACTG   
  
  
- TACTTAGGTT CGTACTTAAT CAGGCCCGCA ACAATGAACG ATGTTCCCAA TACTCGTACC TTCCTTTTTA   
  
  
- TTAATTTTTT ATTTTTTTTA ATTTAATGAA GTTAGTGTTT CAACTTTTAT TATTTTAACA CAAACCTTCA   
  
  
- TAAACATGAA TTTCAAAAAA TCATTTTCAC AAAGGGATTT TCCACAAAAA AAATCTCTTT CGTCGATATT   
  
  
- TTACAAAAAA GTTTTTATAA AAAAATAAAT TTTTGTGAAA GAATTTAGTG AACAATATTC AGTGGAATCT   
  
  
- TACTGACTGC CACAACGTGT ATAGTTTTTC GTAAATACGG GATCACGGTA AAATAATAAA ATATAGAATT   
  
  
- AAAATCTATA AAAATAAAAA TAAATTACTA AATATAAACT TGCAATTAAA AGTTATATAG TATAACATAG   
  
  
- AAAATATCGG TGCAAATAAG TTTATATAAA AAATATAAAT ATTTATAAAA AAGATTTTAT ACTAAGCTCT   
  
  
- TACGACTGTA CACTTAGCGT ACCCCCATCC CGTTCGGTAG AACCTGGGTA ATTTGAAGTT TAAGGTACTA   
  
  
- ACTGATCCTG AACGGGTTTT TAAAGTAATG GGGTCTACGG GTGTACACTT ACGTCAGAGA CTGTCTCATC   
  
  
- CACCCTTATT TGGTTTTACG TAAATAAATA AAAAGTTCTA AAATTAATAA TAAGTGGATT TTAGGAAGTA   
  
  
- TCCCTAATTT TATAATCTGG TAACCTAAAC GAGAGGAGTT ATCCCAACTT GAAACTAATC ATGCAAATAT   
  
  
- AATAAAAAAA GGAAAAAGCA GTTTTACGTA AGACAGTGTT TTAATTACAT CAGTTCCTTA ACTGTTCACT   
  
  
- CTTCTCTCAG TGAAATTTAC TAAGTCGACA GGTGTAATTT CTATTCAGTG TAAACACCAG GTATGAGGGG   
  
  
- TTCTGAAATT AAACTTAAAT CGAGTCTGAA AAGCATAAAA TAAACACAAA AAGATAAATT CGATAGTTAT   
  
  
- GTAAATATTT ATTCACTAAA ATTATATTTT CTAGTTCATT TTTTGTTTAT TTCATGTATT TTATAAAAAT   
  
  
- AAGTTAATGT ATAACATAGT TTAAAAGTTC TCGTTTTAAA TATCGTAAAA AGTTACAAAA CCTTTAGTTT   
  
  
- TTTCATTATA TAATAGTCAA GTGATCAATT TAACCACTAA CTTGGTATCT ACTACAGTAT TATATATAAA   
  
  
- ATATAAATAA TTTTAATTTT TATAAATATT CTTTAAAATT TATATACTCT TTAATTTTCA GTTATTATAA   
  
  
- AAAAAGAAGT AGAAATTATA ATAGTTTACT TATATATATA TATATATTTA CATAACTATA AAATTTTAAA   
  
  
- ACAATTTATT TTATATATAT TATACTTCTA TAAATAACTT TCAATAAATT AAATGGATAT ATATATAATT   
  
  
- TTCAATAAAT ACCAACATTT TTTATAATGT AATAAAATAA ATATAATAAT TATAATGTAA TAAAATAAAT   
  
  
- ATAATAAATA TTAAAGTAAT AATATTTTTC AAACTTTTAT AATAACGATA TATTAAACAA CAACACACTT   
  
  
- CTAGCCGTTT TTTTGTCCTC ATCGTCACAC CACAGCAGGA ACGGGGAACT TGACCTCCAA CCAAAGGGTA   
  
  
- AGGAGGGAGG GTGCTTAGGG TGTACATTTC GTTTATTTTC TCGAAATGTA TGTGTACTAA TTACATAAAA   
  
  
- ATAACAGTTA TTTAAAATAT TATTTTGAAT ATTTAGAATA TTTAAATTAT CCTTTTTTTT TTCAGAAGAT   
  
  
- CTTAACTAAA ATATTATGTG GTATGGTTCT AGTGTACTTA ACGTACACAG CCACCTGTTC CAACGGTTTT   
  
  
- AAGGAATAAG GATATGTCTT AGAAAAGTAA CAATATAATA GTATCAGCTT TGGGTCATCA TATATTTCTC   
  
  
- CAATGCTCAC CTCCTACCTA TACTTTATTT ATTCTCCGGA ATCTCTAGTC CCACACTCTG TACCTAAACC   
  
  
- GTTTACCACT CTCTAGATCA CTCG

+     Gap-box

| Site Name | Organism | Position | Strand | Matrix score. | sequence | function |
| --- | --- | --- | --- | --- | --- | --- |
| Gap-box | Arabidopsis thaliana | 3104 | + | 9 | CAAATGAA(A/G)A | part of a light responsive element |

>PlantCARE\_32120   
+ AATCATTAAA GTCTAATTCT TCAAAATTCC GAATTCTAAA TTTAGCTATT TAAAATTCCA ATTGTCCTAT   
  
  
+ CTCCGAACTT AATTTTCAGT TTCCCATGCT CCAATTCCCG TATTTACCCC GCTACTTATT ATTATCATTA   
  
  
+ TCATTATCAC CTTATTCATT ATTTCTATAA ATTCCGCCTT CGAATGATTT CTAAGATCTC CAATTTTTAT   
  
  
+ ACATCAACTT TCATAATTTC TACGTCTCAA ATAACATACG ATTCTGATTC CTTTCAAAAT TTAACTCCCA   
  
  
+ TAATTCCAAT TTTCGTAACT TAATTTTTCT TAAAAATCCC GAACTTTCAA AATCCCAATT TTCTTTCAAA   
  
  
+ TTTAATTTCT AAATGTTCTC ATTCTTACAT CTAATTCCTA AGAATCCAAT TTTCAAATAA TCTCTAAGAT   
  
  
+ TCCGATTCTC AAATGAATTT CAAAAATCCA GTTTCCCTTC GAACAATTTT AATCCCTGTT TAGTGATGCA   
  
  
+ TGTAATATGT TTTGTGCTCT ATATGGTGTA CTAACCCTCT CTATTGATAG CGCATGGTGG TTCGTGGCCC   
  
  
+ AGGTATGTAC CCATTTTCTC TCTAATCGTT GTCTATGCAT GTCTCGATTC TTATGTGTGC ACGATCATTC   
  
  
+ AGGATATTCA TTGATTTACT CATCAATTGT CACGGCAGCT TCATTTTATT AGTAGAGACC CGACTTTAGG   
  
  
+ GACTTAGAGG GGTGCTACGG TCCTTACCGT ACCTTCCTGA TAAGTAACCT GACCCCCGAA CCCGATCCGG   
  
  
+ TTTTTCACAG ACCATCTTTT CCAAAATAAG GAGTCACACT TAGGGTTTTT CTTTCTTATT TTGTTTACCC   
  
  
+ TTTAAAAATA AAACAAAAAT AAGTGGCGAC TCCAAGTCAT TTTTCTTAAT CAATAAAGAT CATTTTTTCG   
  
  
+ AAAACGAAAA TCGAGCTCGC CATCGAGTGG GAAACGCATT GAGCCGAAAT GCGGGGTCCA TAGGTATGTT   
  
  
+ TTTTATTTAT GAGATAAAAA GTAAATAATA TTTTTTTCGG GTTTAAAAAA TATATTATTG ATTACTTGTA   
  
  
+ ATTATATGAG CCTCGTTTGT ACTTATTATT AAATACATAT TCAAGGTAAA ATTATCCTTT AAAAAATTCT   
  
  
+ TAGGCCGAGG GAACCTTCCT ATTAATATAA GTCATCCAGC CCACCTTATG GGGGTAATTC TCCCCATATA   
  
  
+ CCATGGGTCC AAATCGGCGG CCCGGTCAGT TCAACGACAG TACGATGGCA CTCTGAACCC TGTGCTGCAT   
  
  
+ CTGCTCACAA AAAGTACACC ACAGTTGTCT TTCACTCTCT CTGCCCATTC ACTATGCAAA CACGTTTATA   
  
  
+ AAATTGGTTA TTTTATAAAC GTTTCTCTTT CAAATGGTTA TTTTATATTA GTAACAAAAA TATTATAATA   
  
  
+ ATAAAAAAAT CAATATTACG TAATTGCTAA AAATGAAATT ACATCATGTT GTAAGATATT TCATTTGCCT   
  
  
+ ATTAATAATT TTTTATTATT TATAAAATTG GGTTGAATTT CAATATTTTT TAAAATAAGT CTTATTTATT   
  
  
+ TAAGTGAAAT TTTTTTTATA GATAAAGTCA CAAAAATCAT TTTTCTAAAT AATTTATAAA AAGATACTTT   
  
  
+ TCCAATAAAA ATTAAATTAA AATGCTTTTA TAAATAAAAT TGTAACAATT CATTCCATAG CAAAGGTAAA   
  
  
+ TAAAGCCATT TTCTTATCAA TTAAATCAAG CTTCTCTTTT TTATGAAATT GTTTTTAATA AATTCTTTTA   
  
  
+ GTAACAATAA GATTTTGTTC ATATTTTAAT ACATCTCCTT GGCAATTGGG CAAAGATGGC TGGTAGTGAC   
  
  
+ ATGAATCCAA GCATGAATTA GTCCGGGCGT TGTTACTTGC TACAAGGGTT ATGAGCATGG AAGGAAAAAT   
  
  
+ AATTAAAAAA TAAAAAAAAT TAAATTACTT CAATCACAAA GTTGAAAATA ATAAAATTGT GTTTGGAAGT   
  
  
+ ATTTGTACTT AAAGTTTTTT AGTAAAAGTG TTTCCCTAAA AGGTGTTTTT TTTAGAGAAA GCAGCTATAA   
  
  
+ AATGTTTTTT CAAAAATATT TTTTTATTTA AAAACACTTT CTTAAATCAC TTGTTATAAG TCACCTTAGA   
  
  
+ ATGACTGACG GTGTTGCACA TATCAAAAAG CATTTATGCC CTAGTGCCAT TTTATTATTT TATATCTTAA   
  
  
+ TTTTAGATAT TTTTATTTTT ATTTAATGAT TTATATTTGA ACGTTAATTT TCAATATATC ATATTGTATC   
  
  
+ TTTTATAGCC ACGTTTATTC AAATATATTT TTTATATTTA TAAATATTTT TTCTAAAATA TGATTCGAGA   
  
  
+ ATGCTGACAT GTGAATCGCA TGGGGGTAGG GCAAGCCATC TTGGACCCAT TAAACTTCAA ATTCCATGAT   
  
  
+ TGACTAGGAC TTGCCCAAAA ATTTCATTAC CCCAGATGCC CACATGTGAA TGCAGTCTCT GACAGAGTAG   
  
  
+ GTGGGAATAA ACCAAAATGC ATTTATTTAT TTTTCAAGAT TTTAATTATT ATTCACCTAA AATCCTTCAT   
  
  
+ AGGGATTAAA ATATTAGACC ATTGGATTTG CTCTCCTCAA TAGGGTTGAA CTTTGATTAG TACGTTTATA   
  
  
+ TTATTTTTTT CCTTTTTCGT CAAAATGCAT TCTGTCACAA AATTAATGTA GTCAAGGAAT TGACAAGTGA   
  
  
+ GAAGAGAGTC ACTTTAAATG ATTCAGCTGT CCACATTAAA GATAAGTCAC ATTTGTGGTC CATACTCCCC   
  
  
+ AAGACTTTAA TTTGAATTTA GCTCAGACTT TTCGTATTTT ATTTGTGTTT TTCTATTTAA GCTATCAATA   
  
  
+ CATTTATAAA TAAGTGATTT TAATATAAAA GATCAAGTAA AAAACAAATA AAGTACATAA AATATTTTTA   
  
  
+ TTCAATTACA TATTGTATCA AATTTTCAAG AGCAAAATTT ATAGCATTTT TCAATGTTTT GGAAATCAAA   
  
  
+ AAAGTAATAT ATTATCAGTT CACTAGTTAA ATTGGTGATT GAACCATAGA TGATGTCATA ATATATATTT   
  
  
+ TATATTTATT AAAATTAAAA ATATTTATAA GAAATTTTAA ATATATGAGA AATTAAAAGT CAATAATATT   
  
  
+ TTTTTCTTCA TCTTTAATAT TATCAAATGA ATATATATAT ATATATAAAT GTATTGATAT TTTAAAATTT   
  
  
+ TGTTAAATAA AATATATATA ATATGAAGAT ATTTATTGAA AGTTATTTAA TTTACCTATA TATATATTAA   
  
  
+ AAGTTATTTA TGGTTGTAAA AAATATTACA TTATTTTATT TATATTATTA ATATTACATT ATTTTATTTA   
  
  
+ TATTATTTAT AATTTCATTA TTATAAAAAG TTTGAAAATA TTATTGCTAT ATAATTTGTT GTTGTGTGAA   
  
  
+ GATCGGCAAA AAAACAGGAG TAGCAGTGTG GTGTCGTCCT TGCCCCTTGA ACTGGAGGTT GGTTTCCCAT   
  
  
+ TCCTCCCTCC CACGAATCCC ACATGTAAAG CAAATAAAAG AGCTTTACAT ACACATGATT AATGTATTTT   
  
  
+ TATTGTCAAT AAATTTTATA ATAAAACTTA TAAATCTTAT AAATTTAATA GGAAAAAAAA AAGTCTTCTA   
  
  
+ GAATTGATTT TATAATACAC CATACCAAGA TCACATGAAT TGCATGTGTC GGTGGACAAG GTTGCCAAAA   
  
  
+ TTCCTTATTC CTATACAGAA TCTTTTCATT GTTATATTAT CATAGTCGAA ACCCAGTAGT ATATAAAGAG   
  
  
+ GTTACGAGTG GAGGATGGAT ATGAAATAAA TAAGAGGCCT TAGAGATCAG GGTGTGAGAC ATGGATTTGG   
  
  
+ CAAATGGTGA GAGATCTAGT GAGC  

- TTAGTAATTT CAGATTAAGA AGTTTTAAGG CTTAAGATTT AAATCGATAA ATTTTAAGGT TAACAGGATA   
  
  
- GAGGCTTGAA TTAAAAGTCA AAGGGTACGA GGTTAAGGGC ATAAATGGGG CGATGAATAA TAATAGTAAT   
  
  
- AGTAATAGTG GAATAAGTAA TAAAGATATT TAAGGCGGAA GCTTACTAAA GATTCTAGAG GTTAAAAATA   
  
  
- TGTAGTTGAA AGTATTAAAG ATGCAGAGTT TATTGTATGC TAAGACTAAG GAAAGTTTTA AATTGAGGGT   
  
  
- ATTAAGGTTA AAAGCATTGA ATTAAAAAGA ATTTTTAGGG CTTGAAAGTT TTAGGGTTAA AAGAAAGTTT   
  
  
- AAATTAAAGA TTTACAAGAG TAAGAATGTA GATTAAGGAT TCTTAGGTTA AAAGTTTATT AGAGATTCTA   
  
  
- AGGCTAAGAG TTTACTTAAA GTTTTTAGGT CAAAGGGAAG CTTGTTAAAA TTAGGGACAA ATCACTACGT   
  
  
- ACATTATACA AAACACGAGA TATACCACAT GATTGGGAGA GATAACTATC GCGTACCACC AAGCACCGGG   
  
  
- TCCATACATG GGTAAAAGAG AGATTAGCAA CAGATACGTA CAGAGCTAAG AATACACACG TGCTAGTAAG   
  
  
- TCCTATAAGT AACTAAATGA GTAGTTAACA GTGCCGTCGA AGTAAAATAA TCATCTCTGG GCTGAAATCC   
  
  
- CTGAATCTCC CCACGATGCC AGGAATGGCA TGGAAGGACT ATTCATTGGA CTGGGGGCTT GGGCTAGGCC   
  
  
- AAAAAGTGTC TGGTAGAAAA GGTTTTATTC CTCAGTGTGA ATCCCAAAAA GAAAGAATAA AACAAATGGG   
  
  
- AAATTTTTAT TTTGTTTTTA TTCACCGCTG AGGTTCAGTA AAAAGAATTA GTTATTTCTA GTAAAAAAGC   
  
  
- TTTTGCTTTT AGCTCGAGCG GTAGCTCACC CTTTGCGTAA CTCGGCTTTA CGCCCCAGGT ATCCATACAA   
  
  
- AAAATAAATA CTCTATTTTT CATTTATTAT AAAAAAAGCC CAAATTTTTT ATATAATAAC TAATGAACAT   
  
  
- TAATATACTC GGAGCAAACA TGAATAATAA TTTATGTATA AGTTCCATTT TAATAGGAAA TTTTTTAAGA   
  
  
- ATCCGGCTCC CTTGGAAGGA TAATTATATT CAGTAGGTCG GGTGGAATAC CCCCATTAAG AGGGGTATAT   
  
  
- GGTACCCAGG TTTAGCCGCC GGGCCAGTCA AGTTGCTGTC ATGCTACCGT GAGACTTGGG ACACGACGTA   
  
  
- GACGAGTGTT TTTCATGTGG TGTCAACAGA AAGTGAGAGA GACGGGTAAG TGATACGTTT GTGCAAATAT   
  
  
- TTTAACCAAT AAAATATTTG CAAAGAGAAA GTTTACCAAT AAAATATAAT CATTGTTTTT ATAATATTAT   
  
  
- TATTTTTTTA GTTATAATGC ATTAACGATT TTTACTTTAA TGTAGTACAA CATTCTATAA AGTAAACGGA   
  
  
- TAATTATTAA AAAATAATAA ATATTTTAAC CCAACTTAAA GTTATAAAAA ATTTTATTCA GAATAAATAA   
  
  
- ATTCACTTTA AAAAAAATAT CTATTTCAGT GTTTTTAGTA AAAAGATTTA TTAAATATTT TTCTATGAAA   
  
  
- AGGTTATTTT TAATTTAATT TTACGAAAAT ATTTATTTTA ACATTGTTAA GTAAGGTATC GTTTCCATTT   
  
  
- ATTTCGGTAA AAGAATAGTT AATTTAGTTC GAAGAGAAAA AATACTTTAA CAAAAATTAT TTAAGAAAAT   
  
  
- CATTGTTATT CTAAAACAAG TATAAAATTA TGTAGAGGAA CCGTTAACCC GTTTCTACCG ACCATCACTG   
  
  
- TACTTAGGTT CGTACTTAAT CAGGCCCGCA ACAATGAACG ATGTTCCCAA TACTCGTACC TTCCTTTTTA   
  
  
- TTAATTTTTT ATTTTTTTTA ATTTAATGAA GTTAGTGTTT CAACTTTTAT TATTTTAACA CAAACCTTCA   
  
  
- TAAACATGAA TTTCAAAAAA TCATTTTCAC AAAGGGATTT TCCACAAAAA AAATCTCTTT CGTCGATATT   
  
  
- TTACAAAAAA GTTTTTATAA AAAAATAAAT TTTTGTGAAA GAATTTAGTG AACAATATTC AGTGGAATCT   
  
  
- TACTGACTGC CACAACGTGT ATAGTTTTTC GTAAATACGG GATCACGGTA AAATAATAAA ATATAGAATT   
  
  
- AAAATCTATA AAAATAAAAA TAAATTACTA AATATAAACT TGCAATTAAA AGTTATATAG TATAACATAG   
  
  
- AAAATATCGG TGCAAATAAG TTTATATAAA AAATATAAAT ATTTATAAAA AAGATTTTAT ACTAAGCTCT   
  
  
- TACGACTGTA CACTTAGCGT ACCCCCATCC CGTTCGGTAG AACCTGGGTA ATTTGAAGTT TAAGGTACTA   
  
  
- ACTGATCCTG AACGGGTTTT TAAAGTAATG GGGTCTACGG GTGTACACTT ACGTCAGAGA CTGTCTCATC   
  
  
- CACCCTTATT TGGTTTTACG TAAATAAATA AAAAGTTCTA AAATTAATAA TAAGTGGATT TTAGGAAGTA   
  
  
- TCCCTAATTT TATAATCTGG TAACCTAAAC GAGAGGAGTT ATCCCAACTT GAAACTAATC ATGCAAATAT   
  
  
- AATAAAAAAA GGAAAAAGCA GTTTTACGTA AGACAGTGTT TTAATTACAT CAGTTCCTTA ACTGTTCACT   
  
  
- CTTCTCTCAG TGAAATTTAC TAAGTCGACA GGTGTAATTT CTATTCAGTG TAAACACCAG GTATGAGGGG   
  
  
- TTCTGAAATT AAACTTAAAT CGAGTCTGAA AAGCATAAAA TAAACACAAA AAGATAAATT CGATAGTTAT   
  
  
- GTAAATATTT ATTCACTAAA ATTATATTTT CTAGTTCATT TTTTGTTTAT TTCATGTATT TTATAAAAAT   
  
  
- AAGTTAATGT ATAACATAGT TTAAAAGTTC TCGTTTTAAA TATCGTAAAA AGTTACAAAA CCTTTAGTTT   
  
  
- TTTCATTATA TAATAGTCAA GTGATCAATT TAACCACTAA CTTGGTATCT ACTACAGTAT TATATATAAA   
  
  
- ATATAAATAA TTTTAATTTT TATAAATATT CTTTAAAATT TATATACTCT TTAATTTTCA GTTATTATAA   
  
  
- AAAAAGAAGT AGAAATTATA ATAGTTTACT TATATATATA TATATATTTA CATAACTATA AAATTTTAAA   
  
  
- ACAATTTATT TTATATATAT TATACTTCTA TAAATAACTT TCAATAAATT AAATGGATAT ATATATAATT   
  
  
- TTCAATAAAT ACCAACATTT TTTATAATGT AATAAAATAA ATATAATAAT TATAATGTAA TAAAATAAAT   
  
  
- ATAATAAATA TTAAAGTAAT AATATTTTTC AAACTTTTAT AATAACGATA TATTAAACAA CAACACACTT   
  
  
- CTAGCCGTTT TTTTGTCCTC ATCGTCACAC CACAGCAGGA ACGGGGAACT TGACCTCCAA CCAAAGGGTA   
  
  
- AGGAGGGAGG GTGCTTAGGG TGTACATTTC GTTTATTTTC TCGAAATGTA TGTGTACTAA TTACATAAAA   
  
  
- ATAACAGTTA TTTAAAATAT TATTTTGAAT ATTTAGAATA TTTAAATTAT CCTTTTTTTT TTCAGAAGAT   
  
  
- CTTAACTAAA ATATTATGTG GTATGGTTCT AGTGTACTTA ACGTACACAG CCACCTGTTC CAACGGTTTT   
  
  
- AAGGAATAAG GATATGTCTT AGAAAAGTAA CAATATAATA GTATCAGCTT TGGGTCATCA TATATTTCTC   
  
  
- CAATGCTCAC CTCCTACCTA TACTTTATTT ATTCTCCGGA ATCTCTAGTC CCACACTCTG TACCTAAACC   
  
  
- GTTTACCACT CTCTAGATCA CTCG

+     I-box

| Site Name | Organism | Position | Strand | Matrix score. | sequence | function |
| --- | --- | --- | --- | --- | --- | --- |
| I-box | Zea mays | 148 | - | 9 | gGATAAGGTG | part of a light responsive element |

>PlantCARE\_32120   
+ AATCATTAAA GTCTAATTCT TCAAAATTCC GAATTCTAAA TTTAGCTATT TAAAATTCCA ATTGTCCTAT   
  
  
+ CTCCGAACTT AATTTTCAGT TTCCCATGCT CCAATTCCCG TATTTACCCC GCTACTTATT ATTATCATTA   
  
  
+ TCATTATCAC CTTATTCATT ATTTCTATAA ATTCCGCCTT CGAATGATTT CTAAGATCTC CAATTTTTAT   
  
  
+ ACATCAACTT TCATAATTTC TACGTCTCAA ATAACATACG ATTCTGATTC CTTTCAAAAT TTAACTCCCA   
  
  
+ TAATTCCAAT TTTCGTAACT TAATTTTTCT TAAAAATCCC GAACTTTCAA AATCCCAATT TTCTTTCAAA   
  
  
+ TTTAATTTCT AAATGTTCTC ATTCTTACAT CTAATTCCTA AGAATCCAAT TTTCAAATAA TCTCTAAGAT   
  
  
+ TCCGATTCTC AAATGAATTT CAAAAATCCA GTTTCCCTTC GAACAATTTT AATCCCTGTT TAGTGATGCA   
  
  
+ TGTAATATGT TTTGTGCTCT ATATGGTGTA CTAACCCTCT CTATTGATAG CGCATGGTGG TTCGTGGCCC   
  
  
+ AGGTATGTAC CCATTTTCTC TCTAATCGTT GTCTATGCAT GTCTCGATTC TTATGTGTGC ACGATCATTC   
  
  
+ AGGATATTCA TTGATTTACT CATCAATTGT CACGGCAGCT TCATTTTATT AGTAGAGACC CGACTTTAGG   
  
  
+ GACTTAGAGG GGTGCTACGG TCCTTACCGT ACCTTCCTGA TAAGTAACCT GACCCCCGAA CCCGATCCGG   
  
  
+ TTTTTCACAG ACCATCTTTT CCAAAATAAG GAGTCACACT TAGGGTTTTT CTTTCTTATT TTGTTTACCC   
  
  
+ TTTAAAAATA AAACAAAAAT AAGTGGCGAC TCCAAGTCAT TTTTCTTAAT CAATAAAGAT CATTTTTTCG   
  
  
+ AAAACGAAAA TCGAGCTCGC CATCGAGTGG GAAACGCATT GAGCCGAAAT GCGGGGTCCA TAGGTATGTT   
  
  
+ TTTTATTTAT GAGATAAAAA GTAAATAATA TTTTTTTCGG GTTTAAAAAA TATATTATTG ATTACTTGTA   
  
  
+ ATTATATGAG CCTCGTTTGT ACTTATTATT AAATACATAT TCAAGGTAAA ATTATCCTTT AAAAAATTCT   
  
  
+ TAGGCCGAGG GAACCTTCCT ATTAATATAA GTCATCCAGC CCACCTTATG GGGGTAATTC TCCCCATATA   
  
  
+ CCATGGGTCC AAATCGGCGG CCCGGTCAGT TCAACGACAG TACGATGGCA CTCTGAACCC TGTGCTGCAT   
  
  
+ CTGCTCACAA AAAGTACACC ACAGTTGTCT TTCACTCTCT CTGCCCATTC ACTATGCAAA CACGTTTATA   
  
  
+ AAATTGGTTA TTTTATAAAC GTTTCTCTTT CAAATGGTTA TTTTATATTA GTAACAAAAA TATTATAATA   
  
  
+ ATAAAAAAAT CAATATTACG TAATTGCTAA AAATGAAATT ACATCATGTT GTAAGATATT TCATTTGCCT   
  
  
+ ATTAATAATT TTTTATTATT TATAAAATTG GGTTGAATTT CAATATTTTT TAAAATAAGT CTTATTTATT   
  
  
+ TAAGTGAAAT TTTTTTTATA GATAAAGTCA CAAAAATCAT TTTTCTAAAT AATTTATAAA AAGATACTTT   
  
  
+ TCCAATAAAA ATTAAATTAA AATGCTTTTA TAAATAAAAT TGTAACAATT CATTCCATAG CAAAGGTAAA   
  
  
+ TAAAGCCATT TTCTTATCAA TTAAATCAAG CTTCTCTTTT TTATGAAATT GTTTTTAATA AATTCTTTTA   
  
  
+ GTAACAATAA GATTTTGTTC ATATTTTAAT ACATCTCCTT GGCAATTGGG CAAAGATGGC TGGTAGTGAC   
  
  
+ ATGAATCCAA GCATGAATTA GTCCGGGCGT TGTTACTTGC TACAAGGGTT ATGAGCATGG AAGGAAAAAT   
  
  
+ AATTAAAAAA TAAAAAAAAT TAAATTACTT CAATCACAAA GTTGAAAATA ATAAAATTGT GTTTGGAAGT   
  
  
+ ATTTGTACTT AAAGTTTTTT AGTAAAAGTG TTTCCCTAAA AGGTGTTTTT TTTAGAGAAA GCAGCTATAA   
  
  
+ AATGTTTTTT CAAAAATATT TTTTTATTTA AAAACACTTT CTTAAATCAC TTGTTATAAG TCACCTTAGA   
  
  
+ ATGACTGACG GTGTTGCACA TATCAAAAAG CATTTATGCC CTAGTGCCAT TTTATTATTT TATATCTTAA   
  
  
+ TTTTAGATAT TTTTATTTTT ATTTAATGAT TTATATTTGA ACGTTAATTT TCAATATATC ATATTGTATC   
  
  
+ TTTTATAGCC ACGTTTATTC AAATATATTT TTTATATTTA TAAATATTTT TTCTAAAATA TGATTCGAGA   
  
  
+ ATGCTGACAT GTGAATCGCA TGGGGGTAGG GCAAGCCATC TTGGACCCAT TAAACTTCAA ATTCCATGAT   
  
  
+ TGACTAGGAC TTGCCCAAAA ATTTCATTAC CCCAGATGCC CACATGTGAA TGCAGTCTCT GACAGAGTAG   
  
  
+ GTGGGAATAA ACCAAAATGC ATTTATTTAT TTTTCAAGAT TTTAATTATT ATTCACCTAA AATCCTTCAT   
  
  
+ AGGGATTAAA ATATTAGACC ATTGGATTTG CTCTCCTCAA TAGGGTTGAA CTTTGATTAG TACGTTTATA   
  
  
+ TTATTTTTTT CCTTTTTCGT CAAAATGCAT TCTGTCACAA AATTAATGTA GTCAAGGAAT TGACAAGTGA   
  
  
+ GAAGAGAGTC ACTTTAAATG ATTCAGCTGT CCACATTAAA GATAAGTCAC ATTTGTGGTC CATACTCCCC   
  
  
+ AAGACTTTAA TTTGAATTTA GCTCAGACTT TTCGTATTTT ATTTGTGTTT TTCTATTTAA GCTATCAATA   
  
  
+ CATTTATAAA TAAGTGATTT TAATATAAAA GATCAAGTAA AAAACAAATA AAGTACATAA AATATTTTTA   
  
  
+ TTCAATTACA TATTGTATCA AATTTTCAAG AGCAAAATTT ATAGCATTTT TCAATGTTTT GGAAATCAAA   
  
  
+ AAAGTAATAT ATTATCAGTT CACTAGTTAA ATTGGTGATT GAACCATAGA TGATGTCATA ATATATATTT   
  
  
+ TATATTTATT AAAATTAAAA ATATTTATAA GAAATTTTAA ATATATGAGA AATTAAAAGT CAATAATATT   
  
  
+ TTTTTCTTCA TCTTTAATAT TATCAAATGA ATATATATAT ATATATAAAT GTATTGATAT TTTAAAATTT   
  
  
+ TGTTAAATAA AATATATATA ATATGAAGAT ATTTATTGAA AGTTATTTAA TTTACCTATA TATATATTAA   
  
  
+ AAGTTATTTA TGGTTGTAAA AAATATTACA TTATTTTATT TATATTATTA ATATTACATT ATTTTATTTA   
  
  
+ TATTATTTAT AATTTCATTA TTATAAAAAG TTTGAAAATA TTATTGCTAT ATAATTTGTT GTTGTGTGAA   
  
  
+ GATCGGCAAA AAAACAGGAG TAGCAGTGTG GTGTCGTCCT TGCCCCTTGA ACTGGAGGTT GGTTTCCCAT   
  
  
+ TCCTCCCTCC CACGAATCCC ACATGTAAAG CAAATAAAAG AGCTTTACAT ACACATGATT AATGTATTTT   
  
  
+ TATTGTCAAT AAATTTTATA ATAAAACTTA TAAATCTTAT AAATTTAATA GGAAAAAAAA AAGTCTTCTA   
  
  
+ GAATTGATTT TATAATACAC CATACCAAGA TCACATGAAT TGCATGTGTC GGTGGACAAG GTTGCCAAAA   
  
  
+ TTCCTTATTC CTATACAGAA TCTTTTCATT GTTATATTAT CATAGTCGAA ACCCAGTAGT ATATAAAGAG   
  
  
+ GTTACGAGTG GAGGATGGAT ATGAAATAAA TAAGAGGCCT TAGAGATCAG GGTGTGAGAC ATGGATTTGG   
  
  
+ CAAATGGTGA GAGATCTAGT GAGC  

- TTAGTAATTT CAGATTAAGA AGTTTTAAGG CTTAAGATTT AAATCGATAA ATTTTAAGGT TAACAGGATA   
  
  
- GAGGCTTGAA TTAAAAGTCA AAGGGTACGA GGTTAAGGGC ATAAATGGGG CGATGAATAA TAATAGTAAT   
  
  
- AGTAATAGTG GAATAAGTAA TAAAGATATT TAAGGCGGAA GCTTACTAAA GATTCTAGAG GTTAAAAATA   
  
  
- TGTAGTTGAA AGTATTAAAG ATGCAGAGTT TATTGTATGC TAAGACTAAG GAAAGTTTTA AATTGAGGGT   
  
  
- ATTAAGGTTA AAAGCATTGA ATTAAAAAGA ATTTTTAGGG CTTGAAAGTT TTAGGGTTAA AAGAAAGTTT   
  
  
- AAATTAAAGA TTTACAAGAG TAAGAATGTA GATTAAGGAT TCTTAGGTTA AAAGTTTATT AGAGATTCTA   
  
  
- AGGCTAAGAG TTTACTTAAA GTTTTTAGGT CAAAGGGAAG CTTGTTAAAA TTAGGGACAA ATCACTACGT   
  
  
- ACATTATACA AAACACGAGA TATACCACAT GATTGGGAGA GATAACTATC GCGTACCACC AAGCACCGGG   
  
  
- TCCATACATG GGTAAAAGAG AGATTAGCAA CAGATACGTA CAGAGCTAAG AATACACACG TGCTAGTAAG   
  
  
- TCCTATAAGT AACTAAATGA GTAGTTAACA GTGCCGTCGA AGTAAAATAA TCATCTCTGG GCTGAAATCC   
  
  
- CTGAATCTCC CCACGATGCC AGGAATGGCA TGGAAGGACT ATTCATTGGA CTGGGGGCTT GGGCTAGGCC   
  
  
- AAAAAGTGTC TGGTAGAAAA GGTTTTATTC CTCAGTGTGA ATCCCAAAAA GAAAGAATAA AACAAATGGG   
  
  
- AAATTTTTAT TTTGTTTTTA TTCACCGCTG AGGTTCAGTA AAAAGAATTA GTTATTTCTA GTAAAAAAGC   
  
  
- TTTTGCTTTT AGCTCGAGCG GTAGCTCACC CTTTGCGTAA CTCGGCTTTA CGCCCCAGGT ATCCATACAA   
  
  
- AAAATAAATA CTCTATTTTT CATTTATTAT AAAAAAAGCC CAAATTTTTT ATATAATAAC TAATGAACAT   
  
  
- TAATATACTC GGAGCAAACA TGAATAATAA TTTATGTATA AGTTCCATTT TAATAGGAAA TTTTTTAAGA   
  
  
- ATCCGGCTCC CTTGGAAGGA TAATTATATT CAGTAGGTCG GGTGGAATAC CCCCATTAAG AGGGGTATAT   
  
  
- GGTACCCAGG TTTAGCCGCC GGGCCAGTCA AGTTGCTGTC ATGCTACCGT GAGACTTGGG ACACGACGTA   
  
  
- GACGAGTGTT TTTCATGTGG TGTCAACAGA AAGTGAGAGA GACGGGTAAG TGATACGTTT GTGCAAATAT   
  
  
- TTTAACCAAT AAAATATTTG CAAAGAGAAA GTTTACCAAT AAAATATAAT CATTGTTTTT ATAATATTAT   
  
  
- TATTTTTTTA GTTATAATGC ATTAACGATT TTTACTTTAA TGTAGTACAA CATTCTATAA AGTAAACGGA   
  
  
- TAATTATTAA AAAATAATAA ATATTTTAAC CCAACTTAAA GTTATAAAAA ATTTTATTCA GAATAAATAA   
  
  
- ATTCACTTTA AAAAAAATAT CTATTTCAGT GTTTTTAGTA AAAAGATTTA TTAAATATTT TTCTATGAAA   
  
  
- AGGTTATTTT TAATTTAATT TTACGAAAAT ATTTATTTTA ACATTGTTAA GTAAGGTATC GTTTCCATTT   
  
  
- ATTTCGGTAA AAGAATAGTT AATTTAGTTC GAAGAGAAAA AATACTTTAA CAAAAATTAT TTAAGAAAAT   
  
  
- CATTGTTATT CTAAAACAAG TATAAAATTA TGTAGAGGAA CCGTTAACCC GTTTCTACCG ACCATCACTG   
  
  
- TACTTAGGTT CGTACTTAAT CAGGCCCGCA ACAATGAACG ATGTTCCCAA TACTCGTACC TTCCTTTTTA   
  
  
- TTAATTTTTT ATTTTTTTTA ATTTAATGAA GTTAGTGTTT CAACTTTTAT TATTTTAACA CAAACCTTCA   
  
  
- TAAACATGAA TTTCAAAAAA TCATTTTCAC AAAGGGATTT TCCACAAAAA AAATCTCTTT CGTCGATATT   
  
  
- TTACAAAAAA GTTTTTATAA AAAAATAAAT TTTTGTGAAA GAATTTAGTG AACAATATTC AGTGGAATCT   
  
  
- TACTGACTGC CACAACGTGT ATAGTTTTTC GTAAATACGG GATCACGGTA AAATAATAAA ATATAGAATT   
  
  
- AAAATCTATA AAAATAAAAA TAAATTACTA AATATAAACT TGCAATTAAA AGTTATATAG TATAACATAG   
  
  
- AAAATATCGG TGCAAATAAG TTTATATAAA AAATATAAAT ATTTATAAAA AAGATTTTAT ACTAAGCTCT   
  
  
- TACGACTGTA CACTTAGCGT ACCCCCATCC CGTTCGGTAG AACCTGGGTA ATTTGAAGTT TAAGGTACTA   
  
  
- ACTGATCCTG AACGGGTTTT TAAAGTAATG GGGTCTACGG GTGTACACTT ACGTCAGAGA CTGTCTCATC   
  
  
- CACCCTTATT TGGTTTTACG TAAATAAATA AAAAGTTCTA AAATTAATAA TAAGTGGATT TTAGGAAGTA   
  
  
- TCCCTAATTT TATAATCTGG TAACCTAAAC GAGAGGAGTT ATCCCAACTT GAAACTAATC ATGCAAATAT   
  
  
- AATAAAAAAA GGAAAAAGCA GTTTTACGTA AGACAGTGTT TTAATTACAT CAGTTCCTTA ACTGTTCACT   
  
  
- CTTCTCTCAG TGAAATTTAC TAAGTCGACA GGTGTAATTT CTATTCAGTG TAAACACCAG GTATGAGGGG   
  
  
- TTCTGAAATT AAACTTAAAT CGAGTCTGAA AAGCATAAAA TAAACACAAA AAGATAAATT CGATAGTTAT   
  
  
- GTAAATATTT ATTCACTAAA ATTATATTTT CTAGTTCATT TTTTGTTTAT TTCATGTATT TTATAAAAAT   
  
  
- AAGTTAATGT ATAACATAGT TTAAAAGTTC TCGTTTTAAA TATCGTAAAA AGTTACAAAA CCTTTAGTTT   
  
  
- TTTCATTATA TAATAGTCAA GTGATCAATT TAACCACTAA CTTGGTATCT ACTACAGTAT TATATATAAA   
  
  
- ATATAAATAA TTTTAATTTT TATAAATATT CTTTAAAATT TATATACTCT TTAATTTTCA GTTATTATAA   
  
  
- AAAAAGAAGT AGAAATTATA ATAGTTTACT TATATATATA TATATATTTA CATAACTATA AAATTTTAAA   
  
  
- ACAATTTATT TTATATATAT TATACTTCTA TAAATAACTT TCAATAAATT AAATGGATAT ATATATAATT   
  
  
- TTCAATAAAT ACCAACATTT TTTATAATGT AATAAAATAA ATATAATAAT TATAATGTAA TAAAATAAAT   
  
  
- ATAATAAATA TTAAAGTAAT AATATTTTTC AAACTTTTAT AATAACGATA TATTAAACAA CAACACACTT   
  
  
- CTAGCCGTTT TTTTGTCCTC ATCGTCACAC CACAGCAGGA ACGGGGAACT TGACCTCCAA CCAAAGGGTA   
  
  
- AGGAGGGAGG GTGCTTAGGG TGTACATTTC GTTTATTTTC TCGAAATGTA TGTGTACTAA TTACATAAAA   
  
  
- ATAACAGTTA TTTAAAATAT TATTTTGAAT ATTTAGAATA TTTAAATTAT CCTTTTTTTT TTCAGAAGAT   
  
  
- CTTAACTAAA ATATTATGTG GTATGGTTCT AGTGTACTTA ACGTACACAG CCACCTGTTC CAACGGTTTT   
  
  
- AAGGAATAAG GATATGTCTT AGAAAAGTAA CAATATAATA GTATCAGCTT TGGGTCATCA TATATTTCTC   
  
  
- CAATGCTCAC CTCCTACCTA TACTTTATTT ATTCTCCGGA ATCTCTAGTC CCACACTCTG TACCTAAACC   
  
  
- GTTTACCACT CTCTAGATCA CTCG

+     L-box

| Site Name | Organism | Position | Strand | Matrix score. | sequence | function |
| --- | --- | --- | --- | --- | --- | --- |
| L-box | Petroselinum crispum | 2447 | - | 10 | ATCCCACCTAC | part of a light responsive element |

>PlantCARE\_32120   
+ AATCATTAAA GTCTAATTCT TCAAAATTCC GAATTCTAAA TTTAGCTATT TAAAATTCCA ATTGTCCTAT   
  
  
+ CTCCGAACTT AATTTTCAGT TTCCCATGCT CCAATTCCCG TATTTACCCC GCTACTTATT ATTATCATTA   
  
  
+ TCATTATCAC CTTATTCATT ATTTCTATAA ATTCCGCCTT CGAATGATTT CTAAGATCTC CAATTTTTAT   
  
  
+ ACATCAACTT TCATAATTTC TACGTCTCAA ATAACATACG ATTCTGATTC CTTTCAAAAT TTAACTCCCA   
  
  
+ TAATTCCAAT TTTCGTAACT TAATTTTTCT TAAAAATCCC GAACTTTCAA AATCCCAATT TTCTTTCAAA   
  
  
+ TTTAATTTCT AAATGTTCTC ATTCTTACAT CTAATTCCTA AGAATCCAAT TTTCAAATAA TCTCTAAGAT   
  
  
+ TCCGATTCTC AAATGAATTT CAAAAATCCA GTTTCCCTTC GAACAATTTT AATCCCTGTT TAGTGATGCA   
  
  
+ TGTAATATGT TTTGTGCTCT ATATGGTGTA CTAACCCTCT CTATTGATAG CGCATGGTGG TTCGTGGCCC   
  
  
+ AGGTATGTAC CCATTTTCTC TCTAATCGTT GTCTATGCAT GTCTCGATTC TTATGTGTGC ACGATCATTC   
  
  
+ AGGATATTCA TTGATTTACT CATCAATTGT CACGGCAGCT TCATTTTATT AGTAGAGACC CGACTTTAGG   
  
  
+ GACTTAGAGG GGTGCTACGG TCCTTACCGT ACCTTCCTGA TAAGTAACCT GACCCCCGAA CCCGATCCGG   
  
  
+ TTTTTCACAG ACCATCTTTT CCAAAATAAG GAGTCACACT TAGGGTTTTT CTTTCTTATT TTGTTTACCC   
  
  
+ TTTAAAAATA AAACAAAAAT AAGTGGCGAC TCCAAGTCAT TTTTCTTAAT CAATAAAGAT CATTTTTTCG   
  
  
+ AAAACGAAAA TCGAGCTCGC CATCGAGTGG GAAACGCATT GAGCCGAAAT GCGGGGTCCA TAGGTATGTT   
  
  
+ TTTTATTTAT GAGATAAAAA GTAAATAATA TTTTTTTCGG GTTTAAAAAA TATATTATTG ATTACTTGTA   
  
  
+ ATTATATGAG CCTCGTTTGT ACTTATTATT AAATACATAT TCAAGGTAAA ATTATCCTTT AAAAAATTCT   
  
  
+ TAGGCCGAGG GAACCTTCCT ATTAATATAA GTCATCCAGC CCACCTTATG GGGGTAATTC TCCCCATATA   
  
  
+ CCATGGGTCC AAATCGGCGG CCCGGTCAGT TCAACGACAG TACGATGGCA CTCTGAACCC TGTGCTGCAT   
  
  
+ CTGCTCACAA AAAGTACACC ACAGTTGTCT TTCACTCTCT CTGCCCATTC ACTATGCAAA CACGTTTATA   
  
  
+ AAATTGGTTA TTTTATAAAC GTTTCTCTTT CAAATGGTTA TTTTATATTA GTAACAAAAA TATTATAATA   
  
  
+ ATAAAAAAAT CAATATTACG TAATTGCTAA AAATGAAATT ACATCATGTT GTAAGATATT TCATTTGCCT   
  
  
+ ATTAATAATT TTTTATTATT TATAAAATTG GGTTGAATTT CAATATTTTT TAAAATAAGT CTTATTTATT   
  
  
+ TAAGTGAAAT TTTTTTTATA GATAAAGTCA CAAAAATCAT TTTTCTAAAT AATTTATAAA AAGATACTTT   
  
  
+ TCCAATAAAA ATTAAATTAA AATGCTTTTA TAAATAAAAT TGTAACAATT CATTCCATAG CAAAGGTAAA   
  
  
+ TAAAGCCATT TTCTTATCAA TTAAATCAAG CTTCTCTTTT TTATGAAATT GTTTTTAATA AATTCTTTTA   
  
  
+ GTAACAATAA GATTTTGTTC ATATTTTAAT ACATCTCCTT GGCAATTGGG CAAAGATGGC TGGTAGTGAC   
  
  
+ ATGAATCCAA GCATGAATTA GTCCGGGCGT TGTTACTTGC TACAAGGGTT ATGAGCATGG AAGGAAAAAT   
  
  
+ AATTAAAAAA TAAAAAAAAT TAAATTACTT CAATCACAAA GTTGAAAATA ATAAAATTGT GTTTGGAAGT   
  
  
+ ATTTGTACTT AAAGTTTTTT AGTAAAAGTG TTTCCCTAAA AGGTGTTTTT TTTAGAGAAA GCAGCTATAA   
  
  
+ AATGTTTTTT CAAAAATATT TTTTTATTTA AAAACACTTT CTTAAATCAC TTGTTATAAG TCACCTTAGA   
  
  
+ ATGACTGACG GTGTTGCACA TATCAAAAAG CATTTATGCC CTAGTGCCAT TTTATTATTT TATATCTTAA   
  
  
+ TTTTAGATAT TTTTATTTTT ATTTAATGAT TTATATTTGA ACGTTAATTT TCAATATATC ATATTGTATC   
  
  
+ TTTTATAGCC ACGTTTATTC AAATATATTT TTTATATTTA TAAATATTTT TTCTAAAATA TGATTCGAGA   
  
  
+ ATGCTGACAT GTGAATCGCA TGGGGGTAGG GCAAGCCATC TTGGACCCAT TAAACTTCAA ATTCCATGAT   
  
  
+ TGACTAGGAC TTGCCCAAAA ATTTCATTAC CCCAGATGCC CACATGTGAA TGCAGTCTCT GACAGAGTAG   
  
  
+ GTGGGAATAA ACCAAAATGC ATTTATTTAT TTTTCAAGAT TTTAATTATT ATTCACCTAA AATCCTTCAT   
  
  
+ AGGGATTAAA ATATTAGACC ATTGGATTTG CTCTCCTCAA TAGGGTTGAA CTTTGATTAG TACGTTTATA   
  
  
+ TTATTTTTTT CCTTTTTCGT CAAAATGCAT TCTGTCACAA AATTAATGTA GTCAAGGAAT TGACAAGTGA   
  
  
+ GAAGAGAGTC ACTTTAAATG ATTCAGCTGT CCACATTAAA GATAAGTCAC ATTTGTGGTC CATACTCCCC   
  
  
+ AAGACTTTAA TTTGAATTTA GCTCAGACTT TTCGTATTTT ATTTGTGTTT TTCTATTTAA GCTATCAATA   
  
  
+ CATTTATAAA TAAGTGATTT TAATATAAAA GATCAAGTAA AAAACAAATA AAGTACATAA AATATTTTTA   
  
  
+ TTCAATTACA TATTGTATCA AATTTTCAAG AGCAAAATTT ATAGCATTTT TCAATGTTTT GGAAATCAAA   
  
  
+ AAAGTAATAT ATTATCAGTT CACTAGTTAA ATTGGTGATT GAACCATAGA TGATGTCATA ATATATATTT   
  
  
+ TATATTTATT AAAATTAAAA ATATTTATAA GAAATTTTAA ATATATGAGA AATTAAAAGT CAATAATATT   
  
  
+ TTTTTCTTCA TCTTTAATAT TATCAAATGA ATATATATAT ATATATAAAT GTATTGATAT TTTAAAATTT   
  
  
+ TGTTAAATAA AATATATATA ATATGAAGAT ATTTATTGAA AGTTATTTAA TTTACCTATA TATATATTAA   
  
  
+ AAGTTATTTA TGGTTGTAAA AAATATTACA TTATTTTATT TATATTATTA ATATTACATT ATTTTATTTA   
  
  
+ TATTATTTAT AATTTCATTA TTATAAAAAG TTTGAAAATA TTATTGCTAT ATAATTTGTT GTTGTGTGAA   
  
  
+ GATCGGCAAA AAAACAGGAG TAGCAGTGTG GTGTCGTCCT TGCCCCTTGA ACTGGAGGTT GGTTTCCCAT   
  
  
+ TCCTCCCTCC CACGAATCCC ACATGTAAAG CAAATAAAAG AGCTTTACAT ACACATGATT AATGTATTTT   
  
  
+ TATTGTCAAT AAATTTTATA ATAAAACTTA TAAATCTTAT AAATTTAATA GGAAAAAAAA AAGTCTTCTA   
  
  
+ GAATTGATTT TATAATACAC CATACCAAGA TCACATGAAT TGCATGTGTC GGTGGACAAG GTTGCCAAAA   
  
  
+ TTCCTTATTC CTATACAGAA TCTTTTCATT GTTATATTAT CATAGTCGAA ACCCAGTAGT ATATAAAGAG   
  
  
+ GTTACGAGTG GAGGATGGAT ATGAAATAAA TAAGAGGCCT TAGAGATCAG GGTGTGAGAC ATGGATTTGG   
  
  
+ CAAATGGTGA GAGATCTAGT GAGC  

- TTAGTAATTT CAGATTAAGA AGTTTTAAGG CTTAAGATTT AAATCGATAA ATTTTAAGGT TAACAGGATA   
  
  
- GAGGCTTGAA TTAAAAGTCA AAGGGTACGA GGTTAAGGGC ATAAATGGGG CGATGAATAA TAATAGTAAT   
  
  
- AGTAATAGTG GAATAAGTAA TAAAGATATT TAAGGCGGAA GCTTACTAAA GATTCTAGAG GTTAAAAATA   
  
  
- TGTAGTTGAA AGTATTAAAG ATGCAGAGTT TATTGTATGC TAAGACTAAG GAAAGTTTTA AATTGAGGGT   
  
  
- ATTAAGGTTA AAAGCATTGA ATTAAAAAGA ATTTTTAGGG CTTGAAAGTT TTAGGGTTAA AAGAAAGTTT   
  
  
- AAATTAAAGA TTTACAAGAG TAAGAATGTA GATTAAGGAT TCTTAGGTTA AAAGTTTATT AGAGATTCTA   
  
  
- AGGCTAAGAG TTTACTTAAA GTTTTTAGGT CAAAGGGAAG CTTGTTAAAA TTAGGGACAA ATCACTACGT   
  
  
- ACATTATACA AAACACGAGA TATACCACAT GATTGGGAGA GATAACTATC GCGTACCACC AAGCACCGGG   
  
  
- TCCATACATG GGTAAAAGAG AGATTAGCAA CAGATACGTA CAGAGCTAAG AATACACACG TGCTAGTAAG   
  
  
- TCCTATAAGT AACTAAATGA GTAGTTAACA GTGCCGTCGA AGTAAAATAA TCATCTCTGG GCTGAAATCC   
  
  
- CTGAATCTCC CCACGATGCC AGGAATGGCA TGGAAGGACT ATTCATTGGA CTGGGGGCTT GGGCTAGGCC   
  
  
- AAAAAGTGTC TGGTAGAAAA GGTTTTATTC CTCAGTGTGA ATCCCAAAAA GAAAGAATAA AACAAATGGG   
  
  
- AAATTTTTAT TTTGTTTTTA TTCACCGCTG AGGTTCAGTA AAAAGAATTA GTTATTTCTA GTAAAAAAGC   
  
  
- TTTTGCTTTT AGCTCGAGCG GTAGCTCACC CTTTGCGTAA CTCGGCTTTA CGCCCCAGGT ATCCATACAA   
  
  
- AAAATAAATA CTCTATTTTT CATTTATTAT AAAAAAAGCC CAAATTTTTT ATATAATAAC TAATGAACAT   
  
  
- TAATATACTC GGAGCAAACA TGAATAATAA TTTATGTATA AGTTCCATTT TAATAGGAAA TTTTTTAAGA   
  
  
- ATCCGGCTCC CTTGGAAGGA TAATTATATT CAGTAGGTCG GGTGGAATAC CCCCATTAAG AGGGGTATAT   
  
  
- GGTACCCAGG TTTAGCCGCC GGGCCAGTCA AGTTGCTGTC ATGCTACCGT GAGACTTGGG ACACGACGTA   
  
  
- GACGAGTGTT TTTCATGTGG TGTCAACAGA AAGTGAGAGA GACGGGTAAG TGATACGTTT GTGCAAATAT   
  
  
- TTTAACCAAT AAAATATTTG CAAAGAGAAA GTTTACCAAT AAAATATAAT CATTGTTTTT ATAATATTAT   
  
  
- TATTTTTTTA GTTATAATGC ATTAACGATT TTTACTTTAA TGTAGTACAA CATTCTATAA AGTAAACGGA   
  
  
- TAATTATTAA AAAATAATAA ATATTTTAAC CCAACTTAAA GTTATAAAAA ATTTTATTCA GAATAAATAA   
  
  
- ATTCACTTTA AAAAAAATAT CTATTTCAGT GTTTTTAGTA AAAAGATTTA TTAAATATTT TTCTATGAAA   
  
  
- AGGTTATTTT TAATTTAATT TTACGAAAAT ATTTATTTTA ACATTGTTAA GTAAGGTATC GTTTCCATTT   
  
  
- ATTTCGGTAA AAGAATAGTT AATTTAGTTC GAAGAGAAAA AATACTTTAA CAAAAATTAT TTAAGAAAAT   
  
  
- CATTGTTATT CTAAAACAAG TATAAAATTA TGTAGAGGAA CCGTTAACCC GTTTCTACCG ACCATCACTG   
  
  
- TACTTAGGTT CGTACTTAAT CAGGCCCGCA ACAATGAACG ATGTTCCCAA TACTCGTACC TTCCTTTTTA   
  
  
- TTAATTTTTT ATTTTTTTTA ATTTAATGAA GTTAGTGTTT CAACTTTTAT TATTTTAACA CAAACCTTCA   
  
  
- TAAACATGAA TTTCAAAAAA TCATTTTCAC AAAGGGATTT TCCACAAAAA AAATCTCTTT CGTCGATATT   
  
  
- TTACAAAAAA GTTTTTATAA AAAAATAAAT TTTTGTGAAA GAATTTAGTG AACAATATTC AGTGGAATCT   
  
  
- TACTGACTGC CACAACGTGT ATAGTTTTTC GTAAATACGG GATCACGGTA AAATAATAAA ATATAGAATT   
  
  
- AAAATCTATA AAAATAAAAA TAAATTACTA AATATAAACT TGCAATTAAA AGTTATATAG TATAACATAG   
  
  
- AAAATATCGG TGCAAATAAG TTTATATAAA AAATATAAAT ATTTATAAAA AAGATTTTAT ACTAAGCTCT   
  
  
- TACGACTGTA CACTTAGCGT ACCCCCATCC CGTTCGGTAG AACCTGGGTA ATTTGAAGTT TAAGGTACTA   
  
  
- ACTGATCCTG AACGGGTTTT TAAAGTAATG GGGTCTACGG GTGTACACTT ACGTCAGAGA CTGTCTCATC   
  
  
- CACCCTTATT TGGTTTTACG TAAATAAATA AAAAGTTCTA AAATTAATAA TAAGTGGATT TTAGGAAGTA   
  
  
- TCCCTAATTT TATAATCTGG TAACCTAAAC GAGAGGAGTT ATCCCAACTT GAAACTAATC ATGCAAATAT   
  
  
- AATAAAAAAA GGAAAAAGCA GTTTTACGTA AGACAGTGTT TTAATTACAT CAGTTCCTTA ACTGTTCACT   
  
  
- CTTCTCTCAG TGAAATTTAC TAAGTCGACA GGTGTAATTT CTATTCAGTG TAAACACCAG GTATGAGGGG   
  
  
- TTCTGAAATT AAACTTAAAT CGAGTCTGAA AAGCATAAAA TAAACACAAA AAGATAAATT CGATAGTTAT   
  
  
- GTAAATATTT ATTCACTAAA ATTATATTTT CTAGTTCATT TTTTGTTTAT TTCATGTATT TTATAAAAAT   
  
  
- AAGTTAATGT ATAACATAGT TTAAAAGTTC TCGTTTTAAA TATCGTAAAA AGTTACAAAA CCTTTAGTTT   
  
  
- TTTCATTATA TAATAGTCAA GTGATCAATT TAACCACTAA CTTGGTATCT ACTACAGTAT TATATATAAA   
  
  
- ATATAAATAA TTTTAATTTT TATAAATATT CTTTAAAATT TATATACTCT TTAATTTTCA GTTATTATAA   
  
  
- AAAAAGAAGT AGAAATTATA ATAGTTTACT TATATATATA TATATATTTA CATAACTATA AAATTTTAAA   
  
  
- ACAATTTATT TTATATATAT TATACTTCTA TAAATAACTT TCAATAAATT AAATGGATAT ATATATAATT   
  
  
- TTCAATAAAT ACCAACATTT TTTATAATGT AATAAAATAA ATATAATAAT TATAATGTAA TAAAATAAAT   
  
  
- ATAATAAATA TTAAAGTAAT AATATTTTTC AAACTTTTAT AATAACGATA TATTAAACAA CAACACACTT   
  
  
- CTAGCCGTTT TTTTGTCCTC ATCGTCACAC CACAGCAGGA ACGGGGAACT TGACCTCCAA CCAAAGGGTA   
  
  
- AGGAGGGAGG GTGCTTAGGG TGTACATTTC GTTTATTTTC TCGAAATGTA TGTGTACTAA TTACATAAAA   
  
  
- ATAACAGTTA TTTAAAATAT TATTTTGAAT ATTTAGAATA TTTAAATTAT CCTTTTTTTT TTCAGAAGAT   
  
  
- CTTAACTAAA ATATTATGTG GTATGGTTCT AGTGTACTTA ACGTACACAG CCACCTGTTC CAACGGTTTT   
  
  
- AAGGAATAAG GATATGTCTT AGAAAAGTAA CAATATAATA GTATCAGCTT TGGGTCATCA TATATTTCTC   
  
  
- CAATGCTCAC CTCCTACCTA TACTTTATTT ATTCTCCGGA ATCTCTAGTC CCACACTCTG TACCTAAACC   
  
  
- GTTTACCACT CTCTAGATCA CTCG

+     LTR

| Site Name | Organism | Position | Strand | Matrix score. | sequence | function |
| --- | --- | --- | --- | --- | --- | --- |
| LTR | Hordeum vulgare | 954 | + | 6 | CCGAAA | cis-acting element involved in low-temperature responsiveness |
| LTR | Hordeum vulgare | 1015 | - | 6 | CCGAAA | cis-acting element involved in low-temperature responsiveness |

>PlantCARE\_32120   
+ AATCATTAAA GTCTAATTCT TCAAAATTCC GAATTCTAAA TTTAGCTATT TAAAATTCCA ATTGTCCTAT   
  
  
+ CTCCGAACTT AATTTTCAGT TTCCCATGCT CCAATTCCCG TATTTACCCC GCTACTTATT ATTATCATTA   
  
  
+ TCATTATCAC CTTATTCATT ATTTCTATAA ATTCCGCCTT CGAATGATTT CTAAGATCTC CAATTTTTAT   
  
  
+ ACATCAACTT TCATAATTTC TACGTCTCAA ATAACATACG ATTCTGATTC CTTTCAAAAT TTAACTCCCA   
  
  
+ TAATTCCAAT TTTCGTAACT TAATTTTTCT TAAAAATCCC GAACTTTCAA AATCCCAATT TTCTTTCAAA   
  
  
+ TTTAATTTCT AAATGTTCTC ATTCTTACAT CTAATTCCTA AGAATCCAAT TTTCAAATAA TCTCTAAGAT   
  
  
+ TCCGATTCTC AAATGAATTT CAAAAATCCA GTTTCCCTTC GAACAATTTT AATCCCTGTT TAGTGATGCA   
  
  
+ TGTAATATGT TTTGTGCTCT ATATGGTGTA CTAACCCTCT CTATTGATAG CGCATGGTGG TTCGTGGCCC   
  
  
+ AGGTATGTAC CCATTTTCTC TCTAATCGTT GTCTATGCAT GTCTCGATTC TTATGTGTGC ACGATCATTC   
  
  
+ AGGATATTCA TTGATTTACT CATCAATTGT CACGGCAGCT TCATTTTATT AGTAGAGACC CGACTTTAGG   
  
  
+ GACTTAGAGG GGTGCTACGG TCCTTACCGT ACCTTCCTGA TAAGTAACCT GACCCCCGAA CCCGATCCGG   
  
  
+ TTTTTCACAG ACCATCTTTT CCAAAATAAG GAGTCACACT TAGGGTTTTT CTTTCTTATT TTGTTTACCC   
  
  
+ TTTAAAAATA AAACAAAAAT AAGTGGCGAC TCCAAGTCAT TTTTCTTAAT CAATAAAGAT CATTTTTTCG   
  
  
+ AAAACGAAAA TCGAGCTCGC CATCGAGTGG GAAACGCATT GAGCCGAAAT GCGGGGTCCA TAGGTATGTT   
  
  
+ TTTTATTTAT GAGATAAAAA GTAAATAATA TTTTTTTCGG GTTTAAAAAA TATATTATTG ATTACTTGTA   
  
  
+ ATTATATGAG CCTCGTTTGT ACTTATTATT AAATACATAT TCAAGGTAAA ATTATCCTTT AAAAAATTCT   
  
  
+ TAGGCCGAGG GAACCTTCCT ATTAATATAA GTCATCCAGC CCACCTTATG GGGGTAATTC TCCCCATATA   
  
  
+ CCATGGGTCC AAATCGGCGG CCCGGTCAGT TCAACGACAG TACGATGGCA CTCTGAACCC TGTGCTGCAT   
  
  
+ CTGCTCACAA AAAGTACACC ACAGTTGTCT TTCACTCTCT CTGCCCATTC ACTATGCAAA CACGTTTATA   
  
  
+ AAATTGGTTA TTTTATAAAC GTTTCTCTTT CAAATGGTTA TTTTATATTA GTAACAAAAA TATTATAATA   
  
  
+ ATAAAAAAAT CAATATTACG TAATTGCTAA AAATGAAATT ACATCATGTT GTAAGATATT TCATTTGCCT   
  
  
+ ATTAATAATT TTTTATTATT TATAAAATTG GGTTGAATTT CAATATTTTT TAAAATAAGT CTTATTTATT   
  
  
+ TAAGTGAAAT TTTTTTTATA GATAAAGTCA CAAAAATCAT TTTTCTAAAT AATTTATAAA AAGATACTTT   
  
  
+ TCCAATAAAA ATTAAATTAA AATGCTTTTA TAAATAAAAT TGTAACAATT CATTCCATAG CAAAGGTAAA   
  
  
+ TAAAGCCATT TTCTTATCAA TTAAATCAAG CTTCTCTTTT TTATGAAATT GTTTTTAATA AATTCTTTTA   
  
  
+ GTAACAATAA GATTTTGTTC ATATTTTAAT ACATCTCCTT GGCAATTGGG CAAAGATGGC TGGTAGTGAC   
  
  
+ ATGAATCCAA GCATGAATTA GTCCGGGCGT TGTTACTTGC TACAAGGGTT ATGAGCATGG AAGGAAAAAT   
  
  
+ AATTAAAAAA TAAAAAAAAT TAAATTACTT CAATCACAAA GTTGAAAATA ATAAAATTGT GTTTGGAAGT   
  
  
+ ATTTGTACTT AAAGTTTTTT AGTAAAAGTG TTTCCCTAAA AGGTGTTTTT TTTAGAGAAA GCAGCTATAA   
  
  
+ AATGTTTTTT CAAAAATATT TTTTTATTTA AAAACACTTT CTTAAATCAC TTGTTATAAG TCACCTTAGA   
  
  
+ ATGACTGACG GTGTTGCACA TATCAAAAAG CATTTATGCC CTAGTGCCAT TTTATTATTT TATATCTTAA   
  
  
+ TTTTAGATAT TTTTATTTTT ATTTAATGAT TTATATTTGA ACGTTAATTT TCAATATATC ATATTGTATC   
  
  
+ TTTTATAGCC ACGTTTATTC AAATATATTT TTTATATTTA TAAATATTTT TTCTAAAATA TGATTCGAGA   
  
  
+ ATGCTGACAT GTGAATCGCA TGGGGGTAGG GCAAGCCATC TTGGACCCAT TAAACTTCAA ATTCCATGAT   
  
  
+ TGACTAGGAC TTGCCCAAAA ATTTCATTAC CCCAGATGCC CACATGTGAA TGCAGTCTCT GACAGAGTAG   
  
  
+ GTGGGAATAA ACCAAAATGC ATTTATTTAT TTTTCAAGAT TTTAATTATT ATTCACCTAA AATCCTTCAT   
  
  
+ AGGGATTAAA ATATTAGACC ATTGGATTTG CTCTCCTCAA TAGGGTTGAA CTTTGATTAG TACGTTTATA   
  
  
+ TTATTTTTTT CCTTTTTCGT CAAAATGCAT TCTGTCACAA AATTAATGTA GTCAAGGAAT TGACAAGTGA   
  
  
+ GAAGAGAGTC ACTTTAAATG ATTCAGCTGT CCACATTAAA GATAAGTCAC ATTTGTGGTC CATACTCCCC   
  
  
+ AAGACTTTAA TTTGAATTTA GCTCAGACTT TTCGTATTTT ATTTGTGTTT TTCTATTTAA GCTATCAATA   
  
  
+ CATTTATAAA TAAGTGATTT TAATATAAAA GATCAAGTAA AAAACAAATA AAGTACATAA AATATTTTTA   
  
  
+ TTCAATTACA TATTGTATCA AATTTTCAAG AGCAAAATTT ATAGCATTTT TCAATGTTTT GGAAATCAAA   
  
  
+ AAAGTAATAT ATTATCAGTT CACTAGTTAA ATTGGTGATT GAACCATAGA TGATGTCATA ATATATATTT   
  
  
+ TATATTTATT AAAATTAAAA ATATTTATAA GAAATTTTAA ATATATGAGA AATTAAAAGT CAATAATATT   
  
  
+ TTTTTCTTCA TCTTTAATAT TATCAAATGA ATATATATAT ATATATAAAT GTATTGATAT TTTAAAATTT   
  
  
+ TGTTAAATAA AATATATATA ATATGAAGAT ATTTATTGAA AGTTATTTAA TTTACCTATA TATATATTAA   
  
  
+ AAGTTATTTA TGGTTGTAAA AAATATTACA TTATTTTATT TATATTATTA ATATTACATT ATTTTATTTA   
  
  
+ TATTATTTAT AATTTCATTA TTATAAAAAG TTTGAAAATA TTATTGCTAT ATAATTTGTT GTTGTGTGAA   
  
  
+ GATCGGCAAA AAAACAGGAG TAGCAGTGTG GTGTCGTCCT TGCCCCTTGA ACTGGAGGTT GGTTTCCCAT   
  
  
+ TCCTCCCTCC CACGAATCCC ACATGTAAAG CAAATAAAAG AGCTTTACAT ACACATGATT AATGTATTTT   
  
  
+ TATTGTCAAT AAATTTTATA ATAAAACTTA TAAATCTTAT AAATTTAATA GGAAAAAAAA AAGTCTTCTA   
  
  
+ GAATTGATTT TATAATACAC CATACCAAGA TCACATGAAT TGCATGTGTC GGTGGACAAG GTTGCCAAAA   
  
  
+ TTCCTTATTC CTATACAGAA TCTTTTCATT GTTATATTAT CATAGTCGAA ACCCAGTAGT ATATAAAGAG   
  
  
+ GTTACGAGTG GAGGATGGAT ATGAAATAAA TAAGAGGCCT TAGAGATCAG GGTGTGAGAC ATGGATTTGG   
  
  
+ CAAATGGTGA GAGATCTAGT GAGC  

- TTAGTAATTT CAGATTAAGA AGTTTTAAGG CTTAAGATTT AAATCGATAA ATTTTAAGGT TAACAGGATA   
  
  
- GAGGCTTGAA TTAAAAGTCA AAGGGTACGA GGTTAAGGGC ATAAATGGGG CGATGAATAA TAATAGTAAT   
  
  
- AGTAATAGTG GAATAAGTAA TAAAGATATT TAAGGCGGAA GCTTACTAAA GATTCTAGAG GTTAAAAATA   
  
  
- TGTAGTTGAA AGTATTAAAG ATGCAGAGTT TATTGTATGC TAAGACTAAG GAAAGTTTTA AATTGAGGGT   
  
  
- ATTAAGGTTA AAAGCATTGA ATTAAAAAGA ATTTTTAGGG CTTGAAAGTT TTAGGGTTAA AAGAAAGTTT   
  
  
- AAATTAAAGA TTTACAAGAG TAAGAATGTA GATTAAGGAT TCTTAGGTTA AAAGTTTATT AGAGATTCTA   
  
  
- AGGCTAAGAG TTTACTTAAA GTTTTTAGGT CAAAGGGAAG CTTGTTAAAA TTAGGGACAA ATCACTACGT   
  
  
- ACATTATACA AAACACGAGA TATACCACAT GATTGGGAGA GATAACTATC GCGTACCACC AAGCACCGGG   
  
  
- TCCATACATG GGTAAAAGAG AGATTAGCAA CAGATACGTA CAGAGCTAAG AATACACACG TGCTAGTAAG   
  
  
- TCCTATAAGT AACTAAATGA GTAGTTAACA GTGCCGTCGA AGTAAAATAA TCATCTCTGG GCTGAAATCC   
  
  
- CTGAATCTCC CCACGATGCC AGGAATGGCA TGGAAGGACT ATTCATTGGA CTGGGGGCTT GGGCTAGGCC   
  
  
- AAAAAGTGTC TGGTAGAAAA GGTTTTATTC CTCAGTGTGA ATCCCAAAAA GAAAGAATAA AACAAATGGG   
  
  
- AAATTTTTAT TTTGTTTTTA TTCACCGCTG AGGTTCAGTA AAAAGAATTA GTTATTTCTA GTAAAAAAGC   
  
  
- TTTTGCTTTT AGCTCGAGCG GTAGCTCACC CTTTGCGTAA CTCGGCTTTA CGCCCCAGGT ATCCATACAA   
  
  
- AAAATAAATA CTCTATTTTT CATTTATTAT AAAAAAAGCC CAAATTTTTT ATATAATAAC TAATGAACAT   
  
  
- TAATATACTC GGAGCAAACA TGAATAATAA TTTATGTATA AGTTCCATTT TAATAGGAAA TTTTTTAAGA   
  
  
- ATCCGGCTCC CTTGGAAGGA TAATTATATT CAGTAGGTCG GGTGGAATAC CCCCATTAAG AGGGGTATAT   
  
  
- GGTACCCAGG TTTAGCCGCC GGGCCAGTCA AGTTGCTGTC ATGCTACCGT GAGACTTGGG ACACGACGTA   
  
  
- GACGAGTGTT TTTCATGTGG TGTCAACAGA AAGTGAGAGA GACGGGTAAG TGATACGTTT GTGCAAATAT   
  
  
- TTTAACCAAT AAAATATTTG CAAAGAGAAA GTTTACCAAT AAAATATAAT CATTGTTTTT ATAATATTAT   
  
  
- TATTTTTTTA GTTATAATGC ATTAACGATT TTTACTTTAA TGTAGTACAA CATTCTATAA AGTAAACGGA   
  
  
- TAATTATTAA AAAATAATAA ATATTTTAAC CCAACTTAAA GTTATAAAAA ATTTTATTCA GAATAAATAA   
  
  
- ATTCACTTTA AAAAAAATAT CTATTTCAGT GTTTTTAGTA AAAAGATTTA TTAAATATTT TTCTATGAAA   
  
  
- AGGTTATTTT TAATTTAATT TTACGAAAAT ATTTATTTTA ACATTGTTAA GTAAGGTATC GTTTCCATTT   
  
  
- ATTTCGGTAA AAGAATAGTT AATTTAGTTC GAAGAGAAAA AATACTTTAA CAAAAATTAT TTAAGAAAAT   
  
  
- CATTGTTATT CTAAAACAAG TATAAAATTA TGTAGAGGAA CCGTTAACCC GTTTCTACCG ACCATCACTG   
  
  
- TACTTAGGTT CGTACTTAAT CAGGCCCGCA ACAATGAACG ATGTTCCCAA TACTCGTACC TTCCTTTTTA   
  
  
- TTAATTTTTT ATTTTTTTTA ATTTAATGAA GTTAGTGTTT CAACTTTTAT TATTTTAACA CAAACCTTCA   
  
  
- TAAACATGAA TTTCAAAAAA TCATTTTCAC AAAGGGATTT TCCACAAAAA AAATCTCTTT CGTCGATATT   
  
  
- TTACAAAAAA GTTTTTATAA AAAAATAAAT TTTTGTGAAA GAATTTAGTG AACAATATTC AGTGGAATCT   
  
  
- TACTGACTGC CACAACGTGT ATAGTTTTTC GTAAATACGG GATCACGGTA AAATAATAAA ATATAGAATT   
  
  
- AAAATCTATA AAAATAAAAA TAAATTACTA AATATAAACT TGCAATTAAA AGTTATATAG TATAACATAG   
  
  
- AAAATATCGG TGCAAATAAG TTTATATAAA AAATATAAAT ATTTATAAAA AAGATTTTAT ACTAAGCTCT   
  
  
- TACGACTGTA CACTTAGCGT ACCCCCATCC CGTTCGGTAG AACCTGGGTA ATTTGAAGTT TAAGGTACTA   
  
  
- ACTGATCCTG AACGGGTTTT TAAAGTAATG GGGTCTACGG GTGTACACTT ACGTCAGAGA CTGTCTCATC   
  
  
- CACCCTTATT TGGTTTTACG TAAATAAATA AAAAGTTCTA AAATTAATAA TAAGTGGATT TTAGGAAGTA   
  
  
- TCCCTAATTT TATAATCTGG TAACCTAAAC GAGAGGAGTT ATCCCAACTT GAAACTAATC ATGCAAATAT   
  
  
- AATAAAAAAA GGAAAAAGCA GTTTTACGTA AGACAGTGTT TTAATTACAT CAGTTCCTTA ACTGTTCACT   
  
  
- CTTCTCTCAG TGAAATTTAC TAAGTCGACA GGTGTAATTT CTATTCAGTG TAAACACCAG GTATGAGGGG   
  
  
- TTCTGAAATT AAACTTAAAT CGAGTCTGAA AAGCATAAAA TAAACACAAA AAGATAAATT CGATAGTTAT   
  
  
- GTAAATATTT ATTCACTAAA ATTATATTTT CTAGTTCATT TTTTGTTTAT TTCATGTATT TTATAAAAAT   
  
  
- AAGTTAATGT ATAACATAGT TTAAAAGTTC TCGTTTTAAA TATCGTAAAA AGTTACAAAA CCTTTAGTTT   
  
  
- TTTCATTATA TAATAGTCAA GTGATCAATT TAACCACTAA CTTGGTATCT ACTACAGTAT TATATATAAA   
  
  
- ATATAAATAA TTTTAATTTT TATAAATATT CTTTAAAATT TATATACTCT TTAATTTTCA GTTATTATAA   
  
  
- AAAAAGAAGT AGAAATTATA ATAGTTTACT TATATATATA TATATATTTA CATAACTATA AAATTTTAAA   
  
  
- ACAATTTATT TTATATATAT TATACTTCTA TAAATAACTT TCAATAAATT AAATGGATAT ATATATAATT   
  
  
- TTCAATAAAT ACCAACATTT TTTATAATGT AATAAAATAA ATATAATAAT TATAATGTAA TAAAATAAAT   
  
  
- ATAATAAATA TTAAAGTAAT AATATTTTTC AAACTTTTAT AATAACGATA TATTAAACAA CAACACACTT   
  
  
- CTAGCCGTTT TTTTGTCCTC ATCGTCACAC CACAGCAGGA ACGGGGAACT TGACCTCCAA CCAAAGGGTA   
  
  
- AGGAGGGAGG GTGCTTAGGG TGTACATTTC GTTTATTTTC TCGAAATGTA TGTGTACTAA TTACATAAAA   
  
  
- ATAACAGTTA TTTAAAATAT TATTTTGAAT ATTTAGAATA TTTAAATTAT CCTTTTTTTT TTCAGAAGAT   
  
  
- CTTAACTAAA ATATTATGTG GTATGGTTCT AGTGTACTTA ACGTACACAG CCACCTGTTC CAACGGTTTT   
  
  
- AAGGAATAAG GATATGTCTT AGAAAAGTAA CAATATAATA GTATCAGCTT TGGGTCATCA TATATTTCTC   
  
  
- CAATGCTCAC CTCCTACCTA TACTTTATTT ATTCTCCGGA ATCTCTAGTC CCACACTCTG TACCTAAACC   
  
  
- GTTTACCACT CTCTAGATCA CTCG

+     MBS

| Site Name | Organism | Position | Strand | Matrix score. | sequence | function |
| --- | --- | --- | --- | --- | --- | --- |
| MBS | Arabidopsis thaliana | 1282 | - | 6 | CAACTG | MYB binding site involved in drought-inducibility |

>PlantCARE\_32120   
+ AATCATTAAA GTCTAATTCT TCAAAATTCC GAATTCTAAA TTTAGCTATT TAAAATTCCA ATTGTCCTAT   
  
  
+ CTCCGAACTT AATTTTCAGT TTCCCATGCT CCAATTCCCG TATTTACCCC GCTACTTATT ATTATCATTA   
  
  
+ TCATTATCAC CTTATTCATT ATTTCTATAA ATTCCGCCTT CGAATGATTT CTAAGATCTC CAATTTTTAT   
  
  
+ ACATCAACTT TCATAATTTC TACGTCTCAA ATAACATACG ATTCTGATTC CTTTCAAAAT TTAACTCCCA   
  
  
+ TAATTCCAAT TTTCGTAACT TAATTTTTCT TAAAAATCCC GAACTTTCAA AATCCCAATT TTCTTTCAAA   
  
  
+ TTTAATTTCT AAATGTTCTC ATTCTTACAT CTAATTCCTA AGAATCCAAT TTTCAAATAA TCTCTAAGAT   
  
  
+ TCCGATTCTC AAATGAATTT CAAAAATCCA GTTTCCCTTC GAACAATTTT AATCCCTGTT TAGTGATGCA   
  
  
+ TGTAATATGT TTTGTGCTCT ATATGGTGTA CTAACCCTCT CTATTGATAG CGCATGGTGG TTCGTGGCCC   
  
  
+ AGGTATGTAC CCATTTTCTC TCTAATCGTT GTCTATGCAT GTCTCGATTC TTATGTGTGC ACGATCATTC   
  
  
+ AGGATATTCA TTGATTTACT CATCAATTGT CACGGCAGCT TCATTTTATT AGTAGAGACC CGACTTTAGG   
  
  
+ GACTTAGAGG GGTGCTACGG TCCTTACCGT ACCTTCCTGA TAAGTAACCT GACCCCCGAA CCCGATCCGG   
  
  
+ TTTTTCACAG ACCATCTTTT CCAAAATAAG GAGTCACACT TAGGGTTTTT CTTTCTTATT TTGTTTACCC   
  
  
+ TTTAAAAATA AAACAAAAAT AAGTGGCGAC TCCAAGTCAT TTTTCTTAAT CAATAAAGAT CATTTTTTCG   
  
  
+ AAAACGAAAA TCGAGCTCGC CATCGAGTGG GAAACGCATT GAGCCGAAAT GCGGGGTCCA TAGGTATGTT   
  
  
+ TTTTATTTAT GAGATAAAAA GTAAATAATA TTTTTTTCGG GTTTAAAAAA TATATTATTG ATTACTTGTA   
  
  
+ ATTATATGAG CCTCGTTTGT ACTTATTATT AAATACATAT TCAAGGTAAA ATTATCCTTT AAAAAATTCT   
  
  
+ TAGGCCGAGG GAACCTTCCT ATTAATATAA GTCATCCAGC CCACCTTATG GGGGTAATTC TCCCCATATA   
  
  
+ CCATGGGTCC AAATCGGCGG CCCGGTCAGT TCAACGACAG TACGATGGCA CTCTGAACCC TGTGCTGCAT   
  
  
+ CTGCTCACAA AAAGTACACC ACAGTTGTCT TTCACTCTCT CTGCCCATTC ACTATGCAAA CACGTTTATA   
  
  
+ AAATTGGTTA TTTTATAAAC GTTTCTCTTT CAAATGGTTA TTTTATATTA GTAACAAAAA TATTATAATA   
  
  
+ ATAAAAAAAT CAATATTACG TAATTGCTAA AAATGAAATT ACATCATGTT GTAAGATATT TCATTTGCCT   
  
  
+ ATTAATAATT TTTTATTATT TATAAAATTG GGTTGAATTT CAATATTTTT TAAAATAAGT CTTATTTATT   
  
  
+ TAAGTGAAAT TTTTTTTATA GATAAAGTCA CAAAAATCAT TTTTCTAAAT AATTTATAAA AAGATACTTT   
  
  
+ TCCAATAAAA ATTAAATTAA AATGCTTTTA TAAATAAAAT TGTAACAATT CATTCCATAG CAAAGGTAAA   
  
  
+ TAAAGCCATT TTCTTATCAA TTAAATCAAG CTTCTCTTTT TTATGAAATT GTTTTTAATA AATTCTTTTA   
  
  
+ GTAACAATAA GATTTTGTTC ATATTTTAAT ACATCTCCTT GGCAATTGGG CAAAGATGGC TGGTAGTGAC   
  
  
+ ATGAATCCAA GCATGAATTA GTCCGGGCGT TGTTACTTGC TACAAGGGTT ATGAGCATGG AAGGAAAAAT   
  
  
+ AATTAAAAAA TAAAAAAAAT TAAATTACTT CAATCACAAA GTTGAAAATA ATAAAATTGT GTTTGGAAGT   
  
  
+ ATTTGTACTT AAAGTTTTTT AGTAAAAGTG TTTCCCTAAA AGGTGTTTTT TTTAGAGAAA GCAGCTATAA   
  
  
+ AATGTTTTTT CAAAAATATT TTTTTATTTA AAAACACTTT CTTAAATCAC TTGTTATAAG TCACCTTAGA   
  
  
+ ATGACTGACG GTGTTGCACA TATCAAAAAG CATTTATGCC CTAGTGCCAT TTTATTATTT TATATCTTAA   
  
  
+ TTTTAGATAT TTTTATTTTT ATTTAATGAT TTATATTTGA ACGTTAATTT TCAATATATC ATATTGTATC   
  
  
+ TTTTATAGCC ACGTTTATTC AAATATATTT TTTATATTTA TAAATATTTT TTCTAAAATA TGATTCGAGA   
  
  
+ ATGCTGACAT GTGAATCGCA TGGGGGTAGG GCAAGCCATC TTGGACCCAT TAAACTTCAA ATTCCATGAT   
  
  
+ TGACTAGGAC TTGCCCAAAA ATTTCATTAC CCCAGATGCC CACATGTGAA TGCAGTCTCT GACAGAGTAG   
  
  
+ GTGGGAATAA ACCAAAATGC ATTTATTTAT TTTTCAAGAT TTTAATTATT ATTCACCTAA AATCCTTCAT   
  
  
+ AGGGATTAAA ATATTAGACC ATTGGATTTG CTCTCCTCAA TAGGGTTGAA CTTTGATTAG TACGTTTATA   
  
  
+ TTATTTTTTT CCTTTTTCGT CAAAATGCAT TCTGTCACAA AATTAATGTA GTCAAGGAAT TGACAAGTGA   
  
  
+ GAAGAGAGTC ACTTTAAATG ATTCAGCTGT CCACATTAAA GATAAGTCAC ATTTGTGGTC CATACTCCCC   
  
  
+ AAGACTTTAA TTTGAATTTA GCTCAGACTT TTCGTATTTT ATTTGTGTTT TTCTATTTAA GCTATCAATA   
  
  
+ CATTTATAAA TAAGTGATTT TAATATAAAA GATCAAGTAA AAAACAAATA AAGTACATAA AATATTTTTA   
  
  
+ TTCAATTACA TATTGTATCA AATTTTCAAG AGCAAAATTT ATAGCATTTT TCAATGTTTT GGAAATCAAA   
  
  
+ AAAGTAATAT ATTATCAGTT CACTAGTTAA ATTGGTGATT GAACCATAGA TGATGTCATA ATATATATTT   
  
  
+ TATATTTATT AAAATTAAAA ATATTTATAA GAAATTTTAA ATATATGAGA AATTAAAAGT CAATAATATT   
  
  
+ TTTTTCTTCA TCTTTAATAT TATCAAATGA ATATATATAT ATATATAAAT GTATTGATAT TTTAAAATTT   
  
  
+ TGTTAAATAA AATATATATA ATATGAAGAT ATTTATTGAA AGTTATTTAA TTTACCTATA TATATATTAA   
  
  
+ AAGTTATTTA TGGTTGTAAA AAATATTACA TTATTTTATT TATATTATTA ATATTACATT ATTTTATTTA   
  
  
+ TATTATTTAT AATTTCATTA TTATAAAAAG TTTGAAAATA TTATTGCTAT ATAATTTGTT GTTGTGTGAA   
  
  
+ GATCGGCAAA AAAACAGGAG TAGCAGTGTG GTGTCGTCCT TGCCCCTTGA ACTGGAGGTT GGTTTCCCAT   
  
  
+ TCCTCCCTCC CACGAATCCC ACATGTAAAG CAAATAAAAG AGCTTTACAT ACACATGATT AATGTATTTT   
  
  
+ TATTGTCAAT AAATTTTATA ATAAAACTTA TAAATCTTAT AAATTTAATA GGAAAAAAAA AAGTCTTCTA   
  
  
+ GAATTGATTT TATAATACAC CATACCAAGA TCACATGAAT TGCATGTGTC GGTGGACAAG GTTGCCAAAA   
  
  
+ TTCCTTATTC CTATACAGAA TCTTTTCATT GTTATATTAT CATAGTCGAA ACCCAGTAGT ATATAAAGAG   
  
  
+ GTTACGAGTG GAGGATGGAT ATGAAATAAA TAAGAGGCCT TAGAGATCAG GGTGTGAGAC ATGGATTTGG   
  
  
+ CAAATGGTGA GAGATCTAGT GAGC  

- TTAGTAATTT CAGATTAAGA AGTTTTAAGG CTTAAGATTT AAATCGATAA ATTTTAAGGT TAACAGGATA   
  
  
- GAGGCTTGAA TTAAAAGTCA AAGGGTACGA GGTTAAGGGC ATAAATGGGG CGATGAATAA TAATAGTAAT   
  
  
- AGTAATAGTG GAATAAGTAA TAAAGATATT TAAGGCGGAA GCTTACTAAA GATTCTAGAG GTTAAAAATA   
  
  
- TGTAGTTGAA AGTATTAAAG ATGCAGAGTT TATTGTATGC TAAGACTAAG GAAAGTTTTA AATTGAGGGT   
  
  
- ATTAAGGTTA AAAGCATTGA ATTAAAAAGA ATTTTTAGGG CTTGAAAGTT TTAGGGTTAA AAGAAAGTTT   
  
  
- AAATTAAAGA TTTACAAGAG TAAGAATGTA GATTAAGGAT TCTTAGGTTA AAAGTTTATT AGAGATTCTA   
  
  
- AGGCTAAGAG TTTACTTAAA GTTTTTAGGT CAAAGGGAAG CTTGTTAAAA TTAGGGACAA ATCACTACGT   
  
  
- ACATTATACA AAACACGAGA TATACCACAT GATTGGGAGA GATAACTATC GCGTACCACC AAGCACCGGG   
  
  
- TCCATACATG GGTAAAAGAG AGATTAGCAA CAGATACGTA CAGAGCTAAG AATACACACG TGCTAGTAAG   
  
  
- TCCTATAAGT AACTAAATGA GTAGTTAACA GTGCCGTCGA AGTAAAATAA TCATCTCTGG GCTGAAATCC   
  
  
- CTGAATCTCC CCACGATGCC AGGAATGGCA TGGAAGGACT ATTCATTGGA CTGGGGGCTT GGGCTAGGCC   
  
  
- AAAAAGTGTC TGGTAGAAAA GGTTTTATTC CTCAGTGTGA ATCCCAAAAA GAAAGAATAA AACAAATGGG   
  
  
- AAATTTTTAT TTTGTTTTTA TTCACCGCTG AGGTTCAGTA AAAAGAATTA GTTATTTCTA GTAAAAAAGC   
  
  
- TTTTGCTTTT AGCTCGAGCG GTAGCTCACC CTTTGCGTAA CTCGGCTTTA CGCCCCAGGT ATCCATACAA   
  
  
- AAAATAAATA CTCTATTTTT CATTTATTAT AAAAAAAGCC CAAATTTTTT ATATAATAAC TAATGAACAT   
  
  
- TAATATACTC GGAGCAAACA TGAATAATAA TTTATGTATA AGTTCCATTT TAATAGGAAA TTTTTTAAGA   
  
  
- ATCCGGCTCC CTTGGAAGGA TAATTATATT CAGTAGGTCG GGTGGAATAC CCCCATTAAG AGGGGTATAT   
  
  
- GGTACCCAGG TTTAGCCGCC GGGCCAGTCA AGTTGCTGTC ATGCTACCGT GAGACTTGGG ACACGACGTA   
  
  
- GACGAGTGTT TTTCATGTGG TGTCAACAGA AAGTGAGAGA GACGGGTAAG TGATACGTTT GTGCAAATAT   
  
  
- TTTAACCAAT AAAATATTTG CAAAGAGAAA GTTTACCAAT AAAATATAAT CATTGTTTTT ATAATATTAT   
  
  
- TATTTTTTTA GTTATAATGC ATTAACGATT TTTACTTTAA TGTAGTACAA CATTCTATAA AGTAAACGGA   
  
  
- TAATTATTAA AAAATAATAA ATATTTTAAC CCAACTTAAA GTTATAAAAA ATTTTATTCA GAATAAATAA   
  
  
- ATTCACTTTA AAAAAAATAT CTATTTCAGT GTTTTTAGTA AAAAGATTTA TTAAATATTT TTCTATGAAA   
  
  
- AGGTTATTTT TAATTTAATT TTACGAAAAT ATTTATTTTA ACATTGTTAA GTAAGGTATC GTTTCCATTT   
  
  
- ATTTCGGTAA AAGAATAGTT AATTTAGTTC GAAGAGAAAA AATACTTTAA CAAAAATTAT TTAAGAAAAT   
  
  
- CATTGTTATT CTAAAACAAG TATAAAATTA TGTAGAGGAA CCGTTAACCC GTTTCTACCG ACCATCACTG   
  
  
- TACTTAGGTT CGTACTTAAT CAGGCCCGCA ACAATGAACG ATGTTCCCAA TACTCGTACC TTCCTTTTTA   
  
  
- TTAATTTTTT ATTTTTTTTA ATTTAATGAA GTTAGTGTTT CAACTTTTAT TATTTTAACA CAAACCTTCA   
  
  
- TAAACATGAA TTTCAAAAAA TCATTTTCAC AAAGGGATTT TCCACAAAAA AAATCTCTTT CGTCGATATT   
  
  
- TTACAAAAAA GTTTTTATAA AAAAATAAAT TTTTGTGAAA GAATTTAGTG AACAATATTC AGTGGAATCT   
  
  
- TACTGACTGC CACAACGTGT ATAGTTTTTC GTAAATACGG GATCACGGTA AAATAATAAA ATATAGAATT   
  
  
- AAAATCTATA AAAATAAAAA TAAATTACTA AATATAAACT TGCAATTAAA AGTTATATAG TATAACATAG   
  
  
- AAAATATCGG TGCAAATAAG TTTATATAAA AAATATAAAT ATTTATAAAA AAGATTTTAT ACTAAGCTCT   
  
  
- TACGACTGTA CACTTAGCGT ACCCCCATCC CGTTCGGTAG AACCTGGGTA ATTTGAAGTT TAAGGTACTA   
  
  
- ACTGATCCTG AACGGGTTTT TAAAGTAATG GGGTCTACGG GTGTACACTT ACGTCAGAGA CTGTCTCATC   
  
  
- CACCCTTATT TGGTTTTACG TAAATAAATA AAAAGTTCTA AAATTAATAA TAAGTGGATT TTAGGAAGTA   
  
  
- TCCCTAATTT TATAATCTGG TAACCTAAAC GAGAGGAGTT ATCCCAACTT GAAACTAATC ATGCAAATAT   
  
  
- AATAAAAAAA GGAAAAAGCA GTTTTACGTA AGACAGTGTT TTAATTACAT CAGTTCCTTA ACTGTTCACT   
  
  
- CTTCTCTCAG TGAAATTTAC TAAGTCGACA GGTGTAATTT CTATTCAGTG TAAACACCAG GTATGAGGGG   
  
  
- TTCTGAAATT AAACTTAAAT CGAGTCTGAA AAGCATAAAA TAAACACAAA AAGATAAATT CGATAGTTAT   
  
  
- GTAAATATTT ATTCACTAAA ATTATATTTT CTAGTTCATT TTTTGTTTAT TTCATGTATT TTATAAAAAT   
  
  
- AAGTTAATGT ATAACATAGT TTAAAAGTTC TCGTTTTAAA TATCGTAAAA AGTTACAAAA CCTTTAGTTT   
  
  
- TTTCATTATA TAATAGTCAA GTGATCAATT TAACCACTAA CTTGGTATCT ACTACAGTAT TATATATAAA   
  
  
- ATATAAATAA TTTTAATTTT TATAAATATT CTTTAAAATT TATATACTCT TTAATTTTCA GTTATTATAA   
  
  
- AAAAAGAAGT AGAAATTATA ATAGTTTACT TATATATATA TATATATTTA CATAACTATA AAATTTTAAA   
  
  
- ACAATTTATT TTATATATAT TATACTTCTA TAAATAACTT TCAATAAATT AAATGGATAT ATATATAATT   
  
  
- TTCAATAAAT ACCAACATTT TTTATAATGT AATAAAATAA ATATAATAAT TATAATGTAA TAAAATAAAT   
  
  
- ATAATAAATA TTAAAGTAAT AATATTTTTC AAACTTTTAT AATAACGATA TATTAAACAA CAACACACTT   
  
  
- CTAGCCGTTT TTTTGTCCTC ATCGTCACAC CACAGCAGGA ACGGGGAACT TGACCTCCAA CCAAAGGGTA   
  
  
- AGGAGGGAGG GTGCTTAGGG TGTACATTTC GTTTATTTTC TCGAAATGTA TGTGTACTAA TTACATAAAA   
  
  
- ATAACAGTTA TTTAAAATAT TATTTTGAAT ATTTAGAATA TTTAAATTAT CCTTTTTTTT TTCAGAAGAT   
  
  
- CTTAACTAAA ATATTATGTG GTATGGTTCT AGTGTACTTA ACGTACACAG CCACCTGTTC CAACGGTTTT   
  
  
- AAGGAATAAG GATATGTCTT AGAAAAGTAA CAATATAATA GTATCAGCTT TGGGTCATCA TATATTTCTC   
  
  
- CAATGCTCAC CTCCTACCTA TACTTTATTT ATTCTCCGGA ATCTCTAGTC CCACACTCTG TACCTAAACC   
  
  
- GTTTACCACT CTCTAGATCA CTCG

+     MYB

| Site Name | Organism | Position | Strand | Matrix score. | sequence | function |
| --- | --- | --- | --- | --- | --- | --- |
| MYB | Arabidopsis thaliana | 1335 | - | 6 | TAACCA |  |
| MYB | Arabidopsis thaliana | 3231 | - | 6 | CAACCA |  |
| MYB | Arabidopsis thaliana | 1365 | - | 6 | TAACCA |  |

>PlantCARE\_32120   
+ AATCATTAAA GTCTAATTCT TCAAAATTCC GAATTCTAAA TTTAGCTATT TAAAATTCCA ATTGTCCTAT   
  
  
+ CTCCGAACTT AATTTTCAGT TTCCCATGCT CCAATTCCCG TATTTACCCC GCTACTTATT ATTATCATTA   
  
  
+ TCATTATCAC CTTATTCATT ATTTCTATAA ATTCCGCCTT CGAATGATTT CTAAGATCTC CAATTTTTAT   
  
  
+ ACATCAACTT TCATAATTTC TACGTCTCAA ATAACATACG ATTCTGATTC CTTTCAAAAT TTAACTCCCA   
  
  
+ TAATTCCAAT TTTCGTAACT TAATTTTTCT TAAAAATCCC GAACTTTCAA AATCCCAATT TTCTTTCAAA   
  
  
+ TTTAATTTCT AAATGTTCTC ATTCTTACAT CTAATTCCTA AGAATCCAAT TTTCAAATAA TCTCTAAGAT   
  
  
+ TCCGATTCTC AAATGAATTT CAAAAATCCA GTTTCCCTTC GAACAATTTT AATCCCTGTT TAGTGATGCA   
  
  
+ TGTAATATGT TTTGTGCTCT ATATGGTGTA CTAACCCTCT CTATTGATAG CGCATGGTGG TTCGTGGCCC   
  
  
+ AGGTATGTAC CCATTTTCTC TCTAATCGTT GTCTATGCAT GTCTCGATTC TTATGTGTGC ACGATCATTC   
  
  
+ AGGATATTCA TTGATTTACT CATCAATTGT CACGGCAGCT TCATTTTATT AGTAGAGACC CGACTTTAGG   
  
  
+ GACTTAGAGG GGTGCTACGG TCCTTACCGT ACCTTCCTGA TAAGTAACCT GACCCCCGAA CCCGATCCGG   
  
  
+ TTTTTCACAG ACCATCTTTT CCAAAATAAG GAGTCACACT TAGGGTTTTT CTTTCTTATT TTGTTTACCC   
  
  
+ TTTAAAAATA AAACAAAAAT AAGTGGCGAC TCCAAGTCAT TTTTCTTAAT CAATAAAGAT CATTTTTTCG   
  
  
+ AAAACGAAAA TCGAGCTCGC CATCGAGTGG GAAACGCATT GAGCCGAAAT GCGGGGTCCA TAGGTATGTT   
  
  
+ TTTTATTTAT GAGATAAAAA GTAAATAATA TTTTTTTCGG GTTTAAAAAA TATATTATTG ATTACTTGTA   
  
  
+ ATTATATGAG CCTCGTTTGT ACTTATTATT AAATACATAT TCAAGGTAAA ATTATCCTTT AAAAAATTCT   
  
  
+ TAGGCCGAGG GAACCTTCCT ATTAATATAA GTCATCCAGC CCACCTTATG GGGGTAATTC TCCCCATATA   
  
  
+ CCATGGGTCC AAATCGGCGG CCCGGTCAGT TCAACGACAG TACGATGGCA CTCTGAACCC TGTGCTGCAT   
  
  
+ CTGCTCACAA AAAGTACACC ACAGTTGTCT TTCACTCTCT CTGCCCATTC ACTATGCAAA CACGTTTATA   
  
  
+ AAATTGGTTA TTTTATAAAC GTTTCTCTTT CAAATGGTTA TTTTATATTA GTAACAAAAA TATTATAATA   
  
  
+ ATAAAAAAAT CAATATTACG TAATTGCTAA AAATGAAATT ACATCATGTT GTAAGATATT TCATTTGCCT   
  
  
+ ATTAATAATT TTTTATTATT TATAAAATTG GGTTGAATTT CAATATTTTT TAAAATAAGT CTTATTTATT   
  
  
+ TAAGTGAAAT TTTTTTTATA GATAAAGTCA CAAAAATCAT TTTTCTAAAT AATTTATAAA AAGATACTTT   
  
  
+ TCCAATAAAA ATTAAATTAA AATGCTTTTA TAAATAAAAT TGTAACAATT CATTCCATAG CAAAGGTAAA   
  
  
+ TAAAGCCATT TTCTTATCAA TTAAATCAAG CTTCTCTTTT TTATGAAATT GTTTTTAATA AATTCTTTTA   
  
  
+ GTAACAATAA GATTTTGTTC ATATTTTAAT ACATCTCCTT GGCAATTGGG CAAAGATGGC TGGTAGTGAC   
  
  
+ ATGAATCCAA GCATGAATTA GTCCGGGCGT TGTTACTTGC TACAAGGGTT ATGAGCATGG AAGGAAAAAT   
  
  
+ AATTAAAAAA TAAAAAAAAT TAAATTACTT CAATCACAAA GTTGAAAATA ATAAAATTGT GTTTGGAAGT   
  
  
+ ATTTGTACTT AAAGTTTTTT AGTAAAAGTG TTTCCCTAAA AGGTGTTTTT TTTAGAGAAA GCAGCTATAA   
  
  
+ AATGTTTTTT CAAAAATATT TTTTTATTTA AAAACACTTT CTTAAATCAC TTGTTATAAG TCACCTTAGA   
  
  
+ ATGACTGACG GTGTTGCACA TATCAAAAAG CATTTATGCC CTAGTGCCAT TTTATTATTT TATATCTTAA   
  
  
+ TTTTAGATAT TTTTATTTTT ATTTAATGAT TTATATTTGA ACGTTAATTT TCAATATATC ATATTGTATC   
  
  
+ TTTTATAGCC ACGTTTATTC AAATATATTT TTTATATTTA TAAATATTTT TTCTAAAATA TGATTCGAGA   
  
  
+ ATGCTGACAT GTGAATCGCA TGGGGGTAGG GCAAGCCATC TTGGACCCAT TAAACTTCAA ATTCCATGAT   
  
  
+ TGACTAGGAC TTGCCCAAAA ATTTCATTAC CCCAGATGCC CACATGTGAA TGCAGTCTCT GACAGAGTAG   
  
  
+ GTGGGAATAA ACCAAAATGC ATTTATTTAT TTTTCAAGAT TTTAATTATT ATTCACCTAA AATCCTTCAT   
  
  
+ AGGGATTAAA ATATTAGACC ATTGGATTTG CTCTCCTCAA TAGGGTTGAA CTTTGATTAG TACGTTTATA   
  
  
+ TTATTTTTTT CCTTTTTCGT CAAAATGCAT TCTGTCACAA AATTAATGTA GTCAAGGAAT TGACAAGTGA   
  
  
+ GAAGAGAGTC ACTTTAAATG ATTCAGCTGT CCACATTAAA GATAAGTCAC ATTTGTGGTC CATACTCCCC   
  
  
+ AAGACTTTAA TTTGAATTTA GCTCAGACTT TTCGTATTTT ATTTGTGTTT TTCTATTTAA GCTATCAATA   
  
  
+ CATTTATAAA TAAGTGATTT TAATATAAAA GATCAAGTAA AAAACAAATA AAGTACATAA AATATTTTTA   
  
  
+ TTCAATTACA TATTGTATCA AATTTTCAAG AGCAAAATTT ATAGCATTTT TCAATGTTTT GGAAATCAAA   
  
  
+ AAAGTAATAT ATTATCAGTT CACTAGTTAA ATTGGTGATT GAACCATAGA TGATGTCATA ATATATATTT   
  
  
+ TATATTTATT AAAATTAAAA ATATTTATAA GAAATTTTAA ATATATGAGA AATTAAAAGT CAATAATATT   
  
  
+ TTTTTCTTCA TCTTTAATAT TATCAAATGA ATATATATAT ATATATAAAT GTATTGATAT TTTAAAATTT   
  
  
+ TGTTAAATAA AATATATATA ATATGAAGAT ATTTATTGAA AGTTATTTAA TTTACCTATA TATATATTAA   
  
  
+ AAGTTATTTA TGGTTGTAAA AAATATTACA TTATTTTATT TATATTATTA ATATTACATT ATTTTATTTA   
  
  
+ TATTATTTAT AATTTCATTA TTATAAAAAG TTTGAAAATA TTATTGCTAT ATAATTTGTT GTTGTGTGAA   
  
  
+ GATCGGCAAA AAAACAGGAG TAGCAGTGTG GTGTCGTCCT TGCCCCTTGA ACTGGAGGTT GGTTTCCCAT   
  
  
+ TCCTCCCTCC CACGAATCCC ACATGTAAAG CAAATAAAAG AGCTTTACAT ACACATGATT AATGTATTTT   
  
  
+ TATTGTCAAT AAATTTTATA ATAAAACTTA TAAATCTTAT AAATTTAATA GGAAAAAAAA AAGTCTTCTA   
  
  
+ GAATTGATTT TATAATACAC CATACCAAGA TCACATGAAT TGCATGTGTC GGTGGACAAG GTTGCCAAAA   
  
  
+ TTCCTTATTC CTATACAGAA TCTTTTCATT GTTATATTAT CATAGTCGAA ACCCAGTAGT ATATAAAGAG   
  
  
+ GTTACGAGTG GAGGATGGAT ATGAAATAAA TAAGAGGCCT TAGAGATCAG GGTGTGAGAC ATGGATTTGG   
  
  
+ CAAATGGTGA GAGATCTAGT GAGC  

- TTAGTAATTT CAGATTAAGA AGTTTTAAGG CTTAAGATTT AAATCGATAA ATTTTAAGGT TAACAGGATA   
  
  
- GAGGCTTGAA TTAAAAGTCA AAGGGTACGA GGTTAAGGGC ATAAATGGGG CGATGAATAA TAATAGTAAT   
  
  
- AGTAATAGTG GAATAAGTAA TAAAGATATT TAAGGCGGAA GCTTACTAAA GATTCTAGAG GTTAAAAATA   
  
  
- TGTAGTTGAA AGTATTAAAG ATGCAGAGTT TATTGTATGC TAAGACTAAG GAAAGTTTTA AATTGAGGGT   
  
  
- ATTAAGGTTA AAAGCATTGA ATTAAAAAGA ATTTTTAGGG CTTGAAAGTT TTAGGGTTAA AAGAAAGTTT   
  
  
- AAATTAAAGA TTTACAAGAG TAAGAATGTA GATTAAGGAT TCTTAGGTTA AAAGTTTATT AGAGATTCTA   
  
  
- AGGCTAAGAG TTTACTTAAA GTTTTTAGGT CAAAGGGAAG CTTGTTAAAA TTAGGGACAA ATCACTACGT   
  
  
- ACATTATACA AAACACGAGA TATACCACAT GATTGGGAGA GATAACTATC GCGTACCACC AAGCACCGGG   
  
  
- TCCATACATG GGTAAAAGAG AGATTAGCAA CAGATACGTA CAGAGCTAAG AATACACACG TGCTAGTAAG   
  
  
- TCCTATAAGT AACTAAATGA GTAGTTAACA GTGCCGTCGA AGTAAAATAA TCATCTCTGG GCTGAAATCC   
  
  
- CTGAATCTCC CCACGATGCC AGGAATGGCA TGGAAGGACT ATTCATTGGA CTGGGGGCTT GGGCTAGGCC   
  
  
- AAAAAGTGTC TGGTAGAAAA GGTTTTATTC CTCAGTGTGA ATCCCAAAAA GAAAGAATAA AACAAATGGG   
  
  
- AAATTTTTAT TTTGTTTTTA TTCACCGCTG AGGTTCAGTA AAAAGAATTA GTTATTTCTA GTAAAAAAGC   
  
  
- TTTTGCTTTT AGCTCGAGCG GTAGCTCACC CTTTGCGTAA CTCGGCTTTA CGCCCCAGGT ATCCATACAA   
  
  
- AAAATAAATA CTCTATTTTT CATTTATTAT AAAAAAAGCC CAAATTTTTT ATATAATAAC TAATGAACAT   
  
  
- TAATATACTC GGAGCAAACA TGAATAATAA TTTATGTATA AGTTCCATTT TAATAGGAAA TTTTTTAAGA   
  
  
- ATCCGGCTCC CTTGGAAGGA TAATTATATT CAGTAGGTCG GGTGGAATAC CCCCATTAAG AGGGGTATAT   
  
  
- GGTACCCAGG TTTAGCCGCC GGGCCAGTCA AGTTGCTGTC ATGCTACCGT GAGACTTGGG ACACGACGTA   
  
  
- GACGAGTGTT TTTCATGTGG TGTCAACAGA AAGTGAGAGA GACGGGTAAG TGATACGTTT GTGCAAATAT   
  
  
- TTTAACCAAT AAAATATTTG CAAAGAGAAA GTTTACCAAT AAAATATAAT CATTGTTTTT ATAATATTAT   
  
  
- TATTTTTTTA GTTATAATGC ATTAACGATT TTTACTTTAA TGTAGTACAA CATTCTATAA AGTAAACGGA   
  
  
- TAATTATTAA AAAATAATAA ATATTTTAAC CCAACTTAAA GTTATAAAAA ATTTTATTCA GAATAAATAA   
  
  
- ATTCACTTTA AAAAAAATAT CTATTTCAGT GTTTTTAGTA AAAAGATTTA TTAAATATTT TTCTATGAAA   
  
  
- AGGTTATTTT TAATTTAATT TTACGAAAAT ATTTATTTTA ACATTGTTAA GTAAGGTATC GTTTCCATTT   
  
  
- ATTTCGGTAA AAGAATAGTT AATTTAGTTC GAAGAGAAAA AATACTTTAA CAAAAATTAT TTAAGAAAAT   
  
  
- CATTGTTATT CTAAAACAAG TATAAAATTA TGTAGAGGAA CCGTTAACCC GTTTCTACCG ACCATCACTG   
  
  
- TACTTAGGTT CGTACTTAAT CAGGCCCGCA ACAATGAACG ATGTTCCCAA TACTCGTACC TTCCTTTTTA   
  
  
- TTAATTTTTT ATTTTTTTTA ATTTAATGAA GTTAGTGTTT CAACTTTTAT TATTTTAACA CAAACCTTCA   
  
  
- TAAACATGAA TTTCAAAAAA TCATTTTCAC AAAGGGATTT TCCACAAAAA AAATCTCTTT CGTCGATATT   
  
  
- TTACAAAAAA GTTTTTATAA AAAAATAAAT TTTTGTGAAA GAATTTAGTG AACAATATTC AGTGGAATCT   
  
  
- TACTGACTGC CACAACGTGT ATAGTTTTTC GTAAATACGG GATCACGGTA AAATAATAAA ATATAGAATT   
  
  
- AAAATCTATA AAAATAAAAA TAAATTACTA AATATAAACT TGCAATTAAA AGTTATATAG TATAACATAG   
  
  
- AAAATATCGG TGCAAATAAG TTTATATAAA AAATATAAAT ATTTATAAAA AAGATTTTAT ACTAAGCTCT   
  
  
- TACGACTGTA CACTTAGCGT ACCCCCATCC CGTTCGGTAG AACCTGGGTA ATTTGAAGTT TAAGGTACTA   
  
  
- ACTGATCCTG AACGGGTTTT TAAAGTAATG GGGTCTACGG GTGTACACTT ACGTCAGAGA CTGTCTCATC   
  
  
- CACCCTTATT TGGTTTTACG TAAATAAATA AAAAGTTCTA AAATTAATAA TAAGTGGATT TTAGGAAGTA   
  
  
- TCCCTAATTT TATAATCTGG TAACCTAAAC GAGAGGAGTT ATCCCAACTT GAAACTAATC ATGCAAATAT   
  
  
- AATAAAAAAA GGAAAAAGCA GTTTTACGTA AGACAGTGTT TTAATTACAT CAGTTCCTTA ACTGTTCACT   
  
  
- CTTCTCTCAG TGAAATTTAC TAAGTCGACA GGTGTAATTT CTATTCAGTG TAAACACCAG GTATGAGGGG   
  
  
- TTCTGAAATT AAACTTAAAT CGAGTCTGAA AAGCATAAAA TAAACACAAA AAGATAAATT CGATAGTTAT   
  
  
- GTAAATATTT ATTCACTAAA ATTATATTTT CTAGTTCATT TTTTGTTTAT TTCATGTATT TTATAAAAAT   
  
  
- AAGTTAATGT ATAACATAGT TTAAAAGTTC TCGTTTTAAA TATCGTAAAA AGTTACAAAA CCTTTAGTTT   
  
  
- TTTCATTATA TAATAGTCAA GTGATCAATT TAACCACTAA CTTGGTATCT ACTACAGTAT TATATATAAA   
  
  
- ATATAAATAA TTTTAATTTT TATAAATATT CTTTAAAATT TATATACTCT TTAATTTTCA GTTATTATAA   
  
  
- AAAAAGAAGT AGAAATTATA ATAGTTTACT TATATATATA TATATATTTA CATAACTATA AAATTTTAAA   
  
  
- ACAATTTATT TTATATATAT TATACTTCTA TAAATAACTT TCAATAAATT AAATGGATAT ATATATAATT   
  
  
- TTCAATAAAT ACCAACATTT TTTATAATGT AATAAAATAA ATATAATAAT TATAATGTAA TAAAATAAAT   
  
  
- ATAATAAATA TTAAAGTAAT AATATTTTTC AAACTTTTAT AATAACGATA TATTAAACAA CAACACACTT   
  
  
- CTAGCCGTTT TTTTGTCCTC ATCGTCACAC CACAGCAGGA ACGGGGAACT TGACCTCCAA CCAAAGGGTA   
  
  
- AGGAGGGAGG GTGCTTAGGG TGTACATTTC GTTTATTTTC TCGAAATGTA TGTGTACTAA TTACATAAAA   
  
  
- ATAACAGTTA TTTAAAATAT TATTTTGAAT ATTTAGAATA TTTAAATTAT CCTTTTTTTT TTCAGAAGAT   
  
  
- CTTAACTAAA ATATTATGTG GTATGGTTCT AGTGTACTTA ACGTACACAG CCACCTGTTC CAACGGTTTT   
  
  
- AAGGAATAAG GATATGTCTT AGAAAAGTAA CAATATAATA GTATCAGCTT TGGGTCATCA TATATTTCTC   
  
  
- CAATGCTCAC CTCCTACCTA TACTTTATTT ATTCTCCGGA ATCTCTAGTC CCACACTCTG TACCTAAACC   
  
  
- GTTTACCACT CTCTAGATCA CTCG

+     MYB-like sequence

| Site Name | Organism | Position | Strand | Matrix score. | sequence | function |
| --- | --- | --- | --- | --- | --- | --- |
| MYB-like sequence | Arabidopsis thaliana | 1335 | - | 6 | TAACCA |  |
| MYB-like sequence | Arabidopsis thaliana | 1365 | - | 6 | TAACCA |  |

>PlantCARE\_32120   
+ AATCATTAAA GTCTAATTCT TCAAAATTCC GAATTCTAAA TTTAGCTATT TAAAATTCCA ATTGTCCTAT   
  
  
+ CTCCGAACTT AATTTTCAGT TTCCCATGCT CCAATTCCCG TATTTACCCC GCTACTTATT ATTATCATTA   
  
  
+ TCATTATCAC CTTATTCATT ATTTCTATAA ATTCCGCCTT CGAATGATTT CTAAGATCTC CAATTTTTAT   
  
  
+ ACATCAACTT TCATAATTTC TACGTCTCAA ATAACATACG ATTCTGATTC CTTTCAAAAT TTAACTCCCA   
  
  
+ TAATTCCAAT TTTCGTAACT TAATTTTTCT TAAAAATCCC GAACTTTCAA AATCCCAATT TTCTTTCAAA   
  
  
+ TTTAATTTCT AAATGTTCTC ATTCTTACAT CTAATTCCTA AGAATCCAAT TTTCAAATAA TCTCTAAGAT   
  
  
+ TCCGATTCTC AAATGAATTT CAAAAATCCA GTTTCCCTTC GAACAATTTT AATCCCTGTT TAGTGATGCA   
  
  
+ TGTAATATGT TTTGTGCTCT ATATGGTGTA CTAACCCTCT CTATTGATAG CGCATGGTGG TTCGTGGCCC   
  
  
+ AGGTATGTAC CCATTTTCTC TCTAATCGTT GTCTATGCAT GTCTCGATTC TTATGTGTGC ACGATCATTC   
  
  
+ AGGATATTCA TTGATTTACT CATCAATTGT CACGGCAGCT TCATTTTATT AGTAGAGACC CGACTTTAGG   
  
  
+ GACTTAGAGG GGTGCTACGG TCCTTACCGT ACCTTCCTGA TAAGTAACCT GACCCCCGAA CCCGATCCGG   
  
  
+ TTTTTCACAG ACCATCTTTT CCAAAATAAG GAGTCACACT TAGGGTTTTT CTTTCTTATT TTGTTTACCC   
  
  
+ TTTAAAAATA AAACAAAAAT AAGTGGCGAC TCCAAGTCAT TTTTCTTAAT CAATAAAGAT CATTTTTTCG   
  
  
+ AAAACGAAAA TCGAGCTCGC CATCGAGTGG GAAACGCATT GAGCCGAAAT GCGGGGTCCA TAGGTATGTT   
  
  
+ TTTTATTTAT GAGATAAAAA GTAAATAATA TTTTTTTCGG GTTTAAAAAA TATATTATTG ATTACTTGTA   
  
  
+ ATTATATGAG CCTCGTTTGT ACTTATTATT AAATACATAT TCAAGGTAAA ATTATCCTTT AAAAAATTCT   
  
  
+ TAGGCCGAGG GAACCTTCCT ATTAATATAA GTCATCCAGC CCACCTTATG GGGGTAATTC TCCCCATATA   
  
  
+ CCATGGGTCC AAATCGGCGG CCCGGTCAGT TCAACGACAG TACGATGGCA CTCTGAACCC TGTGCTGCAT   
  
  
+ CTGCTCACAA AAAGTACACC ACAGTTGTCT TTCACTCTCT CTGCCCATTC ACTATGCAAA CACGTTTATA   
  
  
+ AAATTGGTTA TTTTATAAAC GTTTCTCTTT CAAATGGTTA TTTTATATTA GTAACAAAAA TATTATAATA   
  
  
+ ATAAAAAAAT CAATATTACG TAATTGCTAA AAATGAAATT ACATCATGTT GTAAGATATT TCATTTGCCT   
  
  
+ ATTAATAATT TTTTATTATT TATAAAATTG GGTTGAATTT CAATATTTTT TAAAATAAGT CTTATTTATT   
  
  
+ TAAGTGAAAT TTTTTTTATA GATAAAGTCA CAAAAATCAT TTTTCTAAAT AATTTATAAA AAGATACTTT   
  
  
+ TCCAATAAAA ATTAAATTAA AATGCTTTTA TAAATAAAAT TGTAACAATT CATTCCATAG CAAAGGTAAA   
  
  
+ TAAAGCCATT TTCTTATCAA TTAAATCAAG CTTCTCTTTT TTATGAAATT GTTTTTAATA AATTCTTTTA   
  
  
+ GTAACAATAA GATTTTGTTC ATATTTTAAT ACATCTCCTT GGCAATTGGG CAAAGATGGC TGGTAGTGAC   
  
  
+ ATGAATCCAA GCATGAATTA GTCCGGGCGT TGTTACTTGC TACAAGGGTT ATGAGCATGG AAGGAAAAAT   
  
  
+ AATTAAAAAA TAAAAAAAAT TAAATTACTT CAATCACAAA GTTGAAAATA ATAAAATTGT GTTTGGAAGT   
  
  
+ ATTTGTACTT AAAGTTTTTT AGTAAAAGTG TTTCCCTAAA AGGTGTTTTT TTTAGAGAAA GCAGCTATAA   
  
  
+ AATGTTTTTT CAAAAATATT TTTTTATTTA AAAACACTTT CTTAAATCAC TTGTTATAAG TCACCTTAGA   
  
  
+ ATGACTGACG GTGTTGCACA TATCAAAAAG CATTTATGCC CTAGTGCCAT TTTATTATTT TATATCTTAA   
  
  
+ TTTTAGATAT TTTTATTTTT ATTTAATGAT TTATATTTGA ACGTTAATTT TCAATATATC ATATTGTATC   
  
  
+ TTTTATAGCC ACGTTTATTC AAATATATTT TTTATATTTA TAAATATTTT TTCTAAAATA TGATTCGAGA   
  
  
+ ATGCTGACAT GTGAATCGCA TGGGGGTAGG GCAAGCCATC TTGGACCCAT TAAACTTCAA ATTCCATGAT   
  
  
+ TGACTAGGAC TTGCCCAAAA ATTTCATTAC CCCAGATGCC CACATGTGAA TGCAGTCTCT GACAGAGTAG   
  
  
+ GTGGGAATAA ACCAAAATGC ATTTATTTAT TTTTCAAGAT TTTAATTATT ATTCACCTAA AATCCTTCAT   
  
  
+ AGGGATTAAA ATATTAGACC ATTGGATTTG CTCTCCTCAA TAGGGTTGAA CTTTGATTAG TACGTTTATA   
  
  
+ TTATTTTTTT CCTTTTTCGT CAAAATGCAT TCTGTCACAA AATTAATGTA GTCAAGGAAT TGACAAGTGA   
  
  
+ GAAGAGAGTC ACTTTAAATG ATTCAGCTGT CCACATTAAA GATAAGTCAC ATTTGTGGTC CATACTCCCC   
  
  
+ AAGACTTTAA TTTGAATTTA GCTCAGACTT TTCGTATTTT ATTTGTGTTT TTCTATTTAA GCTATCAATA   
  
  
+ CATTTATAAA TAAGTGATTT TAATATAAAA GATCAAGTAA AAAACAAATA AAGTACATAA AATATTTTTA   
  
  
+ TTCAATTACA TATTGTATCA AATTTTCAAG AGCAAAATTT ATAGCATTTT TCAATGTTTT GGAAATCAAA   
  
  
+ AAAGTAATAT ATTATCAGTT CACTAGTTAA ATTGGTGATT GAACCATAGA TGATGTCATA ATATATATTT   
  
  
+ TATATTTATT AAAATTAAAA ATATTTATAA GAAATTTTAA ATATATGAGA AATTAAAAGT CAATAATATT   
  
  
+ TTTTTCTTCA TCTTTAATAT TATCAAATGA ATATATATAT ATATATAAAT GTATTGATAT TTTAAAATTT   
  
  
+ TGTTAAATAA AATATATATA ATATGAAGAT ATTTATTGAA AGTTATTTAA TTTACCTATA TATATATTAA   
  
  
+ AAGTTATTTA TGGTTGTAAA AAATATTACA TTATTTTATT TATATTATTA ATATTACATT ATTTTATTTA   
  
  
+ TATTATTTAT AATTTCATTA TTATAAAAAG TTTGAAAATA TTATTGCTAT ATAATTTGTT GTTGTGTGAA   
  
  
+ GATCGGCAAA AAAACAGGAG TAGCAGTGTG GTGTCGTCCT TGCCCCTTGA ACTGGAGGTT GGTTTCCCAT   
  
  
+ TCCTCCCTCC CACGAATCCC ACATGTAAAG CAAATAAAAG AGCTTTACAT ACACATGATT AATGTATTTT   
  
  
+ TATTGTCAAT AAATTTTATA ATAAAACTTA TAAATCTTAT AAATTTAATA GGAAAAAAAA AAGTCTTCTA   
  
  
+ GAATTGATTT TATAATACAC CATACCAAGA TCACATGAAT TGCATGTGTC GGTGGACAAG GTTGCCAAAA   
  
  
+ TTCCTTATTC CTATACAGAA TCTTTTCATT GTTATATTAT CATAGTCGAA ACCCAGTAGT ATATAAAGAG   
  
  
+ GTTACGAGTG GAGGATGGAT ATGAAATAAA TAAGAGGCCT TAGAGATCAG GGTGTGAGAC ATGGATTTGG   
  
  
+ CAAATGGTGA GAGATCTAGT GAGC  

- TTAGTAATTT CAGATTAAGA AGTTTTAAGG CTTAAGATTT AAATCGATAA ATTTTAAGGT TAACAGGATA   
  
  
- GAGGCTTGAA TTAAAAGTCA AAGGGTACGA GGTTAAGGGC ATAAATGGGG CGATGAATAA TAATAGTAAT   
  
  
- AGTAATAGTG GAATAAGTAA TAAAGATATT TAAGGCGGAA GCTTACTAAA GATTCTAGAG GTTAAAAATA   
  
  
- TGTAGTTGAA AGTATTAAAG ATGCAGAGTT TATTGTATGC TAAGACTAAG GAAAGTTTTA AATTGAGGGT   
  
  
- ATTAAGGTTA AAAGCATTGA ATTAAAAAGA ATTTTTAGGG CTTGAAAGTT TTAGGGTTAA AAGAAAGTTT   
  
  
- AAATTAAAGA TTTACAAGAG TAAGAATGTA GATTAAGGAT TCTTAGGTTA AAAGTTTATT AGAGATTCTA   
  
  
- AGGCTAAGAG TTTACTTAAA GTTTTTAGGT CAAAGGGAAG CTTGTTAAAA TTAGGGACAA ATCACTACGT   
  
  
- ACATTATACA AAACACGAGA TATACCACAT GATTGGGAGA GATAACTATC GCGTACCACC AAGCACCGGG   
  
  
- TCCATACATG GGTAAAAGAG AGATTAGCAA CAGATACGTA CAGAGCTAAG AATACACACG TGCTAGTAAG   
  
  
- TCCTATAAGT AACTAAATGA GTAGTTAACA GTGCCGTCGA AGTAAAATAA TCATCTCTGG GCTGAAATCC   
  
  
- CTGAATCTCC CCACGATGCC AGGAATGGCA TGGAAGGACT ATTCATTGGA CTGGGGGCTT GGGCTAGGCC   
  
  
- AAAAAGTGTC TGGTAGAAAA GGTTTTATTC CTCAGTGTGA ATCCCAAAAA GAAAGAATAA AACAAATGGG   
  
  
- AAATTTTTAT TTTGTTTTTA TTCACCGCTG AGGTTCAGTA AAAAGAATTA GTTATTTCTA GTAAAAAAGC   
  
  
- TTTTGCTTTT AGCTCGAGCG GTAGCTCACC CTTTGCGTAA CTCGGCTTTA CGCCCCAGGT ATCCATACAA   
  
  
- AAAATAAATA CTCTATTTTT CATTTATTAT AAAAAAAGCC CAAATTTTTT ATATAATAAC TAATGAACAT   
  
  
- TAATATACTC GGAGCAAACA TGAATAATAA TTTATGTATA AGTTCCATTT TAATAGGAAA TTTTTTAAGA   
  
  
- ATCCGGCTCC CTTGGAAGGA TAATTATATT CAGTAGGTCG GGTGGAATAC CCCCATTAAG AGGGGTATAT   
  
  
- GGTACCCAGG TTTAGCCGCC GGGCCAGTCA AGTTGCTGTC ATGCTACCGT GAGACTTGGG ACACGACGTA   
  
  
- GACGAGTGTT TTTCATGTGG TGTCAACAGA AAGTGAGAGA GACGGGTAAG TGATACGTTT GTGCAAATAT   
  
  
- TTTAACCAAT AAAATATTTG CAAAGAGAAA GTTTACCAAT AAAATATAAT CATTGTTTTT ATAATATTAT   
  
  
- TATTTTTTTA GTTATAATGC ATTAACGATT TTTACTTTAA TGTAGTACAA CATTCTATAA AGTAAACGGA   
  
  
- TAATTATTAA AAAATAATAA ATATTTTAAC CCAACTTAAA GTTATAAAAA ATTTTATTCA GAATAAATAA   
  
  
- ATTCACTTTA AAAAAAATAT CTATTTCAGT GTTTTTAGTA AAAAGATTTA TTAAATATTT TTCTATGAAA   
  
  
- AGGTTATTTT TAATTTAATT TTACGAAAAT ATTTATTTTA ACATTGTTAA GTAAGGTATC GTTTCCATTT   
  
  
- ATTTCGGTAA AAGAATAGTT AATTTAGTTC GAAGAGAAAA AATACTTTAA CAAAAATTAT TTAAGAAAAT   
  
  
- CATTGTTATT CTAAAACAAG TATAAAATTA TGTAGAGGAA CCGTTAACCC GTTTCTACCG ACCATCACTG   
  
  
- TACTTAGGTT CGTACTTAAT CAGGCCCGCA ACAATGAACG ATGTTCCCAA TACTCGTACC TTCCTTTTTA   
  
  
- TTAATTTTTT ATTTTTTTTA ATTTAATGAA GTTAGTGTTT CAACTTTTAT TATTTTAACA CAAACCTTCA   
  
  
- TAAACATGAA TTTCAAAAAA TCATTTTCAC AAAGGGATTT TCCACAAAAA AAATCTCTTT CGTCGATATT   
  
  
- TTACAAAAAA GTTTTTATAA AAAAATAAAT TTTTGTGAAA GAATTTAGTG AACAATATTC AGTGGAATCT   
  
  
- TACTGACTGC CACAACGTGT ATAGTTTTTC GTAAATACGG GATCACGGTA AAATAATAAA ATATAGAATT   
  
  
- AAAATCTATA AAAATAAAAA TAAATTACTA AATATAAACT TGCAATTAAA AGTTATATAG TATAACATAG   
  
  
- AAAATATCGG TGCAAATAAG TTTATATAAA AAATATAAAT ATTTATAAAA AAGATTTTAT ACTAAGCTCT   
  
  
- TACGACTGTA CACTTAGCGT ACCCCCATCC CGTTCGGTAG AACCTGGGTA ATTTGAAGTT TAAGGTACTA   
  
  
- ACTGATCCTG AACGGGTTTT TAAAGTAATG GGGTCTACGG GTGTACACTT ACGTCAGAGA CTGTCTCATC   
  
  
- CACCCTTATT TGGTTTTACG TAAATAAATA AAAAGTTCTA AAATTAATAA TAAGTGGATT TTAGGAAGTA   
  
  
- TCCCTAATTT TATAATCTGG TAACCTAAAC GAGAGGAGTT ATCCCAACTT GAAACTAATC ATGCAAATAT   
  
  
- AATAAAAAAA GGAAAAAGCA GTTTTACGTA AGACAGTGTT TTAATTACAT CAGTTCCTTA ACTGTTCACT   
  
  
- CTTCTCTCAG TGAAATTTAC TAAGTCGACA GGTGTAATTT CTATTCAGTG TAAACACCAG GTATGAGGGG   
  
  
- TTCTGAAATT AAACTTAAAT CGAGTCTGAA AAGCATAAAA TAAACACAAA AAGATAAATT CGATAGTTAT   
  
  
- GTAAATATTT ATTCACTAAA ATTATATTTT CTAGTTCATT TTTTGTTTAT TTCATGTATT TTATAAAAAT   
  
  
- AAGTTAATGT ATAACATAGT TTAAAAGTTC TCGTTTTAAA TATCGTAAAA AGTTACAAAA CCTTTAGTTT   
  
  
- TTTCATTATA TAATAGTCAA GTGATCAATT TAACCACTAA CTTGGTATCT ACTACAGTAT TATATATAAA   
  
  
- ATATAAATAA TTTTAATTTT TATAAATATT CTTTAAAATT TATATACTCT TTAATTTTCA GTTATTATAA   
  
  
- AAAAAGAAGT AGAAATTATA ATAGTTTACT TATATATATA TATATATTTA CATAACTATA AAATTTTAAA   
  
  
- ACAATTTATT TTATATATAT TATACTTCTA TAAATAACTT TCAATAAATT AAATGGATAT ATATATAATT   
  
  
- TTCAATAAAT ACCAACATTT TTTATAATGT AATAAAATAA ATATAATAAT TATAATGTAA TAAAATAAAT   
  
  
- ATAATAAATA TTAAAGTAAT AATATTTTTC AAACTTTTAT AATAACGATA TATTAAACAA CAACACACTT   
  
  
- CTAGCCGTTT TTTTGTCCTC ATCGTCACAC CACAGCAGGA ACGGGGAACT TGACCTCCAA CCAAAGGGTA   
  
  
- AGGAGGGAGG GTGCTTAGGG TGTACATTTC GTTTATTTTC TCGAAATGTA TGTGTACTAA TTACATAAAA   
  
  
- ATAACAGTTA TTTAAAATAT TATTTTGAAT ATTTAGAATA TTTAAATTAT CCTTTTTTTT TTCAGAAGAT   
  
  
- CTTAACTAAA ATATTATGTG GTATGGTTCT AGTGTACTTA ACGTACACAG CCACCTGTTC CAACGGTTTT   
  
  
- AAGGAATAAG GATATGTCTT AGAAAAGTAA CAATATAATA GTATCAGCTT TGGGTCATCA TATATTTCTC   
  
  
- CAATGCTCAC CTCCTACCTA TACTTTATTT ATTCTCCGGA ATCTCTAGTC CCACACTCTG TACCTAAACC   
  
  
- GTTTACCACT CTCTAGATCA CTCG

+     MYC

| Site Name | Organism | Position | Strand | Matrix score. | sequence | function |
| --- | --- | --- | --- | --- | --- | --- |
| MYC | Arabidopsis thaliana | 59 | + | 6 | CAATTG |  |
| MYC | Arabidopsis thaliana | 3104 | - | 6 | CATTTG |  |
| MYC | Arabidopsis thaliana | 1462 | + | 6 | CATTTG |  |
| MYC | Arabidopsis thaliana | 3482 | - | 6 | CATGTG |  |
| MYC | Arabidopsis thaliana | 654 | + | 6 | CAATTG |  |
| MYC | Arabidopsis thaliana | 2423 | + | 6 | CATGTG |  |
| MYC | Arabidopsis thaliana | 430 | - | 6 | CATTTG |  |
| MYC | Arabidopsis thaliana | 3613 | + | 6 | CATGTG |  |
| MYC | Arabidopsis thaliana | 1361 | - | 6 | CATTTG |  |
| MYC | Arabidopsis thaliana | 2710 | + | 6 | CATTTG |  |
| MYC | Arabidopsis thaliana | 2318 | + | 6 | CATGTG |  |
| MYC | Arabidopsis thaliana | 3602 | - | 6 | CATGTG |  |
| MYC | Arabidopsis thaliana | 1793 | + | 6 | CAATTG |  |
| MYC | Arabidopsis thaliana | 3450 | - | 6 | CATGTG |  |
| MYC | Arabidopsis thaliana | 2421 | - | 6 | CATGTG |  |
| MYC | Arabidopsis thaliana | 3781 | - | 6 | CATTTG |  |

>PlantCARE\_32120   
+ AATCATTAAA GTCTAATTCT TCAAAATTCC GAATTCTAAA TTTAGCTATT TAAAATTCCA ATTGTCCTAT   
  
  
+ CTCCGAACTT AATTTTCAGT TTCCCATGCT CCAATTCCCG TATTTACCCC GCTACTTATT ATTATCATTA   
  
  
+ TCATTATCAC CTTATTCATT ATTTCTATAA ATTCCGCCTT CGAATGATTT CTAAGATCTC CAATTTTTAT   
  
  
+ ACATCAACTT TCATAATTTC TACGTCTCAA ATAACATACG ATTCTGATTC CTTTCAAAAT TTAACTCCCA   
  
  
+ TAATTCCAAT TTTCGTAACT TAATTTTTCT TAAAAATCCC GAACTTTCAA AATCCCAATT TTCTTTCAAA   
  
  
+ TTTAATTTCT AAATGTTCTC ATTCTTACAT CTAATTCCTA AGAATCCAAT TTTCAAATAA TCTCTAAGAT   
  
  
+ TCCGATTCTC AAATGAATTT CAAAAATCCA GTTTCCCTTC GAACAATTTT AATCCCTGTT TAGTGATGCA   
  
  
+ TGTAATATGT TTTGTGCTCT ATATGGTGTA CTAACCCTCT CTATTGATAG CGCATGGTGG TTCGTGGCCC   
  
  
+ AGGTATGTAC CCATTTTCTC TCTAATCGTT GTCTATGCAT GTCTCGATTC TTATGTGTGC ACGATCATTC   
  
  
+ AGGATATTCA TTGATTTACT CATCAATTGT CACGGCAGCT TCATTTTATT AGTAGAGACC CGACTTTAGG   
  
  
+ GACTTAGAGG GGTGCTACGG TCCTTACCGT ACCTTCCTGA TAAGTAACCT GACCCCCGAA CCCGATCCGG   
  
  
+ TTTTTCACAG ACCATCTTTT CCAAAATAAG GAGTCACACT TAGGGTTTTT CTTTCTTATT TTGTTTACCC   
  
  
+ TTTAAAAATA AAACAAAAAT AAGTGGCGAC TCCAAGTCAT TTTTCTTAAT CAATAAAGAT CATTTTTTCG   
  
  
+ AAAACGAAAA TCGAGCTCGC CATCGAGTGG GAAACGCATT GAGCCGAAAT GCGGGGTCCA TAGGTATGTT   
  
  
+ TTTTATTTAT GAGATAAAAA GTAAATAATA TTTTTTTCGG GTTTAAAAAA TATATTATTG ATTACTTGTA   
  
  
+ ATTATATGAG CCTCGTTTGT ACTTATTATT AAATACATAT TCAAGGTAAA ATTATCCTTT AAAAAATTCT   
  
  
+ TAGGCCGAGG GAACCTTCCT ATTAATATAA GTCATCCAGC CCACCTTATG GGGGTAATTC TCCCCATATA   
  
  
+ CCATGGGTCC AAATCGGCGG CCCGGTCAGT TCAACGACAG TACGATGGCA CTCTGAACCC TGTGCTGCAT   
  
  
+ CTGCTCACAA AAAGTACACC ACAGTTGTCT TTCACTCTCT CTGCCCATTC ACTATGCAAA CACGTTTATA   
  
  
+ AAATTGGTTA TTTTATAAAC GTTTCTCTTT CAAATGGTTA TTTTATATTA GTAACAAAAA TATTATAATA   
  
  
+ ATAAAAAAAT CAATATTACG TAATTGCTAA AAATGAAATT ACATCATGTT GTAAGATATT TCATTTGCCT   
  
  
+ ATTAATAATT TTTTATTATT TATAAAATTG GGTTGAATTT CAATATTTTT TAAAATAAGT CTTATTTATT   
  
  
+ TAAGTGAAAT TTTTTTTATA GATAAAGTCA CAAAAATCAT TTTTCTAAAT AATTTATAAA AAGATACTTT   
  
  
+ TCCAATAAAA ATTAAATTAA AATGCTTTTA TAAATAAAAT TGTAACAATT CATTCCATAG CAAAGGTAAA   
  
  
+ TAAAGCCATT TTCTTATCAA TTAAATCAAG CTTCTCTTTT TTATGAAATT GTTTTTAATA AATTCTTTTA   
  
  
+ GTAACAATAA GATTTTGTTC ATATTTTAAT ACATCTCCTT GGCAATTGGG CAAAGATGGC TGGTAGTGAC   
  
  
+ ATGAATCCAA GCATGAATTA GTCCGGGCGT TGTTACTTGC TACAAGGGTT ATGAGCATGG AAGGAAAAAT   
  
  
+ AATTAAAAAA TAAAAAAAAT TAAATTACTT CAATCACAAA GTTGAAAATA ATAAAATTGT GTTTGGAAGT   
  
  
+ ATTTGTACTT AAAGTTTTTT AGTAAAAGTG TTTCCCTAAA AGGTGTTTTT TTTAGAGAAA GCAGCTATAA   
  
  
+ AATGTTTTTT CAAAAATATT TTTTTATTTA AAAACACTTT CTTAAATCAC TTGTTATAAG TCACCTTAGA   
  
  
+ ATGACTGACG GTGTTGCACA TATCAAAAAG CATTTATGCC CTAGTGCCAT TTTATTATTT TATATCTTAA   
  
  
+ TTTTAGATAT TTTTATTTTT ATTTAATGAT TTATATTTGA ACGTTAATTT TCAATATATC ATATTGTATC   
  
  
+ TTTTATAGCC ACGTTTATTC AAATATATTT TTTATATTTA TAAATATTTT TTCTAAAATA TGATTCGAGA   
  
  
+ ATGCTGACAT GTGAATCGCA TGGGGGTAGG GCAAGCCATC TTGGACCCAT TAAACTTCAA ATTCCATGAT   
  
  
+ TGACTAGGAC TTGCCCAAAA ATTTCATTAC CCCAGATGCC CACATGTGAA TGCAGTCTCT GACAGAGTAG   
  
  
+ GTGGGAATAA ACCAAAATGC ATTTATTTAT TTTTCAAGAT TTTAATTATT ATTCACCTAA AATCCTTCAT   
  
  
+ AGGGATTAAA ATATTAGACC ATTGGATTTG CTCTCCTCAA TAGGGTTGAA CTTTGATTAG TACGTTTATA   
  
  
+ TTATTTTTTT CCTTTTTCGT CAAAATGCAT TCTGTCACAA AATTAATGTA GTCAAGGAAT TGACAAGTGA   
  
  
+ GAAGAGAGTC ACTTTAAATG ATTCAGCTGT CCACATTAAA GATAAGTCAC ATTTGTGGTC CATACTCCCC   
  
  
+ AAGACTTTAA TTTGAATTTA GCTCAGACTT TTCGTATTTT ATTTGTGTTT TTCTATTTAA GCTATCAATA   
  
  
+ CATTTATAAA TAAGTGATTT TAATATAAAA GATCAAGTAA AAAACAAATA AAGTACATAA AATATTTTTA   
  
  
+ TTCAATTACA TATTGTATCA AATTTTCAAG AGCAAAATTT ATAGCATTTT TCAATGTTTT GGAAATCAAA   
  
  
+ AAAGTAATAT ATTATCAGTT CACTAGTTAA ATTGGTGATT GAACCATAGA TGATGTCATA ATATATATTT   
  
  
+ TATATTTATT AAAATTAAAA ATATTTATAA GAAATTTTAA ATATATGAGA AATTAAAAGT CAATAATATT   
  
  
+ TTTTTCTTCA TCTTTAATAT TATCAAATGA ATATATATAT ATATATAAAT GTATTGATAT TTTAAAATTT   
  
  
+ TGTTAAATAA AATATATATA ATATGAAGAT ATTTATTGAA AGTTATTTAA TTTACCTATA TATATATTAA   
  
  
+ AAGTTATTTA TGGTTGTAAA AAATATTACA TTATTTTATT TATATTATTA ATATTACATT ATTTTATTTA   
  
  
+ TATTATTTAT AATTTCATTA TTATAAAAAG TTTGAAAATA TTATTGCTAT ATAATTTGTT GTTGTGTGAA   
  
  
+ GATCGGCAAA AAAACAGGAG TAGCAGTGTG GTGTCGTCCT TGCCCCTTGA ACTGGAGGTT GGTTTCCCAT   
  
  
+ TCCTCCCTCC CACGAATCCC ACATGTAAAG CAAATAAAAG AGCTTTACAT ACACATGATT AATGTATTTT   
  
  
+ TATTGTCAAT AAATTTTATA ATAAAACTTA TAAATCTTAT AAATTTAATA GGAAAAAAAA AAGTCTTCTA   
  
  
+ GAATTGATTT TATAATACAC CATACCAAGA TCACATGAAT TGCATGTGTC GGTGGACAAG GTTGCCAAAA   
  
  
+ TTCCTTATTC CTATACAGAA TCTTTTCATT GTTATATTAT CATAGTCGAA ACCCAGTAGT ATATAAAGAG   
  
  
+ GTTACGAGTG GAGGATGGAT ATGAAATAAA TAAGAGGCCT TAGAGATCAG GGTGTGAGAC ATGGATTTGG   
  
  
+ CAAATGGTGA GAGATCTAGT GAGC  

- TTAGTAATTT CAGATTAAGA AGTTTTAAGG CTTAAGATTT AAATCGATAA ATTTTAAGGT TAACAGGATA   
  
  
- GAGGCTTGAA TTAAAAGTCA AAGGGTACGA GGTTAAGGGC ATAAATGGGG CGATGAATAA TAATAGTAAT   
  
  
- AGTAATAGTG GAATAAGTAA TAAAGATATT TAAGGCGGAA GCTTACTAAA GATTCTAGAG GTTAAAAATA   
  
  
- TGTAGTTGAA AGTATTAAAG ATGCAGAGTT TATTGTATGC TAAGACTAAG GAAAGTTTTA AATTGAGGGT   
  
  
- ATTAAGGTTA AAAGCATTGA ATTAAAAAGA ATTTTTAGGG CTTGAAAGTT TTAGGGTTAA AAGAAAGTTT   
  
  
- AAATTAAAGA TTTACAAGAG TAAGAATGTA GATTAAGGAT TCTTAGGTTA AAAGTTTATT AGAGATTCTA   
  
  
- AGGCTAAGAG TTTACTTAAA GTTTTTAGGT CAAAGGGAAG CTTGTTAAAA TTAGGGACAA ATCACTACGT   
  
  
- ACATTATACA AAACACGAGA TATACCACAT GATTGGGAGA GATAACTATC GCGTACCACC AAGCACCGGG   
  
  
- TCCATACATG GGTAAAAGAG AGATTAGCAA CAGATACGTA CAGAGCTAAG AATACACACG TGCTAGTAAG   
  
  
- TCCTATAAGT AACTAAATGA GTAGTTAACA GTGCCGTCGA AGTAAAATAA TCATCTCTGG GCTGAAATCC   
  
  
- CTGAATCTCC CCACGATGCC AGGAATGGCA TGGAAGGACT ATTCATTGGA CTGGGGGCTT GGGCTAGGCC   
  
  
- AAAAAGTGTC TGGTAGAAAA GGTTTTATTC CTCAGTGTGA ATCCCAAAAA GAAAGAATAA AACAAATGGG   
  
  
- AAATTTTTAT TTTGTTTTTA TTCACCGCTG AGGTTCAGTA AAAAGAATTA GTTATTTCTA GTAAAAAAGC   
  
  
- TTTTGCTTTT AGCTCGAGCG GTAGCTCACC CTTTGCGTAA CTCGGCTTTA CGCCCCAGGT ATCCATACAA   
  
  
- AAAATAAATA CTCTATTTTT CATTTATTAT AAAAAAAGCC CAAATTTTTT ATATAATAAC TAATGAACAT   
  
  
- TAATATACTC GGAGCAAACA TGAATAATAA TTTATGTATA AGTTCCATTT TAATAGGAAA TTTTTTAAGA   
  
  
- ATCCGGCTCC CTTGGAAGGA TAATTATATT CAGTAGGTCG GGTGGAATAC CCCCATTAAG AGGGGTATAT   
  
  
- GGTACCCAGG TTTAGCCGCC GGGCCAGTCA AGTTGCTGTC ATGCTACCGT GAGACTTGGG ACACGACGTA   
  
  
- GACGAGTGTT TTTCATGTGG TGTCAACAGA AAGTGAGAGA GACGGGTAAG TGATACGTTT GTGCAAATAT   
  
  
- TTTAACCAAT AAAATATTTG CAAAGAGAAA GTTTACCAAT AAAATATAAT CATTGTTTTT ATAATATTAT   
  
  
- TATTTTTTTA GTTATAATGC ATTAACGATT TTTACTTTAA TGTAGTACAA CATTCTATAA AGTAAACGGA   
  
  
- TAATTATTAA AAAATAATAA ATATTTTAAC CCAACTTAAA GTTATAAAAA ATTTTATTCA GAATAAATAA   
  
  
- ATTCACTTTA AAAAAAATAT CTATTTCAGT GTTTTTAGTA AAAAGATTTA TTAAATATTT TTCTATGAAA   
  
  
- AGGTTATTTT TAATTTAATT TTACGAAAAT ATTTATTTTA ACATTGTTAA GTAAGGTATC GTTTCCATTT   
  
  
- ATTTCGGTAA AAGAATAGTT AATTTAGTTC GAAGAGAAAA AATACTTTAA CAAAAATTAT TTAAGAAAAT   
  
  
- CATTGTTATT CTAAAACAAG TATAAAATTA TGTAGAGGAA CCGTTAACCC GTTTCTACCG ACCATCACTG   
  
  
- TACTTAGGTT CGTACTTAAT CAGGCCCGCA ACAATGAACG ATGTTCCCAA TACTCGTACC TTCCTTTTTA   
  
  
- TTAATTTTTT ATTTTTTTTA ATTTAATGAA GTTAGTGTTT CAACTTTTAT TATTTTAACA CAAACCTTCA   
  
  
- TAAACATGAA TTTCAAAAAA TCATTTTCAC AAAGGGATTT TCCACAAAAA AAATCTCTTT CGTCGATATT   
  
  
- TTACAAAAAA GTTTTTATAA AAAAATAAAT TTTTGTGAAA GAATTTAGTG AACAATATTC AGTGGAATCT   
  
  
- TACTGACTGC CACAACGTGT ATAGTTTTTC GTAAATACGG GATCACGGTA AAATAATAAA ATATAGAATT   
  
  
- AAAATCTATA AAAATAAAAA TAAATTACTA AATATAAACT TGCAATTAAA AGTTATATAG TATAACATAG   
  
  
- AAAATATCGG TGCAAATAAG TTTATATAAA AAATATAAAT ATTTATAAAA AAGATTTTAT ACTAAGCTCT   
  
  
- TACGACTGTA CACTTAGCGT ACCCCCATCC CGTTCGGTAG AACCTGGGTA ATTTGAAGTT TAAGGTACTA   
  
  
- ACTGATCCTG AACGGGTTTT TAAAGTAATG GGGTCTACGG GTGTACACTT ACGTCAGAGA CTGTCTCATC   
  
  
- CACCCTTATT TGGTTTTACG TAAATAAATA AAAAGTTCTA AAATTAATAA TAAGTGGATT TTAGGAAGTA   
  
  
- TCCCTAATTT TATAATCTGG TAACCTAAAC GAGAGGAGTT ATCCCAACTT GAAACTAATC ATGCAAATAT   
  
  
- AATAAAAAAA GGAAAAAGCA GTTTTACGTA AGACAGTGTT TTAATTACAT CAGTTCCTTA ACTGTTCACT   
  
  
- CTTCTCTCAG TGAAATTTAC TAAGTCGACA GGTGTAATTT CTATTCAGTG TAAACACCAG GTATGAGGGG   
  
  
- TTCTGAAATT AAACTTAAAT CGAGTCTGAA AAGCATAAAA TAAACACAAA AAGATAAATT CGATAGTTAT   
  
  
- GTAAATATTT ATTCACTAAA ATTATATTTT CTAGTTCATT TTTTGTTTAT TTCATGTATT TTATAAAAAT   
  
  
- AAGTTAATGT ATAACATAGT TTAAAAGTTC TCGTTTTAAA TATCGTAAAA AGTTACAAAA CCTTTAGTTT   
  
  
- TTTCATTATA TAATAGTCAA GTGATCAATT TAACCACTAA CTTGGTATCT ACTACAGTAT TATATATAAA   
  
  
- ATATAAATAA TTTTAATTTT TATAAATATT CTTTAAAATT TATATACTCT TTAATTTTCA GTTATTATAA   
  
  
- AAAAAGAAGT AGAAATTATA ATAGTTTACT TATATATATA TATATATTTA CATAACTATA AAATTTTAAA   
  
  
- ACAATTTATT TTATATATAT TATACTTCTA TAAATAACTT TCAATAAATT AAATGGATAT ATATATAATT   
  
  
- TTCAATAAAT ACCAACATTT TTTATAATGT AATAAAATAA ATATAATAAT TATAATGTAA TAAAATAAAT   
  
  
- ATAATAAATA TTAAAGTAAT AATATTTTTC AAACTTTTAT AATAACGATA TATTAAACAA CAACACACTT   
  
  
- CTAGCCGTTT TTTTGTCCTC ATCGTCACAC CACAGCAGGA ACGGGGAACT TGACCTCCAA CCAAAGGGTA   
  
  
- AGGAGGGAGG GTGCTTAGGG TGTACATTTC GTTTATTTTC TCGAAATGTA TGTGTACTAA TTACATAAAA   
  
  
- ATAACAGTTA TTTAAAATAT TATTTTGAAT ATTTAGAATA TTTAAATTAT CCTTTTTTTT TTCAGAAGAT   
  
  
- CTTAACTAAA ATATTATGTG GTATGGTTCT AGTGTACTTA ACGTACACAG CCACCTGTTC CAACGGTTTT   
  
  
- AAGGAATAAG GATATGTCTT AGAAAAGTAA CAATATAATA GTATCAGCTT TGGGTCATCA TATATTTCTC   
  
  
- CAATGCTCAC CTCCTACCTA TACTTTATTT ATTCTCCGGA ATCTCTAGTC CCACACTCTG TACCTAAACC   
  
  
- GTTTACCACT CTCTAGATCA CTCG

+     Myb

| Site Name | Organism | Position | Strand | Matrix score. | sequence | function |
| --- | --- | --- | --- | --- | --- | --- |
| Myb | Arabidopsis thaliana | 1282 | - | 6 | CAACTG |  |

>PlantCARE\_32120   
+ AATCATTAAA GTCTAATTCT TCAAAATTCC GAATTCTAAA TTTAGCTATT TAAAATTCCA ATTGTCCTAT   
  
  
+ CTCCGAACTT AATTTTCAGT TTCCCATGCT CCAATTCCCG TATTTACCCC GCTACTTATT ATTATCATTA   
  
  
+ TCATTATCAC CTTATTCATT ATTTCTATAA ATTCCGCCTT CGAATGATTT CTAAGATCTC CAATTTTTAT   
  
  
+ ACATCAACTT TCATAATTTC TACGTCTCAA ATAACATACG ATTCTGATTC CTTTCAAAAT TTAACTCCCA   
  
  
+ TAATTCCAAT TTTCGTAACT TAATTTTTCT TAAAAATCCC GAACTTTCAA AATCCCAATT TTCTTTCAAA   
  
  
+ TTTAATTTCT AAATGTTCTC ATTCTTACAT CTAATTCCTA AGAATCCAAT TTTCAAATAA TCTCTAAGAT   
  
  
+ TCCGATTCTC AAATGAATTT CAAAAATCCA GTTTCCCTTC GAACAATTTT AATCCCTGTT TAGTGATGCA   
  
  
+ TGTAATATGT TTTGTGCTCT ATATGGTGTA CTAACCCTCT CTATTGATAG CGCATGGTGG TTCGTGGCCC   
  
  
+ AGGTATGTAC CCATTTTCTC TCTAATCGTT GTCTATGCAT GTCTCGATTC TTATGTGTGC ACGATCATTC   
  
  
+ AGGATATTCA TTGATTTACT CATCAATTGT CACGGCAGCT TCATTTTATT AGTAGAGACC CGACTTTAGG   
  
  
+ GACTTAGAGG GGTGCTACGG TCCTTACCGT ACCTTCCTGA TAAGTAACCT GACCCCCGAA CCCGATCCGG   
  
  
+ TTTTTCACAG ACCATCTTTT CCAAAATAAG GAGTCACACT TAGGGTTTTT CTTTCTTATT TTGTTTACCC   
  
  
+ TTTAAAAATA AAACAAAAAT AAGTGGCGAC TCCAAGTCAT TTTTCTTAAT CAATAAAGAT CATTTTTTCG   
  
  
+ AAAACGAAAA TCGAGCTCGC CATCGAGTGG GAAACGCATT GAGCCGAAAT GCGGGGTCCA TAGGTATGTT   
  
  
+ TTTTATTTAT GAGATAAAAA GTAAATAATA TTTTTTTCGG GTTTAAAAAA TATATTATTG ATTACTTGTA   
  
  
+ ATTATATGAG CCTCGTTTGT ACTTATTATT AAATACATAT TCAAGGTAAA ATTATCCTTT AAAAAATTCT   
  
  
+ TAGGCCGAGG GAACCTTCCT ATTAATATAA GTCATCCAGC CCACCTTATG GGGGTAATTC TCCCCATATA   
  
  
+ CCATGGGTCC AAATCGGCGG CCCGGTCAGT TCAACGACAG TACGATGGCA CTCTGAACCC TGTGCTGCAT   
  
  
+ CTGCTCACAA AAAGTACACC ACAGTTGTCT TTCACTCTCT CTGCCCATTC ACTATGCAAA CACGTTTATA   
  
  
+ AAATTGGTTA TTTTATAAAC GTTTCTCTTT CAAATGGTTA TTTTATATTA GTAACAAAAA TATTATAATA   
  
  
+ ATAAAAAAAT CAATATTACG TAATTGCTAA AAATGAAATT ACATCATGTT GTAAGATATT TCATTTGCCT   
  
  
+ ATTAATAATT TTTTATTATT TATAAAATTG GGTTGAATTT CAATATTTTT TAAAATAAGT CTTATTTATT   
  
  
+ TAAGTGAAAT TTTTTTTATA GATAAAGTCA CAAAAATCAT TTTTCTAAAT AATTTATAAA AAGATACTTT   
  
  
+ TCCAATAAAA ATTAAATTAA AATGCTTTTA TAAATAAAAT TGTAACAATT CATTCCATAG CAAAGGTAAA   
  
  
+ TAAAGCCATT TTCTTATCAA TTAAATCAAG CTTCTCTTTT TTATGAAATT GTTTTTAATA AATTCTTTTA   
  
  
+ GTAACAATAA GATTTTGTTC ATATTTTAAT ACATCTCCTT GGCAATTGGG CAAAGATGGC TGGTAGTGAC   
  
  
+ ATGAATCCAA GCATGAATTA GTCCGGGCGT TGTTACTTGC TACAAGGGTT ATGAGCATGG AAGGAAAAAT   
  
  
+ AATTAAAAAA TAAAAAAAAT TAAATTACTT CAATCACAAA GTTGAAAATA ATAAAATTGT GTTTGGAAGT   
  
  
+ ATTTGTACTT AAAGTTTTTT AGTAAAAGTG TTTCCCTAAA AGGTGTTTTT TTTAGAGAAA GCAGCTATAA   
  
  
+ AATGTTTTTT CAAAAATATT TTTTTATTTA AAAACACTTT CTTAAATCAC TTGTTATAAG TCACCTTAGA   
  
  
+ ATGACTGACG GTGTTGCACA TATCAAAAAG CATTTATGCC CTAGTGCCAT TTTATTATTT TATATCTTAA   
  
  
+ TTTTAGATAT TTTTATTTTT ATTTAATGAT TTATATTTGA ACGTTAATTT TCAATATATC ATATTGTATC   
  
  
+ TTTTATAGCC ACGTTTATTC AAATATATTT TTTATATTTA TAAATATTTT TTCTAAAATA TGATTCGAGA   
  
  
+ ATGCTGACAT GTGAATCGCA TGGGGGTAGG GCAAGCCATC TTGGACCCAT TAAACTTCAA ATTCCATGAT   
  
  
+ TGACTAGGAC TTGCCCAAAA ATTTCATTAC CCCAGATGCC CACATGTGAA TGCAGTCTCT GACAGAGTAG   
  
  
+ GTGGGAATAA ACCAAAATGC ATTTATTTAT TTTTCAAGAT TTTAATTATT ATTCACCTAA AATCCTTCAT   
  
  
+ AGGGATTAAA ATATTAGACC ATTGGATTTG CTCTCCTCAA TAGGGTTGAA CTTTGATTAG TACGTTTATA   
  
  
+ TTATTTTTTT CCTTTTTCGT CAAAATGCAT TCTGTCACAA AATTAATGTA GTCAAGGAAT TGACAAGTGA   
  
  
+ GAAGAGAGTC ACTTTAAATG ATTCAGCTGT CCACATTAAA GATAAGTCAC ATTTGTGGTC CATACTCCCC   
  
  
+ AAGACTTTAA TTTGAATTTA GCTCAGACTT TTCGTATTTT ATTTGTGTTT TTCTATTTAA GCTATCAATA   
  
  
+ CATTTATAAA TAAGTGATTT TAATATAAAA GATCAAGTAA AAAACAAATA AAGTACATAA AATATTTTTA   
  
  
+ TTCAATTACA TATTGTATCA AATTTTCAAG AGCAAAATTT ATAGCATTTT TCAATGTTTT GGAAATCAAA   
  
  
+ AAAGTAATAT ATTATCAGTT CACTAGTTAA ATTGGTGATT GAACCATAGA TGATGTCATA ATATATATTT   
  
  
+ TATATTTATT AAAATTAAAA ATATTTATAA GAAATTTTAA ATATATGAGA AATTAAAAGT CAATAATATT   
  
  
+ TTTTTCTTCA TCTTTAATAT TATCAAATGA ATATATATAT ATATATAAAT GTATTGATAT TTTAAAATTT   
  
  
+ TGTTAAATAA AATATATATA ATATGAAGAT ATTTATTGAA AGTTATTTAA TTTACCTATA TATATATTAA   
  
  
+ AAGTTATTTA TGGTTGTAAA AAATATTACA TTATTTTATT TATATTATTA ATATTACATT ATTTTATTTA   
  
  
+ TATTATTTAT AATTTCATTA TTATAAAAAG TTTGAAAATA TTATTGCTAT ATAATTTGTT GTTGTGTGAA   
  
  
+ GATCGGCAAA AAAACAGGAG TAGCAGTGTG GTGTCGTCCT TGCCCCTTGA ACTGGAGGTT GGTTTCCCAT   
  
  
+ TCCTCCCTCC CACGAATCCC ACATGTAAAG CAAATAAAAG AGCTTTACAT ACACATGATT AATGTATTTT   
  
  
+ TATTGTCAAT AAATTTTATA ATAAAACTTA TAAATCTTAT AAATTTAATA GGAAAAAAAA AAGTCTTCTA   
  
  
+ GAATTGATTT TATAATACAC CATACCAAGA TCACATGAAT TGCATGTGTC GGTGGACAAG GTTGCCAAAA   
  
  
+ TTCCTTATTC CTATACAGAA TCTTTTCATT GTTATATTAT CATAGTCGAA ACCCAGTAGT ATATAAAGAG   
  
  
+ GTTACGAGTG GAGGATGGAT ATGAAATAAA TAAGAGGCCT TAGAGATCAG GGTGTGAGAC ATGGATTTGG   
  
  
+ CAAATGGTGA GAGATCTAGT GAGC  

- TTAGTAATTT CAGATTAAGA AGTTTTAAGG CTTAAGATTT AAATCGATAA ATTTTAAGGT TAACAGGATA   
  
  
- GAGGCTTGAA TTAAAAGTCA AAGGGTACGA GGTTAAGGGC ATAAATGGGG CGATGAATAA TAATAGTAAT   
  
  
- AGTAATAGTG GAATAAGTAA TAAAGATATT TAAGGCGGAA GCTTACTAAA GATTCTAGAG GTTAAAAATA   
  
  
- TGTAGTTGAA AGTATTAAAG ATGCAGAGTT TATTGTATGC TAAGACTAAG GAAAGTTTTA AATTGAGGGT   
  
  
- ATTAAGGTTA AAAGCATTGA ATTAAAAAGA ATTTTTAGGG CTTGAAAGTT TTAGGGTTAA AAGAAAGTTT   
  
  
- AAATTAAAGA TTTACAAGAG TAAGAATGTA GATTAAGGAT TCTTAGGTTA AAAGTTTATT AGAGATTCTA   
  
  
- AGGCTAAGAG TTTACTTAAA GTTTTTAGGT CAAAGGGAAG CTTGTTAAAA TTAGGGACAA ATCACTACGT   
  
  
- ACATTATACA AAACACGAGA TATACCACAT GATTGGGAGA GATAACTATC GCGTACCACC AAGCACCGGG   
  
  
- TCCATACATG GGTAAAAGAG AGATTAGCAA CAGATACGTA CAGAGCTAAG AATACACACG TGCTAGTAAG   
  
  
- TCCTATAAGT AACTAAATGA GTAGTTAACA GTGCCGTCGA AGTAAAATAA TCATCTCTGG GCTGAAATCC   
  
  
- CTGAATCTCC CCACGATGCC AGGAATGGCA TGGAAGGACT ATTCATTGGA CTGGGGGCTT GGGCTAGGCC   
  
  
- AAAAAGTGTC TGGTAGAAAA GGTTTTATTC CTCAGTGTGA ATCCCAAAAA GAAAGAATAA AACAAATGGG   
  
  
- AAATTTTTAT TTTGTTTTTA TTCACCGCTG AGGTTCAGTA AAAAGAATTA GTTATTTCTA GTAAAAAAGC   
  
  
- TTTTGCTTTT AGCTCGAGCG GTAGCTCACC CTTTGCGTAA CTCGGCTTTA CGCCCCAGGT ATCCATACAA   
  
  
- AAAATAAATA CTCTATTTTT CATTTATTAT AAAAAAAGCC CAAATTTTTT ATATAATAAC TAATGAACAT   
  
  
- TAATATACTC GGAGCAAACA TGAATAATAA TTTATGTATA AGTTCCATTT TAATAGGAAA TTTTTTAAGA   
  
  
- ATCCGGCTCC CTTGGAAGGA TAATTATATT CAGTAGGTCG GGTGGAATAC CCCCATTAAG AGGGGTATAT   
  
  
- GGTACCCAGG TTTAGCCGCC GGGCCAGTCA AGTTGCTGTC ATGCTACCGT GAGACTTGGG ACACGACGTA   
  
  
- GACGAGTGTT TTTCATGTGG TGTCAACAGA AAGTGAGAGA GACGGGTAAG TGATACGTTT GTGCAAATAT   
  
  
- TTTAACCAAT AAAATATTTG CAAAGAGAAA GTTTACCAAT AAAATATAAT CATTGTTTTT ATAATATTAT   
  
  
- TATTTTTTTA GTTATAATGC ATTAACGATT TTTACTTTAA TGTAGTACAA CATTCTATAA AGTAAACGGA   
  
  
- TAATTATTAA AAAATAATAA ATATTTTAAC CCAACTTAAA GTTATAAAAA ATTTTATTCA GAATAAATAA   
  
  
- ATTCACTTTA AAAAAAATAT CTATTTCAGT GTTTTTAGTA AAAAGATTTA TTAAATATTT TTCTATGAAA   
  
  
- AGGTTATTTT TAATTTAATT TTACGAAAAT ATTTATTTTA ACATTGTTAA GTAAGGTATC GTTTCCATTT   
  
  
- ATTTCGGTAA AAGAATAGTT AATTTAGTTC GAAGAGAAAA AATACTTTAA CAAAAATTAT TTAAGAAAAT   
  
  
- CATTGTTATT CTAAAACAAG TATAAAATTA TGTAGAGGAA CCGTTAACCC GTTTCTACCG ACCATCACTG   
  
  
- TACTTAGGTT CGTACTTAAT CAGGCCCGCA ACAATGAACG ATGTTCCCAA TACTCGTACC TTCCTTTTTA   
  
  
- TTAATTTTTT ATTTTTTTTA ATTTAATGAA GTTAGTGTTT CAACTTTTAT TATTTTAACA CAAACCTTCA   
  
  
- TAAACATGAA TTTCAAAAAA TCATTTTCAC AAAGGGATTT TCCACAAAAA AAATCTCTTT CGTCGATATT   
  
  
- TTACAAAAAA GTTTTTATAA AAAAATAAAT TTTTGTGAAA GAATTTAGTG AACAATATTC AGTGGAATCT   
  
  
- TACTGACTGC CACAACGTGT ATAGTTTTTC GTAAATACGG GATCACGGTA AAATAATAAA ATATAGAATT   
  
  
- AAAATCTATA AAAATAAAAA TAAATTACTA AATATAAACT TGCAATTAAA AGTTATATAG TATAACATAG   
  
  
- AAAATATCGG TGCAAATAAG TTTATATAAA AAATATAAAT ATTTATAAAA AAGATTTTAT ACTAAGCTCT   
  
  
- TACGACTGTA CACTTAGCGT ACCCCCATCC CGTTCGGTAG AACCTGGGTA ATTTGAAGTT TAAGGTACTA   
  
  
- ACTGATCCTG AACGGGTTTT TAAAGTAATG GGGTCTACGG GTGTACACTT ACGTCAGAGA CTGTCTCATC   
  
  
- CACCCTTATT TGGTTTTACG TAAATAAATA AAAAGTTCTA AAATTAATAA TAAGTGGATT TTAGGAAGTA   
  
  
- TCCCTAATTT TATAATCTGG TAACCTAAAC GAGAGGAGTT ATCCCAACTT GAAACTAATC ATGCAAATAT   
  
  
- AATAAAAAAA GGAAAAAGCA GTTTTACGTA AGACAGTGTT TTAATTACAT CAGTTCCTTA ACTGTTCACT   
  
  
- CTTCTCTCAG TGAAATTTAC TAAGTCGACA GGTGTAATTT CTATTCAGTG TAAACACCAG GTATGAGGGG   
  
  
- TTCTGAAATT AAACTTAAAT CGAGTCTGAA AAGCATAAAA TAAACACAAA AAGATAAATT CGATAGTTAT   
  
  
- GTAAATATTT ATTCACTAAA ATTATATTTT CTAGTTCATT TTTTGTTTAT TTCATGTATT TTATAAAAAT   
  
  
- AAGTTAATGT ATAACATAGT TTAAAAGTTC TCGTTTTAAA TATCGTAAAA AGTTACAAAA CCTTTAGTTT   
  
  
- TTTCATTATA TAATAGTCAA GTGATCAATT TAACCACTAA CTTGGTATCT ACTACAGTAT TATATATAAA   
  
  
- ATATAAATAA TTTTAATTTT TATAAATATT CTTTAAAATT TATATACTCT TTAATTTTCA GTTATTATAA   
  
  
- AAAAAGAAGT AGAAATTATA ATAGTTTACT TATATATATA TATATATTTA CATAACTATA AAATTTTAAA   
  
  
- ACAATTTATT TTATATATAT TATACTTCTA TAAATAACTT TCAATAAATT AAATGGATAT ATATATAATT   
  
  
- TTCAATAAAT ACCAACATTT TTTATAATGT AATAAAATAA ATATAATAAT TATAATGTAA TAAAATAAAT   
  
  
- ATAATAAATA TTAAAGTAAT AATATTTTTC AAACTTTTAT AATAACGATA TATTAAACAA CAACACACTT   
  
  
- CTAGCCGTTT TTTTGTCCTC ATCGTCACAC CACAGCAGGA ACGGGGAACT TGACCTCCAA CCAAAGGGTA   
  
  
- AGGAGGGAGG GTGCTTAGGG TGTACATTTC GTTTATTTTC TCGAAATGTA TGTGTACTAA TTACATAAAA   
  
  
- ATAACAGTTA TTTAAAATAT TATTTTGAAT ATTTAGAATA TTTAAATTAT CCTTTTTTTT TTCAGAAGAT   
  
  
- CTTAACTAAA ATATTATGTG GTATGGTTCT AGTGTACTTA ACGTACACAG CCACCTGTTC CAACGGTTTT   
  
  
- AAGGAATAAG GATATGTCTT AGAAAAGTAA CAATATAATA GTATCAGCTT TGGGTCATCA TATATTTCTC   
  
  
- CAATGCTCAC CTCCTACCTA TACTTTATTT ATTCTCCGGA ATCTCTAGTC CCACACTCTG TACCTAAACC   
  
  
- GTTTACCACT CTCTAGATCA CTCG

+     STRE

| Site Name | Organism | Position | Strand | Matrix score. | sequence | function |
| --- | --- | --- | --- | --- | --- | --- |
| STRE | Arabidopsis thaliana | 708 | + | 5 | AGGGG |  |
| STRE | Arabidopsis thaliana | 3403 | - | 5 | AGGGG |  |

>PlantCARE\_32120   
+ AATCATTAAA GTCTAATTCT TCAAAATTCC GAATTCTAAA TTTAGCTATT TAAAATTCCA ATTGTCCTAT   
  
  
+ CTCCGAACTT AATTTTCAGT TTCCCATGCT CCAATTCCCG TATTTACCCC GCTACTTATT ATTATCATTA   
  
  
+ TCATTATCAC CTTATTCATT ATTTCTATAA ATTCCGCCTT CGAATGATTT CTAAGATCTC CAATTTTTAT   
  
  
+ ACATCAACTT TCATAATTTC TACGTCTCAA ATAACATACG ATTCTGATTC CTTTCAAAAT TTAACTCCCA   
  
  
+ TAATTCCAAT TTTCGTAACT TAATTTTTCT TAAAAATCCC GAACTTTCAA AATCCCAATT TTCTTTCAAA   
  
  
+ TTTAATTTCT AAATGTTCTC ATTCTTACAT CTAATTCCTA AGAATCCAAT TTTCAAATAA TCTCTAAGAT   
  
  
+ TCCGATTCTC AAATGAATTT CAAAAATCCA GTTTCCCTTC GAACAATTTT AATCCCTGTT TAGTGATGCA   
  
  
+ TGTAATATGT TTTGTGCTCT ATATGGTGTA CTAACCCTCT CTATTGATAG CGCATGGTGG TTCGTGGCCC   
  
  
+ AGGTATGTAC CCATTTTCTC TCTAATCGTT GTCTATGCAT GTCTCGATTC TTATGTGTGC ACGATCATTC   
  
  
+ AGGATATTCA TTGATTTACT CATCAATTGT CACGGCAGCT TCATTTTATT AGTAGAGACC CGACTTTAGG   
  
  
+ GACTTAGAGG GGTGCTACGG TCCTTACCGT ACCTTCCTGA TAAGTAACCT GACCCCCGAA CCCGATCCGG   
  
  
+ TTTTTCACAG ACCATCTTTT CCAAAATAAG GAGTCACACT TAGGGTTTTT CTTTCTTATT TTGTTTACCC   
  
  
+ TTTAAAAATA AAACAAAAAT AAGTGGCGAC TCCAAGTCAT TTTTCTTAAT CAATAAAGAT CATTTTTTCG   
  
  
+ AAAACGAAAA TCGAGCTCGC CATCGAGTGG GAAACGCATT GAGCCGAAAT GCGGGGTCCA TAGGTATGTT   
  
  
+ TTTTATTTAT GAGATAAAAA GTAAATAATA TTTTTTTCGG GTTTAAAAAA TATATTATTG ATTACTTGTA   
  
  
+ ATTATATGAG CCTCGTTTGT ACTTATTATT AAATACATAT TCAAGGTAAA ATTATCCTTT AAAAAATTCT   
  
  
+ TAGGCCGAGG GAACCTTCCT ATTAATATAA GTCATCCAGC CCACCTTATG GGGGTAATTC TCCCCATATA   
  
  
+ CCATGGGTCC AAATCGGCGG CCCGGTCAGT TCAACGACAG TACGATGGCA CTCTGAACCC TGTGCTGCAT   
  
  
+ CTGCTCACAA AAAGTACACC ACAGTTGTCT TTCACTCTCT CTGCCCATTC ACTATGCAAA CACGTTTATA   
  
  
+ AAATTGGTTA TTTTATAAAC GTTTCTCTTT CAAATGGTTA TTTTATATTA GTAACAAAAA TATTATAATA   
  
  
+ ATAAAAAAAT CAATATTACG TAATTGCTAA AAATGAAATT ACATCATGTT GTAAGATATT TCATTTGCCT   
  
  
+ ATTAATAATT TTTTATTATT TATAAAATTG GGTTGAATTT CAATATTTTT TAAAATAAGT CTTATTTATT   
  
  
+ TAAGTGAAAT TTTTTTTATA GATAAAGTCA CAAAAATCAT TTTTCTAAAT AATTTATAAA AAGATACTTT   
  
  
+ TCCAATAAAA ATTAAATTAA AATGCTTTTA TAAATAAAAT TGTAACAATT CATTCCATAG CAAAGGTAAA   
  
  
+ TAAAGCCATT TTCTTATCAA TTAAATCAAG CTTCTCTTTT TTATGAAATT GTTTTTAATA AATTCTTTTA   
  
  
+ GTAACAATAA GATTTTGTTC ATATTTTAAT ACATCTCCTT GGCAATTGGG CAAAGATGGC TGGTAGTGAC   
  
  
+ ATGAATCCAA GCATGAATTA GTCCGGGCGT TGTTACTTGC TACAAGGGTT ATGAGCATGG AAGGAAAAAT   
  
  
+ AATTAAAAAA TAAAAAAAAT TAAATTACTT CAATCACAAA GTTGAAAATA ATAAAATTGT GTTTGGAAGT   
  
  
+ ATTTGTACTT AAAGTTTTTT AGTAAAAGTG TTTCCCTAAA AGGTGTTTTT TTTAGAGAAA GCAGCTATAA   
  
  
+ AATGTTTTTT CAAAAATATT TTTTTATTTA AAAACACTTT CTTAAATCAC TTGTTATAAG TCACCTTAGA   
  
  
+ ATGACTGACG GTGTTGCACA TATCAAAAAG CATTTATGCC CTAGTGCCAT TTTATTATTT TATATCTTAA   
  
  
+ TTTTAGATAT TTTTATTTTT ATTTAATGAT TTATATTTGA ACGTTAATTT TCAATATATC ATATTGTATC   
  
  
+ TTTTATAGCC ACGTTTATTC AAATATATTT TTTATATTTA TAAATATTTT TTCTAAAATA TGATTCGAGA   
  
  
+ ATGCTGACAT GTGAATCGCA TGGGGGTAGG GCAAGCCATC TTGGACCCAT TAAACTTCAA ATTCCATGAT   
  
  
+ TGACTAGGAC TTGCCCAAAA ATTTCATTAC CCCAGATGCC CACATGTGAA TGCAGTCTCT GACAGAGTAG   
  
  
+ GTGGGAATAA ACCAAAATGC ATTTATTTAT TTTTCAAGAT TTTAATTATT ATTCACCTAA AATCCTTCAT   
  
  
+ AGGGATTAAA ATATTAGACC ATTGGATTTG CTCTCCTCAA TAGGGTTGAA CTTTGATTAG TACGTTTATA   
  
  
+ TTATTTTTTT CCTTTTTCGT CAAAATGCAT TCTGTCACAA AATTAATGTA GTCAAGGAAT TGACAAGTGA   
  
  
+ GAAGAGAGTC ACTTTAAATG ATTCAGCTGT CCACATTAAA GATAAGTCAC ATTTGTGGTC CATACTCCCC   
  
  
+ AAGACTTTAA TTTGAATTTA GCTCAGACTT TTCGTATTTT ATTTGTGTTT TTCTATTTAA GCTATCAATA   
  
  
+ CATTTATAAA TAAGTGATTT TAATATAAAA GATCAAGTAA AAAACAAATA AAGTACATAA AATATTTTTA   
  
  
+ TTCAATTACA TATTGTATCA AATTTTCAAG AGCAAAATTT ATAGCATTTT TCAATGTTTT GGAAATCAAA   
  
  
+ AAAGTAATAT ATTATCAGTT CACTAGTTAA ATTGGTGATT GAACCATAGA TGATGTCATA ATATATATTT   
  
  
+ TATATTTATT AAAATTAAAA ATATTTATAA GAAATTTTAA ATATATGAGA AATTAAAAGT CAATAATATT   
  
  
+ TTTTTCTTCA TCTTTAATAT TATCAAATGA ATATATATAT ATATATAAAT GTATTGATAT TTTAAAATTT   
  
  
+ TGTTAAATAA AATATATATA ATATGAAGAT ATTTATTGAA AGTTATTTAA TTTACCTATA TATATATTAA   
  
  
+ AAGTTATTTA TGGTTGTAAA AAATATTACA TTATTTTATT TATATTATTA ATATTACATT ATTTTATTTA   
  
  
+ TATTATTTAT AATTTCATTA TTATAAAAAG TTTGAAAATA TTATTGCTAT ATAATTTGTT GTTGTGTGAA   
  
  
+ GATCGGCAAA AAAACAGGAG TAGCAGTGTG GTGTCGTCCT TGCCCCTTGA ACTGGAGGTT GGTTTCCCAT   
  
  
+ TCCTCCCTCC CACGAATCCC ACATGTAAAG CAAATAAAAG AGCTTTACAT ACACATGATT AATGTATTTT   
  
  
+ TATTGTCAAT AAATTTTATA ATAAAACTTA TAAATCTTAT AAATTTAATA GGAAAAAAAA AAGTCTTCTA   
  
  
+ GAATTGATTT TATAATACAC CATACCAAGA TCACATGAAT TGCATGTGTC GGTGGACAAG GTTGCCAAAA   
  
  
+ TTCCTTATTC CTATACAGAA TCTTTTCATT GTTATATTAT CATAGTCGAA ACCCAGTAGT ATATAAAGAG   
  
  
+ GTTACGAGTG GAGGATGGAT ATGAAATAAA TAAGAGGCCT TAGAGATCAG GGTGTGAGAC ATGGATTTGG   
  
  
+ CAAATGGTGA GAGATCTAGT GAGC  

- TTAGTAATTT CAGATTAAGA AGTTTTAAGG CTTAAGATTT AAATCGATAA ATTTTAAGGT TAACAGGATA   
  
  
- GAGGCTTGAA TTAAAAGTCA AAGGGTACGA GGTTAAGGGC ATAAATGGGG CGATGAATAA TAATAGTAAT   
  
  
- AGTAATAGTG GAATAAGTAA TAAAGATATT TAAGGCGGAA GCTTACTAAA GATTCTAGAG GTTAAAAATA   
  
  
- TGTAGTTGAA AGTATTAAAG ATGCAGAGTT TATTGTATGC TAAGACTAAG GAAAGTTTTA AATTGAGGGT   
  
  
- ATTAAGGTTA AAAGCATTGA ATTAAAAAGA ATTTTTAGGG CTTGAAAGTT TTAGGGTTAA AAGAAAGTTT   
  
  
- AAATTAAAGA TTTACAAGAG TAAGAATGTA GATTAAGGAT TCTTAGGTTA AAAGTTTATT AGAGATTCTA   
  
  
- AGGCTAAGAG TTTACTTAAA GTTTTTAGGT CAAAGGGAAG CTTGTTAAAA TTAGGGACAA ATCACTACGT   
  
  
- ACATTATACA AAACACGAGA TATACCACAT GATTGGGAGA GATAACTATC GCGTACCACC AAGCACCGGG   
  
  
- TCCATACATG GGTAAAAGAG AGATTAGCAA CAGATACGTA CAGAGCTAAG AATACACACG TGCTAGTAAG   
  
  
- TCCTATAAGT AACTAAATGA GTAGTTAACA GTGCCGTCGA AGTAAAATAA TCATCTCTGG GCTGAAATCC   
  
  
- CTGAATCTCC CCACGATGCC AGGAATGGCA TGGAAGGACT ATTCATTGGA CTGGGGGCTT GGGCTAGGCC   
  
  
- AAAAAGTGTC TGGTAGAAAA GGTTTTATTC CTCAGTGTGA ATCCCAAAAA GAAAGAATAA AACAAATGGG   
  
  
- AAATTTTTAT TTTGTTTTTA TTCACCGCTG AGGTTCAGTA AAAAGAATTA GTTATTTCTA GTAAAAAAGC   
  
  
- TTTTGCTTTT AGCTCGAGCG GTAGCTCACC CTTTGCGTAA CTCGGCTTTA CGCCCCAGGT ATCCATACAA   
  
  
- AAAATAAATA CTCTATTTTT CATTTATTAT AAAAAAAGCC CAAATTTTTT ATATAATAAC TAATGAACAT   
  
  
- TAATATACTC GGAGCAAACA TGAATAATAA TTTATGTATA AGTTCCATTT TAATAGGAAA TTTTTTAAGA   
  
  
- ATCCGGCTCC CTTGGAAGGA TAATTATATT CAGTAGGTCG GGTGGAATAC CCCCATTAAG AGGGGTATAT   
  
  
- GGTACCCAGG TTTAGCCGCC GGGCCAGTCA AGTTGCTGTC ATGCTACCGT GAGACTTGGG ACACGACGTA   
  
  
- GACGAGTGTT TTTCATGTGG TGTCAACAGA AAGTGAGAGA GACGGGTAAG TGATACGTTT GTGCAAATAT   
  
  
- TTTAACCAAT AAAATATTTG CAAAGAGAAA GTTTACCAAT AAAATATAAT CATTGTTTTT ATAATATTAT   
  
  
- TATTTTTTTA GTTATAATGC ATTAACGATT TTTACTTTAA TGTAGTACAA CATTCTATAA AGTAAACGGA   
  
  
- TAATTATTAA AAAATAATAA ATATTTTAAC CCAACTTAAA GTTATAAAAA ATTTTATTCA GAATAAATAA   
  
  
- ATTCACTTTA AAAAAAATAT CTATTTCAGT GTTTTTAGTA AAAAGATTTA TTAAATATTT TTCTATGAAA   
  
  
- AGGTTATTTT TAATTTAATT TTACGAAAAT ATTTATTTTA ACATTGTTAA GTAAGGTATC GTTTCCATTT   
  
  
- ATTTCGGTAA AAGAATAGTT AATTTAGTTC GAAGAGAAAA AATACTTTAA CAAAAATTAT TTAAGAAAAT   
  
  
- CATTGTTATT CTAAAACAAG TATAAAATTA TGTAGAGGAA CCGTTAACCC GTTTCTACCG ACCATCACTG   
  
  
- TACTTAGGTT CGTACTTAAT CAGGCCCGCA ACAATGAACG ATGTTCCCAA TACTCGTACC TTCCTTTTTA   
  
  
- TTAATTTTTT ATTTTTTTTA ATTTAATGAA GTTAGTGTTT CAACTTTTAT TATTTTAACA CAAACCTTCA   
  
  
- TAAACATGAA TTTCAAAAAA TCATTTTCAC AAAGGGATTT TCCACAAAAA AAATCTCTTT CGTCGATATT   
  
  
- TTACAAAAAA GTTTTTATAA AAAAATAAAT TTTTGTGAAA GAATTTAGTG AACAATATTC AGTGGAATCT   
  
  
- TACTGACTGC CACAACGTGT ATAGTTTTTC GTAAATACGG GATCACGGTA AAATAATAAA ATATAGAATT   
  
  
- AAAATCTATA AAAATAAAAA TAAATTACTA AATATAAACT TGCAATTAAA AGTTATATAG TATAACATAG   
  
  
- AAAATATCGG TGCAAATAAG TTTATATAAA AAATATAAAT ATTTATAAAA AAGATTTTAT ACTAAGCTCT   
  
  
- TACGACTGTA CACTTAGCGT ACCCCCATCC CGTTCGGTAG AACCTGGGTA ATTTGAAGTT TAAGGTACTA   
  
  
- ACTGATCCTG AACGGGTTTT TAAAGTAATG GGGTCTACGG GTGTACACTT ACGTCAGAGA CTGTCTCATC   
  
  
- CACCCTTATT TGGTTTTACG TAAATAAATA AAAAGTTCTA AAATTAATAA TAAGTGGATT TTAGGAAGTA   
  
  
- TCCCTAATTT TATAATCTGG TAACCTAAAC GAGAGGAGTT ATCCCAACTT GAAACTAATC ATGCAAATAT   
  
  
- AATAAAAAAA GGAAAAAGCA GTTTTACGTA AGACAGTGTT TTAATTACAT CAGTTCCTTA ACTGTTCACT   
  
  
- CTTCTCTCAG TGAAATTTAC TAAGTCGACA GGTGTAATTT CTATTCAGTG TAAACACCAG GTATGAGGGG   
  
  
- TTCTGAAATT AAACTTAAAT CGAGTCTGAA AAGCATAAAA TAAACACAAA AAGATAAATT CGATAGTTAT   
  
  
- GTAAATATTT ATTCACTAAA ATTATATTTT CTAGTTCATT TTTTGTTTAT TTCATGTATT TTATAAAAAT   
  
  
- AAGTTAATGT ATAACATAGT TTAAAAGTTC TCGTTTTAAA TATCGTAAAA AGTTACAAAA CCTTTAGTTT   
  
  
- TTTCATTATA TAATAGTCAA GTGATCAATT TAACCACTAA CTTGGTATCT ACTACAGTAT TATATATAAA   
  
  
- ATATAAATAA TTTTAATTTT TATAAATATT CTTTAAAATT TATATACTCT TTAATTTTCA GTTATTATAA   
  
  
- AAAAAGAAGT AGAAATTATA ATAGTTTACT TATATATATA TATATATTTA CATAACTATA AAATTTTAAA   
  
  
- ACAATTTATT TTATATATAT TATACTTCTA TAAATAACTT TCAATAAATT AAATGGATAT ATATATAATT   
  
  
- TTCAATAAAT ACCAACATTT TTTATAATGT AATAAAATAA ATATAATAAT TATAATGTAA TAAAATAAAT   
  
  
- ATAATAAATA TTAAAGTAAT AATATTTTTC AAACTTTTAT AATAACGATA TATTAAACAA CAACACACTT   
  
  
- CTAGCCGTTT TTTTGTCCTC ATCGTCACAC CACAGCAGGA ACGGGGAACT TGACCTCCAA CCAAAGGGTA   
  
  
- AGGAGGGAGG GTGCTTAGGG TGTACATTTC GTTTATTTTC TCGAAATGTA TGTGTACTAA TTACATAAAA   
  
  
- ATAACAGTTA TTTAAAATAT TATTTTGAAT ATTTAGAATA TTTAAATTAT CCTTTTTTTT TTCAGAAGAT   
  
  
- CTTAACTAAA ATATTATGTG GTATGGTTCT AGTGTACTTA ACGTACACAG CCACCTGTTC CAACGGTTTT   
  
  
- AAGGAATAAG GATATGTCTT AGAAAAGTAA CAATATAATA GTATCAGCTT TGGGTCATCA TATATTTCTC   
  
  
- CAATGCTCAC CTCCTACCTA TACTTTATTT ATTCTCCGGA ATCTCTAGTC CCACACTCTG TACCTAAACC   
  
  
- GTTTACCACT CTCTAGATCA CTCG

+     TATA

| Site Name | Organism | Position | Strand | Matrix score. | sequence | function |
| --- | --- | --- | --- | --- | --- | --- |
| TATA | Arabidopsis thaliana | 1327 | + | 8 | TATAAAAT |  |
| TATA | Arabidopsis thaliana | 3007 | - | 8 | TATAAAAT |  |
| TATA | Arabidopsis thaliana | 2026 | + | 8 | TATAAAAT |  |
| TATA | Arabidopsis thaliana | 3577 | - | 8 | TATAAAAT |  |
| TATA | Arabidopsis thaliana | 1370 | - | 8 | TATAAAAT |  |
| TATA | Arabidopsis thaliana | 3513 | - | 8 | TATAAAAT |  |
| TATA | Arabidopsis thaliana | 2157 | - | 8 | TATAAAAT |  |
| TATA | Arabidopsis thaliana | 1340 | - | 8 | TATAAAAT |  |
| TATA | Arabidopsis thaliana | 1491 | + | 8 | TATAAAAT |  |

>PlantCARE\_32120   
+ AATCATTAAA GTCTAATTCT TCAAAATTCC GAATTCTAAA TTTAGCTATT TAAAATTCCA ATTGTCCTAT   
  
  
+ CTCCGAACTT AATTTTCAGT TTCCCATGCT CCAATTCCCG TATTTACCCC GCTACTTATT ATTATCATTA   
  
  
+ TCATTATCAC CTTATTCATT ATTTCTATAA ATTCCGCCTT CGAATGATTT CTAAGATCTC CAATTTTTAT   
  
  
+ ACATCAACTT TCATAATTTC TACGTCTCAA ATAACATACG ATTCTGATTC CTTTCAAAAT TTAACTCCCA   
  
  
+ TAATTCCAAT TTTCGTAACT TAATTTTTCT TAAAAATCCC GAACTTTCAA AATCCCAATT TTCTTTCAAA   
  
  
+ TTTAATTTCT AAATGTTCTC ATTCTTACAT CTAATTCCTA AGAATCCAAT TTTCAAATAA TCTCTAAGAT   
  
  
+ TCCGATTCTC AAATGAATTT CAAAAATCCA GTTTCCCTTC GAACAATTTT AATCCCTGTT TAGTGATGCA   
  
  
+ TGTAATATGT TTTGTGCTCT ATATGGTGTA CTAACCCTCT CTATTGATAG CGCATGGTGG TTCGTGGCCC   
  
  
+ AGGTATGTAC CCATTTTCTC TCTAATCGTT GTCTATGCAT GTCTCGATTC TTATGTGTGC ACGATCATTC   
  
  
+ AGGATATTCA TTGATTTACT CATCAATTGT CACGGCAGCT TCATTTTATT AGTAGAGACC CGACTTTAGG   
  
  
+ GACTTAGAGG GGTGCTACGG TCCTTACCGT ACCTTCCTGA TAAGTAACCT GACCCCCGAA CCCGATCCGG   
  
  
+ TTTTTCACAG ACCATCTTTT CCAAAATAAG GAGTCACACT TAGGGTTTTT CTTTCTTATT TTGTTTACCC   
  
  
+ TTTAAAAATA AAACAAAAAT AAGTGGCGAC TCCAAGTCAT TTTTCTTAAT CAATAAAGAT CATTTTTTCG   
  
  
+ AAAACGAAAA TCGAGCTCGC CATCGAGTGG GAAACGCATT GAGCCGAAAT GCGGGGTCCA TAGGTATGTT   
  
  
+ TTTTATTTAT GAGATAAAAA GTAAATAATA TTTTTTTCGG GTTTAAAAAA TATATTATTG ATTACTTGTA   
  
  
+ ATTATATGAG CCTCGTTTGT ACTTATTATT AAATACATAT TCAAGGTAAA ATTATCCTTT AAAAAATTCT   
  
  
+ TAGGCCGAGG GAACCTTCCT ATTAATATAA GTCATCCAGC CCACCTTATG GGGGTAATTC TCCCCATATA   
  
  
+ CCATGGGTCC AAATCGGCGG CCCGGTCAGT TCAACGACAG TACGATGGCA CTCTGAACCC TGTGCTGCAT   
  
  
+ CTGCTCACAA AAAGTACACC ACAGTTGTCT TTCACTCTCT CTGCCCATTC ACTATGCAAA CACGTTTATA   
  
  
+ AAATTGGTTA TTTTATAAAC GTTTCTCTTT CAAATGGTTA TTTTATATTA GTAACAAAAA TATTATAATA   
  
  
+ ATAAAAAAAT CAATATTACG TAATTGCTAA AAATGAAATT ACATCATGTT GTAAGATATT TCATTTGCCT   
  
  
+ ATTAATAATT TTTTATTATT TATAAAATTG GGTTGAATTT CAATATTTTT TAAAATAAGT CTTATTTATT   
  
  
+ TAAGTGAAAT TTTTTTTATA GATAAAGTCA CAAAAATCAT TTTTCTAAAT AATTTATAAA AAGATACTTT   
  
  
+ TCCAATAAAA ATTAAATTAA AATGCTTTTA TAAATAAAAT TGTAACAATT CATTCCATAG CAAAGGTAAA   
  
  
+ TAAAGCCATT TTCTTATCAA TTAAATCAAG CTTCTCTTTT TTATGAAATT GTTTTTAATA AATTCTTTTA   
  
  
+ GTAACAATAA GATTTTGTTC ATATTTTAAT ACATCTCCTT GGCAATTGGG CAAAGATGGC TGGTAGTGAC   
  
  
+ ATGAATCCAA GCATGAATTA GTCCGGGCGT TGTTACTTGC TACAAGGGTT ATGAGCATGG AAGGAAAAAT   
  
  
+ AATTAAAAAA TAAAAAAAAT TAAATTACTT CAATCACAAA GTTGAAAATA ATAAAATTGT GTTTGGAAGT   
  
  
+ ATTTGTACTT AAAGTTTTTT AGTAAAAGTG TTTCCCTAAA AGGTGTTTTT TTTAGAGAAA GCAGCTATAA   
  
  
+ AATGTTTTTT CAAAAATATT TTTTTATTTA AAAACACTTT CTTAAATCAC TTGTTATAAG TCACCTTAGA   
  
  
+ ATGACTGACG GTGTTGCACA TATCAAAAAG CATTTATGCC CTAGTGCCAT TTTATTATTT TATATCTTAA   
  
  
+ TTTTAGATAT TTTTATTTTT ATTTAATGAT TTATATTTGA ACGTTAATTT TCAATATATC ATATTGTATC   
  
  
+ TTTTATAGCC ACGTTTATTC AAATATATTT TTTATATTTA TAAATATTTT TTCTAAAATA TGATTCGAGA   
  
  
+ ATGCTGACAT GTGAATCGCA TGGGGGTAGG GCAAGCCATC TTGGACCCAT TAAACTTCAA ATTCCATGAT   
  
  
+ TGACTAGGAC TTGCCCAAAA ATTTCATTAC CCCAGATGCC CACATGTGAA TGCAGTCTCT GACAGAGTAG   
  
  
+ GTGGGAATAA ACCAAAATGC ATTTATTTAT TTTTCAAGAT TTTAATTATT ATTCACCTAA AATCCTTCAT   
  
  
+ AGGGATTAAA ATATTAGACC ATTGGATTTG CTCTCCTCAA TAGGGTTGAA CTTTGATTAG TACGTTTATA   
  
  
+ TTATTTTTTT CCTTTTTCGT CAAAATGCAT TCTGTCACAA AATTAATGTA GTCAAGGAAT TGACAAGTGA   
  
  
+ GAAGAGAGTC ACTTTAAATG ATTCAGCTGT CCACATTAAA GATAAGTCAC ATTTGTGGTC CATACTCCCC   
  
  
+ AAGACTTTAA TTTGAATTTA GCTCAGACTT TTCGTATTTT ATTTGTGTTT TTCTATTTAA GCTATCAATA   
  
  
+ CATTTATAAA TAAGTGATTT TAATATAAAA GATCAAGTAA AAAACAAATA AAGTACATAA AATATTTTTA   
  
  
+ TTCAATTACA TATTGTATCA AATTTTCAAG AGCAAAATTT ATAGCATTTT TCAATGTTTT GGAAATCAAA   
  
  
+ AAAGTAATAT ATTATCAGTT CACTAGTTAA ATTGGTGATT GAACCATAGA TGATGTCATA ATATATATTT   
  
  
+ TATATTTATT AAAATTAAAA ATATTTATAA GAAATTTTAA ATATATGAGA AATTAAAAGT CAATAATATT   
  
  
+ TTTTTCTTCA TCTTTAATAT TATCAAATGA ATATATATAT ATATATAAAT GTATTGATAT TTTAAAATTT   
  
  
+ TGTTAAATAA AATATATATA ATATGAAGAT ATTTATTGAA AGTTATTTAA TTTACCTATA TATATATTAA   
  
  
+ AAGTTATTTA TGGTTGTAAA AAATATTACA TTATTTTATT TATATTATTA ATATTACATT ATTTTATTTA   
  
  
+ TATTATTTAT AATTTCATTA TTATAAAAAG TTTGAAAATA TTATTGCTAT ATAATTTGTT GTTGTGTGAA   
  
  
+ GATCGGCAAA AAAACAGGAG TAGCAGTGTG GTGTCGTCCT TGCCCCTTGA ACTGGAGGTT GGTTTCCCAT   
  
  
+ TCCTCCCTCC CACGAATCCC ACATGTAAAG CAAATAAAAG AGCTTTACAT ACACATGATT AATGTATTTT   
  
  
+ TATTGTCAAT AAATTTTATA ATAAAACTTA TAAATCTTAT AAATTTAATA GGAAAAAAAA AAGTCTTCTA   
  
  
+ GAATTGATTT TATAATACAC CATACCAAGA TCACATGAAT TGCATGTGTC GGTGGACAAG GTTGCCAAAA   
  
  
+ TTCCTTATTC CTATACAGAA TCTTTTCATT GTTATATTAT CATAGTCGAA ACCCAGTAGT ATATAAAGAG   
  
  
+ GTTACGAGTG GAGGATGGAT ATGAAATAAA TAAGAGGCCT TAGAGATCAG GGTGTGAGAC ATGGATTTGG   
  
  
+ CAAATGGTGA GAGATCTAGT GAGC  

- TTAGTAATTT CAGATTAAGA AGTTTTAAGG CTTAAGATTT AAATCGATAA ATTTTAAGGT TAACAGGATA   
  
  
- GAGGCTTGAA TTAAAAGTCA AAGGGTACGA GGTTAAGGGC ATAAATGGGG CGATGAATAA TAATAGTAAT   
  
  
- AGTAATAGTG GAATAAGTAA TAAAGATATT TAAGGCGGAA GCTTACTAAA GATTCTAGAG GTTAAAAATA   
  
  
- TGTAGTTGAA AGTATTAAAG ATGCAGAGTT TATTGTATGC TAAGACTAAG GAAAGTTTTA AATTGAGGGT   
  
  
- ATTAAGGTTA AAAGCATTGA ATTAAAAAGA ATTTTTAGGG CTTGAAAGTT TTAGGGTTAA AAGAAAGTTT   
  
  
- AAATTAAAGA TTTACAAGAG TAAGAATGTA GATTAAGGAT TCTTAGGTTA AAAGTTTATT AGAGATTCTA   
  
  
- AGGCTAAGAG TTTACTTAAA GTTTTTAGGT CAAAGGGAAG CTTGTTAAAA TTAGGGACAA ATCACTACGT   
  
  
- ACATTATACA AAACACGAGA TATACCACAT GATTGGGAGA GATAACTATC GCGTACCACC AAGCACCGGG   
  
  
- TCCATACATG GGTAAAAGAG AGATTAGCAA CAGATACGTA CAGAGCTAAG AATACACACG TGCTAGTAAG   
  
  
- TCCTATAAGT AACTAAATGA GTAGTTAACA GTGCCGTCGA AGTAAAATAA TCATCTCTGG GCTGAAATCC   
  
  
- CTGAATCTCC CCACGATGCC AGGAATGGCA TGGAAGGACT ATTCATTGGA CTGGGGGCTT GGGCTAGGCC   
  
  
- AAAAAGTGTC TGGTAGAAAA GGTTTTATTC CTCAGTGTGA ATCCCAAAAA GAAAGAATAA AACAAATGGG   
  
  
- AAATTTTTAT TTTGTTTTTA TTCACCGCTG AGGTTCAGTA AAAAGAATTA GTTATTTCTA GTAAAAAAGC   
  
  
- TTTTGCTTTT AGCTCGAGCG GTAGCTCACC CTTTGCGTAA CTCGGCTTTA CGCCCCAGGT ATCCATACAA   
  
  
- AAAATAAATA CTCTATTTTT CATTTATTAT AAAAAAAGCC CAAATTTTTT ATATAATAAC TAATGAACAT   
  
  
- TAATATACTC GGAGCAAACA TGAATAATAA TTTATGTATA AGTTCCATTT TAATAGGAAA TTTTTTAAGA   
  
  
- ATCCGGCTCC CTTGGAAGGA TAATTATATT CAGTAGGTCG GGTGGAATAC CCCCATTAAG AGGGGTATAT   
  
  
- GGTACCCAGG TTTAGCCGCC GGGCCAGTCA AGTTGCTGTC ATGCTACCGT GAGACTTGGG ACACGACGTA   
  
  
- GACGAGTGTT TTTCATGTGG TGTCAACAGA AAGTGAGAGA GACGGGTAAG TGATACGTTT GTGCAAATAT   
  
  
- TTTAACCAAT AAAATATTTG CAAAGAGAAA GTTTACCAAT AAAATATAAT CATTGTTTTT ATAATATTAT   
  
  
- TATTTTTTTA GTTATAATGC ATTAACGATT TTTACTTTAA TGTAGTACAA CATTCTATAA AGTAAACGGA   
  
  
- TAATTATTAA AAAATAATAA ATATTTTAAC CCAACTTAAA GTTATAAAAA ATTTTATTCA GAATAAATAA   
  
  
- ATTCACTTTA AAAAAAATAT CTATTTCAGT GTTTTTAGTA AAAAGATTTA TTAAATATTT TTCTATGAAA   
  
  
- AGGTTATTTT TAATTTAATT TTACGAAAAT ATTTATTTTA ACATTGTTAA GTAAGGTATC GTTTCCATTT   
  
  
- ATTTCGGTAA AAGAATAGTT AATTTAGTTC GAAGAGAAAA AATACTTTAA CAAAAATTAT TTAAGAAAAT   
  
  
- CATTGTTATT CTAAAACAAG TATAAAATTA TGTAGAGGAA CCGTTAACCC GTTTCTACCG ACCATCACTG   
  
  
- TACTTAGGTT CGTACTTAAT CAGGCCCGCA ACAATGAACG ATGTTCCCAA TACTCGTACC TTCCTTTTTA   
  
  
- TTAATTTTTT ATTTTTTTTA ATTTAATGAA GTTAGTGTTT CAACTTTTAT TATTTTAACA CAAACCTTCA   
  
  
- TAAACATGAA TTTCAAAAAA TCATTTTCAC AAAGGGATTT TCCACAAAAA AAATCTCTTT CGTCGATATT   
  
  
- TTACAAAAAA GTTTTTATAA AAAAATAAAT TTTTGTGAAA GAATTTAGTG AACAATATTC AGTGGAATCT   
  
  
- TACTGACTGC CACAACGTGT ATAGTTTTTC GTAAATACGG GATCACGGTA AAATAATAAA ATATAGAATT   
  
  
- AAAATCTATA AAAATAAAAA TAAATTACTA AATATAAACT TGCAATTAAA AGTTATATAG TATAACATAG   
  
  
- AAAATATCGG TGCAAATAAG TTTATATAAA AAATATAAAT ATTTATAAAA AAGATTTTAT ACTAAGCTCT   
  
  
- TACGACTGTA CACTTAGCGT ACCCCCATCC CGTTCGGTAG AACCTGGGTA ATTTGAAGTT TAAGGTACTA   
  
  
- ACTGATCCTG AACGGGTTTT TAAAGTAATG GGGTCTACGG GTGTACACTT ACGTCAGAGA CTGTCTCATC   
  
  
- CACCCTTATT TGGTTTTACG TAAATAAATA AAAAGTTCTA AAATTAATAA TAAGTGGATT TTAGGAAGTA   
  
  
- TCCCTAATTT TATAATCTGG TAACCTAAAC GAGAGGAGTT ATCCCAACTT GAAACTAATC ATGCAAATAT   
  
  
- AATAAAAAAA GGAAAAAGCA GTTTTACGTA AGACAGTGTT TTAATTACAT CAGTTCCTTA ACTGTTCACT   
  
  
- CTTCTCTCAG TGAAATTTAC TAAGTCGACA GGTGTAATTT CTATTCAGTG TAAACACCAG GTATGAGGGG   
  
  
- TTCTGAAATT AAACTTAAAT CGAGTCTGAA AAGCATAAAA TAAACACAAA AAGATAAATT CGATAGTTAT   
  
  
- GTAAATATTT ATTCACTAAA ATTATATTTT CTAGTTCATT TTTTGTTTAT TTCATGTATT TTATAAAAAT   
  
  
- AAGTTAATGT ATAACATAGT TTAAAAGTTC TCGTTTTAAA TATCGTAAAA AGTTACAAAA CCTTTAGTTT   
  
  
- TTTCATTATA TAATAGTCAA GTGATCAATT TAACCACTAA CTTGGTATCT ACTACAGTAT TATATATAAA   
  
  
- ATATAAATAA TTTTAATTTT TATAAATATT CTTTAAAATT TATATACTCT TTAATTTTCA GTTATTATAA   
  
  
- AAAAAGAAGT AGAAATTATA ATAGTTTACT TATATATATA TATATATTTA CATAACTATA AAATTTTAAA   
  
  
- ACAATTTATT TTATATATAT TATACTTCTA TAAATAACTT TCAATAAATT AAATGGATAT ATATATAATT   
  
  
- TTCAATAAAT ACCAACATTT TTTATAATGT AATAAAATAA ATATAATAAT TATAATGTAA TAAAATAAAT   
  
  
- ATAATAAATA TTAAAGTAAT AATATTTTTC AAACTTTTAT AATAACGATA TATTAAACAA CAACACACTT   
  
  
- CTAGCCGTTT TTTTGTCCTC ATCGTCACAC CACAGCAGGA ACGGGGAACT TGACCTCCAA CCAAAGGGTA   
  
  
- AGGAGGGAGG GTGCTTAGGG TGTACATTTC GTTTATTTTC TCGAAATGTA TGTGTACTAA TTACATAAAA   
  
  
- ATAACAGTTA TTTAAAATAT TATTTTGAAT ATTTAGAATA TTTAAATTAT CCTTTTTTTT TTCAGAAGAT   
  
  
- CTTAACTAAA ATATTATGTG GTATGGTTCT AGTGTACTTA ACGTACACAG CCACCTGTTC CAACGGTTTT   
  
  
- AAGGAATAAG GATATGTCTT AGAAAAGTAA CAATATAATA GTATCAGCTT TGGGTCATCA TATATTTCTC   
  
  
- CAATGCTCAC CTCCTACCTA TACTTTATTT ATTCTCCGGA ATCTCTAGTC CCACACTCTG TACCTAAACC   
  
  
- GTTTACCACT CTCTAGATCA CTCG

+     TATA-box

| Site Name | Organism | Position | Strand | Matrix score. | sequence | function |
| --- | --- | --- | --- | --- | --- | --- |
| TATA-box | Arabidopsis thaliana | 3702 | - | 4 | TATA | core promoter element around -30 of transcription start |
| TATA-box | Brassica oleracea | 3701 | + | 6 | ATATAA | core promoter element around -30 of transcription start |
| TATA-box | Arabidopsis thaliana | 3700 | - | 6 | TATATA | core promoter element around -30 of transcription start |
| TATA-box | Arabidopsis thaliana | 3673 | - | 4 | TATA | core promoter element around -30 of transcription start |
| TATA-box | Arabidopsis thaliana | 3672 | - | 5 | TATAA | core promoter element around -30 of transcription start |
| TATA-box | Arabidopsis thaliana | 3652 | - | 4 | TATA | core promoter element around -30 of transcription start |
| TATA-box | Arabidopsis thaliana | 3581 | - | 4 | TATA | core promoter element around -30 of transcription start |
| TATA-box | Arabidopsis thaliana | 3580 | - | 5 | TATAA | core promoter element around -30 of transcription start |
| TATA-box | Arabidopsis thaliana | 3538 | - | 4 | TATA | core promoter element around -30 of transcription start |
| TATA-box | Arabidopsis thaliana | 3537 | - | 5 | TATAA | core promoter element around -30 of transcription start |
| TATA-box | Arabidopsis thaliana | 3529 | - | 4 | TATA | core promoter element around -30 of transcription start |
| TATA-box | Arabidopsis thaliana | 3528 | - | 5 | TATAA | core promoter element around -30 of transcription start |
| TATA-box | Arabidopsis thaliana | 3517 | - | 4 | TATA | core promoter element around -30 of transcription start |
| TATA-box | Arabidopsis thaliana | 3516 | - | 5 | TATAA | core promoter element around -30 of transcription start |
| TATA-box | Arabidopsis thaliana | 3340 | - | 4 | TATA | core promoter element around -30 of transcription start |
| TATA-box | Brassica oleracea | 3339 | + | 6 | ATATAA | core promoter element around -30 of transcription start |
| TATA-box | Arabidopsis thaliana | 3338 | - | 6 | TATATA | core promoter element around -30 of transcription start |
| TATA-box | Arabidopsis thaliana | 3312 | - | 4 | TATA | core promoter element around -30 of transcription start |
| TATA-box | Arabidopsis thaliana | 3311 | - | 5 | TATAA | core promoter element around -30 of transcription start |
| TATA-box | Brassica napus | 3310 | + | 6 | ATTATA | core promoter element around -30 of transcription start |
| TATA-box | Arabidopsis thaliana | 3298 | - | 4 | TATA | core promoter element around -30 of transcription start |
| TATA-box | Arabidopsis thaliana | 3297 | - | 5 | TATAA | core promoter element around -30 of transcription start |
| TATA-box | Helianthus annuus | 3296 | - | 6 | TATAAA | core promoter element around -30 of transcription start |
| TATA-box | Brassica juncea | 3295 | - | 7 | TATAAAT | core promoter element around -30 of transcription start |
| TATA-box | Daucus carota | 3294 | - | 8 | TATAAATA | core promoter element around -30 of transcription start |
| TATA-box | Arabidopsis thaliana | 3289 | - | 4 | TATA | core promoter element around -30 of transcription start |
| TATA-box | Arabidopsis thaliana | 3288 | - | 5 | TATAA | core promoter element around -30 of transcription start |
| TATA-box | Helianthus annuus | 3287 | - | 6 | TATAAA | core promoter element around -30 of transcription start |
| TATA-box | Brassica juncea | 3286 | - | 7 | TATAAAT | core promoter element around -30 of transcription start |
| TATA-box | Arabidopsis thaliana | 3261 | - | 4 | TATA | core promoter element around -30 of transcription start |
| TATA-box | Arabidopsis thaliana | 3260 | - | 5 | TATAA | core promoter element around -30 of transcription start |
| TATA-box | Daucus carota | 3285 | - | 8 | TATAAATA | core promoter element around -30 of transcription start |
| TATA-box | Helianthus annuus | 3259 | - | 6 | TATAAA | core promoter element around -30 of transcription start |
| TATA-box | Brassica juncea | 3258 | - | 7 | TATAAAT | core promoter element around -30 of transcription start |
| TATA-box | Brassica napus | 3212 | - | 6 | ATATAT | core promoter element around -30 of transcription start |
| TATA-box | Arabidopsis thaliana | 3211 | - | 6 | TATATA | core promoter element around -30 of transcription start |
| TATA-box | Brassica napus | 3210 | - | 6 | ATATAT | core promoter element around -30 of transcription start |
| TATA-box | Arabidopsis thaliana | 3209 | - | 6 | TATATA | core promoter element around -30 of transcription start |
| TATA-box | Daucus carota | 3257 | - | 8 | TATAAATA | core promoter element around -30 of transcription start |
| TATA-box | Arabidopsis thaliana | 3213 | - | 4 | TATA | core promoter element around -30 of transcription start |
| TATA-box | Pisum sativum | 3514 | - | 7 | TATAAAA | core promoter element around -30 of transcription start |
| TATA-box | Helianthus annuus | 3515 | - | 6 | TATAAA | core promoter element around -30 of transcription start |
| TATA-box | Pisum sativum | 3578 | - | 7 | TATAAAA | core promoter element around -30 of transcription start |
| TATA-box | Helianthus annuus | 3579 | - | 6 | TATAAA | core promoter element around -30 of transcription start |
| TATA-box | Brassica napus | 3051 | - | 6 | ATATAT | core promoter element around -30 of transcription start |
| TATA-box | Brassica napus | 3115 | - | 6 | ATATAT | core promoter element around -30 of transcription start |
| TATA-box | Arabidopsis thaliana | 3052 | - | 4 | TATA | core promoter element around -30 of transcription start |
| TATA-box | Arabidopsis thaliana | 3124 | - | 4 | TATA | core promoter element around -30 of transcription start |
| TATA-box | Arabidopsis thaliana | 3035 | - | 5 | TATAA | core promoter element around -30 of transcription start |
| TATA-box | Brassica napus | 3117 | - | 6 | ATATAT | core promoter element around -30 of transcription start |
| TATA-box | Helianthus annuus | 3034 | - | 6 | TATAAA | core promoter element around -30 of transcription start |
| TATA-box | Arabidopsis thaliana | 3167 | - | 4 | TATA | core promoter element around -30 of transcription start |
| TATA-box | Brassica juncea | 3033 | - | 7 | TATAAAT | core promoter element around -30 of transcription start |
| TATA-box | Brassica napus | 3111 | - | 6 | ATATAT | core promoter element around -30 of transcription start |
| TATA-box | Daucus carota | 3032 | - | 8 | TATAAATA | core promoter element around -30 of transcription start |
| TATA-box | Brassica napus | 3164 | - | 6 | ATATAT | core promoter element around -30 of transcription start |
| TATA-box | Arabidopsis thaliana | 3010 | - | 5 | TATAA | core promoter element around -30 of transcription start |
| TATA-box | Arabidopsis thaliana | 3122 | - | 6 | TATATA | core promoter element around -30 of transcription start |
| TATA-box | Arabidopsis thaliana | 3011 | - | 4 | TATA | core promoter element around -30 of transcription start |
| TATA-box | Brassica napus | 3113 | - | 6 | ATATAT | core promoter element around -30 of transcription start |
| TATA-box | Arabidopsis thaliana | 3046 | - | 8 | TATTTAAA | core promoter element around -30 of transcription start |
| TATA-box | Arabidopsis thaliana | 3120 | - | 6 | TATATA | core promoter element around -30 of transcription start |
| TATA-box | Arabidopsis thaliana | 3036 | - | 4 | TATA | core promoter element around -30 of transcription start |
| TATA-box | Arabidopsis thaliana | 3116 | - | 6 | TATATA | core promoter element around -30 of transcription start |
| TATA-box | Pisum sativum | 3008 | - | 7 | TATAAAA | core promoter element around -30 of transcription start |
| TATA-box | Brassica napus | 3208 | - | 6 | ATATAT | core promoter element around -30 of transcription start |
| TATA-box | Helianthus annuus | 3009 | - | 6 | TATAAA | core promoter element around -30 of transcription start |
| TATA-box | Brassica napus | 3121 | - | 6 | ATATAT | core promoter element around -30 of transcription start |
| TATA-box | Brassica napus | 3003 | - | 6 | ATATAT | core promoter element around -30 of transcription start |
| TATA-box | Arabidopsis thaliana | 3163 | - | 6 | TATATA | core promoter element around -30 of transcription start |
| TATA-box | Arabidopsis thaliana | 3004 | - | 4 | TATA | core promoter element around -30 of transcription start |
| TATA-box | Arabidopsis thaliana | 3114 | - | 6 | TATATA | core promoter element around -30 of transcription start |
| TATA-box | Arabidopsis thaliana | 2909 | - | 5 | TATAA | core promoter element around -30 of transcription start |
| TATA-box | Brassica oleracea | 3166 | + | 6 | ATATAA | core promoter element around -30 of transcription start |
| TATA-box | Arabidopsis thaliana | 2910 | - | 4 | TATA | core promoter element around -30 of transcription start |
| TATA-box | Arabidopsis thaliana | 3118 | - | 6 | TATATA | core promoter element around -30 of transcription start |
| TATA-box | Brassica napus | 3001 | - | 6 | ATATAT | core promoter element around -30 of transcription start |
| TATA-box | Arabidopsis thaliana | 3165 | - | 6 | TATATA | core promoter element around -30 of transcription start |
| TATA-box | Arabidopsis thaliana | 3112 | - | 6 | TATATA | core promoter element around -30 of transcription start |
| TATA-box | Arabidopsis thaliana | 3207 | - | 6 | TATATA | core promoter element around -30 of transcription start |
| TATA-box | Brassica napus | 3119 | - | 6 | ATATAT | core promoter element around -30 of transcription start |
| TATA-box | Brassica napus | 2947 | - | 6 | ATATAT | core promoter element around -30 of transcription start |
| TATA-box | Arabidopsis thaliana | 2948 | - | 4 | TATA | core promoter element around -30 of transcription start |
| TATA-box | Brassica juncea | 2907 | - | 7 | TATAAAT | core promoter element around -30 of transcription start |
| TATA-box | Brassica oleracea | 3123 | + | 6 | ATATAA | core promoter element around -30 of transcription start |
| TATA-box | Brassica napus | 3162 | - | 6 | ATATAT | core promoter element around -30 of transcription start |
| TATA-box | Helianthus annuus | 2908 | - | 6 | TATAAA | core promoter element around -30 of transcription start |
| TATA-box | Arabidopsis thaliana | 2824 | - | 4 | TATA | core promoter element around -30 of transcription start |
| TATA-box | Arabidopsis thaliana | 3002 | - | 6 | TATATA | core promoter element around -30 of transcription start |
| TATA-box | Oryza sativa | 2854 | + | 8 | TACATAAA | core promoter element around -30 of transcription start |
| TATA-box | Brassica napus | 1051 | + | 6 | ATTATA | core promoter element around -30 of transcription start |
| TATA-box | Arabidopsis thaliana | 1612 | + | 9 | ccTATAAAaa | core promoter element around -30 of transcription start |
| TATA-box | Arabidopsis thaliana | 1343 | - | 5 | TATAA | core promoter element around -30 of transcription start |
| TATA-box | Helianthus annuus | 2242 | - | 6 | TATAAA | core promoter element around -30 of transcription start |
| TATA-box | Arabidopsis thaliana | 1187 | + | 4 | TATA | core promoter element around -30 of transcription start |
| TATA-box | Arabidopsis thaliana | 2160 | - | 5 | TATAA | core promoter element around -30 of transcription start |
| TATA-box | Arabidopsis thaliana | 1490 | - | 5 | TATAA | core promoter element around -30 of transcription start |
| TATA-box | Arabidopsis thaliana | 2278 | - | 5 | TATAA | core promoter element around -30 of transcription start |
| TATA-box | Arabidopsis thaliana | 47 | + | 8 | TATTTAAA | core promoter element around -30 of transcription start |
| TATA-box | Arabidopsis thaliana | 2026 | - | 4 | TATA | core promoter element around -30 of transcription start |
| TATA-box | Brassica napus | 1392 | + | 6 | ATTATA | core promoter element around -30 of transcription start |
| TATA-box | Helianthus annuus | 2271 | - | 6 | TATAAA | core promoter element around -30 of transcription start |
| TATA-box | Arabidopsis thaliana | 1326 | - | 5 | TATAA | core promoter element around -30 of transcription start |
| TATA-box | Arabidopsis thaliana | 2202 | - | 4 | TATA | core promoter element around -30 of transcription start |
| TATA-box | Arabidopsis thaliana | 1557 | + | 4 | TATA | core promoter element around -30 of transcription start |
| TATA-box | Helianthus annuus | 2803 | - | 6 | TATAAA | core promoter element around -30 of transcription start |
| TATA-box | Arabidopsis thaliana | 1052 | - | 5 | TATAA | core promoter element around -30 of transcription start |
| TATA-box | Arabidopsis thaliana | 1638 | - | 5 | TATAA | core promoter element around -30 of transcription start |
| TATA-box | Arabidopsis thaliana | 1373 | - | 5 | TATAA | core promoter element around -30 of transcription start |
| TATA-box | Arabidopsis thaliana | 2264 | - | 4 | TATA | core promoter element around -30 of transcription start |
| TATA-box | Arabidopsis thaliana | 1053 | + | 4 | TATA | core promoter element around -30 of transcription start |
| TATA-box | Helianthus annuus | 2200 | - | 6 | TATAAA | core promoter element around -30 of transcription start |
| TATA-box | Arabidopsis thaliana | 2587 | - | 4 | TATA | core promoter element around -30 of transcription start |
| TATA-box | Pisum sativum | 2158 | - | 7 | TATAAAA | core promoter element around -30 of transcription start |
| TATA-box | Brassica juncea | 2276 | - | 7 | TATAAAT | core promoter element around -30 of transcription start |
| TATA-box | Arabidopsis thaliana | 2225 | - | 4 | TATA | core promoter element around -30 of transcription start |
| TATA-box | Arabidopsis thaliana | 2805 | - | 4 | TATA | core promoter element around -30 of transcription start |
| TATA-box | Pisum sativum | 1636 | - | 7 | TATAAAA | core promoter element around -30 of transcription start |
| TATA-box | Arabidopsis thaliana | 2244 | - | 4 | TATA | core promoter element around -30 of transcription start |
| TATA-box | Arabidopsis thaliana | 2198 | - | 9 | taTATAAAtc | core promoter element around -30 of transcription start |
| TATA-box | Helianthus annuus | 2585 | - | 6 | TATAAA | core promoter element around -30 of transcription start |
| TATA-box | Arabidopsis thaliana | 2084 | - | 5 | TATAA | core promoter element around -30 of transcription start |
| TATA-box | Arabidopsis thaliana | 2273 | - | 4 | TATA | core promoter element around -30 of transcription start |
| TATA-box | Brassica napus | 2224 | - | 6 | ATATAT | core promoter element around -30 of transcription start |
| TATA-box | Arabidopsis thaliana | 2804 | - | 5 | TATAA | core promoter element around -30 of transcription start |
| TATA-box | Arabidopsis thaliana | 1639 | + | 4 | TATA | core promoter element around -30 of transcription start |
| TATA-box | Pisum sativum | 2270 | - | 7 | TATAAAA | core promoter element around -30 of transcription start |
| TATA-box | Arabidopsis thaliana | 2201 | - | 5 | TATAA | core promoter element around -30 of transcription start |
| TATA-box | Brassica juncea | 2802 | - | 7 | TATAAAT | core promoter element around -30 of transcription start |
| TATA-box | Helianthus annuus | 2159 | - | 6 | TATAAA | core promoter element around -30 of transcription start |
| TATA-box | Helianthus annuus | 2277 | - | 6 | TATAAA | core promoter element around -30 of transcription start |
| TATA-box | Pisum sativum | 2241 | - | 7 | TATAAAA | core promoter element around -30 of transcription start |
| TATA-box | Brassica oleracea | 2823 | + | 6 | ATATAA | core promoter element around -30 of transcription start |
| TATA-box | Arabidopsis thaliana | 1595 | + | 4 | TATA | core promoter element around -30 of transcription start |
| TATA-box | Arabidopsis thaliana | 2243 | - | 5 | TATAA | core promoter element around -30 of transcription start |
| TATA-box | Arabidopsis thaliana | 2161 | - | 4 | TATA | core promoter element around -30 of transcription start |
| TATA-box | Arabidopsis thaliana | 2279 | - | 4 | TATA | core promoter element around -30 of transcription start |
| TATA-box | Arabidopsis thaliana | 2055 | + | 8 | TATTTAAA | core promoter element around -30 of transcription start |
| TATA-box | Arabidopsis thaliana | 2272 | - | 5 | TATAA | core promoter element around -30 of transcription start |
| TATA-box | Brassica juncea | 2199 | - | 7 | TATAAAT | core promoter element around -30 of transcription start |
| TATA-box | Arabidopsis thaliana | 2586 | - | 5 | TATAA | core promoter element around -30 of transcription start |
| TATA-box | Helianthus annuus | 1637 | - | 6 | TATAAA | core promoter element around -30 of transcription start |
| TATA-box | Brassica napus | 2263 | - | 6 | ATATAT | core promoter element around -30 of transcription start |
| TATA-box | Arabidopsis thaliana | 2085 | - | 4 | TATA | core promoter element around -30 of transcription start |
| TATA-box | Daucus carota | 2275 | - | 8 | TATAAATA | core promoter element around -30 of transcription start |
| TATA-box | Helianthus annuus | 1555 | - | 6 | TATAAA | core promoter element around -30 of transcription start |
| TATA-box | Arabidopsis thaliana | 166 | + | 4 | TATA | core promoter element around -30 of transcription start |
| TATA-box | Brassica juncea | 1488 | - | 7 | TATAAAT | core promoter element around -30 of transcription start |
| TATA-box | Pisum sativum | 1341 | - | 7 | TATAAAA | core promoter element around -30 of transcription start |
| TATA-box | Helianthus annuus | 1593 | - | 6 | TATAAA | core promoter element around -30 of transcription start |
| TATA-box | Brassica napus | 1030 | + | 6 | ATATAT | core promoter element around -30 of transcription start |
| TATA-box | Pisum sativum | 1371 | - | 7 | TATAAAA | core promoter element around -30 of transcription start |
| TATA-box | Arabidopsis thaliana | 1106 | + | 9 | ccTATAAAaa | core promoter element around -30 of transcription start |
| TATA-box | Arabidopsis thaliana | 1553 | - | 9 | ccTATAAAaa | core promoter element around -30 of transcription start |
| TATA-box | Arabidopsis thaliana | 207 | - | 5 | TATAA | core promoter element around -30 of transcription start |
| TATA-box | Arabidopsis thaliana | 1394 | + | 4 | TATA | core promoter element around -30 of transcription start |
| TATA-box | Arabidopsis thaliana | 1327 | + | 4 | TATA | core promoter element around -30 of transcription start |
| TATA-box | Brassica juncea | 1592 | - | 7 | TATAAAT | core promoter element around -30 of transcription start |
| TATA-box | Arabidopsis thaliana | 208 | + | 4 | TATA | core promoter element around -30 of transcription start |
| TATA-box | Arabidopsis thaliana | 1374 | + | 4 | TATA | core promoter element around -30 of transcription start |
| TATA-box | Helianthus annuus | 1325 | - | 6 | TATAAA | core promoter element around -30 of transcription start |
| TATA-box | Arabidopsis thaliana | 1556 | - | 5 | TATAA | core promoter element around -30 of transcription start |
| TATA-box | Arabidopsis thaliana | 1031 | + | 4 | TATA | core promoter element around -30 of transcription start |
| TATA-box | Helianthus annuus | 1489 | - | 6 | TATAAA | core promoter element around -30 of transcription start |
| TATA-box | Helianthus annuus | 1342 | - | 6 | TATAAA | core promoter element around -30 of transcription start |
| TATA-box | Arabidopsis thaliana | 1594 | - | 5 | TATAA | core promoter element around -30 of transcription start |
| TATA-box | Arabidopsis thaliana | 510 | + | 4 | TATA | core promoter element around -30 of transcription start |
| TATA-box | Arabidopsis thaliana | 1344 | + | 4 | TATA | core promoter element around -30 of transcription start |
| TATA-box | Brassica oleracea | 1145 | + | 6 | ATATAA | core promoter element around -30 of transcription start |
| TATA-box | Arabidopsis thaliana | 1491 | + | 4 | TATA | core promoter element around -30 of transcription start |
| TATA-box | Arabidopsis thaliana | 839 | + | 9 | ccTATAAAaa | core promoter element around -30 of transcription start |
| TATA-box | Arabidopsis thaliana | 1393 | - | 5 | TATAA | core promoter element around -30 of transcription start |
| TATA-box | Arabidopsis thaliana | 1146 | + | 4 | TATA | core promoter element around -30 of transcription start |
| TATA-box | Pisum sativum | 1554 | - | 7 | TATAAAA | core promoter element around -30 of transcription start |
| TATA-box | Pisum sativum | 205 | - | 7 | TATAAAA | core promoter element around -30 of transcription start |
| TATA-box | Helianthus annuus | 1372 | - | 6 | TATAAA | core promoter element around -30 of transcription start |
| TATA-box | Helianthus annuus | 206 | - | 6 | TATAAA | core promoter element around -30 of transcription start |
| TATA-box | Daucus carota | 1487 | - | 8 | TATAAATA | core promoter element around -30 of transcription start |

>PlantCARE\_32120   
+ AATCATTAAA GTCTAATTCT TCAAAATTCC GAATTCTAAA TTTAGCTATT TAAAATTCCA ATTGTCCTAT   
  
  
+ CTCCGAACTT AATTTTCAGT TTCCCATGCT CCAATTCCCG TATTTACCCC GCTACTTATT ATTATCATTA   
  
  
+ TCATTATCAC CTTATTCATT ATTTCTATAA ATTCCGCCTT CGAATGATTT CTAAGATCTC CAATTTTTAT   
  
  
+ ACATCAACTT TCATAATTTC TACGTCTCAA ATAACATACG ATTCTGATTC CTTTCAAAAT TTAACTCCCA   
  
  
+ TAATTCCAAT TTTCGTAACT TAATTTTTCT TAAAAATCCC GAACTTTCAA AATCCCAATT TTCTTTCAAA   
  
  
+ TTTAATTTCT AAATGTTCTC ATTCTTACAT CTAATTCCTA AGAATCCAAT TTTCAAATAA TCTCTAAGAT   
  
  
+ TCCGATTCTC AAATGAATTT CAAAAATCCA GTTTCCCTTC GAACAATTTT AATCCCTGTT TAGTGATGCA   
  
  
+ TGTAATATGT TTTGTGCTCT ATATGGTGTA CTAACCCTCT CTATTGATAG CGCATGGTGG TTCGTGGCCC   
  
  
+ AGGTATGTAC CCATTTTCTC TCTAATCGTT GTCTATGCAT GTCTCGATTC TTATGTGTGC ACGATCATTC   
  
  
+ AGGATATTCA TTGATTTACT CATCAATTGT CACGGCAGCT TCATTTTATT AGTAGAGACC CGACTTTAGG   
  
  
+ GACTTAGAGG GGTGCTACGG TCCTTACCGT ACCTTCCTGA TAAGTAACCT GACCCCCGAA CCCGATCCGG   
  
  
+ TTTTTCACAG ACCATCTTTT CCAAAATAAG GAGTCACACT TAGGGTTTTT CTTTCTTATT TTGTTTACCC   
  
  
+ TTTAAAAATA AAACAAAAAT AAGTGGCGAC TCCAAGTCAT TTTTCTTAAT CAATAAAGAT CATTTTTTCG   
  
  
+ AAAACGAAAA TCGAGCTCGC CATCGAGTGG GAAACGCATT GAGCCGAAAT GCGGGGTCCA TAGGTATGTT   
  
  
+ TTTTATTTAT GAGATAAAAA GTAAATAATA TTTTTTTCGG GTTTAAAAAA TATATTATTG ATTACTTGTA   
  
  
+ ATTATATGAG CCTCGTTTGT ACTTATTATT AAATACATAT TCAAGGTAAA ATTATCCTTT AAAAAATTCT   
  
  
+ TAGGCCGAGG GAACCTTCCT ATTAATATAA GTCATCCAGC CCACCTTATG GGGGTAATTC TCCCCATATA   
  
  
+ CCATGGGTCC AAATCGGCGG CCCGGTCAGT TCAACGACAG TACGATGGCA CTCTGAACCC TGTGCTGCAT   
  
  
+ CTGCTCACAA AAAGTACACC ACAGTTGTCT TTCACTCTCT CTGCCCATTC ACTATGCAAA CACGTTTATA   
  
  
+ AAATTGGTTA TTTTATAAAC GTTTCTCTTT CAAATGGTTA TTTTATATTA GTAACAAAAA TATTATAATA   
  
  
+ ATAAAAAAAT CAATATTACG TAATTGCTAA AAATGAAATT ACATCATGTT GTAAGATATT TCATTTGCCT   
  
  
+ ATTAATAATT TTTTATTATT TATAAAATTG GGTTGAATTT CAATATTTTT TAAAATAAGT CTTATTTATT   
  
  
+ TAAGTGAAAT TTTTTTTATA GATAAAGTCA CAAAAATCAT TTTTCTAAAT AATTTATAAA AAGATACTTT   
  
  
+ TCCAATAAAA ATTAAATTAA AATGCTTTTA TAAATAAAAT TGTAACAATT CATTCCATAG CAAAGGTAAA   
  
  
+ TAAAGCCATT TTCTTATCAA TTAAATCAAG CTTCTCTTTT TTATGAAATT GTTTTTAATA AATTCTTTTA   
  
  
+ GTAACAATAA GATTTTGTTC ATATTTTAAT ACATCTCCTT GGCAATTGGG CAAAGATGGC TGGTAGTGAC   
  
  
+ ATGAATCCAA GCATGAATTA GTCCGGGCGT TGTTACTTGC TACAAGGGTT ATGAGCATGG AAGGAAAAAT   
  
  
+ AATTAAAAAA TAAAAAAAAT TAAATTACTT CAATCACAAA GTTGAAAATA ATAAAATTGT GTTTGGAAGT   
  
  
+ ATTTGTACTT AAAGTTTTTT AGTAAAAGTG TTTCCCTAAA AGGTGTTTTT TTTAGAGAAA GCAGCTATAA   
  
  
+ AATGTTTTTT CAAAAATATT TTTTTATTTA AAAACACTTT CTTAAATCAC TTGTTATAAG TCACCTTAGA   
  
  
+ ATGACTGACG GTGTTGCACA TATCAAAAAG CATTTATGCC CTAGTGCCAT TTTATTATTT TATATCTTAA   
  
  
+ TTTTAGATAT TTTTATTTTT ATTTAATGAT TTATATTTGA ACGTTAATTT TCAATATATC ATATTGTATC   
  
  
+ TTTTATAGCC ACGTTTATTC AAATATATTT TTTATATTTA TAAATATTTT TTCTAAAATA TGATTCGAGA   
  
  
+ ATGCTGACAT GTGAATCGCA TGGGGGTAGG GCAAGCCATC TTGGACCCAT TAAACTTCAA ATTCCATGAT   
  
  
+ TGACTAGGAC TTGCCCAAAA ATTTCATTAC CCCAGATGCC CACATGTGAA TGCAGTCTCT GACAGAGTAG   
  
  
+ GTGGGAATAA ACCAAAATGC ATTTATTTAT TTTTCAAGAT TTTAATTATT ATTCACCTAA AATCCTTCAT   
  
  
+ AGGGATTAAA ATATTAGACC ATTGGATTTG CTCTCCTCAA TAGGGTTGAA CTTTGATTAG TACGTTTATA   
  
  
+ TTATTTTTTT CCTTTTTCGT CAAAATGCAT TCTGTCACAA AATTAATGTA GTCAAGGAAT TGACAAGTGA   
  
  
+ GAAGAGAGTC ACTTTAAATG ATTCAGCTGT CCACATTAAA GATAAGTCAC ATTTGTGGTC CATACTCCCC   
  
  
+ AAGACTTTAA TTTGAATTTA GCTCAGACTT TTCGTATTTT ATTTGTGTTT TTCTATTTAA GCTATCAATA   
  
  
+ CATTTATAAA TAAGTGATTT TAATATAAAA GATCAAGTAA AAAACAAATA AAGTACATAA AATATTTTTA   
  
  
+ TTCAATTACA TATTGTATCA AATTTTCAAG AGCAAAATTT ATAGCATTTT TCAATGTTTT GGAAATCAAA   
  
  
+ AAAGTAATAT ATTATCAGTT CACTAGTTAA ATTGGTGATT GAACCATAGA TGATGTCATA ATATATATTT   
  
  
+ TATATTTATT AAAATTAAAA ATATTTATAA GAAATTTTAA ATATATGAGA AATTAAAAGT CAATAATATT   
  
  
+ TTTTTCTTCA TCTTTAATAT TATCAAATGA ATATATATAT ATATATAAAT GTATTGATAT TTTAAAATTT   
  
  
+ TGTTAAATAA AATATATATA ATATGAAGAT ATTTATTGAA AGTTATTTAA TTTACCTATA TATATATTAA   
  
  
+ AAGTTATTTA TGGTTGTAAA AAATATTACA TTATTTTATT TATATTATTA ATATTACATT ATTTTATTTA   
  
  
+ TATTATTTAT AATTTCATTA TTATAAAAAG TTTGAAAATA TTATTGCTAT ATAATTTGTT GTTGTGTGAA   
  
  
+ GATCGGCAAA AAAACAGGAG TAGCAGTGTG GTGTCGTCCT TGCCCCTTGA ACTGGAGGTT GGTTTCCCAT   
  
  
+ TCCTCCCTCC CACGAATCCC ACATGTAAAG CAAATAAAAG AGCTTTACAT ACACATGATT AATGTATTTT   
  
  
+ TATTGTCAAT AAATTTTATA ATAAAACTTA TAAATCTTAT AAATTTAATA GGAAAAAAAA AAGTCTTCTA   
  
  
+ GAATTGATTT TATAATACAC CATACCAAGA TCACATGAAT TGCATGTGTC GGTGGACAAG GTTGCCAAAA   
  
  
+ TTCCTTATTC CTATACAGAA TCTTTTCATT GTTATATTAT CATAGTCGAA ACCCAGTAGT ATATAAAGAG   
  
  
+ GTTACGAGTG GAGGATGGAT ATGAAATAAA TAAGAGGCCT TAGAGATCAG GGTGTGAGAC ATGGATTTGG   
  
  
+ CAAATGGTGA GAGATCTAGT GAGC  

- TTAGTAATTT CAGATTAAGA AGTTTTAAGG CTTAAGATTT AAATCGATAA ATTTTAAGGT TAACAGGATA   
  
  
- GAGGCTTGAA TTAAAAGTCA AAGGGTACGA GGTTAAGGGC ATAAATGGGG CGATGAATAA TAATAGTAAT   
  
  
- AGTAATAGTG GAATAAGTAA TAAAGATATT TAAGGCGGAA GCTTACTAAA GATTCTAGAG GTTAAAAATA   
  
  
- TGTAGTTGAA AGTATTAAAG ATGCAGAGTT TATTGTATGC TAAGACTAAG GAAAGTTTTA AATTGAGGGT   
  
  
- ATTAAGGTTA AAAGCATTGA ATTAAAAAGA ATTTTTAGGG CTTGAAAGTT TTAGGGTTAA AAGAAAGTTT   
  
  
- AAATTAAAGA TTTACAAGAG TAAGAATGTA GATTAAGGAT TCTTAGGTTA AAAGTTTATT AGAGATTCTA   
  
  
- AGGCTAAGAG TTTACTTAAA GTTTTTAGGT CAAAGGGAAG CTTGTTAAAA TTAGGGACAA ATCACTACGT   
  
  
- ACATTATACA AAACACGAGA TATACCACAT GATTGGGAGA GATAACTATC GCGTACCACC AAGCACCGGG   
  
  
- TCCATACATG GGTAAAAGAG AGATTAGCAA CAGATACGTA CAGAGCTAAG AATACACACG TGCTAGTAAG   
  
  
- TCCTATAAGT AACTAAATGA GTAGTTAACA GTGCCGTCGA AGTAAAATAA TCATCTCTGG GCTGAAATCC   
  
  
- CTGAATCTCC CCACGATGCC AGGAATGGCA TGGAAGGACT ATTCATTGGA CTGGGGGCTT GGGCTAGGCC   
  
  
- AAAAAGTGTC TGGTAGAAAA GGTTTTATTC CTCAGTGTGA ATCCCAAAAA GAAAGAATAA AACAAATGGG   
  
  
- AAATTTTTAT TTTGTTTTTA TTCACCGCTG AGGTTCAGTA AAAAGAATTA GTTATTTCTA GTAAAAAAGC   
  
  
- TTTTGCTTTT AGCTCGAGCG GTAGCTCACC CTTTGCGTAA CTCGGCTTTA CGCCCCAGGT ATCCATACAA   
  
  
- AAAATAAATA CTCTATTTTT CATTTATTAT AAAAAAAGCC CAAATTTTTT ATATAATAAC TAATGAACAT   
  
  
- TAATATACTC GGAGCAAACA TGAATAATAA TTTATGTATA AGTTCCATTT TAATAGGAAA TTTTTTAAGA   
  
  
- ATCCGGCTCC CTTGGAAGGA TAATTATATT CAGTAGGTCG GGTGGAATAC CCCCATTAAG AGGGGTATAT   
  
  
- GGTACCCAGG TTTAGCCGCC GGGCCAGTCA AGTTGCTGTC ATGCTACCGT GAGACTTGGG ACACGACGTA   
  
  
- GACGAGTGTT TTTCATGTGG TGTCAACAGA AAGTGAGAGA GACGGGTAAG TGATACGTTT GTGCAAATAT   
  
  
- TTTAACCAAT AAAATATTTG CAAAGAGAAA GTTTACCAAT AAAATATAAT CATTGTTTTT ATAATATTAT   
  
  
- TATTTTTTTA GTTATAATGC ATTAACGATT TTTACTTTAA TGTAGTACAA CATTCTATAA AGTAAACGGA   
  
  
- TAATTATTAA AAAATAATAA ATATTTTAAC CCAACTTAAA GTTATAAAAA ATTTTATTCA GAATAAATAA   
  
  
- ATTCACTTTA AAAAAAATAT CTATTTCAGT GTTTTTAGTA AAAAGATTTA TTAAATATTT TTCTATGAAA   
  
  
- AGGTTATTTT TAATTTAATT TTACGAAAAT ATTTATTTTA ACATTGTTAA GTAAGGTATC GTTTCCATTT   
  
  
- ATTTCGGTAA AAGAATAGTT AATTTAGTTC GAAGAGAAAA AATACTTTAA CAAAAATTAT TTAAGAAAAT   
  
  
- CATTGTTATT CTAAAACAAG TATAAAATTA TGTAGAGGAA CCGTTAACCC GTTTCTACCG ACCATCACTG   
  
  
- TACTTAGGTT CGTACTTAAT CAGGCCCGCA ACAATGAACG ATGTTCCCAA TACTCGTACC TTCCTTTTTA   
  
  
- TTAATTTTTT ATTTTTTTTA ATTTAATGAA GTTAGTGTTT CAACTTTTAT TATTTTAACA CAAACCTTCA   
  
  
- TAAACATGAA TTTCAAAAAA TCATTTTCAC AAAGGGATTT TCCACAAAAA AAATCTCTTT CGTCGATATT   
  
  
- TTACAAAAAA GTTTTTATAA AAAAATAAAT TTTTGTGAAA GAATTTAGTG AACAATATTC AGTGGAATCT   
  
  
- TACTGACTGC CACAACGTGT ATAGTTTTTC GTAAATACGG GATCACGGTA AAATAATAAA ATATAGAATT   
  
  
- AAAATCTATA AAAATAAAAA TAAATTACTA AATATAAACT TGCAATTAAA AGTTATATAG TATAACATAG   
  
  
- AAAATATCGG TGCAAATAAG TTTATATAAA AAATATAAAT ATTTATAAAA AAGATTTTAT ACTAAGCTCT   
  
  
- TACGACTGTA CACTTAGCGT ACCCCCATCC CGTTCGGTAG AACCTGGGTA ATTTGAAGTT TAAGGTACTA   
  
  
- ACTGATCCTG AACGGGTTTT TAAAGTAATG GGGTCTACGG GTGTACACTT ACGTCAGAGA CTGTCTCATC   
  
  
- CACCCTTATT TGGTTTTACG TAAATAAATA AAAAGTTCTA AAATTAATAA TAAGTGGATT TTAGGAAGTA   
  
  
- TCCCTAATTT TATAATCTGG TAACCTAAAC GAGAGGAGTT ATCCCAACTT GAAACTAATC ATGCAAATAT   
  
  
- AATAAAAAAA GGAAAAAGCA GTTTTACGTA AGACAGTGTT TTAATTACAT CAGTTCCTTA ACTGTTCACT   
  
  
- CTTCTCTCAG TGAAATTTAC TAAGTCGACA GGTGTAATTT CTATTCAGTG TAAACACCAG GTATGAGGGG   
  
  
- TTCTGAAATT AAACTTAAAT CGAGTCTGAA AAGCATAAAA TAAACACAAA AAGATAAATT CGATAGTTAT   
  
  
- GTAAATATTT ATTCACTAAA ATTATATTTT CTAGTTCATT TTTTGTTTAT TTCATGTATT TTATAAAAAT   
  
  
- AAGTTAATGT ATAACATAGT TTAAAAGTTC TCGTTTTAAA TATCGTAAAA AGTTACAAAA CCTTTAGTTT   
  
  
- TTTCATTATA TAATAGTCAA GTGATCAATT TAACCACTAA CTTGGTATCT ACTACAGTAT TATATATAAA   
  
  
- ATATAAATAA TTTTAATTTT TATAAATATT CTTTAAAATT TATATACTCT TTAATTTTCA GTTATTATAA   
  
  
- AAAAAGAAGT AGAAATTATA ATAGTTTACT TATATATATA TATATATTTA CATAACTATA AAATTTTAAA   
  
  
- ACAATTTATT TTATATATAT TATACTTCTA TAAATAACTT TCAATAAATT AAATGGATAT ATATATAATT   
  
  
- TTCAATAAAT ACCAACATTT TTTATAATGT AATAAAATAA ATATAATAAT TATAATGTAA TAAAATAAAT   
  
  
- ATAATAAATA TTAAAGTAAT AATATTTTTC AAACTTTTAT AATAACGATA TATTAAACAA CAACACACTT   
  
  
- CTAGCCGTTT TTTTGTCCTC ATCGTCACAC CACAGCAGGA ACGGGGAACT TGACCTCCAA CCAAAGGGTA   
  
  
- AGGAGGGAGG GTGCTTAGGG TGTACATTTC GTTTATTTTC TCGAAATGTA TGTGTACTAA TTACATAAAA   
  
  
- ATAACAGTTA TTTAAAATAT TATTTTGAAT ATTTAGAATA TTTAAATTAT CCTTTTTTTT TTCAGAAGAT   
  
  
- CTTAACTAAA ATATTATGTG GTATGGTTCT AGTGTACTTA ACGTACACAG CCACCTGTTC CAACGGTTTT   
  
  
- AAGGAATAAG GATATGTCTT AGAAAAGTAA CAATATAATA GTATCAGCTT TGGGTCATCA TATATTTCTC   
  
  
- CAATGCTCAC CTCCTACCTA TACTTTATTT ATTCTCCGGA ATCTCTAGTC CCACACTCTG TACCTAAACC   
  
  
- GTTTACCACT CTCTAGATCA CTCG

+     TCA-element

| Site Name | Organism | Position | Strand | Matrix score. | sequence | function |
| --- | --- | --- | --- | --- | --- | --- |
| TCA-element | Nicotiana tabacum | 782 | + | 9 | CCATCTTTTT | cis-acting element involved in salicylic acid responsiveness |

>PlantCARE\_32120   
+ AATCATTAAA GTCTAATTCT TCAAAATTCC GAATTCTAAA TTTAGCTATT TAAAATTCCA ATTGTCCTAT   
  
  
+ CTCCGAACTT AATTTTCAGT TTCCCATGCT CCAATTCCCG TATTTACCCC GCTACTTATT ATTATCATTA   
  
  
+ TCATTATCAC CTTATTCATT ATTTCTATAA ATTCCGCCTT CGAATGATTT CTAAGATCTC CAATTTTTAT   
  
  
+ ACATCAACTT TCATAATTTC TACGTCTCAA ATAACATACG ATTCTGATTC CTTTCAAAAT TTAACTCCCA   
  
  
+ TAATTCCAAT TTTCGTAACT TAATTTTTCT TAAAAATCCC GAACTTTCAA AATCCCAATT TTCTTTCAAA   
  
  
+ TTTAATTTCT AAATGTTCTC ATTCTTACAT CTAATTCCTA AGAATCCAAT TTTCAAATAA TCTCTAAGAT   
  
  
+ TCCGATTCTC AAATGAATTT CAAAAATCCA GTTTCCCTTC GAACAATTTT AATCCCTGTT TAGTGATGCA   
  
  
+ TGTAATATGT TTTGTGCTCT ATATGGTGTA CTAACCCTCT CTATTGATAG CGCATGGTGG TTCGTGGCCC   
  
  
+ AGGTATGTAC CCATTTTCTC TCTAATCGTT GTCTATGCAT GTCTCGATTC TTATGTGTGC ACGATCATTC   
  
  
+ AGGATATTCA TTGATTTACT CATCAATTGT CACGGCAGCT TCATTTTATT AGTAGAGACC CGACTTTAGG   
  
  
+ GACTTAGAGG GGTGCTACGG TCCTTACCGT ACCTTCCTGA TAAGTAACCT GACCCCCGAA CCCGATCCGG   
  
  
+ TTTTTCACAG ACCATCTTTT CCAAAATAAG GAGTCACACT TAGGGTTTTT CTTTCTTATT TTGTTTACCC   
  
  
+ TTTAAAAATA AAACAAAAAT AAGTGGCGAC TCCAAGTCAT TTTTCTTAAT CAATAAAGAT CATTTTTTCG   
  
  
+ AAAACGAAAA TCGAGCTCGC CATCGAGTGG GAAACGCATT GAGCCGAAAT GCGGGGTCCA TAGGTATGTT   
  
  
+ TTTTATTTAT GAGATAAAAA GTAAATAATA TTTTTTTCGG GTTTAAAAAA TATATTATTG ATTACTTGTA   
  
  
+ ATTATATGAG CCTCGTTTGT ACTTATTATT AAATACATAT TCAAGGTAAA ATTATCCTTT AAAAAATTCT   
  
  
+ TAGGCCGAGG GAACCTTCCT ATTAATATAA GTCATCCAGC CCACCTTATG GGGGTAATTC TCCCCATATA   
  
  
+ CCATGGGTCC AAATCGGCGG CCCGGTCAGT TCAACGACAG TACGATGGCA CTCTGAACCC TGTGCTGCAT   
  
  
+ CTGCTCACAA AAAGTACACC ACAGTTGTCT TTCACTCTCT CTGCCCATTC ACTATGCAAA CACGTTTATA   
  
  
+ AAATTGGTTA TTTTATAAAC GTTTCTCTTT CAAATGGTTA TTTTATATTA GTAACAAAAA TATTATAATA   
  
  
+ ATAAAAAAAT CAATATTACG TAATTGCTAA AAATGAAATT ACATCATGTT GTAAGATATT TCATTTGCCT   
  
  
+ ATTAATAATT TTTTATTATT TATAAAATTG GGTTGAATTT CAATATTTTT TAAAATAAGT CTTATTTATT   
  
  
+ TAAGTGAAAT TTTTTTTATA GATAAAGTCA CAAAAATCAT TTTTCTAAAT AATTTATAAA AAGATACTTT   
  
  
+ TCCAATAAAA ATTAAATTAA AATGCTTTTA TAAATAAAAT TGTAACAATT CATTCCATAG CAAAGGTAAA   
  
  
+ TAAAGCCATT TTCTTATCAA TTAAATCAAG CTTCTCTTTT TTATGAAATT GTTTTTAATA AATTCTTTTA   
  
  
+ GTAACAATAA GATTTTGTTC ATATTTTAAT ACATCTCCTT GGCAATTGGG CAAAGATGGC TGGTAGTGAC   
  
  
+ ATGAATCCAA GCATGAATTA GTCCGGGCGT TGTTACTTGC TACAAGGGTT ATGAGCATGG AAGGAAAAAT   
  
  
+ AATTAAAAAA TAAAAAAAAT TAAATTACTT CAATCACAAA GTTGAAAATA ATAAAATTGT GTTTGGAAGT   
  
  
+ ATTTGTACTT AAAGTTTTTT AGTAAAAGTG TTTCCCTAAA AGGTGTTTTT TTTAGAGAAA GCAGCTATAA   
  
  
+ AATGTTTTTT CAAAAATATT TTTTTATTTA AAAACACTTT CTTAAATCAC TTGTTATAAG TCACCTTAGA   
  
  
+ ATGACTGACG GTGTTGCACA TATCAAAAAG CATTTATGCC CTAGTGCCAT TTTATTATTT TATATCTTAA   
  
  
+ TTTTAGATAT TTTTATTTTT ATTTAATGAT TTATATTTGA ACGTTAATTT TCAATATATC ATATTGTATC   
  
  
+ TTTTATAGCC ACGTTTATTC AAATATATTT TTTATATTTA TAAATATTTT TTCTAAAATA TGATTCGAGA   
  
  
+ ATGCTGACAT GTGAATCGCA TGGGGGTAGG GCAAGCCATC TTGGACCCAT TAAACTTCAA ATTCCATGAT   
  
  
+ TGACTAGGAC TTGCCCAAAA ATTTCATTAC CCCAGATGCC CACATGTGAA TGCAGTCTCT GACAGAGTAG   
  
  
+ GTGGGAATAA ACCAAAATGC ATTTATTTAT TTTTCAAGAT TTTAATTATT ATTCACCTAA AATCCTTCAT   
  
  
+ AGGGATTAAA ATATTAGACC ATTGGATTTG CTCTCCTCAA TAGGGTTGAA CTTTGATTAG TACGTTTATA   
  
  
+ TTATTTTTTT CCTTTTTCGT CAAAATGCAT TCTGTCACAA AATTAATGTA GTCAAGGAAT TGACAAGTGA   
  
  
+ GAAGAGAGTC ACTTTAAATG ATTCAGCTGT CCACATTAAA GATAAGTCAC ATTTGTGGTC CATACTCCCC   
  
  
+ AAGACTTTAA TTTGAATTTA GCTCAGACTT TTCGTATTTT ATTTGTGTTT TTCTATTTAA GCTATCAATA   
  
  
+ CATTTATAAA TAAGTGATTT TAATATAAAA GATCAAGTAA AAAACAAATA AAGTACATAA AATATTTTTA   
  
  
+ TTCAATTACA TATTGTATCA AATTTTCAAG AGCAAAATTT ATAGCATTTT TCAATGTTTT GGAAATCAAA   
  
  
+ AAAGTAATAT ATTATCAGTT CACTAGTTAA ATTGGTGATT GAACCATAGA TGATGTCATA ATATATATTT   
  
  
+ TATATTTATT AAAATTAAAA ATATTTATAA GAAATTTTAA ATATATGAGA AATTAAAAGT CAATAATATT   
  
  
+ TTTTTCTTCA TCTTTAATAT TATCAAATGA ATATATATAT ATATATAAAT GTATTGATAT TTTAAAATTT   
  
  
+ TGTTAAATAA AATATATATA ATATGAAGAT ATTTATTGAA AGTTATTTAA TTTACCTATA TATATATTAA   
  
  
+ AAGTTATTTA TGGTTGTAAA AAATATTACA TTATTTTATT TATATTATTA ATATTACATT ATTTTATTTA   
  
  
+ TATTATTTAT AATTTCATTA TTATAAAAAG TTTGAAAATA TTATTGCTAT ATAATTTGTT GTTGTGTGAA   
  
  
+ GATCGGCAAA AAAACAGGAG TAGCAGTGTG GTGTCGTCCT TGCCCCTTGA ACTGGAGGTT GGTTTCCCAT   
  
  
+ TCCTCCCTCC CACGAATCCC ACATGTAAAG CAAATAAAAG AGCTTTACAT ACACATGATT AATGTATTTT   
  
  
+ TATTGTCAAT AAATTTTATA ATAAAACTTA TAAATCTTAT AAATTTAATA GGAAAAAAAA AAGTCTTCTA   
  
  
+ GAATTGATTT TATAATACAC CATACCAAGA TCACATGAAT TGCATGTGTC GGTGGACAAG GTTGCCAAAA   
  
  
+ TTCCTTATTC CTATACAGAA TCTTTTCATT GTTATATTAT CATAGTCGAA ACCCAGTAGT ATATAAAGAG   
  
  
+ GTTACGAGTG GAGGATGGAT ATGAAATAAA TAAGAGGCCT TAGAGATCAG GGTGTGAGAC ATGGATTTGG   
  
  
+ CAAATGGTGA GAGATCTAGT GAGC  

- TTAGTAATTT CAGATTAAGA AGTTTTAAGG CTTAAGATTT AAATCGATAA ATTTTAAGGT TAACAGGATA   
  
  
- GAGGCTTGAA TTAAAAGTCA AAGGGTACGA GGTTAAGGGC ATAAATGGGG CGATGAATAA TAATAGTAAT   
  
  
- AGTAATAGTG GAATAAGTAA TAAAGATATT TAAGGCGGAA GCTTACTAAA GATTCTAGAG GTTAAAAATA   
  
  
- TGTAGTTGAA AGTATTAAAG ATGCAGAGTT TATTGTATGC TAAGACTAAG GAAAGTTTTA AATTGAGGGT   
  
  
- ATTAAGGTTA AAAGCATTGA ATTAAAAAGA ATTTTTAGGG CTTGAAAGTT TTAGGGTTAA AAGAAAGTTT   
  
  
- AAATTAAAGA TTTACAAGAG TAAGAATGTA GATTAAGGAT TCTTAGGTTA AAAGTTTATT AGAGATTCTA   
  
  
- AGGCTAAGAG TTTACTTAAA GTTTTTAGGT CAAAGGGAAG CTTGTTAAAA TTAGGGACAA ATCACTACGT   
  
  
- ACATTATACA AAACACGAGA TATACCACAT GATTGGGAGA GATAACTATC GCGTACCACC AAGCACCGGG   
  
  
- TCCATACATG GGTAAAAGAG AGATTAGCAA CAGATACGTA CAGAGCTAAG AATACACACG TGCTAGTAAG   
  
  
- TCCTATAAGT AACTAAATGA GTAGTTAACA GTGCCGTCGA AGTAAAATAA TCATCTCTGG GCTGAAATCC   
  
  
- CTGAATCTCC CCACGATGCC AGGAATGGCA TGGAAGGACT ATTCATTGGA CTGGGGGCTT GGGCTAGGCC   
  
  
- AAAAAGTGTC TGGTAGAAAA GGTTTTATTC CTCAGTGTGA ATCCCAAAAA GAAAGAATAA AACAAATGGG   
  
  
- AAATTTTTAT TTTGTTTTTA TTCACCGCTG AGGTTCAGTA AAAAGAATTA GTTATTTCTA GTAAAAAAGC   
  
  
- TTTTGCTTTT AGCTCGAGCG GTAGCTCACC CTTTGCGTAA CTCGGCTTTA CGCCCCAGGT ATCCATACAA   
  
  
- AAAATAAATA CTCTATTTTT CATTTATTAT AAAAAAAGCC CAAATTTTTT ATATAATAAC TAATGAACAT   
  
  
- TAATATACTC GGAGCAAACA TGAATAATAA TTTATGTATA AGTTCCATTT TAATAGGAAA TTTTTTAAGA   
  
  
- ATCCGGCTCC CTTGGAAGGA TAATTATATT CAGTAGGTCG GGTGGAATAC CCCCATTAAG AGGGGTATAT   
  
  
- GGTACCCAGG TTTAGCCGCC GGGCCAGTCA AGTTGCTGTC ATGCTACCGT GAGACTTGGG ACACGACGTA   
  
  
- GACGAGTGTT TTTCATGTGG TGTCAACAGA AAGTGAGAGA GACGGGTAAG TGATACGTTT GTGCAAATAT   
  
  
- TTTAACCAAT AAAATATTTG CAAAGAGAAA GTTTACCAAT AAAATATAAT CATTGTTTTT ATAATATTAT   
  
  
- TATTTTTTTA GTTATAATGC ATTAACGATT TTTACTTTAA TGTAGTACAA CATTCTATAA AGTAAACGGA   
  
  
- TAATTATTAA AAAATAATAA ATATTTTAAC CCAACTTAAA GTTATAAAAA ATTTTATTCA GAATAAATAA   
  
  
- ATTCACTTTA AAAAAAATAT CTATTTCAGT GTTTTTAGTA AAAAGATTTA TTAAATATTT TTCTATGAAA   
  
  
- AGGTTATTTT TAATTTAATT TTACGAAAAT ATTTATTTTA ACATTGTTAA GTAAGGTATC GTTTCCATTT   
  
  
- ATTTCGGTAA AAGAATAGTT AATTTAGTTC GAAGAGAAAA AATACTTTAA CAAAAATTAT TTAAGAAAAT   
  
  
- CATTGTTATT CTAAAACAAG TATAAAATTA TGTAGAGGAA CCGTTAACCC GTTTCTACCG ACCATCACTG   
  
  
- TACTTAGGTT CGTACTTAAT CAGGCCCGCA ACAATGAACG ATGTTCCCAA TACTCGTACC TTCCTTTTTA   
  
  
- TTAATTTTTT ATTTTTTTTA ATTTAATGAA GTTAGTGTTT CAACTTTTAT TATTTTAACA CAAACCTTCA   
  
  
- TAAACATGAA TTTCAAAAAA TCATTTTCAC AAAGGGATTT TCCACAAAAA AAATCTCTTT CGTCGATATT   
  
  
- TTACAAAAAA GTTTTTATAA AAAAATAAAT TTTTGTGAAA GAATTTAGTG AACAATATTC AGTGGAATCT   
  
  
- TACTGACTGC CACAACGTGT ATAGTTTTTC GTAAATACGG GATCACGGTA AAATAATAAA ATATAGAATT   
  
  
- AAAATCTATA AAAATAAAAA TAAATTACTA AATATAAACT TGCAATTAAA AGTTATATAG TATAACATAG   
  
  
- AAAATATCGG TGCAAATAAG TTTATATAAA AAATATAAAT ATTTATAAAA AAGATTTTAT ACTAAGCTCT   
  
  
- TACGACTGTA CACTTAGCGT ACCCCCATCC CGTTCGGTAG AACCTGGGTA ATTTGAAGTT TAAGGTACTA   
  
  
- ACTGATCCTG AACGGGTTTT TAAAGTAATG GGGTCTACGG GTGTACACTT ACGTCAGAGA CTGTCTCATC   
  
  
- CACCCTTATT TGGTTTTACG TAAATAAATA AAAAGTTCTA AAATTAATAA TAAGTGGATT TTAGGAAGTA   
  
  
- TCCCTAATTT TATAATCTGG TAACCTAAAC GAGAGGAGTT ATCCCAACTT GAAACTAATC ATGCAAATAT   
  
  
- AATAAAAAAA GGAAAAAGCA GTTTTACGTA AGACAGTGTT TTAATTACAT CAGTTCCTTA ACTGTTCACT   
  
  
- CTTCTCTCAG TGAAATTTAC TAAGTCGACA GGTGTAATTT CTATTCAGTG TAAACACCAG GTATGAGGGG   
  
  
- TTCTGAAATT AAACTTAAAT CGAGTCTGAA AAGCATAAAA TAAACACAAA AAGATAAATT CGATAGTTAT   
  
  
- GTAAATATTT ATTCACTAAA ATTATATTTT CTAGTTCATT TTTTGTTTAT TTCATGTATT TTATAAAAAT   
  
  
- AAGTTAATGT ATAACATAGT TTAAAAGTTC TCGTTTTAAA TATCGTAAAA AGTTACAAAA CCTTTAGTTT   
  
  
- TTTCATTATA TAATAGTCAA GTGATCAATT TAACCACTAA CTTGGTATCT ACTACAGTAT TATATATAAA   
  
  
- ATATAAATAA TTTTAATTTT TATAAATATT CTTTAAAATT TATATACTCT TTAATTTTCA GTTATTATAA   
  
  
- AAAAAGAAGT AGAAATTATA ATAGTTTACT TATATATATA TATATATTTA CATAACTATA AAATTTTAAA   
  
  
- ACAATTTATT TTATATATAT TATACTTCTA TAAATAACTT TCAATAAATT AAATGGATAT ATATATAATT   
  
  
- TTCAATAAAT ACCAACATTT TTTATAATGT AATAAAATAA ATATAATAAT TATAATGTAA TAAAATAAAT   
  
  
- ATAATAAATA TTAAAGTAAT AATATTTTTC AAACTTTTAT AATAACGATA TATTAAACAA CAACACACTT   
  
  
- CTAGCCGTTT TTTTGTCCTC ATCGTCACAC CACAGCAGGA ACGGGGAACT TGACCTCCAA CCAAAGGGTA   
  
  
- AGGAGGGAGG GTGCTTAGGG TGTACATTTC GTTTATTTTC TCGAAATGTA TGTGTACTAA TTACATAAAA   
  
  
- ATAACAGTTA TTTAAAATAT TATTTTGAAT ATTTAGAATA TTTAAATTAT CCTTTTTTTT TTCAGAAGAT   
  
  
- CTTAACTAAA ATATTATGTG GTATGGTTCT AGTGTACTTA ACGTACACAG CCACCTGTTC CAACGGTTTT   
  
  
- AAGGAATAAG GATATGTCTT AGAAAAGTAA CAATATAATA GTATCAGCTT TGGGTCATCA TATATTTCTC   
  
  
- CAATGCTCAC CTCCTACCTA TACTTTATTT ATTCTCCGGA ATCTCTAGTC CCACACTCTG TACCTAAACC   
  
  
- GTTTACCACT CTCTAGATCA CTCG

+     TCT-motif

| Site Name | Organism | Position | Strand | Matrix score. | sequence | function |
| --- | --- | --- | --- | --- | --- | --- |
| TCT-motif | Arabidopsis thaliana | 373 | + | 6 | TCTTAC | part of a light responsive element |
| TCT-motif | Arabidopsis thaliana | 1451 | - | 6 | TCTTAC | part of a light responsive element |

>PlantCARE\_32120   
+ AATCATTAAA GTCTAATTCT TCAAAATTCC GAATTCTAAA TTTAGCTATT TAAAATTCCA ATTGTCCTAT   
  
  
+ CTCCGAACTT AATTTTCAGT TTCCCATGCT CCAATTCCCG TATTTACCCC GCTACTTATT ATTATCATTA   
  
  
+ TCATTATCAC CTTATTCATT ATTTCTATAA ATTCCGCCTT CGAATGATTT CTAAGATCTC CAATTTTTAT   
  
  
+ ACATCAACTT TCATAATTTC TACGTCTCAA ATAACATACG ATTCTGATTC CTTTCAAAAT TTAACTCCCA   
  
  
+ TAATTCCAAT TTTCGTAACT TAATTTTTCT TAAAAATCCC GAACTTTCAA AATCCCAATT TTCTTTCAAA   
  
  
+ TTTAATTTCT AAATGTTCTC ATTCTTACAT CTAATTCCTA AGAATCCAAT TTTCAAATAA TCTCTAAGAT   
  
  
+ TCCGATTCTC AAATGAATTT CAAAAATCCA GTTTCCCTTC GAACAATTTT AATCCCTGTT TAGTGATGCA   
  
  
+ TGTAATATGT TTTGTGCTCT ATATGGTGTA CTAACCCTCT CTATTGATAG CGCATGGTGG TTCGTGGCCC   
  
  
+ AGGTATGTAC CCATTTTCTC TCTAATCGTT GTCTATGCAT GTCTCGATTC TTATGTGTGC ACGATCATTC   
  
  
+ AGGATATTCA TTGATTTACT CATCAATTGT CACGGCAGCT TCATTTTATT AGTAGAGACC CGACTTTAGG   
  
  
+ GACTTAGAGG GGTGCTACGG TCCTTACCGT ACCTTCCTGA TAAGTAACCT GACCCCCGAA CCCGATCCGG   
  
  
+ TTTTTCACAG ACCATCTTTT CCAAAATAAG GAGTCACACT TAGGGTTTTT CTTTCTTATT TTGTTTACCC   
  
  
+ TTTAAAAATA AAACAAAAAT AAGTGGCGAC TCCAAGTCAT TTTTCTTAAT CAATAAAGAT CATTTTTTCG   
  
  
+ AAAACGAAAA TCGAGCTCGC CATCGAGTGG GAAACGCATT GAGCCGAAAT GCGGGGTCCA TAGGTATGTT   
  
  
+ TTTTATTTAT GAGATAAAAA GTAAATAATA TTTTTTTCGG GTTTAAAAAA TATATTATTG ATTACTTGTA   
  
  
+ ATTATATGAG CCTCGTTTGT ACTTATTATT AAATACATAT TCAAGGTAAA ATTATCCTTT AAAAAATTCT   
  
  
+ TAGGCCGAGG GAACCTTCCT ATTAATATAA GTCATCCAGC CCACCTTATG GGGGTAATTC TCCCCATATA   
  
  
+ CCATGGGTCC AAATCGGCGG CCCGGTCAGT TCAACGACAG TACGATGGCA CTCTGAACCC TGTGCTGCAT   
  
  
+ CTGCTCACAA AAAGTACACC ACAGTTGTCT TTCACTCTCT CTGCCCATTC ACTATGCAAA CACGTTTATA   
  
  
+ AAATTGGTTA TTTTATAAAC GTTTCTCTTT CAAATGGTTA TTTTATATTA GTAACAAAAA TATTATAATA   
  
  
+ ATAAAAAAAT CAATATTACG TAATTGCTAA AAATGAAATT ACATCATGTT GTAAGATATT TCATTTGCCT   
  
  
+ ATTAATAATT TTTTATTATT TATAAAATTG GGTTGAATTT CAATATTTTT TAAAATAAGT CTTATTTATT   
  
  
+ TAAGTGAAAT TTTTTTTATA GATAAAGTCA CAAAAATCAT TTTTCTAAAT AATTTATAAA AAGATACTTT   
  
  
+ TCCAATAAAA ATTAAATTAA AATGCTTTTA TAAATAAAAT TGTAACAATT CATTCCATAG CAAAGGTAAA   
  
  
+ TAAAGCCATT TTCTTATCAA TTAAATCAAG CTTCTCTTTT TTATGAAATT GTTTTTAATA AATTCTTTTA   
  
  
+ GTAACAATAA GATTTTGTTC ATATTTTAAT ACATCTCCTT GGCAATTGGG CAAAGATGGC TGGTAGTGAC   
  
  
+ ATGAATCCAA GCATGAATTA GTCCGGGCGT TGTTACTTGC TACAAGGGTT ATGAGCATGG AAGGAAAAAT   
  
  
+ AATTAAAAAA TAAAAAAAAT TAAATTACTT CAATCACAAA GTTGAAAATA ATAAAATTGT GTTTGGAAGT   
  
  
+ ATTTGTACTT AAAGTTTTTT AGTAAAAGTG TTTCCCTAAA AGGTGTTTTT TTTAGAGAAA GCAGCTATAA   
  
  
+ AATGTTTTTT CAAAAATATT TTTTTATTTA AAAACACTTT CTTAAATCAC TTGTTATAAG TCACCTTAGA   
  
  
+ ATGACTGACG GTGTTGCACA TATCAAAAAG CATTTATGCC CTAGTGCCAT TTTATTATTT TATATCTTAA   
  
  
+ TTTTAGATAT TTTTATTTTT ATTTAATGAT TTATATTTGA ACGTTAATTT TCAATATATC ATATTGTATC   
  
  
+ TTTTATAGCC ACGTTTATTC AAATATATTT TTTATATTTA TAAATATTTT TTCTAAAATA TGATTCGAGA   
  
  
+ ATGCTGACAT GTGAATCGCA TGGGGGTAGG GCAAGCCATC TTGGACCCAT TAAACTTCAA ATTCCATGAT   
  
  
+ TGACTAGGAC TTGCCCAAAA ATTTCATTAC CCCAGATGCC CACATGTGAA TGCAGTCTCT GACAGAGTAG   
  
  
+ GTGGGAATAA ACCAAAATGC ATTTATTTAT TTTTCAAGAT TTTAATTATT ATTCACCTAA AATCCTTCAT   
  
  
+ AGGGATTAAA ATATTAGACC ATTGGATTTG CTCTCCTCAA TAGGGTTGAA CTTTGATTAG TACGTTTATA   
  
  
+ TTATTTTTTT CCTTTTTCGT CAAAATGCAT TCTGTCACAA AATTAATGTA GTCAAGGAAT TGACAAGTGA   
  
  
+ GAAGAGAGTC ACTTTAAATG ATTCAGCTGT CCACATTAAA GATAAGTCAC ATTTGTGGTC CATACTCCCC   
  
  
+ AAGACTTTAA TTTGAATTTA GCTCAGACTT TTCGTATTTT ATTTGTGTTT TTCTATTTAA GCTATCAATA   
  
  
+ CATTTATAAA TAAGTGATTT TAATATAAAA GATCAAGTAA AAAACAAATA AAGTACATAA AATATTTTTA   
  
  
+ TTCAATTACA TATTGTATCA AATTTTCAAG AGCAAAATTT ATAGCATTTT TCAATGTTTT GGAAATCAAA   
  
  
+ AAAGTAATAT ATTATCAGTT CACTAGTTAA ATTGGTGATT GAACCATAGA TGATGTCATA ATATATATTT   
  
  
+ TATATTTATT AAAATTAAAA ATATTTATAA GAAATTTTAA ATATATGAGA AATTAAAAGT CAATAATATT   
  
  
+ TTTTTCTTCA TCTTTAATAT TATCAAATGA ATATATATAT ATATATAAAT GTATTGATAT TTTAAAATTT   
  
  
+ TGTTAAATAA AATATATATA ATATGAAGAT ATTTATTGAA AGTTATTTAA TTTACCTATA TATATATTAA   
  
  
+ AAGTTATTTA TGGTTGTAAA AAATATTACA TTATTTTATT TATATTATTA ATATTACATT ATTTTATTTA   
  
  
+ TATTATTTAT AATTTCATTA TTATAAAAAG TTTGAAAATA TTATTGCTAT ATAATTTGTT GTTGTGTGAA   
  
  
+ GATCGGCAAA AAAACAGGAG TAGCAGTGTG GTGTCGTCCT TGCCCCTTGA ACTGGAGGTT GGTTTCCCAT   
  
  
+ TCCTCCCTCC CACGAATCCC ACATGTAAAG CAAATAAAAG AGCTTTACAT ACACATGATT AATGTATTTT   
  
  
+ TATTGTCAAT AAATTTTATA ATAAAACTTA TAAATCTTAT AAATTTAATA GGAAAAAAAA AAGTCTTCTA   
  
  
+ GAATTGATTT TATAATACAC CATACCAAGA TCACATGAAT TGCATGTGTC GGTGGACAAG GTTGCCAAAA   
  
  
+ TTCCTTATTC CTATACAGAA TCTTTTCATT GTTATATTAT CATAGTCGAA ACCCAGTAGT ATATAAAGAG   
  
  
+ GTTACGAGTG GAGGATGGAT ATGAAATAAA TAAGAGGCCT TAGAGATCAG GGTGTGAGAC ATGGATTTGG   
  
  
+ CAAATGGTGA GAGATCTAGT GAGC  

- TTAGTAATTT CAGATTAAGA AGTTTTAAGG CTTAAGATTT AAATCGATAA ATTTTAAGGT TAACAGGATA   
  
  
- GAGGCTTGAA TTAAAAGTCA AAGGGTACGA GGTTAAGGGC ATAAATGGGG CGATGAATAA TAATAGTAAT   
  
  
- AGTAATAGTG GAATAAGTAA TAAAGATATT TAAGGCGGAA GCTTACTAAA GATTCTAGAG GTTAAAAATA   
  
  
- TGTAGTTGAA AGTATTAAAG ATGCAGAGTT TATTGTATGC TAAGACTAAG GAAAGTTTTA AATTGAGGGT   
  
  
- ATTAAGGTTA AAAGCATTGA ATTAAAAAGA ATTTTTAGGG CTTGAAAGTT TTAGGGTTAA AAGAAAGTTT   
  
  
- AAATTAAAGA TTTACAAGAG TAAGAATGTA GATTAAGGAT TCTTAGGTTA AAAGTTTATT AGAGATTCTA   
  
  
- AGGCTAAGAG TTTACTTAAA GTTTTTAGGT CAAAGGGAAG CTTGTTAAAA TTAGGGACAA ATCACTACGT   
  
  
- ACATTATACA AAACACGAGA TATACCACAT GATTGGGAGA GATAACTATC GCGTACCACC AAGCACCGGG   
  
  
- TCCATACATG GGTAAAAGAG AGATTAGCAA CAGATACGTA CAGAGCTAAG AATACACACG TGCTAGTAAG   
  
  
- TCCTATAAGT AACTAAATGA GTAGTTAACA GTGCCGTCGA AGTAAAATAA TCATCTCTGG GCTGAAATCC   
  
  
- CTGAATCTCC CCACGATGCC AGGAATGGCA TGGAAGGACT ATTCATTGGA CTGGGGGCTT GGGCTAGGCC   
  
  
- AAAAAGTGTC TGGTAGAAAA GGTTTTATTC CTCAGTGTGA ATCCCAAAAA GAAAGAATAA AACAAATGGG   
  
  
- AAATTTTTAT TTTGTTTTTA TTCACCGCTG AGGTTCAGTA AAAAGAATTA GTTATTTCTA GTAAAAAAGC   
  
  
- TTTTGCTTTT AGCTCGAGCG GTAGCTCACC CTTTGCGTAA CTCGGCTTTA CGCCCCAGGT ATCCATACAA   
  
  
- AAAATAAATA CTCTATTTTT CATTTATTAT AAAAAAAGCC CAAATTTTTT ATATAATAAC TAATGAACAT   
  
  
- TAATATACTC GGAGCAAACA TGAATAATAA TTTATGTATA AGTTCCATTT TAATAGGAAA TTTTTTAAGA   
  
  
- ATCCGGCTCC CTTGGAAGGA TAATTATATT CAGTAGGTCG GGTGGAATAC CCCCATTAAG AGGGGTATAT   
  
  
- GGTACCCAGG TTTAGCCGCC GGGCCAGTCA AGTTGCTGTC ATGCTACCGT GAGACTTGGG ACACGACGTA   
  
  
- GACGAGTGTT TTTCATGTGG TGTCAACAGA AAGTGAGAGA GACGGGTAAG TGATACGTTT GTGCAAATAT   
  
  
- TTTAACCAAT AAAATATTTG CAAAGAGAAA GTTTACCAAT AAAATATAAT CATTGTTTTT ATAATATTAT   
  
  
- TATTTTTTTA GTTATAATGC ATTAACGATT TTTACTTTAA TGTAGTACAA CATTCTATAA AGTAAACGGA   
  
  
- TAATTATTAA AAAATAATAA ATATTTTAAC CCAACTTAAA GTTATAAAAA ATTTTATTCA GAATAAATAA   
  
  
- ATTCACTTTA AAAAAAATAT CTATTTCAGT GTTTTTAGTA AAAAGATTTA TTAAATATTT TTCTATGAAA   
  
  
- AGGTTATTTT TAATTTAATT TTACGAAAAT ATTTATTTTA ACATTGTTAA GTAAGGTATC GTTTCCATTT   
  
  
- ATTTCGGTAA AAGAATAGTT AATTTAGTTC GAAGAGAAAA AATACTTTAA CAAAAATTAT TTAAGAAAAT   
  
  
- CATTGTTATT CTAAAACAAG TATAAAATTA TGTAGAGGAA CCGTTAACCC GTTTCTACCG ACCATCACTG   
  
  
- TACTTAGGTT CGTACTTAAT CAGGCCCGCA ACAATGAACG ATGTTCCCAA TACTCGTACC TTCCTTTTTA   
  
  
- TTAATTTTTT ATTTTTTTTA ATTTAATGAA GTTAGTGTTT CAACTTTTAT TATTTTAACA CAAACCTTCA   
  
  
- TAAACATGAA TTTCAAAAAA TCATTTTCAC AAAGGGATTT TCCACAAAAA AAATCTCTTT CGTCGATATT   
  
  
- TTACAAAAAA GTTTTTATAA AAAAATAAAT TTTTGTGAAA GAATTTAGTG AACAATATTC AGTGGAATCT   
  
  
- TACTGACTGC CACAACGTGT ATAGTTTTTC GTAAATACGG GATCACGGTA AAATAATAAA ATATAGAATT   
  
  
- AAAATCTATA AAAATAAAAA TAAATTACTA AATATAAACT TGCAATTAAA AGTTATATAG TATAACATAG   
  
  
- AAAATATCGG TGCAAATAAG TTTATATAAA AAATATAAAT ATTTATAAAA AAGATTTTAT ACTAAGCTCT   
  
  
- TACGACTGTA CACTTAGCGT ACCCCCATCC CGTTCGGTAG AACCTGGGTA ATTTGAAGTT TAAGGTACTA   
  
  
- ACTGATCCTG AACGGGTTTT TAAAGTAATG GGGTCTACGG GTGTACACTT ACGTCAGAGA CTGTCTCATC   
  
  
- CACCCTTATT TGGTTTTACG TAAATAAATA AAAAGTTCTA AAATTAATAA TAAGTGGATT TTAGGAAGTA   
  
  
- TCCCTAATTT TATAATCTGG TAACCTAAAC GAGAGGAGTT ATCCCAACTT GAAACTAATC ATGCAAATAT   
  
  
- AATAAAAAAA GGAAAAAGCA GTTTTACGTA AGACAGTGTT TTAATTACAT CAGTTCCTTA ACTGTTCACT   
  
  
- CTTCTCTCAG TGAAATTTAC TAAGTCGACA GGTGTAATTT CTATTCAGTG TAAACACCAG GTATGAGGGG   
  
  
- TTCTGAAATT AAACTTAAAT CGAGTCTGAA AAGCATAAAA TAAACACAAA AAGATAAATT CGATAGTTAT   
  
  
- GTAAATATTT ATTCACTAAA ATTATATTTT CTAGTTCATT TTTTGTTTAT TTCATGTATT TTATAAAAAT   
  
  
- AAGTTAATGT ATAACATAGT TTAAAAGTTC TCGTTTTAAA TATCGTAAAA AGTTACAAAA CCTTTAGTTT   
  
  
- TTTCATTATA TAATAGTCAA GTGATCAATT TAACCACTAA CTTGGTATCT ACTACAGTAT TATATATAAA   
  
  
- ATATAAATAA TTTTAATTTT TATAAATATT CTTTAAAATT TATATACTCT TTAATTTTCA GTTATTATAA   
  
  
- AAAAAGAAGT AGAAATTATA ATAGTTTACT TATATATATA TATATATTTA CATAACTATA AAATTTTAAA   
  
  
- ACAATTTATT TTATATATAT TATACTTCTA TAAATAACTT TCAATAAATT AAATGGATAT ATATATAATT   
  
  
- TTCAATAAAT ACCAACATTT TTTATAATGT AATAAAATAA ATATAATAAT TATAATGTAA TAAAATAAAT   
  
  
- ATAATAAATA TTAAAGTAAT AATATTTTTC AAACTTTTAT AATAACGATA TATTAAACAA CAACACACTT   
  
  
- CTAGCCGTTT TTTTGTCCTC ATCGTCACAC CACAGCAGGA ACGGGGAACT TGACCTCCAA CCAAAGGGTA   
  
  
- AGGAGGGAGG GTGCTTAGGG TGTACATTTC GTTTATTTTC TCGAAATGTA TGTGTACTAA TTACATAAAA   
  
  
- ATAACAGTTA TTTAAAATAT TATTTTGAAT ATTTAGAATA TTTAAATTAT CCTTTTTTTT TTCAGAAGAT   
  
  
- CTTAACTAAA ATATTATGTG GTATGGTTCT AGTGTACTTA ACGTACACAG CCACCTGTTC CAACGGTTTT   
  
  
- AAGGAATAAG GATATGTCTT AGAAAAGTAA CAATATAATA GTATCAGCTT TGGGTCATCA TATATTTCTC   
  
  
- CAATGCTCAC CTCCTACCTA TACTTTATTT ATTCTCCGGA ATCTCTAGTC CCACACTCTG TACCTAAACC   
  
  
- GTTTACCACT CTCTAGATCA CTCG

+     TGA-element

| Site Name | Organism | Position | Strand | Matrix score. | sequence | function |
| --- | --- | --- | --- | --- | --- | --- |
| TGA-element | Brassica oleracea | 1223 | + | 6 | AACGAC | auxin-responsive element |

>PlantCARE\_32120   
+ AATCATTAAA GTCTAATTCT TCAAAATTCC GAATTCTAAA TTTAGCTATT TAAAATTCCA ATTGTCCTAT   
  
  
+ CTCCGAACTT AATTTTCAGT TTCCCATGCT CCAATTCCCG TATTTACCCC GCTACTTATT ATTATCATTA   
  
  
+ TCATTATCAC CTTATTCATT ATTTCTATAA ATTCCGCCTT CGAATGATTT CTAAGATCTC CAATTTTTAT   
  
  
+ ACATCAACTT TCATAATTTC TACGTCTCAA ATAACATACG ATTCTGATTC CTTTCAAAAT TTAACTCCCA   
  
  
+ TAATTCCAAT TTTCGTAACT TAATTTTTCT TAAAAATCCC GAACTTTCAA AATCCCAATT TTCTTTCAAA   
  
  
+ TTTAATTTCT AAATGTTCTC ATTCTTACAT CTAATTCCTA AGAATCCAAT TTTCAAATAA TCTCTAAGAT   
  
  
+ TCCGATTCTC AAATGAATTT CAAAAATCCA GTTTCCCTTC GAACAATTTT AATCCCTGTT TAGTGATGCA   
  
  
+ TGTAATATGT TTTGTGCTCT ATATGGTGTA CTAACCCTCT CTATTGATAG CGCATGGTGG TTCGTGGCCC   
  
  
+ AGGTATGTAC CCATTTTCTC TCTAATCGTT GTCTATGCAT GTCTCGATTC TTATGTGTGC ACGATCATTC   
  
  
+ AGGATATTCA TTGATTTACT CATCAATTGT CACGGCAGCT TCATTTTATT AGTAGAGACC CGACTTTAGG   
  
  
+ GACTTAGAGG GGTGCTACGG TCCTTACCGT ACCTTCCTGA TAAGTAACCT GACCCCCGAA CCCGATCCGG   
  
  
+ TTTTTCACAG ACCATCTTTT CCAAAATAAG GAGTCACACT TAGGGTTTTT CTTTCTTATT TTGTTTACCC   
  
  
+ TTTAAAAATA AAACAAAAAT AAGTGGCGAC TCCAAGTCAT TTTTCTTAAT CAATAAAGAT CATTTTTTCG   
  
  
+ AAAACGAAAA TCGAGCTCGC CATCGAGTGG GAAACGCATT GAGCCGAAAT GCGGGGTCCA TAGGTATGTT   
  
  
+ TTTTATTTAT GAGATAAAAA GTAAATAATA TTTTTTTCGG GTTTAAAAAA TATATTATTG ATTACTTGTA   
  
  
+ ATTATATGAG CCTCGTTTGT ACTTATTATT AAATACATAT TCAAGGTAAA ATTATCCTTT AAAAAATTCT   
  
  
+ TAGGCCGAGG GAACCTTCCT ATTAATATAA GTCATCCAGC CCACCTTATG GGGGTAATTC TCCCCATATA   
  
  
+ CCATGGGTCC AAATCGGCGG CCCGGTCAGT TCAACGACAG TACGATGGCA CTCTGAACCC TGTGCTGCAT   
  
  
+ CTGCTCACAA AAAGTACACC ACAGTTGTCT TTCACTCTCT CTGCCCATTC ACTATGCAAA CACGTTTATA   
  
  
+ AAATTGGTTA TTTTATAAAC GTTTCTCTTT CAAATGGTTA TTTTATATTA GTAACAAAAA TATTATAATA   
  
  
+ ATAAAAAAAT CAATATTACG TAATTGCTAA AAATGAAATT ACATCATGTT GTAAGATATT TCATTTGCCT   
  
  
+ ATTAATAATT TTTTATTATT TATAAAATTG GGTTGAATTT CAATATTTTT TAAAATAAGT CTTATTTATT   
  
  
+ TAAGTGAAAT TTTTTTTATA GATAAAGTCA CAAAAATCAT TTTTCTAAAT AATTTATAAA AAGATACTTT   
  
  
+ TCCAATAAAA ATTAAATTAA AATGCTTTTA TAAATAAAAT TGTAACAATT CATTCCATAG CAAAGGTAAA   
  
  
+ TAAAGCCATT TTCTTATCAA TTAAATCAAG CTTCTCTTTT TTATGAAATT GTTTTTAATA AATTCTTTTA   
  
  
+ GTAACAATAA GATTTTGTTC ATATTTTAAT ACATCTCCTT GGCAATTGGG CAAAGATGGC TGGTAGTGAC   
  
  
+ ATGAATCCAA GCATGAATTA GTCCGGGCGT TGTTACTTGC TACAAGGGTT ATGAGCATGG AAGGAAAAAT   
  
  
+ AATTAAAAAA TAAAAAAAAT TAAATTACTT CAATCACAAA GTTGAAAATA ATAAAATTGT GTTTGGAAGT   
  
  
+ ATTTGTACTT AAAGTTTTTT AGTAAAAGTG TTTCCCTAAA AGGTGTTTTT TTTAGAGAAA GCAGCTATAA   
  
  
+ AATGTTTTTT CAAAAATATT TTTTTATTTA AAAACACTTT CTTAAATCAC TTGTTATAAG TCACCTTAGA   
  
  
+ ATGACTGACG GTGTTGCACA TATCAAAAAG CATTTATGCC CTAGTGCCAT TTTATTATTT TATATCTTAA   
  
  
+ TTTTAGATAT TTTTATTTTT ATTTAATGAT TTATATTTGA ACGTTAATTT TCAATATATC ATATTGTATC   
  
  
+ TTTTATAGCC ACGTTTATTC AAATATATTT TTTATATTTA TAAATATTTT TTCTAAAATA TGATTCGAGA   
  
  
+ ATGCTGACAT GTGAATCGCA TGGGGGTAGG GCAAGCCATC TTGGACCCAT TAAACTTCAA ATTCCATGAT   
  
  
+ TGACTAGGAC TTGCCCAAAA ATTTCATTAC CCCAGATGCC CACATGTGAA TGCAGTCTCT GACAGAGTAG   
  
  
+ GTGGGAATAA ACCAAAATGC ATTTATTTAT TTTTCAAGAT TTTAATTATT ATTCACCTAA AATCCTTCAT   
  
  
+ AGGGATTAAA ATATTAGACC ATTGGATTTG CTCTCCTCAA TAGGGTTGAA CTTTGATTAG TACGTTTATA   
  
  
+ TTATTTTTTT CCTTTTTCGT CAAAATGCAT TCTGTCACAA AATTAATGTA GTCAAGGAAT TGACAAGTGA   
  
  
+ GAAGAGAGTC ACTTTAAATG ATTCAGCTGT CCACATTAAA GATAAGTCAC ATTTGTGGTC CATACTCCCC   
  
  
+ AAGACTTTAA TTTGAATTTA GCTCAGACTT TTCGTATTTT ATTTGTGTTT TTCTATTTAA GCTATCAATA   
  
  
+ CATTTATAAA TAAGTGATTT TAATATAAAA GATCAAGTAA AAAACAAATA AAGTACATAA AATATTTTTA   
  
  
+ TTCAATTACA TATTGTATCA AATTTTCAAG AGCAAAATTT ATAGCATTTT TCAATGTTTT GGAAATCAAA   
  
  
+ AAAGTAATAT ATTATCAGTT CACTAGTTAA ATTGGTGATT GAACCATAGA TGATGTCATA ATATATATTT   
  
  
+ TATATTTATT AAAATTAAAA ATATTTATAA GAAATTTTAA ATATATGAGA AATTAAAAGT CAATAATATT   
  
  
+ TTTTTCTTCA TCTTTAATAT TATCAAATGA ATATATATAT ATATATAAAT GTATTGATAT TTTAAAATTT   
  
  
+ TGTTAAATAA AATATATATA ATATGAAGAT ATTTATTGAA AGTTATTTAA TTTACCTATA TATATATTAA   
  
  
+ AAGTTATTTA TGGTTGTAAA AAATATTACA TTATTTTATT TATATTATTA ATATTACATT ATTTTATTTA   
  
  
+ TATTATTTAT AATTTCATTA TTATAAAAAG TTTGAAAATA TTATTGCTAT ATAATTTGTT GTTGTGTGAA   
  
  
+ GATCGGCAAA AAAACAGGAG TAGCAGTGTG GTGTCGTCCT TGCCCCTTGA ACTGGAGGTT GGTTTCCCAT   
  
  
+ TCCTCCCTCC CACGAATCCC ACATGTAAAG CAAATAAAAG AGCTTTACAT ACACATGATT AATGTATTTT   
  
  
+ TATTGTCAAT AAATTTTATA ATAAAACTTA TAAATCTTAT AAATTTAATA GGAAAAAAAA AAGTCTTCTA   
  
  
+ GAATTGATTT TATAATACAC CATACCAAGA TCACATGAAT TGCATGTGTC GGTGGACAAG GTTGCCAAAA   
  
  
+ TTCCTTATTC CTATACAGAA TCTTTTCATT GTTATATTAT CATAGTCGAA ACCCAGTAGT ATATAAAGAG   
  
  
+ GTTACGAGTG GAGGATGGAT ATGAAATAAA TAAGAGGCCT TAGAGATCAG GGTGTGAGAC ATGGATTTGG   
  
  
+ CAAATGGTGA GAGATCTAGT GAGC  

- TTAGTAATTT CAGATTAAGA AGTTTTAAGG CTTAAGATTT AAATCGATAA ATTTTAAGGT TAACAGGATA   
  
  
- GAGGCTTGAA TTAAAAGTCA AAGGGTACGA GGTTAAGGGC ATAAATGGGG CGATGAATAA TAATAGTAAT   
  
  
- AGTAATAGTG GAATAAGTAA TAAAGATATT TAAGGCGGAA GCTTACTAAA GATTCTAGAG GTTAAAAATA   
  
  
- TGTAGTTGAA AGTATTAAAG ATGCAGAGTT TATTGTATGC TAAGACTAAG GAAAGTTTTA AATTGAGGGT   
  
  
- ATTAAGGTTA AAAGCATTGA ATTAAAAAGA ATTTTTAGGG CTTGAAAGTT TTAGGGTTAA AAGAAAGTTT   
  
  
- AAATTAAAGA TTTACAAGAG TAAGAATGTA GATTAAGGAT TCTTAGGTTA AAAGTTTATT AGAGATTCTA   
  
  
- AGGCTAAGAG TTTACTTAAA GTTTTTAGGT CAAAGGGAAG CTTGTTAAAA TTAGGGACAA ATCACTACGT   
  
  
- ACATTATACA AAACACGAGA TATACCACAT GATTGGGAGA GATAACTATC GCGTACCACC AAGCACCGGG   
  
  
- TCCATACATG GGTAAAAGAG AGATTAGCAA CAGATACGTA CAGAGCTAAG AATACACACG TGCTAGTAAG   
  
  
- TCCTATAAGT AACTAAATGA GTAGTTAACA GTGCCGTCGA AGTAAAATAA TCATCTCTGG GCTGAAATCC   
  
  
- CTGAATCTCC CCACGATGCC AGGAATGGCA TGGAAGGACT ATTCATTGGA CTGGGGGCTT GGGCTAGGCC   
  
  
- AAAAAGTGTC TGGTAGAAAA GGTTTTATTC CTCAGTGTGA ATCCCAAAAA GAAAGAATAA AACAAATGGG   
  
  
- AAATTTTTAT TTTGTTTTTA TTCACCGCTG AGGTTCAGTA AAAAGAATTA GTTATTTCTA GTAAAAAAGC   
  
  
- TTTTGCTTTT AGCTCGAGCG GTAGCTCACC CTTTGCGTAA CTCGGCTTTA CGCCCCAGGT ATCCATACAA   
  
  
- AAAATAAATA CTCTATTTTT CATTTATTAT AAAAAAAGCC CAAATTTTTT ATATAATAAC TAATGAACAT   
  
  
- TAATATACTC GGAGCAAACA TGAATAATAA TTTATGTATA AGTTCCATTT TAATAGGAAA TTTTTTAAGA   
  
  
- ATCCGGCTCC CTTGGAAGGA TAATTATATT CAGTAGGTCG GGTGGAATAC CCCCATTAAG AGGGGTATAT   
  
  
- GGTACCCAGG TTTAGCCGCC GGGCCAGTCA AGTTGCTGTC ATGCTACCGT GAGACTTGGG ACACGACGTA   
  
  
- GACGAGTGTT TTTCATGTGG TGTCAACAGA AAGTGAGAGA GACGGGTAAG TGATACGTTT GTGCAAATAT   
  
  
- TTTAACCAAT AAAATATTTG CAAAGAGAAA GTTTACCAAT AAAATATAAT CATTGTTTTT ATAATATTAT   
  
  
- TATTTTTTTA GTTATAATGC ATTAACGATT TTTACTTTAA TGTAGTACAA CATTCTATAA AGTAAACGGA   
  
  
- TAATTATTAA AAAATAATAA ATATTTTAAC CCAACTTAAA GTTATAAAAA ATTTTATTCA GAATAAATAA   
  
  
- ATTCACTTTA AAAAAAATAT CTATTTCAGT GTTTTTAGTA AAAAGATTTA TTAAATATTT TTCTATGAAA   
  
  
- AGGTTATTTT TAATTTAATT TTACGAAAAT ATTTATTTTA ACATTGTTAA GTAAGGTATC GTTTCCATTT   
  
  
- ATTTCGGTAA AAGAATAGTT AATTTAGTTC GAAGAGAAAA AATACTTTAA CAAAAATTAT TTAAGAAAAT   
  
  
- CATTGTTATT CTAAAACAAG TATAAAATTA TGTAGAGGAA CCGTTAACCC GTTTCTACCG ACCATCACTG   
  
  
- TACTTAGGTT CGTACTTAAT CAGGCCCGCA ACAATGAACG ATGTTCCCAA TACTCGTACC TTCCTTTTTA   
  
  
- TTAATTTTTT ATTTTTTTTA ATTTAATGAA GTTAGTGTTT CAACTTTTAT TATTTTAACA CAAACCTTCA   
  
  
- TAAACATGAA TTTCAAAAAA TCATTTTCAC AAAGGGATTT TCCACAAAAA AAATCTCTTT CGTCGATATT   
  
  
- TTACAAAAAA GTTTTTATAA AAAAATAAAT TTTTGTGAAA GAATTTAGTG AACAATATTC AGTGGAATCT   
  
  
- TACTGACTGC CACAACGTGT ATAGTTTTTC GTAAATACGG GATCACGGTA AAATAATAAA ATATAGAATT   
  
  
- AAAATCTATA AAAATAAAAA TAAATTACTA AATATAAACT TGCAATTAAA AGTTATATAG TATAACATAG   
  
  
- AAAATATCGG TGCAAATAAG TTTATATAAA AAATATAAAT ATTTATAAAA AAGATTTTAT ACTAAGCTCT   
  
  
- TACGACTGTA CACTTAGCGT ACCCCCATCC CGTTCGGTAG AACCTGGGTA ATTTGAAGTT TAAGGTACTA   
  
  
- ACTGATCCTG AACGGGTTTT TAAAGTAATG GGGTCTACGG GTGTACACTT ACGTCAGAGA CTGTCTCATC   
  
  
- CACCCTTATT TGGTTTTACG TAAATAAATA AAAAGTTCTA AAATTAATAA TAAGTGGATT TTAGGAAGTA   
  
  
- TCCCTAATTT TATAATCTGG TAACCTAAAC GAGAGGAGTT ATCCCAACTT GAAACTAATC ATGCAAATAT   
  
  
- AATAAAAAAA GGAAAAAGCA GTTTTACGTA AGACAGTGTT TTAATTACAT CAGTTCCTTA ACTGTTCACT   
  
  
- CTTCTCTCAG TGAAATTTAC TAAGTCGACA GGTGTAATTT CTATTCAGTG TAAACACCAG GTATGAGGGG   
  
  
- TTCTGAAATT AAACTTAAAT CGAGTCTGAA AAGCATAAAA TAAACACAAA AAGATAAATT CGATAGTTAT   
  
  
- GTAAATATTT ATTCACTAAA ATTATATTTT CTAGTTCATT TTTTGTTTAT TTCATGTATT TTATAAAAAT   
  
  
- AAGTTAATGT ATAACATAGT TTAAAAGTTC TCGTTTTAAA TATCGTAAAA AGTTACAAAA CCTTTAGTTT   
  
  
- TTTCATTATA TAATAGTCAA GTGATCAATT TAACCACTAA CTTGGTATCT ACTACAGTAT TATATATAAA   
  
  
- ATATAAATAA TTTTAATTTT TATAAATATT CTTTAAAATT TATATACTCT TTAATTTTCA GTTATTATAA   
  
  
- AAAAAGAAGT AGAAATTATA ATAGTTTACT TATATATATA TATATATTTA CATAACTATA AAATTTTAAA   
  
  
- ACAATTTATT TTATATATAT TATACTTCTA TAAATAACTT TCAATAAATT AAATGGATAT ATATATAATT   
  
  
- TTCAATAAAT ACCAACATTT TTTATAATGT AATAAAATAA ATATAATAAT TATAATGTAA TAAAATAAAT   
  
  
- ATAATAAATA TTAAAGTAAT AATATTTTTC AAACTTTTAT AATAACGATA TATTAAACAA CAACACACTT   
  
  
- CTAGCCGTTT TTTTGTCCTC ATCGTCACAC CACAGCAGGA ACGGGGAACT TGACCTCCAA CCAAAGGGTA   
  
  
- AGGAGGGAGG GTGCTTAGGG TGTACATTTC GTTTATTTTC TCGAAATGTA TGTGTACTAA TTACATAAAA   
  
  
- ATAACAGTTA TTTAAAATAT TATTTTGAAT ATTTAGAATA TTTAAATTAT CCTTTTTTTT TTCAGAAGAT   
  
  
- CTTAACTAAA ATATTATGTG GTATGGTTCT AGTGTACTTA ACGTACACAG CCACCTGTTC CAACGGTTTT   
  
  
- AAGGAATAAG GATATGTCTT AGAAAAGTAA CAATATAATA GTATCAGCTT TGGGTCATCA TATATTTCTC   
  
  
- CAATGCTCAC CTCCTACCTA TACTTTATTT ATTCTCCGGA ATCTCTAGTC CCACACTCTG TACCTAAACC   
  
  
- GTTTACCACT CTCTAGATCA CTCG

+     TGACG-motif

| Site Name | Organism | Position | Strand | Matrix score. | sequence | function |
| --- | --- | --- | --- | --- | --- | --- |
| TGACG-motif | Hordeum vulgare | 2106 | + | 5 | TGACG | cis-acting regulatory element involved in the MeJA-responsiveness |
| TGACG-motif | Hordeum vulgare | 2608 | - | 5 | TGACG | cis-acting regulatory element involved in the MeJA-responsiveness |

>PlantCARE\_32120   
+ AATCATTAAA GTCTAATTCT TCAAAATTCC GAATTCTAAA TTTAGCTATT TAAAATTCCA ATTGTCCTAT   
  
  
+ CTCCGAACTT AATTTTCAGT TTCCCATGCT CCAATTCCCG TATTTACCCC GCTACTTATT ATTATCATTA   
  
  
+ TCATTATCAC CTTATTCATT ATTTCTATAA ATTCCGCCTT CGAATGATTT CTAAGATCTC CAATTTTTAT   
  
  
+ ACATCAACTT TCATAATTTC TACGTCTCAA ATAACATACG ATTCTGATTC CTTTCAAAAT TTAACTCCCA   
  
  
+ TAATTCCAAT TTTCGTAACT TAATTTTTCT TAAAAATCCC GAACTTTCAA AATCCCAATT TTCTTTCAAA   
  
  
+ TTTAATTTCT AAATGTTCTC ATTCTTACAT CTAATTCCTA AGAATCCAAT TTTCAAATAA TCTCTAAGAT   
  
  
+ TCCGATTCTC AAATGAATTT CAAAAATCCA GTTTCCCTTC GAACAATTTT AATCCCTGTT TAGTGATGCA   
  
  
+ TGTAATATGT TTTGTGCTCT ATATGGTGTA CTAACCCTCT CTATTGATAG CGCATGGTGG TTCGTGGCCC   
  
  
+ AGGTATGTAC CCATTTTCTC TCTAATCGTT GTCTATGCAT GTCTCGATTC TTATGTGTGC ACGATCATTC   
  
  
+ AGGATATTCA TTGATTTACT CATCAATTGT CACGGCAGCT TCATTTTATT AGTAGAGACC CGACTTTAGG   
  
  
+ GACTTAGAGG GGTGCTACGG TCCTTACCGT ACCTTCCTGA TAAGTAACCT GACCCCCGAA CCCGATCCGG   
  
  
+ TTTTTCACAG ACCATCTTTT CCAAAATAAG GAGTCACACT TAGGGTTTTT CTTTCTTATT TTGTTTACCC   
  
  
+ TTTAAAAATA AAACAAAAAT AAGTGGCGAC TCCAAGTCAT TTTTCTTAAT CAATAAAGAT CATTTTTTCG   
  
  
+ AAAACGAAAA TCGAGCTCGC CATCGAGTGG GAAACGCATT GAGCCGAAAT GCGGGGTCCA TAGGTATGTT   
  
  
+ TTTTATTTAT GAGATAAAAA GTAAATAATA TTTTTTTCGG GTTTAAAAAA TATATTATTG ATTACTTGTA   
  
  
+ ATTATATGAG CCTCGTTTGT ACTTATTATT AAATACATAT TCAAGGTAAA ATTATCCTTT AAAAAATTCT   
  
  
+ TAGGCCGAGG GAACCTTCCT ATTAATATAA GTCATCCAGC CCACCTTATG GGGGTAATTC TCCCCATATA   
  
  
+ CCATGGGTCC AAATCGGCGG CCCGGTCAGT TCAACGACAG TACGATGGCA CTCTGAACCC TGTGCTGCAT   
  
  
+ CTGCTCACAA AAAGTACACC ACAGTTGTCT TTCACTCTCT CTGCCCATTC ACTATGCAAA CACGTTTATA   
  
  
+ AAATTGGTTA TTTTATAAAC GTTTCTCTTT CAAATGGTTA TTTTATATTA GTAACAAAAA TATTATAATA   
  
  
+ ATAAAAAAAT CAATATTACG TAATTGCTAA AAATGAAATT ACATCATGTT GTAAGATATT TCATTTGCCT   
  
  
+ ATTAATAATT TTTTATTATT TATAAAATTG GGTTGAATTT CAATATTTTT TAAAATAAGT CTTATTTATT   
  
  
+ TAAGTGAAAT TTTTTTTATA GATAAAGTCA CAAAAATCAT TTTTCTAAAT AATTTATAAA AAGATACTTT   
  
  
+ TCCAATAAAA ATTAAATTAA AATGCTTTTA TAAATAAAAT TGTAACAATT CATTCCATAG CAAAGGTAAA   
  
  
+ TAAAGCCATT TTCTTATCAA TTAAATCAAG CTTCTCTTTT TTATGAAATT GTTTTTAATA AATTCTTTTA   
  
  
+ GTAACAATAA GATTTTGTTC ATATTTTAAT ACATCTCCTT GGCAATTGGG CAAAGATGGC TGGTAGTGAC   
  
  
+ ATGAATCCAA GCATGAATTA GTCCGGGCGT TGTTACTTGC TACAAGGGTT ATGAGCATGG AAGGAAAAAT   
  
  
+ AATTAAAAAA TAAAAAAAAT TAAATTACTT CAATCACAAA GTTGAAAATA ATAAAATTGT GTTTGGAAGT   
  
  
+ ATTTGTACTT AAAGTTTTTT AGTAAAAGTG TTTCCCTAAA AGGTGTTTTT TTTAGAGAAA GCAGCTATAA   
  
  
+ AATGTTTTTT CAAAAATATT TTTTTATTTA AAAACACTTT CTTAAATCAC TTGTTATAAG TCACCTTAGA   
  
  
+ ATGACTGACG GTGTTGCACA TATCAAAAAG CATTTATGCC CTAGTGCCAT TTTATTATTT TATATCTTAA   
  
  
+ TTTTAGATAT TTTTATTTTT ATTTAATGAT TTATATTTGA ACGTTAATTT TCAATATATC ATATTGTATC   
  
  
+ TTTTATAGCC ACGTTTATTC AAATATATTT TTTATATTTA TAAATATTTT TTCTAAAATA TGATTCGAGA   
  
  
+ ATGCTGACAT GTGAATCGCA TGGGGGTAGG GCAAGCCATC TTGGACCCAT TAAACTTCAA ATTCCATGAT   
  
  
+ TGACTAGGAC TTGCCCAAAA ATTTCATTAC CCCAGATGCC CACATGTGAA TGCAGTCTCT GACAGAGTAG   
  
  
+ GTGGGAATAA ACCAAAATGC ATTTATTTAT TTTTCAAGAT TTTAATTATT ATTCACCTAA AATCCTTCAT   
  
  
+ AGGGATTAAA ATATTAGACC ATTGGATTTG CTCTCCTCAA TAGGGTTGAA CTTTGATTAG TACGTTTATA   
  
  
+ TTATTTTTTT CCTTTTTCGT CAAAATGCAT TCTGTCACAA AATTAATGTA GTCAAGGAAT TGACAAGTGA   
  
  
+ GAAGAGAGTC ACTTTAAATG ATTCAGCTGT CCACATTAAA GATAAGTCAC ATTTGTGGTC CATACTCCCC   
  
  
+ AAGACTTTAA TTTGAATTTA GCTCAGACTT TTCGTATTTT ATTTGTGTTT TTCTATTTAA GCTATCAATA   
  
  
+ CATTTATAAA TAAGTGATTT TAATATAAAA GATCAAGTAA AAAACAAATA AAGTACATAA AATATTTTTA   
  
  
+ TTCAATTACA TATTGTATCA AATTTTCAAG AGCAAAATTT ATAGCATTTT TCAATGTTTT GGAAATCAAA   
  
  
+ AAAGTAATAT ATTATCAGTT CACTAGTTAA ATTGGTGATT GAACCATAGA TGATGTCATA ATATATATTT   
  
  
+ TATATTTATT AAAATTAAAA ATATTTATAA GAAATTTTAA ATATATGAGA AATTAAAAGT CAATAATATT   
  
  
+ TTTTTCTTCA TCTTTAATAT TATCAAATGA ATATATATAT ATATATAAAT GTATTGATAT TTTAAAATTT   
  
  
+ TGTTAAATAA AATATATATA ATATGAAGAT ATTTATTGAA AGTTATTTAA TTTACCTATA TATATATTAA   
  
  
+ AAGTTATTTA TGGTTGTAAA AAATATTACA TTATTTTATT TATATTATTA ATATTACATT ATTTTATTTA   
  
  
+ TATTATTTAT AATTTCATTA TTATAAAAAG TTTGAAAATA TTATTGCTAT ATAATTTGTT GTTGTGTGAA   
  
  
+ GATCGGCAAA AAAACAGGAG TAGCAGTGTG GTGTCGTCCT TGCCCCTTGA ACTGGAGGTT GGTTTCCCAT   
  
  
+ TCCTCCCTCC CACGAATCCC ACATGTAAAG CAAATAAAAG AGCTTTACAT ACACATGATT AATGTATTTT   
  
  
+ TATTGTCAAT AAATTTTATA ATAAAACTTA TAAATCTTAT AAATTTAATA GGAAAAAAAA AAGTCTTCTA   
  
  
+ GAATTGATTT TATAATACAC CATACCAAGA TCACATGAAT TGCATGTGTC GGTGGACAAG GTTGCCAAAA   
  
  
+ TTCCTTATTC CTATACAGAA TCTTTTCATT GTTATATTAT CATAGTCGAA ACCCAGTAGT ATATAAAGAG   
  
  
+ GTTACGAGTG GAGGATGGAT ATGAAATAAA TAAGAGGCCT TAGAGATCAG GGTGTGAGAC ATGGATTTGG   
  
  
+ CAAATGGTGA GAGATCTAGT GAGC  

- TTAGTAATTT CAGATTAAGA AGTTTTAAGG CTTAAGATTT AAATCGATAA ATTTTAAGGT TAACAGGATA   
  
  
- GAGGCTTGAA TTAAAAGTCA AAGGGTACGA GGTTAAGGGC ATAAATGGGG CGATGAATAA TAATAGTAAT   
  
  
- AGTAATAGTG GAATAAGTAA TAAAGATATT TAAGGCGGAA GCTTACTAAA GATTCTAGAG GTTAAAAATA   
  
  
- TGTAGTTGAA AGTATTAAAG ATGCAGAGTT TATTGTATGC TAAGACTAAG GAAAGTTTTA AATTGAGGGT   
  
  
- ATTAAGGTTA AAAGCATTGA ATTAAAAAGA ATTTTTAGGG CTTGAAAGTT TTAGGGTTAA AAGAAAGTTT   
  
  
- AAATTAAAGA TTTACAAGAG TAAGAATGTA GATTAAGGAT TCTTAGGTTA AAAGTTTATT AGAGATTCTA   
  
  
- AGGCTAAGAG TTTACTTAAA GTTTTTAGGT CAAAGGGAAG CTTGTTAAAA TTAGGGACAA ATCACTACGT   
  
  
- ACATTATACA AAACACGAGA TATACCACAT GATTGGGAGA GATAACTATC GCGTACCACC AAGCACCGGG   
  
  
- TCCATACATG GGTAAAAGAG AGATTAGCAA CAGATACGTA CAGAGCTAAG AATACACACG TGCTAGTAAG   
  
  
- TCCTATAAGT AACTAAATGA GTAGTTAACA GTGCCGTCGA AGTAAAATAA TCATCTCTGG GCTGAAATCC   
  
  
- CTGAATCTCC CCACGATGCC AGGAATGGCA TGGAAGGACT ATTCATTGGA CTGGGGGCTT GGGCTAGGCC   
  
  
- AAAAAGTGTC TGGTAGAAAA GGTTTTATTC CTCAGTGTGA ATCCCAAAAA GAAAGAATAA AACAAATGGG   
  
  
- AAATTTTTAT TTTGTTTTTA TTCACCGCTG AGGTTCAGTA AAAAGAATTA GTTATTTCTA GTAAAAAAGC   
  
  
- TTTTGCTTTT AGCTCGAGCG GTAGCTCACC CTTTGCGTAA CTCGGCTTTA CGCCCCAGGT ATCCATACAA   
  
  
- AAAATAAATA CTCTATTTTT CATTTATTAT AAAAAAAGCC CAAATTTTTT ATATAATAAC TAATGAACAT   
  
  
- TAATATACTC GGAGCAAACA TGAATAATAA TTTATGTATA AGTTCCATTT TAATAGGAAA TTTTTTAAGA   
  
  
- ATCCGGCTCC CTTGGAAGGA TAATTATATT CAGTAGGTCG GGTGGAATAC CCCCATTAAG AGGGGTATAT   
  
  
- GGTACCCAGG TTTAGCCGCC GGGCCAGTCA AGTTGCTGTC ATGCTACCGT GAGACTTGGG ACACGACGTA   
  
  
- GACGAGTGTT TTTCATGTGG TGTCAACAGA AAGTGAGAGA GACGGGTAAG TGATACGTTT GTGCAAATAT   
  
  
- TTTAACCAAT AAAATATTTG CAAAGAGAAA GTTTACCAAT AAAATATAAT CATTGTTTTT ATAATATTAT   
  
  
- TATTTTTTTA GTTATAATGC ATTAACGATT TTTACTTTAA TGTAGTACAA CATTCTATAA AGTAAACGGA   
  
  
- TAATTATTAA AAAATAATAA ATATTTTAAC CCAACTTAAA GTTATAAAAA ATTTTATTCA GAATAAATAA   
  
  
- ATTCACTTTA AAAAAAATAT CTATTTCAGT GTTTTTAGTA AAAAGATTTA TTAAATATTT TTCTATGAAA   
  
  
- AGGTTATTTT TAATTTAATT TTACGAAAAT ATTTATTTTA ACATTGTTAA GTAAGGTATC GTTTCCATTT   
  
  
- ATTTCGGTAA AAGAATAGTT AATTTAGTTC GAAGAGAAAA AATACTTTAA CAAAAATTAT TTAAGAAAAT   
  
  
- CATTGTTATT CTAAAACAAG TATAAAATTA TGTAGAGGAA CCGTTAACCC GTTTCTACCG ACCATCACTG   
  
  
- TACTTAGGTT CGTACTTAAT CAGGCCCGCA ACAATGAACG ATGTTCCCAA TACTCGTACC TTCCTTTTTA   
  
  
- TTAATTTTTT ATTTTTTTTA ATTTAATGAA GTTAGTGTTT CAACTTTTAT TATTTTAACA CAAACCTTCA   
  
  
- TAAACATGAA TTTCAAAAAA TCATTTTCAC AAAGGGATTT TCCACAAAAA AAATCTCTTT CGTCGATATT   
  
  
- TTACAAAAAA GTTTTTATAA AAAAATAAAT TTTTGTGAAA GAATTTAGTG AACAATATTC AGTGGAATCT   
  
  
- TACTGACTGC CACAACGTGT ATAGTTTTTC GTAAATACGG GATCACGGTA AAATAATAAA ATATAGAATT   
  
  
- AAAATCTATA AAAATAAAAA TAAATTACTA AATATAAACT TGCAATTAAA AGTTATATAG TATAACATAG   
  
  
- AAAATATCGG TGCAAATAAG TTTATATAAA AAATATAAAT ATTTATAAAA AAGATTTTAT ACTAAGCTCT   
  
  
- TACGACTGTA CACTTAGCGT ACCCCCATCC CGTTCGGTAG AACCTGGGTA ATTTGAAGTT TAAGGTACTA   
  
  
- ACTGATCCTG AACGGGTTTT TAAAGTAATG GGGTCTACGG GTGTACACTT ACGTCAGAGA CTGTCTCATC   
  
  
- CACCCTTATT TGGTTTTACG TAAATAAATA AAAAGTTCTA AAATTAATAA TAAGTGGATT TTAGGAAGTA   
  
  
- TCCCTAATTT TATAATCTGG TAACCTAAAC GAGAGGAGTT ATCCCAACTT GAAACTAATC ATGCAAATAT   
  
  
- AATAAAAAAA GGAAAAAGCA GTTTTACGTA AGACAGTGTT TTAATTACAT CAGTTCCTTA ACTGTTCACT   
  
  
- CTTCTCTCAG TGAAATTTAC TAAGTCGACA GGTGTAATTT CTATTCAGTG TAAACACCAG GTATGAGGGG   
  
  
- TTCTGAAATT AAACTTAAAT CGAGTCTGAA AAGCATAAAA TAAACACAAA AAGATAAATT CGATAGTTAT   
  
  
- GTAAATATTT ATTCACTAAA ATTATATTTT CTAGTTCATT TTTTGTTTAT TTCATGTATT TTATAAAAAT   
  
  
- AAGTTAATGT ATAACATAGT TTAAAAGTTC TCGTTTTAAA TATCGTAAAA AGTTACAAAA CCTTTAGTTT   
  
  
- TTTCATTATA TAATAGTCAA GTGATCAATT TAACCACTAA CTTGGTATCT ACTACAGTAT TATATATAAA   
  
  
- ATATAAATAA TTTTAATTTT TATAAATATT CTTTAAAATT TATATACTCT TTAATTTTCA GTTATTATAA   
  
  
- AAAAAGAAGT AGAAATTATA ATAGTTTACT TATATATATA TATATATTTA CATAACTATA AAATTTTAAA   
  
  
- ACAATTTATT TTATATATAT TATACTTCTA TAAATAACTT TCAATAAATT AAATGGATAT ATATATAATT   
  
  
- TTCAATAAAT ACCAACATTT TTTATAATGT AATAAAATAA ATATAATAAT TATAATGTAA TAAAATAAAT   
  
  
- ATAATAAATA TTAAAGTAAT AATATTTTTC AAACTTTTAT AATAACGATA TATTAAACAA CAACACACTT   
  
  
- CTAGCCGTTT TTTTGTCCTC ATCGTCACAC CACAGCAGGA ACGGGGAACT TGACCTCCAA CCAAAGGGTA   
  
  
- AGGAGGGAGG GTGCTTAGGG TGTACATTTC GTTTATTTTC TCGAAATGTA TGTGTACTAA TTACATAAAA   
  
  
- ATAACAGTTA TTTAAAATAT TATTTTGAAT ATTTAGAATA TTTAAATTAT CCTTTTTTTT TTCAGAAGAT   
  
  
- CTTAACTAAA ATATTATGTG GTATGGTTCT AGTGTACTTA ACGTACACAG CCACCTGTTC CAACGGTTTT   
  
  
- AAGGAATAAG GATATGTCTT AGAAAAGTAA CAATATAATA GTATCAGCTT TGGGTCATCA TATATTTCTC   
  
  
- CAATGCTCAC CTCCTACCTA TACTTTATTT ATTCTCCGGA ATCTCTAGTC CCACACTCTG TACCTAAACC   
  
  
- GTTTACCACT CTCTAGATCA CTCG

+     Unnamed\_\_1

| Site Name | Organism | Position | Strand | Matrix score. | sequence | function |
| --- | --- | --- | --- | --- | --- | --- |
| Unnamed\_\_1 | Zea mays | 553 | + | 5 | CGTGG |  |
| Unnamed\_\_1 | Zea mays | 3440 | - | 5 | CGTGG |  |
| Unnamed\_\_1 | Zea mays | 2249 | - | 5 | CGTGG |  |

>PlantCARE\_32120   
+ AATCATTAAA GTCTAATTCT TCAAAATTCC GAATTCTAAA TTTAGCTATT TAAAATTCCA ATTGTCCTAT   
  
  
+ CTCCGAACTT AATTTTCAGT TTCCCATGCT CCAATTCCCG TATTTACCCC GCTACTTATT ATTATCATTA   
  
  
+ TCATTATCAC CTTATTCATT ATTTCTATAA ATTCCGCCTT CGAATGATTT CTAAGATCTC CAATTTTTAT   
  
  
+ ACATCAACTT TCATAATTTC TACGTCTCAA ATAACATACG ATTCTGATTC CTTTCAAAAT TTAACTCCCA   
  
  
+ TAATTCCAAT TTTCGTAACT TAATTTTTCT TAAAAATCCC GAACTTTCAA AATCCCAATT TTCTTTCAAA   
  
  
+ TTTAATTTCT AAATGTTCTC ATTCTTACAT CTAATTCCTA AGAATCCAAT TTTCAAATAA TCTCTAAGAT   
  
  
+ TCCGATTCTC AAATGAATTT CAAAAATCCA GTTTCCCTTC GAACAATTTT AATCCCTGTT TAGTGATGCA   
  
  
+ TGTAATATGT TTTGTGCTCT ATATGGTGTA CTAACCCTCT CTATTGATAG CGCATGGTGG TTCGTGGCCC   
  
  
+ AGGTATGTAC CCATTTTCTC TCTAATCGTT GTCTATGCAT GTCTCGATTC TTATGTGTGC ACGATCATTC   
  
  
+ AGGATATTCA TTGATTTACT CATCAATTGT CACGGCAGCT TCATTTTATT AGTAGAGACC CGACTTTAGG   
  
  
+ GACTTAGAGG GGTGCTACGG TCCTTACCGT ACCTTCCTGA TAAGTAACCT GACCCCCGAA CCCGATCCGG   
  
  
+ TTTTTCACAG ACCATCTTTT CCAAAATAAG GAGTCACACT TAGGGTTTTT CTTTCTTATT TTGTTTACCC   
  
  
+ TTTAAAAATA AAACAAAAAT AAGTGGCGAC TCCAAGTCAT TTTTCTTAAT CAATAAAGAT CATTTTTTCG   
  
  
+ AAAACGAAAA TCGAGCTCGC CATCGAGTGG GAAACGCATT GAGCCGAAAT GCGGGGTCCA TAGGTATGTT   
  
  
+ TTTTATTTAT GAGATAAAAA GTAAATAATA TTTTTTTCGG GTTTAAAAAA TATATTATTG ATTACTTGTA   
  
  
+ ATTATATGAG CCTCGTTTGT ACTTATTATT AAATACATAT TCAAGGTAAA ATTATCCTTT AAAAAATTCT   
  
  
+ TAGGCCGAGG GAACCTTCCT ATTAATATAA GTCATCCAGC CCACCTTATG GGGGTAATTC TCCCCATATA   
  
  
+ CCATGGGTCC AAATCGGCGG CCCGGTCAGT TCAACGACAG TACGATGGCA CTCTGAACCC TGTGCTGCAT   
  
  
+ CTGCTCACAA AAAGTACACC ACAGTTGTCT TTCACTCTCT CTGCCCATTC ACTATGCAAA CACGTTTATA   
  
  
+ AAATTGGTTA TTTTATAAAC GTTTCTCTTT CAAATGGTTA TTTTATATTA GTAACAAAAA TATTATAATA   
  
  
+ ATAAAAAAAT CAATATTACG TAATTGCTAA AAATGAAATT ACATCATGTT GTAAGATATT TCATTTGCCT   
  
  
+ ATTAATAATT TTTTATTATT TATAAAATTG GGTTGAATTT CAATATTTTT TAAAATAAGT CTTATTTATT   
  
  
+ TAAGTGAAAT TTTTTTTATA GATAAAGTCA CAAAAATCAT TTTTCTAAAT AATTTATAAA AAGATACTTT   
  
  
+ TCCAATAAAA ATTAAATTAA AATGCTTTTA TAAATAAAAT TGTAACAATT CATTCCATAG CAAAGGTAAA   
  
  
+ TAAAGCCATT TTCTTATCAA TTAAATCAAG CTTCTCTTTT TTATGAAATT GTTTTTAATA AATTCTTTTA   
  
  
+ GTAACAATAA GATTTTGTTC ATATTTTAAT ACATCTCCTT GGCAATTGGG CAAAGATGGC TGGTAGTGAC   
  
  
+ ATGAATCCAA GCATGAATTA GTCCGGGCGT TGTTACTTGC TACAAGGGTT ATGAGCATGG AAGGAAAAAT   
  
  
+ AATTAAAAAA TAAAAAAAAT TAAATTACTT CAATCACAAA GTTGAAAATA ATAAAATTGT GTTTGGAAGT   
  
  
+ ATTTGTACTT AAAGTTTTTT AGTAAAAGTG TTTCCCTAAA AGGTGTTTTT TTTAGAGAAA GCAGCTATAA   
  
  
+ AATGTTTTTT CAAAAATATT TTTTTATTTA AAAACACTTT CTTAAATCAC TTGTTATAAG TCACCTTAGA   
  
  
+ ATGACTGACG GTGTTGCACA TATCAAAAAG CATTTATGCC CTAGTGCCAT TTTATTATTT TATATCTTAA   
  
  
+ TTTTAGATAT TTTTATTTTT ATTTAATGAT TTATATTTGA ACGTTAATTT TCAATATATC ATATTGTATC   
  
  
+ TTTTATAGCC ACGTTTATTC AAATATATTT TTTATATTTA TAAATATTTT TTCTAAAATA TGATTCGAGA   
  
  
+ ATGCTGACAT GTGAATCGCA TGGGGGTAGG GCAAGCCATC TTGGACCCAT TAAACTTCAA ATTCCATGAT   
  
  
+ TGACTAGGAC TTGCCCAAAA ATTTCATTAC CCCAGATGCC CACATGTGAA TGCAGTCTCT GACAGAGTAG   
  
  
+ GTGGGAATAA ACCAAAATGC ATTTATTTAT TTTTCAAGAT TTTAATTATT ATTCACCTAA AATCCTTCAT   
  
  
+ AGGGATTAAA ATATTAGACC ATTGGATTTG CTCTCCTCAA TAGGGTTGAA CTTTGATTAG TACGTTTATA   
  
  
+ TTATTTTTTT CCTTTTTCGT CAAAATGCAT TCTGTCACAA AATTAATGTA GTCAAGGAAT TGACAAGTGA   
  
  
+ GAAGAGAGTC ACTTTAAATG ATTCAGCTGT CCACATTAAA GATAAGTCAC ATTTGTGGTC CATACTCCCC   
  
  
+ AAGACTTTAA TTTGAATTTA GCTCAGACTT TTCGTATTTT ATTTGTGTTT TTCTATTTAA GCTATCAATA   
  
  
+ CATTTATAAA TAAGTGATTT TAATATAAAA GATCAAGTAA AAAACAAATA AAGTACATAA AATATTTTTA   
  
  
+ TTCAATTACA TATTGTATCA AATTTTCAAG AGCAAAATTT ATAGCATTTT TCAATGTTTT GGAAATCAAA   
  
  
+ AAAGTAATAT ATTATCAGTT CACTAGTTAA ATTGGTGATT GAACCATAGA TGATGTCATA ATATATATTT   
  
  
+ TATATTTATT AAAATTAAAA ATATTTATAA GAAATTTTAA ATATATGAGA AATTAAAAGT CAATAATATT   
  
  
+ TTTTTCTTCA TCTTTAATAT TATCAAATGA ATATATATAT ATATATAAAT GTATTGATAT TTTAAAATTT   
  
  
+ TGTTAAATAA AATATATATA ATATGAAGAT ATTTATTGAA AGTTATTTAA TTTACCTATA TATATATTAA   
  
  
+ AAGTTATTTA TGGTTGTAAA AAATATTACA TTATTTTATT TATATTATTA ATATTACATT ATTTTATTTA   
  
  
+ TATTATTTAT AATTTCATTA TTATAAAAAG TTTGAAAATA TTATTGCTAT ATAATTTGTT GTTGTGTGAA   
  
  
+ GATCGGCAAA AAAACAGGAG TAGCAGTGTG GTGTCGTCCT TGCCCCTTGA ACTGGAGGTT GGTTTCCCAT   
  
  
+ TCCTCCCTCC CACGAATCCC ACATGTAAAG CAAATAAAAG AGCTTTACAT ACACATGATT AATGTATTTT   
  
  
+ TATTGTCAAT AAATTTTATA ATAAAACTTA TAAATCTTAT AAATTTAATA GGAAAAAAAA AAGTCTTCTA   
  
  
+ GAATTGATTT TATAATACAC CATACCAAGA TCACATGAAT TGCATGTGTC GGTGGACAAG GTTGCCAAAA   
  
  
+ TTCCTTATTC CTATACAGAA TCTTTTCATT GTTATATTAT CATAGTCGAA ACCCAGTAGT ATATAAAGAG   
  
  
+ GTTACGAGTG GAGGATGGAT ATGAAATAAA TAAGAGGCCT TAGAGATCAG GGTGTGAGAC ATGGATTTGG   
  
  
+ CAAATGGTGA GAGATCTAGT GAGC  

- TTAGTAATTT CAGATTAAGA AGTTTTAAGG CTTAAGATTT AAATCGATAA ATTTTAAGGT TAACAGGATA   
  
  
- GAGGCTTGAA TTAAAAGTCA AAGGGTACGA GGTTAAGGGC ATAAATGGGG CGATGAATAA TAATAGTAAT   
  
  
- AGTAATAGTG GAATAAGTAA TAAAGATATT TAAGGCGGAA GCTTACTAAA GATTCTAGAG GTTAAAAATA   
  
  
- TGTAGTTGAA AGTATTAAAG ATGCAGAGTT TATTGTATGC TAAGACTAAG GAAAGTTTTA AATTGAGGGT   
  
  
- ATTAAGGTTA AAAGCATTGA ATTAAAAAGA ATTTTTAGGG CTTGAAAGTT TTAGGGTTAA AAGAAAGTTT   
  
  
- AAATTAAAGA TTTACAAGAG TAAGAATGTA GATTAAGGAT TCTTAGGTTA AAAGTTTATT AGAGATTCTA   
  
  
- AGGCTAAGAG TTTACTTAAA GTTTTTAGGT CAAAGGGAAG CTTGTTAAAA TTAGGGACAA ATCACTACGT   
  
  
- ACATTATACA AAACACGAGA TATACCACAT GATTGGGAGA GATAACTATC GCGTACCACC AAGCACCGGG   
  
  
- TCCATACATG GGTAAAAGAG AGATTAGCAA CAGATACGTA CAGAGCTAAG AATACACACG TGCTAGTAAG   
  
  
- TCCTATAAGT AACTAAATGA GTAGTTAACA GTGCCGTCGA AGTAAAATAA TCATCTCTGG GCTGAAATCC   
  
  
- CTGAATCTCC CCACGATGCC AGGAATGGCA TGGAAGGACT ATTCATTGGA CTGGGGGCTT GGGCTAGGCC   
  
  
- AAAAAGTGTC TGGTAGAAAA GGTTTTATTC CTCAGTGTGA ATCCCAAAAA GAAAGAATAA AACAAATGGG   
  
  
- AAATTTTTAT TTTGTTTTTA TTCACCGCTG AGGTTCAGTA AAAAGAATTA GTTATTTCTA GTAAAAAAGC   
  
  
- TTTTGCTTTT AGCTCGAGCG GTAGCTCACC CTTTGCGTAA CTCGGCTTTA CGCCCCAGGT ATCCATACAA   
  
  
- AAAATAAATA CTCTATTTTT CATTTATTAT AAAAAAAGCC CAAATTTTTT ATATAATAAC TAATGAACAT   
  
  
- TAATATACTC GGAGCAAACA TGAATAATAA TTTATGTATA AGTTCCATTT TAATAGGAAA TTTTTTAAGA   
  
  
- ATCCGGCTCC CTTGGAAGGA TAATTATATT CAGTAGGTCG GGTGGAATAC CCCCATTAAG AGGGGTATAT   
  
  
- GGTACCCAGG TTTAGCCGCC GGGCCAGTCA AGTTGCTGTC ATGCTACCGT GAGACTTGGG ACACGACGTA   
  
  
- GACGAGTGTT TTTCATGTGG TGTCAACAGA AAGTGAGAGA GACGGGTAAG TGATACGTTT GTGCAAATAT   
  
  
- TTTAACCAAT AAAATATTTG CAAAGAGAAA GTTTACCAAT AAAATATAAT CATTGTTTTT ATAATATTAT   
  
  
- TATTTTTTTA GTTATAATGC ATTAACGATT TTTACTTTAA TGTAGTACAA CATTCTATAA AGTAAACGGA   
  
  
- TAATTATTAA AAAATAATAA ATATTTTAAC CCAACTTAAA GTTATAAAAA ATTTTATTCA GAATAAATAA   
  
  
- ATTCACTTTA AAAAAAATAT CTATTTCAGT GTTTTTAGTA AAAAGATTTA TTAAATATTT TTCTATGAAA   
  
  
- AGGTTATTTT TAATTTAATT TTACGAAAAT ATTTATTTTA ACATTGTTAA GTAAGGTATC GTTTCCATTT   
  
  
- ATTTCGGTAA AAGAATAGTT AATTTAGTTC GAAGAGAAAA AATACTTTAA CAAAAATTAT TTAAGAAAAT   
  
  
- CATTGTTATT CTAAAACAAG TATAAAATTA TGTAGAGGAA CCGTTAACCC GTTTCTACCG ACCATCACTG   
  
  
- TACTTAGGTT CGTACTTAAT CAGGCCCGCA ACAATGAACG ATGTTCCCAA TACTCGTACC TTCCTTTTTA   
  
  
- TTAATTTTTT ATTTTTTTTA ATTTAATGAA GTTAGTGTTT CAACTTTTAT TATTTTAACA CAAACCTTCA   
  
  
- TAAACATGAA TTTCAAAAAA TCATTTTCAC AAAGGGATTT TCCACAAAAA AAATCTCTTT CGTCGATATT   
  
  
- TTACAAAAAA GTTTTTATAA AAAAATAAAT TTTTGTGAAA GAATTTAGTG AACAATATTC AGTGGAATCT   
  
  
- TACTGACTGC CACAACGTGT ATAGTTTTTC GTAAATACGG GATCACGGTA AAATAATAAA ATATAGAATT   
  
  
- AAAATCTATA AAAATAAAAA TAAATTACTA AATATAAACT TGCAATTAAA AGTTATATAG TATAACATAG   
  
  
- AAAATATCGG TGCAAATAAG TTTATATAAA AAATATAAAT ATTTATAAAA AAGATTTTAT ACTAAGCTCT   
  
  
- TACGACTGTA CACTTAGCGT ACCCCCATCC CGTTCGGTAG AACCTGGGTA ATTTGAAGTT TAAGGTACTA   
  
  
- ACTGATCCTG AACGGGTTTT TAAAGTAATG GGGTCTACGG GTGTACACTT ACGTCAGAGA CTGTCTCATC   
  
  
- CACCCTTATT TGGTTTTACG TAAATAAATA AAAAGTTCTA AAATTAATAA TAAGTGGATT TTAGGAAGTA   
  
  
- TCCCTAATTT TATAATCTGG TAACCTAAAC GAGAGGAGTT ATCCCAACTT GAAACTAATC ATGCAAATAT   
  
  
- AATAAAAAAA GGAAAAAGCA GTTTTACGTA AGACAGTGTT TTAATTACAT CAGTTCCTTA ACTGTTCACT   
  
  
- CTTCTCTCAG TGAAATTTAC TAAGTCGACA GGTGTAATTT CTATTCAGTG TAAACACCAG GTATGAGGGG   
  
  
- TTCTGAAATT AAACTTAAAT CGAGTCTGAA AAGCATAAAA TAAACACAAA AAGATAAATT CGATAGTTAT   
  
  
- GTAAATATTT ATTCACTAAA ATTATATTTT CTAGTTCATT TTTTGTTTAT TTCATGTATT TTATAAAAAT   
  
  
- AAGTTAATGT ATAACATAGT TTAAAAGTTC TCGTTTTAAA TATCGTAAAA AGTTACAAAA CCTTTAGTTT   
  
  
- TTTCATTATA TAATAGTCAA GTGATCAATT TAACCACTAA CTTGGTATCT ACTACAGTAT TATATATAAA   
  
  
- ATATAAATAA TTTTAATTTT TATAAATATT CTTTAAAATT TATATACTCT TTAATTTTCA GTTATTATAA   
  
  
- AAAAAGAAGT AGAAATTATA ATAGTTTACT TATATATATA TATATATTTA CATAACTATA AAATTTTAAA   
  
  
- ACAATTTATT TTATATATAT TATACTTCTA TAAATAACTT TCAATAAATT AAATGGATAT ATATATAATT   
  
  
- TTCAATAAAT ACCAACATTT TTTATAATGT AATAAAATAA ATATAATAAT TATAATGTAA TAAAATAAAT   
  
  
- ATAATAAATA TTAAAGTAAT AATATTTTTC AAACTTTTAT AATAACGATA TATTAAACAA CAACACACTT   
  
  
- CTAGCCGTTT TTTTGTCCTC ATCGTCACAC CACAGCAGGA ACGGGGAACT TGACCTCCAA CCAAAGGGTA   
  
  
- AGGAGGGAGG GTGCTTAGGG TGTACATTTC GTTTATTTTC TCGAAATGTA TGTGTACTAA TTACATAAAA   
  
  
- ATAACAGTTA TTTAAAATAT TATTTTGAAT ATTTAGAATA TTTAAATTAT CCTTTTTTTT TTCAGAAGAT   
  
  
- CTTAACTAAA ATATTATGTG GTATGGTTCT AGTGTACTTA ACGTACACAG CCACCTGTTC CAACGGTTTT   
  
  
- AAGGAATAAG GATATGTCTT AGAAAAGTAA CAATATAATA GTATCAGCTT TGGGTCATCA TATATTTCTC   
  
  
- CAATGCTCAC CTCCTACCTA TACTTTATTT ATTCTCCGGA ATCTCTAGTC CCACACTCTG TACCTAAACC   
  
  
- GTTTACCACT CTCTAGATCA CTCG

+     Unnamed\_\_4

| Site Name | Organism | Position | Strand | Matrix score. | sequence | function |
| --- | --- | --- | --- | --- | --- | --- |
| Unnamed\_\_4 | Petroselinum hortense | 71 | + | 4 | CTCC |  |
| Unnamed\_\_4 | Petroselinum hortense | 2553 | + | 4 | CTCC |  |
| Unnamed\_\_4 | Petroselinum hortense | 800 | - | 4 | CTCC |  |
| Unnamed\_\_4 | Petroselinum hortense | 3433 | + | 4 | CTCC |  |
| Unnamed\_\_4 | Petroselinum hortense | 198 | + | 4 | CTCC |  |
| Unnamed\_\_4 | Petroselinum hortense | 3377 | - | 4 | CTCC |  |
| Unnamed\_\_4 | Petroselinum hortense | 1180 | + | 4 | CTCC |  |
| Unnamed\_\_4 | Petroselinum hortense | 3720 | - | 4 | CTCC |  |
| Unnamed\_\_4 | Petroselinum hortense | 99 | + | 4 | CTCC |  |
| Unnamed\_\_4 | Petroselinum hortense | 2725 | + | 4 | CTCC |  |
| Unnamed\_\_4 | Petroselinum hortense | 870 | + | 4 | CTCC |  |
| Unnamed\_\_4 | Petroselinum hortense | 3437 | + | 4 | CTCC |  |
| Unnamed\_\_4 | Petroselinum hortense | 275 | + | 4 | CTCC |  |
| Unnamed\_\_4 | Petroselinum hortense | 3414 | - | 4 | CTCC |  |
| Unnamed\_\_4 | Petroselinum hortense | 1785 | + | 4 | CTCC |  |

>PlantCARE\_32120   
+ AATCATTAAA GTCTAATTCT TCAAAATTCC GAATTCTAAA TTTAGCTATT TAAAATTCCA ATTGTCCTAT   
  
  
+ CTCCGAACTT AATTTTCAGT TTCCCATGCT CCAATTCCCG TATTTACCCC GCTACTTATT ATTATCATTA   
  
  
+ TCATTATCAC CTTATTCATT ATTTCTATAA ATTCCGCCTT CGAATGATTT CTAAGATCTC CAATTTTTAT   
  
  
+ ACATCAACTT TCATAATTTC TACGTCTCAA ATAACATACG ATTCTGATTC CTTTCAAAAT TTAACTCCCA   
  
  
+ TAATTCCAAT TTTCGTAACT TAATTTTTCT TAAAAATCCC GAACTTTCAA AATCCCAATT TTCTTTCAAA   
  
  
+ TTTAATTTCT AAATGTTCTC ATTCTTACAT CTAATTCCTA AGAATCCAAT TTTCAAATAA TCTCTAAGAT   
  
  
+ TCCGATTCTC AAATGAATTT CAAAAATCCA GTTTCCCTTC GAACAATTTT AATCCCTGTT TAGTGATGCA   
  
  
+ TGTAATATGT TTTGTGCTCT ATATGGTGTA CTAACCCTCT CTATTGATAG CGCATGGTGG TTCGTGGCCC   
  
  
+ AGGTATGTAC CCATTTTCTC TCTAATCGTT GTCTATGCAT GTCTCGATTC TTATGTGTGC ACGATCATTC   
  
  
+ AGGATATTCA TTGATTTACT CATCAATTGT CACGGCAGCT TCATTTTATT AGTAGAGACC CGACTTTAGG   
  
  
+ GACTTAGAGG GGTGCTACGG TCCTTACCGT ACCTTCCTGA TAAGTAACCT GACCCCCGAA CCCGATCCGG   
  
  
+ TTTTTCACAG ACCATCTTTT CCAAAATAAG GAGTCACACT TAGGGTTTTT CTTTCTTATT TTGTTTACCC   
  
  
+ TTTAAAAATA AAACAAAAAT AAGTGGCGAC TCCAAGTCAT TTTTCTTAAT CAATAAAGAT CATTTTTTCG   
  
  
+ AAAACGAAAA TCGAGCTCGC CATCGAGTGG GAAACGCATT GAGCCGAAAT GCGGGGTCCA TAGGTATGTT   
  
  
+ TTTTATTTAT GAGATAAAAA GTAAATAATA TTTTTTTCGG GTTTAAAAAA TATATTATTG ATTACTTGTA   
  
  
+ ATTATATGAG CCTCGTTTGT ACTTATTATT AAATACATAT TCAAGGTAAA ATTATCCTTT AAAAAATTCT   
  
  
+ TAGGCCGAGG GAACCTTCCT ATTAATATAA GTCATCCAGC CCACCTTATG GGGGTAATTC TCCCCATATA   
  
  
+ CCATGGGTCC AAATCGGCGG CCCGGTCAGT TCAACGACAG TACGATGGCA CTCTGAACCC TGTGCTGCAT   
  
  
+ CTGCTCACAA AAAGTACACC ACAGTTGTCT TTCACTCTCT CTGCCCATTC ACTATGCAAA CACGTTTATA   
  
  
+ AAATTGGTTA TTTTATAAAC GTTTCTCTTT CAAATGGTTA TTTTATATTA GTAACAAAAA TATTATAATA   
  
  
+ ATAAAAAAAT CAATATTACG TAATTGCTAA AAATGAAATT ACATCATGTT GTAAGATATT TCATTTGCCT   
  
  
+ ATTAATAATT TTTTATTATT TATAAAATTG GGTTGAATTT CAATATTTTT TAAAATAAGT CTTATTTATT   
  
  
+ TAAGTGAAAT TTTTTTTATA GATAAAGTCA CAAAAATCAT TTTTCTAAAT AATTTATAAA AAGATACTTT   
  
  
+ TCCAATAAAA ATTAAATTAA AATGCTTTTA TAAATAAAAT TGTAACAATT CATTCCATAG CAAAGGTAAA   
  
  
+ TAAAGCCATT TTCTTATCAA TTAAATCAAG CTTCTCTTTT TTATGAAATT GTTTTTAATA AATTCTTTTA   
  
  
+ GTAACAATAA GATTTTGTTC ATATTTTAAT ACATCTCCTT GGCAATTGGG CAAAGATGGC TGGTAGTGAC   
  
  
+ ATGAATCCAA GCATGAATTA GTCCGGGCGT TGTTACTTGC TACAAGGGTT ATGAGCATGG AAGGAAAAAT   
  
  
+ AATTAAAAAA TAAAAAAAAT TAAATTACTT CAATCACAAA GTTGAAAATA ATAAAATTGT GTTTGGAAGT   
  
  
+ ATTTGTACTT AAAGTTTTTT AGTAAAAGTG TTTCCCTAAA AGGTGTTTTT TTTAGAGAAA GCAGCTATAA   
  
  
+ AATGTTTTTT CAAAAATATT TTTTTATTTA AAAACACTTT CTTAAATCAC TTGTTATAAG TCACCTTAGA   
  
  
+ ATGACTGACG GTGTTGCACA TATCAAAAAG CATTTATGCC CTAGTGCCAT TTTATTATTT TATATCTTAA   
  
  
+ TTTTAGATAT TTTTATTTTT ATTTAATGAT TTATATTTGA ACGTTAATTT TCAATATATC ATATTGTATC   
  
  
+ TTTTATAGCC ACGTTTATTC AAATATATTT TTTATATTTA TAAATATTTT TTCTAAAATA TGATTCGAGA   
  
  
+ ATGCTGACAT GTGAATCGCA TGGGGGTAGG GCAAGCCATC TTGGACCCAT TAAACTTCAA ATTCCATGAT   
  
  
+ TGACTAGGAC TTGCCCAAAA ATTTCATTAC CCCAGATGCC CACATGTGAA TGCAGTCTCT GACAGAGTAG   
  
  
+ GTGGGAATAA ACCAAAATGC ATTTATTTAT TTTTCAAGAT TTTAATTATT ATTCACCTAA AATCCTTCAT   
  
  
+ AGGGATTAAA ATATTAGACC ATTGGATTTG CTCTCCTCAA TAGGGTTGAA CTTTGATTAG TACGTTTATA   
  
  
+ TTATTTTTTT CCTTTTTCGT CAAAATGCAT TCTGTCACAA AATTAATGTA GTCAAGGAAT TGACAAGTGA   
  
  
+ GAAGAGAGTC ACTTTAAATG ATTCAGCTGT CCACATTAAA GATAAGTCAC ATTTGTGGTC CATACTCCCC   
  
  
+ AAGACTTTAA TTTGAATTTA GCTCAGACTT TTCGTATTTT ATTTGTGTTT TTCTATTTAA GCTATCAATA   
  
  
+ CATTTATAAA TAAGTGATTT TAATATAAAA GATCAAGTAA AAAACAAATA AAGTACATAA AATATTTTTA   
  
  
+ TTCAATTACA TATTGTATCA AATTTTCAAG AGCAAAATTT ATAGCATTTT TCAATGTTTT GGAAATCAAA   
  
  
+ AAAGTAATAT ATTATCAGTT CACTAGTTAA ATTGGTGATT GAACCATAGA TGATGTCATA ATATATATTT   
  
  
+ TATATTTATT AAAATTAAAA ATATTTATAA GAAATTTTAA ATATATGAGA AATTAAAAGT CAATAATATT   
  
  
+ TTTTTCTTCA TCTTTAATAT TATCAAATGA ATATATATAT ATATATAAAT GTATTGATAT TTTAAAATTT   
  
  
+ TGTTAAATAA AATATATATA ATATGAAGAT ATTTATTGAA AGTTATTTAA TTTACCTATA TATATATTAA   
  
  
+ AAGTTATTTA TGGTTGTAAA AAATATTACA TTATTTTATT TATATTATTA ATATTACATT ATTTTATTTA   
  
  
+ TATTATTTAT AATTTCATTA TTATAAAAAG TTTGAAAATA TTATTGCTAT ATAATTTGTT GTTGTGTGAA   
  
  
+ GATCGGCAAA AAAACAGGAG TAGCAGTGTG GTGTCGTCCT TGCCCCTTGA ACTGGAGGTT GGTTTCCCAT   
  
  
+ TCCTCCCTCC CACGAATCCC ACATGTAAAG CAAATAAAAG AGCTTTACAT ACACATGATT AATGTATTTT   
  
  
+ TATTGTCAAT AAATTTTATA ATAAAACTTA TAAATCTTAT AAATTTAATA GGAAAAAAAA AAGTCTTCTA   
  
  
+ GAATTGATTT TATAATACAC CATACCAAGA TCACATGAAT TGCATGTGTC GGTGGACAAG GTTGCCAAAA   
  
  
+ TTCCTTATTC CTATACAGAA TCTTTTCATT GTTATATTAT CATAGTCGAA ACCCAGTAGT ATATAAAGAG   
  
  
+ GTTACGAGTG GAGGATGGAT ATGAAATAAA TAAGAGGCCT TAGAGATCAG GGTGTGAGAC ATGGATTTGG   
  
  
+ CAAATGGTGA GAGATCTAGT GAGC  

- TTAGTAATTT CAGATTAAGA AGTTTTAAGG CTTAAGATTT AAATCGATAA ATTTTAAGGT TAACAGGATA   
  
  
- GAGGCTTGAA TTAAAAGTCA AAGGGTACGA GGTTAAGGGC ATAAATGGGG CGATGAATAA TAATAGTAAT   
  
  
- AGTAATAGTG GAATAAGTAA TAAAGATATT TAAGGCGGAA GCTTACTAAA GATTCTAGAG GTTAAAAATA   
  
  
- TGTAGTTGAA AGTATTAAAG ATGCAGAGTT TATTGTATGC TAAGACTAAG GAAAGTTTTA AATTGAGGGT   
  
  
- ATTAAGGTTA AAAGCATTGA ATTAAAAAGA ATTTTTAGGG CTTGAAAGTT TTAGGGTTAA AAGAAAGTTT   
  
  
- AAATTAAAGA TTTACAAGAG TAAGAATGTA GATTAAGGAT TCTTAGGTTA AAAGTTTATT AGAGATTCTA   
  
  
- AGGCTAAGAG TTTACTTAAA GTTTTTAGGT CAAAGGGAAG CTTGTTAAAA TTAGGGACAA ATCACTACGT   
  
  
- ACATTATACA AAACACGAGA TATACCACAT GATTGGGAGA GATAACTATC GCGTACCACC AAGCACCGGG   
  
  
- TCCATACATG GGTAAAAGAG AGATTAGCAA CAGATACGTA CAGAGCTAAG AATACACACG TGCTAGTAAG   
  
  
- TCCTATAAGT AACTAAATGA GTAGTTAACA GTGCCGTCGA AGTAAAATAA TCATCTCTGG GCTGAAATCC   
  
  
- CTGAATCTCC CCACGATGCC AGGAATGGCA TGGAAGGACT ATTCATTGGA CTGGGGGCTT GGGCTAGGCC   
  
  
- AAAAAGTGTC TGGTAGAAAA GGTTTTATTC CTCAGTGTGA ATCCCAAAAA GAAAGAATAA AACAAATGGG   
  
  
- AAATTTTTAT TTTGTTTTTA TTCACCGCTG AGGTTCAGTA AAAAGAATTA GTTATTTCTA GTAAAAAAGC   
  
  
- TTTTGCTTTT AGCTCGAGCG GTAGCTCACC CTTTGCGTAA CTCGGCTTTA CGCCCCAGGT ATCCATACAA   
  
  
- AAAATAAATA CTCTATTTTT CATTTATTAT AAAAAAAGCC CAAATTTTTT ATATAATAAC TAATGAACAT   
  
  
- TAATATACTC GGAGCAAACA TGAATAATAA TTTATGTATA AGTTCCATTT TAATAGGAAA TTTTTTAAGA   
  
  
- ATCCGGCTCC CTTGGAAGGA TAATTATATT CAGTAGGTCG GGTGGAATAC CCCCATTAAG AGGGGTATAT   
  
  
- GGTACCCAGG TTTAGCCGCC GGGCCAGTCA AGTTGCTGTC ATGCTACCGT GAGACTTGGG ACACGACGTA   
  
  
- GACGAGTGTT TTTCATGTGG TGTCAACAGA AAGTGAGAGA GACGGGTAAG TGATACGTTT GTGCAAATAT   
  
  
- TTTAACCAAT AAAATATTTG CAAAGAGAAA GTTTACCAAT AAAATATAAT CATTGTTTTT ATAATATTAT   
  
  
- TATTTTTTTA GTTATAATGC ATTAACGATT TTTACTTTAA TGTAGTACAA CATTCTATAA AGTAAACGGA   
  
  
- TAATTATTAA AAAATAATAA ATATTTTAAC CCAACTTAAA GTTATAAAAA ATTTTATTCA GAATAAATAA   
  
  
- ATTCACTTTA AAAAAAATAT CTATTTCAGT GTTTTTAGTA AAAAGATTTA TTAAATATTT TTCTATGAAA   
  
  
- AGGTTATTTT TAATTTAATT TTACGAAAAT ATTTATTTTA ACATTGTTAA GTAAGGTATC GTTTCCATTT   
  
  
- ATTTCGGTAA AAGAATAGTT AATTTAGTTC GAAGAGAAAA AATACTTTAA CAAAAATTAT TTAAGAAAAT   
  
  
- CATTGTTATT CTAAAACAAG TATAAAATTA TGTAGAGGAA CCGTTAACCC GTTTCTACCG ACCATCACTG   
  
  
- TACTTAGGTT CGTACTTAAT CAGGCCCGCA ACAATGAACG ATGTTCCCAA TACTCGTACC TTCCTTTTTA   
  
  
- TTAATTTTTT ATTTTTTTTA ATTTAATGAA GTTAGTGTTT CAACTTTTAT TATTTTAACA CAAACCTTCA   
  
  
- TAAACATGAA TTTCAAAAAA TCATTTTCAC AAAGGGATTT TCCACAAAAA AAATCTCTTT CGTCGATATT   
  
  
- TTACAAAAAA GTTTTTATAA AAAAATAAAT TTTTGTGAAA GAATTTAGTG AACAATATTC AGTGGAATCT   
  
  
- TACTGACTGC CACAACGTGT ATAGTTTTTC GTAAATACGG GATCACGGTA AAATAATAAA ATATAGAATT   
  
  
- AAAATCTATA AAAATAAAAA TAAATTACTA AATATAAACT TGCAATTAAA AGTTATATAG TATAACATAG   
  
  
- AAAATATCGG TGCAAATAAG TTTATATAAA AAATATAAAT ATTTATAAAA AAGATTTTAT ACTAAGCTCT   
  
  
- TACGACTGTA CACTTAGCGT ACCCCCATCC CGTTCGGTAG AACCTGGGTA ATTTGAAGTT TAAGGTACTA   
  
  
- ACTGATCCTG AACGGGTTTT TAAAGTAATG GGGTCTACGG GTGTACACTT ACGTCAGAGA CTGTCTCATC   
  
  
- CACCCTTATT TGGTTTTACG TAAATAAATA AAAAGTTCTA AAATTAATAA TAAGTGGATT TTAGGAAGTA   
  
  
- TCCCTAATTT TATAATCTGG TAACCTAAAC GAGAGGAGTT ATCCCAACTT GAAACTAATC ATGCAAATAT   
  
  
- AATAAAAAAA GGAAAAAGCA GTTTTACGTA AGACAGTGTT TTAATTACAT CAGTTCCTTA ACTGTTCACT   
  
  
- CTTCTCTCAG TGAAATTTAC TAAGTCGACA GGTGTAATTT CTATTCAGTG TAAACACCAG GTATGAGGGG   
  
  
- TTCTGAAATT AAACTTAAAT CGAGTCTGAA AAGCATAAAA TAAACACAAA AAGATAAATT CGATAGTTAT   
  
  
- GTAAATATTT ATTCACTAAA ATTATATTTT CTAGTTCATT TTTTGTTTAT TTCATGTATT TTATAAAAAT   
  
  
- AAGTTAATGT ATAACATAGT TTAAAAGTTC TCGTTTTAAA TATCGTAAAA AGTTACAAAA CCTTTAGTTT   
  
  
- TTTCATTATA TAATAGTCAA GTGATCAATT TAACCACTAA CTTGGTATCT ACTACAGTAT TATATATAAA   
  
  
- ATATAAATAA TTTTAATTTT TATAAATATT CTTTAAAATT TATATACTCT TTAATTTTCA GTTATTATAA   
  
  
- AAAAAGAAGT AGAAATTATA ATAGTTTACT TATATATATA TATATATTTA CATAACTATA AAATTTTAAA   
  
  
- ACAATTTATT TTATATATAT TATACTTCTA TAAATAACTT TCAATAAATT AAATGGATAT ATATATAATT   
  
  
- TTCAATAAAT ACCAACATTT TTTATAATGT AATAAAATAA ATATAATAAT TATAATGTAA TAAAATAAAT   
  
  
- ATAATAAATA TTAAAGTAAT AATATTTTTC AAACTTTTAT AATAACGATA TATTAAACAA CAACACACTT   
  
  
- CTAGCCGTTT TTTTGTCCTC ATCGTCACAC CACAGCAGGA ACGGGGAACT TGACCTCCAA CCAAAGGGTA   
  
  
- AGGAGGGAGG GTGCTTAGGG TGTACATTTC GTTTATTTTC TCGAAATGTA TGTGTACTAA TTACATAAAA   
  
  
- ATAACAGTTA TTTAAAATAT TATTTTGAAT ATTTAGAATA TTTAAATTAT CCTTTTTTTT TTCAGAAGAT   
  
  
- CTTAACTAAA ATATTATGTG GTATGGTTCT AGTGTACTTA ACGTACACAG CCACCTGTTC CAACGGTTTT   
  
  
- AAGGAATAAG GATATGTCTT AGAAAAGTAA CAATATAATA GTATCAGCTT TGGGTCATCA TATATTTCTC   
  
  
- CAATGCTCAC CTCCTACCTA TACTTTATTT ATTCTCCGGA ATCTCTAGTC CCACACTCTG TACCTAAACC   
  
  
- GTTTACCACT CTCTAGATCA CTCG

+     Unnamed\_\_6

| Site Name | Organism | Position | Strand | Matrix score. | sequence | function |
| --- | --- | --- | --- | --- | --- | --- |
| Unnamed\_\_6 | Zea mays | 2175 | - | 10 | taTAAATATct |  |
| Unnamed\_\_6 | Zea mays | 3029 | - | 10 | taTAAATATct |  |
| Unnamed\_\_6 | Zea mays | 2279 | + | 10 | taTAAATATct |  |
| Unnamed\_\_6 | Zea mays | 3177 | - | 10 | taTAAATATct |  |

>PlantCARE\_32120   
+ AATCATTAAA GTCTAATTCT TCAAAATTCC GAATTCTAAA TTTAGCTATT TAAAATTCCA ATTGTCCTAT   
  
  
+ CTCCGAACTT AATTTTCAGT TTCCCATGCT CCAATTCCCG TATTTACCCC GCTACTTATT ATTATCATTA   
  
  
+ TCATTATCAC CTTATTCATT ATTTCTATAA ATTCCGCCTT CGAATGATTT CTAAGATCTC CAATTTTTAT   
  
  
+ ACATCAACTT TCATAATTTC TACGTCTCAA ATAACATACG ATTCTGATTC CTTTCAAAAT TTAACTCCCA   
  
  
+ TAATTCCAAT TTTCGTAACT TAATTTTTCT TAAAAATCCC GAACTTTCAA AATCCCAATT TTCTTTCAAA   
  
  
+ TTTAATTTCT AAATGTTCTC ATTCTTACAT CTAATTCCTA AGAATCCAAT TTTCAAATAA TCTCTAAGAT   
  
  
+ TCCGATTCTC AAATGAATTT CAAAAATCCA GTTTCCCTTC GAACAATTTT AATCCCTGTT TAGTGATGCA   
  
  
+ TGTAATATGT TTTGTGCTCT ATATGGTGTA CTAACCCTCT CTATTGATAG CGCATGGTGG TTCGTGGCCC   
  
  
+ AGGTATGTAC CCATTTTCTC TCTAATCGTT GTCTATGCAT GTCTCGATTC TTATGTGTGC ACGATCATTC   
  
  
+ AGGATATTCA TTGATTTACT CATCAATTGT CACGGCAGCT TCATTTTATT AGTAGAGACC CGACTTTAGG   
  
  
+ GACTTAGAGG GGTGCTACGG TCCTTACCGT ACCTTCCTGA TAAGTAACCT GACCCCCGAA CCCGATCCGG   
  
  
+ TTTTTCACAG ACCATCTTTT CCAAAATAAG GAGTCACACT TAGGGTTTTT CTTTCTTATT TTGTTTACCC   
  
  
+ TTTAAAAATA AAACAAAAAT AAGTGGCGAC TCCAAGTCAT TTTTCTTAAT CAATAAAGAT CATTTTTTCG   
  
  
+ AAAACGAAAA TCGAGCTCGC CATCGAGTGG GAAACGCATT GAGCCGAAAT GCGGGGTCCA TAGGTATGTT   
  
  
+ TTTTATTTAT GAGATAAAAA GTAAATAATA TTTTTTTCGG GTTTAAAAAA TATATTATTG ATTACTTGTA   
  
  
+ ATTATATGAG CCTCGTTTGT ACTTATTATT AAATACATAT TCAAGGTAAA ATTATCCTTT AAAAAATTCT   
  
  
+ TAGGCCGAGG GAACCTTCCT ATTAATATAA GTCATCCAGC CCACCTTATG GGGGTAATTC TCCCCATATA   
  
  
+ CCATGGGTCC AAATCGGCGG CCCGGTCAGT TCAACGACAG TACGATGGCA CTCTGAACCC TGTGCTGCAT   
  
  
+ CTGCTCACAA AAAGTACACC ACAGTTGTCT TTCACTCTCT CTGCCCATTC ACTATGCAAA CACGTTTATA   
  
  
+ AAATTGGTTA TTTTATAAAC GTTTCTCTTT CAAATGGTTA TTTTATATTA GTAACAAAAA TATTATAATA   
  
  
+ ATAAAAAAAT CAATATTACG TAATTGCTAA AAATGAAATT ACATCATGTT GTAAGATATT TCATTTGCCT   
  
  
+ ATTAATAATT TTTTATTATT TATAAAATTG GGTTGAATTT CAATATTTTT TAAAATAAGT CTTATTTATT   
  
  
+ TAAGTGAAAT TTTTTTTATA GATAAAGTCA CAAAAATCAT TTTTCTAAAT AATTTATAAA AAGATACTTT   
  
  
+ TCCAATAAAA ATTAAATTAA AATGCTTTTA TAAATAAAAT TGTAACAATT CATTCCATAG CAAAGGTAAA   
  
  
+ TAAAGCCATT TTCTTATCAA TTAAATCAAG CTTCTCTTTT TTATGAAATT GTTTTTAATA AATTCTTTTA   
  
  
+ GTAACAATAA GATTTTGTTC ATATTTTAAT ACATCTCCTT GGCAATTGGG CAAAGATGGC TGGTAGTGAC   
  
  
+ ATGAATCCAA GCATGAATTA GTCCGGGCGT TGTTACTTGC TACAAGGGTT ATGAGCATGG AAGGAAAAAT   
  
  
+ AATTAAAAAA TAAAAAAAAT TAAATTACTT CAATCACAAA GTTGAAAATA ATAAAATTGT GTTTGGAAGT   
  
  
+ ATTTGTACTT AAAGTTTTTT AGTAAAAGTG TTTCCCTAAA AGGTGTTTTT TTTAGAGAAA GCAGCTATAA   
  
  
+ AATGTTTTTT CAAAAATATT TTTTTATTTA AAAACACTTT CTTAAATCAC TTGTTATAAG TCACCTTAGA   
  
  
+ ATGACTGACG GTGTTGCACA TATCAAAAAG CATTTATGCC CTAGTGCCAT TTTATTATTT TATATCTTAA   
  
  
+ TTTTAGATAT TTTTATTTTT ATTTAATGAT TTATATTTGA ACGTTAATTT TCAATATATC ATATTGTATC   
  
  
+ TTTTATAGCC ACGTTTATTC AAATATATTT TTTATATTTA TAAATATTTT TTCTAAAATA TGATTCGAGA   
  
  
+ ATGCTGACAT GTGAATCGCA TGGGGGTAGG GCAAGCCATC TTGGACCCAT TAAACTTCAA ATTCCATGAT   
  
  
+ TGACTAGGAC TTGCCCAAAA ATTTCATTAC CCCAGATGCC CACATGTGAA TGCAGTCTCT GACAGAGTAG   
  
  
+ GTGGGAATAA ACCAAAATGC ATTTATTTAT TTTTCAAGAT TTTAATTATT ATTCACCTAA AATCCTTCAT   
  
  
+ AGGGATTAAA ATATTAGACC ATTGGATTTG CTCTCCTCAA TAGGGTTGAA CTTTGATTAG TACGTTTATA   
  
  
+ TTATTTTTTT CCTTTTTCGT CAAAATGCAT TCTGTCACAA AATTAATGTA GTCAAGGAAT TGACAAGTGA   
  
  
+ GAAGAGAGTC ACTTTAAATG ATTCAGCTGT CCACATTAAA GATAAGTCAC ATTTGTGGTC CATACTCCCC   
  
  
+ AAGACTTTAA TTTGAATTTA GCTCAGACTT TTCGTATTTT ATTTGTGTTT TTCTATTTAA GCTATCAATA   
  
  
+ CATTTATAAA TAAGTGATTT TAATATAAAA GATCAAGTAA AAAACAAATA AAGTACATAA AATATTTTTA   
  
  
+ TTCAATTACA TATTGTATCA AATTTTCAAG AGCAAAATTT ATAGCATTTT TCAATGTTTT GGAAATCAAA   
  
  
+ AAAGTAATAT ATTATCAGTT CACTAGTTAA ATTGGTGATT GAACCATAGA TGATGTCATA ATATATATTT   
  
  
+ TATATTTATT AAAATTAAAA ATATTTATAA GAAATTTTAA ATATATGAGA AATTAAAAGT CAATAATATT   
  
  
+ TTTTTCTTCA TCTTTAATAT TATCAAATGA ATATATATAT ATATATAAAT GTATTGATAT TTTAAAATTT   
  
  
+ TGTTAAATAA AATATATATA ATATGAAGAT ATTTATTGAA AGTTATTTAA TTTACCTATA TATATATTAA   
  
  
+ AAGTTATTTA TGGTTGTAAA AAATATTACA TTATTTTATT TATATTATTA ATATTACATT ATTTTATTTA   
  
  
+ TATTATTTAT AATTTCATTA TTATAAAAAG TTTGAAAATA TTATTGCTAT ATAATTTGTT GTTGTGTGAA   
  
  
+ GATCGGCAAA AAAACAGGAG TAGCAGTGTG GTGTCGTCCT TGCCCCTTGA ACTGGAGGTT GGTTTCCCAT   
  
  
+ TCCTCCCTCC CACGAATCCC ACATGTAAAG CAAATAAAAG AGCTTTACAT ACACATGATT AATGTATTTT   
  
  
+ TATTGTCAAT AAATTTTATA ATAAAACTTA TAAATCTTAT AAATTTAATA GGAAAAAAAA AAGTCTTCTA   
  
  
+ GAATTGATTT TATAATACAC CATACCAAGA TCACATGAAT TGCATGTGTC GGTGGACAAG GTTGCCAAAA   
  
  
+ TTCCTTATTC CTATACAGAA TCTTTTCATT GTTATATTAT CATAGTCGAA ACCCAGTAGT ATATAAAGAG   
  
  
+ GTTACGAGTG GAGGATGGAT ATGAAATAAA TAAGAGGCCT TAGAGATCAG GGTGTGAGAC ATGGATTTGG   
  
  
+ CAAATGGTGA GAGATCTAGT GAGC  

- TTAGTAATTT CAGATTAAGA AGTTTTAAGG CTTAAGATTT AAATCGATAA ATTTTAAGGT TAACAGGATA   
  
  
- GAGGCTTGAA TTAAAAGTCA AAGGGTACGA GGTTAAGGGC ATAAATGGGG CGATGAATAA TAATAGTAAT   
  
  
- AGTAATAGTG GAATAAGTAA TAAAGATATT TAAGGCGGAA GCTTACTAAA GATTCTAGAG GTTAAAAATA   
  
  
- TGTAGTTGAA AGTATTAAAG ATGCAGAGTT TATTGTATGC TAAGACTAAG GAAAGTTTTA AATTGAGGGT   
  
  
- ATTAAGGTTA AAAGCATTGA ATTAAAAAGA ATTTTTAGGG CTTGAAAGTT TTAGGGTTAA AAGAAAGTTT   
  
  
- AAATTAAAGA TTTACAAGAG TAAGAATGTA GATTAAGGAT TCTTAGGTTA AAAGTTTATT AGAGATTCTA   
  
  
- AGGCTAAGAG TTTACTTAAA GTTTTTAGGT CAAAGGGAAG CTTGTTAAAA TTAGGGACAA ATCACTACGT   
  
  
- ACATTATACA AAACACGAGA TATACCACAT GATTGGGAGA GATAACTATC GCGTACCACC AAGCACCGGG   
  
  
- TCCATACATG GGTAAAAGAG AGATTAGCAA CAGATACGTA CAGAGCTAAG AATACACACG TGCTAGTAAG   
  
  
- TCCTATAAGT AACTAAATGA GTAGTTAACA GTGCCGTCGA AGTAAAATAA TCATCTCTGG GCTGAAATCC   
  
  
- CTGAATCTCC CCACGATGCC AGGAATGGCA TGGAAGGACT ATTCATTGGA CTGGGGGCTT GGGCTAGGCC   
  
  
- AAAAAGTGTC TGGTAGAAAA GGTTTTATTC CTCAGTGTGA ATCCCAAAAA GAAAGAATAA AACAAATGGG   
  
  
- AAATTTTTAT TTTGTTTTTA TTCACCGCTG AGGTTCAGTA AAAAGAATTA GTTATTTCTA GTAAAAAAGC   
  
  
- TTTTGCTTTT AGCTCGAGCG GTAGCTCACC CTTTGCGTAA CTCGGCTTTA CGCCCCAGGT ATCCATACAA   
  
  
- AAAATAAATA CTCTATTTTT CATTTATTAT AAAAAAAGCC CAAATTTTTT ATATAATAAC TAATGAACAT   
  
  
- TAATATACTC GGAGCAAACA TGAATAATAA TTTATGTATA AGTTCCATTT TAATAGGAAA TTTTTTAAGA   
  
  
- ATCCGGCTCC CTTGGAAGGA TAATTATATT CAGTAGGTCG GGTGGAATAC CCCCATTAAG AGGGGTATAT   
  
  
- GGTACCCAGG TTTAGCCGCC GGGCCAGTCA AGTTGCTGTC ATGCTACCGT GAGACTTGGG ACACGACGTA   
  
  
- GACGAGTGTT TTTCATGTGG TGTCAACAGA AAGTGAGAGA GACGGGTAAG TGATACGTTT GTGCAAATAT   
  
  
- TTTAACCAAT AAAATATTTG CAAAGAGAAA GTTTACCAAT AAAATATAAT CATTGTTTTT ATAATATTAT   
  
  
- TATTTTTTTA GTTATAATGC ATTAACGATT TTTACTTTAA TGTAGTACAA CATTCTATAA AGTAAACGGA   
  
  
- TAATTATTAA AAAATAATAA ATATTTTAAC CCAACTTAAA GTTATAAAAA ATTTTATTCA GAATAAATAA   
  
  
- ATTCACTTTA AAAAAAATAT CTATTTCAGT GTTTTTAGTA AAAAGATTTA TTAAATATTT TTCTATGAAA   
  
  
- AGGTTATTTT TAATTTAATT TTACGAAAAT ATTTATTTTA ACATTGTTAA GTAAGGTATC GTTTCCATTT   
  
  
- ATTTCGGTAA AAGAATAGTT AATTTAGTTC GAAGAGAAAA AATACTTTAA CAAAAATTAT TTAAGAAAAT   
  
  
- CATTGTTATT CTAAAACAAG TATAAAATTA TGTAGAGGAA CCGTTAACCC GTTTCTACCG ACCATCACTG   
  
  
- TACTTAGGTT CGTACTTAAT CAGGCCCGCA ACAATGAACG ATGTTCCCAA TACTCGTACC TTCCTTTTTA   
  
  
- TTAATTTTTT ATTTTTTTTA ATTTAATGAA GTTAGTGTTT CAACTTTTAT TATTTTAACA CAAACCTTCA   
  
  
- TAAACATGAA TTTCAAAAAA TCATTTTCAC AAAGGGATTT TCCACAAAAA AAATCTCTTT CGTCGATATT   
  
  
- TTACAAAAAA GTTTTTATAA AAAAATAAAT TTTTGTGAAA GAATTTAGTG AACAATATTC AGTGGAATCT   
  
  
- TACTGACTGC CACAACGTGT ATAGTTTTTC GTAAATACGG GATCACGGTA AAATAATAAA ATATAGAATT   
  
  
- AAAATCTATA AAAATAAAAA TAAATTACTA AATATAAACT TGCAATTAAA AGTTATATAG TATAACATAG   
  
  
- AAAATATCGG TGCAAATAAG TTTATATAAA AAATATAAAT ATTTATAAAA AAGATTTTAT ACTAAGCTCT   
  
  
- TACGACTGTA CACTTAGCGT ACCCCCATCC CGTTCGGTAG AACCTGGGTA ATTTGAAGTT TAAGGTACTA   
  
  
- ACTGATCCTG AACGGGTTTT TAAAGTAATG GGGTCTACGG GTGTACACTT ACGTCAGAGA CTGTCTCATC   
  
  
- CACCCTTATT TGGTTTTACG TAAATAAATA AAAAGTTCTA AAATTAATAA TAAGTGGATT TTAGGAAGTA   
  
  
- TCCCTAATTT TATAATCTGG TAACCTAAAC GAGAGGAGTT ATCCCAACTT GAAACTAATC ATGCAAATAT   
  
  
- AATAAAAAAA GGAAAAAGCA GTTTTACGTA AGACAGTGTT TTAATTACAT CAGTTCCTTA ACTGTTCACT   
  
  
- CTTCTCTCAG TGAAATTTAC TAAGTCGACA GGTGTAATTT CTATTCAGTG TAAACACCAG GTATGAGGGG   
  
  
- TTCTGAAATT AAACTTAAAT CGAGTCTGAA AAGCATAAAA TAAACACAAA AAGATAAATT CGATAGTTAT   
  
  
- GTAAATATTT ATTCACTAAA ATTATATTTT CTAGTTCATT TTTTGTTTAT TTCATGTATT TTATAAAAAT   
  
  
- AAGTTAATGT ATAACATAGT TTAAAAGTTC TCGTTTTAAA TATCGTAAAA AGTTACAAAA CCTTTAGTTT   
  
  
- TTTCATTATA TAATAGTCAA GTGATCAATT TAACCACTAA CTTGGTATCT ACTACAGTAT TATATATAAA   
  
  
- ATATAAATAA TTTTAATTTT TATAAATATT CTTTAAAATT TATATACTCT TTAATTTTCA GTTATTATAA   
  
  
- AAAAAGAAGT AGAAATTATA ATAGTTTACT TATATATATA TATATATTTA CATAACTATA AAATTTTAAA   
  
  
- ACAATTTATT TTATATATAT TATACTTCTA TAAATAACTT TCAATAAATT AAATGGATAT ATATATAATT   
  
  
- TTCAATAAAT ACCAACATTT TTTATAATGT AATAAAATAA ATATAATAAT TATAATGTAA TAAAATAAAT   
  
  
- ATAATAAATA TTAAAGTAAT AATATTTTTC AAACTTTTAT AATAACGATA TATTAAACAA CAACACACTT   
  
  
- CTAGCCGTTT TTTTGTCCTC ATCGTCACAC CACAGCAGGA ACGGGGAACT TGACCTCCAA CCAAAGGGTA   
  
  
- AGGAGGGAGG GTGCTTAGGG TGTACATTTC GTTTATTTTC TCGAAATGTA TGTGTACTAA TTACATAAAA   
  
  
- ATAACAGTTA TTTAAAATAT TATTTTGAAT ATTTAGAATA TTTAAATTAT CCTTTTTTTT TTCAGAAGAT   
  
  
- CTTAACTAAA ATATTATGTG GTATGGTTCT AGTGTACTTA ACGTACACAG CCACCTGTTC CAACGGTTTT   
  
  
- AAGGAATAAG GATATGTCTT AGAAAAGTAA CAATATAATA GTATCAGCTT TGGGTCATCA TATATTTCTC   
  
  
- CAATGCTCAC CTCCTACCTA TACTTTATTT ATTCTCCGGA ATCTCTAGTC CCACACTCTG TACCTAAACC   
  
  
- GTTTACCACT CTCTAGATCA CTCG

+     WRE3

| Site Name | Organism | Position | Strand | Matrix score. | sequence | function |
| --- | --- | --- | --- | --- | --- | --- |
| WRE3 | Pisum sativum | 1161 | + | 6 | CCACCT |  |
| WRE3 | Pisum sativum | 2449 | - | 6 | CCACCT |  |

>PlantCARE\_32120   
+ AATCATTAAA GTCTAATTCT TCAAAATTCC GAATTCTAAA TTTAGCTATT TAAAATTCCA ATTGTCCTAT   
  
  
+ CTCCGAACTT AATTTTCAGT TTCCCATGCT CCAATTCCCG TATTTACCCC GCTACTTATT ATTATCATTA   
  
  
+ TCATTATCAC CTTATTCATT ATTTCTATAA ATTCCGCCTT CGAATGATTT CTAAGATCTC CAATTTTTAT   
  
  
+ ACATCAACTT TCATAATTTC TACGTCTCAA ATAACATACG ATTCTGATTC CTTTCAAAAT TTAACTCCCA   
  
  
+ TAATTCCAAT TTTCGTAACT TAATTTTTCT TAAAAATCCC GAACTTTCAA AATCCCAATT TTCTTTCAAA   
  
  
+ TTTAATTTCT AAATGTTCTC ATTCTTACAT CTAATTCCTA AGAATCCAAT TTTCAAATAA TCTCTAAGAT   
  
  
+ TCCGATTCTC AAATGAATTT CAAAAATCCA GTTTCCCTTC GAACAATTTT AATCCCTGTT TAGTGATGCA   
  
  
+ TGTAATATGT TTTGTGCTCT ATATGGTGTA CTAACCCTCT CTATTGATAG CGCATGGTGG TTCGTGGCCC   
  
  
+ AGGTATGTAC CCATTTTCTC TCTAATCGTT GTCTATGCAT GTCTCGATTC TTATGTGTGC ACGATCATTC   
  
  
+ AGGATATTCA TTGATTTACT CATCAATTGT CACGGCAGCT TCATTTTATT AGTAGAGACC CGACTTTAGG   
  
  
+ GACTTAGAGG GGTGCTACGG TCCTTACCGT ACCTTCCTGA TAAGTAACCT GACCCCCGAA CCCGATCCGG   
  
  
+ TTTTTCACAG ACCATCTTTT CCAAAATAAG GAGTCACACT TAGGGTTTTT CTTTCTTATT TTGTTTACCC   
  
  
+ TTTAAAAATA AAACAAAAAT AAGTGGCGAC TCCAAGTCAT TTTTCTTAAT CAATAAAGAT CATTTTTTCG   
  
  
+ AAAACGAAAA TCGAGCTCGC CATCGAGTGG GAAACGCATT GAGCCGAAAT GCGGGGTCCA TAGGTATGTT   
  
  
+ TTTTATTTAT GAGATAAAAA GTAAATAATA TTTTTTTCGG GTTTAAAAAA TATATTATTG ATTACTTGTA   
  
  
+ ATTATATGAG CCTCGTTTGT ACTTATTATT AAATACATAT TCAAGGTAAA ATTATCCTTT AAAAAATTCT   
  
  
+ TAGGCCGAGG GAACCTTCCT ATTAATATAA GTCATCCAGC CCACCTTATG GGGGTAATTC TCCCCATATA   
  
  
+ CCATGGGTCC AAATCGGCGG CCCGGTCAGT TCAACGACAG TACGATGGCA CTCTGAACCC TGTGCTGCAT   
  
  
+ CTGCTCACAA AAAGTACACC ACAGTTGTCT TTCACTCTCT CTGCCCATTC ACTATGCAAA CACGTTTATA   
  
  
+ AAATTGGTTA TTTTATAAAC GTTTCTCTTT CAAATGGTTA TTTTATATTA GTAACAAAAA TATTATAATA   
  
  
+ ATAAAAAAAT CAATATTACG TAATTGCTAA AAATGAAATT ACATCATGTT GTAAGATATT TCATTTGCCT   
  
  
+ ATTAATAATT TTTTATTATT TATAAAATTG GGTTGAATTT CAATATTTTT TAAAATAAGT CTTATTTATT   
  
  
+ TAAGTGAAAT TTTTTTTATA GATAAAGTCA CAAAAATCAT TTTTCTAAAT AATTTATAAA AAGATACTTT   
  
  
+ TCCAATAAAA ATTAAATTAA AATGCTTTTA TAAATAAAAT TGTAACAATT CATTCCATAG CAAAGGTAAA   
  
  
+ TAAAGCCATT TTCTTATCAA TTAAATCAAG CTTCTCTTTT TTATGAAATT GTTTTTAATA AATTCTTTTA   
  
  
+ GTAACAATAA GATTTTGTTC ATATTTTAAT ACATCTCCTT GGCAATTGGG CAAAGATGGC TGGTAGTGAC   
  
  
+ ATGAATCCAA GCATGAATTA GTCCGGGCGT TGTTACTTGC TACAAGGGTT ATGAGCATGG AAGGAAAAAT   
  
  
+ AATTAAAAAA TAAAAAAAAT TAAATTACTT CAATCACAAA GTTGAAAATA ATAAAATTGT GTTTGGAAGT   
  
  
+ ATTTGTACTT AAAGTTTTTT AGTAAAAGTG TTTCCCTAAA AGGTGTTTTT TTTAGAGAAA GCAGCTATAA   
  
  
+ AATGTTTTTT CAAAAATATT TTTTTATTTA AAAACACTTT CTTAAATCAC TTGTTATAAG TCACCTTAGA   
  
  
+ ATGACTGACG GTGTTGCACA TATCAAAAAG CATTTATGCC CTAGTGCCAT TTTATTATTT TATATCTTAA   
  
  
+ TTTTAGATAT TTTTATTTTT ATTTAATGAT TTATATTTGA ACGTTAATTT TCAATATATC ATATTGTATC   
  
  
+ TTTTATAGCC ACGTTTATTC AAATATATTT TTTATATTTA TAAATATTTT TTCTAAAATA TGATTCGAGA   
  
  
+ ATGCTGACAT GTGAATCGCA TGGGGGTAGG GCAAGCCATC TTGGACCCAT TAAACTTCAA ATTCCATGAT   
  
  
+ TGACTAGGAC TTGCCCAAAA ATTTCATTAC CCCAGATGCC CACATGTGAA TGCAGTCTCT GACAGAGTAG   
  
  
+ GTGGGAATAA ACCAAAATGC ATTTATTTAT TTTTCAAGAT TTTAATTATT ATTCACCTAA AATCCTTCAT   
  
  
+ AGGGATTAAA ATATTAGACC ATTGGATTTG CTCTCCTCAA TAGGGTTGAA CTTTGATTAG TACGTTTATA   
  
  
+ TTATTTTTTT CCTTTTTCGT CAAAATGCAT TCTGTCACAA AATTAATGTA GTCAAGGAAT TGACAAGTGA   
  
  
+ GAAGAGAGTC ACTTTAAATG ATTCAGCTGT CCACATTAAA GATAAGTCAC ATTTGTGGTC CATACTCCCC   
  
  
+ AAGACTTTAA TTTGAATTTA GCTCAGACTT TTCGTATTTT ATTTGTGTTT TTCTATTTAA GCTATCAATA   
  
  
+ CATTTATAAA TAAGTGATTT TAATATAAAA GATCAAGTAA AAAACAAATA AAGTACATAA AATATTTTTA   
  
  
+ TTCAATTACA TATTGTATCA AATTTTCAAG AGCAAAATTT ATAGCATTTT TCAATGTTTT GGAAATCAAA   
  
  
+ AAAGTAATAT ATTATCAGTT CACTAGTTAA ATTGGTGATT GAACCATAGA TGATGTCATA ATATATATTT   
  
  
+ TATATTTATT AAAATTAAAA ATATTTATAA GAAATTTTAA ATATATGAGA AATTAAAAGT CAATAATATT   
  
  
+ TTTTTCTTCA TCTTTAATAT TATCAAATGA ATATATATAT ATATATAAAT GTATTGATAT TTTAAAATTT   
  
  
+ TGTTAAATAA AATATATATA ATATGAAGAT ATTTATTGAA AGTTATTTAA TTTACCTATA TATATATTAA   
  
  
+ AAGTTATTTA TGGTTGTAAA AAATATTACA TTATTTTATT TATATTATTA ATATTACATT ATTTTATTTA   
  
  
+ TATTATTTAT AATTTCATTA TTATAAAAAG TTTGAAAATA TTATTGCTAT ATAATTTGTT GTTGTGTGAA   
  
  
+ GATCGGCAAA AAAACAGGAG TAGCAGTGTG GTGTCGTCCT TGCCCCTTGA ACTGGAGGTT GGTTTCCCAT   
  
  
+ TCCTCCCTCC CACGAATCCC ACATGTAAAG CAAATAAAAG AGCTTTACAT ACACATGATT AATGTATTTT   
  
  
+ TATTGTCAAT AAATTTTATA ATAAAACTTA TAAATCTTAT AAATTTAATA GGAAAAAAAA AAGTCTTCTA   
  
  
+ GAATTGATTT TATAATACAC CATACCAAGA TCACATGAAT TGCATGTGTC GGTGGACAAG GTTGCCAAAA   
  
  
+ TTCCTTATTC CTATACAGAA TCTTTTCATT GTTATATTAT CATAGTCGAA ACCCAGTAGT ATATAAAGAG   
  
  
+ GTTACGAGTG GAGGATGGAT ATGAAATAAA TAAGAGGCCT TAGAGATCAG GGTGTGAGAC ATGGATTTGG   
  
  
+ CAAATGGTGA GAGATCTAGT GAGC  

- TTAGTAATTT CAGATTAAGA AGTTTTAAGG CTTAAGATTT AAATCGATAA ATTTTAAGGT TAACAGGATA   
  
  
- GAGGCTTGAA TTAAAAGTCA AAGGGTACGA GGTTAAGGGC ATAAATGGGG CGATGAATAA TAATAGTAAT   
  
  
- AGTAATAGTG GAATAAGTAA TAAAGATATT TAAGGCGGAA GCTTACTAAA GATTCTAGAG GTTAAAAATA   
  
  
- TGTAGTTGAA AGTATTAAAG ATGCAGAGTT TATTGTATGC TAAGACTAAG GAAAGTTTTA AATTGAGGGT   
  
  
- ATTAAGGTTA AAAGCATTGA ATTAAAAAGA ATTTTTAGGG CTTGAAAGTT TTAGGGTTAA AAGAAAGTTT   
  
  
- AAATTAAAGA TTTACAAGAG TAAGAATGTA GATTAAGGAT TCTTAGGTTA AAAGTTTATT AGAGATTCTA   
  
  
- AGGCTAAGAG TTTACTTAAA GTTTTTAGGT CAAAGGGAAG CTTGTTAAAA TTAGGGACAA ATCACTACGT   
  
  
- ACATTATACA AAACACGAGA TATACCACAT GATTGGGAGA GATAACTATC GCGTACCACC AAGCACCGGG   
  
  
- TCCATACATG GGTAAAAGAG AGATTAGCAA CAGATACGTA CAGAGCTAAG AATACACACG TGCTAGTAAG   
  
  
- TCCTATAAGT AACTAAATGA GTAGTTAACA GTGCCGTCGA AGTAAAATAA TCATCTCTGG GCTGAAATCC   
  
  
- CTGAATCTCC CCACGATGCC AGGAATGGCA TGGAAGGACT ATTCATTGGA CTGGGGGCTT GGGCTAGGCC   
  
  
- AAAAAGTGTC TGGTAGAAAA GGTTTTATTC CTCAGTGTGA ATCCCAAAAA GAAAGAATAA AACAAATGGG   
  
  
- AAATTTTTAT TTTGTTTTTA TTCACCGCTG AGGTTCAGTA AAAAGAATTA GTTATTTCTA GTAAAAAAGC   
  
  
- TTTTGCTTTT AGCTCGAGCG GTAGCTCACC CTTTGCGTAA CTCGGCTTTA CGCCCCAGGT ATCCATACAA   
  
  
- AAAATAAATA CTCTATTTTT CATTTATTAT AAAAAAAGCC CAAATTTTTT ATATAATAAC TAATGAACAT   
  
  
- TAATATACTC GGAGCAAACA TGAATAATAA TTTATGTATA AGTTCCATTT TAATAGGAAA TTTTTTAAGA   
  
  
- ATCCGGCTCC CTTGGAAGGA TAATTATATT CAGTAGGTCG GGTGGAATAC CCCCATTAAG AGGGGTATAT   
  
  
- GGTACCCAGG TTTAGCCGCC GGGCCAGTCA AGTTGCTGTC ATGCTACCGT GAGACTTGGG ACACGACGTA   
  
  
- GACGAGTGTT TTTCATGTGG TGTCAACAGA AAGTGAGAGA GACGGGTAAG TGATACGTTT GTGCAAATAT   
  
  
- TTTAACCAAT AAAATATTTG CAAAGAGAAA GTTTACCAAT AAAATATAAT CATTGTTTTT ATAATATTAT   
  
  
- TATTTTTTTA GTTATAATGC ATTAACGATT TTTACTTTAA TGTAGTACAA CATTCTATAA AGTAAACGGA   
  
  
- TAATTATTAA AAAATAATAA ATATTTTAAC CCAACTTAAA GTTATAAAAA ATTTTATTCA GAATAAATAA   
  
  
- ATTCACTTTA AAAAAAATAT CTATTTCAGT GTTTTTAGTA AAAAGATTTA TTAAATATTT TTCTATGAAA   
  
  
- AGGTTATTTT TAATTTAATT TTACGAAAAT ATTTATTTTA ACATTGTTAA GTAAGGTATC GTTTCCATTT   
  
  
- ATTTCGGTAA AAGAATAGTT AATTTAGTTC GAAGAGAAAA AATACTTTAA CAAAAATTAT TTAAGAAAAT   
  
  
- CATTGTTATT CTAAAACAAG TATAAAATTA TGTAGAGGAA CCGTTAACCC GTTTCTACCG ACCATCACTG   
  
  
- TACTTAGGTT CGTACTTAAT CAGGCCCGCA ACAATGAACG ATGTTCCCAA TACTCGTACC TTCCTTTTTA   
  
  
- TTAATTTTTT ATTTTTTTTA ATTTAATGAA GTTAGTGTTT CAACTTTTAT TATTTTAACA CAAACCTTCA   
  
  
- TAAACATGAA TTTCAAAAAA TCATTTTCAC AAAGGGATTT TCCACAAAAA AAATCTCTTT CGTCGATATT   
  
  
- TTACAAAAAA GTTTTTATAA AAAAATAAAT TTTTGTGAAA GAATTTAGTG AACAATATTC AGTGGAATCT   
  
  
- TACTGACTGC CACAACGTGT ATAGTTTTTC GTAAATACGG GATCACGGTA AAATAATAAA ATATAGAATT   
  
  
- AAAATCTATA AAAATAAAAA TAAATTACTA AATATAAACT TGCAATTAAA AGTTATATAG TATAACATAG   
  
  
- AAAATATCGG TGCAAATAAG TTTATATAAA AAATATAAAT ATTTATAAAA AAGATTTTAT ACTAAGCTCT   
  
  
- TACGACTGTA CACTTAGCGT ACCCCCATCC CGTTCGGTAG AACCTGGGTA ATTTGAAGTT TAAGGTACTA   
  
  
- ACTGATCCTG AACGGGTTTT TAAAGTAATG GGGTCTACGG GTGTACACTT ACGTCAGAGA CTGTCTCATC   
  
  
- CACCCTTATT TGGTTTTACG TAAATAAATA AAAAGTTCTA AAATTAATAA TAAGTGGATT TTAGGAAGTA   
  
  
- TCCCTAATTT TATAATCTGG TAACCTAAAC GAGAGGAGTT ATCCCAACTT GAAACTAATC ATGCAAATAT   
  
  
- AATAAAAAAA GGAAAAAGCA GTTTTACGTA AGACAGTGTT TTAATTACAT CAGTTCCTTA ACTGTTCACT   
  
  
- CTTCTCTCAG TGAAATTTAC TAAGTCGACA GGTGTAATTT CTATTCAGTG TAAACACCAG GTATGAGGGG   
  
  
- TTCTGAAATT AAACTTAAAT CGAGTCTGAA AAGCATAAAA TAAACACAAA AAGATAAATT CGATAGTTAT   
  
  
- GTAAATATTT ATTCACTAAA ATTATATTTT CTAGTTCATT TTTTGTTTAT TTCATGTATT TTATAAAAAT   
  
  
- AAGTTAATGT ATAACATAGT TTAAAAGTTC TCGTTTTAAA TATCGTAAAA AGTTACAAAA CCTTTAGTTT   
  
  
- TTTCATTATA TAATAGTCAA GTGATCAATT TAACCACTAA CTTGGTATCT ACTACAGTAT TATATATAAA   
  
  
- ATATAAATAA TTTTAATTTT TATAAATATT CTTTAAAATT TATATACTCT TTAATTTTCA GTTATTATAA   
  
  
- AAAAAGAAGT AGAAATTATA ATAGTTTACT TATATATATA TATATATTTA CATAACTATA AAATTTTAAA   
  
  
- ACAATTTATT TTATATATAT TATACTTCTA TAAATAACTT TCAATAAATT AAATGGATAT ATATATAATT   
  
  
- TTCAATAAAT ACCAACATTT TTTATAATGT AATAAAATAA ATATAATAAT TATAATGTAA TAAAATAAAT   
  
  
- ATAATAAATA TTAAAGTAAT AATATTTTTC AAACTTTTAT AATAACGATA TATTAAACAA CAACACACTT   
  
  
- CTAGCCGTTT TTTTGTCCTC ATCGTCACAC CACAGCAGGA ACGGGGAACT TGACCTCCAA CCAAAGGGTA   
  
  
- AGGAGGGAGG GTGCTTAGGG TGTACATTTC GTTTATTTTC TCGAAATGTA TGTGTACTAA TTACATAAAA   
  
  
- ATAACAGTTA TTTAAAATAT TATTTTGAAT ATTTAGAATA TTTAAATTAT CCTTTTTTTT TTCAGAAGAT   
  
  
- CTTAACTAAA ATATTATGTG GTATGGTTCT AGTGTACTTA ACGTACACAG CCACCTGTTC CAACGGTTTT   
  
  
- AAGGAATAAG GATATGTCTT AGAAAAGTAA CAATATAATA GTATCAGCTT TGGGTCATCA TATATTTCTC   
  
  
- CAATGCTCAC CTCCTACCTA TACTTTATTT ATTCTCCGGA ATCTCTAGTC CCACACTCTG TACCTAAACC   
  
  
- GTTTACCACT CTCTAGATCA CTCG

+     WUN-motif

| Site Name | Organism | Position | Strand | Matrix score. | sequence | function |
| --- | --- | --- | --- | --- | --- | --- |
| WUN-motif | Nicotiana glutinosa | 3039 | - | 9 | AAATTTCTT |  |
| WUN-motif | Nicotiana glutinosa | 1912 | + | 8 | AAATTACT |  |
| WUN-motif | Nicotiana glutinosa | 2873 | + | 9 | CAATTACAT |  |

>PlantCARE\_32120   
+ AATCATTAAA GTCTAATTCT TCAAAATTCC GAATTCTAAA TTTAGCTATT TAAAATTCCA ATTGTCCTAT   
  
  
+ CTCCGAACTT AATTTTCAGT TTCCCATGCT CCAATTCCCG TATTTACCCC GCTACTTATT ATTATCATTA   
  
  
+ TCATTATCAC CTTATTCATT ATTTCTATAA ATTCCGCCTT CGAATGATTT CTAAGATCTC CAATTTTTAT   
  
  
+ ACATCAACTT TCATAATTTC TACGTCTCAA ATAACATACG ATTCTGATTC CTTTCAAAAT TTAACTCCCA   
  
  
+ TAATTCCAAT TTTCGTAACT TAATTTTTCT TAAAAATCCC GAACTTTCAA AATCCCAATT TTCTTTCAAA   
  
  
+ TTTAATTTCT AAATGTTCTC ATTCTTACAT CTAATTCCTA AGAATCCAAT TTTCAAATAA TCTCTAAGAT   
  
  
+ TCCGATTCTC AAATGAATTT CAAAAATCCA GTTTCCCTTC GAACAATTTT AATCCCTGTT TAGTGATGCA   
  
  
+ TGTAATATGT TTTGTGCTCT ATATGGTGTA CTAACCCTCT CTATTGATAG CGCATGGTGG TTCGTGGCCC   
  
  
+ AGGTATGTAC CCATTTTCTC TCTAATCGTT GTCTATGCAT GTCTCGATTC TTATGTGTGC ACGATCATTC   
  
  
+ AGGATATTCA TTGATTTACT CATCAATTGT CACGGCAGCT TCATTTTATT AGTAGAGACC CGACTTTAGG   
  
  
+ GACTTAGAGG GGTGCTACGG TCCTTACCGT ACCTTCCTGA TAAGTAACCT GACCCCCGAA CCCGATCCGG   
  
  
+ TTTTTCACAG ACCATCTTTT CCAAAATAAG GAGTCACACT TAGGGTTTTT CTTTCTTATT TTGTTTACCC   
  
  
+ TTTAAAAATA AAACAAAAAT AAGTGGCGAC TCCAAGTCAT TTTTCTTAAT CAATAAAGAT CATTTTTTCG   
  
  
+ AAAACGAAAA TCGAGCTCGC CATCGAGTGG GAAACGCATT GAGCCGAAAT GCGGGGTCCA TAGGTATGTT   
  
  
+ TTTTATTTAT GAGATAAAAA GTAAATAATA TTTTTTTCGG GTTTAAAAAA TATATTATTG ATTACTTGTA   
  
  
+ ATTATATGAG CCTCGTTTGT ACTTATTATT AAATACATAT TCAAGGTAAA ATTATCCTTT AAAAAATTCT   
  
  
+ TAGGCCGAGG GAACCTTCCT ATTAATATAA GTCATCCAGC CCACCTTATG GGGGTAATTC TCCCCATATA   
  
  
+ CCATGGGTCC AAATCGGCGG CCCGGTCAGT TCAACGACAG TACGATGGCA CTCTGAACCC TGTGCTGCAT   
  
  
+ CTGCTCACAA AAAGTACACC ACAGTTGTCT TTCACTCTCT CTGCCCATTC ACTATGCAAA CACGTTTATA   
  
  
+ AAATTGGTTA TTTTATAAAC GTTTCTCTTT CAAATGGTTA TTTTATATTA GTAACAAAAA TATTATAATA   
  
  
+ ATAAAAAAAT CAATATTACG TAATTGCTAA AAATGAAATT ACATCATGTT GTAAGATATT TCATTTGCCT   
  
  
+ ATTAATAATT TTTTATTATT TATAAAATTG GGTTGAATTT CAATATTTTT TAAAATAAGT CTTATTTATT   
  
  
+ TAAGTGAAAT TTTTTTTATA GATAAAGTCA CAAAAATCAT TTTTCTAAAT AATTTATAAA AAGATACTTT   
  
  
+ TCCAATAAAA ATTAAATTAA AATGCTTTTA TAAATAAAAT TGTAACAATT CATTCCATAG CAAAGGTAAA   
  
  
+ TAAAGCCATT TTCTTATCAA TTAAATCAAG CTTCTCTTTT TTATGAAATT GTTTTTAATA AATTCTTTTA   
  
  
+ GTAACAATAA GATTTTGTTC ATATTTTAAT ACATCTCCTT GGCAATTGGG CAAAGATGGC TGGTAGTGAC   
  
  
+ ATGAATCCAA GCATGAATTA GTCCGGGCGT TGTTACTTGC TACAAGGGTT ATGAGCATGG AAGGAAAAAT   
  
  
+ AATTAAAAAA TAAAAAAAAT TAAATTACTT CAATCACAAA GTTGAAAATA ATAAAATTGT GTTTGGAAGT   
  
  
+ ATTTGTACTT AAAGTTTTTT AGTAAAAGTG TTTCCCTAAA AGGTGTTTTT TTTAGAGAAA GCAGCTATAA   
  
  
+ AATGTTTTTT CAAAAATATT TTTTTATTTA AAAACACTTT CTTAAATCAC TTGTTATAAG TCACCTTAGA   
  
  
+ ATGACTGACG GTGTTGCACA TATCAAAAAG CATTTATGCC CTAGTGCCAT TTTATTATTT TATATCTTAA   
  
  
+ TTTTAGATAT TTTTATTTTT ATTTAATGAT TTATATTTGA ACGTTAATTT TCAATATATC ATATTGTATC   
  
  
+ TTTTATAGCC ACGTTTATTC AAATATATTT TTTATATTTA TAAATATTTT TTCTAAAATA TGATTCGAGA   
  
  
+ ATGCTGACAT GTGAATCGCA TGGGGGTAGG GCAAGCCATC TTGGACCCAT TAAACTTCAA ATTCCATGAT   
  
  
+ TGACTAGGAC TTGCCCAAAA ATTTCATTAC CCCAGATGCC CACATGTGAA TGCAGTCTCT GACAGAGTAG   
  
  
+ GTGGGAATAA ACCAAAATGC ATTTATTTAT TTTTCAAGAT TTTAATTATT ATTCACCTAA AATCCTTCAT   
  
  
+ AGGGATTAAA ATATTAGACC ATTGGATTTG CTCTCCTCAA TAGGGTTGAA CTTTGATTAG TACGTTTATA   
  
  
+ TTATTTTTTT CCTTTTTCGT CAAAATGCAT TCTGTCACAA AATTAATGTA GTCAAGGAAT TGACAAGTGA   
  
  
+ GAAGAGAGTC ACTTTAAATG ATTCAGCTGT CCACATTAAA GATAAGTCAC ATTTGTGGTC CATACTCCCC   
  
  
+ AAGACTTTAA TTTGAATTTA GCTCAGACTT TTCGTATTTT ATTTGTGTTT TTCTATTTAA GCTATCAATA   
  
  
+ CATTTATAAA TAAGTGATTT TAATATAAAA GATCAAGTAA AAAACAAATA AAGTACATAA AATATTTTTA   
  
  
+ TTCAATTACA TATTGTATCA AATTTTCAAG AGCAAAATTT ATAGCATTTT TCAATGTTTT GGAAATCAAA   
  
  
+ AAAGTAATAT ATTATCAGTT CACTAGTTAA ATTGGTGATT GAACCATAGA TGATGTCATA ATATATATTT   
  
  
+ TATATTTATT AAAATTAAAA ATATTTATAA GAAATTTTAA ATATATGAGA AATTAAAAGT CAATAATATT   
  
  
+ TTTTTCTTCA TCTTTAATAT TATCAAATGA ATATATATAT ATATATAAAT GTATTGATAT TTTAAAATTT   
  
  
+ TGTTAAATAA AATATATATA ATATGAAGAT ATTTATTGAA AGTTATTTAA TTTACCTATA TATATATTAA   
  
  
+ AAGTTATTTA TGGTTGTAAA AAATATTACA TTATTTTATT TATATTATTA ATATTACATT ATTTTATTTA   
  
  
+ TATTATTTAT AATTTCATTA TTATAAAAAG TTTGAAAATA TTATTGCTAT ATAATTTGTT GTTGTGTGAA   
  
  
+ GATCGGCAAA AAAACAGGAG TAGCAGTGTG GTGTCGTCCT TGCCCCTTGA ACTGGAGGTT GGTTTCCCAT   
  
  
+ TCCTCCCTCC CACGAATCCC ACATGTAAAG CAAATAAAAG AGCTTTACAT ACACATGATT AATGTATTTT   
  
  
+ TATTGTCAAT AAATTTTATA ATAAAACTTA TAAATCTTAT AAATTTAATA GGAAAAAAAA AAGTCTTCTA   
  
  
+ GAATTGATTT TATAATACAC CATACCAAGA TCACATGAAT TGCATGTGTC GGTGGACAAG GTTGCCAAAA   
  
  
+ TTCCTTATTC CTATACAGAA TCTTTTCATT GTTATATTAT CATAGTCGAA ACCCAGTAGT ATATAAAGAG   
  
  
+ GTTACGAGTG GAGGATGGAT ATGAAATAAA TAAGAGGCCT TAGAGATCAG GGTGTGAGAC ATGGATTTGG   
  
  
+ CAAATGGTGA GAGATCTAGT GAGC  

- TTAGTAATTT CAGATTAAGA AGTTTTAAGG CTTAAGATTT AAATCGATAA ATTTTAAGGT TAACAGGATA   
  
  
- GAGGCTTGAA TTAAAAGTCA AAGGGTACGA GGTTAAGGGC ATAAATGGGG CGATGAATAA TAATAGTAAT   
  
  
- AGTAATAGTG GAATAAGTAA TAAAGATATT TAAGGCGGAA GCTTACTAAA GATTCTAGAG GTTAAAAATA   
  
  
- TGTAGTTGAA AGTATTAAAG ATGCAGAGTT TATTGTATGC TAAGACTAAG GAAAGTTTTA AATTGAGGGT   
  
  
- ATTAAGGTTA AAAGCATTGA ATTAAAAAGA ATTTTTAGGG CTTGAAAGTT TTAGGGTTAA AAGAAAGTTT   
  
  
- AAATTAAAGA TTTACAAGAG TAAGAATGTA GATTAAGGAT TCTTAGGTTA AAAGTTTATT AGAGATTCTA   
  
  
- AGGCTAAGAG TTTACTTAAA GTTTTTAGGT CAAAGGGAAG CTTGTTAAAA TTAGGGACAA ATCACTACGT   
  
  
- ACATTATACA AAACACGAGA TATACCACAT GATTGGGAGA GATAACTATC GCGTACCACC AAGCACCGGG   
  
  
- TCCATACATG GGTAAAAGAG AGATTAGCAA CAGATACGTA CAGAGCTAAG AATACACACG TGCTAGTAAG   
  
  
- TCCTATAAGT AACTAAATGA GTAGTTAACA GTGCCGTCGA AGTAAAATAA TCATCTCTGG GCTGAAATCC   
  
  
- CTGAATCTCC CCACGATGCC AGGAATGGCA TGGAAGGACT ATTCATTGGA CTGGGGGCTT GGGCTAGGCC   
  
  
- AAAAAGTGTC TGGTAGAAAA GGTTTTATTC CTCAGTGTGA ATCCCAAAAA GAAAGAATAA AACAAATGGG   
  
  
- AAATTTTTAT TTTGTTTTTA TTCACCGCTG AGGTTCAGTA AAAAGAATTA GTTATTTCTA GTAAAAAAGC   
  
  
- TTTTGCTTTT AGCTCGAGCG GTAGCTCACC CTTTGCGTAA CTCGGCTTTA CGCCCCAGGT ATCCATACAA   
  
  
- AAAATAAATA CTCTATTTTT CATTTATTAT AAAAAAAGCC CAAATTTTTT ATATAATAAC TAATGAACAT   
  
  
- TAATATACTC GGAGCAAACA TGAATAATAA TTTATGTATA AGTTCCATTT TAATAGGAAA TTTTTTAAGA   
  
  
- ATCCGGCTCC CTTGGAAGGA TAATTATATT CAGTAGGTCG GGTGGAATAC CCCCATTAAG AGGGGTATAT   
  
  
- GGTACCCAGG TTTAGCCGCC GGGCCAGTCA AGTTGCTGTC ATGCTACCGT GAGACTTGGG ACACGACGTA   
  
  
- GACGAGTGTT TTTCATGTGG TGTCAACAGA AAGTGAGAGA GACGGGTAAG TGATACGTTT GTGCAAATAT   
  
  
- TTTAACCAAT AAAATATTTG CAAAGAGAAA GTTTACCAAT AAAATATAAT CATTGTTTTT ATAATATTAT   
  
  
- TATTTTTTTA GTTATAATGC ATTAACGATT TTTACTTTAA TGTAGTACAA CATTCTATAA AGTAAACGGA   
  
  
- TAATTATTAA AAAATAATAA ATATTTTAAC CCAACTTAAA GTTATAAAAA ATTTTATTCA GAATAAATAA   
  
  
- ATTCACTTTA AAAAAAATAT CTATTTCAGT GTTTTTAGTA AAAAGATTTA TTAAATATTT TTCTATGAAA   
  
  
- AGGTTATTTT TAATTTAATT TTACGAAAAT ATTTATTTTA ACATTGTTAA GTAAGGTATC GTTTCCATTT   
  
  
- ATTTCGGTAA AAGAATAGTT AATTTAGTTC GAAGAGAAAA AATACTTTAA CAAAAATTAT TTAAGAAAAT   
  
  
- CATTGTTATT CTAAAACAAG TATAAAATTA TGTAGAGGAA CCGTTAACCC GTTTCTACCG ACCATCACTG   
  
  
- TACTTAGGTT CGTACTTAAT CAGGCCCGCA ACAATGAACG ATGTTCCCAA TACTCGTACC TTCCTTTTTA   
  
  
- TTAATTTTTT ATTTTTTTTA ATTTAATGAA GTTAGTGTTT CAACTTTTAT TATTTTAACA CAAACCTTCA   
  
  
- TAAACATGAA TTTCAAAAAA TCATTTTCAC AAAGGGATTT TCCACAAAAA AAATCTCTTT CGTCGATATT   
  
  
- TTACAAAAAA GTTTTTATAA AAAAATAAAT TTTTGTGAAA GAATTTAGTG AACAATATTC AGTGGAATCT   
  
  
- TACTGACTGC CACAACGTGT ATAGTTTTTC GTAAATACGG GATCACGGTA AAATAATAAA ATATAGAATT   
  
  
- AAAATCTATA AAAATAAAAA TAAATTACTA AATATAAACT TGCAATTAAA AGTTATATAG TATAACATAG   
  
  
- AAAATATCGG TGCAAATAAG TTTATATAAA AAATATAAAT ATTTATAAAA AAGATTTTAT ACTAAGCTCT   
  
  
- TACGACTGTA CACTTAGCGT ACCCCCATCC CGTTCGGTAG AACCTGGGTA ATTTGAAGTT TAAGGTACTA   
  
  
- ACTGATCCTG AACGGGTTTT TAAAGTAATG GGGTCTACGG GTGTACACTT ACGTCAGAGA CTGTCTCATC   
  
  
- CACCCTTATT TGGTTTTACG TAAATAAATA AAAAGTTCTA AAATTAATAA TAAGTGGATT TTAGGAAGTA   
  
  
- TCCCTAATTT TATAATCTGG TAACCTAAAC GAGAGGAGTT ATCCCAACTT GAAACTAATC ATGCAAATAT   
  
  
- AATAAAAAAA GGAAAAAGCA GTTTTACGTA AGACAGTGTT TTAATTACAT CAGTTCCTTA ACTGTTCACT   
  
  
- CTTCTCTCAG TGAAATTTAC TAAGTCGACA GGTGTAATTT CTATTCAGTG TAAACACCAG GTATGAGGGG   
  
  
- TTCTGAAATT AAACTTAAAT CGAGTCTGAA AAGCATAAAA TAAACACAAA AAGATAAATT CGATAGTTAT   
  
  
- GTAAATATTT ATTCACTAAA ATTATATTTT CTAGTTCATT TTTTGTTTAT TTCATGTATT TTATAAAAAT   
  
  
- AAGTTAATGT ATAACATAGT TTAAAAGTTC TCGTTTTAAA TATCGTAAAA AGTTACAAAA CCTTTAGTTT   
  
  
- TTTCATTATA TAATAGTCAA GTGATCAATT TAACCACTAA CTTGGTATCT ACTACAGTAT TATATATAAA   
  
  
- ATATAAATAA TTTTAATTTT TATAAATATT CTTTAAAATT TATATACTCT TTAATTTTCA GTTATTATAA   
  
  
- AAAAAGAAGT AGAAATTATA ATAGTTTACT TATATATATA TATATATTTA CATAACTATA AAATTTTAAA   
  
  
- ACAATTTATT TTATATATAT TATACTTCTA TAAATAACTT TCAATAAATT AAATGGATAT ATATATAATT   
  
  
- TTCAATAAAT ACCAACATTT TTTATAATGT AATAAAATAA ATATAATAAT TATAATGTAA TAAAATAAAT   
  
  
- ATAATAAATA TTAAAGTAAT AATATTTTTC AAACTTTTAT AATAACGATA TATTAAACAA CAACACACTT   
  
  
- CTAGCCGTTT TTTTGTCCTC ATCGTCACAC CACAGCAGGA ACGGGGAACT TGACCTCCAA CCAAAGGGTA   
  
  
- AGGAGGGAGG GTGCTTAGGG TGTACATTTC GTTTATTTTC TCGAAATGTA TGTGTACTAA TTACATAAAA   
  
  
- ATAACAGTTA TTTAAAATAT TATTTTGAAT ATTTAGAATA TTTAAATTAT CCTTTTTTTT TTCAGAAGAT   
  
  
- CTTAACTAAA ATATTATGTG GTATGGTTCT AGTGTACTTA ACGTACACAG CCACCTGTTC CAACGGTTTT   
  
  
- AAGGAATAAG GATATGTCTT AGAAAAGTAA CAATATAATA GTATCAGCTT TGGGTCATCA TATATTTCTC   
  
  
- CAATGCTCAC CTCCTACCTA TACTTTATTT ATTCTCCGGA ATCTCTAGTC CCACACTCTG TACCTAAACC   
  
  
- GTTTACCACT CTCTAGATCA CTCG

+     as-1

| Site Name | Organism | Position | Strand | Matrix score. | sequence | function |
| --- | --- | --- | --- | --- | --- | --- |
| as-1 | Arabidopsis thaliana | 2106 | + | 5 | TGACG |  |
| as-1 | Arabidopsis thaliana | 2608 | - | 5 | TGACG |  |

>PlantCARE\_32120   
+ AATCATTAAA GTCTAATTCT TCAAAATTCC GAATTCTAAA TTTAGCTATT TAAAATTCCA ATTGTCCTAT   
  
  
+ CTCCGAACTT AATTTTCAGT TTCCCATGCT CCAATTCCCG TATTTACCCC GCTACTTATT ATTATCATTA   
  
  
+ TCATTATCAC CTTATTCATT ATTTCTATAA ATTCCGCCTT CGAATGATTT CTAAGATCTC CAATTTTTAT   
  
  
+ ACATCAACTT TCATAATTTC TACGTCTCAA ATAACATACG ATTCTGATTC CTTTCAAAAT TTAACTCCCA   
  
  
+ TAATTCCAAT TTTCGTAACT TAATTTTTCT TAAAAATCCC GAACTTTCAA AATCCCAATT TTCTTTCAAA   
  
  
+ TTTAATTTCT AAATGTTCTC ATTCTTACAT CTAATTCCTA AGAATCCAAT TTTCAAATAA TCTCTAAGAT   
  
  
+ TCCGATTCTC AAATGAATTT CAAAAATCCA GTTTCCCTTC GAACAATTTT AATCCCTGTT TAGTGATGCA   
  
  
+ TGTAATATGT TTTGTGCTCT ATATGGTGTA CTAACCCTCT CTATTGATAG CGCATGGTGG TTCGTGGCCC   
  
  
+ AGGTATGTAC CCATTTTCTC TCTAATCGTT GTCTATGCAT GTCTCGATTC TTATGTGTGC ACGATCATTC   
  
  
+ AGGATATTCA TTGATTTACT CATCAATTGT CACGGCAGCT TCATTTTATT AGTAGAGACC CGACTTTAGG   
  
  
+ GACTTAGAGG GGTGCTACGG TCCTTACCGT ACCTTCCTGA TAAGTAACCT GACCCCCGAA CCCGATCCGG   
  
  
+ TTTTTCACAG ACCATCTTTT CCAAAATAAG GAGTCACACT TAGGGTTTTT CTTTCTTATT TTGTTTACCC   
  
  
+ TTTAAAAATA AAACAAAAAT AAGTGGCGAC TCCAAGTCAT TTTTCTTAAT CAATAAAGAT CATTTTTTCG   
  
  
+ AAAACGAAAA TCGAGCTCGC CATCGAGTGG GAAACGCATT GAGCCGAAAT GCGGGGTCCA TAGGTATGTT   
  
  
+ TTTTATTTAT GAGATAAAAA GTAAATAATA TTTTTTTCGG GTTTAAAAAA TATATTATTG ATTACTTGTA   
  
  
+ ATTATATGAG CCTCGTTTGT ACTTATTATT AAATACATAT TCAAGGTAAA ATTATCCTTT AAAAAATTCT   
  
  
+ TAGGCCGAGG GAACCTTCCT ATTAATATAA GTCATCCAGC CCACCTTATG GGGGTAATTC TCCCCATATA   
  
  
+ CCATGGGTCC AAATCGGCGG CCCGGTCAGT TCAACGACAG TACGATGGCA CTCTGAACCC TGTGCTGCAT   
  
  
+ CTGCTCACAA AAAGTACACC ACAGTTGTCT TTCACTCTCT CTGCCCATTC ACTATGCAAA CACGTTTATA   
  
  
+ AAATTGGTTA TTTTATAAAC GTTTCTCTTT CAAATGGTTA TTTTATATTA GTAACAAAAA TATTATAATA   
  
  
+ ATAAAAAAAT CAATATTACG TAATTGCTAA AAATGAAATT ACATCATGTT GTAAGATATT TCATTTGCCT   
  
  
+ ATTAATAATT TTTTATTATT TATAAAATTG GGTTGAATTT CAATATTTTT TAAAATAAGT CTTATTTATT   
  
  
+ TAAGTGAAAT TTTTTTTATA GATAAAGTCA CAAAAATCAT TTTTCTAAAT AATTTATAAA AAGATACTTT   
  
  
+ TCCAATAAAA ATTAAATTAA AATGCTTTTA TAAATAAAAT TGTAACAATT CATTCCATAG CAAAGGTAAA   
  
  
+ TAAAGCCATT TTCTTATCAA TTAAATCAAG CTTCTCTTTT TTATGAAATT GTTTTTAATA AATTCTTTTA   
  
  
+ GTAACAATAA GATTTTGTTC ATATTTTAAT ACATCTCCTT GGCAATTGGG CAAAGATGGC TGGTAGTGAC   
  
  
+ ATGAATCCAA GCATGAATTA GTCCGGGCGT TGTTACTTGC TACAAGGGTT ATGAGCATGG AAGGAAAAAT   
  
  
+ AATTAAAAAA TAAAAAAAAT TAAATTACTT CAATCACAAA GTTGAAAATA ATAAAATTGT GTTTGGAAGT   
  
  
+ ATTTGTACTT AAAGTTTTTT AGTAAAAGTG TTTCCCTAAA AGGTGTTTTT TTTAGAGAAA GCAGCTATAA   
  
  
+ AATGTTTTTT CAAAAATATT TTTTTATTTA AAAACACTTT CTTAAATCAC TTGTTATAAG TCACCTTAGA   
  
  
+ ATGACTGACG GTGTTGCACA TATCAAAAAG CATTTATGCC CTAGTGCCAT TTTATTATTT TATATCTTAA   
  
  
+ TTTTAGATAT TTTTATTTTT ATTTAATGAT TTATATTTGA ACGTTAATTT TCAATATATC ATATTGTATC   
  
  
+ TTTTATAGCC ACGTTTATTC AAATATATTT TTTATATTTA TAAATATTTT TTCTAAAATA TGATTCGAGA   
  
  
+ ATGCTGACAT GTGAATCGCA TGGGGGTAGG GCAAGCCATC TTGGACCCAT TAAACTTCAA ATTCCATGAT   
  
  
+ TGACTAGGAC TTGCCCAAAA ATTTCATTAC CCCAGATGCC CACATGTGAA TGCAGTCTCT GACAGAGTAG   
  
  
+ GTGGGAATAA ACCAAAATGC ATTTATTTAT TTTTCAAGAT TTTAATTATT ATTCACCTAA AATCCTTCAT   
  
  
+ AGGGATTAAA ATATTAGACC ATTGGATTTG CTCTCCTCAA TAGGGTTGAA CTTTGATTAG TACGTTTATA   
  
  
+ TTATTTTTTT CCTTTTTCGT CAAAATGCAT TCTGTCACAA AATTAATGTA GTCAAGGAAT TGACAAGTGA   
  
  
+ GAAGAGAGTC ACTTTAAATG ATTCAGCTGT CCACATTAAA GATAAGTCAC ATTTGTGGTC CATACTCCCC   
  
  
+ AAGACTTTAA TTTGAATTTA GCTCAGACTT TTCGTATTTT ATTTGTGTTT TTCTATTTAA GCTATCAATA   
  
  
+ CATTTATAAA TAAGTGATTT TAATATAAAA GATCAAGTAA AAAACAAATA AAGTACATAA AATATTTTTA   
  
  
+ TTCAATTACA TATTGTATCA AATTTTCAAG AGCAAAATTT ATAGCATTTT TCAATGTTTT GGAAATCAAA   
  
  
+ AAAGTAATAT ATTATCAGTT CACTAGTTAA ATTGGTGATT GAACCATAGA TGATGTCATA ATATATATTT   
  
  
+ TATATTTATT AAAATTAAAA ATATTTATAA GAAATTTTAA ATATATGAGA AATTAAAAGT CAATAATATT   
  
  
+ TTTTTCTTCA TCTTTAATAT TATCAAATGA ATATATATAT ATATATAAAT GTATTGATAT TTTAAAATTT   
  
  
+ TGTTAAATAA AATATATATA ATATGAAGAT ATTTATTGAA AGTTATTTAA TTTACCTATA TATATATTAA   
  
  
+ AAGTTATTTA TGGTTGTAAA AAATATTACA TTATTTTATT TATATTATTA ATATTACATT ATTTTATTTA   
  
  
+ TATTATTTAT AATTTCATTA TTATAAAAAG TTTGAAAATA TTATTGCTAT ATAATTTGTT GTTGTGTGAA   
  
  
+ GATCGGCAAA AAAACAGGAG TAGCAGTGTG GTGTCGTCCT TGCCCCTTGA ACTGGAGGTT GGTTTCCCAT   
  
  
+ TCCTCCCTCC CACGAATCCC ACATGTAAAG CAAATAAAAG AGCTTTACAT ACACATGATT AATGTATTTT   
  
  
+ TATTGTCAAT AAATTTTATA ATAAAACTTA TAAATCTTAT AAATTTAATA GGAAAAAAAA AAGTCTTCTA   
  
  
+ GAATTGATTT TATAATACAC CATACCAAGA TCACATGAAT TGCATGTGTC GGTGGACAAG GTTGCCAAAA   
  
  
+ TTCCTTATTC CTATACAGAA TCTTTTCATT GTTATATTAT CATAGTCGAA ACCCAGTAGT ATATAAAGAG   
  
  
+ GTTACGAGTG GAGGATGGAT ATGAAATAAA TAAGAGGCCT TAGAGATCAG GGTGTGAGAC ATGGATTTGG   
  
  
+ CAAATGGTGA GAGATCTAGT GAGC  

- TTAGTAATTT CAGATTAAGA AGTTTTAAGG CTTAAGATTT AAATCGATAA ATTTTAAGGT TAACAGGATA   
  
  
- GAGGCTTGAA TTAAAAGTCA AAGGGTACGA GGTTAAGGGC ATAAATGGGG CGATGAATAA TAATAGTAAT   
  
  
- AGTAATAGTG GAATAAGTAA TAAAGATATT TAAGGCGGAA GCTTACTAAA GATTCTAGAG GTTAAAAATA   
  
  
- TGTAGTTGAA AGTATTAAAG ATGCAGAGTT TATTGTATGC TAAGACTAAG GAAAGTTTTA AATTGAGGGT   
  
  
- ATTAAGGTTA AAAGCATTGA ATTAAAAAGA ATTTTTAGGG CTTGAAAGTT TTAGGGTTAA AAGAAAGTTT   
  
  
- AAATTAAAGA TTTACAAGAG TAAGAATGTA GATTAAGGAT TCTTAGGTTA AAAGTTTATT AGAGATTCTA   
  
  
- AGGCTAAGAG TTTACTTAAA GTTTTTAGGT CAAAGGGAAG CTTGTTAAAA TTAGGGACAA ATCACTACGT   
  
  
- ACATTATACA AAACACGAGA TATACCACAT GATTGGGAGA GATAACTATC GCGTACCACC AAGCACCGGG   
  
  
- TCCATACATG GGTAAAAGAG AGATTAGCAA CAGATACGTA CAGAGCTAAG AATACACACG TGCTAGTAAG   
  
  
- TCCTATAAGT AACTAAATGA GTAGTTAACA GTGCCGTCGA AGTAAAATAA TCATCTCTGG GCTGAAATCC   
  
  
- CTGAATCTCC CCACGATGCC AGGAATGGCA TGGAAGGACT ATTCATTGGA CTGGGGGCTT GGGCTAGGCC   
  
  
- AAAAAGTGTC TGGTAGAAAA GGTTTTATTC CTCAGTGTGA ATCCCAAAAA GAAAGAATAA AACAAATGGG   
  
  
- AAATTTTTAT TTTGTTTTTA TTCACCGCTG AGGTTCAGTA AAAAGAATTA GTTATTTCTA GTAAAAAAGC   
  
  
- TTTTGCTTTT AGCTCGAGCG GTAGCTCACC CTTTGCGTAA CTCGGCTTTA CGCCCCAGGT ATCCATACAA   
  
  
- AAAATAAATA CTCTATTTTT CATTTATTAT AAAAAAAGCC CAAATTTTTT ATATAATAAC TAATGAACAT   
  
  
- TAATATACTC GGAGCAAACA TGAATAATAA TTTATGTATA AGTTCCATTT TAATAGGAAA TTTTTTAAGA   
  
  
- ATCCGGCTCC CTTGGAAGGA TAATTATATT CAGTAGGTCG GGTGGAATAC CCCCATTAAG AGGGGTATAT   
  
  
- GGTACCCAGG TTTAGCCGCC GGGCCAGTCA AGTTGCTGTC ATGCTACCGT GAGACTTGGG ACACGACGTA   
  
  
- GACGAGTGTT TTTCATGTGG TGTCAACAGA AAGTGAGAGA GACGGGTAAG TGATACGTTT GTGCAAATAT   
  
  
- TTTAACCAAT AAAATATTTG CAAAGAGAAA GTTTACCAAT AAAATATAAT CATTGTTTTT ATAATATTAT   
  
  
- TATTTTTTTA GTTATAATGC ATTAACGATT TTTACTTTAA TGTAGTACAA CATTCTATAA AGTAAACGGA   
  
  
- TAATTATTAA AAAATAATAA ATATTTTAAC CCAACTTAAA GTTATAAAAA ATTTTATTCA GAATAAATAA   
  
  
- ATTCACTTTA AAAAAAATAT CTATTTCAGT GTTTTTAGTA AAAAGATTTA TTAAATATTT TTCTATGAAA   
  
  
- AGGTTATTTT TAATTTAATT TTACGAAAAT ATTTATTTTA ACATTGTTAA GTAAGGTATC GTTTCCATTT   
  
  
- ATTTCGGTAA AAGAATAGTT AATTTAGTTC GAAGAGAAAA AATACTTTAA CAAAAATTAT TTAAGAAAAT   
  
  
- CATTGTTATT CTAAAACAAG TATAAAATTA TGTAGAGGAA CCGTTAACCC GTTTCTACCG ACCATCACTG   
  
  
- TACTTAGGTT CGTACTTAAT CAGGCCCGCA ACAATGAACG ATGTTCCCAA TACTCGTACC TTCCTTTTTA   
  
  
- TTAATTTTTT ATTTTTTTTA ATTTAATGAA GTTAGTGTTT CAACTTTTAT TATTTTAACA CAAACCTTCA   
  
  
- TAAACATGAA TTTCAAAAAA TCATTTTCAC AAAGGGATTT TCCACAAAAA AAATCTCTTT CGTCGATATT   
  
  
- TTACAAAAAA GTTTTTATAA AAAAATAAAT TTTTGTGAAA GAATTTAGTG AACAATATTC AGTGGAATCT   
  
  
- TACTGACTGC CACAACGTGT ATAGTTTTTC GTAAATACGG GATCACGGTA AAATAATAAA ATATAGAATT   
  
  
- AAAATCTATA AAAATAAAAA TAAATTACTA AATATAAACT TGCAATTAAA AGTTATATAG TATAACATAG   
  
  
- AAAATATCGG TGCAAATAAG TTTATATAAA AAATATAAAT ATTTATAAAA AAGATTTTAT ACTAAGCTCT   
  
  
- TACGACTGTA CACTTAGCGT ACCCCCATCC CGTTCGGTAG AACCTGGGTA ATTTGAAGTT TAAGGTACTA   
  
  
- ACTGATCCTG AACGGGTTTT TAAAGTAATG GGGTCTACGG GTGTACACTT ACGTCAGAGA CTGTCTCATC   
  
  
- CACCCTTATT TGGTTTTACG TAAATAAATA AAAAGTTCTA AAATTAATAA TAAGTGGATT TTAGGAAGTA   
  
  
- TCCCTAATTT TATAATCTGG TAACCTAAAC GAGAGGAGTT ATCCCAACTT GAAACTAATC ATGCAAATAT   
  
  
- AATAAAAAAA GGAAAAAGCA GTTTTACGTA AGACAGTGTT TTAATTACAT CAGTTCCTTA ACTGTTCACT   
  
  
- CTTCTCTCAG TGAAATTTAC TAAGTCGACA GGTGTAATTT CTATTCAGTG TAAACACCAG GTATGAGGGG   
  
  
- TTCTGAAATT AAACTTAAAT CGAGTCTGAA AAGCATAAAA TAAACACAAA AAGATAAATT CGATAGTTAT   
  
  
- GTAAATATTT ATTCACTAAA ATTATATTTT CTAGTTCATT TTTTGTTTAT TTCATGTATT TTATAAAAAT   
  
  
- AAGTTAATGT ATAACATAGT TTAAAAGTTC TCGTTTTAAA TATCGTAAAA AGTTACAAAA CCTTTAGTTT   
  
  
- TTTCATTATA TAATAGTCAA GTGATCAATT TAACCACTAA CTTGGTATCT ACTACAGTAT TATATATAAA   
  
  
- ATATAAATAA TTTTAATTTT TATAAATATT CTTTAAAATT TATATACTCT TTAATTTTCA GTTATTATAA   
  
  
- AAAAAGAAGT AGAAATTATA ATAGTTTACT TATATATATA TATATATTTA CATAACTATA AAATTTTAAA   
  
  
- ACAATTTATT TTATATATAT TATACTTCTA TAAATAACTT TCAATAAATT AAATGGATAT ATATATAATT   
  
  
- TTCAATAAAT ACCAACATTT TTTATAATGT AATAAAATAA ATATAATAAT TATAATGTAA TAAAATAAAT   
  
  
- ATAATAAATA TTAAAGTAAT AATATTTTTC AAACTTTTAT AATAACGATA TATTAAACAA CAACACACTT   
  
  
- CTAGCCGTTT TTTTGTCCTC ATCGTCACAC CACAGCAGGA ACGGGGAACT TGACCTCCAA CCAAAGGGTA   
  
  
- AGGAGGGAGG GTGCTTAGGG TGTACATTTC GTTTATTTTC TCGAAATGTA TGTGTACTAA TTACATAAAA   
  
  
- ATAACAGTTA TTTAAAATAT TATTTTGAAT ATTTAGAATA TTTAAATTAT CCTTTTTTTT TTCAGAAGAT   
  
  
- CTTAACTAAA ATATTATGTG GTATGGTTCT AGTGTACTTA ACGTACACAG CCACCTGTTC CAACGGTTTT   
  
  
- AAGGAATAAG GATATGTCTT AGAAAAGTAA CAATATAATA GTATCAGCTT TGGGTCATCA TATATTTCTC   
  
  
- CAATGCTCAC CTCCTACCTA TACTTTATTT ATTCTCCGGA ATCTCTAGTC CCACACTCTG TACCTAAACC   
  
  
- GTTTACCACT CTCTAGATCA CTCG
